# Effects of T-Type Calcium Channel Blockers on Renal Function and Aldosterone in Patients with Hypertension: A Systematic Review and Meta-Analysis

**DOI:** 10.1371/journal.pone.0109834

**Published:** 2014-10-17

**Authors:** Xue Li, Mao Sheng Yang

**Affiliations:** Department of Pharmacology, College of Pharmacy, Chongqing Medical University, Chongqing, People’s Republic of China; Osaka University Graduate School of Medicine, Japan

## Abstract

**Background:**

High blood pressure can cause kidney damage, which can increase blood pressure, leading to a vicious cycle. It is not clear whether the protective effects of T-type calcium channel blockers (T-type CCBs) on renal function are better than those of L-type CCBs or renin-angiotensin system (RAS) antagonists in patients with hypertension.

**Methods and Findings:**

PUBMED, MEDLINE, EMBASE, OVID, Web of Science, Cochrane, CNKI, MEDCH, VIP, and WANFANG databases were searched for clinical trials published in English or Chinese from January 1, 1990, to December 31, 2013. The weighted mean difference (WMD) and 95% confidence interval (*CI*) were calculated and reported. A total of 1494 reports were collected, of which 24 studies with 1,696 participants (including 809 reports comparing T-type CCBs versus L-type CCBs and 887 reports comparing T-type CCB versus RAS antagonists) met the inclusion criteria. Compared with L-type CCBs, T-type CCBs resulted in a significant decline in aldosterone (mean difference = −15.19, 95% *CI* −19.65–−10.72, p<1×10^−5^), proteinuria (mean difference = −0.73, 95% *CI* −0.88–−0.57, p<1×10^−5^), protein to creatinine ratio (mean difference = −0.22, 95% *CI* −0.41–−0.03, p = 0.02), and urinary albumin to creatinine ratio (mean difference = −55.38, 95% *CI* −86.67–*−*24.09, p = 0.0005); no significant difference was noted for systolic blood pressure (SBP) (p = 0.76) and diastolic blood pressure (DBP) (p = 0.16). The effects of T-type CCBs did not significantly differ from those of RAS antagonists for SBP (p = 0.98), DBP (p = 0.86), glomerular filtration rate (p = 0.93), albuminuria (p = 0.97), creatinine clearance rate (p = 0.24), and serum creatinine (p = 0.27) in patients with hypertension.

**Conclusion:**

In a pooled analysis of data from 24 studies measuring the effects of T-type CCBs on renal function and aldosterone, the protective effects of T-type CCBs on renal function were enhanced compared with L-type CCBs but did not differ from RAS antagonists. Their protective effects on renal function were independent of blood pressure.

## Introduction

It is well known that long-term high blood pressure (HBP) can cause kidney damage and that kidney damage can increase blood pressure, thereby leading to a vicious cycle. HBP control might aid in the prevention of kidney damage. Calcium channel blockers (CCB) are a widely used antihypertensive agent. Several studies indicate that T-type calcium channel blockers (T-type CCBs) are better than L-type CCBs at reducing glomerular pressure and protecting the kidneys [1]–[3]. The reduction of glomerular pressure is a principal strategy for reducing proteinuria in hypertensive patients [4]. To decrease glomerular pressure, HBP and arteriolar resistance in efferent arterioles must first be effectively controlled [5]–[6]. Renin-angiotensin system (RAS) antagonists play an important role in blood pressure and renal function. Angiotensin II type 1 receptors are localized in both afferent and efferent arterioles [7], and angiotensin II receptor blockers (ARBs) [8] and angiotensin-converting enzyme inhibitors (ACEIs) [9] reduce proteinuria. However, the “aldosterone escape” might emerge after administration of ARBs or ACEIs, and high plasma concentrations of aldosterone can aggravate kidney vascular injury, glomerular sclerosis, and kidney interstitial fibrosis and reduce the therapeutic effects of antihypertensive agents [10].

Due to the above factors, only a limited number of independent studies are available. Thus, it is difficult to establish the beneficial effects of T-type CCBs, L-type CCBs, or RAS antagonists on renal function and aldosterone from individual studies. Hence, a systematic review and meta-analysis might aid in the clarification of this issue and determine whether the protective effects of T-type CCBs on renal function are more effective than L-type CCBs or RAS antagonists. The major aim of the present study was to evaluate the effects of antihypertensive agents on the protection of renal function and aldosterone.

## Methods

### Data Sources

Studies were identified by searches of PUBMED, MEDLINE, EMBASE, OVID, Web of Science, Cochrane, CNKI, MEDCH, VIP and WANFANG databases for relevant articles published in English or Chinese during the period from January 1, 1990, to December 31, 2013. In addition, the bibliographies of relevant studies, review articles, and meta-analyses were also considered to identify additional works not indexed by the above databases. The search terms included “L-type calcium channel blockers”; “T-type calcium channel blockers”; “calcium channel blockers” or “CCB”; “renin-angiotensin system antagonists” or “RAS antagonists”; “ARB”; “ACEI”; “efonidipine”; “azelnidipine”; “benidipine”; “manidipine”; “nilvadipine”; “glomerular filtration rate”, “GFR” or “eGFR”; “proteinuria” or “urinary protein”; “albuminuria” or “urinary albumin”; “microalbuminuria”; “creatinine”; “aldosterone” or “plasma aldosterone concentration”; and “kidney”, “renal” or “nephropathy”.

### Study Selection

**Figure 1:**
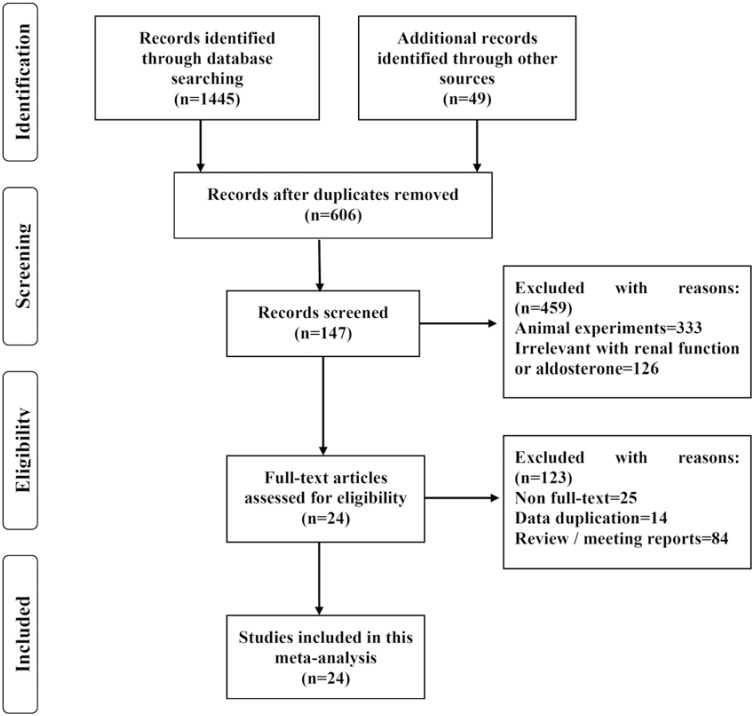
A schematic diagram of the search strategy for published reports.

A total of 1494 published studies were identified using the screening procedure presented in Figure 1. Among these studies, 1445 records were identified through database searching, and forty-nine reports were identified from other sources. After searching, the following information was extracted: author, year of publication, ethnicity of research subjects, number of patients, medicine(s) used in treatment, age of patients, and duration of follow-up. Studies were eligible for inclusion if they were randomized controlled trials or comparative studies that reported renal function or plasma aldosterone associated with the current use of T-type calcium channel blockers in population settings.

### Quality Assessment

Studies were included if they met the following criteria: (1) contained original data (if multiple studies used overlapping subjects, only the study with the bigger/biggest sample size was used); (2) contained data regarding renal function or plasma aldosterone; (3) contained data regarding T-type calcium channel blockers and/or L-type calcium channel blockers or renin-angiotensin system antagonists, and CCBs or RAS antagonists were separately administered as a primary drug; and (4) randomized controlled trials (RCTs) or comparative studies involving participants 18 years or older.

Studies were excluded for the following reasons: (1) not associated with renal function and aldosterone; (2) involved animal experiments; (3) data duplication; (4) not written in English or Chinese; (5) missing or insufficient data; (6) no control group; or (7) not an original study. Two investigators independently extracted all of the information, and no inconsistencies were noted. The quality and overall risk of bias of each included study were evaluated. We also conducted sensitivity analyses in which the pooled WMD was recalculated by omitting one study at a time.

### Statistical Analysis

To compare the effects of T-type CCBs, L-type CCBs, and RAS antagonists on systolic blood pressure (SBP), diastolic blood pressure (DBP), glomerular filtration rate (GFR), creatinine clearance rate (CCr), serum creatinine (SCr), proteinuria, albuminuria, aldosterone, the weighted mean difference (WMD) and its 95% confidence interval (*CI*) were calculated and reported. Publication bias was detected by Egger’s linear regression test, which measures funnel plot asymmetry on the scale of mean differences (MD). As described in detail previously [11], the statistical tests were conducted using the GRADEprofiler version 3.2.2 (The GRADE Working Group, http://www.gradeworkinggroup.org/index.htm), RevMan version 5.0 (The Cochrane collaboration, Oxford, England) and Origin 8.6 statistical software (OriginLab Corporation, Northampton, USA). A *p*-value of less than 0.05 was considered as statistically significant.

## Results

The derivation of the databases and published articles is described in Figure 1. A total of 1494 studies were identified. Among these studies, twenty-four studies [12]–[35] with a total of 1,696 participants (including 809 studies assessing T-type CCBs versus L-type CCBs and 887 studies assessing T-type CCBs versus RAS antagonists) met the inclusion criteria and were selected for the statistical test (see Table 1). Six articles [13], [16], [18], [20], [21], [22] lacked data regarding renal function or plasma aldosterone. Therefore, we contacted the authors to ask for additional information, but only one [22] replied. The remaining five [13], [16], [18], [20], [21] authors did not respond. The age of patients in the experimental and control groups are well matched in each study (see Table 1); the influence of age on the parameters associated with renal function, such as GFR and SCr, can be excluded.

**Table 1:** Characteristics of twenty-four studies included in the meta-analysis.

Sourceyear(reference)	Ethnicity	Treatment	Cases ofPatient		Age of Cases (Mean±SD)		Time offollowed-up(month)	Quality of theevidence(GRADE)	Overall risk of biasassessment(RevMan)
			T-type CCBs (Male)	L-type CCBs or RAS antagonists (Male)	Experimental	Control			
**T-type CCBs vs L-type CCBs**									
Tadashi Konoshita 2013[12] *	Asia	Efonidipine vs Amlodipine	50(22)	50(22)	69.8±10.8	69.8±10.8	3	Moderate	Unclear
Tadashi Konoshita 2013[12] *	Asia	Efonidipine vs Nifedipine	50(22)	50(22)	69.8±10.8	69.8±10.8	3	Moderate	Unclear
Tsuneo Takenaka 2012[13] *	Asia	Azelnidipine vs Amlodipine	29(18)	30(18)	66±2	67±2	12	High	Unclear
Tsukasa Nakamura 2011[14]	Asia	Azelnidipine vs Amlodipine	15(9)	15(9)	45.3±9.6	45.5±8.8	6	High	Unclear
Masanori Abe 2011[15] *	Asia	Benidipine vs Amlodipine	52(30)	52(30)	67.3±1.4	67.5±1.5	6	High	High
Masanori Abe 2011.6[16]	Asia	Azelnidipine vs Amlodipine	34(21)	33(20)	65.8±1.7	66.0±1.4	6	High	Unclear
Nobuyuki Nakano 2010[17]	Asia	Efonidipine vs Amlodipine	20(11)	20(11)	66.8±10.1	66.8±10.1	3	High	Low
Tsukasa Nakamura 2010[18]	Asia	Benidipine vs Amlodipine	20(11)	20(11)	33.5±7.0	31.6±5.3	12	High	Low
Takayoshi Tsutamoto 2009[19]	Asia	Efonidipine vs Amlodipine	30(17)	30(16)	64.1±10.5	63.8±8.1	18	High	Low
Hidehisa Sasaki 2009[20]	Asia	Efonidipine vs Amlodipine	20(14)	20(12)	63.3±2.5	65.5±3.0	12	High	High
Masanori Abe 2009[21]	Asia	Benidipine vs Amlodipine	24(15)	23(15)	65.9±2.2	65.5±2.1	6	High	High
Martinez Martin 2008[22] *	Europe	Manidipine vs Amlodipine	61(24)	30(13)	56.9±13.3	55.8±12.7	24	Moderate	Unclear
Toshinari Tanaka 2007[23]	Asia	Efonidipine vs Amlodipine	40(27)	40(27)	67.4±1.0	67.4±1.0	6	Moderate	High
Tsukasa Nakamura 2007[24]	Asia	Azelnidipine vs Amlodipine	15(8)	15(7)	48±16	46±14	6	High	Unclear
Toshihiko Ishimitsu 2007[25]	Asia	Efonidipine vs Amlodipine	21(16)	21(16)	54±13	54±13	4	High	Low
Tetsuya Oshima 2005[26]	Asia	Efonidipine vs Nifedipine	20(13)	20(13)	55±11	55±11	3	High	Low
Hajime Ueshiba 2004[27]	Asia	Manidipine vs Amlodipine	10(5)	10(5)	55.3±9.2	57.4±6.6	6	High	Low
Guido Bellinghieri 2003[28] *	Europe	Manidipine vs Nifedipine	48(37)	50(38)	50.7±11.9	51.3±10.9	3	Moderate	Unclear
**T-type CCBs vs RAS antagonists**									
Rong Qi Han 2013[29]	Asia	Benidipine vs benazepril	40(29)	40(25)	48.6±6.8	48.6±6.8	3	High	Unclear
Ming Lian Gong 2012[30]	Asia	Benidipine vs valsartan	45(28)	45(28)	53.0±6.5	53.0±6.5	6	High	Unclear
Jian Sheng Gan 2012[31]	Asia	Benidipine vs valsartan	143(87)	143(88)	64.5±5.6	64.5±5.6	6	High	Unclear
Bo Dong 2011[32]	Asia	Benidipine vs perindopril	30(19)	30(18)	68.9±3.7	69.3±3.5	12	High	Unclear
Tao Peng 2009[33]	Asia	Benidipine vs Valsartan	118(61)	118(61)	43.2±9.5	43.2±9.5	12	High	High
Lucia Del Vecchio 2004[34] *	Europe	Manidipine vs Enalapril	67(49)	64(42)	52.9±10.5	56.4±10.0	12	Moderate	Unclear
Koichi Hayashi 2003[35] *	Asia	Efonidipine vs ACEI	23(18)	20(12)	58±3	57±3	12	Moderate	Unclear

CCBs: Calcium Channel Blockers; RAS: Renin-angiotensin system.

*Some patients were lost to follow-up or withdrew, and the rate of lost to follow-up was not significantly different between the two groups.

GRADE Working Group grades of evidence. High quality: Further research is very unlikely to change our confidence in the estimate of effect. Moderate quality: Further research is likely to have an important impact on our confidence in the estimate of effect and may change the estimate. Low quality: Further research is very likely to have an important impact on our confidence in the estimate of effect and is likely to change the estimate. Very low quality: We are very uncertain about the estimate.

The risk of bias assessment is done using RevMan. Low risk of bias: Plausible bias unlikely to seriously alter the results, low risk of bias for all key domains (within a study), and most information is from studies at low risk of bias (across studies). Unclear risk of bias: That raises some doubt about the results, unclear risk of bias for one or more key domains (within a study), and most information is from studies at low or unclear risk of bias (across studies). High risk of bias: Plausible bias that seriously weakens confidence in the results, high risk of bias for one or more key domains (within a study), the proportion of information from studies at high risk of bias is sufficient to affect the interpretation of results (across studies).

The quantity and quality of original investigations play a significant role in determining the quality of the meta-analysis. To control for publication bias, the funnel test was performed (see Figure S1). No evidence of publication bias was identified in the included twenty-four studies. According to the results from the Cochran’s Q-statistic test and *I* ^2^ analysis, the heterogeneity between studies was not statistical significance (*I* ^2^ less than 50%, *p*>0.05). Therefore, the fixed effects model was used for the meta-analysis. However, for DBP and SCr in the T-type CCB vs. RAS groups, the *I* ^2^ value was greater than 50%; hence, the random effects model was used. The results of quality assessment for each included study indicated that eighteen reports [13]–[21], [24]–[27], [29]–[33] were high quality and that the remaining six studies [12], [22]–[23], [28], [34]–[35] were moderate quality (see Table 1 and Table S1). The overall quality of the evidence was high in our statistical tests. The results from the overall risk of bias assessment for each included study indicated that six reports [17]–[19], [25]–[27] exhibited a low risk of bias, thirteen reports [12]–[14], [16], [22], [24], [28]–[32], [34]–[35] exhibited an unclear risk of bias, and the remaining five studies [15], [20]–[21], [23], [33] exhibited a high risk of bias (see Table 1, Figure S2 and Table S2).

The issue of patient loss to follow-up or withdrawal was identified in the following studies. Two studies [17], [26] reported that no patients were lost to follow-up or withdrew. Fifteen studies [14], [16], [18]–[21], [23]–[25], [27], [29]–[33] did not report information regarding patient follow-up or withdrawal. The remaining seven studies [12], [13], [15], [22], [28], [34], [35] reported that some patients were lost to follow-up or withdrew and provided the reasons; the rate of loss to follow-up did not significantly differ between the experimental and control groups (see Table 1, Figure S2 and Table S2). Hence, we did not compare the incidences of withdrawals due to adverse effects among the different treatment groups because it would likely result in bias. Several reports [13], [16], [18], [20], [21], [23], [24], [27] used figures to present results, so the raw data were re-extracted using the Origin 8.6 program. We also attempted to contact the authors of the included twenty-four studies. The authors of seven reports could not be contacted, and the authors of ten reports did not respond. The authors of one report provided the information requested. The authors of six reports responded but did not provide the information we requested. Therefore, we could not perform other sub-group analyses.

### Comparison of Protective Effects on Renal Function between T-type CCBs and L-type CCBs

#### Systolic blood pressure

**Figure 2:**
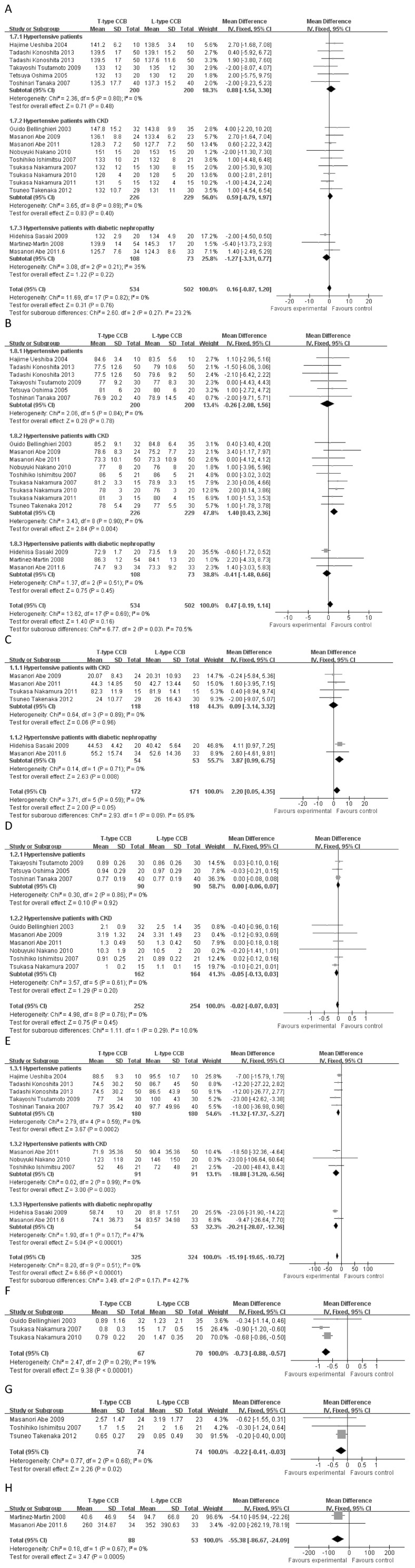
Mean differences and 95% *CIs* of included studies and pooled data for T-type CCBs versus L-type CCBs. (A) Systolic blood pressure (SBP). (B) Diastolic blood pressure (DBP). (C) Glomerular filtration rate (GFR). (D) Serum creatinine (SCr). (E) Aldosterone. (F) Proteinuria in hypertensive patients with CKD. (G) The urinary protein to creatinine ratio in hypertensive patients with CKD. (H) The urinary albumin to creatinine ratio in hypertensive patients with diabetic nephropathy.

Seventeen independent reports with 534 experimental subjects and 502 controls were included [12]–[28]. No significant difference was noted for SBP (MD = 0.16, 95% *CI* −0.87–1.20, *p* = 0.76) between T-type CCBs and L-type CCBs (see Figure 2-A).

#### Diastolic blood pressure

Seventeen reports with 534 experimental subjects and 502 controls were included in this meta-analysis [12]–[28]. No significant difference was noted for DBP in the overall-test (MD = 0.47, 95% *CI* −0.19–1.14, *p* = 0.16) between T-type CCBs and L-type CCBs. However, in subgroup containing hypertensive patients with CKD, L-type CCBs resulted in a significant decline in DBP (MD = 1.40, 95% *CI* 0.43–2.36, *p* = 0.004) (see Figure 2-B).

#### Glomerular filtration rate

Six studies were included [13]–[16], [20]–[21], consisting of 172 experimental subjects and 171 controls. In the subgroup containing hypertensive patients with diabetic nephropathy, the GFR was significantly increased (MD = 3.87, 95% *CI* 0.99–6.75, *p* = 0.008) with T-type CCBs compared with L-type CCBs. In the subgroup containing hypertensive patients with CKD, the GFR did not significantly differ between T-type CCBs and L-type CCBs (MD = 0.09, 95% *CI* −3.14–3.32, *p* = 0.96), and overall statistical analysis revealed that the GFR also did not significantly differ between T-type CCBs and L-type CCBs (MD = 2.20, 95% *CI* 0.05–4.35, *p* = 0.05) (see Figure 2-C).

#### Serum creatinine

Nine studies were included [15], [17], [19], [21], [23], [24]–[26], [28], consisting of 252 experimental subjects and 254 controls. No statistically significant differences were observed for the SCr concentrations in the overall (*p* = 0.45) and subgroup analysis (*p*≥0.20) between T-type CCBs and L-type CCBs (see Figure 2-D).

#### Plasma aldosterone concentration

Nine reports with 325 experimental subjects and 324 controls were included in this meta-analysis [12], [15]–[17], [19]–[20], [23], [25], [27]. Compared with L-type CCBs, T-type CCBs significantly decreased plasma aldosterone concentrations in the overall-test (mean difference = −15.19, 95% *CI* −19.65–−10.72, *p*<1×10^−5^), in the hypertensive patient subgroup (MD = −11.32, 95% *CI* −17.37–*−*5.27, *p* = 0.0002), the hypertensive patient with CKD subgroup (MD = −18.88, 95% *CI* −31.20–−6.56, *p* = 0.003), and the hypertensive patients with diabetic nephropathy subgroup (mean difference = −20.21, 95% *CI −*28.07–−12.36, *p*<1×10^−5^) (see Figure 2-E).

#### Proteinuria

Three studies were included [18], [24], [28], with a total of 67 experimental subjects and 70 controls. Compared with L-type CCBs, T-type CCBs resulted in an obvious decline in proteinuria (MD = −0.73, 95% *CI* −0.88–−0.57, *p*<1×10^−5^) in hypertensive patients with CKD (see Figure 2-F).

#### Protein to creatinine ratio

Three studies were included [13], [21], [25], consisting of 74 experimental subjects and 74 controls. Compared with L-type CCBs, T-type CCBs resulted in a significant decline in the protein to creatinine ratio (mean difference = −0.22, 95% *CI* −0.41–−0.03, *p* = 0.02) in hypertensive patients with CKD (see Figure 2-G).

#### Albumin to creatinine ratio

Two independent reports with 88 experimental subjects and 53 controls were included in this meta-analysis [16], [22]. Compared with L-type CCBs, T-type CCBs resulted in an obvious decline in the urinary albumin to creatinine ratio (mean difference = −55.38, 95% *CI* −86.67–−24.09, *p* = 0.0005) in hypertensive patients with diabetic nephropathy (see Figure 2-H).

### Comparison of Protective Effects on Renal Function between T-type CCBs and RAS antagonists

#### Systolic blood pressure

**Figure 3:**
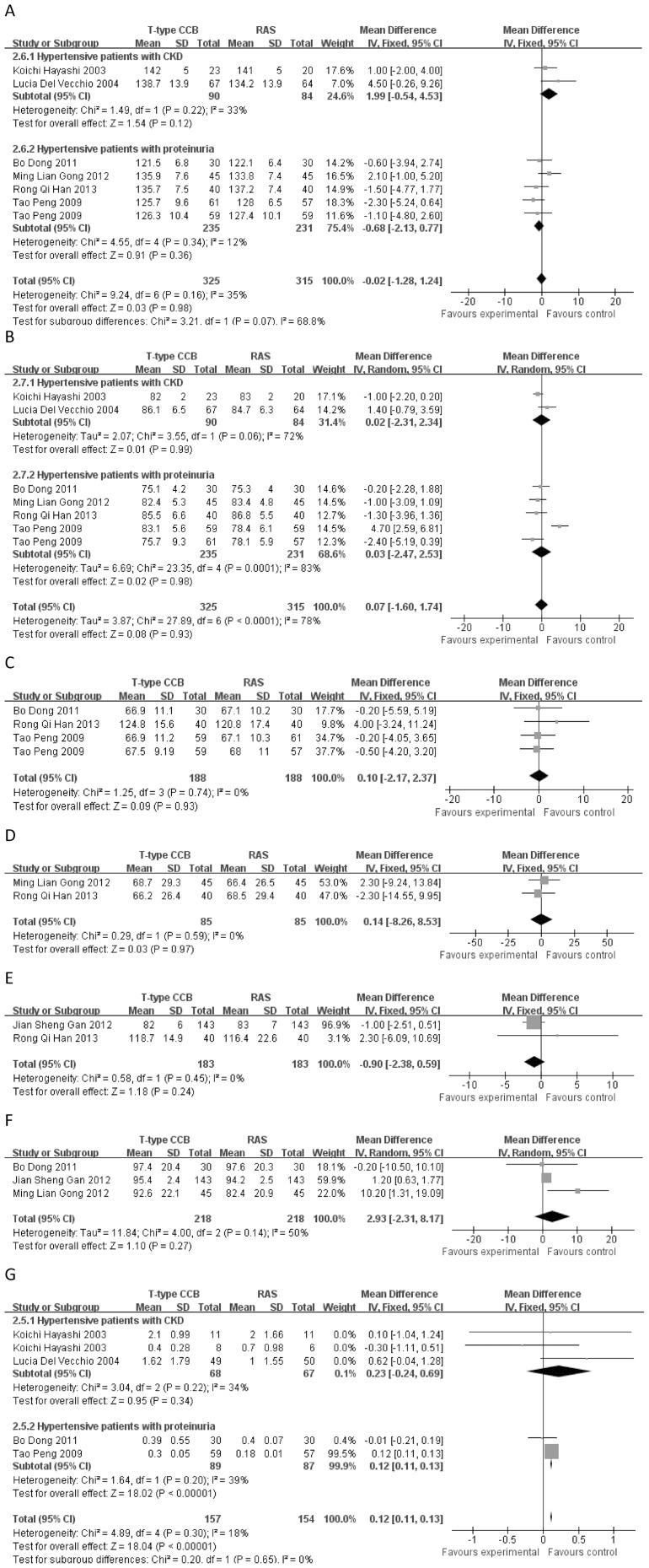
Mean differences and 95% *CIs* of included studies and pooled data for T-type CCBs versus RAS antagonists. (A) Systolic blood pressure (SBP). (B) Diastolic blood pressure (DBP). (C) The glomerular filtration rate (GFR) in hypertensive patients with proteinuria. (D) Albuminuria in hypertensive patients with proteinuria. (E) The creatinine clearance rate (CCr) in hypertensive patients with proteinuria. (F) Serum creatinine (SCr) in hypertensive patients with proteinuria. (G) Proteinuria.

Six independent reports with 325 experimental subjects and 315 controls were included [29]–[30], [32]–[35]. No significant difference in SBP was observed (mean difference = −0.02, 95% *CI* −1.28–1.24, *p* = 0.98) between T-type CCBs and RAS antagonists (see Figure 3-A).

#### Diastolic blood pressure

Six independent reports with 325 experimental subjects and 315 controls were included [29]–[30], [32]–[35]. No significant difference in DBP was observed (mean difference = −0.06, 95% *CI* −0.80–0.67, *p* = 0.86) between T-type CCBs and RAS antagonists (see Figure 3-B).

#### Glomerular filtration rate

Three studies were included [29], [32], [33], consisting of 188 experimental subjects and 188 controls. The GFR did not significantly differ (mean difference = 0.10, 95% *CI* −2.17–2.37, *p* = 0.93) between T-type CCBs and RAS antagonists (see Figure 3-C).

#### Albuminuria

Two studies were included [29]–[30], with a total of 85 experimental subjects and 85 controls. No significant difference in albuminuria was noted (mean difference = 0.14, 95% *CI* −8.26–8.53, *p* = 0.97) between T-type CCBs and RAS antagonists (see Figure 3-D).

#### Creatinine clearance rate

Two independent reports with 183 experimental subjects and 183 controls were included [29], [31]. No significant difference in CCr was observed (mean difference = −0.90, 95% *CI* −2.38–0.59, *p* = 0.24) between T-type CCBs and RAS antagonists (see Figure 3-E).

#### Serum creatinine

Three studies were included [30]–[32], with a total of 218 experimental subjects and 218 controls. No significant difference in SCr was observed (mean difference = 2.93, 95% *CI* −2.31–8.17, *p* = 0.27) between T-type CCBs and RAS antagonists (see Figure 3-F).

#### Proteinuria

Four independent reports with 157 experimental subjects and 154 controls were included [32]–[35]. The overall test revealed that RAS antagonists resulted in an obvious decline in proteinuria (mean difference = 0.12, 95% *CI* 0.11–0.13, *p*<1×10^−5^) compared with T-type CCBs. However, in the hypertensive patients with CKD subgroup, proteinuria did not significantly differ (mean difference = 0.23, 95% *CI* −0.24–0.69, *p* = 0.34) between T-type CCBs and RAS antagonists (see Figure 3-G).

#### Sensitivity Analyses

Sensitivity analyses were conducted using RevMan 5.0. The primary results were not influenced by the use of the fixed-effect or random-effect models, the loss to follow-up, or omission of one study at a time (see File S1).

## Discussion

The kidney is a vital organ for blood pressure regulation. Long-term high blood pressure can cause kidney damage, and kidney damage can increase blood pressure, leading to a vicious cycle [36]. Therefore, the reduction of kidney damage is critical for hypertensive patients. Angiotensin-converting enzyme inhibitors, angiotensin receptor antagonists and calcium channel blockers are also used widely as the first-line antihypertensive agent, as they increase the glomerular filtration rate and renal blood flow by acting on the preglomerular arterioles [37]–[41]. More and more evidence show a significant role for T-type calcium channel blockers in adrenal gland that may be related to aldosterone release [42]. In addition, the new T-type CCBs, including benidipine, efonidipine and nilvadipine, have been developed and used [43]–[46]. T-type CCBs expand the efferent and afferent arterioles; reduce glomerular capillary pressure, aldosterone, and proteinuria; and play a role in kidney damage prevention and renal function protection [47]. The inhibitory effects of T-type CCBs on aldosterone synthesis and secretion [48] might play a role in the protection of renal function. Our work present new evidence supports the renal function protection of CCBs [41]. However, it is unclear which type of CCBs displays stronger renoprotective effects. Long-term treatment with ARBs or ACEIs can cause “aldosterone escape”, [10] and T-type CCBs might aid in the control of this “aldosterone escape”. These results suggest that the inhibitory effects on aldosterone synthesis and secretion might serve as a new mechanism by which T-type CCBs lower blood pressure and protect renal function. Our results provided evidence to suggest that reduced high blood pressure can improve glomerular filtration, reduce proteinuria, and protect renal function. In addition, T-type CCBs are more effective than L-type CCBs in the protection of renal function, but the effects of T-type CCBs did not significantly differ from RAS antagonists (additional studies are needed to validate this finding because small sample size, different ethnicities, and different publishing languages might lead to bias). No significant differences in SBP (p = 0.76) and DBP (p = 0.16) were noted between T-type CCBs and L-type CCBs as well as T-type CCBs and RAS antagonists; therefore, the protective effects of these agents on renal function were independent of blood pressure. The antiproteinuric effects of T-type CCBs were obvious, but the effects of GFR were not evident. In addition, the raw data of diabetic and non-diabetic phenotypes were not presented in some original studies; therefore, we were unable to examine certain subgroups. Thus, further animal experiments and clinical trials are required to elucidate the above issues. The findings reported here are important for the clinical use of antihypertensive agents to control hypertension and prevent kidney damage in hypertension patients.

However, this study had some limitations and caveats. First, the overall quality was high in our statistical tests, and the whole sample size was sufficient; however, the sample size of each subgroup was relatively small and susceptible to false positive or negative results. Second, similar to other types of research, systematic reviews are inevitably based on subjective judgments. Third, insufficient individual patient-level data could result in bias. Fourth, only studies published in English or Chinese were included, which might make the study vulnerable to the bias of language and ethnicity. Fifth, only four types of T-type CCBs, two types of L-type CCBs, and four types of RAS antagonists were assessed in this report. Moreover, the addition or withdrawal other medicines might also lead to an underestimation of the real differences in the protection of renal function between the previous reports. In addition, studies on non-dihydropyridine calcium channel blockers were not identified, so we were unable to assess their effects on renal function and aldosterone. Sixth, the CKD stage could not be distinguished in our work because most studies did not prove detailed information regarding CKD stage, which might also lead to bias. Seventh, the follow-up time of CCB or RAS treatment varied greatly (from 3 to 24 months) among different studies, potentially resulting in bias. Therefore, more head-to-head randomized controlled trials are required to investigate the association between other antihypertensive agents and the protection of renal function or aldosterone and to provide a better estimate the benefits of antihypertensive agents against kidney damage in hypertensive populations.

In conclusion, this analysis indicates that T-type CCBs, L-type CCBs, and RAS antagonists can protect renal function in the hypertensive populations. These effects can be explained in part by the antihypertensive effects of these agents. Among these agents, T-type CCBs is more effective than L-type CCBs in the protection of renal function, but did not differ from RAS antagonists. However, the proteinuria inhibitory effect of RAS antagonists was absolutely superior to T-type CCB. This systematic review and meta-analysis provided a thorough examination of the literature regarding the effects of T-type CCBs against kidney damage and provided new insights for health professionals and those engaged in the prevention of kidney damage and protection of renal function in hypertensive populations.

## Supporting Information

Figure S1 Publication bias detected by Egger’s linear regression test.
(DOC)

Figure S2 The risk of bias assessment for each included study by RevMan version 5.0.
(DOC)

Table S1 The quality assessment of evidence for each included study by GRADE profiler software version 3.2.2.
(DOC)

Table S2 The risk of bias assessment for each included study.
(DOC)

File S1 Sensitivity analysis.
(DOC)

File S2 Web resources of twenty-four studies included in the meta-analysis.
(DOC)

File S3 PDF files of twenty-four studies included in the meta-analysis.
(ZIP)

Checklist S1 PRISMA checklist.
(DOC)
