# Supplementary material for: Effects of T-Type Calcium Channel Blockers on Renal Function and Aldosterone in Patients with Hypertension: A Systematic Review and Meta-Analysis
Source: PLoS One. 2014 Oct 17;9(10):e109834. doi: 10.1371/journal.pone.0109834 (PMC4201480; doi:10.1371/journal.pone.0109834)
Supplement: Figure S1 — Publication bias detected by Egger’s linear regression test. (DOC) [file pone.0109834.s001.doc]

**Figure S1**

**Publication bias detected by Egger’s linear regression test**

A


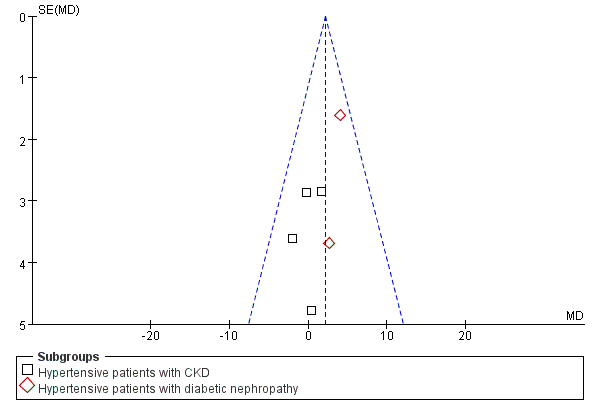


B


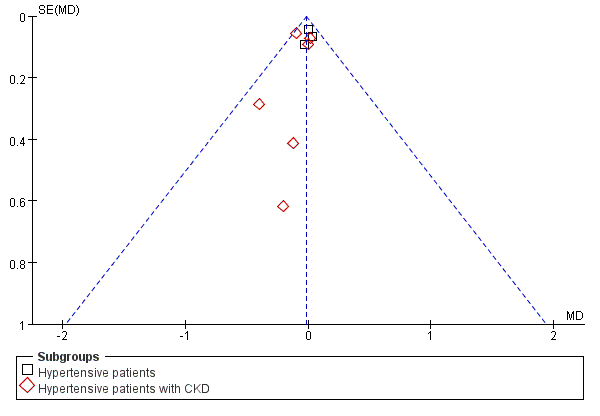


C


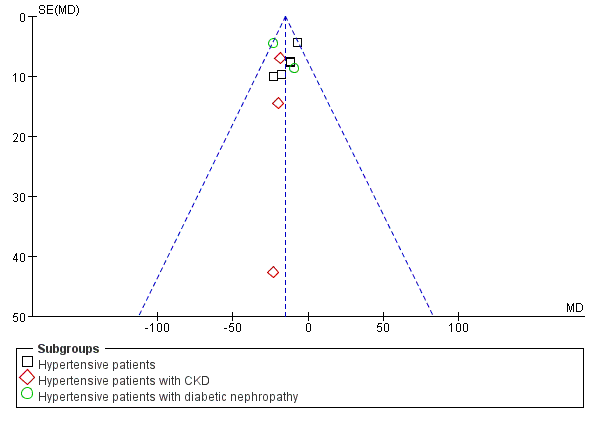


D


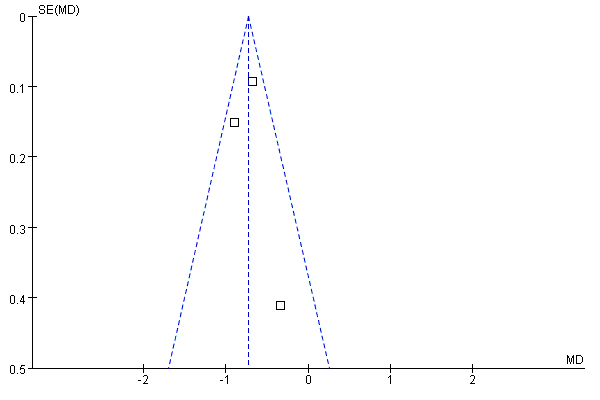


E


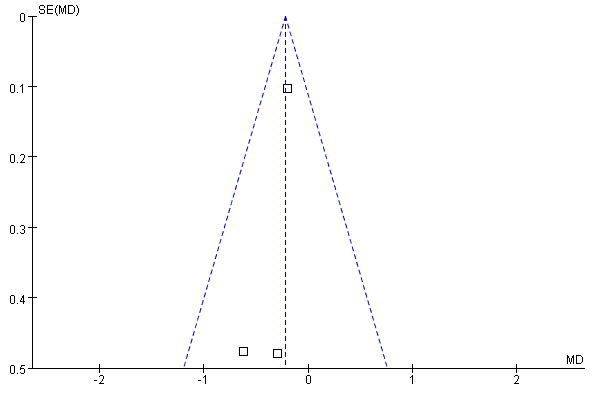


F


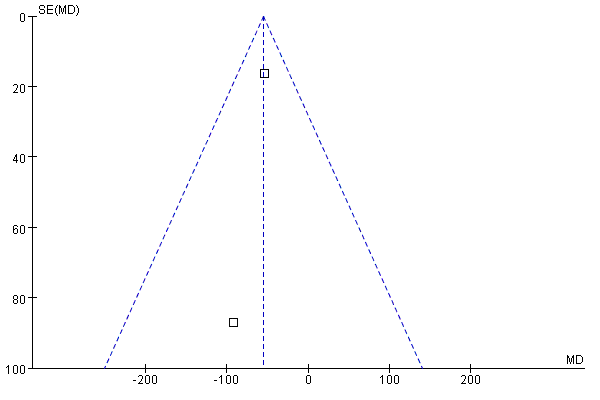


G


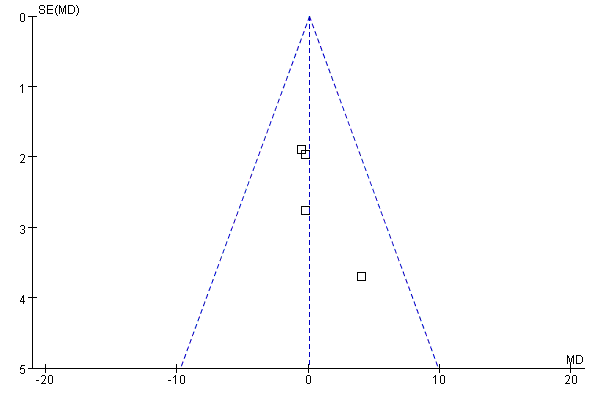


H


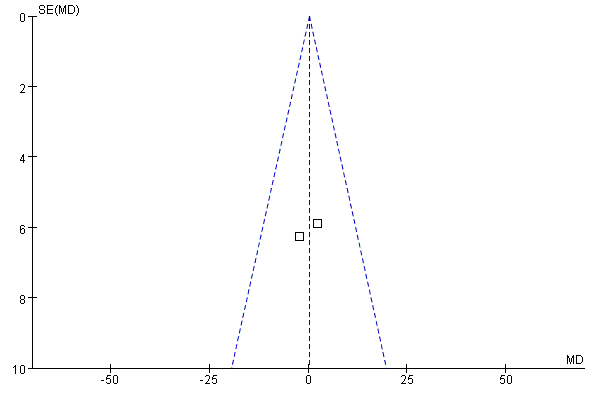


I


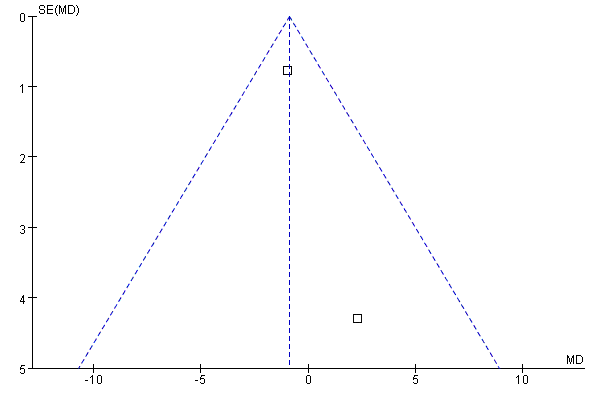


J


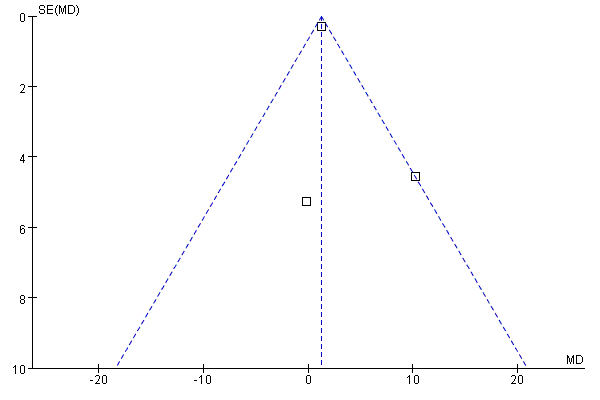


K


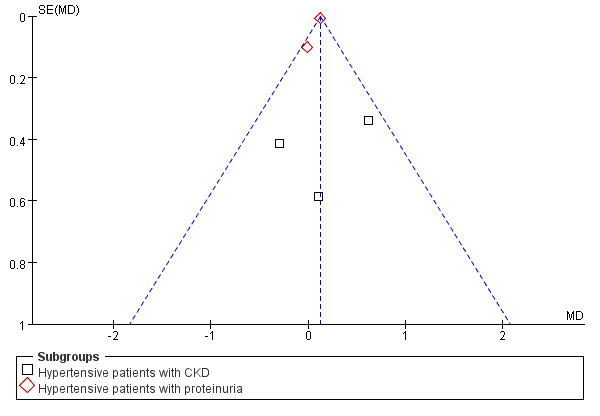


**Figure S1** Funnel plots of odds ratios for all studies in the meta-analyses. 1) T-type CCBs versus L-type CCBs: (A) Glomerular filtration rate (GFR), (B) Serum creatinine (SCr), (C) Aldosterone, (D) Proteiuria, (E) Protein to creatinine ratio, (F) Albumin to creatinine ratio; 2) T-type CCBs versus RAS antagonists: (G) Glomerular filtration rate (GFR), (H) Albuminuria, (I) Creatinine clearance rate (CCr), (J) Serum creatinine (SCr), (K) Proteinuria; No evidence of publication bias was found in any of them.
