# Supplementary figures and images for: Effects of T-Type Calcium Channel Blockers on Renal Function and Aldosterone in Patients with Hypertension: A Systematic Review and Meta-Analysis
Source: PLoS One. 2014 Oct 17;9(10):e109834. doi: 10.1371/journal.pone.0109834 (PMC4201480; doi:10.1371/journal.pone.0109834)

**Figure S2**

**The risk of bias assessment for each included study by RevMan version 5.0.**


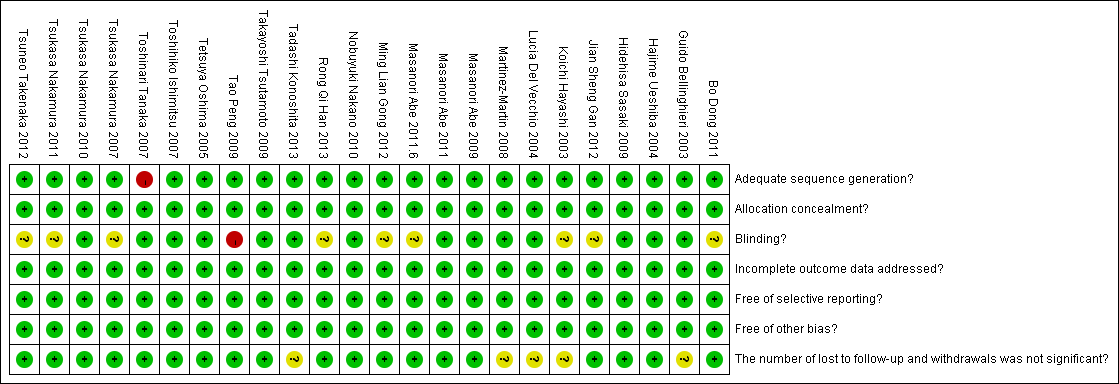


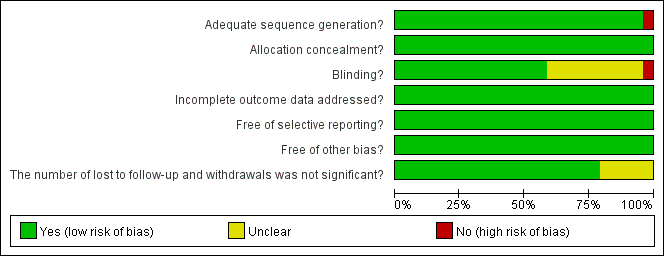

Supplement: Figure S2 — The risk of bias assessment for each included study by RevMan version 5.0. (DOC) [file pone.0109834.s002.doc]
