# Supplementary material for: Effects of T-Type Calcium Channel Blockers on Renal Function and Aldosterone in Patients with Hypertension: A Systematic Review and Meta-Analysis
Source: PLoS One. 2014 Oct 17;9(10):e109834. doi: 10.1371/journal.pone.0109834 (PMC4201480; doi:10.1371/journal.pone.0109834)
Supplement: Table S1 — The quality assessment of evidence for each included study by GRADE profiler software version 3.2.2. (DOC) [file pone.0109834.s003.doc]

**Table S1**

**The quality assessment of evidence for each included study by GRADE profiler software version 3.2.2.**

**1. T-type CCBs vs L-type CCBs**

| **T-type CCB compared to L-type CCB for Hypertensive patients with CKD for GFR** | | | | | | |
| --- | --- | --- | --- | --- | --- | --- |
| **Patient or population:** patients with Hypertensive patients with CKD for GFR **Settings:**  **Intervention:** T-type CCB **Comparison:** L-type CCB | | | | | | |
| **Outcomes** | **Illustrative comparative risks* (95% CI)** | | **Relative effect (95% CI)** | **No of Participants (studies)** | **Quality of the evidence (GRADE)** | **Comments**  **(patients of lost to follow-up)** |
|  | Assumed risk | Corresponding risk |  |  |  |  |
|  | **L-type CCB** | **T-type CCB** |  |  |  |  |
| **Masanori Abe 2009** Follow-up: 6 months |  | The mean Masanori Abe 2009 in the intervention groups was **0.24 lower** (5.84 lower to 5.36 higher) |  | 47 (1 study) | ⊕⊕⊕⊕ **high**^1^ | No report |
| **Masanori Abe 2011** Follow-up: 6 months |  | The mean Masanori Abe 2011 in the intervention groups was **1.60 higher** (3.95 lower to 7.15 higher) |  | 100 (1 study) | ⊕⊕⊕⊕ **high**^1^ | 2 lost in experimental group  2 lost in control group |
| **Tsukasa Nakamura 2011** Follow-up: 6 months |  | The mean Tsukasa Nakamura 2011 in the intervention groups was **0.40 higher** (8.94 lower to 9.74 higher) |  | 30 (1 study) | ⊕⊕⊕⊕ **high**^1^ | No report |
| **Tsuneo Takenaka 2012** Follow-up: 12 months |  | The mean Tsuneo Takenaka 2012 in the intervention groups was **2.00 lower** (9.07 lower to 5.07 higher) |  | 59 (1 study) | ⊕⊕⊕⊕ **high**^1^ | 1 lost in experimental group  2 lost in control group |
| *The basis for the **assumed risk** (e.g. the median control group risk across studies) is provided in footnotes. The **corresponding risk** (and its 95% confidence interval) is based on the assumed risk in the comparison group and the **relative effect** of the intervention (and its 95% CI).  **CI:** Confidence interval; | | | | | | |
| GRADE Working Group grades of evidence **High quality:** Further research is very unlikely to change our confidence in the estimate of effect.  **Moderate quality:** Further research is likely to have an important impact on our confidence in the estimate of effect and may change the estimate. **Low quality:** Further research is very likely to have an important impact on our confidence in the estimate of effect and is likely to change the estimate. **Very low quality:** We are very uncertain about the estimate. | | | | | | |
| ^1^ The number of lost to follow-up and withdrawals was less than 10% defined as high quality; and the rate of lost to follow-up was not significantly difference between the experimental and control groups. | | | | | | |

**Author(s):**
**Date:** 2014-03-06
**Question:** Should T-type CCB vs L-type CCB be used for Hypertensive patients with CKD for GFR?
**Settings:**
**Bibliography:**

| **Quality assessment** | | | | | | | **Summary of findings** | | | | | **Importance** |
| --- | --- | --- | --- | --- | --- | --- | --- | --- | --- | --- | --- | --- |
|  |  |  |  |  |  |  | **No of patients** | | **Effect** | | **Quality** |  |
| **No of studies** | **Design** | **Limitations** | **Inconsistency** | **Indirectness** | **Imprecision** | **Other considerations** | **T-type CCB** | **L-type CCB** | **Relative (95% CI)** | **Absolute** |  |  |
| **Masanori Abe 2009 (follow-up 6 months; Better indicated by higher values)** | | | | | | | | | | | | |
| 1 | randomised trials | no serious limitations^1^ | no serious inconsistency | no serious indirectness | no serious imprecision | none | 24 | 23 | - | MD 0.24 lower (5.84 lower to 5.36 higher) | ⊕⊕⊕⊕ HIGH | IMPORTANT |
| **Masanori Abe 2011 (follow-up 6 months; Better indicated by higher values)** | | | | | | | | | | | | |
| 1 | randomised trials | no serious limitations^1^ | no serious inconsistency | no serious indirectness | no serious imprecision | none | 50 | 50 | - | MD 1.60 higher (3.95 lower to 7.15 higher) | ⊕⊕⊕⊕ HIGH | IMPORTANT |
| **Tsukasa Nakamura 2011 (follow-up 6 months; Better indicated by higher values)** | | | | | | | | | | | | |
| 1 | randomised trials | no serious limitations^1^ | no serious inconsistency | no serious indirectness | no serious imprecision | none | 15 | 15 | - | MD 0.40 higher (8.94 lower to 9.74 higher) | ⊕⊕⊕⊕ HIGH | IMPORTANT |
| **Tsuneo Takenaka 2012 (follow-up 12 months; Better indicated by higher values)** | | | | | | | | | | | | |
| 1 | randomised trials | no serious limitations^1^ | no serious inconsistency | no serious indirectness | no serious imprecision | none | 29 | 30 | - | MD 2.00 lower (9.07 lower to 5.07 higher) | ⊕⊕⊕⊕ HIGH | IMPORTANT |

^1^ The number of lost to follow-up and withdrawals was less than 10% defined as high quality; and the rate of lost to follow-up was not significantly difference between the experimental and control groups.

| **T-type CCB compared to L-type CCB for Hypertensive patients with diabetic nephropathy for GFR** | | | | | | |
| --- | --- | --- | --- | --- | --- | --- |
| **Patient or population:** patients with Hypertensive patients with diabetic nephropathy for GFR **Settings:**  **Intervention:** T-type CCB **Comparison:** L-type CCB | | | | | | |
| **Outcomes** | **Illustrative comparative risks* (95% CI)** | | **Relative effect (95% CI)** | **No of Participants (studies)** | **Quality of the evidence (GRADE)** | **Comments**  **(patients of lost to follow-up)** |
|  | Assumed risk | Corresponding risk |  |  |  |  |
|  | **L-type CCB** | **T-type CCB** |  |  |  |  |
| **Hidehisa Sasaki 2009** Follow-up: 12 months |  | The mean Hidehisa Sasaki 2009 in the intervention groups was **4.11 higher** (0.97 to 7.25 higher) |  | 40 (1 study) | ⊕⊕⊕⊕ **high**^1^ | No report |
| **Masanori Abe 2011.6** Follow-up: 6 months |  | The mean Masanori Abe 2011.6 in the intervention groups was **2.60 higher** (4.61 lower to 9.81 higher) |  | 67 (1 study) | ⊕⊕⊕⊕ **high**^1^ | No report |
| *The basis for the **assumed risk** (e.g. the median control group risk across studies) is provided in footnotes. The **corresponding risk** (and its 95% confidence interval) is based on the assumed risk in the comparison group and the **relative effect** of the intervention (and its 95% CI).  **CI:** Confidence interval; | | | | | | |
| GRADE Working Group grades of evidence **High quality:** Further research is very unlikely to change our confidence in the estimate of effect.  **Moderate quality:** Further research is likely to have an important impact on our confidence in the estimate of effect and may change the estimate. **Low quality:** Further research is very likely to have an important impact on our confidence in the estimate of effect and is likely to change the estimate. **Very low quality:** We are very uncertain about the estimate. | | | | | | |
| ^1^ The number of lost to follow-up and withdrawals was less than 10% defined as high quality; and the rate of lost to follow-up was not significantly difference between the experimental and control groups. | | | | | | |

**Author(s):**
**Date:** 2014-03-06
**Question:** Should T-type CCB vs L-type CCB be used for Hypertensive patients with diabetic nephropathy for GFR?
**Settings:**
**Bibliography:**

| **Quality assessment** | | | | | | | **Summary of findings** | | | | | **Importance** |
| --- | --- | --- | --- | --- | --- | --- | --- | --- | --- | --- | --- | --- |
|  |  |  |  |  |  |  | **No of patients** | | **Effect** | | **Quality** |  |
| **No of studies** | **Design** | **Limitations** | **Inconsistency** | **Indirectness** | **Imprecision** | **Other considerations** | **T-type CCB** | **L-type CCB** | **Relative (95% CI)** | **Absolute** |  |  |
| **Hidehisa Sasaki 2009 (follow-up 12 months; Better indicated by higher values)** | | | | | | | | | | | | |
| 1 | randomised trials | no serious limitations^1^ | no serious inconsistency | no serious indirectness | no serious imprecision | none | 20 | 20 | - | MD 4.11 higher (0.97 to 7.25 higher) | ⊕⊕⊕⊕ HIGH | IMPORTANT |
| **Masanori Abe 2011.6 (follow-up 6 months; Better indicated by higher values)** | | | | | | | | | | | | |
| 1 | randomised trials | no serious limitations^1^ | no serious inconsistency | no serious indirectness | no serious imprecision | none | 34 | 33 | - | MD 2.60 higher (4.61 lower to 9.81 higher) | ⊕⊕⊕⊕ HIGH | IMPORTANT |

^1^ The number of lost to follow-up and withdrawals was less than 10% defined as high quality; and the rate of lost to follow-up was not significantly difference between the experimental and control groups.

| **T-type CCB compared to L-type CCB for Hypertensive patients for SCr** | | | | | | |
| --- | --- | --- | --- | --- | --- | --- |
| **Patient or population:** patients with Hypertensive patients for SCr **Settings:**  **Intervention:** T-type CCB **Comparison:** L-type CCB | | | | | | |
| **Outcomes** | **Illustrative comparative risks* (95% CI)** | | **Relative effect (95% CI)** | **No of Participants (studies)** | **Quality of the evidence (GRADE)** | **Comments**  **(patients of lost to follow-up)** |
|  | Assumed risk | Corresponding risk |  |  |  |  |
|  | **L-type CCB** | **T-type CCB** |  |  |  |  |
| **Takayoshi Tsutamoto 2009** Follow-up: 18 months |  | The mean Takayoshi Tsutamoto 2009 in the intervention groups was **0.03 higher** (0.1 lower to 0.16 higher) |  | 60 (1 study) | ⊕⊕⊕⊕ **high**^1^ | No report |
| **Tetsuya Oshima 2005** Follow-up: 3 months |  | The mean Tetsuya Oshima 2005 in the intervention groups was **0.03 lower** (0.21 lower to 0.15 higher) |  | 40 (1 study) | ⊕⊕⊕⊕ **high**^1^ | No patient was lost to follow-up and withdrew |
| **Toshinari Tanaka 2007** Follow-up: 6 months |  | The mean Toshinari Tanaka 2007 in the intervention groups was **0.00 higher** (0.08 lower to 0.08 higher) |  | 80 (1 study) | ⊕⊕⊕⊝ **moderate**^2^ | No report |
| *The basis for the **assumed risk** (e.g. the median control group risk across studies) is provided in footnotes. The **corresponding risk** (and its 95% confidence interval) is based on the assumed risk in the comparison group and the **relative effect** of the intervention (and its 95% CI).  **CI:** Confidence interval; | | | | | | |
| GRADE Working Group grades of evidence **High quality:** Further research is very unlikely to change our confidence in the estimate of effect.  **Moderate quality:** Further research is likely to have an important impact on our confidence in the estimate of effect and may change the estimate. **Low quality:** Further research is very likely to have an important impact on our confidence in the estimate of effect and is likely to change the estimate. **Very low quality:** We are very uncertain about the estimate. | | | | | | |
| ^1^ The number of lost to follow-up and withdrawals was less than 10% defined as high quality; and the rate of lost to follow-up was not significantly difference between the experimental and control groups.  ^2^ It was comparative study | | | | | | |

**Author(s):**
**Date:** 2014-03-06
**Question:** Should T-type CCB vs L-type CCB be used for Hypertensive patients for SCr?
**Settings:**
**Bibliography:**

| **Quality assessment** | | | | | | | **Summary of findings** | | | | | **Importance** |
| --- | --- | --- | --- | --- | --- | --- | --- | --- | --- | --- | --- | --- |
|  |  |  |  |  |  |  | **No of patients** | | **Effect** | | **Quality** |  |
| **No of studies** | **Design** | **Limitations** | **Inconsistency** | **Indirectness** | **Imprecision** | **Other considerations** | **T-type CCB** | **L-type CCB** | **Relative (95% CI)** | **Absolute** |  |  |
| **Takayoshi Tsutamoto 2009 (follow-up 18 months; Better indicated by lower values)** | | | | | | | | | | | | |
| 1 | randomised trials | no serious limitations^1^ | no serious inconsistency | no serious indirectness | no serious imprecision | none | 30 | 30 | - | MD 0.03 higher (0.1 lower to 0.16 higher) | ⊕⊕⊕⊕ HIGH | IMPORTANT |
| **Tetsuya Oshima 2005 (follow-up 3 months; Better indicated by lower values)** | | | | | | | | | | | | |
| 1 | randomised trials | no serious limitations^1^ | no serious inconsistency | no serious indirectness | no serious imprecision | none | 20 | 20 | - | MD 0.03 lower (0.21 lower to 0.15 higher) | ⊕⊕⊕⊕ HIGH | IMPORTANT |
| **Toshinari Tanaka 2007 (follow-up 6 months; Better indicated by lower values)** | | | | | | | | | | | | |
| 1 | randomised trials | serious^2^ | no serious inconsistency | no serious indirectness | no serious imprecision | none | 40 | 40 | - | MD 0.00 higher (0.08 lower to 0.08 higher) | ⊕⊕⊕O MODERATE | IMPORTANT |

^1^ The number of lost to follow-up and withdrawals was less than 10% defined as high quality; and the rate of lost to follow-up was not significantly difference between the experimental and control groups.
^2^ It was comparative study

| **T-type CCB compared to L-type CCB for Hypertensive patients with CKD for SCr** | | | | | | |
| --- | --- | --- | --- | --- | --- | --- |
| **Patient or population:** patients with Hypertensive patients with CKD for SCr **Settings:**  **Intervention:** T-type CCB **Comparison:** L-type CCB | | | | | | |
| **Outcomes** | **Illustrative comparative risks* (95% CI)** | | **Relative effect (95% CI)** | **No of Participants (studies)** | **Quality of the evidence (GRADE)** | **Comments**  **(patients of lost to follow-up)** |
|  | Assumed risk | Corresponding risk |  |  |  |  |
|  | **L-type CCB** | **T-type CCB** |  |  |  |  |
| **Guido Bellinghieri 2003** Follow-up: 3 months |  | The mean Guido Bellinghieri 2003 in the intervention groups was **0.40 lower** (0.96 lower to 0.16 higher) |  | 67 (1 study) | ⊕⊕⊕⊝ **moderate**^1^ | 16 lost in experimental group  15 lost in control group |
| **Masanori Abe 2009** Follow-up: 6 months |  | The mean Masanori Abe 2009 in the intervention groups was **0.12 lower** (0.93 lower to 0.69 higher) |  | 47 (1 study) | ⊕⊕⊕⊕ **high**^2^ | No report |
| **Masanori Abe 2011** Follow-up: 6 months |  | The mean Masanori Abe 2011 in the intervention groups was **0.00 higher** (0.18 lower to 0.18 higher) |  | 100 (1 study) | ⊕⊕⊕⊕ **high**^2^ | 2 lost in experimental group  2 lost in control group |
| **Nobuyuki Nakano 2010** Follow-up: 3 months |  | The mean Nobuyuki Nakano 2010 in the intervention groups was **0.20 lower** (1.41 lower to 1.01 higher) |  | 20 (1 study) | ⊕⊕⊕⊕ **high**^2^ | No patient was lost to follow-up and withdrew |
| **Toshihiko Ishimitsu 2007** Follow-up: 4 months |  | The mean Toshihiko Ishimitsu 2007 in the intervention groups was **0.02 higher** (0.12 lower to 0.16 higher) |  | 42 (1 study) | ⊕⊕⊕⊕ **high**^2^ | No report |
| **Tsukasa Nakamura 2007** Follow-up: 6 months |  | The mean Tsukasa Nakamura 2007 in the intervention groups was **0.10 lower** (0.21 lower to 0.01 higher) |  | 30 (1 study) | ⊕⊕⊕⊕ **high**^2^ | No report |
| *The basis for the **assumed risk** (e.g. the median control group risk across studies) is provided in footnotes. The **corresponding risk** (and its 95% confidence interval) is based on the assumed risk in the comparison group and the **relative effect** of the intervention (and its 95% CI).  **CI:** Confidence interval; | | | | | | |
| GRADE Working Group grades of evidence **High quality:** Further research is very unlikely to change our confidence in the estimate of effect.  **Moderate quality:** Further research is likely to have an important impact on our confidence in the estimate of effect and may change the estimate. **Low quality:** Further research is very likely to have an important impact on our confidence in the estimate of effect and is likely to change the estimate. **Very low quality:** We are very uncertain about the estimate. | | | | | | |
| ^1^ The number of lost to follow-up and withdrawals was more than 10% defined as moderate quality; and the rate of lost to follow-up was not significantly difference between the experimental and control groups. ^2^ The number of lost to follow-up and withdrawals was less than 10% defined as high quality; and the rate of lost to follow-up was not significantly difference between the experimental and control groups. | | | | | | |

**Author(s):**
**Date:** 2014-03-06
**Question:** Should T-type CCB vs L-type CCB be used for Hypertensive patients with CKD for SCr?
**Settings:**
**Bibliography:**

| **Quality assessment** | | | | | | | **Summary of findings** | | | | | **Importance** |
| --- | --- | --- | --- | --- | --- | --- | --- | --- | --- | --- | --- | --- |
|  |  |  |  |  |  |  | **No of patients** | | **Effect** | | **Quality** |  |
| **No of studies** | **Design** | **Limitations** | **Inconsistency** | **Indirectness** | **Imprecision** | **Other considerations** | **T-type CCB** | **L-type CCB** | **Relative (95% CI)** | **Absolute** |  |  |
| **Guido Bellinghieri 2003 (follow-up 3 months; Better indicated by lower values)** | | | | | | | | | | | | |
| 1 | randomised trials | serious^1^ | no serious inconsistency | no serious indirectness | no serious imprecision | none | 32 | 35 | - | MD 0.40 lower (0.96 lower to 0.16 higher) | ⊕⊕⊕O MODERATE | IMPORTANT |
| **Masanori Abe 2009 (follow-up 6 months; Better indicated by lower values)** | | | | | | | | | | | | |
| 1 | randomised trials | no serious limitations^2^ | no serious inconsistency | no serious indirectness | no serious imprecision | none | 24 | 23 | - | MD 0.12 lower (0.93 lower to 0.69 higher) | ⊕⊕⊕⊕ HIGH | IMPORTANT |
| **Masanori Abe 2011 (follow-up 6 months; Better indicated by lower values)** | | | | | | | | | | | | |
| 1 | randomised trials | no serious limitations^2^ | no serious inconsistency | no serious indirectness | no serious imprecision | none | 50 | 50 | - | MD 0.00 higher (0.18 lower to 0.18 higher) | ⊕⊕⊕⊕ HIGH | IMPORTANT |
| **Nobuyuki Nakano 2010 (follow-up 3 months; Better indicated by lower values)** | | | | | | | | | | | | |
| 1 | randomised trials | no serious limitations^2^ | no serious inconsistency | no serious indirectness | no serious imprecision | none | 20 | 0 | - | MD 0.20 lower (1.41 lower to 1.01 higher) | ⊕⊕⊕⊕ HIGH | IMPORTANT |
| **Toshihiko Ishimitsu 2007 (follow-up 4 months; Better indicated by lower values)** | | | | | | | | | | | | |
| 1 | randomised trials | no serious limitations^2^ | no serious inconsistency | no serious indirectness | no serious imprecision | none | 21 | 21 | - | MD 0.02 higher (0.12 lower to 0.16 higher) | ⊕⊕⊕⊕ HIGH | IMPORTANT |
| **Tsukasa Nakamura 2007 (follow-up 6 months; Better indicated by lower values)** | | | | | | | | | | | | |
| 1 | randomised trials | no serious limitations^2^ | no serious inconsistency | no serious indirectness | no serious imprecision | none | 15 | 15 | - | MD 0.10 lower (0.21 lower to 0.01 higher) | ⊕⊕⊕⊕ HIGH | IMPORTANT |

^1^ The number of lost to follow-up and withdrawals was more than 10% defined as moderate quality; and the rate of lost to follow-up was not significantly difference between the experimental and control groups.
^2^ The number of lost to follow-up and withdrawals was less than 10% defined as high quality; and the rate of lost to follow-up was not significantly difference between the experimental and control groups.

| **T-type CCB compared to L-type CCB for Hypertensive patients for Aldosterone** | | | | | | |
| --- | --- | --- | --- | --- | --- | --- |
| **Patient or population:** patients with Hypertensive patients for Aldosterone **Settings:**  **Intervention:** T-type CCB **Comparison:** L-type CCB | | | | | | |
| **Outcomes** | **Illustrative comparative risks* (95% CI)** | | **Relative effect (95% CI)** | **No of Participants (studies)** | **Quality of the evidence (GRADE)** | **Comments**  **(patients of lost to follow-up)** |
|  | Assumed risk | Corresponding risk |  |  |  |  |
|  | **L-type CCB** | **T-type CCB** |  |  |  |  |
| **Hajime Ueshiba 2004** Follow-up: 6 months |  | The mean Hajime Ueshiba 2004 in the intervention groups was **7.00 lower** (15.79 lower to 1.79 higher) |  | 20 (1 study) | ⊕⊕⊕⊕ **high**^1^ | No report |
| **Tadashi Konoshita 2013** Follow-up: 3 months |  | The mean Tadashi Konoshita 2013 in the intervention groups was **12.20 lower** (27.22 lower to 2.82 higher) |  | 100 (1 study) | ⊕⊕⊕⊝ **moderate**^2^ | 8 patients were lost to follow-up and withdrew |
| **Takayoshi Tsutamoto 2009** Follow-up: 18 months |  | The mean Takayoshi Tsutamoto 2009 in the intervention groups was **23.00 lower** (42.62 to 3.38 lower) |  | 60 (1 study) | ⊕⊕⊕⊕ **high**^1^ | No report |
| **Toshinari Tanaka 2007** Follow-up: 6 months |  | The mean Toshinari Tanaka 2007 in the intervention groups was **18.00 lower** (36.98 lower to 0.98 higher) |  | 80 (1 study) | ⊕⊕⊕⊝ **moderate**^3^ | No report |
| *The basis for the **assumed risk** (e.g. the median control group risk across studies) is provided in footnotes. The **corresponding risk** (and its 95% confidence interval) is based on the assumed risk in the comparison group and the **relative effect** of the intervention (and its 95% CI).  **CI:** Confidence interval; | | | | | | |
| GRADE Working Group grades of evidence **High quality:** Further research is very unlikely to change our confidence in the estimate of effect.  **Moderate quality:** Further research is likely to have an important impact on our confidence in the estimate of effect and may change the estimate. **Low quality:** Further research is very likely to have an important impact on our confidence in the estimate of effect and is likely to change the estimate. **Very low quality:** We are very uncertain about the estimate. | | | | | | |
| ^1^ The number of lost to follow-up and withdrawals was less than 10% defined as high quality; and the rate of lost to follow-up was not significantly difference between the experimental and control groups.  ^2^ The number of lost to follow-up and withdrawals was more than 10% defined as moderate quality; and the rate of lost to follow-up was not significantly difference between the experimental and control groups. ^3^ Comparative Study | | | | | | |

**Author(s):**
**Date:** 2014-03-06
**Question:** Should T-type CCB vs L-type CCB be used for Hypertensive patients for Aldosterone?
**Settings:**
**Bibliography:**

| **Quality assessment** | | | | | | | **Summary of findings** | | | | | **Importance** |
| --- | --- | --- | --- | --- | --- | --- | --- | --- | --- | --- | --- | --- |
|  |  |  |  |  |  |  | **No of patients** | | **Effect** | | **Quality** |  |
| **No of studies** | **Design** | **Limitations** | **Inconsistency** | **Indirectness** | **Imprecision** | **Other considerations** | **T-type CCB** | **L-type CCB** | **Relative (95% CI)** | **Absolute** |  |  |
| **Hajime Ueshiba 2004 (follow-up 6 months; Better indicated by lower values)** | | | | | | | | | | | | |
| 1 | randomised trials | no serious limitations^1^ | no serious inconsistency | no serious indirectness | no serious imprecision | none | 10 | 10 | - | MD 7.00 lower (15.79 lower to 1.79 higher) | ⊕⊕⊕⊕ HIGH | IMPORTANT |
| **Tadashi Konoshita 2013 (follow-up 3 months; Better indicated by lower values)** | | | | | | | | | | | | |
| 1 | randomised trials | serious^2^ | no serious inconsistency | no serious indirectness | no serious imprecision | none | 50 | 50 | - | MD 12.20 lower (27.22 lower to 2.82 higher) | ⊕⊕⊕O MODERATE | IMPORTANT |
| **Takayoshi Tsutamoto 2009 (follow-up 18 months; Better indicated by lower values)** | | | | | | | | | | | | |
| 1 | randomised trials | no serious limitations^1^ | no serious inconsistency | no serious indirectness | no serious imprecision | none | 30 | 30 | - | MD 23.00 lower (42.62 to 3.38 lower) | ⊕⊕⊕⊕ HIGH | IMPORTANT |
| **Toshinari Tanaka 2007 (follow-up 6 months; Better indicated by lower values)** | | | | | | | | | | | | |
| 1 | randomised trials | serious^3^ | no serious inconsistency | no serious indirectness | no serious imprecision | none | 40 | 40 | - | MD 18.00 lower (36.98 lower to 0.98 higher) | ⊕⊕⊕O MODERATE | IMPORTANT |

^1^ The number of lost to follow-up and withdrawals was less than 10% defined as high quality; and the rate of lost to follow-up was not significantly difference between the experimental and control groups.
^2^ The number of lost to follow-up and withdrawals was more than 10% defined as moderate quality; and the rate of lost to follow-up was not significantly difference between the experimental and control groups.
^3^ Comparative Study

| **T-type CCB compared to L-type CCB for Hypertensive patients with CKD for Aldosterone** | | | | | | |
| --- | --- | --- | --- | --- | --- | --- |
| **Patient or population:** patients with Hypertensive patients with CKD for Aldosterone **Settings:**  **Intervention:** T-type CCB **Comparison:** L-type CCB | | | | | | |
| **Outcomes** | **Illustrative comparative risks* (95% CI)** | | **Relative effect (95% CI)** | **No of Participants (studies)** | **Quality of the evidence (GRADE)** | **Comments**  **(patients of lost to follow-up)** |
|  | Assumed risk | Corresponding risk |  |  |  |  |
|  | **L-type CCB** | **T-type CCB** |  |  |  |  |
| **Masanori Abe 2011** Follow-up: 6 months |  | The mean Masanori Abe 2011 in the intervention groups was **18.50 lower** (32.36 to 4.64 lower) |  | 100 (1 study) | ⊕⊕⊕⊕ **high**^1^ | 2 lost in experimental group  2 lost in control group |
| **Nobuyuki Nakano 2010** Follow-up: 3 months |  | The mean Nobuyuki Nakano 2010 in the intervention groups was **23.00 lower** (106.64 lower to 60.64 higher) |  | 40 (1 study) | ⊕⊕⊕⊕ **high**^1^ | No patient was lost to follow-up and withdrew |
| **Toshihiko Ishimitsu 2007** Follow-up: 4 months |  | The mean Toshihiko Ishimitsu 2007 in the intervention groups was **20.00 lower** (48.43 lower to 8.43 higher) |  | 42 (1 study) | ⊕⊕⊕⊕ **high**^1^ | No report |
| *The basis for the **assumed risk** (e.g. the median control group risk across studies) is provided in footnotes. The **corresponding risk** (and its 95% confidence interval) is based on the assumed risk in the comparison group and the **relative effect** of the intervention (and its 95% CI).  **CI:** Confidence interval; | | | | | | |
| GRADE Working Group grades of evidence **High quality:** Further research is very unlikely to change our confidence in the estimate of effect.  **Moderate quality:** Further research is likely to have an important impact on our confidence in the estimate of effect and may change the estimate. **Low quality:** Further research is very likely to have an important impact on our confidence in the estimate of effect and is likely to change the estimate. **Very low quality:** We are very uncertain about the estimate. | | | | | | |
| ^1^ The number of lost to follow-up and withdrawals was less than 10% defined as high quality; and the rate of lost to follow-up was not significantly difference between the experimental and control groups. | | | | | | |

**Author(s):**
**Date:** 2014-03-06
**Question:** Should T-type CCB vs L-type CCB be used for Hypertensive patients with CKD for Aldosterone?
**Settings:**
**Bibliography:**

| **Quality assessment** | | | | | | | **Summary of findings** | | | | | **Importance** |
| --- | --- | --- | --- | --- | --- | --- | --- | --- | --- | --- | --- | --- |
|  |  |  |  |  |  |  | **No of patients** | | **Effect** | | **Quality** |  |
| **No of studies** | **Design** | **Limitations** | **Inconsistency** | **Indirectness** | **Imprecision** | **Other considerations** | **T-type CCB** | **L-type CCB** | **Relative (95% CI)** | **Absolute** |  |  |
| **Masanori Abe 2011 (follow-up 6 months; Better indicated by lower values)** | | | | | | | | | | | | |
| 1 | randomised trials | no serious limitations^1^ | no serious inconsistency | no serious indirectness | no serious imprecision | none | 50 | 50 | - | MD 18.50 lower (32.36 to 4.64 lower) | ⊕⊕⊕⊕ HIGH | IMPORTANT |
| **Nobuyuki Nakano 2010 (follow-up 3 months; Better indicated by lower values)** | | | | | | | | | | | | |
| 1 | randomised trials | no serious limitations^1^ | no serious inconsistency | no serious indirectness | no serious imprecision | none | 20 | 20 | - | MD 23.00 lower (106.64 lower to 60.64 higher) | ⊕⊕⊕⊕ HIGH | IMPORTANT |
| **Toshihiko Ishimitsu 2007 (follow-up 4 months; Better indicated by lower values)** | | | | | | | | | | | | |
| 1 | randomised trials | no serious limitations^1^ | no serious inconsistency | no serious indirectness | no serious imprecision | none | 21 | 21 | - | MD 20.00 lower (48.43 lower to 8.43 higher) | ⊕⊕⊕⊕ HIGH | IMPORTANT |

^1^ The number of lost to follow-up and withdrawals was less than 10% defined as high quality; and the rate of lost to follow-up was not significantly difference between the experimental and control groups.

| **T-type CCB compared to L-type CCB for Hypertensive patients with diabetic nephropathy for Aldosterone** | | | | | | |
| --- | --- | --- | --- | --- | --- | --- |
| **Patient or population:** patients with Hypertensive patients with diabetic nephropathy for Aldosterone **Settings:**  **Intervention:** T-type CCB **Comparison:** L-type CCB | | | | | | |
| **Outcomes** | **Illustrative comparative risks* (95% CI)** | | **Relative effect (95% CI)** | **No of Participants (studies)** | **Quality of the evidence (GRADE)** | **Comments**  **(patients of lost to follow-up)** |
|  | Assumed risk | Corresponding risk |  |  |  |  |
|  | **L-type CCB** | **T-type CCB** |  |  |  |  |
| **Hidehisa Sasaki 2009** Follow-up: 12 months |  | The mean Hidehisa Sasaki 2009 in the intervention groups was **23.06 lower** (31.9 to 14.22 lower) |  | 40 (1 study) | ⊕⊕⊕⊕ **high**^1^ | No report |
| **Masanori Abe 2011.6** Follow-up: 6 months |  | The mean Masanori Abe 2011.6 in the intervention groups was **9.47 lower** (26.64 lower to 7.7 higher) |  | 67 (1 study) | ⊕⊕⊕⊕ **high**^1^ | No report |
| *The basis for the **assumed risk** (e.g. the median control group risk across studies) is provided in footnotes. The **corresponding risk** (and its 95% confidence interval) is based on the assumed risk in the comparison group and the **relative effect** of the intervention (and its 95% CI).  **CI:** Confidence interval; | | | | | | |
| GRADE Working Group grades of evidence **High quality:** Further research is very unlikely to change our confidence in the estimate of effect.  **Moderate quality:** Further research is likely to have an important impact on our confidence in the estimate of effect and may change the estimate. **Low quality:** Further research is very likely to have an important impact on our confidence in the estimate of effect and is likely to change the estimate. **Very low quality:** We are very uncertain about the estimate. | | | | | | |
| ^1^ The number of lost to follow-up and withdrawals was less than 10% defined as high quality; and the rate of lost to follow-up was not significantly difference between the experimental and control groups. | | | | | | |

**Author(s):**
**Date:** 2014-03-06
**Question:** Should T-type CCB vs L-type CCB be used for Hypertensive patients with diabetic nephropathy for Aldosterone?
**Settings:**
**Bibliography:**

| **Quality assessment** | | | | | | | **Summary of findings** | | | | | **Importance** |
| --- | --- | --- | --- | --- | --- | --- | --- | --- | --- | --- | --- | --- |
|  |  |  |  |  |  |  | **No of patients** | | **Effect** | | **Quality** |  |
| **No of studies** | **Design** | **Limitations** | **Inconsistency** | **Indirectness** | **Imprecision** | **Other considerations** | **T-type CCB** | **L-type CCB** | **Relative (95% CI)** | **Absolute** |  |  |
| **Hidehisa Sasaki 2009 (follow-up 12 months; Better indicated by lower values)** | | | | | | | | | | | | |
| 1 | randomised trials | no serious limitations^1^ | no serious inconsistency | no serious indirectness | no serious imprecision | none | 20 | 20 | - | MD 23.06 lower (31.9 to 14.22 lower) | ⊕⊕⊕⊕ HIGH | IMPORTANT |
| **Masanori Abe 2011.6 (follow-up 6 months; Better indicated by lower values)** | | | | | | | | | | | | |
| 1 | randomised trials | no serious limitations^1^ | no serious inconsistency | no serious indirectness | no serious imprecision | none | 34 | 33 | - | MD 9.47 lower (26.64 lower to 7.7 higher) | ⊕⊕⊕⊕ HIGH | IMPORTANT |

^1^ The number of lost to follow-up and withdrawals was less than 10% defined as high quality; and the rate of lost to follow-up was not significantly difference between the experimental and control groups.

| **T-type CCB compared to L-type CCB for Hypertensive patients with CKD of proteiuria** | | | | | | |
| --- | --- | --- | --- | --- | --- | --- |
| **Patient or population:** patients with Hypertensive patients with CKD of proteiuria **Settings:**  **Intervention:** T-type CCB **Comparison:** L-type CCB | | | | | | |
| **Outcomes** | **Illustrative comparative risks* (95% CI)** | | **Relative effect (95% CI)** | **No of Participants (studies)** | **Quality of the evidence (GRADE)** | **Comments**  **(patients of lost to follow-up)** |
|  | Assumed risk | Corresponding risk |  |  |  |  |
|  | **L-type CCB** | **T-type CCB** |  |  |  |  |
| **Guido Bellinghieri 2003** Follow-up: 3 months |  | The mean Guido Bellinghieri 2003 in the intervention groups was **0.34 lower** (1.14 lower to 0.46 higher) |  | 67 (1 study) | ⊕⊕⊕⊝ **moderate**^1^ | 16 lost in experimental group  15 lost in control group |
| **Tsukasa Nakamura 2007** Follow-up: 6 months |  | The mean Tsukasa Nakamura 2007 in the intervention groups was **0.90 lower** (1.2 to 0.6 lower) |  | 30 (1 study) | ⊕⊕⊕⊕ **high**^2^ | No report |
| **Tsukasa Nakamura 2010** Follow-up: 12 months |  | The mean Tsukasa Nakamura 2010 in the intervention groups was **0.68 lower** (0.86 to 0.5 lower) |  | 40 (1 study) | ⊕⊕⊕⊕ **high**^2^ | No report |
| *The basis for the **assumed risk** (e.g. the median control group risk across studies) is provided in footnotes. The **corresponding risk** (and its 95% confidence interval) is based on the assumed risk in the comparison group and the **relative effect** of the intervention (and its 95% CI).  **CI:** Confidence interval; | | | | | | |
| GRADE Working Group grades of evidence **High quality:** Further research is very unlikely to change our confidence in the estimate of effect.  **Moderate quality:** Further research is likely to have an important impact on our confidence in the estimate of effect and may change the estimate. **Low quality:** Further research is very likely to have an important impact on our confidence in the estimate of effect and is likely to change the estimate. **Very low quality:** We are very uncertain about the estimate. | | | | | | |
| ^1^ The number of lost to follow-up and withdrawals was more than 10% defined as moderate quality; and the rate of lost to follow-up was not significantly difference between the experimental and control groups. ^2^ The number of lost to follow-up and withdrawals was less than 10% defined as high quality; and the rate of lost to follow-up was not significantly difference between the experimental and control groups. | | | | | | |

**Author(s):**
**Date:** 2014-03-06
**Question:** Should T-type CCB vs L-type CCB be used for Hypertensive patients with CKD of proteiuria?
**Settings:**
**Bibliography:**

| **Quality assessment** | | | | | | | **Summary of findings** | | | | | **Importance** |
| --- | --- | --- | --- | --- | --- | --- | --- | --- | --- | --- | --- | --- |
|  |  |  |  |  |  |  | **No of patients** | | **Effect** | | **Quality** |  |
| **No of studies** | **Design** | **Limitations** | **Inconsistency** | **Indirectness** | **Imprecision** | **Other considerations** | **T-type CCB** | **L-type CCB** | **Relative (95% CI)** | **Absolute** |  |  |
| **Guido Bellinghieri 2003 (follow-up 3 months; Better indicated by lower values)** | | | | | | | | | | | | |
| 1 | randomised trials | serious^1^ | no serious inconsistency | no serious indirectness | no serious imprecision | none | 32 | 35 | - | MD 0.34 lower (1.14 lower to 0.46 higher) | ⊕⊕⊕O MODERATE | IMPORTANT |
| **Tsukasa Nakamura 2007 (follow-up 6 months; Better indicated by lower values)** | | | | | | | | | | | | |
| 1 | randomised trials | no serious limitations^2^ | no serious inconsistency | no serious indirectness | no serious imprecision | none | 15 | 15 | - | MD 0.90 lower (1.2 to 0.6 lower) | ⊕⊕⊕⊕ HIGH | IMPORTANT |
| **Tsukasa Nakamura 2010 (follow-up 12 months; Better indicated by lower values)** | | | | | | | | | | | | |
| 1 | randomised trials | no serious limitations^2^ | no serious inconsistency | no serious indirectness | no serious imprecision | none | 20 | 20 | - | MD 0.68 lower (0.86 to 0.5 lower) | ⊕⊕⊕⊕ HIGH | IMPORTANT |

^1^ The number of lost to follow-up and withdrawals was more than 10% defined as moderate quality; and the rate of lost to follow-up was not significantly difference between the experimental and control groups.
^2^ The number of lost to follow-up and withdrawals was less than 10% defined as high quality; and the rate of lost to follow-up was not significantly difference between the experimental and control groups.

| **T-type CCB compared to L-type CCB for Hypertensive patients with CKD of urinary protein to creatinine ratio** | | | | | | |
| --- | --- | --- | --- | --- | --- | --- |
| **Patient or population:** patients with Hypertensive patients with CKD of urinary protein to creatinine ratio **Settings:**  **Intervention:** T-type CCB **Comparison:** L-type CCB | | | | | | |
| **Outcomes** | **Illustrative comparative risks* (95% CI)** | | **Relative effect (95% CI)** | **No of Participants (studies)** | **Quality of the evidence (GRADE)** | **Comments**  **(patients of lost to follow-up)** |
|  | Assumed risk | Corresponding risk |  |  |  |  |
|  | **L-type CCB** | **T-type CCB** |  |  |  |  |
| **Masanori Abe 2009** Follow-up: 6 months |  | The mean Masanori Abe 2009 in the intervention groups was **0.62 lower** (1.55 lower to 0.31 higher) |  | 47 (1 study) | ⊕⊕⊕⊕ **high**^1^ | No report |
| **Toshihiko Ishimitsu 2007** Follow-up: 4 months |  | The mean Toshihiko Ishimitsu 2007 in the intervention groups was **0.30 lower** (1.24 lower to 0.64 higher) |  | 42 (1 study) | ⊕⊕⊕⊕ **high**^1^ | No report |
| **Tsuneo Takenaka 2012** Follow-up: 12 months |  | The mean Tsuneo Takenaka 2012 in the intervention groups was **0.20 lower** (0.4 lower to 0 higher) |  | 59 (1 study) | ⊕⊕⊕⊕ **high**^1^ | 1 lost in experimental group  2 lost in control group |
| *The basis for the **assumed risk** (e.g. the median control group risk across studies) is provided in footnotes. The **corresponding risk** (and its 95% confidence interval) is based on the assumed risk in the comparison group and the **relative effect** of the intervention (and its 95% CI).  **CI:** Confidence interval; | | | | | | |
| GRADE Working Group grades of evidence **High quality:** Further research is very unlikely to change our confidence in the estimate of effect.  **Moderate quality:** Further research is likely to have an important impact on our confidence in the estimate of effect and may change the estimate. **Low quality:** Further research is very likely to have an important impact on our confidence in the estimate of effect and is likely to change the estimate. **Very low quality:** We are very uncertain about the estimate. | | | | | | |
| ^1^ The number of lost to follow-up and withdrawals was less than 10% defined as high quality; and the rate of lost to follow-up was not significantly difference between the experimental and control groups. | | | | | | |

**Author(s):**
**Date:** 2014-03-06
**Question:** Should T-type CCB vs L-type CCB be used for Hypertensive patients with CKD of urinary protein to creatinine ratio?
**Settings:**
**Bibliography:**

| **Quality assessment** | | | | | | | **Summary of findings** | | | | | **Importance** |
| --- | --- | --- | --- | --- | --- | --- | --- | --- | --- | --- | --- | --- |
|  |  |  |  |  |  |  | **No of patients** | | **Effect** | | **Quality** |  |
| **No of studies** | **Design** | **Limitations** | **Inconsistency** | **Indirectness** | **Imprecision** | **Other considerations** | **T-type CCB** | **L-type CCB** | **Relative (95% CI)** | **Absolute** |  |  |
| **Masanori Abe 2009 (follow-up 6 months; Better indicated by lower values)** | | | | | | | | | | | | |
| 1 | randomised trials | no serious limitations^1^ | no serious inconsistency | no serious indirectness | no serious imprecision | none | 24 | 23 | - | MD 0.62 lower (1.55 lower to 0.31 higher) | ⊕⊕⊕⊕ HIGH | IMPORTANT |
| **Toshihiko Ishimitsu 2007 (follow-up 4 months; Better indicated by lower values)** | | | | | | | | | | | | |
| 1 | randomised trials | no serious limitations^1^ | no serious inconsistency | no serious indirectness | no serious imprecision | none | 21 | 21 | - | MD 0.30 lower (1.24 lower to 0.64 higher) | ⊕⊕⊕⊕ HIGH | IMPORTANT |
| **Tsuneo Takenaka 2012 (follow-up 12 months; Better indicated by lower values)** | | | | | | | | | | | | |
| 1 | randomised trials | no serious limitations^1^ | no serious inconsistency | no serious indirectness | no serious imprecision | none | 29 | 30 | - | MD 0.20 lower (0.4 lower to 0 higher) | ⊕⊕⊕⊕ HIGH | IMPORTANT |

^1^ The number of lost to follow-up and withdrawals was less than 10% defined as high quality; and the rate of lost to follow-up was not significantly difference between the experimental and control groups.

| **T-type CCB compared to L-type CCB for Hypertensive patients with diabetic nephropathy of urinary albumin to creatinine ratio** | | | | | | |
| --- | --- | --- | --- | --- | --- | --- |
| **Patient or population:** patients with Hypertensive patients with diabetic nephropathy of urinary albumin to creatinine ratio **Settings:**  **Intervention:** T-type CCB **Comparison:** L-type CCB | | | | | | |
| **Outcomes** | **Illustrative comparative risks* (95% CI)** | | **Relative effect (95% CI)** | **No of Participants (studies)** | **Quality of the evidence (GRADE)** | **Comments**  **(patients of lost to follow-up)** |
|  | Assumed risk | Corresponding risk |  |  |  |  |
|  | **L-type CCB** | **T-type CCB** |  |  |  |  |
| **Martinez-Martin 2008** Follow-up: 24 months |  | The mean Martinez-Martin 2008 in the intervention groups was **54.10 lower** (85.94 to 22.26 lower) |  | 74 (1 study) | ⊕⊕⊕⊝ **moderate**^1^ | 7 lost in experimental group  10 lost in control group |
| **Masanori Abe 2011.6** Follow-up: 6 months |  | The mean Masanori Abe 2011.6 in the intervention groups was **92.00 lower** (262.19 lower to 78.19 higher) |  | 67 (1 study) | ⊕⊕⊕⊕ **high**^2^ | No report |
| *The basis for the **assumed risk** (e.g. the median control group risk across studies) is provided in footnotes. The **corresponding risk** (and its 95% confidence interval) is based on the assumed risk in the comparison group and the **relative effect** of the intervention (and its 95% CI).  **CI:** Confidence interval; | | | | | | |
| GRADE Working Group grades of evidence **High quality:** Further research is very unlikely to change our confidence in the estimate of effect.  **Moderate quality:** Further research is likely to have an important impact on our confidence in the estimate of effect and may change the estimate. **Low quality:** Further research is very likely to have an important impact on our confidence in the estimate of effect and is likely to change the estimate. **Very low quality:** We are very uncertain about the estimate. | | | | | | |
| ^1^ The number of lost to follow-up and withdrawals was more than 10% defined as moderate quality; and the rate of lost to follow-up was not significantly difference between the experimental and control groups. ^2^ The number of lost to follow-up and withdrawals was less than 10% defined as high quality; and the rate of lost to follow-up was not significantly difference between the experimental and control groups. | | | | | | |

**Author(s):**
**Date:** 2014-03-06
**Question:** Should T-type CCB vs L-type CCB be used for Hypertensive patients with diabetic nephropathy of urinary albumin to creatinine ratio?
**Settings:**
**Bibliography:**

| **Quality assessment** | | | | | | | **Summary of findings** | | | | | **Importance** |
| --- | --- | --- | --- | --- | --- | --- | --- | --- | --- | --- | --- | --- |
|  |  |  |  |  |  |  | **No of patients** | | **Effect** | | **Quality** |  |
| **No of studies** | **Design** | **Limitations** | **Inconsistency** | **Indirectness** | **Imprecision** | **Other considerations** | **T-type CCB** | **L-type CCB** | **Relative (95% CI)** | **Absolute** |  |  |
| **Martinez-Martin 2008 (follow-up 24 months; Better indicated by lower values)** | | | | | | | | | | | | |
| 1 | randomised trials | serious^1^ | no serious inconsistency | no serious indirectness | no serious imprecision | none | 54 | 20 | - | MD 54.10 lower (85.94 to 22.26 lower) | ⊕⊕⊕O MODERATE | IMPORTANT |
| **Masanori Abe 2011.6 (follow-up 6 months; Better indicated by lower values)** | | | | | | | | | | | | |
| 1 | randomised trials | no serious limitations^2^ | no serious inconsistency | no serious indirectness | no serious imprecision | none | 34 | 33 | - | MD 92.00 lower (262.19 lower to 78.19 higher) | ⊕⊕⊕⊕ HIGH | IMPORTANT |

^1^ The number of lost to follow-up and withdrawals was more than 10% defined as moderate quality; and the rate of lost to follow-up was not significantly difference between the experimental and control groups.
^2^ The number of lost to follow-up and withdrawals was less than 10% defined as high quality; and the rate of lost to follow-up was not significantly difference between the experimental and control groups.

**2. T-type CCBs vs RAS antagonists**

| **T-type CCB compared to RAS for Hypertensive patients with proteinuria for GFR** | | | | | | |
| --- | --- | --- | --- | --- | --- | --- |
| **Patient or population:** patients with Hypertensive patients with proteinuria for GFR **Settings:**  **Intervention:** T-type CCB **Comparison:** RAS | | | | | | |
| **Outcomes** | **Illustrative comparative risks* (95% CI)** | | **Relative effect (95% CI)** | **No of Participants (studies)** | **Quality of the evidence (GRADE)** | **Comments**  **(patients of lost to follow-up)** |
|  | Assumed risk | Corresponding risk |  |  |  |  |
|  | **RAS** | **T-type CCB** |  |  |  |  |
| **Bo Dong 2011** Follow-up: 12 months |  | The mean Bo Dong 2011 in the intervention groups was **0.20 lower** (5.59 lower to 5.19 higher) |  | 60 (1 study) | ⊕⊕⊕⊕ **high**^1^ | No report |
| **Rong Qi Han 2013** Follow-up: 3 months |  | The mean Rong Qi Han 2013 in the intervention groups was **4.00 higher** (3.24 lower to 11.24 higher) |  | 80 (1 study) | ⊕⊕⊕⊕ **high**^1^ | No report |
| **Tao Peng 2009** Follow-up: 12 months |  | The mean Tao Peng 2009 in the intervention groups was **0.50 lower** (4.2 lower to 3.2 higher) |  | 116 (1 study) | ⊕⊕⊕⊕ **high**^1^ | No report |
| **Tao Peng 2009** Follow-up: 12 months |  | The mean Tao Peng 2009 in the intervention groups was **0.20 lower** (4.05 lower to 3.65 higher) |  | 120 (1 study) | ⊕⊕⊕⊕ **high**^1^ | No report |
| *The basis for the **assumed risk** (e.g. the median control group risk across studies) is provided in footnotes. The **corresponding risk** (and its 95% confidence interval) is based on the assumed risk in the comparison group and the **relative effect** of the intervention (and its 95% CI).  **CI:** Confidence interval; | | | | | | |
| GRADE Working Group grades of evidence **High quality:** Further research is very unlikely to change our confidence in the estimate of effect.  **Moderate quality:** Further research is likely to have an important impact on our confidence in the estimate of effect and may change the estimate. **Low quality:** Further research is very likely to have an important impact on our confidence in the estimate of effect and is likely to change the estimate. **Very low quality:** We are very uncertain about the estimate. | | | | | | |
| ^1^ The number of lost to follow-up and withdrawals was less than 10% defined as high quality; and the rate of lost to follow-up was not significantly difference between the experimental and control groups. | | | | | | |

**Author(s):**
**Date:** 2014-03-06
**Question:** Should T-type CCB vs RAS be used for Hypertensive patients with proteinuria for GFR?
**Settings:**
**Bibliography:**

| **Quality assessment** | | | | | | | **Summary of findings** | | | | | **Importance** |
| --- | --- | --- | --- | --- | --- | --- | --- | --- | --- | --- | --- | --- |
|  |  |  |  |  |  |  | **No of patients** | | **Effect** | | **Quality** |  |
| **No of studies** | **Design** | **Limitations** | **Inconsistency** | **Indirectness** | **Imprecision** | **Other considerations** | **T-type CCB** | **RAS** | **Relative (95% CI)** | **Absolute** |  |  |
| **Bo Dong 2011 (follow-up 12 months; Better indicated by higher values)** | | | | | | | | | | | | |
| 1 | randomised trials | no serious limitations^1^ | no serious inconsistency | no serious indirectness | no serious imprecision | none | 30 | 30 | - | MD 0.20 lower (5.59 lower to 5.19 higher) | ⊕⊕⊕⊕ HIGH | IMPORTANT |
| **Rong Qi Han 2013 (follow-up 3 months; Better indicated by higher values)** | | | | | | | | | | | | |
| 1 | randomised trials | no serious limitations^1^ | no serious inconsistency | no serious indirectness | no serious imprecision | none | 40 | 40 | - | MD 4.00 higher (3.24 lower to 11.24 higher) | ⊕⊕⊕⊕ HIGH | IMPORTANT |
| **Tao Peng 2009 (follow-up 12 months; Better indicated by higher values)** | | | | | | | | | | | | |
| 1 | randomised trials | no serious limitations^1^ | no serious inconsistency | no serious indirectness | no serious imprecision | none | 59 | 57 | - | MD 0.50 lower (4.2 lower to 3.2 higher) | ⊕⊕⊕⊕ HIGH | IMPORTANT |
| **Tao Peng 2009 (follow-up 12 months; Better indicated by higher values)** | | | | | | | | | | | | |
| 1 | randomised trials | no serious limitations^1^ | no serious inconsistency | no serious indirectness | no serious imprecision | none | 59 | 61 | - | MD 0.20 lower (4.05 lower to 3.65 higher) | ⊕⊕⊕⊕ HIGH | IMPORTANT |

^1^ The number of lost to follow-up and withdrawals was less than 10% defined as high quality; and the rate of lost to follow-up was not significantly difference between the experimental and control groups.

| **T-type CCB compared to RAS for Hypertensive patients with proteinuria for Albuminuria** | | | | | | |
| --- | --- | --- | --- | --- | --- | --- |
| **Patient or population:** patients with Hypertensive patients with proteinuria for Albuminuria **Settings:**  **Intervention:** T-type CCB **Comparison:** RAS | | | | | | |
| **Outcomes** | **Illustrative comparative risks* (95% CI)** | | **Relative effect (95% CI)** | **No of Participants (studies)** | **Quality of the evidence (GRADE)** | **Comments**  **(patients of lost to follow-up)** |
|  | Assumed risk | Corresponding risk |  |  |  |  |
|  | **RAS** | **T-type CCB** |  |  |  |  |
| **Ming Lian Gong 2012** Follow-up: 6 months |  | The mean Ming Lian Gong 2012 in the intervention groups was **2.30 higher** (9.24 lower to 13.84 higher) |  | 90 (1 study) | ⊕⊕⊕⊕ **high**^1^ | No report |
| **Rong Qi Han 2013** Follow-up: 3 months |  | The mean Rong Qi Han 2013 in the intervention groups was **2.30 lower** (14.55 lower to 9.95 higher) |  | 80 (1 study) | ⊕⊕⊕⊕ **high**^1^ | No report |
| *The basis for the **assumed risk** (e.g. the median control group risk across studies) is provided in footnotes. The **corresponding risk** (and its 95% confidence interval) is based on the assumed risk in the comparison group and the **relative effect** of the intervention (and its 95% CI).  **CI:** Confidence interval; | | | | | | |
| GRADE Working Group grades of evidence **High quality:** Further research is very unlikely to change our confidence in the estimate of effect.  **Moderate quality:** Further research is likely to have an important impact on our confidence in the estimate of effect and may change the estimate. **Low quality:** Further research is very likely to have an important impact on our confidence in the estimate of effect and is likely to change the estimate. **Very low quality:** We are very uncertain about the estimate. | | | | | | |
| ^1^ The number of lost to follow-up and withdrawals was less than 10% defined as high quality; and the rate of lost to follow-up was not significantly difference between the experimental and control groups. | | | | | | |

**Author(s):**
**Date:** 2014-03-06
**Question:** Should T-type CCB vs RAS be used for Hypertensive patients with proteinuria for Albuminuria?
**Settings:**
**Bibliography:**

| **Quality assessment** | | | | | | | **Summary of findings** | | | | | **Importance** |
| --- | --- | --- | --- | --- | --- | --- | --- | --- | --- | --- | --- | --- |
|  |  |  |  |  |  |  | **No of patients** | | **Effect** | | **Quality** |  |
| **No of studies** | **Design** | **Limitations** | **Inconsistency** | **Indirectness** | **Imprecision** | **Other considerations** | **T-type CCB** | **RAS** | **Relative (95% CI)** | **Absolute** |  |  |
| **Ming Lian Gong 2012 (follow-up 6 months; Better indicated by lower values)** | | | | | | | | | | | | |
| 1 | randomised trials | no serious limitations^1^ | no serious inconsistency | no serious indirectness | no serious imprecision | none | 45 | 45 | - | MD 2.30 higher (9.24 lower to 13.84 higher) | ⊕⊕⊕⊕ HIGH | IMPORTANT |
| **Rong Qi Han 2013 (follow-up 3 months; Better indicated by lower values)** | | | | | | | | | | | | |
| 1 | randomised trials | no serious limitations^1^ | no serious inconsistency | no serious indirectness | no serious imprecision | none | 40 | 40 | - | MD 2.30 lower (14.55 lower to 9.95 higher) | ⊕⊕⊕⊕ HIGH | IMPORTANT |

^1^ The number of lost to follow-up and withdrawals was less than 10% defined as high quality; and the rate of lost to follow-up was not significantly difference between the experimental and control groups.

| **T-type CCB compared to RAS for Hypertensive patients with proteinuria for CCr** | | | | | | |
| --- | --- | --- | --- | --- | --- | --- |
| **Patient or population:** patients with Hypertensive patients with proteinuria for CCr **Settings:**  **Intervention:** T-type CCB **Comparison:** RAS | | | | | | |
| **Outcomes** | **Illustrative comparative risks* (95% CI)** | | **Relative effect (95% CI)** | **No of Participants (studies)** | **Quality of the evidence (GRADE)** | **Comments**  **(patients of lost to follow-up)** |
|  | Assumed risk | Corresponding risk |  |  |  |  |
|  | **RAS** | **T-type CCB** |  |  |  |  |
| **Jian Sheng Gan 2012** Follow-up: 6 months |  | The mean Jian Sheng Gan 2012 in the intervention groups was **1.00 lower** (2.51 lower to 0.51 higher) |  | 286 (1 study) | ⊕⊕⊕⊕ **high**^1^ | No report |
| **Rong Qi Han 2013** Follow-up: 3 months |  | The mean Rong Qi Han 2013 in the intervention groups was **2.30 higher** (6.09 lower to 10.69 higher) |  | 80 (1 study) | ⊕⊕⊕⊕ **high**^1^ | No report |
| *The basis for the **assumed risk** (e.g. the median control group risk across studies) is provided in footnotes. The **corresponding risk** (and its 95% confidence interval) is based on the assumed risk in the comparison group and the **relative effect** of the intervention (and its 95% CI).  **CI:** Confidence interval; | | | | | | |
| GRADE Working Group grades of evidence **High quality:** Further research is very unlikely to change our confidence in the estimate of effect.  **Moderate quality:** Further research is likely to have an important impact on our confidence in the estimate of effect and may change the estimate. **Low quality:** Further research is very likely to have an important impact on our confidence in the estimate of effect and is likely to change the estimate. **Very low quality:** We are very uncertain about the estimate. | | | | | | |
| ^1^ The number of lost to follow-up and withdrawals was less than 10% defined as high quality; and the rate of lost to follow-up was not significantly difference between the experimental and control groups. | | | | | | |

**Author(s):**
**Date:** 2014-03-06
**Question:** Should T-type CCB vs RAS be used for Hypertensive patients with proteinuria for CCr?
**Settings:**
**Bibliography:**

| **Quality assessment** | | | | | | | **Summary of findings** | | | | | **Importance** |
| --- | --- | --- | --- | --- | --- | --- | --- | --- | --- | --- | --- | --- |
|  |  |  |  |  |  |  | **No of patients** | | **Effect** | | **Quality** |  |
| **No of studies** | **Design** | **Limitations** | **Inconsistency** | **Indirectness** | **Imprecision** | **Other considerations** | **T-type CCB** | **RAS** | **Relative (95% CI)** | **Absolute** |  |  |
| **Jian Sheng Gan 2012 (follow-up 6 months; Better indicated by higher values)** | | | | | | | | | | | | |
| 1 | randomised trials | no serious limitations^1^ | no serious inconsistency | no serious indirectness | no serious imprecision | none | 143 | 143 | - | MD 1.00 lower (2.51 lower to 0.51 higher) | ⊕⊕⊕⊕ HIGH | IMPORTANT |
| **Rong Qi Han 2013 (follow-up 3 months; Better indicated by higher values)** | | | | | | | | | | | | |
| 1 | randomised trials | no serious limitations^1^ | no serious inconsistency | no serious indirectness | no serious imprecision | none | 40 | 40 | - | MD 2.30 higher (6.09 lower to 10.69 higher) | ⊕⊕⊕⊕ HIGH | IMPORTANT |

^1^ The number of lost to follow-up and withdrawals was less than 10% defined as high quality; and the rate of lost to follow-up was not significantly difference between the experimental and control groups.

| **T-type CCB compared to RAS for Hypertensive patients with proteinuria for SCr** | | | | | | |
| --- | --- | --- | --- | --- | --- | --- |
| **Patient or population:** patients with Hypertensive patients with proteinuria for SCr **Settings:**  **Intervention:** T-type CCB **Comparison:** RAS | | | | | | |
| **Outcomes** | **Illustrative comparative risks* (95% CI)** | | **Relative effect (95% CI)** | **No of Participants (studies)** | **Quality of the evidence (GRADE)** | **Comments**  **(patients of lost to follow-up)** |
|  | Assumed risk | Corresponding risk |  |  |  |  |
|  | **RAS** | **T-type CCB** |  |  |  |  |
| **Bo Dong 2011** Follow-up: 12 months |  | The mean Bo Dong 2011 in the intervention groups was **0.20 lower** (10.5 lower to 10.1 higher) |  | 60 (1 study) | ⊕⊕⊕⊕ **high**^1^ | No report |
| **Jian Sheng Gan 2012** Follow-up: 6 months |  | The mean Jian Sheng Gan 2012 in the intervention groups was **1.20 higher** (0.63 to 1.77 higher) |  | 286 (1 study) | ⊕⊕⊕⊕ **high**^1^ | No report |
| **Ming Lian Gong 2012** Follow-up: 6 months |  | The mean Ming Lian Gong 2012 in the intervention groups was **10.20 higher** (1.31 to 19.09 higher) |  | 90 (1 study) | ⊕⊕⊕⊕ **high**^1^ | No report |
| *The basis for the **assumed risk** (e.g. the median control group risk across studies) is provided in footnotes. The **corresponding risk** (and its 95% confidence interval) is based on the assumed risk in the comparison group and the **relative effect** of the intervention (and its 95% CI).  **CI:** Confidence interval; | | | | | | |
| GRADE Working Group grades of evidence **High quality:** Further research is very unlikely to change our confidence in the estimate of effect.  **Moderate quality:** Further research is likely to have an important impact on our confidence in the estimate of effect and may change the estimate. **Low quality:** Further research is very likely to have an important impact on our confidence in the estimate of effect and is likely to change the estimate. **Very low quality:** We are very uncertain about the estimate. | | | | | | |
| ^1^ The number of lost to follow-up and withdrawals was less than 10% defined as high quality; and the rate of lost to follow-up was not significantly difference between the experimental and control groups. | | | | | | |

**Author(s):**
**Date:** 2014-03-06
**Question:** Should T-type CCB vs RAS be used for Hypertensive patients with proteinuria for SCr?
**Settings:**
**Bibliography:**

| **Quality assessment** | | | | | | | **Summary of findings** | | | | | **Importance** |
| --- | --- | --- | --- | --- | --- | --- | --- | --- | --- | --- | --- | --- |
|  |  |  |  |  |  |  | **No of patients** | | **Effect** | | **Quality** |  |
| **No of studies** | **Design** | **Limitations** | **Inconsistency** | **Indirectness** | **Imprecision** | **Other considerations** | **T-type CCB** | **RAS** | **Relative (95% CI)** | **Absolute** |  |  |
| **Bo Dong 2011 (follow-up 12 months; Better indicated by lower values)** | | | | | | | | | | | | |
| 1 | randomised trials | no serious limitations^1^ | no serious inconsistency | no serious indirectness | no serious imprecision | none | 30 | 30 | - | MD 0.20 lower (10.5 lower to 10.1 higher) | ⊕⊕⊕⊕ HIGH | IMPORTANT |
| **Jian Sheng Gan 2012 (follow-up 6 months; Better indicated by lower values)** | | | | | | | | | | | | |
| 1 | randomised trials | no serious limitations^1^ | no serious inconsistency | no serious indirectness | no serious imprecision | none | 143 | 143 | - | MD 1.20 higher (0.63 to 1.77 higher) | ⊕⊕⊕⊕ HIGH | IMPORTANT |
| **Ming Lian Gong 2012 (follow-up 6 months; Better indicated by lower values)** | | | | | | | | | | | | |
| 1 | randomised trials | no serious limitations^1^ | no serious inconsistency | no serious indirectness | no serious imprecision | none | 45 | 45 | - | MD 10.20 higher (1.31 to 19.09 higher) | ⊕⊕⊕⊕ HIGH | IMPORTANT |

^1^ The number of lost to follow-up and withdrawals was less than 10% defined as high quality; and the rate of lost to follow-up was not significantly difference between the experimental and control groups.

| **T-type CCB compared to RAS for Hypertensive patients with CKD for Proteinuria** | | | | | | |
| --- | --- | --- | --- | --- | --- | --- |
| **Patient or population:** patients with Hypertensive patients with CKD for Proteinuria **Settings:**  **Intervention:** T-type CCB **Comparison:** RAS | | | | | | |
| **Outcomes** | **Illustrative comparative risks* (95% CI)** | | **Relative effect (95% CI)** | **No of Participants (studies)** | **Quality of the evidence (GRADE)** | **Comments**  **(patients of lost to follow-up)** |
|  | Assumed risk | Corresponding risk |  |  |  |  |
|  | **RAS** | **T-type CCB** |  |  |  |  |
| **Koichi Hayashi 2003** Follow-up: 12 months |  | The mean Koichi Hayashi 2003 in the intervention groups was **0.10 higher** (1.04 lower to 1.24 higher) |  | 22 (1 study) | ⊕⊕⊕⊝ **moderate**^1^ | 15 lost in experimental group  10 lost in control group |
| **Koichi Hayashi 2003** Follow-up: 12 |  | The mean Koichi Hayashi 2003 in the intervention groups was **0.30 lower** (1.11 lower to 0.51 higher) |  | 14 (1 study) | ⊕⊕⊕⊝ **moderate**^1^ | 15 lost in experimental group  10 lost in control group |
| **Lucia Del Vecchio 2004** Follow-up: 12 months |  | The mean Lucia Del Vecchio 2004 in the intervention groups was **0.62 higher** (0.04 lower to 1.28 higher) |  | 99 (1 study) | ⊕⊕⊕⊝ **moderate**^1^ | 17 lost in experimental group  18 lost in control group |
| *The basis for the **assumed risk** (e.g. the median control group risk across studies) is provided in footnotes. The **corresponding risk** (and its 95% confidence interval) is based on the assumed risk in the comparison group and the **relative effect** of the intervention (and its 95% CI).  **CI:** Confidence interval; | | | | | | |
| GRADE Working Group grades of evidence **High quality:** Further research is very unlikely to change our confidence in the estimate of effect.  **Moderate quality:** Further research is likely to have an important impact on our confidence in the estimate of effect and may change the estimate. **Low quality:** Further research is very likely to have an important impact on our confidence in the estimate of effect and is likely to change the estimate. **Very low quality:** We are very uncertain about the estimate. | | | | | | |
| ^1^ The number of lost to follow-up and withdrawals was more than 10% defined as moderate quality; and the rate of lost to follow-up was not significantly difference between the experimental and control groups. | | | | | | |

**Author(s):**
**Date:** 2014-03-06
**Question:** Should T-type CCB vs RAS be used for Hypertensive patients with CKD for Proteinuria?
**Settings:**
**Bibliography:**

| **Quality assessment** | | | | | | | **Summary of findings** | | | | | **Importance** |
| --- | --- | --- | --- | --- | --- | --- | --- | --- | --- | --- | --- | --- |
|  |  |  |  |  |  |  | **No of patients** | | **Effect** | | **Quality** |  |
| **No of studies** | **Design** | **Limitations** | **Inconsistency** | **Indirectness** | **Imprecision** | **Other considerations** | **T-type CCB** | **RAS** | **Relative (95% CI)** | **Absolute** |  |  |
| **Koichi Hayashi 2003 (follow-up 12 months; Better indicated by lower values)** | | | | | | | | | | | | |
| 1 | randomised trials | serious^1^ | no serious inconsistency | no serious indirectness | no serious imprecision | none | 11 | 11 | - | MD 0.10 higher (1.04 lower to 1.24 higher) | ⊕⊕⊕O MODERATE | IMPORTANT |
| **Koichi Hayashi 2003 (follow-up 12; Better indicated by lower values)** | | | | | | | | | | | | |
| 1 | randomised trials | serious^1^ | no serious inconsistency | no serious indirectness | no serious imprecision | none | 8 | 6 | - | MD 0.30 lower (1.11 lower to 0.51 higher) | ⊕⊕⊕O MODERATE | IMPORTANT |
| **Lucia Del Vecchio 2004 (follow-up 12 months; Better indicated by lower values)** | | | | | | | | | | | | |
| 1 | randomised trials | serious^1^ | no serious inconsistency | no serious indirectness | no serious imprecision | none | 49 | 50 | - | MD 0.62 higher (0.04 lower to 1.28 higher) | ⊕⊕⊕O MODERATE | IMPORTANT |

^1^ The number of lost to follow-up and withdrawals was more than 10% defined as moderate quality; and the rate of lost to follow-up was not significantly difference between the experimental and control groups.

| **T-type CCB compared to RAS for Hypertensive patients with proteinuria for Proteinuria** | | | | | | |
| --- | --- | --- | --- | --- | --- | --- |
| **Patient or population:** patients with Hypertensive patients with proteinuria for Proteinuria **Settings:**  **Intervention:** T-type CCB **Comparison:** RAS | | | | | | |
| **Outcomes** | **Illustrative comparative risks* (95% CI)** | | **Relative effect (95% CI)** | **No of Participants (studies)** | **Quality of the evidence (GRADE)** | **Comments**  **(patients of lost to follow-up)** |
|  | Assumed risk | Corresponding risk |  |  |  |  |
|  | **RAS** | **T-type CCB** |  |  |  |  |
| **Bo Dong 2011** Follow-up: 12 months |  | The mean Bo Dong 2011 in the intervention groups was **0.01 lower** (0.21 lower to 0.19 higher) |  | 60 (1 study) | ⊕⊕⊕⊕ **high**^1^ | No report |
| **Tao Peng 2009** Follow-up: 12 months |  | The mean Tao Peng 2009 in the intervention groups was **0.12 higher** (0.11 to 0.13 higher) |  | 116 (1 study) | ⊕⊕⊕⊕ **high**^1^ | No report |
| *The basis for the **assumed risk** (e.g. the median control group risk across studies) is provided in footnotes. The **corresponding risk** (and its 95% confidence interval) is based on the assumed risk in the comparison group and the **relative effect** of the intervention (and its 95% CI).  **CI:** Confidence interval; | | | | | | |
| GRADE Working Group grades of evidence **High quality:** Further research is very unlikely to change our confidence in the estimate of effect.  **Moderate quality:** Further research is likely to have an important impact on our confidence in the estimate of effect and may change the estimate. **Low quality:** Further research is very likely to have an important impact on our confidence in the estimate of effect and is likely to change the estimate. **Very low quality:** We are very uncertain about the estimate. | | | | | | |
| ^1^ The number of lost to follow-up and withdrawals was less than 10% defined as high quality; and the rate of lost to follow-up was not significantly difference between the experimental and control groups. | | | | | | |

**Author(s):**
**Date:** 2014-03-06
**Question:** Should T-type CCB vs RAS be used for Hypertensive patients with proteinuria for Proteinuria?
**Settings:**
**Bibliography:**

| **Quality assessment** | | | | | | | **Summary of findings** | | | | | **Importance** |
| --- | --- | --- | --- | --- | --- | --- | --- | --- | --- | --- | --- | --- |
|  |  |  |  |  |  |  | **No of patients** | | **Effect** | | **Quality** |  |
| **No of studies** | **Design** | **Limitations** | **Inconsistency** | **Indirectness** | **Imprecision** | **Other considerations** | **T-type CCB** | **RAS** | **Relative (95% CI)** | **Absolute** |  |  |
| **Bo Dong 2011 (follow-up 12 months; Better indicated by lower values)** | | | | | | | | | | | | |
| 1 | randomised trials | no serious limitations^1^ | no serious inconsistency | no serious indirectness | no serious imprecision | none | 30 | 30 | - | MD 0.01 lower (0.21 lower to 0.19 higher) | ⊕⊕⊕⊕ HIGH | IMPORTANT |
| **Tao Peng 2009 (follow-up 12 months; Better indicated by lower values)** | | | | | | | | | | | | |
| 1 | randomised trials | no serious limitations^1^ | no serious inconsistency | no serious indirectness | no serious imprecision | none | 59 | 57 | - | MD 0.12 higher (0.11 to 0.13 higher) | ⊕⊕⊕⊕ HIGH | IMPORTANT |

^1^ The number of lost to follow-up and withdrawals was less than 10% defined as high quality; and the rate of lost to follow-up was not significantly difference between the experimental and control groups.

**3. Subgroup quality assessment**

| **T-type CCB compared to L-type CCB for Renal Function** | | | | | | |
| --- | --- | --- | --- | --- | --- | --- |
| **Patient or population:** patients with Renal Function **Settings:**  **Intervention:** T-type CCB **Comparison:** L-type CCB | | | | | | |
| **Outcomes** | **Illustrative comparative risks* (95% CI)** | | **Relative effect (95% CI)** | **No of Participants (studies)** | **Quality of the evidence (GRADE)** | **Comments** |
|  | Assumed risk | Corresponding risk |  |  |  |  |
|  | **L-type CCB** | **T-type CCB** |  |  |  |  |
| **GFR** |  | The mean GFR in the intervention groups was **2.2 higher** (0.05 to 4.35 higher) |  | 343 (6 studies) | ⊕⊕⊕⊕ **high**^1^ |  |
| **GFR - Hypertensive patients with CKD** |  | The mean GFR - Hypertensive patients with CKD in the intervention groups was **0.09 higher** (3.14 lower to 3.32 higher) |  | 236 (4 studies) | ⊕⊕⊕⊕ **high**^1^ |  |
| **GFR - Hypertensive patients with diabetic nephropathy** |  | The mean GFR - Hypertensive patients with diabetic nephropathy in the intervention groups was **3.87 higher** (0.99 to 6.75 higher) |  | 107 (2 studies) | ⊕⊕⊕⊕ **high**^1^ |  |
| **SCr** |  | The mean SCr in the intervention groups was **0.02 lower** (0.07 lower to 0.03 higher) |  | 506 (9 studies) | ⊕⊕⊕⊕ **high**^1^ |  |
| **SCr - Hypertensive patients** |  | The mean SCr - Hypertensive patients in the intervention groups was **0 higher** (0.06 lower to 0.07 higher) |  | 180 (3 studies) | ⊕⊕⊕⊕ **high**^1^ |  |
| **SCr - Hypertensive patients with CKD** |  | The mean SCr - Hypertensive patients with CKD in the intervention groups was **0.05 lower** (0.13 lower to 0.03 higher) |  | 326 (6 studies) | ⊕⊕⊕⊕ **high**^1^ |  |
| **Aldosterone** |  | The mean Aldosterone in the intervention groups was **15.19 lower** (19.65 to 10.72 lower) |  | 649 (9 studies) | ⊕⊕⊕⊕ **high**^1^ |  |
| **Aldosterone - Hypertensive patients** |  | The mean Aldosterone - Hypertensive patients in the intervention groups was **11.32 lower** (17.37 to 5.27 lower) |  | 360 (4 studies) | ⊕⊕⊕⊕ **high**^1^ |  |
| **Aldosterone - Hypertensive patients with CKD** |  | The mean Aldosterone - Hypertensive patients with CKD in the intervention groups was **18.88 lower** (31.2 to 6.56 lower) |  | 182 (3 studies) | ⊕⊕⊕⊕ **high**^1^ |  |
| **Aldosterone - Hypertensive patients with diabetic nephropathy** |  | The mean Aldosterone - Hypertensive patients with diabetic nephropathy in the intervention groups was **20.21 lower** (28.07 to 12.36 lower) |  | 107 (2 studies) | ⊕⊕⊕⊕ **high**^1^ |  |
| **Hypertensive patients with CKD of proteiuria** |  | The mean Hypertensive patients with CKD of proteiuria in the intervention groups was **0.73 lower** (0.88 to 0.57 lower) |  | 137 (3 studies) | ⊕⊕⊕⊕ **high**^1^ |  |
| **Hypertensive patients with CKD of urinary protein to creatinine ratio** |  | The mean Hypertensive patients with CKD of urinary protein to creatinine ratio in the intervention groups was **0.22 lower** (0.41 to 0.03 lower) |  | 148 (3 studies) | ⊕⊕⊕⊕ **high**^1^ |  |
| **Hypertensive patients with diabetic nephropathy of urinary albumin to creatinine ratio** |  | The mean Hypertensive patients with diabetic nephropathy of urinary albumin to creatinine ratio in the intervention groups was **55.38 lower** (86.67 to 24.09 lower) |  | 141 (2 studies) | ⊕⊕⊕⊕ **high**^1^ |  |
| *The basis for the **assumed risk** (e.g. the median control group risk across studies) is provided in footnotes. The **corresponding risk** (and its 95% confidence interval) is based on the assumed risk in the comparison group and the **relative effect** of the intervention (and its 95% CI).  **CI:** Confidence interval; | | | | | | |
| GRADE Working Group grades of evidence **High quality:** Further research is very unlikely to change our confidence in the estimate of effect.  **Moderate quality:** Further research is likely to have an important impact on our confidence in the estimate of effect and may change the estimate. **Low quality:** Further research is very likely to have an important impact on our confidence in the estimate of effect and is likely to change the estimate. **Very low quality:** We are very uncertain about the estimate. | | | | | | |

^1^ We collected published randomized controlled trials or comparative study.

**Author(s):**
**Date:** 2014-03-06
**Question:** T-type CCB vs L-type CCB for Renal Function
**Settings:**
**Bibliography:** . T-type CCB for Renal Function. Cochrane Database of Systematic Reviews [Year], Issue [Issue].

| **Quality assessment** | | | | | | | **Summary of findings** | | | | | **Importance** |
| --- | --- | --- | --- | --- | --- | --- | --- | --- | --- | --- | --- | --- |
|  |  |  |  |  |  |  | **No of patients** | | **Effect** | | **Quality** |  |
| **No of studies** | **Design** | **Limitations** | **Inconsistency** | **Indirectness** | **Imprecision** | **Other considerations** | **T-type CCB** | **L-type CCB** | **Relative (95% CI)** | **Absolute** |  |  |
| **GFR (Better indicated by higher values)** | | | | | | | | | | | | |
| 6 | randomised trials | no serious limitations^1^ | no serious inconsistency | no serious indirectness | no serious imprecision | none | 172 | 171 | - | MD 2.2 higher (0.05 to 4.35 higher) | ⊕⊕⊕⊕ HIGH | IMPORTANT |
| **GFR - Hypertensive patients with CKD (Better indicated by higher values)** | | | | | | | | | | | | |
| 4 | randomised trials | no serious limitations^1^ | no serious inconsistency | no serious indirectness | no serious imprecision | none | 118 | 118 | - | MD 0.09 higher (3.14 lower to 3.32 higher) | ⊕⊕⊕⊕ HIGH | IMPORTANT |
| **GFR - Hypertensive patients with diabetic nephropathy (Better indicated by higher values)** | | | | | | | | | | | | |
| 2 | randomised trials | no serious limitations^1^ | no serious inconsistency | no serious indirectness | no serious imprecision | none | 54 | 53 | - | MD 3.87 higher (0.99 to 6.75 higher) | ⊕⊕⊕⊕ HIGH | IMPORTANT |
| **SCr (Better indicated by lower values)** | | | | | | | | | | | | |
| 9 | randomised trials | no serious limitations^1^ | no serious inconsistency | no serious indirectness | no serious imprecision | none | 252 | 254 | - | MD 0.02 lower (0.07 lower to 0.03 higher) | ⊕⊕⊕⊕ HIGH | IMPORTANT |
| **SCr - Hypertensive patients (Better indicated by lower values)** | | | | | | | | | | | | |
| 3 | randomised trials | no serious limitations^1^ | no serious inconsistency | no serious indirectness | no serious imprecision | none | 90 | 90 | - | MD 0 higher (0.06 lower to 0.07 higher) | ⊕⊕⊕⊕ HIGH | IMPORTANT |
| **SCr - Hypertensive patients with CKD (Better indicated by lower values)** | | | | | | | | | | | | |
| 6 | randomised trials | no serious limitations^1^ | no serious inconsistency | no serious indirectness | no serious imprecision | none | 162 | 164 | - | MD 0.05 lower (0.13 lower to 0.03 higher) | ⊕⊕⊕⊕ HIGH | IMPORTANT |
| **Aldosterone (Better indicated by lower values)** | | | | | | | | | | | | |
| 9 | randomised trials | no serious limitations^1^ | no serious inconsistency | no serious indirectness | no serious imprecision | none | 325 | 324 | - | MD 15.19 lower (19.65 to 10.72 lower) | ⊕⊕⊕⊕ HIGH | IMPORTANT |
| **Aldosterone - Hypertensive patients (Better indicated by lower values)** | | | | | | | | | | | | |
| 4 | randomised trials | no serious limitations^1^ | no serious inconsistency | no serious indirectness | no serious imprecision | none | 180 | 180 | - | MD 11.32 lower (17.37 to 5.27 lower) | ⊕⊕⊕⊕ HIGH | IMPORTANT |
| **Aldosterone - Hypertensive patients with CKD (Better indicated by lower values)** | | | | | | | | | | | | |
| 3 | randomised trials | no serious limitations^1^ | no serious inconsistency | no serious indirectness | no serious imprecision | none | 91 | 91 | - | MD 18.88 lower (31.2 to 6.56 lower) | ⊕⊕⊕⊕ HIGH | IMPORTANT |
| **Aldosterone - Hypertensive patients with diabetic nephropathy (Better indicated by lower values)** | | | | | | | | | | | | |
| 2 | randomised trials | no serious limitations^1^ | no serious inconsistency | no serious indirectness | no serious imprecision | none | 54 | 53 | - | MD 20.21 lower (28.07 to 12.36 lower) | ⊕⊕⊕⊕ HIGH | IMPORTANT |
| **Hypertensive patients with CKD of proteiuria (Better indicated by lower values)** | | | | | | | | | | | | |
| 3 | randomised trials | no serious limitations^1^ | no serious inconsistency | no serious indirectness | no serious imprecision | none | 67 | 70 | - | MD 0.73 lower (0.88 to 0.57 lower) | ⊕⊕⊕⊕ HIGH | IMPORTANT |
| **Hypertensive patients with CKD of urinary protein to creatinine ratio (Better indicated by lower values)** | | | | | | | | | | | | |
| 3 | randomised trials | no serious limitations^1^ | no serious inconsistency | no serious indirectness | no serious imprecision | none | 74 | 74 | - | MD 0.22 lower (0.41 to 0.03 lower) | ⊕⊕⊕⊕ HIGH | IMPORTANT |
| **Hypertensive patients with diabetic nephropathy of urinary albumin to creatinine ratio (Better indicated by lower values)** | | | | | | | | | | | | |
| 2 | randomised trials | no serious limitations^1^ | no serious inconsistency | no serious indirectness | no serious imprecision | none | 88 | 53 | - | MD 55.38 lower (86.67 to 24.09 lower) | ⊕⊕⊕⊕ HIGH | IMPORTANT |

^1^ We collected published randomized controlled trials or comparative study.

| **T-type CCB compared to RAS for Renal Function** | | | | | | |
| --- | --- | --- | --- | --- | --- | --- |
| **Patient or population:** patients with Renal Function **Settings:**  **Intervention:** T-type CCB **Comparison:** RAS | | | | | | |
| **Outcomes** | **Illustrative comparative risks* (95% CI)** | | **Relative effect (95% CI)** | **No of Participants (studies)** | **Quality of the evidence (GRADE)** | **Comments** |
|  | Assumed risk | Corresponding risk |  |  |  |  |
|  | **RAS** | **T-type CCB** |  |  |  |  |
| **GFR** |  | The mean GFR in the intervention groups was **0.1 higher** (2.17 lower to 2.37 higher) |  | 376 (3 studies) | ⊕⊕⊕⊕ **high**^1^ |  |
| **Albuminuria** |  | The mean Albuminuria in the intervention groups was **0.14 higher** (8.26 lower to 8.53 higher) |  | 170 (2 studies) | ⊕⊕⊕⊕ **high**^1^ |  |
| **CCr** |  | The mean CCr in the intervention groups was **0.9 lower** (2.38 lower to 0.59 higher) |  | 366 (2 studies) | ⊕⊕⊕⊕ **high**^1^ |  |
| **SCr** |  | The mean SCr in the intervention groups was **2.93 higher** (2.31 lower to 8.17 higher) |  | 436 (3 studies) | ⊕⊕⊕⊕ **high**^1^ |  |
| **Proteinuria** |  | The mean Proteinuria in the intervention groups was **0.12 higher** (0.11 to 0.13 higher) |  | 311 (4 studies) | ⊕⊕⊕⊕ **high**^1^ |  |
| **Proteinuria - Hypertensive patients with CKD** |  | The mean Proteinuria - Hypertensive patients with CKD in the intervention groups was **0.23 higher** (0.24 lower to 0.69 higher) |  | 135 (2 studies) | ⊕⊕⊕⊕ **high**^1^ |  |
| **Proteinuria - Hypertensive patients with proteinuria** |  | The mean Proteinuria - Hypertensive patients with proteinuria in the intervention groups was **0.12 higher** (0.11 to 0.13 higher) |  | 176 (2 studies) | ⊕⊕⊕⊕ **high**^1^ |  |
| *The basis for the **assumed risk** (e.g. the median control group risk across studies) is provided in footnotes. The **corresponding risk** (and its 95% confidence interval) is based on the assumed risk in the comparison group and the **relative effect** of the intervention (and its 95% CI).  **CI:** Confidence interval; | | | | | | |
| GRADE Working Group grades of evidence **High quality:** Further research is very unlikely to change our confidence in the estimate of effect.  **Moderate quality:** Further research is likely to have an important impact on our confidence in the estimate of effect and may change the estimate. **Low quality:** Further research is very likely to have an important impact on our confidence in the estimate of effect and is likely to change the estimate. **Very low quality:** We are very uncertain about the estimate. | | | | | | |
| ^1^ We collected published randomized controlled trials or comparative study. | | | | | | |

**Author(s):**
**Date:** 2014-03-06
**Question:** T-type CCB vs RAS for Renal Function
**Settings:**
**Bibliography:** . T-type CCB for Renal Function. Cochrane Database of Systematic Reviews [Year], Issue [Issue].

| **Quality assessment** | | | | | | | **Summary of findings** | | | | | **Importance** |
| --- | --- | --- | --- | --- | --- | --- | --- | --- | --- | --- | --- | --- |
|  |  |  |  |  |  |  | **No of patients** | | **Effect** | | **Quality** |  |
| **No of studies** | **Design** | **Limitations** | **Inconsistency** | **Indirectness** | **Imprecision** | **Other considerations** | **T-type CCB** | **RAS** | **Relative (95% CI)** | **Absolute** |  |  |
| **GFR (Better indicated by higher values)** | | | | | | | | | | | | |
| 3 | randomised trials | no serious limitations^1^ | no serious inconsistency | no serious indirectness | no serious imprecision | none | 188 | 188 | - | MD 0.1 higher (2.17 lower to 2.37 higher) | ⊕⊕⊕⊕ HIGH | IMPORTANT |
| **Albuminuria (Better indicated by lower values)** | | | | | | | | | | | | |
| 2 | randomised trials | no serious limitations^1^ | no serious inconsistency | no serious indirectness | no serious imprecision | none | 85 | 85 | - | MD 0.14 higher (8.26 lower to 8.53 higher) | ⊕⊕⊕⊕ HIGH | IMPORTANT |
| **CCr (Better indicated by lower values)** | | | | | | | | | | | | |
| 2 | randomised trials | no serious limitations^1^ | no serious inconsistency | no serious indirectness | no serious imprecision | none | 183 | 183 | - | MD 0.9 lower (2.38 lower to 0.59 higher) | ⊕⊕⊕⊕ HIGH | IMPORTANT |
| **SCr (Better indicated by lower values)** | | | | | | | | | | | | |
| 3 | randomised trials | no serious limitations^1^ | no serious inconsistency | no serious indirectness | no serious imprecision | none | 218 | 218 | - | MD 2.93 higher (2.31 lower to 8.17 higher) | ⊕⊕⊕⊕ HIGH | IMPORTANT |
| **Proteinuria (Better indicated by lower values)** | | | | | | | | | | | | |
| 4 | randomised trials | no serious limitations^1^ | no serious inconsistency | no serious indirectness | no serious imprecision | none | 157 | 154 | - | MD 0.12 higher (0.11 to 0.13 higher) | ⊕⊕⊕⊕ HIGH | IMPORTANT |
| **Proteinuria - Hypertensive patients with CKD (Better indicated by lower values)** | | | | | | | | | | | | |
| 2 | randomised trials | no serious limitations^1^ | no serious inconsistency | no serious indirectness | no serious imprecision | none | 68 | 67 | - | MD 0.23 higher (0.24 lower to 0.69 higher) | ⊕⊕⊕⊕ HIGH | IMPORTANT |
| **Proteinuria - Hypertensive patients with proteinuria (Better indicated by lower values)** | | | | | | | | | | | | |
| 2 | randomised trials | no serious limitations^1^ | no serious inconsistency | no serious indirectness | no serious imprecision | none | 89 | 87 | - | MD 0.12 higher (0.11 to 0.13 higher) | ⊕⊕⊕⊕ HIGH | IMPORTANT |

^1^ We collected published randomized controlled trials or comparative study.
