# Supplementary material for: Effects of T-Type Calcium Channel Blockers on Renal Function and Aldosterone in Patients with Hypertension: A Systematic Review and Meta-Analysis
Source: PLoS One. 2014 Oct 17;9(10):e109834. doi: 10.1371/journal.pone.0109834 (PMC4201480; doi:10.1371/journal.pone.0109834)
Supplement: Table S2 — The risk of bias assessment for each included study. (DOC) [file pone.0109834.s004.doc]

**Table S2**

**The risk of bias assessment for each included study**

1. **T-type CCBs vs L-type CCBs**

### Guido Bellinghieri 2003

| **Item** | **Judgement** | **Description** |
| --- | --- | --- |
| Adequate sequence generation? | Yes | Quote:"Randomization was performed centrally by computer-generated code"  Comment: multi-central randomization |
| Allocation concealment? | Yes | Quote:"Randomization was performed centrally by computer-generated code"  Comment: multi-central randomization |
| Blinding? | Yes | Quote:"double-blind, randomized controlled study" Comment: definitely yes |
| Incomplete outcome data addressed? | Yes | Quote:"The number of patients that lost to follow-up was carefully described.16 lost in experimental group,15 lost in control group" Comment: the remaining patients were 100% follow up |
| Free of selective reporting? | Yes | All outcomes listed in methods section are reported on in the results section |
| Free of other bias? | Yes | Study was stopped for insufficient accrual but not for benefit |
| The number of lost to follow-up and withdrawals was not significant? | Unclear | 16 lost in experimental group,15 lost in control group |

### Hajime Ueshiba 2004

| **Item** | **Judgement** | **Description** |
| --- | --- | --- |
| Adequate sequence generation? | Yes | Quote:"Randomization was successful without significant differences in the baseline characteristics. All results was based on intention to treatment analyses" |
| Allocation concealment? | Yes | Randomization was done on an intention-to-treat basis |
| Blinding? | Yes | Quote:"double-blind, randomized controlled study"  Comment: probably yes |
| Incomplete outcome data addressed? | Yes | All outcomes of interest reported |
| Free of selective reporting? | Yes | All outcomes listed in methods section are reported on in the results section |
| Free of other bias? | Yes | Study not reported |
| The number of lost to follow-up and withdrawals was not significant? | Yes | No patient was lost to follow-up and no patient refused to continue in the study |

### Hidehisa Sasaki 2009

| **Item** | **Judgement** | **Description** |
| --- | --- | --- |
| Adequate sequence generation? | Yes | Quote:"Open, randomization was performed centrally by computer-generated code" Comment: definitely yes |
| Allocation concealment? | Yes | Quote:"Open, randomization was performed centrally by computer-generated code" Comment: definitely yes |
| Blinding? | Yes | Quote:"double-blind, randomized controlled study"  Comment: probably yes |
| Incomplete outcome data addressed? | Yes | All outcomes of interest reported |
| Free of selective reporting? | Yes | All outcomes listed in methods section are reported on in the results section |
| Free of other bias? | Yes | Study not reported |
| The number of lost to follow-up and withdrawals was not significant? | Yes | No patient was lost to follow-up and no patient refused to continue in the study |

### Martinez-Martin 2008

| **Item** | **Judgement** | **Description** |
| --- | --- | --- |
| Adequate sequence generation? | Yes | Quote:"This trial was a prospective, randomized, open-label,double-blind " |
| Allocation concealment? | Yes | Quote:"This trial was a prospective, randomized, open-label,double-blind " |
| Blinding? | Yes | Quote:"This trial was a prospective, randomized, open-label,double-blind " |
| Incomplete outcome data addressed? | Yes | Quote:"The number of patients that lost to follow-up was carefully described. 7 lost in experimental group,10 lost in control group"  Comment: the remaining patients were 100% follow up |
| Free of selective reporting? | Yes | All outcomes listed in methods section are reported on in the results section |
| Free of other bias? | Yes | Study was stopped for insufficient accrual but not for benefit |
| The number of lost to follow-up and withdrawals was not significant? | Unclear | 7 lost in experimental group,10 lost in control group |

### Masanori Abe 2009

| **Item** | **Judgement** | **Description** |
| --- | --- | --- |
| Adequate sequence generation? | Yes | Quote:"single-center, prospective, randomized, open-labeled clinical trial." |
| Allocation concealment? | Yes | Quote:"single-center, prospective, randomized, open-labeled clinical trial." |
| Blinding? | Yes | Quote:"Randomized, A single-blind trial" Comment: definitely yes |
| Incomplete outcome data addressed? | Yes | All outcomes listed in methods section are reported on in the results section |
| Free of selective reporting? | Yes | All outcomes of interest reported |
| Free of other bias? | Yes | No reported |
| The number of lost to follow-up and withdrawals was not significant? | Yes | No patient was lost to follow-up and no patient refused to continue in the study |

### Masanori Abe 2011

| **Item** | **Judgement** | **Description** |
| --- | --- | --- |
| Adequate sequence generation? | Yes | Quote:"an open-label and randomized trial." |
| Allocation concealment? | Yes | Quote:"an open-label and randomized trial." |
| Blinding? | Yes | Quote:"Randomized, A single-blind trial" Comment: definitely yes |
| Incomplete outcome data addressed? | Yes | Quote:"The number of patients that lost to follow-up was carefully described. 2 lost in experimental group, 2 lost in control group"  Comment: the remaining patients were 100% follow up |
| Free of selective reporting? | Yes | All outcomes of interest reported |
| Free of other bias? | Yes | Study was stopped for insufficient accrual but not for benefitNo reported |
| The number of lost to follow-up and withdrawals was not significant? | Yes | 2 lost in experimental group, 2 lost in control group |

### Masanori Abe 2011.6

| **Item** | **Judgement** | **Description** |
| --- | --- | --- |
| Adequate sequence generation? | Yes | Quote:"Randomization was done on an intention-to-treat basis" |
| Allocation concealment? | Yes | Quote:"Randomization was done on an intention-to-treat basis" |
| Blinding? | Unclear | no mention |
| Incomplete outcome data addressed? | Yes | All outcomes listed in methods section are reported on in the results section |
| Free of selective reporting? | Yes | All outcomes of interest reported |
| Free of other bias? | Yes | Study was stopped for insufficient accrual but not for benefit |
| The number of lost to follow-up and withdrawals was not significant? | Yes | No patient was lost to follow-up and no patient refused to continue in the study |

### Nobuyuki Nakano 2010

| **Item** | **Judgement** | **Description** |
| --- | --- | --- |
| Adequate sequence generation? | Yes | Quote:"This was a randomized, double-blind, single-center study" |
| Allocation concealment? | Yes | Quote:"This was a randomized, double-blind, single-center study" |
| Blinding? | Yes | Quote:"This was a randomized, double-blind, single-center study" |
| Incomplete outcome data addressed? | Yes | All outcomes listed in methods section are reported on in the results section |
| Free of selective reporting? | Yes | All outcomes listed in methods section are reported on in the results section |
| Free of other bias? | Yes | Study was stopped for insufficient accrual but not for benefit |
| The number of lost to follow-up and withdrawals was not significant? | Yes | Patient follow-up was complete when a final assessment form was received via the online data system or a death report was received. For all patients not completing the final assessment visit, lost to follow-up, or withdrawn, data were censored according to last visit date. |

### Tadashi Konoshita 2013

| **Item** | **Judgement** | **Description** |
| --- | --- | --- |
| Adequate sequence generation? | Yes | Quote:"a randomized crossover study" |
| Allocation concealment? | Yes | Quote:"a randomized crossover study" |
| Blinding? | Yes | Quote:"double-blind, randomized controlled study"  Comment: probably yes |
| Incomplete outcome data addressed? | Yes | All outcomes listed in methods section are reported on in the results section |
| Free of selective reporting? | Yes | All outcomes of interest reported |
| Free of other bias? | Yes | Study was stopped for insufficient accrual but not for benefit |
| The number of lost to follow-up and withdrawals was not significant? | Unclear | 8 lost to follow-up and no patient refused to continue in the study |

### Takayoshi Tsutamoto 2009

| **Item** | **Judgement** | **Description** |
| --- | --- | --- |
| Adequate sequence generation? | Yes | Quote:"Randomization was done on an intention-to-treat basis" |
| Allocation concealment? | Yes | Quote:"Randomization was done on an intention-to-treat basis" |
| Blinding? | Yes | Quote:"Randomized, A single-blind trial" Comment: definitely yes |
| Incomplete outcome data addressed? | Yes | All outcomes listed in methods section are reported on in the results section |
| Free of selective reporting? | Yes | All outcomes of interest reported |
| Free of other bias? | Yes | Study was stopped for insufficient accrual but not for benefit |
| The number of lost to follow-up and withdrawals was not significant? | Yes | No patient was lost to follow-up and no patient refused to continue in the study |

### Tetsuya Oshima 2005

| **Item** | **Judgement** | **Description** |
| --- | --- | --- |
| Adequate sequence generation? | Yes | Quote:"The sequence of treatments was randomized double-blind." |
| Allocation concealment? | Yes | Quote:"This was a randomized, double-blind." |
| Blinding? | Yes | Quote:"This was a randomized, double-blind." |
| Incomplete outcome data addressed? | Yes | All outcomes listed in methods section are reported on in the results section |
| Free of selective reporting? | Yes | All outcomes of interest reported |
| Free of other bias? | Yes | Study was stopped for insufficient accrual but not for benefit |
| The number of lost to follow-up and withdrawals was not significant? | Yes | No patient was lost to follow-up and no patient refused to continue in the study |

### Toshihiko Ishimitsu 2007

| **Item** | **Judgement** | **Description** |
| --- | --- | --- |
| Adequate sequence generation? | Yes | Quote:"The sequence of treatments was randomized double-blind." |
| Allocation concealment? | Yes | Quote:"This was a randomized, double-blind." |
| Blinding? | yes | Quote:"The sequence of treatments was randomized double-blind |
| Incomplete outcome data addressed? | Yes | All outcomes listed in methods section are reported on in the results section |
| Free of selective reporting? | Yes | All outcomes of interest reported |
| Free of other bias? | Yes | No reported |
| The number of lost to follow-up and withdrawals was not significant? | Yes | No patient was lost to follow-up and no patient refused to continue in the study |

### Toshinari Tanaka 2007

| **Item** | **Judgement** | **Description** |
| --- | --- | --- |
| Adequate sequence generation? | No | Quote:"The sequence of treatments was a randomized, placebo-controlled designed" Comment: definitely no |
| Allocation concealment? | Yes | Quote:"The study conformed to good clinical practice guidelines and was done in accord with the Declaration of Helsinki" |
| Blinding? | Yes | Quote:"Double-blind prospective study"  Comment: definitely yes |
| Incomplete outcome data addressed? | Yes | All outcomes listed in methods section are reported on in the results section |
| Free of selective reporting? | Yes | All outcomes of interest reported |
| Free of other bias? | Yes | No reported |
| The number of lost to follow-up and withdrawals was not significant? | Yes | Patient follow-up was complete when a final assessment form was received via the online data system or a death report was received. For all patients not completing the final assessment visit, lost to follow-up, or withdrawn, data were censored according to last visit date. |

### Tsukasa Nakamura 2007

| **Item** | **Judgement** | **Description** |
| --- | --- | --- |
| Adequate sequence generation? | Yes | Quote:"a prospective, randomized, open, masked-endpoint trial was designed" |
| Allocation concealment? | Yes | Quote:"a prospective, randomized, open, masked-endpoint trial was designed, which is similar to routine clinical practice"  Comment: definitely yes |
| Blinding? | Unclear | no mention |
| Incomplete outcome data addressed? | Yes | All outcomes listed in methods section are reported on in the results section |
| Free of selective reporting? | Yes | All outcomes of interest reported |
| Free of other bias? | Yes | No reported |
| The number of lost to follow-up and withdrawals was not significant? | Yes | No patient was lost to follow-up and no patient refused to continue in the study |

### Tsukasa Nakamura 2010

| **Item** | **Judgement** | **Description** |
| --- | --- | --- |
| Adequate sequence generation? | Yes | Quote: "Study was an investigator-designed, prospective, double-bind, randomized, parallel-group trial" |
| Allocation concealment? | Yes | Quote:"an investigator-designed, prospective, double-blind, randomized, parallel group trial" Comment: definitely yes |
| Blinding? | yes | Quote:"an investigator-designed, prospective, double-blind, randomized, parallel group trial" Comment: definitely yes |
| Incomplete outcome data addressed? | Yes | Quote:"No patients was lost to follow-up"  Comment:100% follow up |
| Free of selective reporting? | Yes | All outcomes of interest reported |
| Free of other bias? | Yes | All outcomes of interest reported |
| The number of lost to follow-up and withdrawals was not significant? | Yes | No patients was lost to follow-up |

### Tsukasa Nakamura 2011

| **Item** | **Judgement** | **Description** |
| --- | --- | --- |
| Adequate sequence generation? | Yes | Quote:"Randomization was performed by the intention-to-treat analysis" |
| Allocation concealment? | Yes | Quote:"this trial used the principles outlined in the Helsinki declaration" |
| Blinding? | Unclear | no mention |
| Incomplete outcome data addressed? | Yes | Quote:"No patients was lost to follow-up"  Comment:100% follow up |
| Free of selective reporting? | Yes | All outcomes of interest reported |
| Free of other bias? | Yes | All outcomes of interest reported |
| The number of lost to follow-up and withdrawals was not significant? | Yes | Patient follow-up was complete when a final assessment form was received via the online data system or a death report was received. For all patients not completing the final assessment visit, lost to follow-up, or withdrawn, data were censored according to last visit date. |

### Tsuneo Takenaka 2012

| **Item** | **Judgement** | **Description** |
| --- | --- | --- |
| Adequate sequence generation? | Yes | Quote: "Study was a prospective, double-bind, randomized, parallel-group trial" |
| Allocation concealment? | Yes | Quote:"a prospective, randomized , open was designed, which is similar to routine clinical practice"  Comment: definitely yes |
| Blinding? | Unclear | Quote:"double-blind, placebo controlled study"  Comment: probably no |
| Incomplete outcome data addressed? | Yes | All outcomes listed in methods section are reported on in the results section |
| Free of selective reporting? | Yes | All outcomes of interest reported |
| Free of other bias? | Yes | Study not reported |
| The number of lost to follow-up and withdrawals was not significant? | Yes | 1 lost in experimental group,2 lost in control group |

1. **T-type CCBs vs RAS antagonists**

### Tao Peng 2009

| **Item** | **Judgement** | **Description** |
| --- | --- | --- |
| Adequate sequence generation? | Yes | Quote:"Randomization was previously designed" |
| Allocation concealment? | Yes | Quote:"Randomization was previously designed" |
| Blinding? | No | Quote:"the lack of blinding of the study." |
| Incomplete outcome data addressed? | Yes | All outcomes listed in methods section are reported on in the results section |
| Free of selective reporting? | Yes | All outcomes of interest reported |
| Free of other bias? | Yes | No reported |
| The number of lost to follow-up and withdrawals was not significant? | Yes | No patient was lost to follow-up and no patient refused to continue in the study |

Bo Dong 2011

| **Item** | **Judgement** | **Description** |
| --- | --- | --- |
| Adequate sequence generation? | Yes | Quote:"Randomization was done on an intention-to-treat basis" |
| Allocation concealment? | Yes | Quote:"Randomization was done on an intention-to-treat basis" |
| Blinding? | Unclear | Quote:"Randomization was designed as an open-label, randomized prospective trial" |
| Incomplete outcome data addressed? | Yes | All outcomes listed in methods section are reported on in the results section |
| Free of selective reporting? | Yes | All outcomes of interest reported |
| Free of other bias? | Yes | No reported |
| The number of lost to follow-up and withdrawals was not significant? | Yes | No patient was lost to follow-up and no patient refused to continue in the study |

### Jian Sheng Gan 2012

| **Item** | **Judgement** | **Description** |
| --- | --- | --- |
| Adequate sequence generation? | Yes | Quote:"Randomization was done on an intention-to-treat basis" |
| Allocation concealment? | Yes | Quote:"Randomization was done on an intention-to-treat basis" |
| Blinding? | Unclear | no mention |
| Incomplete outcome data addressed? | Yes | All outcomes listed in methods section are reported on in the results section |
| Free of selective reporting? | Yes | All outcomes of interest reported |
| Free of other bias? | Yes | No reported |
| The number of lost to follow-up and withdrawals was not significant? | Yes | No patient was lost to follow-up and no patient refused to continue in the study |

### Koichi Hayashi 2003

| **Item** | **Judgement** | **Description** |
| --- | --- | --- |
| Adequate sequence generation? | Yes | Quote:"Analysis was by intention to treat" |
| Allocation concealment? | Yes | Quote:"Analysis was by intention to treat" |
| Blinding? | Unclear | Quote:"Randomized, double-blind, multicenter clinical trial"  Comment: probably no |
| Incomplete outcome data addressed? | Yes | All outcomes listed in methods section are reported on in the results section |
| Free of selective reporting? | Yes | All outcomes of interest reported |
| Free of other bias? | Yes | No reported |
| The number of lost to follow-up and withdrawals was not significant? | Unclear | 15 lost in experimental group,10 lost in control group |

### Lucia Del Vecchio 2004

| **Item** | **Judgement** | **Description** |
| --- | --- | --- |
| Adequate sequence generation? | Yes | Quote:"a prospective, randomized , open, double-blind was designed" |
| Allocation concealment? | Yes | Quote:"a prospective, randomized , open, double-blind was designed" |
| Blinding? | Yes | Quote:"Randomized, double-blind, multicenter clinical trial"  Comment: definitely yes |
| Incomplete outcome data addressed? | Yes | Quote:"The number of patients that lost to follow-up was carefully described.17 lost in experimental group,18 lost in control group"  Comment: the remaining patients were 100% follow up |
| Free of selective reporting? | Yes | All outcomes listed in methods section are reported on in the results section |
| Free of other bias? | Yes | Study was stopped for insufficient accrual but not for benefit |
| The number of lost to follow-up and withdrawals was not significant? | Unclear | 17 lost in experimental group,18 lost in control group |

### Ming Lian Gong 2012

| **Item** | **Judgement** | **Description** |
| --- | --- | --- |
| Adequate sequence generation? | Yes | Quote:"Randomization was done on an intention-to-treat basis" |
| Allocation concealment? | Yes | Quote:"Randomization was done on an intention-to-treat basis" |
| Blinding? | Unclear | no mention |
| Incomplete outcome data addressed? | Yes | All outcomes listed in methods section are reported on in the results section |
| Free of selective reporting? | Yes | All outcomes of interest reported |
| Free of other bias? | Yes | No reported |
| The number of lost to follow-up and withdrawals was not significant? | Yes | No patient was lost to follow-up and no patient refused to continue in the study |

### Rong Qi Han 2013

| **Item** | **Judgement** | **Description** |
| --- | --- | --- |
| Adequate sequence generation? | Yes | Quote:"Randomization was done on an intention-to-treat basis" |
| Allocation concealment? | Yes | Quote:"Randomization was done on an intention-to-treat basis" |
| Blinding? | Unclear | no mention |
| Incomplete outcome data addressed? | Yes | All outcomes listed in methods section are reported on in the results section |
| Free of selective reporting? | Yes | All outcomes of interest reported |
| Free of other bias? | Yes | No reported |
| The number of lost to follow-up and withdrawals was not significant? | Yes | No patient was lost to follow-up and no patient refused to continue in the study |
