# Supplementary material for: Effects of T-Type Calcium Channel Blockers on Renal Function and Aldosterone in Patients with Hypertension: A Systematic Review and Meta-Analysis
Source: PLoS One. 2014 Oct 17;9(10):e109834. doi: 10.1371/journal.pone.0109834 (PMC4201480; doi:10.1371/journal.pone.0109834)
Supplement: File S1 — Sensitivity analysis. (DOC) [file pone.0109834.s005.doc]

**File S1**

**Sensitivity analysis**

1. **Recalculated by omitting one study at a time**

1. T-type CCBs vs L-type CCBs: Hypertensive patients for Aldosterone (p=0.0002 to 0.0003), for overall effect (p<1×10^-5^).

2. T-type CCBs vs RAS antagonists: Hypertensive patients with CKD for proteinuria (p=0.34 to 0.62), for overall effect (p<1×10^-5^).

1. **Change the model (from fixed-effect model to random-effect model)**

1. T-type CCBs vs L-type CCBs: Hypertensive patients with CKD for GFR (p=0.96), Hypertensive patients with diabetic nephropathy for GFR (p=0.008), for overall effect (p=0.05).

2. T-type CCBs vs RAS antagonists: Hypertensive patients with proteinuria for Albuminuria (p=0.97).

1. **Loss-to-follow-up**

1. T-type CCBs vs L-type CCBs: Hypertensive patients with CKD for SCr (p=0.27 to 0.20), for overall effect (p=0.45 to 0.53).

2. T-type CCBs vs RAS antagonists: Hypertensive patients with CKD for proteinuria (p=0.34 to 0.33), for overall effect (p<1×10^-5^).
