# Supplementary material for: Effects of T-Type Calcium Channel Blockers on Renal Function and Aldosterone in Patients with Hypertension: A Systematic Review and Meta-Analysis
Source: PLoS One. 2014 Oct 17;9(10):e109834. doi: 10.1371/journal.pone.0109834 (PMC4201480; doi:10.1371/journal.pone.0109834)
Supplement: File S2 — Web resources of twenty-four studies included in the meta-analysis. (DOC) [file pone.0109834.s006.doc]

**File S2**

**Web resources of twenty-four studies included in the meta-analysis.**

The URLs for data presented herein are as follows:

12. Konoshita T, Makino Y, Kimura T, Fujii M, Morikawa N, et al. (2013) A Crossover Comparison of Urinary Albumin Excretion as a New Surrogate Marker for Cardiovascular Disease among 4 Types of Calcium Channel Blockers. Int J Cardiol 166:448-452.

From [EBSCO(asp/bsp)](http://fjour.blyun.com/readenmag.jsp?dxid=165180371648&aid=170&gj=1&type=41&d=2136C4A8701D7C0059337451D79C7836) homepage, <http://search.ebscohost.com/>, or [ScienceDirect](http://fjour.blyun.com/readenmag.jsp?dxid=165180371648&aid=170&gj=1&type=26&d=3732AC9E673867F5C5E94725CF3420F5) homepage, <http://www.sciencedirect.com/>

13. Takenaka T, Seto T, Okayama M, Kojima E, Nodaira Y, et al. (2012) Long-Term Effects of Calcium Antagonists on Augmentation Index in Hypertensive Patients with Chronic Kidney Disease: A Randomized Controlled Study. Am J Nephrol 35:416-423.

From [PubMed](http://fjour.blyun.com/readenmag.jsp?dxid=165153168259&aid=170&gj=1&type=68&d=74CEF36D89F959D4331ECE5A962D8192) homepage, <http://www.ncbi.nlm.nih.gov/pubmed>

14. Nakamura T, Sato E, Fujiwara N, Kawagoe Y, Koide H, et al. (2011) Calcium Channel Blocker Inhibition of Age and Rage Axis Limits Renal Injury in Nondiabetic Patients with Stage I or Ii Chronic Kidney Disease. Clin Cardiol 34:372-377.

From [PubMed](http://fjour.blyun.com/readenmag.jsp?dxid=165153168259&aid=170&gj=1&type=68&d=74CEF36D89F959D4331ECE5A962D8192) homepage, <http://www.ncbi.nlm.nih.gov/pubmed>

15. Abe M, Okada K, Maruyama N, Matsumoto S, Maruyama T, et al. (2011) Benidipine Reduces Albuminuria and Plasma Aldosterone in Mild-to-Moderate Stage Chronic Kidney Disease with Albuminuria. Hypertens Res 34:268-273.

From [PubMed](http://fjour.blyun.com/readenmag.jsp?dxid=165153168259&aid=170&gj=1&type=68&d=74CEF36D89F959D4331ECE5A962D8192) homepage, <http://www.ncbi.nlm.nih.gov/pubmed>

16. Abe M, Maruyama N, Okada K, Matsumoto S, Matsumoto K, et al. (2011) Additive Antioxidative Effects of Azelnidipine on Angiotensin Receptor Blocker Olmesartan Treatment for Type 2 Diabetic Patients with Albuminuria. Hypertens Res 34:935-941.

From [PubMed](http://fjour.blyun.com/readenmag.jsp?dxid=165153168259&aid=170&gj=1&type=68&d=74CEF36D89F959D4331ECE5A962D8192) homepage, <http://www.ncbi.nlm.nih.gov/pubmed>

17. Nakano N, Ishimitsu T, Takahashi T, Inada H, Okamura A, et al. (2010) Effects of Efonidipine, an L- and T-Type Calcium Channel Blocker, on the Renin-Angiotensin-Aldosterone System in Chronic Hemodialysis Patients. Int Heart J 51:188-192.

From [PubMed](http://fjour.blyun.com/readenmag.jsp?dxid=165153168259&aid=170&gj=1&type=68&d=74CEF36D89F959D4331ECE5A962D8192) homepage, <http://www.ncbi.nlm.nih.gov/pubmed>

18. Nakamura T, Sato E, Fujiwara N, Kawagoe Y, Ueda Y, et al. (2010) Comparative Effects of Benidipine and Amlodipine on Proteinuria, Urinary 8-Ohdg, Urinary L-Fabp, and Inflammatory and Atherosclerosis Markers in Early-Stage Chronic Kidney Disease. Am J Med Sci 339:157-163.

From [PubMed](http://fjour.blyun.com/readenmag.jsp?dxid=165153168259&aid=170&gj=1&type=68&d=74CEF36D89F959D4331ECE5A962D8192) homepage, <http://www.ncbi.nlm.nih.gov/pubmed>

19. Tsutamoto T, Tanaka T, Nishiyama K, Yamaji M, Kawahara C, et al. (2009) Long-Term Effect of Efonidipine Therapy on Plasma Aldosterone and Left Ventricular Mass Index in Patients with Essential Hypertension. Hypertens Res 32:670-674.

From [PubMed](http://fjour.blyun.com/readenmag.jsp?dxid=165153168259&aid=170&gj=1&type=68&d=74CEF36D89F959D4331ECE5A962D8192) homepage, <http://www.ncbi.nlm.nih.gov/pubmed>

20. Sasaki H, Saiki A, Endo K, Ban N, Yamaguchi T, et al. (2009) Protective Effects of Efonidipine, a T- and L-Type Calcium Channel Blocker, on Renal Function and Arterial Stiffness in Type 2 Diabetic Patients with Hypertension and Nephropathy. J Atheroscler Thromb 16:568-575.

From [PubMed](http://fjour.blyun.com/readenmag.jsp?dxid=165153168259&aid=170&gj=1&type=68&d=74CEF36D89F959D4331ECE5A962D8192) homepage, <http://www.ncbi.nlm.nih.gov/pubmed>

21. Abe M, Okada K, Maruyama T, Maruyama N, Matsumoto K (2009) Comparison of the Antiproteinuric Effects of the Calcium Channel Blockers Benidipine and Amlodipine Administered in Combination with Angiotensin Receptor Blockers to Hypertensive Patients with Stage 3-5 Chronic Kidney Disease. Hypertens Res 32:270-275.

From [PubMed](http://fjour.blyun.com/readenmag.jsp?dxid=165153168259&aid=170&gj=1&type=68&d=74CEF36D89F959D4331ECE5A962D8192) homepage, <http://www.ncbi.nlm.nih.gov/pubmed>

22. Martinez-Martin FJ, Saiz-Satjes M (2008) Add-on Manidipine Versus Amlodipine in Diabetic Patients with Hypertension and Microalbuminuria: The Amandha Study. Expert Rev Cardiovasc Ther 6:1347-1355.

From [PubMed](http://fjour.blyun.com/readenmag.jsp?dxid=165153168259&aid=170&gj=1&type=68&d=74CEF36D89F959D4331ECE5A962D8192) homepage, <http://www.ncbi.nlm.nih.gov/pubmed>

23. Tanaka T, Tsutamoto T, Sakai H, Fujii M, Yamamoto T, et al. (2007) Comparison of the Effects of Efonidipine and Amlodipine on Aldosterone in Patients with Hypertension. Hypertens Res 30:691-697.

From [PubMed](http://fjour.blyun.com/readenmag.jsp?dxid=165153168259&aid=170&gj=1&type=68&d=74CEF36D89F959D4331ECE5A962D8192) homepage, <http://www.ncbi.nlm.nih.gov/pubmed>

24. Nakamura T, Sugaya T, Kawagoe Y, Suzuki T, Ueda Y, et al. (2007) Azelnidipine Reduces Urinary Protein Excretion and Urinary Liver-Type Fatty Acid Binding Protein in Patients with Hypertensive Chronic Kidney Disease. Am J Med Sci 333:321-326.

From [PubMed](http://fjour.blyun.com/readenmag.jsp?dxid=165153168259&aid=170&gj=1&type=68&d=74CEF36D89F959D4331ECE5A962D8192) homepage, <http://www.ncbi.nlm.nih.gov/pubmed>

25. Ishimitsu T, Kameda T, Akashiba A, Takahashi T, Ohta S, et al. (2007) Efonidipine Reduces Proteinuria and Plasma Aldosterone in Patients with Chronic Glomerulonephritis. Hypertens Res 30:621-626.

From [PubMed](http://fjour.blyun.com/readenmag.jsp?dxid=165153168259&aid=170&gj=1&type=68&d=74CEF36D89F959D4331ECE5A962D8192) homepage, <http://www.ncbi.nlm.nih.gov/pubmed>

26. Oshima T, Ozono R, Yano Y, Higashi Y, Teragawa H, et al. (2005) Beneficial Effect of T-Type Calcium Channel Blockers on Endothelial Function in Patients with Essential Hypertension. Hypertens Res 28:889-894.

From [PubMed](http://fjour.blyun.com/readenmag.jsp?dxid=165153168259&aid=170&gj=1&type=68&d=74CEF36D89F959D4331ECE5A962D8192) homepage, <http://www.ncbi.nlm.nih.gov/pubmed>

27. Ueshiba H, Miyachi Y (2004) Effects of the Long-Acting Calcium Channel Blockers, Amlodipine, Manidipine and Cilnidipine on Steroid Hormones and Insulin Resistance in Hypertensive Obese Patients. Intern Med 43:561-565.

From [PubMed](http://fjour.blyun.com/readenmag.jsp?dxid=165153168259&aid=170&gj=1&type=68&d=74CEF36D89F959D4331ECE5A962D8192) homepage, <http://www.ncbi.nlm.nih.gov/pubmed>

28. Bellinghieri G, Mazzaglia G, Savica V, Santoro D (2003) Effects of Manidipine and Nifedipine on Blood Pressure and Renal Function in Patients with Chronic Renal Failure: A Multicenter Randomized Controlled Trial. Ren Fail 25:681-689.

From [PubMed](http://fjour.blyun.com/readenmag.jsp?dxid=165153168259&aid=170&gj=1&type=68&d=74CEF36D89F959D4331ECE5A962D8192) homepage, <http://www.ncbi.nlm.nih.gov/pubmed>

29. Han RQ, Luo CL, Jiang M, Li HY, Shan FJ (2013) Observed the efficacy of benidipine combined with benazepril treatment of hypertension nephropathy. Clinical drug of the world 7:401-404. (in Chinese)

From CNKI homepage, <http://www.cnki.net/>

30. Gong ML, Gao YX, Zhang J (2012) Benidipine combined with valsartan on renal function in patients with essential hypertension. Chinese Medicine 14: 95-96+99. (in Chinese)

From CNKI homepage, <http://www.cnki.net/>

31. Gan JS, Gan L (2012) Effect of valsartan and benidipine on renal function in patients with essential hypertension with proteinuria. China Pharmaceutical Guide 27:201-202. (in Chinese)

From CNKI homepage, <http://www.cnki.net/>

32. Dong B, Xiao Qu B (2011) Benidipine and perindopril in elderly hypertensive patients with proteinuria affect renal function. Journal of China Modern Medicine 11:54-56. (in Chinese)

From CNKI homepage, <http://www.cnki.net/>

33. Peng T, Hu Z, Xia Q, Bei Jiang, Xianhua Li, et al. (2009) A Comparative Study of the Renoprotective Effects of Benidipine and Valsartan in Primary Hypertensive Patients with Proteinuria. Arzneimittelforschung 59:647-650.

From [PubMed](http://fjour.blyun.com/readenmag.jsp?dxid=165153168259&aid=170&gj=1&type=68&d=74CEF36D89F959D4331ECE5A962D8192) homepage, <http://www.ncbi.nlm.nih.gov/pubmed>

34. Del Vecchio L, Pozzi M, Salvetti A, Maschio G, Fusaroli M, et al. (2004) Efficacy and Tolerability of Manidipine in the Treatment of Hypertension in Patients with Non-Diabetic Chronic Kidney Disease without Glomerular Disease. Prospective, Randomized, Double-Blind Study of Parallel Groups in Comparison with Enalapril. J Nephrol 17:261-269.

From [PubMed](http://fjour.blyun.com/readenmag.jsp?dxid=165153168259&aid=170&gj=1&type=68&d=74CEF36D89F959D4331ECE5A962D8192) homepage, <http://www.ncbi.nlm.nih.gov/pubmed>

35. Hayashi K, Kumagai H, and Saruta T (2003) Effect of Efonidipine and Ace Inhibitors on Proteinuria in Human Hypertension with Renal Impairment. Am J Hypertens 16: 116-122.

From [PubMed](http://fjour.blyun.com/readenmag.jsp?dxid=165153168259&aid=170&gj=1&type=68&d=74CEF36D89F959D4331ECE5A962D8192) homepage, <http://www.ncbi.nlm.nih.gov/pubmed>
