# Supplementary material for: Effects of T-Type Calcium Channel Blockers on Renal Function and Aldosterone in Patients with Hypertension: A Systematic Review and Meta-Analysis
Source: PLoS One. 2014 Oct 17;9(10):e109834. doi: 10.1371/journal.pone.0109834 (PMC4201480; doi:10.1371/journal.pone.0109834)
Supplement: File S3 — PDF files of twenty-four studies included in the meta-analysis. (ZIP) [file pone.0109834.s007.zip › Supporting information-PDF files/12. Int J Cardiol 2013[166(2)]448-452.pdf]

International Journal of Cardiology 166 (2013) 448–452

Contents lists available at SciVerse ScienceDirect

International Journal of Cardiology

journal homepage: www.elsevier.com/locate/ijcard

A crossover comparison of urinary albumin excretion as a new surrogate marker for cardiovascular disease among 4 types of calcium channel blockers

Tadashi Konoshita *, Yasukazu Makino, Tomoko Kimura, Miki Fujii, Norihiro Morikawa, Shigeyuki Wakahara, Kenichiro Arakawa, Isao Inoki, Hiroyuki Nakamura, Isamu Miyamori
and The Genomic Disease Outcome Consortium (G-DOC) Study Investigators

Third Department of Internal Medicine, Fukui University School of Medicine, Department of Environmental and Preventive Medicine, Kanazawa University Graduate School of Medical Science, Japan

ARTICLE INFO

Article history:
Received 28 July 2011
Received in revised form 3 October 2011
Accepted 30 October 2011
Available online 21 November 2011

Keywords:
Renin–angiotensin system
Calcium channel blocker
L type calcium channel
N type calcium channel
T type calcium channel
Albuminuria

ABSTRACT

Background: At the intervention for cardiovascular disease (CVD), albuminuria is a new pivotal target. Calcium channel blocker (CCB) is one of the most expected agents. Currently CCBs have been classified by delivery system, half-life and channel types. We tested anti-albuminuric effect among 4 types of CCBs.
Methods: Subjects were 50 hypertensives (SBP/DBP 164.7 ± 17.1/92.3 ± 12.2 mm Hg, s-Cr 0.81 ± 0.37 mg/dl, urinary albumin excretion (UAE) 69.4 (33.5–142.6) mg/gCr). Four CCBs were administered in a crossover setting: nifedipine CR, a long biological half-life L type by controlled release; cilnidipine, an N/L type; efonidipine, a T/L type; and amlodipine, a long biological half-life L type.
Results: Comparable BP reductions were obtained. UAE at endpoints ware as follows (mg/gCr, *P<0.01): nifedipine CR 30.8 (17.3–81.1),* cilnidipine 33.9 (18.0–67.7),* efonidipine 51.0 (21.2–129.8), amlodipine 40.6 (18.7–94.7). By all agents, significant augmentations were observed in PRA, angiotensin I and angiotensin II (AngII). AngII at cilnidipine was significantly lower than that at amlodipine. PAC at cilnidipine and efonidipine was significantly lower than that at amlodipine. Nifedipine CR significantly reduced ANP concentration.
Conclusions: It is revealed that only nifedipine CR and cilnidipine could reduce albuminuria statistically. Thus, it is suggested that the 2 CCBs might be favorable for organ protection in hypertensives.
© 2011 Elsevier Ireland Ltd. All rights reserved.

1. Introduction

Recent studies have revealed that proteinuria and albuminuria are risk factors for end stage renal disease and at the same time risk factors for cardiovascular disease (CVD) [1–3]. Reduction in proteinuria and/or albuminuria is associated with a trend in reducing renal death and cardiovascular events [4, 5]. These results extend the new concept that high albuminuria/proteinuria itself should be the target for reducing hard end points just like established treatments for high blood pressure, high blood glucose and high LDL cholesterol. Thus, in other words, at the intervention of hypertension, albuminuria reduction is one of the most pivotal surrogate goals for hypertension treatment as well as strict blood pressure (BP) control for the final goals, reducing renal death and CVD. Blockade of the renin–angiotensin system (RAS) is essential for albuminuria. Agents such as angiotensin converting enzyme (ACEI) and angiotensin II receptor blocker (ARB) reduce albuminuria and consequent renal and cardiovascular events in diabetic subjects [6, 7]. However, in many cases, additional anti-hypertensive drugs are indispensable to obtain the target BP levels. One of the most expected candidates is calcium channel blocker (CCB), which is a useful agent with a sure BP reduction and no crucial adverse effect. Several studies revealed that combination of RAS blockade agents with some CCBs reduce albuminuria additionally [8]. However glomerular hypertension and the RAS activation provoked by CCBs are unfavorable characteristics, because the RAS plays major roles in blood pressure regulation and electrolyte metabolism [9], at the same time, the over-activation of the RAS is thought to play pivotal roles in the pathophysiology of cardiovascular [10], renal [11] and metabolic conditions [12]. The activation of the system by CCB is thought to be inevitable via intra-cellular calcium reduction and sympathetic nerve activation from the transcriptional level of the human renin gene [13–17]. Recently Ca channels are classified into at least five subtypes based on electrophysiological and pharmacological characteristics, namely, L-, N-, P/Q-, R- and T-types [18, 19]. Currently, several types of CCBs are available and have been classified from the respects of biological half-life, drug delivery system and blocking channel types and a new classification of dihydropyridines according to the sympathetic nerve effects has been proposed [20]. The purpose of the study is to compare anti-albuminuric effect as the primary endpoint and the level of the circulatory RAS activation among 4 types of CCBs by a simplified crossover manner.

* Corresponding author at: Third Department of Internal Medicine, Fukui University School of Medicine, 23-3, Matsuokashimoaizuki, Eiheiji, Fukui, 910-1193, Japan. Tel.: +81 776 61 8353; fax: +81 776 61 8111.
E-mail address: konosita@u-fukui.ac.jp (T. Konoshita).

0167-5273/$ – see front matter © 2011 Elsevier Ireland Ltd. All rights reserved.
doi:10.1016/j.ijcard.2011.10.133

2. Materials and methods

2.1. Subjects and treatment

We enrolled 58 consecutive hypertensives of our out clinic into the study and 50 subjects completed the study (Fig. 1). All subjects were Japanese. Subjects with age less than 20 years old, secondary hypertension, acute phase disorder and severe organ failure were excluded. All subjects had been under the condition without any anti-hypertensive or anti-dyslipidemic agents at least 1 week before the first sampling for the study and thereafter anti-dyslipidemic agents were restarted. All other medications needed other than anti-hypertensive drugs were freely prescribed according to each patient disorder. Especially, diabetic subjects, 30.0% of the total subjects, which is thought to be standard prevalence in Japan, continued to receive their usual care for diabetes. A target glycosylated hemoglobin A1c level of less than 6.5% was recommended for all subjects. All subjects were guided to take dietary NaCl less than 6 g daily. Home blood pressure was measured for excluding the subjects with white coat hypertension. At out clinic, on each occasion, blood pressure was taken at least three readings separated by as much time as practical with an automated digital device (Terumo, ES-H51). If readings varied more than 5 mm Hg, additional readings were taken until the last two were close. Diabetes was diagnosed according to the criteria of the World Health Organization. Dyslipidemia was diagnosed according to the criteria of the International Diabetes Federation. The body mass index (BMI) was calculated as the weight in kilograms divided by the square of the height in meters. Arterial hypertension was defined as a systolic blood pressure (SBP) of 140 mm Hg or more or a diastolic blood pressure (DBP) of 90 mm Hg or more in the sitting position on two separate occasions in the morning. Daily 20–60 mg of nifedipine CR or 10–20 mg of cilnidipine or 20–60 mg of efonidipine or 5–10 mg of amlodipine besilate was administered to subjects and titrated to achieve a target blood pressure, 130/80 mm Hg (140/90 mm Hg in the elderly), for 12 weeks in a randomized 4 groups 4 terms cross-over manner (Fig. 1). Actually the subjects were seen twice or thrice in each period and compliance for the medication was checked by interview.

2.2. Study measures

At the baseline day and the last day of the 12 weeks administration of each drug, the items shown in Tables 1, 2 and 3 were examined. The samples were obtained once at each drug tested period in the forenoon. Urinary albumin excretion (UAE) was measured by immunoturbidimetry. At the run-in period, we measured urinary albumin several times and confirmed albuminuria at least 2 times of 3 measurements including the baseline points according to K-DOQI Guidelines 2007 [21]. Estimated glomerular filtration rate (eGFR) was calculated according to the formula for Japanese subjects: eGFR (ml/min/1.73 m^2) $= 194 \times Cr^{-1.094} \times Age^{-0.287}$ ($\times 0.739$, in the case of female). After 15 min of rest in the supine position, blood samples were drawn for the measurement. For avoiding cold activation and degradation of humoral factors, the plasma samples were frozen as soon as possible. For plasma renin activity (PRA) measurement sample were incubated 37 °C for adequate hours and generated angiotensin I was measured by radioimmunoassay with a commercial kit (BML, Japan). Angiotensin I (AngI) and angiotensin II (AngII) were assayed by radioimmunoassay with commercial kits (SRL, Japan). Plasma aldosterone concentration (PAC) and atrial natriuretic peptide (ANP) were assayed by radioimmunoassay with commercial kits (BML, Japan).

2.3. Statistical analyses

As the distribution of UAE, the primary endpoint of the study, is deviated, the needed sample size estimation is difficult. So that it was tentatively calculated estimating a standard deviation for UAE of about 100 mg/gCr; a difference to be detected between groups of 40 mg/gCr and used a bilateral paired Student's *t*-test with protection against type I error of 5% and 80% of power. Thus, it was calculated the study required around 50 subjects in total tentatively. Actually differences of UAE were analyzed by Wilcoxon signed rank test. Statistical analyses were performed with SPSS Version 17.0 (SPSS Japan, Inc., Japan). Data were presented as numbers, percentage, means ± SD or medians (interquartile ranges), as appropriate. The differences between 2 paired continuous variables were analyzed by Student's *t*-test fundamentally or Wilcoxon signed rank test when data do not show normal distribution. All P values are two-sided.

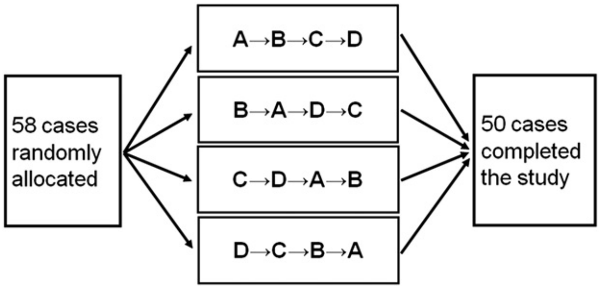

Fig. 1. Schematic protocol of a randomized crossover study. Four types of calcium channel blockers were administered to subjects for 12 weeks each in a randomized 4 groups 4 terms cross-over manner. A: amlodipine, B: cilnidipine, C: efonidipine, D: nifedipine CR.

Table 1
Baseline characteristics of subjects*.

Characteristics	
Number	50
Age — years	69.8 ± 10.8
Male sex — no. (%)	22(44.0%)
Body-mass index†	24.2 ± 3.6
Waist circumference — cm	86.7 ± 10.1
Metabolic syndrome, no. (%)	16 (32.0%)
Diabetes mellitus, no. (%)	15 (30.0%)
Dyslipidemia no. (%)	31 (62.0%)
Glucose — mg/dl	109.3 ± 23.7
Glycosylated hemoglobin — %	5.57 ± 0.79
Triglyceride — mg/dl‡	110 (77.0–145.0)
Cholesterol — mg/dl	
High-density lipoprotein‡	53.5(47.0–61.0)
Low-density lipoprotein	110.6 ± 27.4
Serum creatinine — mg/dl	0.81 ± 0.37
Estimated GFR— ml/min/1.73 m^2	68.7 ± 20.2
Angiotensin converting enzyme — IU/l	14.7 ± 4.3
Urinary natrium excretion — mEq/creatinine‡	203.5 (105.1–282.0)
Urinary kalium excretion — mEq/creatinine‡	49.3 (34.6–80.2)
Urinary chlorine excretion — mEq/creatinine‡	198.8 (108.2–313.1)

* Plus–minus values are means ± SD.
† The body-mass index is the weight in kilograms divided by square of the height in meters.
‡ Values shown are medians (interquartile ranges).

3. Results

3.1. Study course and blood pressure

From 58 enrolled cases, a total of 50 subjects completed the cross-over study; monotherapy of nifedipine CR, cilnidipine, efonidipine or amlodipine besilate by turns for 12 weeks each. Three patients dropped out because of palpitation, eruption or edema in each one case for nifedipine CR. One patient dropped out because of palpitation for cilnidipine. Two patients dropped out because of insufficient blood pressure reduction for efonidipine. Two patients dropped out because of gingivitis or edema in each one case for amlodipine. However, including the 8 cases, no serious adverse effect occurred in the study term. The major demographic and baseline clinical characteristics were summarized in Tables 1, 2 and 3. Final doses of nifedipine CR, cilnidipine, efonidipine and amlodipine were 33.6 ± 9.4 mg/day, 14.1 ± 5.1 mg/day, 44.8 ± 13.7 mg/day and 6.6 ± 2.7 mg/day, respectively. Changes in blood pressure and pulse rate are summarized in Table 2. With each CCB administration, significant reductions in systolic and diastolic blood pressure from baseline were achieved. The BP reductions are comparable between each combination comparison. No significant change was observed in pulse rates from baseline and no significant difference was observed among the drugs.

3.2. Urinary albumin excretion and humoral factors

As shown in Fig. 2, UAE at endpoints of each CCB were as follows: nifedipine CR 30.8 (17.3–81.1), cilnidipine 33.9 (18.0–67.7), efonidipine 51.0 (21.2–129.8), amlodipine 40.6 (18.7–94.7). Thus, in spite of the comparable BP reduction levels, it is revealed that only nifedipine CR and cilnidipine could reduce UAE significantly among 4 tested CCBs. The exact P values from baseline were, 0.002, 0.003, 0.325 and 0.077 for nifedipine CR, cilnidipine, efonidipine and amlodipine, respectively.

With regard to humoral factors (Table 3), by all agents, significant augmentations were observed in PRA, AngI and AngII concentrations. AngII concentration at cilnidipine was significantly lower than that at

Table 2
Blood pressure and heart rate of each drug administration[*].

Characteristics	Baseline	Nifedipine CR	Cilnidipine	Efonidipine	Amlodipine
Blood pressure (mm Hg)					
Systolic	164.7 ± 17.1	139.1 ± 15.2[†]	137.2 ± 13.0[†]	139.5 ± 17.0[†]	137.6 ± 11.6[†]
Diastolic	92.3 ± 12.2	79.6 ± 9.2[†]	79.2 ± 10.1[†]	77.5 ± 12.6[†]	79.0 ± 10.6[†]
Pulse rate (beats/min)	74.0 ± 11.4	73.8 ± 13.4	73.9 ± 11.3	72.9 ± 10.6	74.5 ± 13.2

[*] Plus–minus values are means ± SD.
[†] P<0.05 for the comparison with Baseline. The differences between 2 paired continuous variables were analyzed by Student's *t*-test. All P values are two-sided.

amlodipine. PAC at cilnidipine and efonidipine was significantly lower than that at amlodipine. Nifedipine CR significantly reduced ANP concentration.

4. Discussion

Recent studies have revealed that at the intervention of hypertension, albuminuria reduction is one of the most pivotal surrogate goals for reducing renal death and CVD as well as strict BP control [4, 5]. CCB is one of the most expected agents for albuminuria next to the RAS blocking agents. Especially, N- and T-type Ca channel blocking agents are thought to have dilating effects on efferent glomerular arterioles as well as afferent ones, resulting in lesser glomerular hypertension [22, 23]. However, it has not been clarified which CCB could reduce albuminuria/proteinuria until now. Thereby we tested anti-albuminuric effects of 4 representative types of CCBs; nifedipine CR, a long biological half-life L type CCB with controlled release system, cilnidipine, an N/L type CCB, efonidipine, a T/L type CCB and amlodipine besilate, a long biological half-life L type CCB with trans-membrane approach. Our results revealed that nifedipine CR and cilnidipine could reduce albuminuria. Previous studies demonstrated the reducing effects of albuminuria/proteinuria by nifedipine [24] and cilnidipine [25–27] in the combination of RAS blockade therapy. As a design of monotherapy, albuminuria reduction was observed by cilnidipine compared with amlodipine [28]. Finally, for the first time, our study could indicate that significant albuminuria reduction was obtained by nifedipine CR and cilnidipine in a comparison among 4 types of CCBs as a monotherapy. The difference of effect for morning blood pressure among CCBs might be considered for the results, as the diurnal blood pressure such as morning surge is supposed to be related with UAE [29].

CCB administration is thought to give rise to considerable changes in humoral factors. Especially, at the human renin gene, cAMP response element (CRE) and negative calcium responsive element (nCaRE) has been characterized [13–17] i.e., sympathetic nerve activation and intra-cellular calcium reduction provoked by CCBs up-regulate the transcription of the renin gene via catecholamines-β1 adrenoceptor-cAMP-CREB-CRE pathway and intra-cellular Ca-Ref1-nCaRE pathway. Consequently CCB is thought to give rise to over-activation of whole cascades of the RAS. Our results of the study indicate that, by all CCBs tested, significant augmentations were observed in PRA, AngI and AngII concentrations as expected. However, PRA and AngI at cilnidipine tended to be low compared to those at the other CCBs and Ang II and PAC were significantly lower than those at amlodipine. This phenomenon of less activation of the RAS by cilnidipine compared to amlodipine was already shown in animals and human subjects [30–32] explained by N-type calcium channel's regulation of norepinephrine release [33] and N-type calcium channel suppression and reduction of norepinephrine secretion rate by cilnidipine [34, 35]. On the other hand, PAC at cilnidipine and efonidipine was significantly lower than that at amlodipine. For cilnidipine the result could be explained by the less activation of RAS. A recent report suggests that cilnidipine suppressed the development of proteinuria greater than amlodipine possibly through inhibiting N-type calcium channel-dependent podocyte injury in SHR/ND [36]. For efonidipine, the result might be explained by suppression effect on transcription of aldosterone synthase (CYP11B2) [37]. Finally nifedipine CR significantly reduced ANP concentration. This might be explained by recently suggested mineralocorticoid receptor antagonist activity of a number of dihydropyridine CCB with nifedipine being stronger than amlodipine [38]. Anyway, anti-albuminuric effects could be explained not only by the difference of blocking channel types of each agent but some original pleiotropic effect, for example, effects on transcriptional mechanisms [39, 40].

Several limitations of this study should be noted. Considering the earnest practical limit for clinical human subjects, washout period between the 2 drugs administration was not set up. The sample number may be still relatively small. Although primary endpoint of the study was changes in albuminuria from baseline clearly, we used multiple statistical comparisons for between 2 drugs and humoral factors additionally as sub-analysis with significance level 0.05. Although population admixture is thought to contribute to concordant results among studies, our study is comprised of only Japanese population. On the contrary, inhomogeneous subjects' selection including 30% diabetics could be a source of bias in pathogenic mechanism. Again recently RAS blockade agent is gold standard therapy for prevent microalbuminuria in diabetes [41]. Considering the suggested target

Table 3
Effects of each calcium channel blocker on humoral factors[*].

Characteristics	Baseline	Nifedipine CR	Cilnidipine	Efonidipine	Amlodipine
PRA (ng/ml/h)	0.60 ± 0.71	1.19 ± 1.34[†]	0.97 ± 0.86[†]	1.20 ± 1.63[†]	1.27 ± 1.53[†]
Angiotensin I (pg/ml)	56.8 ± 40.0	107.3 ± 89.4[†]	93.5 ± 72.7[†]	131.5 ± 165.6[†]	98.0 ± 73.2[†]
Angiotensin II(pg/ml)	4.7 ± 4.2	8.3 ± 5.9[†]	8.6 ± 5.5[†,‡]	13.7 ± 18.6[†]	9.0 ± 5.9[†,‡]
PAC (pg/ml)	76.7 ± 41.8	86.5 ± 43.9[§]	75.0 ± 39.5[§,‡]	74.5 ± 30.2[‖]	86.7 ± 45.0[‡,‖]
ANP (pg/ml)	39.3 ± 24.6	29.7 ± 18.2[†,¶,∫]	32.3 ± 19.3[**]	43.1 ± 20.6[∫,**,‖]	36.5 ± 27.3[¶,‖]

[*] Plus-minus values are means ± SD. The differences between 2 paired continuous variables were analyzed by Student's *t*-test. All P values are two-sided.
[†] P<0.05 for the comparison with Baseline.
[‡] P<0.05 for the comparison with Cilnidipine vs Amlodipine.
[§] P<0.05 for the comparison with Nifedipine CR vs Cilnidipine.
[‖] P<0.05 for the comparison with Efonidipine vs Amlodipine.
[¶] P<0.05 for the comparison with Nifedipine CR vs Amlodipine.
[∫] P<0.05 for the comparison with Nifedipine CR vs Efonidipine.
[**] P<0.05 for the comparison with Cilnidipine vs Efonidipine.

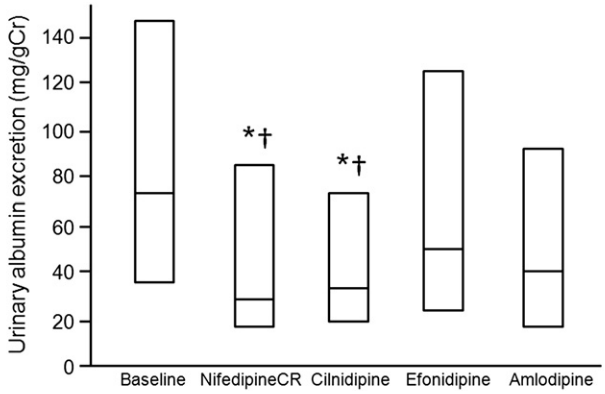

Fig. 2. Urinary albumin excretion at the endpoint of each calcium channel blocker administration. Boxes express the interquartile ranges and horizontal lines in the boxes express the medians. * $P<0.01$ for the comparison with Baseline. †$P<0.05$ for the comparison with Efonidipine. The exact P values from baseline were, 0.002, 0.003, 0.325 and 0.077 for nifedipine CR, cilnidipine, efonidipine and amlodipine, respectively. The differences were analyzed by Wilcoxon signed rank test. All P values are two-sided.

by guidelines and the renal autoregulation [42], the obtained blood pressure reduction might not be sufficient due to the mild target blood pressure in the elderly. Our study design could not provide natural history of albuminuria independent of blood pressure reduction. And one another limitation was that the achieved systolic blood pressure differed by about 2 mm Hg although it did not reach statistic difference.

It is suggested that, in respect of albuminuria reduction, nifedipine CR and cilnidipine may have favorable characteristics for organ protection. These differences seem to be explained by some original pleiotropic effect as well as the blocking channel types of each agent. In addition to albuminuria, other organ damage markers such as left ventricular hypertrophy and retinal changes might be useful for evaluation for anti-hypertensive agents. Further investigations should be necessary for appropriate adoption of drug including elucidation of predictors for responder as indicated at ARB [43].

Acknowledgments

We are grateful to the study investigators of the Genomic Disease Outcome Consortium (G-DOC), which was organized as the Kanazawa Renal Disease Study Group in 1995 for presenting a part of the subjects of this study. We are also grateful to Miss Youko Hayashida for secretarial assistance. This work was supported by grants-in-aid 08770879, 09770843, 14571020 and 22590909 from the Ministry of Education, Science and Culture of Japan.

The authors of this manuscript have certified that they comply with the principles of ethical publishing in the International Journal of Cardiology.

References

[1] K/DOQI. clinical practice guidelines for chronic kidney disease: evaluation, classification, and stratification. Am J Kidney Dis 2002;39:S1–S266.
[2] Sarnak MJ, Levey AS, Schoolwerth AC, et al. Kidney disease as a risk factor for development of cardiovascular disease: a statement from the American Heart Association councils on kidney in cardiovascular disease, high blood pressure research, clinical cardiology, and epidemiology and prevention. Circulation 2003;108:2154–69.
[3] Kalaitzidis R, Bakris G. Pathogenesis and treatment of microalbuminuria in patients with diabetes: the road ahead. J Clin Hypertens (Greenwich) 2009;11: 636–43.
[4] Ruggenenti P, Perna A, Remuzzi G. Retarding progression of chronic renal disease: the neglected issue of residual proteinuria. Kidney Int 2003;63:2254–61.
[5] de Zeeuw D, Remuzzi G, Parving HH, et al. Albuminuria, a therapeutic target for cardiovascular protection in type 2 diabetic patients with nephropathy. Circulation 2004;110:921–7.
[6] Effects of ramipril on cardiovascular and microvascular outcomes in people with diabetes mellitus: results of the HOPE study and MICRO-HOPE substudy. Heart Outcomes Prevention Evaluation Study Investigators. Lancet 2000;355:253–9.
[7] Brenner BM, Cooper ME, de Zeeuw D, et al. Effects of losartan on renal and cardiovascular outcomes in patients with type 2 diabetes and nephropathy. N Engl J Med 2001;345:861–9.
[8] Bakris GL, Weir MR, DeQuattro V, McMahon FG. Effects of an ACE inhibitor/calcium antagonist combination on proteinuria in diabetic nephropathy. Kidney Int 1998;54:1283–9.
[9] Corvol P, Soubrier F, Jeunemaitre X. Molecular genetics of the renin–angiotensin–aldosterone system in human hypertension. Pathol Biol (Paris) 1997;45:229–39.
[10] Dzau V. The cardiovascular continuum and renin–angiotensin–aldosterone system blockade. J Hypertens Suppl 2005;23:S9–S17.
[11] Wakahara S, Konoshita T, Mizuno S, et al. Synergistic expression of angiotensin-converting enzyme (ACE) and ACE2 in human renal tissue and confounding effects of hypertension on the ACE to ACE2 ratio. Endocrinology 2007;148:2453–7.
[12] Konoshita T, Wakahara S, Mizuno S, et al. Tissue gene expression of renin–angiotensin system in human type 2 diabetic nephropathy. Diabetes Care 2006;29:848–52.
[13] Konoshita T, Germain S, Philippe J, Corvol P, Pinet F. Evidence that renal and chorionic tissues contain similar nuclear binding proteins that recognize the human renin promoter. Kidney Int 1996;50:1515–24.
[14] Germain S, Konoshita T, Philippe J, Corvol P, Pinet F. Transcriptional induction of the human renin gene by cyclic AMP requires cyclic AMP response element-binding protein (CREB) and a factor binding a pituitary-specific trans-acting factor (Pit-1) motif. Biochem J 1996;316:107–13.
[15] Fuchs S, Philippe J, Corvol P, Pinet F. Implication of Ref-1 in the repression of renin gene transcription by intracellular calcium. J Hypertens 2003;21:327–35.
[16] Konoshita T, Makino Y, Wakahara S, et al. Candidate cis-elements for human renin gene expression in the promoter region. J Cell Biochem 2004;93:327–36.
[17] Konoshita T, Fuchs S, Makino Y, Wakahara S, Miyamori I. A proximal direct repeat motif characterized as a negative regulatory element in the human renin gene. J Cell Biochem 2007;102:1043–50.
[18] Catterall WA. Structure and regulation of voltage-gated Ca^{2+} channels. Annu Rev Cell Dev Biol 2000;16:521–55.
[19] Ertel EA, Campbell KP, Harpold MM, et al. Nomenclature of voltage-gated calcium channels. Neuron 2000;25:533–5.
[20] Takahara A. Cilnidipine: a new generation ca channel blocker with inhibitory action on sympathetic neurotransmitter release. Cardiovasc Ther 2009;27: 124–39.
[21] KDOQI. Clinical practice guidelines and clinical practice recommendations for diabetes and chronic kidney disease. Am J Kidney Dis 2007;49:S12–S154.
[22] Konno Y, Kimura K. Vasodilatory effect of cilnidipine, an L-type and N-type calcium channel blocker, on rat kidney glomerular arterioles. Int Heart J 2008;49:723–32.
[23] Hayashi K, Wakino S, Sugano N, Ozawa Y, Homma K, Saruta T. Ca^{2+} channel subtypes and pharmacology in the kidney. Circ Res 2007;100:342–53.
[24] Hasebe N, Kikuchi K. Controlled-release nifedipine and candesartan low-dose combination therapy in patients with essential hypertension: the NICE Combi (Nifedipine and Candesartan Combination) Study. J Hypertens 2005;23:445–53.
[25] Katayama K, Nomura S, Ishikawa H, Murata T, Koyabu S, Nakano T. Comparison between valsartan and valsartan plus cilnidipine in type II diabetics with normo- and microalbuminuria. Kidney Int 2006;70:151–6.
[26] Fujisawa T, Ikegami H, Noso S, et al. Renoprotective effect of N-type Ca channel blockade in diabetic nephropathy. J Diabetes Complications 2007;21:252–7.
[27] Fujita T, Ando K, Nishimura H, et al. Antiproteinuric effect of the calcium channel blocker cilnidipine added to renin–angiotensin inhibition in hypertensive patients with chronic renal disease. Kidney Int 2007;72:1543–9.
[28] Morimoto S, Yano Y, Maki K, Iwasaka T. Renal and vascular protective effects of cilnidipine in patients with essential hypertension. J Hypertens 2007;25:2178–83.
[29] Kario K. Morning surge in blood pressure and cardiovascular risk: evidence and perspectives. Hypertension 2010;56:765–73.
[30] Konda T, Enomoto A, Matsushita J, Takahara A, Moriyama T. The N- and L-type calcium channel blocker cilnidipine suppresses renal injury in dahl rats fed a high-sucrose diet, an experimental model of metabolic syndrome. Nephron Physiol 2005;101:1–13.
[31] Konda T, Enomoto A, Aritomi S, et al. Different effects of L/N-type and L-type calcium channel blockers on the renin–angiotensin–aldosterone system in SHR/Izm. Am J Nephrol 2009;30:155–61.
[32] Konoshita T, Makino Y, Kimura T, et al. A new-generation N/L-type calcium channel blocker leads to less activation of the renin–angiotensin system compared with conventional L type calcium channel blocker. J Hypertens 2010;28:2156–60.
[33] Hirning LD, Fox AP, McCleskey EW, et al. Dominant role of N-type Ca^{2+} channels in evoked release of norepinephrine from sympathetic neurons. Science 1988;239:57–61.
[34] Uneyama H, Takahara A, Dohmoto H, Yoshimoto R, Inoue K, Akaike N. Blockade of N-type Ca^{2+} current by cilnidipine (FRC-8653) in acutely dissociated rat sympathetic neurones. Br J Pharmacol 1997;122:37–42.
[35] Takahara A, Dohmoto H, Hisa H, Satoh S, Yoshimoto R. Cilnidipine attenuates renal nerve stimulation-induced renal vasoconstriction and antinatriuresis in anesthetized dogs. Jpn J Pharmacol 1997;75:27–32.
[36] Fan YY, Kohno M, Nakano D, et al. Cilnidipine suppresses podocyte injury and proteinuria in metabolic syndrome rats: possible involvement of N-type calcium channel in podocyte. J Hypertens 2010;28:1034–43.

[37] Somekawa S, Imagawa K, Naya N, et al. Regulation of aldosterone and cortisol production by the transcriptional repressor neuron restrictive silencer factor. Endocrinology 2009;150:3110–7.
[38] Dietz JD, Du S, Bolten CW, et al. A number of marketed dihydropyridine calcium channel blockers have mineralocorticoid receptor antagonist activity. Hypertension 2008;51:742–8.
[39] Sugiura T, Imai E, Moriyama T, Horio M, Hori M. Calcium channel blockers inhibit proliferation and matrix production in rat mesangial cells: possible mechanism of suppression of AP-1 and CREB activities. Nephron 2000;85:71–80.
[40] Matsumori A, Nunokawa Y, Sasayama S. Nifedipine inhibits activation of transcription factor NF-kappaB. Life Sci 2000;67:2655–61.
[41] Haller H, Ito S, Izzo Jr JL, et al. Olmesartan for the delay or prevention of microalbuminuria in type 2 diabetes. N Engl J Med 2011;364:907–17.
[42] Griffin KA, Picken M, Bakris GL, Bidani AK. Comparative effects of selective T- and L-type calcium channel blockers in the remnant kidney model. Hypertension 2001;37:1268–72.
[43] Konoshita T, Kato N, Fuchs S, et al. Genetic variant of the renin–angiotensin system and diabetes influences blood pressure response to angiotensin receptor blockers. Diabetes Care 2009;32:1485–90.
