# Supplementary material for: Effects of T-Type Calcium Channel Blockers on Renal Function and Aldosterone in Patients with Hypertension: A Systematic Review and Meta-Analysis
Source: PLoS One. 2014 Oct 17;9(10):e109834. doi: 10.1371/journal.pone.0109834 (PMC4201480; doi:10.1371/journal.pone.0109834)
Supplement: File S3 — PDF files of twenty-four studies included in the meta-analysis. (ZIP) [file pone.0109834.s007.zip › Supporting information-PDF files/13. Am J Nephrol 2012[35(5)]416-423.pdf]

Original Report: Patient-Oriented, Translational Research

American Journal of Nephrology

Am J Nephrol 2012;35:416–423
DOI: 10.1159/000338132

Received: January 17, 2012
Accepted: March 14, 2012
Published online: April 20, 2012

Long-Term Effects of Calcium Antagonists on Augmentation Index in Hypertensive Patients with Chronic Kidney Disease: A Randomized Controlled Study

Tsuneo Takenaka[a] Takeru Seto[a] Mika Okayama[a] Eriko Kojima[a] Yuka Nodaira[a] Keita Sueyoshi[a] Tomohiro Kikuta[a] Yusuke Watanabe[a] Tsutomu Inoue[a] Hiroshi Takane[a] Yoichi Ohno[b] Hiromichi Suzuki[a]

[a]Department of Nephrology, and [b]Community Health Center, Saitama Medical University, Faculty of Medicine, Moroyama, Japan

Key Words

Angiotensin receptor blocker · Glomerular capillary pressure · Sympathetic nervous system · Central blood pressure · Cardiovascular disease

Abstract

Background: Our previous retrospective study showed that benidipine was superior to amlodipine (AM) for reducing proteinuria and preserving the augmentation index (AI) in patients with chronic kidney disease (CKD). ***Methods:*** The present study enrolled CKD patients whose blood pressure was not well controlled by an angiotensin receptor blocker (ARB) and a calcium channel blocker other than AM or azelnidipine (AZ). Either AM (5 mg) or AZ (16 mg) was prescribed randomly. Clinical parameters, including proteinuria, serum creatinine, and AI, were measured before initiation of AM or AZ and 1 year later to assess the long-term effect on renal function and central blood pressure. ***Results:*** Brachial and central blood pressures were similarly reduced in both groups. However, pulse rate increased in the AM group, but decreased in the AZ group (+3 ± 1 vs. –2 ± 1 bpm, $p < 0.0001$). The reduction of proteinuria was greater in the AZ group (–29 ± 2 vs. –38 ± 3%, $p < 0.01$). Improvement of AI adjusted for a pulse rate of 75 bpm was larger in the AZ group than in the AM group (–4 ± 1 vs. –9 ± 1%, $p < 0.05$). In both groups, estimated GFR remained unchanged throughout the observation period. ***Conclusion:*** In hypertensive patients with CKD, combined treatment with AZ and an ARB decreases proteinuria and preferentially improves arterial reflection.

Copyright © 2012 S. Karger AG, Basel

Introduction

Patients with chronic kidney disease (CKD) have an increased risk of developing cardiovascular (CV) diseases. Therefore, the aim of treatment for CKD should not only delay the progression of renal dysfunction, but also prevent the development of CV disease. A recent large-scale clinical trial demonstrated that combined treatment with amlodipine (AM) plus perindopril is superior

Parts of the data in the manuscript were presented at the annual scientific meeting of Japanese Society of Hypertension, November 2010, Fukuoka, Japan.

KARGER

Fax +41 61 306 12 34
E-Mail karger@karger.ch
www.karger.com

© 2012 S. Karger AG, Basel
0250–8095/12/0355–0416$38.00/0

Accessible online at:
www.karger.com/ajn

Tsuneo Takenaka MD, PhD, Associate Professor
Department of Nephrology, Saitama Medical University, Faculty of Medicine
38 Moro-hongo Moroyama
Iruma, Saitama 350-0495 (Japan)
Tel. +81 49 276 1611, E-Mail takenaka@saitama-med.ac.jp

Downloaded by:
Third Military Medical Univ.
113.204.149.2 - 12/9/2013 1:07:05 PM

to atenolol plus thiazide for reducing the rates of fatal and nonfatal stroke, CV events and procedures, and all-cause mortality in hypertensive patients [1]. In addition, the former regimen more markedly reduced both central blood pressure and the augmentation index (AI) compared with the latter regimen, even though brachial blood pressure was similar in both groups [2]. Furthermore, after heart rate adjustment, AI in the former group was lower than the latter by approximately 2%, suggesting that aortic stiffness and vascular remodeling, which could be differentially influenced by drugs, may account for residual variability [3]. Bakris et al. [4] reported that combined treatment with AM plus an angiotensin-converting enzyme inhibitor was superior to an angiotensin-converting enzyme inhibitor plus thiazide for preserving renal function in hypertensive CKD patients.

At present, numerous calcium channel blockers (CCB) are available on the market. The joint committee of the Japanese Society of Hypertension and the Japanese Society of Nephrology has recommended using CCBs to dilate the efferent arterioles in hypertensive CKD patients with a high CV risk if their blood pressure cannot be controlled within the target range of 130/80 mm Hg by either an angiotensin receptor blocker (ARB) or angiotensin-converting enzyme inhibitor alone [5]. Fujita et al. [6] reported that cilnidipine, which blocks L/N-type channels and dilates the efferent arterioles, shows a superior renoprotective effect compared with AM. However, the CARTER study did not assess the effect of cilnidipine, which only has a weak inhibitory effect on T-type calcium channels, on AI [6–8]. We previously performed a retrospective study in hypertensive CKD patients, and found that benidipine (which blocks T-type calcium channels) combined with an angiotensin inhibitor was more beneficial for both proteinuria and AI than AM when similar blood pressure control was attained [9]. These findings suggest that antiproteinuric CCBs may not only protect renal function, but also improve arterial stiffness in CKD patients. However, a prospective randomized clinical trial would be required to corroborate this hypothesis.

Azelnidipine (AZ) is a potent new antihypertensive agent that shows an antiproteinuric effect [10]. AZ is a dihydropyridine CCB that blocks both L-type and T-type channels, uniquely reducing the pulse rate [11]. Antihypertensive agents show differing effects on the central blood pressure and AI [12]. Matsui et al. [13] reported that AZ reduced heart rate-adjusted AI more than diuretics when administered concomitantly with an ARB to hypertensive patients. However, they did not compare the influence of different CCBs on the AI in hypertensive patients. To our knowledge, the effects of AZ on AI in CKD patients have not been well characterized. Accordingly, the present study was performed to assess the long-term effect of AZ on both AI and renal function in CKD patients with hypertension.

Methods

This was an investigator-initiated, open-label, parallel group, randomized, controlled study, which examined the effect on arterial reflection of switching other CCBs to AM (5 mg) or AZ (16 mg) for 1 year. From April 2008 to March 2009, 67 hypertensive patients with CKD were enrolled (fig. 1). The sample size was determined based on our previous observation of longitudinal changes in AI caused by CCBs [9]. Subjects who attended the outpatient department of our nephrology center once a month for CKD management, were taking an ARB and a CCB other than AM or AZ, and were considered to have poor blood pressure control (>130/80 mm Hg on at least 2 visits) were eligible for the study if their estimated GFR (eGFR) was >10 ml/min/1,73 m^2 and informed consent was provided. Five patients were excluded from the study, including 3 patients with diabetes, 1 patient who refused consent, and 1 patient with poor drug compliance. Randomization was performed by the envelope method. At study entry, some patients were taking other antihypertensives including β-blockers, α-blockers, diuretics, and drugs affecting the central nervous system. We did not exclude such patients, but the dosage of other antihypertensive medications was not changed during the AM or AZ treatment period [9]. Baseline demographic data are displayed in table 1. Before AM or AZ treatment, 44 patients were on slow-acting nifedipine (20–40 mg daily, mean dose: 30 mg), 6 patients were taking nisoldipine (5–10 mg daily, mean dose: 7.5 mg), 3 patients were using valnidipine (10 mg daily), 4 patients were on nitrendipine (5–10 mg daily, mean dose: 7.5 mg), and 2 patients were taking manidipine (10–20 mg daily, mean dose: 15 mg). Patients with dyslipidemia stayed on the same doses of lipid-lowering medications throughout the study [14].

All of the subjects switched their once-daily CCBs to AM or AZ. No washout period was set due to ethical considerations. The office systolic blood pressure (SBP) and diastolic blood pressure (DBP) were measured after the patient had rested for at least 5 min between 9 and 11 a.m. using a mercury sphygmomanometer (the first and fifth Korotkoff sounds were used to identify the SBP and DBP, respectively). Two measurements were done after the patient had been in the sitting position for 5 and 10 min, respectively, and the average of the two values was defined as the blood pressure for efficacy analysis. The target blood pressure for this study was ≤130/80 mm Hg. ARBs were taken once in the morning. If blood pressure was not controlled, the ARB dose would have been titrated, but this was not required. Patients were requested to continue their usual diet and daily activities during the study period. We analyzed data at 1 and 12 months because earlier studies have indicated that a 12-month treatment period is sufficient to observe the long-term effects of CCBs on blood pressure and arterial stiffness [9, 13], and because a longer study period might have obscured the effects of AM or AZ by changing other factors such as diet and daily activities [14].

Downloaded by:
Third Military Medical Univ.
113.204.149.2 - 12/9/2013 1:07:05 PM

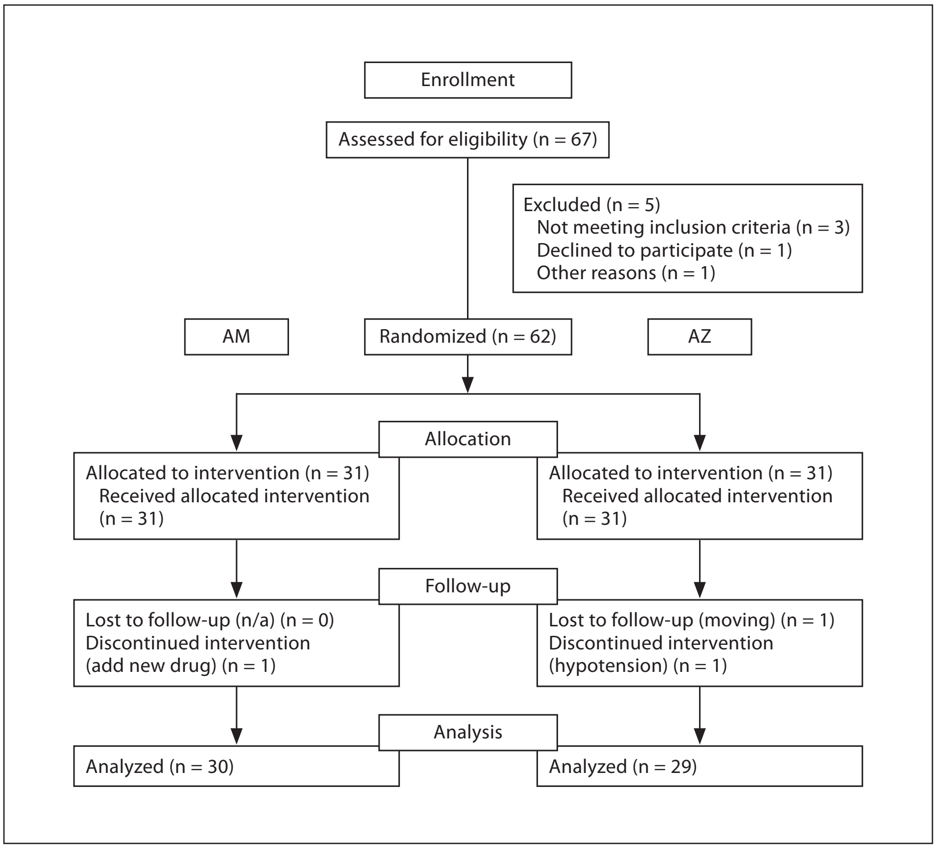

Fig. 1. Flow diagram of the study. Left and right columns show the AM and AZ groups, respectively.

At the time of study entry, as well as 1 and 12 months after starting AM or AZ treatment, the central and brachial blood pressures, urinary protein, serum creatinine, and AI were measured while fasting. The eGFR was calculated by using the MDRD equation modified for Japan [5]. Radial artery pressure pulse waveforms were recorded with an automated tonometric system (HEM-9000AI; Omron Healthcare, Kyoto, Japan) while the patient was in the sitting position [15, 16]. The waveform was calibrated automatically using a built-in oscillometric brachial sphygmomanometer. The peak and trough of the radial pressure wave were adjusted to brachial SBP and DBP, respectively. The HEM-9000AI algorithm automatically performed online detection of the second peak (late systolic inflection) based on the second maximum of the fourth derivative of the radial pressure waveform to determine the late or second SBP (SBP2) as an index of central blood pressure. SBP2 is well correlated with the aortic SBP measured simultaneously by direct catheterization [17], validating wave reflection measurement with this device. Then the AI was calculated as SBP2-DBP divided by SBP-DBP. Thus, AI is not an absolute pressure, but a ratio of reflection pressure against ejection pressure at radial artery. Two measurements were obtained 1 min apart and averaged for analysis. A single observer performed all waveform measurements to avoid interobserver errors. Brain natriuretic peptide (BNP) was measured because central blood pressure is the important stress acting on the left ventricle.

Exclusion criteria were as follows: a history of recent (<6 months) myocardial infarction, unstable angina, heart failure, and cerebrovascular events; severe hepatic disease; renal replacement therapy (or predicted to start renal replacement within a year); and a history of malignancy. Patients with diabetes (a positive history, HbA_{1c} >6.5% or taking hypoglycemic medications including insulin) were also excluded [14, 16, 18]. The criteria for discontinuing the study were uncontrolled hypertension (>170/110 mm Hg), persistent hypotension (SBP <110 mm Hg), doubling of serum creatinine and the requirement for renal replacement therapy. The study was conducted according to the principles of Good Clinical Practice and the Declaration of Helsinki. The study protocol was approved by the ethics committee of Saitama Medical University and was properly registered (UMIN-ID: 000003323).

Results are expressed as the mean ± SEM. Data were subject to analysis of variance for repeated measures, followed by Tukey's HSD test for all comparisons as formal analyses. The χ^2 test with Yates' correction was used to compare discrete variables between groups. To perform an exploratory analysis, the value obtained at the end of the study was converted to percent change or difference from the respective baseline value, and Student's t test was employed for comparisons between groups. Multivariate regression was used to assess variability of clinical parameters. Statistical significance was defined as $p < 0.05$. All analyses were performed with SPSS software (version 17; SPSS Inc., Chicago, Ill., USA).

Downloaded by:
Third Military Medical Univ.
113.204.149.2 - 12/9/2013 1:07:05 PM

Table 1. Patient background

	AM group	AZ group
Number of patients	30	29
Age, years	67 ± 2	66 ± 2
Male, %	60	63
ARB prescribed, n (dose; average dose)		
Olmesartan	18 (20; 20)	19 (20; 20)
Losartan	4 (100; 100)	3 (100; 100)
Telmisartan	4 (20–40; 30)	3 (20–40; 33)
Candesartan	2 (8; 8)	1 (8; 8)
Valsartan	2 (80–160; 120)	3 (80–160; 93)
Underlying renal diseases		
Nephrosclerosis	6	8
IgAN	13	10
Other GN	5	6
PCK	1	2
Unknown	5	3

Dosage of ARB for a day is depicted in mg. IgAN = IgA nephropathy; GN = glomerulonephritis; PCK = polycystic kidney disease.

Table 2. Relationship of AI with clinical parameters

	Slope	t	p
Age	0.47	5.0	<0.0001
Sex (male = 1, female = 0)	–3.02	–2.0	0.04
eGFR	–1.89	–0.63	0.56
SBP	0.02	0.14	0.88
DBP	0.06	0.45	0.65
PR	–0.28	–2.5	0.02
UP	0.04	1.15	0.15
BNP	0.02	2.3	0.03

F = 23, d.f. (8, 50), p < 0.0001. UP = Urinary protein; PR = pulse rate.

Results

Thirty-one patients were initially allocated to each group (fig. 1). During follow-up, 2 patients dropped out of the AZ group due to moving and hypotension, respectively. One patient dropped out of the AM group because a new antihypertensive agent was added to control the blood pressure. In total, 59 patients completed the study and were analyzed. As shown in table 1, patient backgrounds were similar between the two groups. During the study, 1 patient from the AM group complained of palpitations, and 1 patient from the AZ group had ankle edema. However, their symptoms resolved without specific treatment.

At baseline, the variability of clinical parameters was assessed (table 2). AI tended to be higher in females, and correlated positively to both age and BNP, and inversely to pulse rate.

At study entry, SBP and DBP did not differ between the AM and AZ groups (table 3). After switching to AM or AZ, SBP decreased in both groups. However, AZ did not decrease DBP significantly at 1 month. After 1 year, both SBP and DBP became identical in AM and AZ groups. At study entry, pulse rates were similar between the AM and AZ groups (fig. 2). After 1 month, the pulse rate was increased by AM and decreased by AZ, and the same trend persisted during observation for 12 months. Thus, an exploratory analysis showed that annual changes in pulse rate differed significantly different between the two groups (+3 ± 1 vs. –2 ± 1 bpm, $p < 0.0001$). As shown in table 2, AM decreased SBP2 after 1 month and was still reduced at 1 year. SBP2 was not decreased by AZ at 1 month, but it was reduced at 1 year. No significant differences in SBP2 were observed between the two groups.

Protein excretion was similar in the AM and AZ groups at study entry (fig. 3). Treatment with both AM and AZ gradually reduced protein excretion and a significant decrease of proteinuria was observed after only 1 month in both groups. Exploratory analysis revealed that the annual reduction of urinary protein excretion was larger in the AZ group than the AM group (–29 ± 2 vs. –38 ± 3%, $p < 0.01$). After 1 year, eGFR was unchanged in both groups and it was similar between the two groups throughout the study, even though AM and AZ tended to cause an increase or decrease in eGFR at 1 year (1 ± 2 vs. –2 ± 4%). To assess whether the subjects had stable CKD, the eGFR at 6 months before the study was examined. The results were 26 ± 3 versus 27 ± 3 ml/min/1.73 m^2 in the AM and AZ groups, respectively, suggesting stable disease.

At study entry, AI did not differ between the AM and AZ groups (table 3). After starting AM or AZ, AI decreased gradually in both groups. While AM reduced AI at 1 month, AZ did not. At 1 year, however, AI was significantly reduced in both groups. Because AM and AZ had differing effects on pulse rate, which is an important determinant of AI (table 2), and because the other significant confounders such as age, sex and BNP were similar between groups, this parameter was adjusted for a pulse rate of 75 bpm (AI75). As a result, AI75 did not differ between the AM and AZ groups at the time of study

Downloaded by:
Third Military Medical Univ.
113.204.149.2 - 12/9/2013 1:07:05 PM

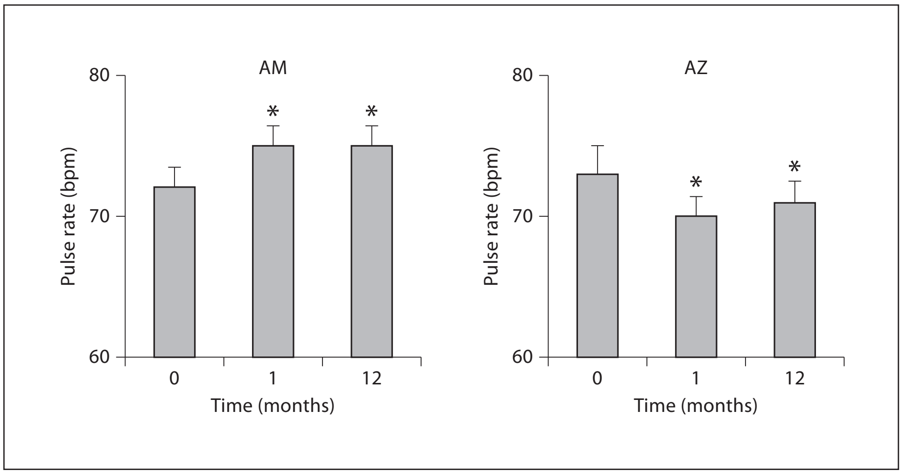

Fig. 2. Influence of exchanging calcium antagonist to AM or AZ on pulse rate. * Indicates significance from respective basal value.

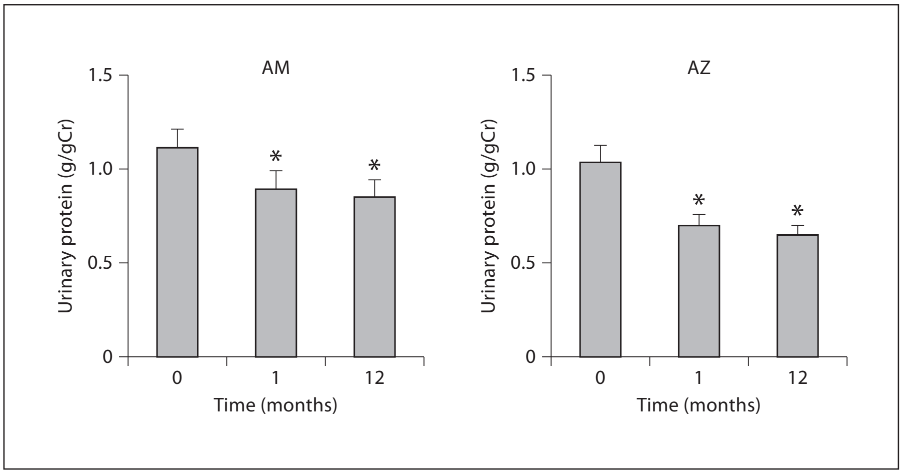

Fig. 3. Effects of switching calcium antagonist to AM or AZ on protein excretion. * Depicts significance from respective basal value.

Table 3. Temporal profiles of clinical parameters

	AM group			AZ group		
Time, month	0	1	12	0	1	12
SBP, mm Hg	140 ± 2	133 ± 2*	131 ± 2*	139 ± 2	135 ± 2*	132 ± 2*
DBP , mm Hg	86 ± 2	79 ± 1*	77 ± 1*	83 ± 2	80 ± 2	78 ± 1*
SBP2, mm Hg	125 ± 2	116 ± 2*	114 ± 2*	124 ± 2	119 ± 2	114 ± 2*
BNP, pg/ml	176 ± 12	168 ± 14	165 ± 13	173 ± 15	168 ± 16	146 ± 14
eGFR, ml/min/1.73 m^2	25 ± 3	26 ± 3	26 ± 3	26 ± 3	24 ± 2	24 ± 2
AI	74 ± 2	69 ± 2*	69 ± 2*	73 ± 2	71 ± 2	68 ± 2*

* Significant difference from the respective basal value (time 0).

entry (fig. 4). Administration of AM or AZ gradually reduced AI75 in each group. In contrast to AI, there was a significant decrease in AI75 at 1 month in the AZ group. Exploratory analysis revealed that the decrement of AI75 in the AZ group was greater than that in the AM group at 12 months (–4 ± 1 vs. –9 ± 1%, $p < 0.05$). In the case of BNP, ANOVA did not reveal any differences between the two groups or within each group.

Downloaded by:
Third Military Medical Univ.
113.204.149.2 - 12/9/2013 1:07:05 PM

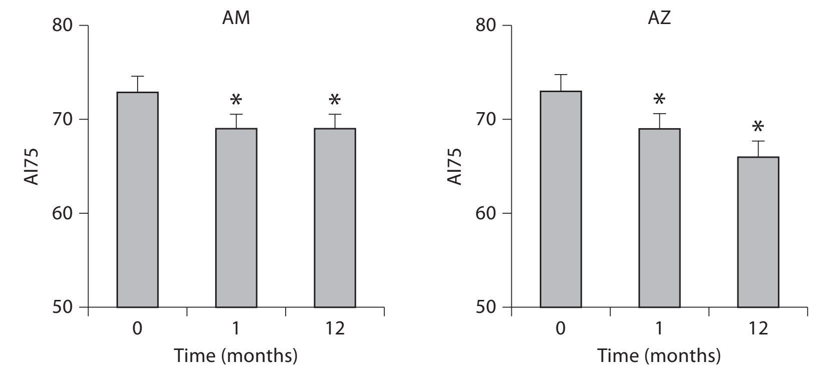

Fig. 4. Impact of exchanging calcium antagonist to AM or AZ on AI75. * Describes significance from respective basal value.

Discussion

Hypertension exacerbates renal dysfunction and antihypertensive treatment is a mainstay of therapy for CKD [1]. In the present study, administration of AM or AZ significantly decreased blood pressure. Importantly, AZ elicited a progressive reduction of blood pressure in hypertensive CKD patients over 1 month, although its effect on DBP was slower than that of AM. Our results indicate that AZ is as efficient as AM over the long term for reducing blood pressure in CKD patients, and suggest that more than 1 month should be allowed to see the maximal antihypertensive effect of AZ before adding any other antihypertensive drugs to avoid hypotension.

Our present findings are compatible with a previous report [19], and indicate that AM slightly increases pulse rate. Surprisingly, AZ decreased both blood pressure and pulse rate in the present study. AZ inhibits sympathetic outflow by a direct hyperpolarizing effect on rostral ventrolateral medullary neurons and by an indirect action on medulla via induction of nitric oxide synthesis [19, 20]. Hermsmeyer [21] reported direct inhibitory effects of T channel blocker on cardiac automaticity. The present finding that central blood pressure (SBP2) was similar in two groups suggest that AZ reduces double product (pulse rate × central blood pressure) to a larger extent than AM, and accounts for the fact that BNP tended to be lower in the AZ group after 1 year. Lin et al. [22] suggested that in type C waveforms, SBP2 may incorrectly estimate central blood pressure, especially at low SBP. Average SBP was over 130 mm Hg in the present study. There is a debate regarding the influence of pulse rate on CV disease. Sympathetic activation and a high pulse rate should worsen CV prognosis [19, 20], but decreasing pulse rate by itself could elevate central blood pressure and worsen CV prognosis [23]. Collectively, these data suggest that AZ may improve CV prognosis by decreasing the pulse rate without causing an increase in central blood pressure.

Benidipine and efonidipine dilate both afferent and efferent arterioles, thus conferring renal protection [8]. Efferent arterioles possess T-type, but not L-type voltage-dependent calcium channels (VDCC). Blockade of T-type VDCC attenuates glomerular hypertension and proteinuria in CKD patients via efferent arteriolar dilation. Our present data indicate that AZ, which inhibits T-type and L-type VDCCs, significantly decreases proteinuria after 1 month without changing SBP2, and suggest that AZ promptly reduces glomerular pressure without changing renal perfusion pressure. However, AZ may weakly inhibit T channels in clinical settings. The possibility remains that AZ induces efferent arteriolar dilation via the inhibition of transient receptor potential channels [24]. In addition, we identified the novel finding that AZ reduced proteinuria more than AM after 1 year. At that time, SBP2 and AI showed a similar decrease in both groups. Central rather than brachial SBP causes damage to the kidneys as well as the brain [25]. Our previous studies indicated that a higher AI is related to more proteinuria and more rapid decline of creatinine clearance [15]. Thus, the present data suggest that AZ decreases glomerular pressure by reducing efferent arteriolar tone as well as central blood pressure.

AI is a marker of reflection pressure [9, 13, 17]. Many CKD patients suffer from atherosclerotic CV disease, and atherosclerosis increases arterial stiffness and then re-

Downloaded by:
Third Military Medical Univ.
113.204.149.2 - 12/9/2013 1:07:05 PM

flection wave [1, 26]. Recent studies have indicated that AI predicts CV risk and events in CKD patients [16, 27]. In addition, Covic et al. [26] demonstrated that AI predicts the extent of coronary artery disease in CKD patients. Since antihypertensives often alter pulse rate, changes in arterial reflection should be assessed by AI75. The present data constitute new demonstrations that AZ reduces AI75 more effectively than AM, suggesting that AZ achieves considerable improvement of wave reflection independently of changes in pulse rates. CKD is characterized by increased oxidative stress, which induces arterial contraction and remodeling [28, 29]. AZ is a potent antioxidant, so it may decrease wave reflection acutely by increasing the bioavailability of vasodilatory nitric oxide and chronically by altering vascular remodeling [29, 30]. Together, these data indicate that AZ is more effective in reducing AI75 than AM in CKD patients, and suggest that antihypertensive treatment with AZ improves both renal and CV outcomes in CKD patients.

The present study has several limitations. Patients with diabetes were excluded from the study because autonomic neuropathy could increase the variability of both blood pressure and AI, possibly confounding interpretation [16]. In addition, we enrolled patients from a single center in one country, a potential bias in patient selection. Although statistical significance was attained, the sample size was relatively small, especially for comparisons between groups. Larger studies across a number of countries will be required to draw final conclusions. Thus, great caution is required when generalizing the results. However, our present data are compatible with those of Fujita et al. [6], and have further revealed that AZ preferentially reduces arterial stiffness as well as proteinuria in CKD patients.

In summary, the present study indicated that AZ is as efficient as AM for reducing blood pressure in CKD patients when administered concomitantly with an ARB. In addition, we obtained evidence that AZ reduces proteinuria without changing eGFR. Finally, our observations suggest that AZ decreases both arterial reflection and pulse rate, thus presumably improving the CV prognosis.

Acknowledgements

The authors thank Ms. Sachiko Nakazato for her excellent secretary help during preparing the manuscript.

Disclosure Statement

We did not receive any funding specific to this study; however, we have received grants from Takeda Pharmaceutical Co. Ltd., Daiichi Sankyo Co. Ltd., Kyowa Hakko Kirin Co. Ltd., Chugai Pharmaceutical Co. Ltd., Pfizer Co. Ltd., Novartis International AG, Merck & Co. Inc., Astellas Pharma Inc., Taisho-Toyama Pharmaceutical Co. Ltd., Ajinomoto Pharma Co. Ltd., Dainippon-Sumitomo Pharma Co. Ltd., and Bayer Pharmaceutical Co. Ltd.

References

1 Dahlöf B, Sever PS, Poulter NR, Wedel H, Beevers DG, Caulfield M, Collins R, Kjeldsen SE, Kristinsson A, McInnes GT, Mehlsen J, Nieminen M, O'Brien E, Ostergren J, ASCOT Investigators: Prevention of cardiovascular events with an antihypertensive regimen of amlodipine adding perindopril as required versus atenolol adding bendroflumethiazide as required, in the Anglo-Scandinavian Cardiac Outcomes Trial-Blood Pressure Lowering Arm (ASCOT-BPLA): a multicentre randomised controlled trial. Lancet 2005;366:895–906.

2 Williams B, Lacy PS, Thom SM, Cruickshank K, Stanton A, Collier D, Hughes AD, Thurston H, O'Rourke M, CAFE Investigators, Anglo-Scandinavian Cardiac Outcomes Trial Investigators, CAFE Steering Committee and Writing Committee: Differential impact of blood pressure-lowering drugs on central aortic pressure and clinical outcomes: principal results of the Conduit Artery Function Evaluation (CAFE) study. Circulation 2006;113:1213–1225.

3 Williams B, Lacy PS, CAFE and the ASCOT (Anglo-Scandinavian Cardiac Outcomes Trial) Investigators: Impact of heart rate on central aortic pressures and hemodynamics: analysis from the CAFE (Conduit Artery Function Evaluation) study: CAFE-Heart Rate. J Am Coll Cardiol 2009;54:705–713.

4 Bakris GL, Sarafidis PA, Weir MR, Dahlöf B, Pitt B, Jamerson K, Velazquez EJ, Staikos-Byrne L, Kelly RY, Shi V, Chiang YT, Weber MA, ACCOMPLISH Trial Investigators: Renal outcomes with different fixed-dose combination therapies in patients with hypertension at high risk for cardiovascular events (ACCOMPLISH): a prespecified secondary analysis of a randomised controlled trial. Lancet 2010;375:1173–1181.

5 Guidelines for the management of chronic kidney diseases; in: Hypertension. Tokyo-Igaku, Japanese Society of Hypertension and Japanese Society of Nephrology, 2008, pp 5–29. http://www.jpnsh.org/data/CKD-kouketsuatsu.pdf.

6 Fujita T, Ando K, Nishimura H, Ideura T, Yasuda G, Isshiki M, Takahashi K, Cilnidipine versus Amlodipine Randomised Trial for Evaluation in Renal Disease (CARTER) Study Investigators: Antiproteinuric effect of the calcium channel blocker cilnidipine added to renin-angiotensin inhibition in hypertensive patients with chronic renal disease. Kidney Int 2007;72:1543–1549.

7 Zhou X, Ono H, Ono Y, Frohlich ED: N- and L-type calcium channel antagonist improves glomerular dynamics, reverses severe nephrosclerosis, and inhibits apoptosis and proliferation in an l-NAME/SHR model. J Hypertens 2002;20:993–1000.

8 Hayashi K, Wakino S, Sugano N, Ozawa Y, Homma K, Saruta T: Ca2+ channel subtypes and pharmacology in the kidney. Circ Res 2007;100:342–353.

Downloaded by:
Third Military Medical Univ.
113.204.149.2 - 12/9/2013 1:07:05 PM

9 Takenaka T, Takane H, Okada H, Ohno Y, Suzuki H: Long-term effects of calcium antagonists on augmentation index in hypertensive patients with chronic kidney diseases. NDT Plus 2009;2:192–193.

10 Sada T, Saito H: Pharmacological profiles and clinical effects of azelnidipine, a long-acting calcium channel blocker. Folia Pharmacol Jpn 2003;122:539–547.

11 Furukawa T, Nukada T, Namiki Y, Miyashita Y, Hatsuno K, Ueno Y, Yamakawa T, Isshiki T: Five different profiles of dihydropyridines in blocking T-type Ca(2+) channel subtypes (Ca(v)3.1 (alpha(1G)), Ca(v)3.2 (alpha(1H)), and Ca(v)3.3 (alpha(1I))) expressed in Xenopus oocytes. Eur J Pharmacol 2009;613:100–107.

12 Miyashita H, Aizawa A, Hashimoto J, Hirooka Y, Imai Y, Kawano Y, Kohara K, Sunagawa K, Suzuki H, Tabara Y, Takazawa K, Takenaka T, Yasuda H, Shimada K: Cross-sectional characterization of all classes of antihypertensives in terms of central blood pressure in Japanese hypertensive patients. Am J Hypertens 2010;23:260–268.

13 Matsui Y, Eguchi K, O'Rourke MF, Ishikawa J, Miyashita H, Shimada K, Kario K: Differential effects between a calcium channel blocker and a diuretic when used in combination with angiotensin II receptor blocker on central aortic pressure in hypertensive patients. Hypertension 2009;54:716–723.

14 Kobayashi K, Ohno Y, Takenaka T, Ikeda N, Okada H, Kanno Y, Suzuki H: Telmisartan lowers home blood pressure and improves insulin resistance without correlation between their changes. Clin Exp Hypertens 2011;33:100–105.

15 Takenaka T, Mimura T, Kanno Y, Suzuki H: Qualification of arterial stiffness as a risk factor to the progression of chronic kidney diseases. Am J Nephrol 2005;25:417–424.

16 Takenaka T, Sato T, Hoshi H, Kato N, Sueyoshi K, Tsuda M, Watanabe Y, Takane H, Ohno Y, Suzuki H: Height constitutes an important predictor of mortality in end-stage renal disease. Cardiol Res Pract 2010;2011: 242353.

17 Takazawa K, Kobayashi H, Shindo N, Tanaka N, Yamashina A: Relationship between radial and central arterial pulse wave and evaluation of central aortic pressure using the radial arterial pulse wave. Hypertens Res 2007;30:219–228.

18 Kanno Y, Takenaka T, Nakamura T, Suzuki H: Add-on angiotensin receptor blocker in patients who have proteinuric chronic kidney diseases and are treated with angiotensin-converting enzyme inhibitors. Clin J Am Soc Nephrol 2006;1:730–737.

19 Kumagai H, Onami T, Iigaya K, Takimoto C, Hayashi K, Saruta T: Mechanisms for reduced heart rate by azelnidipine: analysis on central and peripheral sympathetic nerves. Prog Med 2004;24:2659–2664.

20 Konno S, Hirooka Y, Araki S, Koga Y, Kishi T, Sunagawa K: Azelnidipine decreases sympathetic nerve activity via antioxidant effect in the rostral ventrolateral medulla of stroke-prone spontaneously hypertensive rats. J Cardiovasc Pharmacol 2008;52:555–560.

21 Hermsmeyer K: Role of T channels in cardiovascular function. Cardiology 1998;89(suppl 1):2–9.

22 Lin MM, Cheng HM, Sung SH, Liao CF, Chen YH, Huang PH, Chen CH: Estimation of central aortic systolic pressure from the second systolic peak of the peripheral upper limb pulse depends on central aortic pressure waveform morphology. J Hypertens 2012;30:581–586.

23 Asmar RG, London GM, O'Rourke ME, Safar ME, REASON Project Coordinators and Investigators: Improvement in blood pressure, arterial stiffness and wave reflections with a very-low-dose perindopril/indapamide combination in hypertensive patient: a comparison with atenolol. Hypertension 2001;38:922–926.

24 Takenaka T, Suzuki H, Okada H, Inoue T, Kanno Y, Ozawa Y, Hayashi K, Saruta T: Transient receptor potential channels in rat renal microcirculation: actions of angiotensin II. Kidney Int 2002;62:558–565.

25 O'Rourke MF, Safar ME: Relationship between aortic stiffening and microvascular disease in brain and kidney: cause and logic of therapy. Hypertension 2005;46:200–204.

26 Covic A, Haydar AA, Bhamra-Ariza P, Gusbeth-Tatomir P, Goldsmith DJ: Aortic pulse wave velocity and arterial wave reflections predict the extent and severity of coronary artery disease in chronic kidney disease patients. J Nephrol 2005;18:388–396.

27 Nishiura R, Kita T, Yamada K, Komatsu H, Hara S, Sato Y, Fujimoto S: Radial augmentation index is related to cardiovascular risk in hemodialysis patients. Ther Apher Dial 2008;12:157–163.

28 Mimura T, Takenaka T, Kanno Y, Moriwaki K, Okada H, Suzuki H: Vascular compliance is secured under angiotensin inhibition in non-diabetic chronic kidney diseases. J Hum Hypertens 2008;22:38–47.

29 Fan YY, Kohno M, Nakano D, Hitomi H, Nagai Y, Fujisawa Y, Lu XM, Fu H, Du J, Ohmori K, Hosomi N, Kimura S, Kiyomoto H, Nishiyama A: Inhibitory effects of a dihydropyridine calcium channel blocker on renal injury in aldosterone-infused rats. J Hypertens 2009;27:1855–1862.

30 Takazawa K, Tanaka N, Takeda K, Kurosu F, Ibukiyama C: Underestimation of vasodilator effects of nitroglycerin by upper limb blood pressure. Hypertension 1995;26:520–523.

Downloaded by:
Third Military Medical Univ.
113.204.149.2 - 12/9/2013 1:07:05 PM
