# Supplementary material for: Effects of T-Type Calcium Channel Blockers on Renal Function and Aldosterone in Patients with Hypertension: A Systematic Review and Meta-Analysis
Source: PLoS One. 2014 Oct 17;9(10):e109834. doi: 10.1371/journal.pone.0109834 (PMC4201480; doi:10.1371/journal.pone.0109834)
Supplement: File S3 — PDF files of twenty-four studies included in the meta-analysis. (ZIP) [file pone.0109834.s007.zip › Supporting information-PDF files/14. Clin Cardiol 2011[34(6)]372-377.pdf]

Clinical Investigations

Calcium Channel Blocker Inhibition of AGE and RAGE Axis Limits Renal Injury in Nondiabetic Patients With Stage I or II Chronic Kidney Disease

Address for correspondence:
Sho-ichi Yamagishi, MD
Department of Pathophysiology and Therapeutics of Diabetic Vascular Complications
Kurume University School of Medicine
67 Asahi-machi
Kurume 830-0011, Japan
shoichi@med.kurume-u.ac.jp

Tsukasa Nakamura, MD, PhD; Eiichi Sato, MD; Nobuharu Fujiwara, MD; Yasuhiro Kawagoe, MD, PhD; Hikaru Koide, MD, PhD; Yoshihiko Ueda, MD, PhD; Masayoshi Takeuchi, PhD; Sho-ichi Yamagishi, MD, PhD

Division of Nephrology, Department of Internal Medicine (Nakamura, Sato, Fujiwara, Kawagoe), Shinmatsudo Central General Hospital, Matsudo, Japan; Division of Nephrology, Department of Internal Medicine (Koide), Koto Hospital, Tokyo, Japan; Department of Pathology (Ueda), Koshigaya Hospital, Dokkyo University School of Medicine, Koshigaya, Japan; Department of Pathophysiological Science (Takeuchi), Faculty of Pharmaceutical Science, Hokuriku University, Kanazawa, Japan; Department of Pathophysiology and Therapeutics of Diabetic Vascular Complications (Yamagishi), Kurume University School of Medicine, Kurume, Japan

ABSTRACT

Background: There is a growing body of evidence that advanced glycation end products (AGE) and their receptor (RAGE) system are implicated in chronic kidney disease (CKD). We have previously found that a long-acting calcium channel blocker, azelnidipine, but not amlodipine, improves renal injury in CKD patients. However, little is known about the effect of azelnidipine on the AGE-RAGE axis in humans. In this study, we examined whether azelnidipine addition could have renoprotective properties in hypertensive CKD patients by reducing serum levels of AGE and soluble form of RAGE (sRAGE). Thirty nondiabetic stage I or II CKD patients who had already been treated with angiotensin II receptor blockers were enrolled in this study.

Hypothesis: We hypothesized that azelnidipine treatment could limit renal injury partly by blocking the AGE-RAGE axis.

Methods: Patients were randomly divided into 2 groups; one group was treated with 16 mg azelnidipine and the other with 5 mg amlodipine once daily. They were followed up for 6 months.

Results: Proteinuria was positively correlated with circulating AGE and sRAGE levels in our subjects. Both drugs exhibited comparable and significant blood pressure (BP)-lowering effects. Although neither of them affected glucose, glycated hemoglobin, lipid levels, and estimated glomerular filtration rate, treatment with azelnidipine, but not amlodipine, decreased circulating AGE, sRAGE, proteinuria, and urinary levels of liver-type fatty acid binding protein, a marker of tubular injury, in a BP-lowering–independent manner.

Conclusions: Our present results suggest that azelnidipine may exert renoprotective properties in nondiabetic hypertensive CKD patients via its unique inhibitory effects on the AGE-RAGE axis.

Introduction

Reducing sugars can react nonenzymatically with the amino groups of proteins to form reversible Schiff bases, and then Amadori products.[1–3] These early glycation products undergo further complex reactions such as rearrangement, dehydration, and condensation to become irreversibly cross-linked, heterogeneous fluorescent derivatives termed advanced glycation end products (AGE).[1–3] The formation and accumulation of AGE have been reported to progress under oxidative stress conditions such as diabetes and chronic kidney disease (CKD).[4–7] Furthermore, there is a growing body of evidence that AGE and their receptor (RAGE) system are implicated in the development and progression of CKD.[6–8] Indeed, accumulation of AGE and subsequent activation of RAGE downstream signaling pathways in the kidney have been shown to contribute to the progressive alteration in renal architecture and loss of renal function in both humans and animal models.[6–8]

Recently, soluble form of RAGE (sRAGE) has been identified in human serum.[9] We, along with others, have shown that sRAGE levels and circulating AGE are correlated with each other and positively associated with the severity of target organ damage in both diabetic and nondiabetic CKD subjects.[5,9–16] Advanced glycation end products up-regulate

The authors have no funding, financial relationships, or conflicts of interest to disclose.

Published online in Wiley Online Library (wileyonlinelibrary.com)
DOI:10.1002/clc.20885 © 2011 Wiley Periodicals, Inc.
Received: August 24, 2010
Accepted with revision: December 7, 2010

RAGE expression in various tissues, and sRAGE could be mainly generated from proteolytic cleavage of membrane-bound RAGE by the actions of sheddase, a disintegrin and metalloproteinases 10 (ADAM 10).[9,17,18] These observations suggest that sRAGE could reflect tissue RAGE expression and that circulating level of AGE and sRAGE may be a biomarker for renal damage in CKD patients.

Azelnidipine is a highly lipid-soluble dihydropyridine-based calcium channel blocker (CCB) with equihypotensive properties of amolodipine.[19,20] We have previously found that azelnidipine, but not amlodipine, reduces proteinuria and decreases urinary excretion levels of liver-type fatty acid binding protein (L-FABP), a marker of tubular injury in nondiabetic CKD patients.[21] Because administration of azelnidipine was reported to reduce urinary levels of N-acetyl-beta-D-glucosaminidase activity, another marker of tubulointerstitial injury, and prevent glomerulosclerosis in exogenously AGE-injected rats,[22] it is provable that azelnidipine may exert renoprotective properties in CKD patients by inhibiting the AGE-RAGE system. However, little is known about the effect of azelnidipine on the AGE-RAGE axis in humans. Therefore, in this study, we examined whether proteinuria was positively correlated with circulating AGE and sRAGE levels in hypertensive stage I or II CKD patients who have already been treated with angiotensin II type 1 receptor blockers (ARBs). Then we investigated whether additional antihypertensive treatment with azelnidipine, but not amlodipine, a control CCB, decreased serum AGE and sRAGE levels and subsequently reduced proteinuria and urinary levels of L-FABP in a blood pressure (BP)-lowering–independent manner.

Methods

Subjects

Thirty nondiabetic stage I or II CKD patients with hypertension (18 men and 12 women, mean age 45.4 yrs) were enrolled in the present study. The definition of CKD is structural or functional kidney damage lasting for ≥ 3 months, manifest by either pathological abnormalities or abnormalities of markers of kidney damage (eg, proteinuria).[23] All subjects had already received ARBs for the treatment of hypertension. Patients were introduced to our hospital for further strict control of BP and for histopathological diagnosis of CKD (IgA nephropathy n = 20, non-IgA proliferative glomerulonephritis n = 5, membranous nephropathy n = 2, unknown etiology n = 3). We excluded any patients with chronic pulmonary disease, collagen disease, liver disease, and neoplastic disorders, and those who had recent (<6 mos) acute coronary syndromes, stroke, and any acute infections. Because BP control was inadequate, 30 patients were randomly assigned into 2 groups and followed up for 6 months: one group was treated with 16 mg azelnidipine once daily (9 males and 6 females; IgA nephropathy n = 10, non-IgA proliferative glomerulonephritis n = 2, membranous nephropathy n = 1, unknown etiology n = 2, mean age 45.3 yrs) and the other with 5 mg amlodipine once daily (9 males and 6 females; IgA nephropathy n = 10, non-IgA proliferative glomerulonephritis n = 3, membranous nephropathy n = 1, unknown etiology n = 1, mean age 45.5 yrs). During the study periods, other antihypertensive drugs including a1pha-1 blockers and diuretics were not changed, and all patients received salt restriction (<6 g/d NaCl). The study protocol was approved by the local ethical committee of Shinmatsudo Central General Hospital, and informed consent was obtained from all study participants. The study complied with the principles of the Helsinki Declaration.

Data Collection

Height and weight were measured, and body mass index (BMI; kg/m^2) was calculated as an index of the presence or absence of obesity. Systolic BP (SBP) and diastolic BP (DBP) were measured in the sitting position twice after 2 minutes of rest using an upright standard sphygmomanometer. Renal function was evaluated by estimated glomerular filtration rate (eGFR) according to the Modification of Diet in Renal Disease equation modified for the Japanese population.[24] Circulating levels of total cholesterol (T-chol), triglycerides (TG), fasting plasma glucose (FPG), and glycated hemoglobin (HbA_{1c}) were measured enzymatically at Shinmatsudo Central General Hospital. Urinary L-fatty acid binding protein, a marker of tubular injury, was measured with an enzyme-linked immunosorbent assay (ELISA) kit according to the manufacturer's protocol (CIMC, Tokyo, Japan).[5] Urinary excretion levels of 8-hydroxydeoxyguanosine (8-OHdG), a marker of oxidative stress, were measured by ELISA as reported previously.[25] Total urinary protein excretion levels were determined with a pyrogallol red method (Wako Pure Chemical Industries, Ltd., Osaka, Japan). Serum levels of AGE and sRAGE were measured with ELISA systems as described previously.[26] In this study, 1U of AGE corresponds to 1 μg of glyceraldehyde-derived AGE-bovine serum albumin as described previously.[26] Intra-assay and interassay coefficient of variations of sRAGE ELISA were 7.7% and 5.7%, respectively.

Statistical Analysis

Data were expressed as mean ± SD. The Student *t* test was used for the statistical comparisons among the parameters before and after treatments. To compare the parameters between azelnidipine- and amlodipine-treated groups, we used the Wilcoxon signed-rank test. One-way analysis of variance was used for analyses of correlations among proteinuria, AGE, and sRAGE. Statistical significance was defined as $P < 0.05$. All statistical analyses were performed with SPSS version 11.0 software (SPSS, Inc., Chicago, IL).

Results

Thirty nondiabetic hypertensive patients were randomly divided into 2 groups, one treated with azelnidipine and one treated with amlodipine. There were no significant differences in the baseline characteristics between the 2 groups, including age, sex, medications for hypertension and dyslipidemia, BMI, SBP, DBP, FPG, HbA_{1c}, T-chol, TG, eGFR, proteinuria, urinary levels of L-FABP, and AGE and sRAGE levels (Table 1). As shown in Figure 1, at baseline, proteinuria was positively correlated with circulating AGE

Table 1. Clinical Variables Before and After Treatment With Azelnidipine or Amlodipine

Characteristics	Azelnidipine		Amlodipine	
	Before	After	Before	After
Age (y)	45.3 ± 9.6	NA	45.5 ± 8.8	NA
N (male)	15 (9)	NA	15 (9)	NA
α 1-blockers (n)	4	4	4	4
Diuretics (n)	3	3	3	3
Statins (n)	4	4	4	4
BMI (kg/m²)	22.6 ± 2.0		22.8 ± 2.2	
SBP (mm Hg)	156 ± 7	131 ± 5[a]	154 ± 7	132 ± 4[b]
DBP (mm Hg)	92 ± 5	81 ± 3[a]	93 ± 4	80 ± 4[b]
T-chol (mg/dL)	164 ± 22	160 ± 20	160 ± 18	158 ± 20
TG (mg/dL)	120 ± 12	118 ± 10	118 ± 14	116 ± 12
FPG (mg/dLl)	92 ± 6	90 ± 5	93 ± 5	92 ± 4
HbA_{1c} (%)	5.2 ± 0.3	5.1 ± 0.2	5.3 ± 0.4	5.2 ± 0.3
eGFR (mL/min)	81.9 ± 12.2	82.3 ± 11.9	81.9 ± 14.6	81.9 ± 14.1
Proteinuria (g/d)	0.99 ± 0.18	0.63 ± 0.17[a,c]	1.01 ± 0.16	1.01 ± 0.16
L-FABP (μg/gCr)	71.4 ± 18.6	48.4 ± 15.8[a,c]	71.0 ± 20.1	71.0 ± 20.5
8-OHdG (ng/mgCr)	25.4 ± 3.6	17.9 ± 4.0[a,c]	25.4 ± 4.0	25.3 ± 4.3
AGE (U/mL)	14.1 ± 2.4	10.8 ± 2.1[a,c]	14.4 ± 2.1	14.3 ± 2.1
sRAGE (pg/mL)	1967.3 ± 357.0	1434.0 ± 277.6[a,c]	2012.7 ± 269.4	2010.7 ± 280.9

Abbreviations: 8-OHdG, 8-hydroxydeoxyguanosine; AGE, advanced glycation end products; BMI, body mass index; Cr, creatinine; DBP, diastolic blood pressure; eGFR, estimated glomerular filtration rate; FPG, fasting plasma glucose; HbA_{1c}, glycated hemoglobin; L-FABP, L-fatty acid binding protein; NA, not applicable; SBP, systolic blood pressure; sRAGE, soluble form of RAGE; T-chol, total cholesterol; TG, triglycerides.
Data are shown as mean ± SD unless otherwise indicated.
[a] $P < 0.01$ vs before azelnidipine treatment. [b] $P < 0.01$ vs before amlodipine treatment. [c] $P < 0.01$ vs after amlodipine treatment.

and sRAGE levels in our subjects. Both CCBs equally reduced SBP and DBP and neither of them affected glucose, HbA_{1c}, lipid levels, and eGFR; however, treatment with azelnidipine, but not amlodipine, decreased circulating AGE, sRAGE, proteinuria, and urinary levels of L-FABP and 8-OHdG in a BP-lowering–independent manner (Table 1 and Figure 2).

Discussion

We have previously reported that azelnidipine, but not amlodipine, improves renal injury in nondiabetic CKD patients.[21] However, the effects of azelnidipine or amlodipine on the AGE-RAGE system in CKD patients remained unclear. In the previous study, some patients received inhibitors of the renin-angiotensin system such as ARBs, but others did not. This could confound the effects of azelnidipine on the AGE-RAGE system, because serum levels of AGE and sRAGE can be influenced by agents that block the renin-angiotensin system.[9,27] So, in the present study, we re-enrolled other hypertensive CKD subjects who have already been treated with ARBs, and then compared the effects of azelnidipine addition on serum levels of AGE, sRAGE, proteinuria, and urinary excretion levels of L-FABP with those of amlodipine.

In this study, we demonstrated for the first time that azelnidipine, but not amlodipine, decreased serum levels of AGE and sRAGE in stage I or II nondiabetic CKD patients who have already received ARBs. Because azelnidipine and amlodipine exhibited comparable and significant BP-lowering effects in our subjects, the present study suggests that azelnidipine reduces serum AGE and sRAGE levels in a BP-lowering–independent manner.

We have previously shown that azelnidipine inhibits endothelial cell damage induced by tumor necrosis factor alpha or angiotensin II through its antioxidative properties via suppression of nicotinamide adenine dinucleotide phosphate-oxidase (NADPH).[19,20,28] The AGE-RAGE axis has been known to contribute to renal damage through the NADPH oxidase–derived oxidative stress generation.[29] In addition, azelnidipine treatment was reported to prevent glomerulosclerosis and tubulointerstitial injury in exogenously AGE-injected rats.[22] These observations suggest that azelnidipine may block the AGE-RAGE axis in the kidney and exert renoprotective properties, in our cases probably by inhibiting NADPH oxidase activity.

Published online in Wiley Online Library (wileyonlinelibrary.com)
DOI:10.1002/clc.20885 © 2011 Wiley Periodicals, Inc.

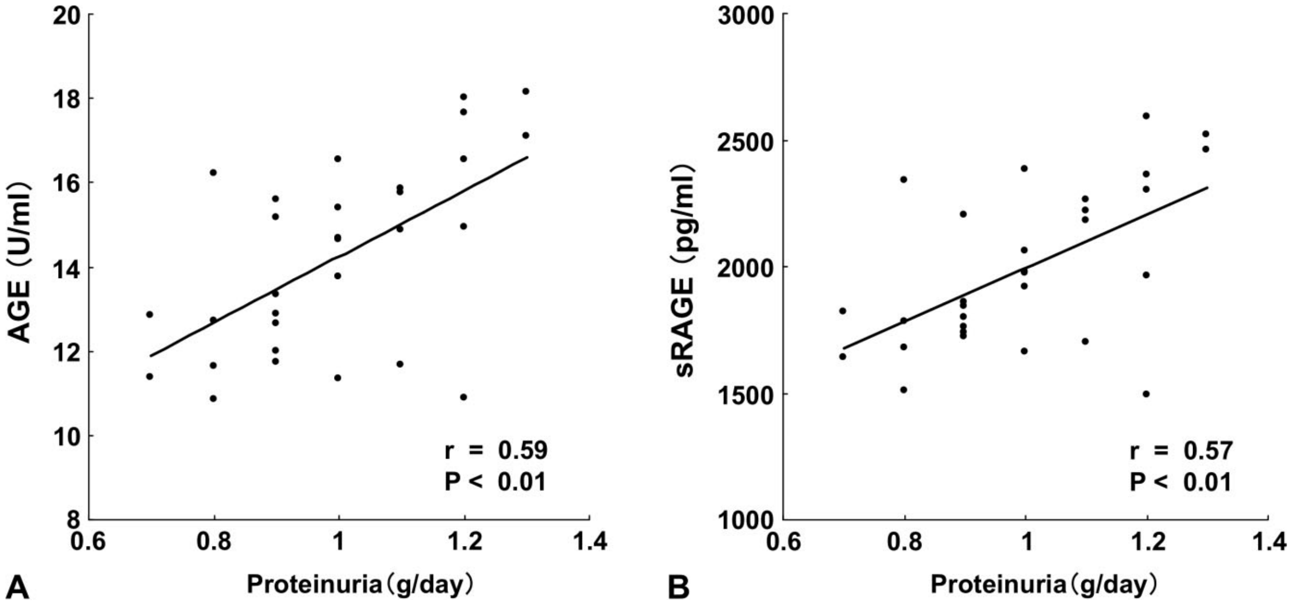

Figure 1. Correlations between proteinuria and AGE (A) and sRAGE (B). Thirty nondiabetic stage I or II CKD patients with hypertension (18 men and 12 women, mean age 45.4 yrs) were enrolled in the present study. At baseline, proteinuria was positively correlated with circulating AGE and sRAGE levels. Abbreviations: AGE, advanced glycation end products; CKD, chronic kidney disease; sRAGE, soluble form of AGE receptor.

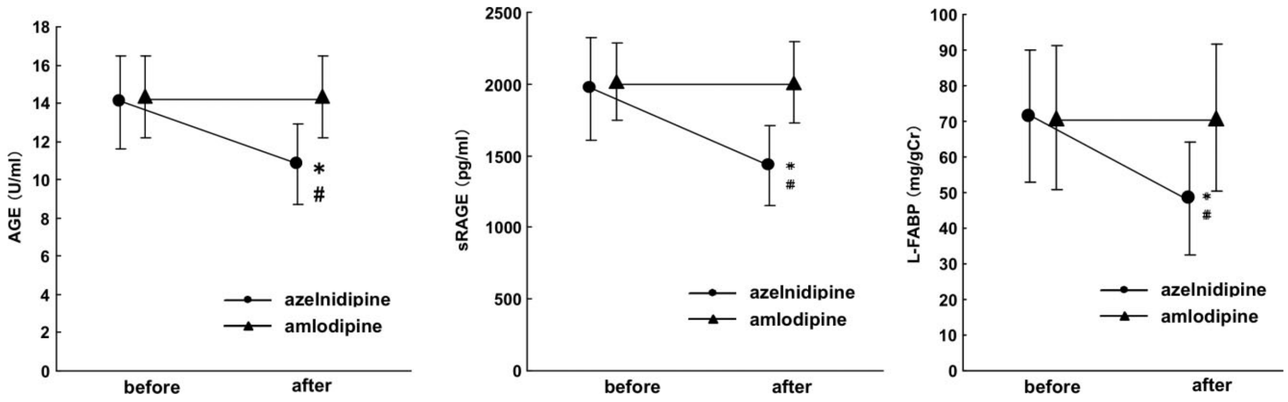

Figure 2. Serum levels of AGE (A) and sRAGE (B), and urinary levels of L-FABP (C), before and after treatment with azelnidipine or amlodipine. Thirty nondiabetic stage I or II CKD patients with hypertension (18 men and 12 women, mean age 45.4 yrs) were enrolled in the present study. All subjects had already received ARBs for the treatment of hypertension. The patients were randomly assigned into 2 groups and followed up for 6 months: one group was treated with 16 mg azelnidipine once daily (9 males and 6 females, mean age 45.3 yrs) and the other with 5 mg amlodipine once daily (9 males and 6 females, mean age 45.5 yrs). Treatment with azelnidipine, but not amlodipine, decreased circulating AGE and sRAGE and urinary levels of L-FABP in a BP-lowering–independent manner. Abbreviations: AGE, advanced glycation end products; ARB, angiotensin II type 1 receptor blocker; BP, blood pressure; CKD, chronic kidney disease; L-FABP, liver-type fatty acid binding proteins; sRAGE, soluble form of AGE receptor. *$P < 0.01$ vs before azelnidipine treatment; #$P < 0.01$ vs after amlodipine treatment.

Azelnidipine added directly to cell homogenates did not affect the NADPH oxidase activity in endothelial cells[19]; it may inhibit the upstream signaling pathways to NADPH oxidase in the kidney. Moreover, oxidative stress generation not only stimulates the formation and accumulation of AGE, but also enhances RAGE gene expression in humans, forming a vicious cycle between the AGE-RAGE axis and oxidative stress generation.[1,9] Because treatment with azelnidipine, but not amlodipine, significantly decreased urinary levels of 8-OHdG, an oxidative stress marker in our subjects (Table), the present findings suggest that azelnidipine could decrease serum levels of AGE and sRAGE in CKD patients by blocking the crosstalk between the AGE-RAGE axis and oxidative stress through its antioxidative properties.

We also found here that additional treatments with azelnidipine, but not amlodipine, significantly decreased proteinuria and urinary levels of L-FABP, a marker of tubular injury in ARB-treated CKD patients (Table and Figure 2). Albumin, one of the major components found in proteinuria, is reported to cause proinflammatory and profibrotic changes in cultured proximal tubular cells.[30,31] Clinical data show a positive correlation of the extent of proteinuria with the severity of tubulointerstitial damage in CKD patients.[32,33] These observations suggest that proteinuria-lowering effects of azelnidipine may be

ascribed, at least in part, to its tubuloprotective properties in our subjects. Although persistent proteinuria shows the existence of glomerulopathy, it has recently been recognized that changes within tubulointerstitium are more important than glomerulopathy in terms of renal prognosis in patients with CKD.[33] This could further support the clinical utility of azelnidipine for the treatment of hypertensive patients with CKD.

We could not clarify here the exact molecular mechanism of how azelnidipine reduced proteinuria in CKD patients. However, in this study, proteinuria was positively correlated with circulating AGE and sRAGE levels (Figure 1). There is a growing body of evidence that the AGE-RAGE axis plays a role in the development and progression of CKD.[9–16] Further, we, along with others, have recently found that circulating AGE and sRAGE levels could reflect tissue AGE and RAGE expression, respectively, and may be biomarkers of renal injury in both diabetic and nondiabetic patients.[5,9–16] These findings suggest that adding azelnidipine to ARBs could reduce proteinuria and exert renoprotective properties in nondiabetic CKD patients by blocking the deleterious effects of AGE-RAGE system.

Study Limitations

First, the number of patients in this study is small. As a result, the study does not have enough statistical power to draw a definite conclusion that azelnidipine treatment improved renal injury in nondiabetic CKD patients via blockade of the AGE-RAGE axis. Second, although azelnidipine is reported to block endothelial-cell damage through its antioxidative properties via suppression of NADPH oxidase activity,[19,20,28] the relationship among NADPH oxidase–driven oxidative stress generation and circulating levels of AGE and sRAGE in humans remains unclear. So, we did not know the exact mechanism of how azelnidipine exerts antioxidative properties in CKD patients. Third, although renal biopsies before and after initiating treatment would help better define the effects of individual drugs, we could not reperform renal biopsy after the drug treatments because patients refused to undergo the procedure.

Conclusion

Further large clinical study is needed to elucidate whether reduction of circulating AGE and sRAGE levels by azelnidipine could be mechanistically related to renal protection in nondiabetic CKD patients.

Acknowledgments

This study was supported in part by grants of Venture Research and Development Centers from the Ministry of Education, Culture, Sports, Science and Technology, Japan (S.Y.).

References

1. Yamagishi S, Imaizumi T. Diabetic vascular complications: pathophysiology, biochemical basis and potential therapeutic strategy. *Curr Pharm Des.* 2005;11:2279–2299.
2. Bucala R, Cerami A. Advanced glycosylation: chemistry, biology, and implications for diabetes and aging. *Adv Pharmacol.* 1992;23: 1–34.
3. Rahbar S, Figarola JL. Novel inhibitors of advanced glycation endproducts. *Arch Biochem Biophys.* 2003;419:63–79.
4. Tahara N, Imaizumi T, Takeuchi M, et al. Insulin resistance is an independent correlate of high serum levels of advanced glycation end products (AGEs) and low testosterone in non-diabetic men. *Oxid Med Cell Longev.* 2010;3:262–265.
5. Nakamura T, Sato E, Fujiwara N, et al. Positive association of serum levels of advanced glycation end products and high mobility group box-1 with asymmetric dimethylarginine in nondiabetic chronic kidney disease patients. *Metabolism.* 2009;58:1624–1628.
6. Yamagishi S, Matsui T. Advanced glycation end products (AGEs), oxidative stress and diabetic nephropathy. *Oxid Med Cell Longev.* 2010;3:101–108.
7. Bohlender JM, Franke S, Stein G, et al. Advanced glycation end products and the kidney. *Am J Physiol Renal Physiol.* 2005;289: F645–F659.
8. Raj DS, Choudhury D, Welbourne TC, et al. Advanced glycation end products: a nephrologist's perspective. *Am J Kidney Dis.* 2000;35:365–380.
9. Yamagishi S, Matsui T, Nakamura K. Kinetics, role and therapeutic implications of endogenous soluble form of receptor for advanced glycation end products (sRAGE) in diabetes. *Curr Drug Targets.* 2007;8:1138–1143.
10. Nakamura K, Yamagishi S, Adachi H, et al. Elevation of soluble form of receptor for advanced glycation end products (sRAGE) in diabetic subjects with coronary artery disease. *Diabetes Metab Res Rev.* 2007;23:368–371.
11. Tan KC, Shiu SW, Chow WS, et al. Association between serum levels of soluble receptor for advanced glycation end products and circulating advanced glycation end products in type 2 diabetes. *Diabetologia.* 2006;49:2756–2762.
12. Nakamura K, Yamagishi S, Matsui T, et al. Serum levels of soluble form of receptor for advanced glycation end products (sRAGE) are correlated with AGEs in both diabetic and non-diabetic subjects. *Clin Exp Med.* 2007;7:188–190.
13. Humpert PM, Djuric Z, Kopf S, et al. Soluble RAGE but not endogenous secretory RAGE is associated with albuminuria in patients with type 2 diabetes. *Cardiovasc Diabetol.* 2007;6:9.
14. Miura J, Yamagishi S, Uchigata Y, et al. Serum levels of non-carboxymethyllysine advanced glycation endproducts are correlated to severity of microvascular complications in patients with Type 1 diabetes. *J Diabetes Complications.* 2003;17:16–21.
15. Nakamura K, Yamagishi S, Adachi H, et al. Circulating advanced glycation end products (AGEs) and soluble form of receptor for AGEs (sRAGE) are independent determinants of serum monocyte chemoattractant protein-1 (MCP-1) levels in patients with type 2 diabetes. *Diabetes Metab Res Rev.* 2008;24:109–114.
16. Yamagishi S, Adachi H, Nakamura K, et al. Positive association between serum levels of advanced glycation end products and the soluble form of receptor for advanced glycation end products in nondiabetic subjects. *Metabolism.* 2006;55:1227–1231.
17. Tanaka N, Yonekura H, Yamagishi S, et al. The receptor for advanced glycation end products is induced by the glycation products themselves and tumor necrosis factor-alpha through nuclear factor-kappa B, and by 17beta-estradiol through Sp-1 in human vascular endothelial cells. *J Biol Chem.* 2000;275: 25781–25790.
18. Raucci A, Cugusi S, Antonelli A, et al. A soluble form of the receptor for advanced glycation endproducts (RAGE) is produced by proteolytic cleavage of the membrane-bound form by the sheddase a disintegrin and metalloprotease 10 (ADAM10). *FASEB J.* 2008;22:3716–3727.
19. Yamagishi S, Inagaki Y, Nakamura K, et al. Azelnidipine, a newly developed long-acting calcium antagonist, inhibits tumor necrosis factor-alpha-induced interleukin-8 expression in endothelial cells through its anti-oxidative properties. *J Cardiovasc Pharm.* 2004;43:724–730.
20. Matsui T, Yamagishi S, Nakamura K, et al. Azelnidipine, a new long-acting calcium-channel blocker, inhibits tumor necrosis factor-alpha-induced monocyte chemoattractant protein-1 expression in endothelial cells. *J Int Med Res.* 2006;34:671–675.

Published online in Wiley Online Library (wileyonlinelibrary.com)
DOI:10.1002/clc.20885 © 2011 Wiley Periodicals, Inc.

21. Nakamura T, Sugaya T, Kawagoe Y, et al. Azelnidipine reduces urinary protein excretion and urinary liver-type fatty acid binding protein in patients with hypertensive chronic kidney disease. *Am J Med Sci.* 2007;333:321–326.
22. Yamagishi S, Takeuchi M, Inoue H. Renoprotective effects of azelnidipine, a dihydropyridine-based calcium antagonists in advanced glycation end product (AGE)-injected rats. *Int J Tissue React.* 2005;27:137–143.
23. National Kidney Foundation. K/DOQI clinical practice guidelines for chronic kidney disease: evaluation, classification, and stratification. *Am J Kidney Dis.* 2002;39(2 suppl 1): S1–S266.
24. Imai E, Horio M, Nitta K, et al. Estimation of glomerular filtration rate by MDRD study equation modified for Japanese patients with chronic kidney disease. *Clin Exp Nephrol.* 2007;11:41–50.
25. Nakamura T, Sato E, Fujiwara N, et al. Ezetimibe decreases serum levels of asymmetric dimethylarginine (ADMA) and ameliorates renal injury in non-diabetic chronic kidney disease patients in a cholesterol-independent manner. *Pharm Res.* 2009;60: 525–528.
26. Nakamura T, Sato E, Fujiwara N, et al. Atorvastatin reduces proteinuria in non-diabetic chronic kidney disease patients partly via lowering serum levels of advanced glycation end products (AGEs). *Oxid Med Cell Longev.* 2010;3:304–307.
27. Yamagishi S, Matsui T, Nakamura K. Potential utility of telmisartan, an angiotensin II type 1 receptor blocker with peroxisome proliferator-activated receptor-gamma (PPAR-gamma)-modulating activity for the treatment of cardiometabolic disorders. *Curr Mol Med.* 2007;7:463–469.
28. Matsui T, Yamagishi S, Nakamura K, et al. Azelnidipine, a dihydropyridine-based calcium antagonist, inhibits angiotensin II-induced oxidative stress generation and downregulation of pigment epithelium-derived factor mRNA levels in microvascular endothelial cells. *Drugs Exp Clin Res.* 2005;31:215–219.
29. Thallas-Bonke V, Thorpe SR, Coughlan MT, et al. Inhibition of NADPH oxidase prevents advanced glycation end product-mediated damage in diabetic nephropathy through a protein kinase C-alpha-dependent pathway. *Diabetes.* 2008;57:460–469.
30. Matsui T, Yamagishi S, Ueda S, et al. Irbesartan inhibits albumin-elicited proximal tubular cell apoptosis and injury in vitro. *Protein Pep Lett.* 2010;17:74–77.
31. D'Amico G, Bazzi C. Pathophysiology of proteinuria. *Kidney Int.* 2003;63:809–825.
32. Burton C, Harris KP. The role of proteinuria in the progression of chronic renal failure. *Am J Kidney Dis.* 1996;27:765–775.
33. Ziyadeh FN, Goldfarb S. The renal tubulointerstitium in diabetes mellitus. *Kidney Int.* 1991;39:464–475.

Published online in Wiley Online Library (wileyonlinelibrary.com)
DOI:10.1002/clc.20885

© 2011 Wiley Periodicals, Inc.
