# Supplementary material for: Effects of T-Type Calcium Channel Blockers on Renal Function and Aldosterone in Patients with Hypertension: A Systematic Review and Meta-Analysis
Source: PLoS One. 2014 Oct 17;9(10):e109834. doi: 10.1371/journal.pone.0109834 (PMC4201480; doi:10.1371/journal.pone.0109834)
Supplement: File S3 — PDF files of twenty-four studies included in the meta-analysis. (ZIP) [file pone.0109834.s007.zip › Supporting information-PDF files/15. Hypertens Res 2011[34(2)]268-273.pdf]

Hypertension Research (2011) 34, 268–273
© 2011 The Japanese Society of Hypertension All rights reserved 0916-9636/11 $32.00
www.nature.com/hr

ORIGINAL ARTICLE

Benidipine reduces albuminuria and plasma aldosterone in mild-to-moderate stage chronic kidney disease with albuminuria

Masanori Abe[1], Kazuyoshi Okada[1], Noriaki Maruyama[1], Shiro Matsumoto[1], Takashi Maruyama[1], Takayuki Fujita[1], Koichi Matsumoto[1] and Masayoshi Soma[1,2]

Benidipine inhibits both L- and T-type Ca channels, and has been shown to dilate the efferent arterioles as effectively as the afferent arterioles. In this study, we conducted an open-label and randomized trial to compare the effects of benidipine with those of amlodipine on blood pressure (BP), albuminuria and aldosterone concentration in hypertensive patients with mild-to-moderate stage chronic kidney disease (CKD). Patients with BP$\geqslant$130/80 mm Hg, with estimated glomerular filtration rate (eGFR) of 30–90 ml min^{-1} per 1.73 m^2, and with albuminuria$>$30 mg per g creatinine (Cr), despite treatment with the maximum recommended dose of angiotensin II receptor blockers (ARBs) were randomly assigned to two groups. Patients received either of the following two treatment regimens: 2 mg per day benidipine, which was increased up to a dose of 8 mg per day (n=52), or 2.5 mg per day amlodipine, which was increased up to a dose of 10 mg per day (n=52). After 6 months of treatment, a significant and comparable reduction in the systolic and diastolic BP was observed in both groups. The decrease in the urinary albumin to Cr ratio in the benidipine group was significantly lower than that in the amlodipine group. Although plasma renin activity was not different in the two groups, plasma aldosterone levels were significantly decreased in the benidipine group. Moreover, urinary Na/K ratio was significantly decreased in the benidipine group but remained unchanged in the serum. It may be concluded that benidipine results in a greater reduction of plasma aldosterone and albuminuria than amlodipine, and that these effects are independent of BP reduction.
Hypertension Research (2011) **34,** 268–273; doi:10.1038/hr.2010.221; published online 2 December 2010

Keywords: albuminuria; aldosterone; amlodipine; benidipine; chronic kidney disease

INTRODUCTION

According to the hyperfiltration theory, an increase in glomerular capillary pressure (that is, glomerular hypertension) has an important role in the progression of diabetic and non-diabetic kidney disease.[1,2] Intraglomerular capillary pressure is dependent on the tone of the glomerular arterioles as well as the level of systemic arterial pressure. Because angiotensin II is a potent constrictor of the efferent arterioles, angiotensin-converting enzyme (ACE) inhibitors and angiotensin II receptor blockers (ARBs), which inhibit the generation or functions of angiotensin II, are effective in alleviating glomerular hypertension.[3] Therefore, ACE inhibitors or ARBs are the first-line treatment options for patients with chronic kidney disease (CKD).

Following recent molecular biological studies, Ca channels have been classified into five subtypes, namely, L, T, N, P/Q and R, according to their location and function.[4,5] Three types of Ca channel blockers (CCBs), the L-, T-, and N-type CCBs, are in clinical use. Both L-type and T-type Ca channels are present in the afferent arterioles, whereas only T-type Ca channels are present in the efferent arterioles. Benidipine has been shown to block both L-type and T-type Ca channels and causes dilatation of both efferent and afferent arterioles.[6]

Aldosterone has an important role in the pathophysiology of hypertension, cardiac hypertrophy and heart failure. Targeting aldosterone synthesis and/or secretion represents a potentially useful approach for the prevention of cardiovascular disease.[7] Aldosterone is synthesized in the zona glomerulosa of the adrenal glands, in which it is secreted in a Ca^{2+}-dependent manner in response to stimuli such as angiotensin II, adrenocorticotropic hormone and increased extracellular K^+.[8] Furthermore, T-type Ca channels are present in the adrenal glands and the results of a recent *in vivo* study have demonstrated that T-type CCBs inhibit aldosterone production in cultured adrenal cells.[9] However, there have been few clinical studies indicating the role of CCBs in reducing plasma aldosterone concentration in patients with CKD. Therefore, we considered that it would be intriguing to compare the effects of a T-type CCB and an L-type

[1]Division of Nephrology, Hypertension and Endocrinology, Department of Internal Medicine, Nihon University School of Medicine, Tokyo, Japan and [2]Division of General Medicine, Department of Internal Medicine, Nihon University School of Medicine, Tokyo, Japan
Correspondence: Dr M Abe, Division of Nephrology, Hypertension and Endocrinology, Department of Internal Medicine, Nihon University School of Medicine, 30-1, Oyaguchi Kami-chou, Itabashi-ku, Tokyo 173-8610, Japan.
E-mail: mabe@med.nihon-u.ac.jp
Received 9 July 2010; revised 12 August 2010; accepted 24 August 2010; published online 2 December 2010

CCB on albuminuria and on the renin–angiotensin–aldosterone system in patients with CKD. In the present randomized study, we compared the clinical effects of benidipine (an L- and T-type CCB) with amlodipine (an L-type CCB) in albuminuric patients with mild-to-moderate stage CKD.

METHODS

We conducted a 6-month, single-center, prospective, randomized and open-label clinical trial. This study was designed to compare the effects of benidipine and amlodipine on blood pressure (BP), plasma aldosterone levels and urinary albumin excretion ratio in hypertensive and albuminuric patients with CKD. Participants were already receiving the maximum recommended dose of ARBs. All study participants provided written informed consent, and the trial protocol was approved by the ethics committee of our institution. This study was conducted in accordance with the Declaration of Helsinki. This prospective study was conducted between June 2009 and May 2010 and the subjects were followed up for 6 months.

The enrollment criteria included (1) hypertension (that is, systolic/diastolic BP $\geqslant$130/80 mm Hg, measured in the sitting position on at least two separate clinic visits); (2) stage 2–3 CKD (that is, an estimated glomerular filtration rate (eGFR) of 30–90 ml min^{-1} per 1.73 m^2) with albuminuria (that is, urinary albumin/creatinine (Cr) ratio $\geqslant$30 mg g^{-1}; average of two consecutive measurements taken during a 4-week period before treatment); and (3) treatment with the maximum recommended dose of an ARB (80 mg per day telmisartan or 40 mg per day olmesartan) for at least 8 weeks before the study.

The exclusion criteria were (1) age $<$20 years or $>$80 years; (2) hypertensive emergency; (3) history of severe heart failure, angina, myocardial infarction or stroke within 6 months before the start of the trial; (4) previous treatment with steroids or immunosuppressants; (5) renovascular hypertension, as determined by renal Doppler ultrasonography before enrollment in the study, or endocrine hypertension; and (6) severe diabetes mellitus, resulting in hospitalization because of extremely high plasma glucose, or with complications such as diabetic ketoacidosis.

Subjects were randomly assigned to two groups before the start of the study. An independent investigator, who had no previous knowledge of the subjects before the commencement of the trial, monitored randomization in the order of entry of the subjects; then, the details of assignment were immediately given to the individual investigators. Dynamic balancing randomization was carried out on the basis of the levels of serum Cr (sCr), urinary albumin/Cr ratio measured at the time of registration, and the presence or absence of diabetic nephropathy. Thus, we ensured that there were no significant differences between the baseline characteristics of the two groups. Patients received either of the following two treatment regimens: benidipine, 2 mg per day, which was increased to daily doses of 8 mg (benidipine group), and amlodipine, 2.5 mg per day, which was increased to daily doses of 10 mg (amlodipine group). In addition, none of the subjects from our previous study[10] was included in either group in this study.

BP was measured at the outpatient clinic at fixed times after the medications were administered. BP measurement was carried out according to the Japanese Society of Hypertension 2009 guidelines.[11] BP was measured monthly using a sphygmomanometer (Nippon Colin, Tokyo, Japan) in duplicate with the patient in the sitting position after a 5-min rest. Patients, particularly those with dietary restrictions, were given guidance on how to maintain their diet.

Doses of ARBs and ACE inhibitors were not altered during the study period. The target BP level was $<$130/80 mm Hg. During the study period, patients were administered combination drug therapy together with other conventional antihypertensive agents at baseline.

Withdrawal of treatment was considered for patients who developed an allergy/intolerance to benidipine or amlodipine during the study period, developed a hypertensive emergency, or developed any other condition or received another therapy that, in the opinion of the investigators, might pose a risk for the patient or confound the results of the study. Furthermore, patients who were administered additional antihypertensive medications (other than ARBs or CCBs) to achieve the target BP, because benidipine or amlodipine failed to reduce the BP to the target level, were not included in the analysis.

The primary end point was change in urinary albumin/Cr ratio measured from baseline to 1, 3 and 6 months after the start of treatment. All parameters for monitoring the effects of the drugs were evaluated once a month during the 6-month period of treatment. Laboratory values of, for example, sCr and electrolytes were measured using commercial kits employing routinely used clinical chemistry procedures. To assess urinary albumin excretion, we measured the urinary concentrations of albumin and Cr (albumin/Cr ratio) in an early-morning spot urine sample. Urinary albumin was measured by the pyrogallol red method. Treatment compliance and safety variables were monitored at each visit to our hospital. Plasma renin activity and aldosterone concentrations were measured by radioimmunoassay at a contact laboratory (SRL, Tokyo, Japan) at baseline and at the end of the study in the supine position after a 20-min rest. To assess urinary Na and K excretion, we measured the urinary concentrations of Na and K (urinary Na/K ratio) at baseline and at the end of the study by using early-morning spot urine sample. Glomerular filtration rate was estimated using the final recommended modified equation for Japanese patients by the Japanese Society of Nephrology-CKD Initiatives (JSN-CKDI), because eGFR obtained by this method is more accurate for Japanese patients with CKD.[12] The following formula was used: eGFR (ml min^{-1} per 1.73 m^2)$=194\times sCr^{-1.094}\times age^{-0.287}$ ($\times$0.739 for women).

Statistical analysis

Data were analyzed according to the randomly assigned groups, regardless of participants' subsequent medication (intention-to-treat analysis) and expressed as mean ± s.e.m. Baseline characteristics of the enrolled patients were compared between treatment groups using the unpaired *t*-test. The mean values of the two groups were compared by the unpaired *t*-test. Analysis of variance with repeated measurements and subsequent multiple comparison tests were applied to determine the effect of treatment on BP, heart rate and urinary albumin/Cr ratio. Statistical significance was set at $P<0.05$.

RESULTS

Study population and baseline characteristics

A total of 104 subjects were enrolled in this study and randomly allocated to the benidipine group (n=52) or amlodipine group (n=52). Baseline characteristics and medications at baseline in the two groups are shown in Table 1. No significant differences were observed between the two groups with regard to baseline characteristics or the number of patients with diabetic nephropathy. Adequate BP control had not been achieved at baseline in any of the enrolled patients. During treatment, two subjects from each group withdrew from the study because additional antihypertensive medications (benidipine group: furosemide, n=1; thiazide diuretics, n=1; amlodipine group: furosemide, n=2) were required to achieve the target BP or improve the edema. Therefore, 100 subjects completed the trial.

BP-lowering effect

The final doses of benidipine and amlodipine were 6.3 ± 0.3 and 5.4 ± 0.4 mg per day, respectively, in the two groups. Figure 1 shows that systolic and diastolic BPs were significantly lower in both groups at 1 month after the initiation of CCB therapy compared with baseline values. Systolic and diastolic BP did not differ between the two groups during the course of treatment. In the last month of treatment, no differences were observed between systolic BP (benidipine group: 128.3 ± 1.0 mm Hg; amlodipine group: 127.7 ± 1.0 mm Hg, not significant (NS)) and diastolic BP (benidipine group: 73.3 ± 1.4 mm Hg; amlodipine group: 73.3 ± 1.5 mm Hg, NS) in the two groups. The target BP value (130/80 mm Hg) was achieved in 56.0 and 60.0% of all subjects in the benidipine and amlodipine groups (NS), respectively. As shown in Figure 1, heart rate at the end of the study was significantly reduced in the benidipine group compared with baseline (benidipine group: from 75.1 ± 1.4 to 73.7 ± 1.3 beats per min, $P<0.001$; amlodipine group: from 74.5 ± 1.7 to 74.8 ± 1.6 beats per

min, NS), but this difference was not observed between the two groups at the end of the trial (benidipine group: 73.7 ± 1.3 beats per min; amlodipine group: 74.8 ± 1.6 beats per min, NS).

Renoprotective effects

sCr levels were not significantly changed in either group after 6 months of treatment, and the difference observed at the end of the study between the two groups was not significant (benidipine group: 1.30 ± 0.07 mg per 100 ml; amlodipine group: 1.30 ± 0.06 mg per 100 ml, NS). Furthermore, eGFR was not significantly changed in either group at the end of the study period; the difference between the groups was also not significant (benidipine group: 44.3 ± 2.1 ml min^{-1} per 1.73 m^2; amlodipine group: 42.7 ± 1.9 ml min^{-1} per 1.73 m^2, NS).

Although the urinary albumin/Cr ratio did not change significantly in the amlodipine group throughout the study period, it did decrease significantly in the benidipine group from 3 months of treatment compared with the baseline value. Furthermore, the urinary albumin/Cr ratio in the last month was significantly lower in the benidipine group than in the amlodipine group (benidipine group: 120 ± 14 mg per g Cr; amlodipine group: 163 ± 34 mg per g Cr, $P<0.01$). When the percent change from baseline was calculated, there was a significant difference between the two groups from 3 months of treatment (−24.8 ± 4.6 % *vs.* −0.5 ± 0.1%, $P<0.0001$) (Figure 2).

Table 1 Baseline characteristics and medication details of the study subjects

Parameters	*Amlodipine group*	*Benidipine group*	P-*value*
Age (years)	67.5 ± 1.5	67.3 ± 1.4	NS
Gender (male/female)	30/22	30/22	NS
Diabetes mellitus (*n* (%))	23 (44.2)	24 (46.1)	NS
Cause of chronic kidney disease (*n* (%))			
Diabetic nephropathy	23 (44.2)	24 (46.0)	NS
Chronic glomerulonephritis	15 (28.8)	14 (27.0)	NS
Hypertensive nephrosclerosis	14 (27.0)	14 (27.0)	NS
Heart rate (beats per min)	74.5 ± 1.7	75.1 ± 1.4	NS
Systolic blood pressure (mm Hg)	145 ± 1.0	144 ± 0.9	NS
Diastolic blood pressure (mm Hg)	81 ± 1.3	82 ± 1.4	NS
Serum creatinine (mg per 100 ml)	1.25 ± 0.06	1.23 ± 0.05	NS
Estimated GFR (ml min^{-1} per 1.73 m^2)	45.1 ± 1.8	44.5 ± 1.9	NS
Hemoglobin A1c (for diabetes)	6.48 ± 0.15 (*n*=23)	6.43 ± 0.11 (*n*=24)	NS
Sodium (mEq l^{-1})	140 ± 0.3	140 ± 0.4	NS
Potassium (mEq l^{-1})	4.4 ± 0.1	4.5 ± 0.1	NS
Baseline therapy			
Details of ARB (*n* (%))			
Telmisartan (80 mg daily)	28 (53.8)	29 (55.8)	NS
Olmesartan (40 mg daily)	24 (46.2)	23 (44.2)	NS
ACE inhibitors (*n* (%))	4 (7.7)	3 (5.8)	NS
Diuretics (*n* (%))	7 (13.4)	6 (11.5)	NS
β-Blockers (*n* (%))	4 (7.7)	5 (9.6)	NS
α-Blockers (*n* (%))	4 (7.7)	4 (7.7)	NS
Spironolactone (*n* (%))	0 (0)	0 (0)	NS

Abbreviations: ACE, angiotensin-converting enzyme; ARB, angiotensin receptor blocker; GFR, glomerular filtration rate; NS, not significant.

Plasma renin activity and aldosterone levels

As shown in Figure 3, plasma renin activity was not significantly changed in either group after 6 months of treatment, and the difference at the end of the study between the two groups was not significant (benidipine group: from 1.75 ± 0.18 to 1.98 ± 0.18 ng ml^{-1} per hour; amlodipine group: from 1.75 ± 0.25 to 1.97 ± 0.33 ng ml^{-1} per hour, NS). Plasma aldosterone levels were not significantly changed in the amlodipine group. However, plasma aldosterone levels were decreased in the benidipine group, and there was a significant difference between the two groups at the end of study (71.9 ± 5.0 *vs.* 90.4 ± 5.0 pg ml^{-1}, $P<0.05$).

Serum and urinary Na^+ and K^+ levels

Although the urinary Na/Cr ratio tended to increase in both groups, this was not statistically significant. However, the urinary K/Cr ratio was significantly decreased and the Na/K ratio was significantly increased in the benidipine group compared with the baseline value. In contrast, there were no significant changes in serum Na or K level in either group (Table 2).

Adverse events

Adverse reactions were not observed in the benidipine group or the amlodipine group. However, during treatment, two subjects from each group withdrew from the study because additional antihypertensive medications (furosemide, *n*=3; thiazide diuretics, *n*=1) were required

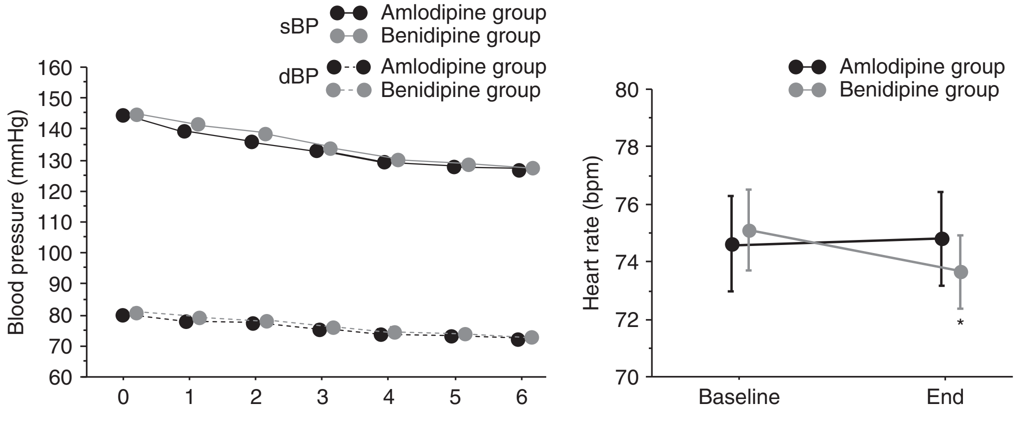

Figure 1 Changes in blood pressure and heart rate during the study period. sBP, systolic blood pressure; dBP, diastolic blood pressure. *$P<0.001$, *vs.* baseline.

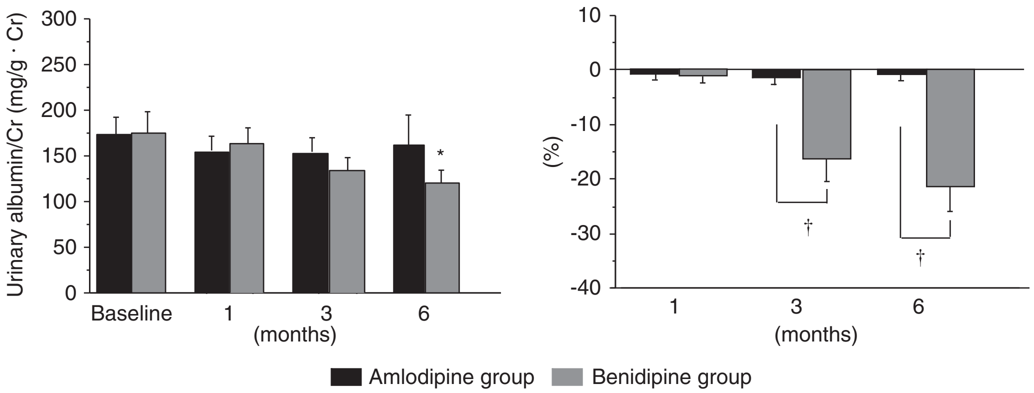

Figure 2 Changes in the urinary albumin/Cr ratio and the respective percent changes from baseline. Cr, creatinine. $^{*}P<0.01$, $^{\dagger}P<0.0001$ *vs.* amlodipine group.

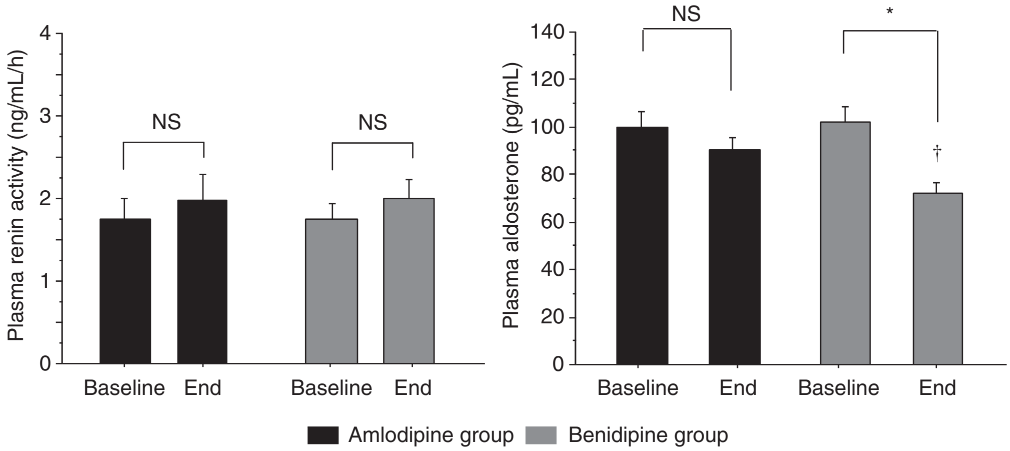

Figure 3 Changes in plasma renin activity and plasma aldosterone levels in the two groups. NS, not significant; $^{*}P<0.01$ *vs.* baseline; $^{\dagger}P<0.05$ *vs.* amlodipine group.

Table 2 Changes in serum and urinary sodium and potassium levels in the two groups

	Amlodipine group (n=50)		*Benidipine group* (n=50)	
	At baseline	*At end*	*At baseline*	*At end*
Serum Na (mEq l^{-1})	140±0.3	140±0.3	140±0.4	140±0.4
Serum K (mEq l^{-1})	4.4±0.1	4.4±0.1	4.5±0.1	4.5±0.1
Urinary Na/Cr ratio	1.61±0.1 5	1.71±0.1 4	1.62±0.16	1.78±0.18
Urinary K/Cr ratio	0.450±0.025	0.455±0.031	0.425±0.023	0.362±0.021*†
Urinary Na/K ratio	3.94±0.35	3.90±0.13	3.90±0.33	4.59±0.39*†

Abbreviations: Cr, creatinine; Na, sodium; K, potassium.
$^{*}P<0.05$ *vs.* baseline; $^{\dagger}P<0.05$ *vs.* amlodipine group.

to achieve the target BP (*n*=1), and edema developed in the lower limbs (*n*=3). Edema resolved with diuretic therapy without discontinuation of CCBs. There were no severe adverse reactions in any of the subjects in either group.

DISCUSSION

Proteinuria is detrimental to the kidney because ultrafiltration of proteins across the glomerular basement membrane brings about mesangial and tubular protein overload, which provokes inflammation and ultimately results in glomerulosclerosis and tubulo-interstitial fibrosis.[13,14] It is known that proteinuria is associated with the risk of cardiovascular disease.[15–17] Therefore, because of the antiproteinuric effect, benidipine would seem to be more advantageous than an L-type CCB, not only by slowing the progression of renal tissue injuries but also by reducing the incidence of cardiovascular events in patients with CKD. In terms of reducing proteinuria in diabetic and non-diabetic renal disease, ACE inhibitors and ARBs have been shown to be more effective than other classes of antihypertensive agents.[18,19] However, it has been reported that the antiproteinuric effects of T-type CCBs are not mediated by angiotensin II inhibition but by blockade of T-type Ca channels in the glomerular arterioles.[6] Furukawa *et al.*[20] surveyed the blocking activity of various CCBs on L-/T-/N-type Ca current in Xenopus oocytes. Although both benidipine and amlodipine exhibited blocking function on both L- and T-type Ca channels, benidipine predominantly blocked the T-type Ca channel, whereas amlodipine predominantly blocked the L-type Ca channel. Furthermore, the concentration required for benidipine to exert the inhibitory effect on T-type Ca channels can be achieved at clinically relevant doses of benidipine used for the treatment of hypertension.[20,21] Therefore, benidipine, an L-/T-type CCB, should offer additive benefits over CCB-inhibiting L-type Ca channels, but not T-type Ca channels. Furthermore, T-type Ca channels are distributed in the cardiac sinus node and are closely associated with pacemaking potentials.[4,22,23] Therefore, in this study, it was suggested that the significant decrease in heart rate was induced by the benidipine

treatment. However, further studies are needed to clarify the effect of benidipine in this regard because little data exist for this finding.

Recently, it has been demonstrated that aldosterone can directly damage various organs, including the heart and blood vessels, via mineralocorticoid receptors, independent of changes in BP.[24–26] It has been suggested that hypertensive incidents and organ damage in humans may occur even at plasma aldosterone levels within the normal physiological range.[24,27–29] Therefore, targeting aldosterone synthesis and release may be clinically important in preventing cardiovascular disease. Ca^{2+} ions are conveyed through the T-type Ca channel to the mitochondria, where they activate aldosterone synthesis, which in turn stimulates T-type Ca channel expression,[30,31] creating a positive feedback loop of aldosterone biosynthesis in adrenal cells. Efonidipine, another T-type CCB, has been shown to inhibit aldosterone synthesis and secretion *in vitro*,[9] as well as in both healthy subjects and patients with essential hypertension.[32,33] In this study, benidipine and amlodipine had no significant effect on clinic blood pressure, but both heart rate and plasma aldosterone level were significantly decreased in the benidipine treatment group. T-type CCBs inhibit renin secretion and renin gene expression *in vivo*, whereas L-type CCBs function as stimulators of the renin system.[34,35] T-type CCBs have a dual effect on plasma renin activity and renin messenger RNA expression *in vivo*, with low concentrations stimulating and high concentrations inhibiting the renin system.[36] However, in this study, plasma renin activity did not change significantly; therefore, the change in aldosterone may not have been due to the effect of renin but rather to a direct inhibition of aldosterone secretion. Furthermore, although serum Na and K levels were not changed after treatment with either CCB, urinary K/Cr and Na/K ratios were decreased in the benidipine group without any decrease in eGFR. Therefore, these findings suggest that benidipine decreased plasma aldosterone levels without any effect on serum Na and K levels when administered to patients with mild-to-moderate stage CKD. Thus, in addition to lowering blood pressure, benidipine would be expected to provide renoprotection by inhibiting aldosterone production. Long-term treatment with ACE inhibitors or ARBs can cause escape or rebound of plasma aldosterone levels. In such a situation, addition of a mineralocorticoid receptor blocker has been shown to reduce proteinuria in patients taking inhibitors of the renin–angiotensin–aldosterone system for the treatment of hypertension and kidney disease; a T-type CCB might be a suitable addition for the purpose of inhibiting aldosterone production and managing aldosterone escape.

Our study is limited by the relatively small sample size and short period of treatment. Moreover, although a prospective, randomized, open-label and parallel-group comparison design was used in this study, more longitudinal, double-blind, comparative and multicenter clinical trials should be conducted in a larger number of patients in order to further clarify the comparative usefulness of these two agents. To examine the effects of aldosterone reduction, we measured the urinary Na/K ratio using early-morning spot urine. However, because this method might not accurately reflect the plasma aldosterone reduction, measuring the urinary Na/K ratio of a 24-h urine collection is needed. In addition to BP profile, the requirement for renal replacement therapy and other renal or cardiovascular events should also be considered as end points.

In conclusion, the present study showed that benidipine, an L- and T-type CCB, results in a greater reduction of albuminuria and plasma aldosterone than amlodipine, an L-type CCB, in patients with CKD. These effects of benidipine would seem to make the drug more advantageous in terms of the progression of renal dysfunction and preventing cardiovascular tissue and organ injuries in patients with hypertension and CKD. However, further studies will be needed in order to determine whether long-term use of benidipine can reduce renal events and cardiovascular morbidity in patients with CKD.

1 Hostetter TH, Olson JL, Rennke HG, Venkatachalam MA, Brenner BM. Hyperfiltration in remnant nephrons: a potentially adverse response to renal ablation. *Am J Physiol* 1981; **241**: F85–F93.
2 Brenner BM, Lawler EV, Mackenzie HS. The hyperfiltration theory: a paradigm shift in nephrology. *Kidney Int* 1996; **49**: 1774–1777.
3 Remuzzi G, Perico N, Macia M, Ruggenenti P. The role of renin-angiotensin- aldosterone system in the progression of chronic kidney disease. *Kidney Int* 2005; **99**: S57–S65.
4 Perez-Reyes E. Molecular physiology of low-voltage-activated T-type calcium channels. *Physiol Rev* 2003; **83**: 117–161.
5 Jones SW. Overview of voltage-dependent calcium channels. *J Bioenerg Biomembr* 1998; **30**: 299–312.
6 Hayashi K, Wakino S, Sugano N, Ozawa Y, Homma K, Saruta T. Ca2+ channel subtypes and pharmacology in the kidney. *Circ Res* 2007; **100**: 342–353.
7 Takeda Y. Pleiotropic actions of aldosterone and the effects of eplerenone, a selective mineralcorticoid receptor antagonists. *Hypertens Res* 2004; **27**: 781–789.
8 Muller J. Aldosterone: the minority hormone of the adrenal cortex. *Steroids* 1995; **60**: 2–9.
9 Imagawa K, Okayama S, Takaoka M, Kawata H, Nata N, Nakajima T, Horii M, Uemura S, Saito Y. Inhibitory effect of efonidipine on aldosterone synthesis and secretion in human adenocarcinoma (H295R) cells. *J Cardiovasc Pharmacol* 2006; **47**: 133–138.
10 Abe M, Okada K, Maruyama T, Maruyama N, Matsumoto K. Comparison of the antiproteinuric effects of the calcium channel blockers benidipine and amlodipine administered in combination with angiotensin receptor blockers to hypertensive patients with stage 3-5 chronic kidney disease. *Hypertens Res* 2009; **32**: 270–275.
11 Japanese Society of Hypertension. Japanese Society of Hypertension Guidelines for the Management of Hypertension (JSH 2009). *Hypertens Res* 2009; **32**: 4–107.
12 Matsuo S, Imai E, Horio M, Yasuda Y, Tomita K, Nitta K, Yamagata K, Tomino Y, Yokoyama H, Hishida A. Collaborators developing the Japanese equation for estimated GFR: revised equations for estimated GFR from serum creatinine in Japan. *Am J Kidney Dis* 2009; **53**: 982–992.
13 Abbate M, Benigni A, Bertani T, Remuzzi G. Nephrotoxicity of increased glomerular protein traffic. *Nephrol Dial Transplant* 1999; **14**: 304–312.
14 Abbate M, Zoja C, Morigi M, Rottoli D, Angioletti S, Tomasoni S, Zanchi C, Longaretti L, Donadelli R, Remuzzi G. Transforming growth factor-beta 1 is up-regulated by podocytes in response to excess intraglomerular passage of proteins: a central pathway in progressive glomerulosclerosis. *Am J Pathol* 2002; **161**: 2179–2193.
15 Culleton BF, Larson MG, Parfrey PS, Kannel WB, Levy D. Proteinuria as a risk factor for cardiovascular disease and mortality in older people: a prospective study. *Am J Med* 2000; **109**: 1–8.
16 Dykeman-Sharpe J. Proteinuria, a modifiable risk factor: angiotensin converting enzyme inhibitors (ACEIs) and angiotensin II receptor blockers (ARBs). *CANNT J* 2003; **13**: 34–38.
17 Hemmelgarn BR, Manns BJ, Lloyd A, James MT, Klarenbach S, Quinn RR, Wiebe N, Tonelli M. Relation between kidney function, proteinuria, and adverse outcomes. *JAMA* 2010; **303**: 423–429.
18 Raij L. Recommendations for the management of special populations: renal disease in diabetes. *Am J Hypertens* 2003; **16**: 46S–49S.
19 Ruggenenti P. Angiotensin-converting enzyme inhibition and angiotensin II antagonism in nondiabetic chronic nephropathies. *Semin Nephrol* 2004; **24**: 158–167.
20 Furukawa T, Nukada T, Miura R, Ooga K, Honda M, Watanabe S, Koganesawa S, Isshiki T. Differential blocking action of dihydropyridine Ca^{2+} antagonists on T-type Ca^{2+} channel (α1G) expressed in xenopus oocytes. *J Cardiovasc Pharmacol* 2005; **45**: 241–246.
21 Akizuki O, Inayoshi A, Kitayama T, Yao K, Shirakura S, Sasaki K, Kusaka H, Matsubara M. Blockade of T-type voltage-dependent Ca^{2+} channels by benidipine, a dihydropyridine calcium channel blocker, inhibits aldosterone production in human adrenocortical cell line NCI-H295R. *Eur J Pharmacol* 2008; **584**: 424–434.
22 Ertel SI, Ertel EA, Clozel J-P. T-type Ca^{2+} channels and pharmacological blockade: potential pathophysiological relevance. *Cardiovasc Drugs Ther* 1997; **11**: 723–739.
23 Nitta Y, Yamamoto R, Yamaguchi Y, Katsuda S, Kaku B, Taguchi T, Takabatake S, Nakahama K, Yamagisi M. Impact of long-acting calcium channel blockers on the prognosis of patients with coronary artery disease with and without chronic kidney disease: a comparison of three drugs. *J Int Med Res* 2010; **38**: 253–265.
24 Tsutamoto T, Wada A, Maeda K, Mabuchi N, Hayashi M, Tsutsui T, Ohnishi M, Sawaki M, Fujii M, Matsumoto T, Matsui T, Kinoshita M. Effects of spironolactone on plasma brain natriuretic peptide and left ventricular remodeling in patients with congestive heart failure. *J Am Coll Cardiol* 2001; **37**: 1228–1233.
25 Pitt B, Zannad F, Remme WJ, Cody R, Castaigne A, Perez A, Palensky J, Wittes J, Randomized Aldactone Evaluation Study Investigators. The effect of spironolactone on morbidity and mortality in patients with severe heart failure. *N Engl J Med* 1999; **341**: 709–717.
26 Pitt B, Remme W, Zannad F, Neaton J, Martinez F, Roniker B, Bittman R, Hurley S, Kleiman J, Gatlin M, Eplerenone Post–Acute Myocardial Infarction Heart Failure Efficacy and Survival Study Investigators. Eplerenone, a selective aldosterone blocker, in patients with left ventricular dysfunction after myocardial infarction. *N Engl J Med* 2003; **348**: 1309–1321.

27 Vasan RS, Evans JC, Benjamin EJ, Levy D, Laeson MG, Sundstrom J, Murabito JM, Sam F, Colucci WS, Wilson PWF. Relations of serum aldosterone to cardiac structure: gender-related difference in the Framingham Heart Study. *Hypertension* 2004; **43**: 957–962.
28 Schunkert H, Hense HW, Muscholl M, Luchner A, Kurzinger S, Danser AH, Riegger GA. Associations between circulating components of the renin-angiotensin-aldosterone system and left ventricular mass. *Heart* 1997; **77**: 24–31.
29 Vasan RS, Evans JC, Larson MG, Wilson PWF, Meigs JB, Rifai N, Benjamin EJ, Levy D. Serum aldosterone and the incidence of hypertension in nonhypertensive persons. *N Engl J Med* 2004; **351**: 33–41.
30 Rossier MF, Lesouhaitier O, Perrier E, Bockhorn L, Chiappe A, Lalevee N. Aldosterone regulation of T-type calcium channels. *J Steroid Biochem Mol Biol* 2003; **85**: 383–388.
31 Laleve N, Rebsamen MC, Barrere-Lemaire S, Perrier E, Nargeot J, Benitah JP, Rossier MF. Aldosterone increases T-type calcium channel expression and *in vitro* beating frequency in neonatal rat cardiomyocytes. *Cardiovasc Res* 2005; **67**: 216–224.
32 Tanaka T, Tsutamoto T, Sakai H, Fujii M, Yamamoto T, Horie M. Comparison of the effects of efonidipine and amlodipine on aldosterone in patients with hypertension. *Hypertens Res* 2007; **30**: 691–697.
33 Okayama S, Imagawa K, Naya N, Iwama H, Somekawa S, Kawata H, Horii M, Nakajima T, Uemura S, Saito Y. Blocking T-type Ca2+ channels with efonidipine decreased plasma aldosterone concentration in healthy volunteers. *Hypertens Res* 2006; **29**: 493–497.
34 Wagner C, Kramer BK, Hinder M, Kieninger M, Kurtz A. T-type and L-type calcium channel blockers exert opposite effects on renin secretion and renin gene expression in conscious rats. *Br J Pharmacol* 1998; **124**: 579–585.
35 Karam H, Clozel JP, Bruneval P, Gonzalez MF, Menard J. Contrasting effects of selective T- and L-type calcium channel blockade on glomerular damage in DOCA hypertensive rats. *Hypertension* 1999; **34**: 673–678.
36 Schweda F, Kurtz A. Cellular mechanism of renin release. *Acta Physiol Scand* 2004; **181**: 383–390.
