# Supplementary material for: Effects of T-Type Calcium Channel Blockers on Renal Function and Aldosterone in Patients with Hypertension: A Systematic Review and Meta-Analysis
Source: PLoS One. 2014 Oct 17;9(10):e109834. doi: 10.1371/journal.pone.0109834 (PMC4201480; doi:10.1371/journal.pone.0109834)
Supplement: File S3 — PDF files of twenty-four studies included in the meta-analysis. (ZIP) [file pone.0109834.s007.zip › Supporting information-PDF files/16. Hypertens Res 2011[34(8)]935-941.pdf]

Hypertension Research (2011) 34, 935–941
© 2011 The Japanese Society of Hypertension All rights reserved 0916-9636/11
www.nature.com/hr

ORIGINAL ARTICLE

Additive antioxidative effects of azelnidipine on angiotensin receptor blocker olmesartan treatment for type 2 diabetic patients with albuminuria

Masanori Abe[1], Noriaki Maruyama[1], Kazuyoshi Okada[1], Shiro Matsumoto[1], Koichi Matsumoto[1] and Masayoshi Soma[1,2]

The present study aimed to determine whether either of two calcium channel blockers affected urinary albumin excretion or urinary levels of 8-hydroxy-2′-deoxyguanosine (8-OHdG) and liver-type fatty acid-binding protein (L-FABP) in hypertensive diabetic patients with chronic kidney disease (CKD) who were already being treated with maximum doses of the angiotensin II receptor blocker olmesartan. We conducted an open-label, randomized, parallel-controlled study on type 2 diabetic patients with stable glycemic control who were receiving fixed doses of antidiabetic agents. The patients received either 8 mg per day azelnidipine, which was increased up to 16 mg per day (azelnidipine group; n=34), or 2.5 mg per day amlodipine, which was increased up to 5 mg per day (amlodipine group; n=33), over a 24-week period. Mean systolic and diastolic blood pressure decreased significantly in both groups, but there was no significant difference between the two groups at the end of the study. Serum creatinine levels and estimated glomerular filtration rate did not differ significantly between the two groups, whereas the urinary albumin/creatinine ratio and 8-OHdG and L-FABP levels decreased significantly in the azelnidipine group compared with the amlodipine group. Plasma aldosterone level was significantly decreased in the azelnidipine group, and its changes correlated significantly with those of urinary 8-OHdG and L-FABP. Our results suggest that the addition of azelnidipine to the maximal recommended dose of olmesartan was more effective in reducing albuminuria and oxidant stress in hypertensive diabetic patients with CKD than the addition of amlodipine.
Hypertension Research (2011) **34,** 935–941; doi:10.1038/hr.2011.67; published online 9 June 2011

Keywords: aldosterone; calcium channel blocker; chronic kidney disease; diabetic nephropathy; oxidant stress

INTRODUCTION

The importance of strict blood pressure (BP) control in patients with chronic kidney disease (CKD) was emphasized by the Japanese Society of Hypertension recommendations of a target BP of $<$130/80 mm Hg for hypertensive patients with CKD. This target is further reduced to $<$125/75 mm Hg for diabetic nephropathy patients whose urinary protein excretion is $>$1 g per day.[1] Reduction in proteinuria using suitable therapeutic interventions, similar to renin–angiotensin system (RAS) blockade an antihypertensive treatment regimen, is associated with a slower decline in renal function compared with other treatment groups with similar BPs.[2,3] RAS blockade with angiotensin-converting enzyme inhibitors or angiotensin II type-1 receptor blockers (ARBs) is currently considered the most effective pharmacological approach for renoprotection. These agents reduce proteinuria more effectively than other antihypertensive drugs, and therefore, RAS inhibitors should be titrated to maximal recommended doses.[1,4] However, it is difficult to control BP with monotherapy, especially in patients with CKD, thus highlighting the need for combination drug therapy.[5]

Glomerular and tubular damage resulting from type 2 diabetes occurs over several years, and it is possible that the excretion of glomerular and tubular proteins precedes the development of microalbuminuria.[6] In patients with diabetic nephropathy, changes in proximal tubuli are observed in the development of progressive diabetic kidney disease, and renal function and prognosis show a higher correlation with structural lesions in the tubulointerstitium than with glomerular changes.[7] Furthermore, inflammation and oxidative stress are known to have an important roles in pathogenesis of diabetic hypertensive organ damages.[8] When advanced glycation end-products, shear stresses and angiotensin II stimulate their respective receptors, NADPH oxidase activity is enhanced and production of oxidative stress is increased.[8,9] Urinary liver-type fatty acid-binding protein (L-FABP) is an important marker of tubulointerstitial changes in diabetic nephropathy, whereas oxidative stress is a pathogenetic

[1]Division of Nephrology, Hypertension and Endocrinology, Department of Internal Medicine, Nihon University School of Medicine, Tokyo, Japan and [2]Division of General Medicine, Department of Internal Medicine, Nihon University School of Medicine, Tokyo, Japan
Correspondence: Dr M Abe, Division of Nephrology, Hypertension and Endocrinology, Department of Internal Medicine, Nihon University School of Medicine, 30-1, Oyaguchi Kami-chou, Itabashi-ku, Tokyo 173-8610, Japan.
E-mail: abe.masanori@nihon-u.ac.jp
Received 11 January 2011; revised 4 March 2011; accepted 13 March 2011; published online 9 June 2011

factor underlying diabetes complications such as nephropathy.[10] For this reason, 8-hydroxy-2′-deoxyguanosine (8-OHdG) has been reported to serve as a sensitive biomarker of oxidative DNA damage *in vivo*, demonstrating significantly increased urinary excretion levels with severe tubulointerstitial damage.[11,12] In addition, aldosterone induces oxidative stress in vascular cells through NADPH oxidase activation, which has a central role in endothelial dysfunction and atherosclerotic vascular disease.[13]

Recently, some calcium channel blockers (CCBs) have been demonstrated to exhibit an organ protection effect that is independent of their antihypertensive action. Azelnidipine attenuates angiotensin II-induced peritubular ischemia and has antioxidative properties that may be involved in its beneficial effect on renal injury.[14] Although it has been reported that azelnidipine decreases not only urinary albumin excretion but also urinary 8-OHdG and L-FABP levels in diabetic nephropathy,[15,16] the dosage and kinds of RAS inhibitors were not fixed and the relationship between the changes in those urinary markers and the changes in plasma aldosterone levels are not well known. Therefore, the objective of this study was to determine the effects of additional therapy from the CCBs azelnidipine or amlodipine using the maximum recommended dose of ARB, 40 mg olmesartan, on BP control and renoprotection in hypertensive diabetic patients with CKD.

METHODS

Study design

The study was conducted with outpatients at the Nihon University Nerima Hikarigaoka Hospital, Japan, who fulfilled the criteria given below. The study protocol was approved by the local ethics committee and all patients gave their written informed consent for participation. The study was conducted in accordance with the Declaration of Helsinki.

An overview of the study design is shown in Figure 1. An independent investigator, who did not treat and had no knowledge of the subjects, monitored randomized subject entry and subjects were then randomly split into two groups; assignment particulars were then immediately delivered to individual investigators. The randomization method was modified by dynamic balancing of the serum creatinine (sCr), estimated glomerular filtration rate (eGFR), and urinary albumin/creatinine (Cr) ratio values recorded at the time of patient registration.

After initial evaluation, one patient group (*n*=34) received 8 mg per day azelnidipine, which was increased up to 16 mg per day (azelnidipine group), while the second group (*n*=33) received 2.5 mg per day amlodipine, which was increased up to 5 mg per day (amlodipine group), over the 24-week period.

Study population

A total of 67 patients were enrolled in the study, and enrollment criteria were as follows.

(1) Type 2 diabetes with nephropathy under stable glycemic control. Stable glycemic control was defined as a hemoglobin A1c (HbA1c) level of $<$7.0% after administration of oral antidiabetic agents or insulin injection therapy for 8 consecutive weeks.
(2) Albuminuria: urinary albumin/Cr ratio of $>30\,mg\,g^{-1}$ (average of two consecutive measurements recorded during the 8-week pretreatment period).
(3) eGFR levels between 90 and $30\,ml\,min^{-1}$ per $1.73\,m^2$.
(4) Hypertension: systolic and diastolic BP $\geqslant$130/80 mm Hg recorded while the patient was in a sitting position during at least two independent clinic visits.
(5) Treatment with 40 mg olmesartan once daily for at least 8 weeks before the study.

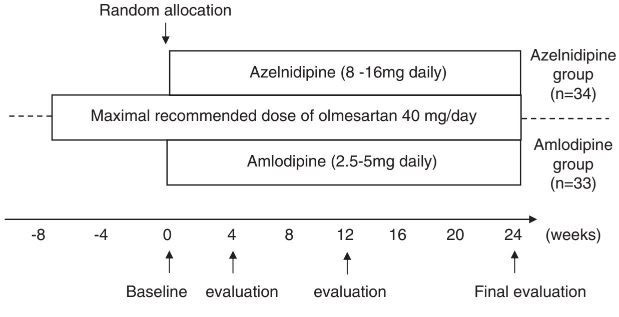

Figure 1 Study design.

Exclusion criteria were as follows.

(1) Age $<$20 years or $>$80 years.
(2) More than second-degree hypertension ($>$160/100 mm Hg).[1]
(3) Massive albuminuria: urinary albumin/Cr ratio $>2000\,mg\,g^{-1}$ (average of two consecutive measurements recorded during the 8-week pretreatment period).
(4) eGFR $>90\,ml\,min^{-1}$ per $1.73\,m^2$ or $<30\,ml\,min^{-1}$ per $1.73\,m^2$.
(5) Severe heart failure, angina, myocardial infarction or stroke occurring within 6 months from the start of the trial.
(6) Diabetes with unstable glycemic control. Unstable glycemic control was defined as an HbA1c $>$7.0% after administration of antidiabetic agents for 8 consecutive weeks.

Interventions

Doses of other antihypertensive agents and statins were maintained during the study. BP measurement was carried out at the outpatient clinic according to Japanese Society of Hypertension 2009 guidelines at fixed times after administration of medications.[1] The target BP was $<$130/80 mm Hg. Measurements were made using a sphygmomanometer (Nippon Colin, Tokyo, Japan) and were performed twice with the patient in a sitting position after a 5-min rest. Patients were given dietary guidance, especially those under dietary restrictions.

Biochemical and urinary assessments

On each patient visit, safety variables and patient compliance with treatment regimens were assessed. Laboratory tests, including those for measuring sCr, fasting plasma glucose, HbA1c, hemoglobin, aspartate aminotransferase, alanine aminotransferase, total cholesterol, low-density lipoprotein-cholesterol, triglyceride, sodium, potassium and uric acid levels, were performed using commercial kits using routinely used clinical chemistry procedures.

High-sensitivity C-reactive protein (CRP) was measured by latex agglutination. Urinary 8-OHdG (8-OHdG Check, Japan Institute for the Control of Aging, Shizuoka, Japan) and L-FABP (CMIC Co. Ltd., Tokyo, Japan) were measured at baseline before treatment and at week 24 by a specific enzyme-linked immunosorbent assay in the first morning urine sample, and the values were expressed as a ratio to the urinary Cr concentration. Urinary albumin excretion was assessed by measuring urinary concentrations of albumin and Cr (albumin/Cr ratio) in the first morning urine sample. Urinary albumins were measured using the immunoturbidimetric assay. Plasma renin activity and aldosterone concentrations were measured by radioimmunoassay at a contact laboratory (SRL, Tokyo, Japan) at baseline and at the end of the study in the supine position after a 20-min rest.

On each visit, safety variables and patient compliance with the treatment regimens were assessed. Glomerular filtration rate was estimated using the final recommended modified equation for Japanese patients provided by the Japanese Society of Nephrology–Chronic Kidney Disease Initiatives, because the estimated value obtained by this method is more accurate for Japanese patients with CKD.[17] The eGFR was calculated using the following formula:

$$\mathrm{eGFR}(\mathrm{ml\ min^{-1}\ per\ 1.73m^2}) = 194\times \mathrm{sCr}^{-1.094}\times \mathrm{age}^{-0.287}(\times 0.739\ \mathrm{for\ women}).$$

Statistical analysis

All values were expressed as mean ± s.e.m. We used the unpaired *t*-test or Fisher's exact test to compare baseline characteristics of patients in the

azelnidipine and amlodipine groups. Differences between the two groups with regard to changes in systolic and diastolic BPs were evaluated by repeated-measures analysis of variance according to a general linear model. If the analysis of variance revealed a significant overall difference, *a priori*-defined pairwise comparisons were performed using the Student's *t*-test without adjustment for multiple tests. Intragroup changes in sCr values and the urinary albumin/Cr ratio were analyzed using the paired *t*-test. The unpaired *t*-test was used to compare sCr values and the urinary albumin/Cr ratio between the azelnidipine and amlodipine groups. Correlations were determined by the Spearman rank correlation test. Statistical significance was set at $P<0.05$.

RESULTS

Baseline characteristics

All enrolled patients (n=67) remained in the study until the end of the trial. Baseline characteristics and initial medication are shown in Table 1. There were no significant differences between the two groups with regard to baseline characteristics.

BP-lowering effect

The final doses of azelnidipine and amlodipine were 13.9 ± 0.6 and 4.0 ± 0.2 mg per day, respectively, in the two groups. Figure 2 shows the changes in systolic and diastolic BP. In both groups, systolic and diastolic BP values were significantly lower than baseline values 1 month after the initiation of CCB therapy. The values did not differ significantly between the two groups during the course of treatment. In the last month of treatment, no significant (NS) differences were observed between systolic BP (azelnidipine group: 125.7 ± 1.3 mm Hg; amlodipine group: 124.3 ± 1.5 mm Hg) and diastolic BP (azelnidipine group: 74.7 ± 1.6 mm Hg; amlodipine group: 73.3 ± 1.6 mm Hg) in the two groups.

Table 1 Baseline characteristics and medications of the patients enrolled in the study

Variables	*Azelnidipine group*	*Amlodipine group*	P *value*
n	34	33	
Age (years)	65.8 ± 1.7	66.0 ± 1.4	NS
Male/female	21/13	20/13	NS
BMI (kg m^{-2})	23.9 ± 0.6	23.8 ± 0.6	NS
Systolic BP (mm Hg)	141.0 ± 1.1	140.4 ± 0.9	NS
Diastolic BP (mm Hg)	81.8 ± 1.6	81.5 ± 1.6	NS
Serum Cr (mg dl^{-1})	1.05 ± 0.06	1.07 ± 0.06	NS
eGFR (ml min^{-1} per 1.73 m^2)	54.7 ± 2.6	52.9 ± 2.7	NS
Urinary albumin/Cr ratio (mg g^{-1} Cr)	387 ± 85	380 ± 71	NS
Medications (n)			
Antihypertensive agents			
Diuretics	8	7	NS
α-Blockers	4	3	NS
β-Blockers	5	5	NS
Antidiabetic agents			
Glimepiride	5	6	NS
Nateglinide	1	2	NS
Mitiglinide	6	5	NS
Metformin	3	4	NS
Voglibose	7	8	NS
Pioglitazone	3	3	NS
Insulin	12	12	NS
Statins	13	12	NS

Abbreviations: BMI, body mass index; BP, blood pressure; Cr, creatinine; eGFR, estimated glomerular filtration rate; NS, not significant.

The BP target value (130/80 mm Hg) was achieved in 78.8 and 81.3% of all subjects in the azelnidipine and amlodipine groups (NS), respectively. The heart rate of participants at the end of the study was significantly reduced in the azelnidipine group compared with baseline (azelnidipine group: from 74.8 ± 1.7 to 73.4 ± 1.6 beats per min, $P<0.0001$; amlodipine group: from 76.8 ± 1.6 to 76.2 ± 1.6 beats per min, NS), but there was no significant difference between the two groups at the end of the trial.

Anti-albuminuric effects

As shown in Figure 3, sCr levels were not significantly different between the two groups by the end of the study (azelnidipine group: 1.04 ± 0.06 mg dl^{-1}; amlodipine group: 1.06 ± 0.05 mg dl^{-1}, NS). eGFR levels were also not significantly different between the two groups by the end of the study (azelnidipine group: 55.2 ± 2.7 ml min^{-1} per 1.73 m^2; amlodipine group: 52.6 ± 2.5 ml min^{-1} per 1.73 m^2, NS).

As shown in Figure 4, there were significant differences in the urinary albumin/Cr ratio between the two groups at the end of study (azelnidipine group: 260 ± 54 mg g^{-1} Cr; amlodipine group: 352 ± 68 mg g^{-1} Cr, $P<0.05$). Figure 4 shows that the percent changes from the baseline value of urinary albumin/Cr ratio recorded for the azelnidipine group was significantly lower from the 4-week time point to the last week of treatment compared with amlodipine group. In contrast, the amlodipine group showed no significant change in this ratio by the end of the study.

Urinary 8-OHdG and L-FABP

As shown in Figure 5, urinary 8-OHdG levels significantly decreased from 13.1 ± 0.9 to 11.0 ± 0.9 ng mg^{-1} Cr after 24 weeks in the azelnidipine group ($P<0.01$). Furthermore, there were significant differences in urinary 8-OHdG levels between the two groups at the end of the study (azelnidipine group: 11.0 ± 0.9 ng mg^{-1} Cr; amlodipine group: 13.7 ± 0.9 ng mg^{-1} Cr, $P<0.05$). Urinary L-FABP levels also significantly decreased from 43.0 ± 5.0 to 27.6 ± 2.6 ng mg^{-1} Cr after 24 weeks in the azelnidipine group ($P<0.0001$), and there were significant differences in these levels between the two groups at the end of the study (azelnidipine group: 27.6 ± 2.6 ng mg^{-1} Cr; amlodipine group: 42.1 ± 3.8 ng mg^{-1} Cr, $P<0.05$). On the other hand, the amlodipine group showed little difference in urinary levels of 8-OHdG and L-FABP during the study period (Figure 5). Although the percent changes in urinary 8-OHdG levels were significantly correlated with the percent changes of L-FABP levels (r=0.44, $P<0.01$) in the azelnidipine group, no significant correlations were found between the percent changes in urinary albumin/Cr ratio and the percent changes in urinary 8-OHdG and L-FABP levels.

Plasma renin activity and aldosterone levels

As shown in Figure 6, plasma renin activity was not significantly changed in either group after 24 weeks of treatment, and the difference at the end of the study between the two groups was not significant (azelnidipine group: from 3.52 ± 0.64 to 3.11 ± 0.51 ng ml^{-1} per h amlodipine group: from 2.83 ± 0.54 to 2.90 ± 0.50 ng ml^{-1} per h, NS). Plasma aldosterone levels were not significantly changed in the amlodipine group. However, plasma aldosterone levels were decreased in the azelnidipine group (from 91.9 ± 6.2 to 74.1 ± 6.3 pg ml^{-1}, $P<0.01$). In addition, there was a significant correlation between the percent reduction in plasma aldosterone and percent changes in urinary L-FABP levels (r=0.520, P=0.0011) and urinary 8-OHdG levels (r=0.380, P=0.0235) in the azelnidipine group.

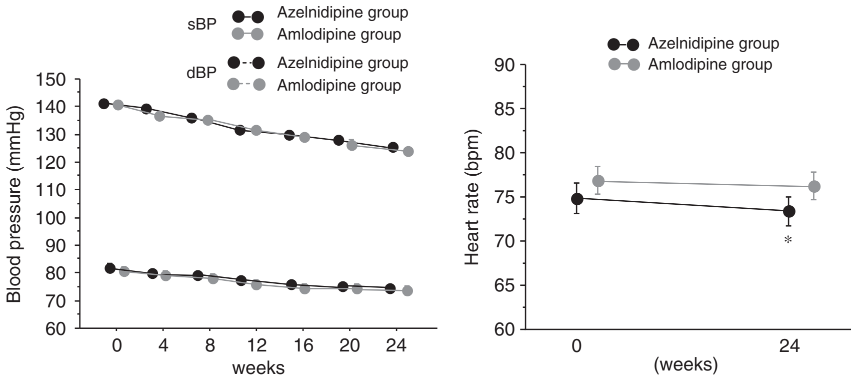

Figure 2 Changes in blood pressure and heart rate during the study period. *$P<0.0001$ *vs.* baseline. dBP, diastolic blood pressure; sBP, systolic blood pressure.

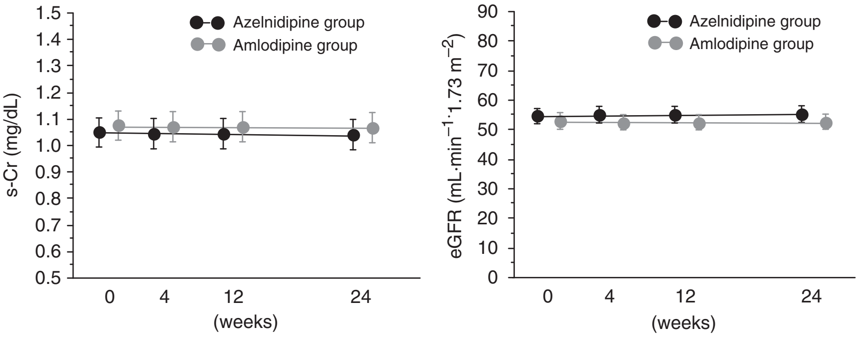

Figure 3 Changes in serum creatinine levels and the estimated glomerular filtration rate (eGFR) between the two groups. sCr, serum creatinine.

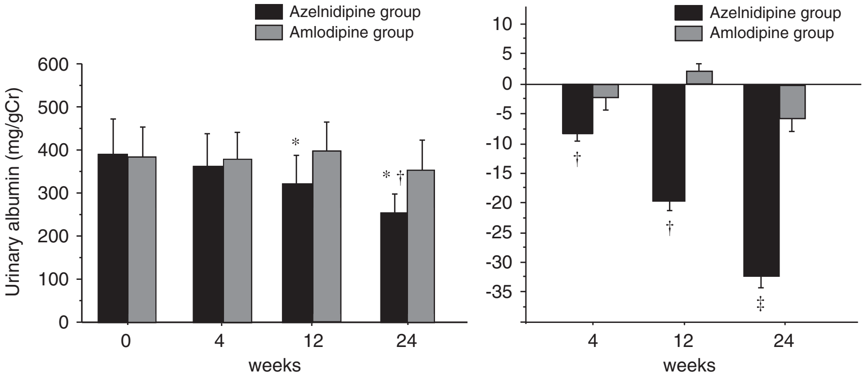

Figure 4 Changes in the urinary albumin/creatinine (Cr) ratio and the corresponding percent changes from the baseline. *$P<0.01$ *vs.* baseline, †$P<0.05$, ‡$P<0.001$ *vs.* amlodipine group.

Biochemical parameters

As listed in Table 2, fasting plasma glucose levels and HbA1c levels did not differ significantly between the two groups during the study period. There were also no significant changes in total cholesterol, low-density lipoprotein cholesterol, triglyceride, electrolyte or uric acid levels in either group. However, high-sensitivity CRP levels were significantly reduced in the azelnidipine group and there was a significant difference compared with the amlodipine group at the end of the study.

Adverse events

During the observation period, none of the patients in either group exhibited a significant increase in the occurrence of adverse effects, such as hypotension, hyperglycemia, liver impairment, anemia or skin rash, and none of the patients required renal replacement therapy for hyperkalemia or progressive loss of renal function.

DISCUSSION

The presence and severity of tubulointerstitial injury is often associated with rapid progression to end-stage renal disease. Various pathophysiological stresses on the proximal tubules such as high proteinuria induce upregulation of human L-FABP gene expression resulting in increased proximal tubular and urinary excretion of L-FABP.[18] Severe proteinuria or ischemia can also exacerbate tubulointerstitial damage by causing an overload of free fatty acids in the proximal tubules.[18] Urinary L-FABP excretion was recently shown to

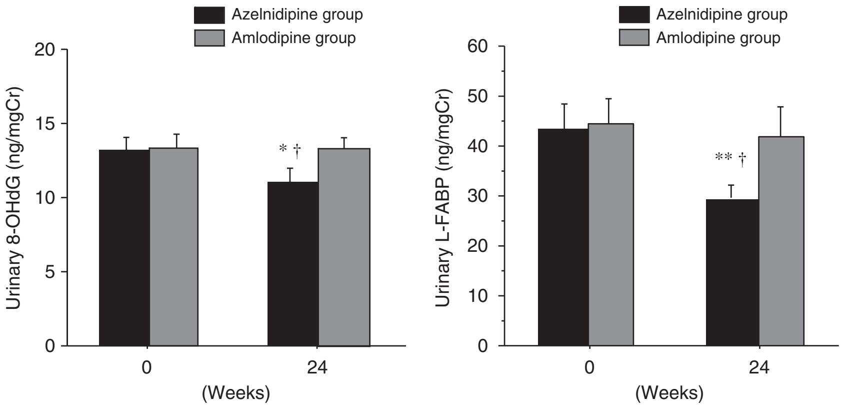

Figure 5 Changes in urinary 8-hydroxy-2′-deoxyguanosine (8-OHdG) and liver-type fatty acid-binding protein (L-FABP) levels between the two groups. *$P<0.01$, **$P<0.0001$ *vs.* baseline, †$P<0.05$ *vs.* amlodipine group.

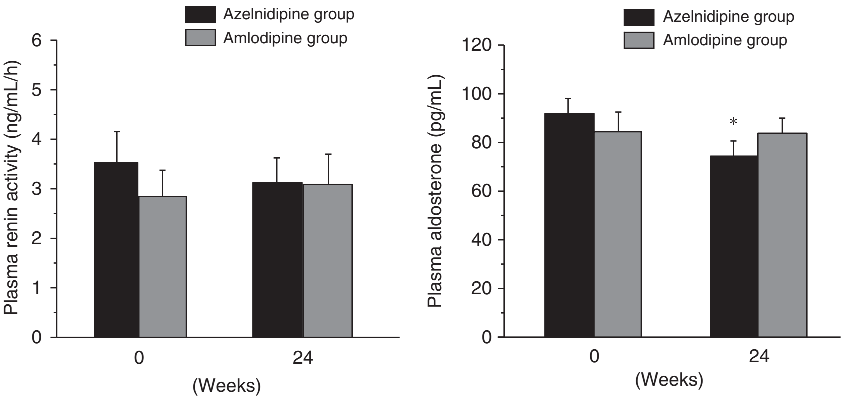

Figure 6 Changes in plasma renin activity and aldosterone levels between the two groups. *$P<0.01$ *vs.* baseline.

Table 2 Changes in the biochemical measurements during the study period

	Azelnidipine group		*Amlodipine group*	
Variables	*At baseline*	*At end*	*At baseline*	*At end*
Fasting plasma glucose (mg dl^{-1})	115±6	112±10	119±7	115±7
Hemoglobin A1c (%)	6.6±0.1	6.6±0.1	6.6±0.1	6.6±0.1
Total cholesterol (mg dl^{-1})	200±7	197±7	199±7	197±6
LDL-cholesterol (mg dl^{-1})	116±4	113±4	115±4	112±4
Triglyceride (mg dl^{-1})	161±14	158±17	163±11	162±16
Na (mEq l^{-1})	140.4±0.3	140.2±0.3	139.8±0.4	139.5±0.4
K (mEq l^{-1})	4.4±0.1	4.4±0.1	4.4±0.1	4.5±0.1
Uric acid (mg dl^{-1})	6.6±0.2	6.7±0.2	6.6±0.2	6.7±0.2
Serum albumin (g dl^{-1})	3.9±0.1	3.9±0.1	3.9±0.1	3.9±0.1
High-sensitivity CRP (mg l^{-1})	0.91±0.12	0.64±0.10*†	0.91±0.11	0.92±0.10

Abbreviations: CRP, C-reactive protein; K, potassium; LDL, low-density lipoprotein; Na, sodium.
*$P<0.01$, *vs.* baseline.
†$P<0.05$, *vs.* amlodipine group.

be a prognostic marker for the deterioration of renal function in patients with chronic glomerulonephritis, and represents a potential clinical biomarker for the monitoring and prediction of CKD progression.[18,19]

Increased oxidative stress induced by hyperglycemia may contribute to the pathogenesis of diabetic complications including nephropathy.[20] Diabetic hyperglycemia is associated with increased production of reactive oxygen species.[20] Reactive oxygen species damage to DNA necessitates the induction of various DNA repair processes, which can result in the urinary excretion of products such as 8-OHdG.[21] Urinary 8-OHdG is therefore a sensitive biomarker of oxidative DNA damage, and is also significantly correlated with the severity of tubulointerstitial lesions.[11] Increased urinary 8-OHdG and the risk of vascular complications may be present at early stages of diabetes.[22] In the present study, we showed that CCBs effectively reduce BP and that azelnidipine is more effective than amlodipine in protecting against glomerular and tubulointerstitial injury and in reducing oxidative stress in diabetic patients with CKD.

Accumulating experimental and clinical evidence show that excess aldosterone not only promotes the retention of sodium and body fluid but also induces cardiac and renal injury causing cardiac hypertrophy, inflammation and fibrosis, glomerulosclerosis, and renal inflammation and fibrosis.[23–25] Moreover, aldosterone induces oxidative stress in vascular cells through NADPH oxidase activation, which has a central role in endothelial dysfunction and atherosclerotic vascular disease.[13,26] Therefore, targeting aldosterone synthesis and release may be clinically important in preventing cardiovascular disease. Ca^{2+} ions are transported via T-type calcium channels to mitochondria, where they activate aldosterone synthesis, which in turn stimulates T-type calcium channel expression,[27,28] creating a positive feedback loop of aldosterone biosynthesis in adrenal cells. Azelnidipine was recently shown to provide not only L-type but also T-type calcium channel-blocking activity.[29] T-type CCBs including benidipine and efonidipine are beneficial for the treatment of renal injury by dilating afferent and efferent arteries; therefore, hypoxic or ischemic tubulointerstitial damage might be improved by the hemodynamic effects of such CCBs.[30–32] Furthermore, T-type CCBs prevent renal Rho kinase activation induced by subtotal nephrectomy and alleviate the progression of kidney disease.[33] As Rho kinase is known to elicit the inflammatory process and the enhancement of vascular tone as well as leading to tubulointerstitial fibrosis, the blockade of T-type calcium channels is expected to help prevent CKD.[34]

In the present study, there was a significant correlation between the changes in plasma aldosterone levels and the changes in urinary 8-OHdG and L-FABP levels. On the other hand, there was no correlation between the changes in urinary albumin excretion, which enhanced intraglomerular hypertension, and plasma aldosterone, urinary 8-OHdG and L-FABP levels, which enhanced tubulointerstitial damage. Therefore, azelnidipine treatment improved not only glomerular hemodynamics but also tubulointerstitial injury, and those efficacies might be independent. However, further studies are needed to clarify whether azelnidipine treatment mediates the reduction of urinary 8-OHdG and L-FABP, which are clinical biomarkers of tubulointerstitial damage, via prevention of aldosterone-induced NADPH oxidase activation and subsequent superoxide production, which cannot simply be explained by azelnidipine's antihypertensive effect.

T-type calcium channels are distributed in the cardiac sinus node and are closely associated with pacemaking potentials.[35,36] Therefore, in the present study, it is conceivable that the observed significant decrease in patient heart rate following treatment was indeed induced by azelnidipine. However, further studies are needed to clarify the full effect of azelnidipine on patient heart rate.

In addition to their beneficial effects on lowering BP, ARBs exhibit anti-atherogenic effects by blocking angiotensin II, and this in turn inhibits oxidative stress, inflammation, vasoconstriction and thrombosis.[37–39] ARBs, particularly telmisartan and olmesartan, also exert antidiabetic effects through their regulation of insulin sensitivity and reduce not only urinary albumin excretion but also urinary 8-OHdG and L-FABP excretion.[40] Statins have been shown to ameliorate tubular and podocyte injury, preserve GFR and reduce proteinuria in renal disease.[41,42] In the present study, although all subjects were already treated with the maximal recommended dose of the ARB olmesartan, and there was no significant difference in the number of patients treated by statins at baseline between the two groups, further reduction in high-sensitivity CRP, urinary 8-OHdG and L-FABP levels was obtained. This suggests that additive azelnidipine therapy may be beneficial in patients with albuminuria or higher levels of urinary 8-OHdG and L-FABP excretion.

The present study suffered from the limitations of small sample size and short treatment duration. Moreover, changes in sCr levels were too small for adequate evaluation of the influence of CCB therapy. Additional long-term investigations are therefore required to accurately assess the renoprotective effect of azelnidipine and olmesartan combination therapy in diabetic patients.

In conclusion, the present study showed that azelnidipine results in a greater reduction of albuminuria, plasma aldosterone, urinary 8-OHdG and L-FABP levels than amlodipine in patients with diabetic nephropathy. These effects of azelnidipine would appear to make the drug more advantageous in terms of the progression of renal dysfunction and preventing cardiovascular tissue and organ injuries in patients with hypertensive patients with diabetic nephropathy. We propose that azelnidipine therapy should be considered as an additive therapeutic modality for hypertensive diabetic patients whose BP is not sufficiently controlled by RAS-inhibition therapy with the maximal recommended dose of ARBs. Azelnidipine may ameliorate tubulointerstitial injury in diabetic hypertensive patients with CKD.

CONFLICT OF INTEREST

The authors declare no conflict of interest.

1 Japanese Society of Hypertension. Japanese Society of Hypertension Guidelines for the Management of Hypertension (JSH 2009). *Hypertension Res* 2009; **32**: 4–107.
2 Lewis EJ, Hunsicker LG, Bain RP, Rohde RD. The effect of angiotensin-converting-enzyme inhibition on diabetic nephropathy. The Collaborative Study Group. *N Eng J Med* 1993; **329**: 1456–1462.
3 Brenner BM, Cooper MD, De Zeeuw D, Keane WF, Mitch WE, Parving HH, Remuzzi G, Snapinn SM, Zhang Z, Shahinfar S. Effects of losartan on renal and cardiovascular outcomes in patients with type 2 diabetes and nephropathy. *N Eng J Med* 2001; **345**: 861–869.
4 Lavermann GD, Henning RH, De Jong PE, Navis G, De Zeeuw D. Optimal antiproteinuric dose of losartan in nondiabetic patients with nephrotic range proteinuria. *Am J Kidney Dis* 2001; **38**: 1381–1384.
5 Waeber B. Managing hypertension in high-risk patients: lessons and promises from the STRATHE and ADVANCE trials. *J Hypertens* 2006; **24**: 19–27.
6 Otsu HH, Can H, Spentzos D, Nelson RG, Hanson RL, Looker HC, Knowler WC, Montroy M, Libermann TA, Karumanchi SA, Thadhani R. Prediction of diabetic nephropathy using urine proteomic profiling 10 years prior to development of nephropathy. *Diabetes Care* 2007; **30**: 638–643.
7 Thomas MC, Burns WC, Cooper ME. Tubular changes in early diabetic nephropathy. *Adv Chronic Kid Dis* 2005; **12**: 177–186.
8 Brownlee M. Biochemistry and molecular cell biology of diabetic complications. *Nature* 2001; **414**: 818–820.
9 Sheetz MJ, King GL. Molecular understanding of hyperglycemia's adverse effects for diabetic complications. *JAMA* 2002; **288**: 2579–2588.
10 Nakamura T, Sugaya T, Kawagoe Y, Ueda Y, Osada S, Koide H. Effect of pitavastatin on urinary liver-type fatty acid-binding protein levels in patients with early diabetic nephropathy. *Diabetes Care* 2005; **28**: 2728–2732.
11 Kanauchi M, Nishioka H, Hashimoto T. Oxidative DNA damage and tubulointerstitial injury in diabetic nephropathy. *Nephron* 2002; **91**: 327–329.
12 Xu GW, Yao QH, Weng QF, Su BL, Zhang X, Xiong JH. Study of urinary 8-hydroxydeoxyguanosine as a biomarker of oxidative DNA damage in diabetic nephropathy. *J Pharm Biomed Anal* 2004; **36**: 101–104.
13 Shiffrin EL. Effects of aldosterone on the vasculature. *Hypertension* 2006; **47**: 312–318.
14 Kondo N, Kiyomoto H, Yamamoto T, Miyatake A, Sun GP, Rahman M, Hitomi H, Moriwaki K, Hara T, Kimura S, Abe Y, Kohno M, Nishiyama A. Effects of calcium channel blockade on angiotensin II-induced peritubular ischemia in rats. *J Pharmacol Exp Ther* 2006; **316**: 1047–1052.
15 Nakamura T, Sugaya T, Kawagoe Y, Suzuki T, Ueda Y, Koide T, Node K. Azelnidipine reduces urinary protein excretion and urinary liver-type fatty acid binding protein in patients with hypertensive chronic kidney disease. *Am J Med Sci* 2007; **333**: 321–326.
16 Ogawa S, Mori T, Nako K, Ito S. Combination therapy with renin-angiotensin system inhibitors and the calcium channel blocker azelnidipine decreases plasma inflammatory markers and urinary oxidative stress markers in patients with diabetic nephropathy. *Hypertens Res* 2008; **31**: 1147–1155.
17 Matsuo S, Imai E, Horio M, Yasuda Y, Tomita K, Nitta K, Yamagata K, Tomino Y, Yokoyama H, Hishida A. Revised equations for estimated GFR from serum creatinine in Japan. *Am J Kidney Dis* 2009; **53**: 982–992.

18 Kamijo A, Kimura K, Sugaya T, Yamanouchi M, Hikawa A, Hirano N, Hirata Y, Goto A, Omata M. Urinary fatty acid-binding protein as a new clinical marker of the progression of chronic renal disease. *J Lab Clin Med* 2004; **143**: 23–30.
19 Kamijo A, Sugaya T, Hikawa A, Yamanouchi M, Hirata Y, Ishimitsu T, Numabe A, Takagi M, Hayakawa H, Tabei F, Sugimoto T, Mise N, Kimura K. Clinical evaluation of urinary excretion of liver-type fatty acid-binding protein as a marker for monitoring of chronic kidney disease: a multicenter trial. *J Lab Clin Med* 2005; **145**: 125–133.
20 Goodarzi MT, Navidi AA, Rezaei M, Rezaei HB. Oxidative damage to DNA and lipids: correlation with protein glycation in patients with type 1 diabetes. *J Clin Lab Anal* 2010; **24**: 71–76.
21 Kasai H. Analysis of a form of oxidative DNA damage 8-hydroxy-2′-deoxyguanosine as a marker of cellular oxidative stress during carcinogenesis. *Mutat Res* 1997; **387**: 146–163.
22 Hata I, Kaji M, Hirano S, Shigematsu Y, Tsukahara H, Mayumi M. Urinary oxidative stress markers in young patients with type 1 diabetes. *Pediatr Int* 2006; **48**: 58–61.
23 Sun Y, Zhang J, Lu L, Chen SS, Quinn MT, Weber KT. Aldosterone-induc inflammation in the rat heart: role of oxidative stress. *Am J Pathol* 2002; **161**: 1773–1781.
24 Struthers AD, MacDonald TM. Review of aldosterone- and angiotensin II induced target organ damage and prevention. *Cardiovasc Res* 2004; **61**: 663–670.
25 Yoshida K, Kim-Mitsuyama S, Wake R, Izumi Y, Yukimura T, Ueda M, Yoshiyama M, Iwao H. Excess aldosterone under normal salt diet induces cardiac hypertrophy and infiltration via oxidative stress. *Hypertens Res* 2005; **28**: 447–455.
26 Miyata K, Rahman M, Shokoji T, Nagai Y, Zhang GX, Sun GP, Kimura S, Yukimura T, Kiyomoto H, Kohno M, Abe Y, Nishiyama A. Aldosterone stimulates reactive oxygen species production through activation of NADPH oxidase in rat mesangial cells. *J Am Soc Nephrol* 2005; **16**: 2906–2912.
27 Rossier MF, Lesouhaitier O, Perrier E, Bockhorn L, Chiappe A, Lalevee N. Aldosterone regulation of T-type calcium channels. *J Steroid Biochem Mol Biol* 2003; **85**: 383–388.
28 Laleve N, Rebsamen MC, Barrere-Lemaire S, Perrier E, Nargeot J, Benitah JP, Rossier MF. Aldosterone increases T-type calcium channel expression and *in vitro* beating frequency in neonatal rat cardiomyocytes. *Cardiovasc Res* 2005; **67**: 216–224.
29 Furukawa T, Nukada T, Namiki Y, Mayashita Y, Hatsuno K, Ueno Y, Yamakawa T, Isshiki T. Five different profiles of dihydropyridines in blocking T-type Ca^{2+} channel subtypes ($Ca_v3.1$ (α_{1G}), Ca3.2 (α_{1H}), and Ca3.3 (α_{1I})) expressed in Xenopus oocytes. *Eur J Pharmacol* 2009; **613**: 100–107.
30 Hayashi K, Wakino S, Sugano N, Ozawa Y, Homma K, Saruta T. Ca^{2+} channel subtypes and pharmacology in the kidney. *Circ Res* 2007; **100**: 342–353.
31 Abe M, Okada K, Maruyama T, Maruyama N, Matsumoto K. Comparison of the antiproteinuric effects of the calcium channel blockers benidipine and amlodipine administered in combination with angiotensin receptor blockers to hypertensive patients with stage 3-5 chronic kidney disease. *Hypertens Res* 2009; **32**: 270–275.
32 Ishimitsu T, Kameda T, Akashiba A, Takahashi T, Ohta S, Yoshii M, Minami J, Ono H, Numabe A, Matsuoka H. Efonidipine reduces proteinuria and plasma aldosterone in patients with chronic glomerulonephritis. *Hypertens Res* 2007; **30**: 621–626.
33 Sugano N, Wakino S, Kanda T, Tatematsu S, Homma K, Yoshioka K, Hasegawa K, Hara Y, Suetsugu Y, Yoshizawa T, Hara Y, Utsunomiya Y, Tokudome G, Hosoya T, Saruta T, Hayashi K. T-type calcium channel blockade as a therapeutic strategy against injury in rats with subtotal nephrectomy. *Kidney Int* 2008; **73**: 826–834.
34 Zhao D, Pothoulakis C. Rho GTPases as therapeutic targets for the treatment of inflammatory diseases. *Expert Opin Ther Targets* 2003; **7**: 583–592.
35 Perez-Reyes E. Molecular physiology of low-voltage-activated T-type calcium channels. *Physiol Rev* 2003; **83**: 117–161.
36 Nitta Y, Yamamoto R, Yamaguchi Y, Katsuda S, Kaku B, Taguchi T, Takabatake S, Nakahama K, Yamagisi M. Impact of long-acting calcium channel blockers on the prognosis of patients with coronary artery disease with and without chronic kidney disease: a comparison of three drugs. *J Int Med Res* 2010; **38**: 253–265.
37 Manotham K, Tanaka T, Matsumoto M, Ohse T, Miyata T, Inagi R, Kurokawa K, Fujita T, Nangaku M. Evidence of tubular hypoxia in the early phase in the remnant kidney model. *J Am Soc Nephrol* 2004; **15**: 1277–1288.
38 Fliser D, Wagner KK, Loos A, Tsikas D, Haller H. Chronic angiotensin II receptor blockade reduces (intra)renal vascular resistance in patients with type 2 diabetes. *J Am Soc Nephrol* 2005; **16**: 1135–1140.
39 Dzau VJ. Theodore Cooper Lecture: tissue angiotesin and pathobiology of vascular disease: a unifying hypothesis. *Hypertension* 2001; **37**: 1047–1052.
40 Nakayama S, Watada H, Mita T, Ikeda F, Shimizu T, Uchino H, Fujitani Y, Hirose Y, Kawamori R. Comparison of effects of olmesartan and telmisartan on blood pressure and metabolic parameters in Japanese early-stage type-2 diabetics with hypertension. *Hypertens Res* 2008; **31**: 7–13.
41 Shibata S, Nagase M, Fujita T. Fluvastatin ameliorates podocyte injury in proteinuria rats via modulation of excessive Rho signaling. *J Am Soc Nephrol* 2006; **17**: 754–764.
42 Agarwal R. Effects of statins on renal function. *Am J Cardiol* 2006; **97**: 748–755.
