# Supplementary material for: Effects of T-Type Calcium Channel Blockers on Renal Function and Aldosterone in Patients with Hypertension: A Systematic Review and Meta-Analysis
Source: PLoS One. 2014 Oct 17;9(10):e109834. doi: 10.1371/journal.pone.0109834 (PMC4201480; doi:10.1371/journal.pone.0109834)
Supplement: File S3 — PDF files of twenty-four studies included in the meta-analysis. (ZIP) [file pone.0109834.s007.zip › Supporting information-PDF files/17. Int Heart J 2010[51(3)]188-192.pdf]

Effects of Efonidipine, an L- and T-type Calcium Channel Blocker, on the Renin-Angiotensin-Aldosterone System in Chronic Hemodialysis Patients

Nobuyuki Nakano,[1] MD, Toshihiko Ishimitsu,[1] MD, Toshiaki Takahashi,[1] MD, Hideki Inada,[1] MD, Atsushi Okamura,[2] MD, Shuichi Ohba,[3] MD, *and* Hiroaki Matsuoka,[2] MD

Summary

Components of the renin-angiotensin-aldosterone system such as angiotensin II and aldosterone are believed to contribute to the development and progression of cardiovascular tissue and organ injuries. We compared the effects of two calcium channel blockers, efonidipine and amlodipine, on the renin-angiotensin-aldosterone system in patients with end-stage renal diseases on maintenance hemodialysis. Twenty hypertensive patients on chronic hemodialysis were given efonidipine 20-60 mg twice daily and amlodipine 2.5-7.5 mg once daily for 12 weeks each in a random crossover manner. The average blood pressure was comparable between the efonidipine and amlodipine periods (151 ± 15/77 ± 8 versus 153 ± 15/76 ± 8 mmHg). The pulse rate did not change significantly during the administration periods. Although the plasma renin activity and plasma angiotensin II were not significantly different between the efonidipine and amlodipine periods, plasma aldosterone was significantly lower in the efonidipine period than in the amlodipine period (123 ± 118 versus 146 ± 150 pg/mL, P = 0.027). The findings suggest that efonidipine reduces plasma aldosterone levels in patients on maintenance hemodialysis, and this seems to be an additional benefit to the cardiovascular protection by antihypertensive therapy with efonidipine in patients with end-stage renal disease. (Int Heart J 2010; 51: 188-192)

Key words: Efonidipine, Hypertension, Renal failure, Hemodialysis, Aldosterone

Patients with end-stage renal disease (ESRD) undergoing dialysis therapy have increased morbidity and mortality as compared with the general population. The high incidences of cardiovascular diseases and infection in particular limit the life expectancy of dialysis patients.[1,2] In order to effectively prevent cardiovascular diseases in ESRD patients, not only traditional cardiovascular risk factors such as hypertension, diabetes, and dyslipidemia, but also nontraditional risk factors should be considered.[3] Such nontraditional risk factors include neurohumoral factors such as activation of the sympathetic nervous system and renin-angiotensin-aldosterone system (RAAS). Recent studies have shown that angiotensin II and aldosterone exhibit deleterious effects on the development and progression of tissue injuries in cardiovascular organs.[4-6] In addition, it has been shown that the circulating RAAS is not necessarily suppressed in patients on chronic hemodialysis.[7]

Renal failure patients have a high prevalence of hypertension,[8-10] a major traditional cardiovascular risk factor, and calcium channel blockers (CCB) are widely used as antihypertensive drugs because of their potent hypotensive effects. CCB principally block L-type calcium channels of vascular smooth muscle, and have not been thought to directly affect RAAS. However, unlike other dihydropyridine CCB, efonidipine is known to block not only L-type but also T-type calcium channels, and recent *in vitro* and *in vivo* studies have indicated that efonidipine suppresses aldosterone secretion from the adrenal.[11-13] In this study, efonidipine, an L- and T-type CCB, and amlodipine, an L-type CCB, were given to chronic hemodialysis patients and their effects on the RAAS were compared.

Methods

A total of 20 hypertensive patients with end-stage renal diseases undergoing stable maintenance hemodialysis as an outpatient for more than 3 months were enrolled as subjects. Hypertension was defined as systolic blood pressure ≥ 140 mmHg and/or diastolic blood pressure ≥ 90 mmHg. Patients taking antihypertensive drugs, including calcium channel blockers (CCB), were also eligible for the study even if their blood pressure was below 140/90 mmHg. After a 1-week run-in period, each patient underwent a 12-week treatment with efonidipine and a 12-week treatment period with amlodipine. The order of the study drug administration was randomized blindly. The CCB given until then was stopped, however, other antihypertensive medication was not changed throughout the study periods. Efonidipine 20-

From the [1] Department of Hypertension and Cardiorenal Medicine, Dokkyo Medical University, [2] Utsunomiya Chuoh Hospital, and [3] Ohba Clinic, Tochigi, Japan.

Address for correspondence: Toshihiko Ishimitsu, MD, Department of Hypertension and Cardiorenal Medicine, Dokkyo Medical University, Mibu, Tochigi 321-0293, Japan.

Received for publication December 9, 2009.

Revised and accepted February 15, 2010.

60 mg was given twice daily after breakfast and supper, and amlodipine 2.5-7.5 mg was given once daily after breakfast. The doses of efonidipine and amlodipine were titrated so that the blood pressure was reduced to below 140 mmHg in systole and below 90 mmHg in diastole.

Blood pressure was measured before each dialysis session with the patients in a supine position after resting more than 10 minutes and the average value during one week was used for evaluation at each time point.[14,15] Body weight gains between dialysis sessions were also averaged for one week. Blood samples were obtained at the beginning and end of each treatment period.

Peripheral blood samples were obtained before starting a dialysis session after more than 15 minutes of supine rest. In addition to routine hematological tests and blood chemistry, circulating components of the renin-angiotensin system were evaluated. Plasma renin activity and plasma concentrations of aldosterone were determined by respective radioimmunoassays. Plasma angiotensin II was directly radioimmunoassayed using an Angiotensin II RIA kit (SRL Inc., Tokyo).

The study protocol was designed in accordance with the recommendations of the World Medical Association for biomedical research involving human subjects (Edinburgh version, 2000) and was approved by the institutional review board. Informed consent was obtained from all subjects.

Values are expressed as the mean ± SD. Clinical data between the two periods were compared by paired *t* test. The antihypertensive effects of drug treatments during the study periods were analyzed using ANOVA for repeated measures followed by Tukey's method for post-hoc multiple comparisons. Correlations between two parametric variables were analyzed by linear regression analysis. A *P* value less than 0.05 was considered to indicate statistical significance.

Table I. Background Characteristics of the Study Subjects

Parameter	Value
Age, years	66.8 ± 10.1
Sex, men/women	11 / 9
Cause of renal failure	
Diabetic nephropathy	10
Chronic glomerulonephritis	6
Nephrosclerosis	2
Others	2
Duration of hemodialysis, years	4.1 ± 4.5
Body mass index, kg/m^2	21.0 ± 3.7
Systolic blood pressure, mmHg	161.1 ± 15.6
Diastolic blood pressure, mmHg	80.1 ± 6.7
Pulse rate, bpm	74.2 ± 4.4
Antihypertensive medication	
Calcium channel blocker	10
Angiotensin II receptor blocker	10
Diuretic	8
α -Blocker	1
β -Blocker	1
Complication of cardiovascular disease	
Coronary heart disease	4
Valvular heart disease	2
Cerebral infarction	2
Arteriosclerotic obliteration	2

Data are mean ± SD.

Results

All 20 patients enrolled completed both administration periods without withdrawing. Table I shows the background characteristics of these patients. Diabetic nephropathy and chronic glomerulonephritis were the most frequent causes of renal failure, 50% and 30%, respectively. Average systolic but not diastolic blood pressure was in a hypertensive range before starting the study periods. Sixteen patients (80%) had already been given antihypertensive drugs such as CCB (50%), angiotensin II receptor antagonists (50%), and diuretics (40%). Six patients were on monotherapy, however, 7 patients were given 2 antihypertensive drugs and 3 were given 3 or more drugs. Ten patients had cardiovascular disease complications (Table I).

Figure 1 depicts the changes in predialysis blood pressure and pulse rate during the efonidipine and amlodipine periods. Both systolic and diastolic blood pressures were significantly lowered by efonidipine and amlodipine, except that the diastolic blood pressure reduction did not reach statistical significance at 4-weeks in the amlodipine period. The blood pressure reductions were comparable between the efonidipine and amlodipine periods during the 12-week periods. The pulse rate was not significantly affected either by efonidipine or amlodipine throughout the study periods.

Table II shows the changes in parameters of body fluid volume. Neither the dry weight setting nor cardiothoracic ratio on chest roentgenograms varied significantly during the efonidipine and amlodipine periods. The body weight increase did not change between the dialysis sessions throughout the study periods. Administration of erythropoietin for the treatment of renal anemia was expected to raise blood pressure, however, the dose was not significantly altered during the study.

Table III lists the laboratory data of the patients at the end of the efonidipine and amlodipine periods. The blood cell counts did not significantly differ between the efonidipine and amlodipine periods. There were no significant differences in any of the blood chemistry data such as serum proteins, electrolytes, and liver enzymes between the 2 periods.

Figure 2 shows the circulating components of the RAAS at the end of each treatment period. The plasma concentration of aldosterone was significantly lower by 16% in the efonidipine period than in the amlodipine period, although plasma renin activity and plasma angiotensin II concentration did not differ significantly between the 2 periods. Fig-

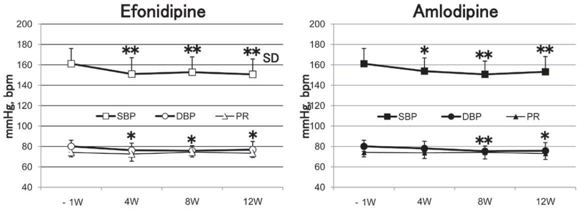

Figure 1. Time-course changes in systolic blood pressure (SBP), diastolic blood pressure (DBP), and pulse rate (PR) in hemodialysis patients during the efonidipine and amlodipine administration periods. SD: standard deviation, $P < 0.05$, ** $P < 0.001$ versus the run-in period (-1W).

Table II. Changes in Body Weight, Cardiothoracic Ratio and Erythropoietin Dose During the Study Period

Parameter	-1W	Efonidipine			Amlodipine		
		4W	8W	12W	4W	8W	12W
Dry weight, kg	52.5 ± 13.4	52.4 ± 13.6	52.3 ± 13.5	52.3 ± 13.4	52.4 ± 13.5	52.2 ± 13.8	52.3 ± 13.5
Interdialysis weight gain, %	4.5 ± 1.5	4.7 ± 1.8	4.8 ± 1.9	4.9 ± 2.0	4.4 ± 1.7	4.6 ± 1.7	4.7 ± 1.7
Cardiothoracic ratio, %	47.8 ± 4.0	48.6 ± 2.5	47.2 ± 2.2	47.7 ± 2.7	46.8 ± 2.8	47.5 ± 3.6	48.7 ± 3.5
Erythropoietin dose, U/week	3938 ± 2744	3750 ± 2725	3788 ± 2614	3713 ± 2635	3488 ± 2859	3413 ± 2459	3713 ± 2293

Data are mean ± SD.

Table III. Laboratory Findings at the End of Each Treatment Period

Parameter	Efonidipine	Amlodipine
White blood cells, × 10^3/mm^3	5.71 ± 1.83	6.30 ± 2.19
Red blood cells, × 10^6/mm^3	3.14 ± 0.41	3.21 ± 0.37
Blood hemoglobin, g/dL	9.86 ± 1.34	9.99 ± 1.02
Hematocrit, %	30.1 ± 0.5	30.7 ± 0.9
Platelet count, × 10^3/mm^3	188 ± 59	196 ± 57
Blood chemistry		
total protein, g/dL	6.6 ± 0.5	6.4 ± 0.2
albumin, g/dL	3.8 ± 0.2	3.8 ± 0.2
AST, U/L	16 ± 9	15 ± 8
ALT, U/L	13 ± 9	14 ± 8
urea nitrogen, mg/dL	72 ± 15	71 ± 17
creatinine, mg/dL	10.3 ± 1.9	10.5 ± 2.0
uric acid, mg/dL	6.8 ± 1.4	7.3 ± 1.5
Na, mEq/L	138 ± 2	138 ± 3
K, mEq/L	5.1 ± 0.7	5.0 ± 0.7
Ca, mg/dL	9.2 ± 0.8	9.5 ± 0.9
P, mg/dL	5.7 ± 0.9	5.9 ± 1.5

Data are mean ± SD. AST indicates aspartate aminotransferase and ALT, alanine aminotransferase.

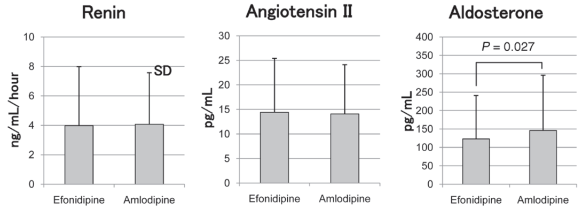

Figure 2. Parameters of renin-angiotensin-aldosterone system in plasma at the end of the efonidipine and amlodipine treatment periods. SD: standard deviation.

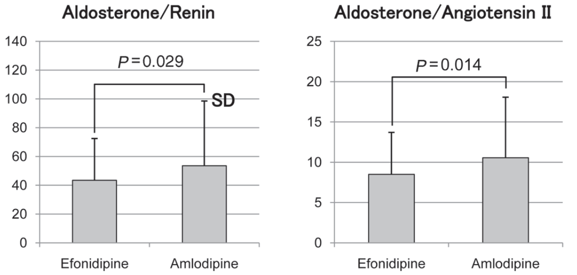

Figure 3. Aldosterone to renin and aldosterone to angiotensin II ratios in plasma at the end of the efonidipine and amlodipine treatment periods. SD: standard deviation.

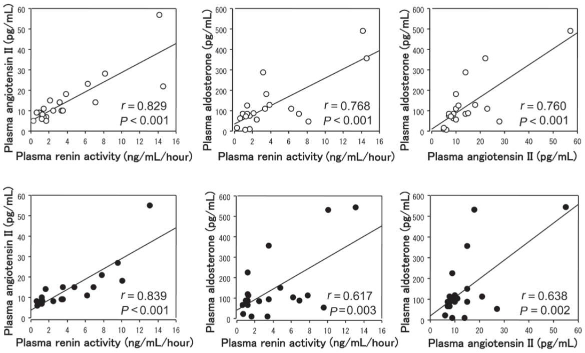

Figure 4. Correlations between parameters of renin-angiotensin-aldosterone system in plasma at the end of the efonidipine (upper panels) and amlodipine (lower panels) treatment periods.

ure 3 compares the aldosterone to renin and the aldosterone to angiotensin II ratios in plasma between the efonidipine and amlodipine periods. The former was lower by 19% and the latter by 20% in the efonidiopine period than in the amlodipine period.

Figure 4 depicts the relations between the plasma components of the RAAS in the efonidipine and amlodipine periods. In both treatment periods, close correlations were observed between renin, angiotensin II, and aldosterone in plasma.

Discussion

Hemodialysis patients are at high risk for developing cardiovascular diseases. In our prospective study following 553 chronic hemodialysis patients for 5 years, 37% patients had suffered the onset of cardiovascular events and 47% of the causes of death were cardiovascular diseases such as stroke and heart failure.[16] In addition to the traditional cardiovascular risk factors such as hypertension and diabetes, recent research has revealed that the RAAS is involved in the process of remodeling and injuries of cardiovascular tissues and organs. Components of the RAAS such as angiotensin II and aldosterone promote hypertrophy of cardiovascular cells and fibrosis of the cardiovascular tissues.[4-6]

With regard to the RAAS in chronic hemodialysis patients, the increase in body fluid volume is expected to

suppress the RAAS in ESRD. However, it seems that the plasma components of RAAS are not necessarily suppressed but rather are often increased in patients undergoing maintenance hemodialysis.[7] It is speculated that the increased angiotensin II and aldosterone may participate in the progression of cardiovascular tissue injuries in hemodialysis patients. In this context, we have previously shown that left ventricular mass is positively correlated with plasma angiotensin II in chronic hemodialysis patients.[17] It has been also reported that left ventricular mass was correlated with plasma aldosterone in hemodialysis patients.[18,19] Furthermore, inhibitors of the RAAS such as angiotensin-converting enzyme (ACE) inhibitors, angiotensin II receptor blockers (ARB), and mineralocorticoid receptor blockers are believed to improve left ventricular hypertrophy and arterial stiffening independently of their antihypertensive effects.[20-23]

It is well recognized that left ventricular hypertrophy provides predisposition to ischemic heart disease, heart failure, and ventricular arrhythmia. In addition, left ventricular hypertrophy is associated with arteriosclerosis and the risk of stroke and mortality.[24,25] Our prospective study also showed that the risk of cardiovascular events is 2 times higher in chronic hemodialysis patients with electrocardiographic signs of left ventricular hypertrophy than in patients without cardiac hypertrophy.[26] It has been also pointed out that the measurement of pulse wave velocity, an index of arterial wall stiffening, is predictive of cardiovascular events and mortality.[27] Accordingly, it is believed that RAAS inhibitors have advantage in preventing cardiovascular events and improving the prognosis of hemodialysis patients through their protective effects against cardiovascular tissue and organ injuries in addition to their antihypertensive effects.[28-30]

On the other hand, strict control of blood pressure and body fluid volume is important in order to prevent the development and progression of cardiovascular organ injuries in hemodialysis patients.[31-34] However, it has been speculated that the blood pressure of hemodialysis patients is more dependent on the degree of a blood volume increase than RAAS activity.[35,36] Among the classes of antihypertensive drugs, CCB directly dilate vascular smooth muscle and the antihypertensive effect is not greatly influenced by the pathogenesis of hypertension. Hence, CCB are widely used in the treatment of hypertension in patients with ESRD. Conversely, CCB do not principally affect the neurohumoral factors, although sympathetic nerve activity and the RAAS would be stimulated by blood pressure reduction. However, unlike other dihydropyridine CCB, efonidipine has been shown to reduce plasma aldosterone in patients with hypertension and renal diseases as well as in healthy subjects.[13,37,38] This effect is supposedly brought about by direct inhibition of aldosterone production by adrenal cells.[12] Therefore, efonidipine is thought to offer cardiovascular protection from the deleterious effects of aldosterone by a mechanism different from that of RAAS inhibitors such as ACE inhibitors, ARB, and mineralocorticoid receptor blockers.

Cardiovascular tissues have been shown to express enzymes generating angiotensin II and aldosterone, and the tissue concentrations may reach higher than the circulating levels.[4,5,39,40] Furthermore, it has been reported that such RAAS in the cardiovascular tissues is enhanced by various stimuli in the process of organ injuries in cardiovascular diseases.[4,5,41] Therefore, in order to prevent cardiovascular organ injuries effectively, it may be important to suppress the cardiovascular tissue RAAS rather than lowering plasma angiotensin II or aldosterone.[42] Considering that efonidipine may inhibit expression of the aldosterone synthase gene, it is inferred that efonidipine is more advantageous for cardiovascular protection compared to other CCB.

In summary, the present study showed that antihypertensive treatment with efonidipine reduces plasma aldosterone in patients on maintenance hemodialysis as compared with amlodipine. This effect may be brought about by the suppression of adrenal aldosterone production. Considering that cardiovascular tissue injury is supposedly promoted by activation of the RAAS, efonidipine seems to have the advantage of preventing cardiovascular diseases in addition to its antihypertensive effect.

References

1. Collins AJ, Foley R, Herzog C, *et al*. Excerpts from the United States Renal Data System 2007 annual data report. Am J Kidney Dis 2008; 51: S1-320.
2. Japanese Society for Dialysis Therapy. An overview of dialysis treatment in Japan (as of Dec. 31, 2007). Nihon Toseki Igakkai Zasshi 2009; 42: 1-45.
3. Sarnak MJ, Levey AS, Schoolwerth AC, *et al*. Kidney disease as a risk factor for development of cardiovascular disease: a statement from the American Heart Association Councils on Kidney in Cardiovascular Disease, High Blood Pressure Research, Clinical Cardiology, and Epidemiology and Prevention. Circulation 2003; 108: 2154-69. (Review)
4. Brewster UC, Setaro JF, Perazella MA. The renin-angiotensin-aldosterone system: cardiorenal effects and implications for renal and cardiovascular disease states. Am J Med Sci 2003; 326: 15-24. (Review)
5. Struthers AD, MacDonald TM. Review of aldosterone- and angiotensin II-induced target organ damage and prevention. Cardiovasc Res 2004; 61: 663-70. (Review)
6. Diamond JA, Phillips RA. Hypertensive heart disease. Hypertens Res 2005; 28: 191-202. (Review)
7. Ishimitsu T, Matsumoto N, Seta H, Yanaka H, Matsuoka H. Implication of angiotensin II as a cardiovascular risk factor in hemodialysis patients. J Jpn Coll Angiol 2007; 47: 383-89.
8. Salem MM. Hypertension in the hemodialysis population: a survey of 649 patients. Am J Kidney Dis 1995; 26: 461-8.
9. Mittal SK, Kowalski E, Trenkle J, *et al*. Prevalence of hypertension in a hemodialysis population. Clin Nephrol 1999; 51: 77-82.
10. Grekas D, Bamichas G, Bacharaki D, Goutzaridis N, Kasimatis E, Tourkantonis A. Hypertension in chronic hemodialysis patients: current view on pathophysiology and treatment. Clin Nephrol 2000; 53: 164-8.
11. Tanaka H, Shigenobu K. Pathophysiological significance of T-type Ca2+ channels: T-type Ca2+ channels and drug development. J Pharmacol Sci 2005; 99: 214-20. (Review)
12. Imagawa K, Okayama S, Takaoka M, *et al*. Inhibitory effect of efonidipine on aldosterone synthesis and secretion in human adrenocarcinoma (H295R) cells. J Cardiovasc Pharmacol 2006; 47: 133-8.
13. Okayama S, Imagawa K, Naya N, *et al*. Blocking T-type Ca2+ channels with efonidipine decreased plasma aldosterone concentration in healthy volunteers. Hypertens Res 2006; 29: 493-

7.
14. Conion PJ, Walshe JJ, Heinle SK, Minda S, Krucoff M, Schwab SJ. Predialysis systolic blood pressure correlates strongly with mean 24-hour systolic blood pressure and left ventricular mass in stable hemodialysis patients. J Am Soc Nephrol 1996; 7: 2658-63.
15. Zoccali C, Mallamaci F, Tripepi G, *et al*. Prediction of left ventricular geometry by clinic, pre-dialysis and 24-h ambulatory BP monitoring in hemodialysis patients: CREED investigators. J Hypertens 1999; 17: 1751-8.
16. Ishimitsu T, Nakano N, Sudo Y, *et al*. Predictive significance of blood pressure values for the incidence of cardiovascular events in chronic hemodialysis patients. Hypertens Res 2008; 31: 1703-9.
17. Yanaka H, Ishimitsu T, Takahashi M, Minami J, Ono H. Analysis of factors influencing the left ventricular mass in chronic hemodialysis patients. J Jpn Soc Dial Ther 2002; 35: 1211-7.
18. Sato A, Funder JW, Saruta T. Involvement of aldosterone in left ventricular hypertrophy of patients with end-stage renal failure treated with hemodialysis. Am J Hypertens 1999; 12: 867-73.
19. Steigerwalt S, Zafar A, Mesiha N, Gardin J, Provenzano R. Role of aldosterone in left ventricular hypertrophy among African-American patients with end-stage renal disease on hemodialysis. Am J Nephrol 2007; 27: 159-63.
20. London GM, Pannier B, Guerin AP, Marchais SJ, Safar ME, Cuche JL. Cardiac hypertrophy, aortic compliance, peripheral resistance, and wave reflection in end-stage renal disease. Comparative effects of ACE inhibition and calcium channel blockade. Circulation 1994; 90: 2786-96.
21. Matsumoto N, Ishimitsu T, Okamura A, Seta H, Takahashi M, Matsuoka H. Effects of imidapril on left ventricular mass in chronic hemodialysis patients. Hypertens Res 2006; 29: 253-60.
22. Shibasaki Y, Masaki H, Nishiue T, Nishikawa M, Matsubara H, Iwasaka T. Angiotensin II type 1 receptor antagonist, losartan, causes regression of left ventricular hypertrophy in end-stage renal disease. Nephron 2002; 90: 256-61.
23. Ichihara A, Hayashi M, Kaneshiro Y, *et al*. Low doses of losartan and trandolapril improve arterial stiffness in hemodialysis patients. Am J Kidney Dis 2005; 45: 866-74.
24. Kawamura M, Fijimoto S, Hisanaga S, Yamamoto Y, Eto T. Incidence, outcome, and risk factors of cerebrovascular events in patients undergoing maintenance hemodialysis. Am J Kidney Dis 1998; 31: 991-6.
25. Silberberg JS, Barre PE, Prichard SS, Sniderman AD. Impact of left ventricular hypertrophy on survival in end-stage renal disease. Kidney Int 1989; 36: 286-90.
26. Ishimitsu T, Tsukada K, Ohta S, *et al*. Increased cardiovascular risk in long-term hemodialysis patients carrying deletion allele of ACE gene polymorphism. Am J Kidney Dis 2004; 44: 466-75.
27. Blacher J, Guerin AP, Pannier B, Marchais SJ, Safar ME, London GM. Impact of aortic stiffness on survival in end-stage renal disease. Circulation 1999; 99: 2434-9.
28. Zannad F, Kessler M, Lehert P, *et al*. Prevention of cardiovascular events in end-stage renal disease: results of a randomized trial of fosinopril and implications for future studies. Kidney Int 2006; 70: 1318-24.
29. Efrati S, Zaidenstein R, Dishy V, *et al*. ACE inhibitors and survival of hemodialysis patients. Am J Kidney Dis 2002; 40: 1023-9.
30. Suzuki H, Kanno Y, Sugahara S, *et al*. Effect of angiotensin receptor blockers on cardiovascular events in patients undergoing hemodialysis: an open-label randomized controlled trial. Am J Kidney Dis 2008; 52: 501-6.
31. Cannella G, Paoletti E, Delfino R, Peloso G, Molinari S, Traverso GB. Regression of left ventricular hypertrophy in hypertensive dialyzed uremic patients on long-term antihypertensive therapy. Kidney Int 1993; 44: 881-6.
32. Lopez-Gomez JM, Verde E, Perez-Garcia R. Blood pressure, left ventricular hypertrophy and long-term prognosis in hemodialysis patients. Kidney Int Suppl 1998; 68: S92-8. (Review)
33. Ozkahya M, Toz H, Qzerkan F, *et al*. Impact of volume control on left ventricular hypertrophy in dialysis patients. J Nephrol 2002; 15: 655-60.
34. Gunal AI, Karaca I, Aygen B, Yavuzkir M, Dogukan A, Celiker H. Strict fluid volume control and left ventricular hypertrophy in hypertensive patients on chronic haemodialysis: a cross-sectional study. J Int Med Res 2004; 32: 70-7.
35. Lins RL, Elseviers M, Rogiers P, *et al*. Importance of volume factors in dialysis related hypertension. Clin Nephrol 1997; 48: 29-33.
36. Ventura JE, Spósito M. Volume sensitivity of blood pressure in end-stage renal disease. Nephrol Dial Transplant 1997; 12: 485-91.
37. Ishimitsu T, Kameda T, Akashiba A, *et al*. Efonidipine reduces proteinuria and plasma aldosterone in patients with chronic glomerulonephritis. Hypertens Res 2007; 30: 621-6.
38. Tsutamoto T, Tanaka T, Nishiyama K, *et al*. Long-term effect of efonidipine therapy on plasma aldosterone and left ventricular mass index in patients with essential hypertension. Hypertens Res 2009; 32: 670-4.
39. Navar LG, Harrison-Bernard LM, Imig JD, Wang CT, Cervenka L, Mitchell KD. Intrarenal angiotensin II generation and renal effects of AT1 receptor blockade. J Am Soc Nephrol 1999; 10: S266-72. (Review)
40. Delcayre C, Silvestre JS. Aldosterone and the heart: towards a physiological function? Cardiovasc Res 1999; 43: 7-12. (Review)
41. Davies E, MacKenzie SM. Extra-adrenal production of corticosteroids. Clin Exp Pharmacol Physiol 2003; 30: 437-45. (Review)
42. Takai S, Jin D, Sakaguchi M, Miyazaki M. Significant target organs for hypertension and cardiac hypertrophy by angiotensin-converting enzyme inhibitors. Hypertens Res 2004; 27: 213-9.
