# Supplementary material for: Effects of T-Type Calcium Channel Blockers on Renal Function and Aldosterone in Patients with Hypertension: A Systematic Review and Meta-Analysis
Source: PLoS One. 2014 Oct 17;9(10):e109834. doi: 10.1371/journal.pone.0109834 (PMC4201480; doi:10.1371/journal.pone.0109834)
Supplement: File S3 — PDF files of twenty-four studies included in the meta-analysis. (ZIP) [file pone.0109834.s007.zip › Supporting information-PDF files/18. Am J Med Sci 2010[339(2)]157-163.pdf]

Comparative Effects of Benidipine and Amlodipine on Proteinuria, Urinary 8-OHdG, Urinary L-FABP, and Inflammatory and Atherosclerosis Markers in Early-Stage Chronic Kidney Disease

Tsukasa Nakamura, MD, Eiichi Sato, MD, Nobuharu Fujiwara, MD, Yasuhiro Kawagoe, MD, Yoshihiko Ueda, MD, Takeshi Sugaya, PhD, Shoichi Yamagishi, MD, Shingo Yamada, MD and Hikaru Koide, MD

Abstract: *Introduction:* We examined the effects of 2 calcium channel blockers, benidipine (T-, L-, and N-type) and amlodipine (L- and N-type), on renal, inflammatory, oxidative, and atherosclerosis markers in hypertensive patients with mild chronic kidney disease (CKD). *Methods:* Forty hypertensive patients with CKD were assigned randomly to either of the 2 treatments: 8 mg benidipine once daily (n = 20, group A) or 5 mg amlodipine once daily (n = 20, group B). Treatment was continued for 12 months. Blood pressure, serum creatinine, estimated glomerular filtration rate, urinary protein excretion, urinary liver-type fatty acid-binding protein, interleukin-6, high mobility group box-1 protein, urinary 8-hydroxy-2′-deoxyguanosine, pulse wave velocity, intima-media thickness, and blood asymmetric dimethylarginine were monitored. *Results:* Blood pressure decreased equally in both groups ($P < 0.001$, at 6 and 12 months versus before treatment). Serum creatinine and estimated glomerular filtration rate changed little during the experimental period in each group. However, urinary protein excretion ($P < 0.001$), urinary liver-type fatty acid-binding protein ($P < 0.001$), urinary 8-hydroxy-2′-deoxyguanosine ($P < 0.001$), blood interleukin-6 ($P < 0.001$), blood high mobility group box-1 ($P < 0.05$), and pulse wave velocity ($P < 0.01$) decreased more in group A than in group B with 12 months of treatment. The percent reductions in intima-media thickness and blood asymmetric dimethylarginine were significantly greater in group A than in group B ($P < 0.001$). *Conclusions:* Benidipine is more effective than amlodipine for protecting renal function and potentially for ameliorating atherosclerosis in hypertensive patients with mild CKD. T-type calcium channel blockers may be effective in patients with CKD.

Key Indexing Terms: Chronic kidney disease; Benidipine; Amlodipine; Liver-type fatty acid-binding protein; Atherosclerosis. **[Am J Med Sci 2010;339(2):157–163.]**

The need for management of hypertension has received considerable attention in patients with chronic kidney disease (CKD), and strict control of blood pressure has been recommended on the basis of several mega studies.[1] CKD is an independent risk factor for cardiovascular disease in patients with hypertension.[2] Inflammation, hypertension, oxidative stress, and increased pulse pressure have been associated with vascular dysfunction and may be targets for future interventional strategies to reduce cardiovascular risk in patients with CKD.[3] Proteinuria is one of the clinical factors used to diagnose renal disease, especially glomerular hypertension, and it has been reported as a risk factor for cardiovascular events.[4] Liver-type fatty acid-binding protein (L-FABP) is expressed predominantly in the proximal tubules of the human kidney. The urinary L-FABP level reflects the clinical prognosis of CKD.[5] Intima-media thickness (IMT) and pulse wave velocity (PWV) are important vascular markers in patients with CKD.[6,7] The blood asymmetric dimethylarginine (ADMA) level is increased in patients with CKD and, as such, is a strong biomarker or predictor of future cardiovascular events.[8] High mobility group box-1 (HMGB1) protein and interleukin (IL)-6 may be associated with inflammation. HMGB1 produced by activated vascular smooth muscle cells may contribute to the progression of atherosclerosis[9]; thus, blocking HMGB1 secretion may be an important strategy for treatment of cardiovascular disease.[10]

Antihypertensive therapy is used to prevent cardiovascular complications. It follows that the organ-protective effect of antihypertensive drugs is important. Angiotensin receptor blockers (ARBs) and angiotensin-converting enzyme inhibitors (ACEIs) are recommended as first-line treatments for hypertension in patients with CKD by various guidelines, including those of the Japanese Society of Hypertension (JSH 2004).[11] However, we cannot always control blood pressure with ARBs or ACEIs alone.

Amlodipine is a representative calcium channel blocker (CCB) that is used widely. It blocks L- and N-type calcium channels[12] and dilates the afferent arterioles more than the efferent arterioles.[13] Benidipine, a T-, L-, and N-type CCB, dilates both efferent and afferent arterioles and reduces glomerular pressure.[14] Recently, Abe et al[15] reported that benidipine had a greater antiproteinuric effect than that of amlodipine in hypertensive patients with stage 3 to 5 CKD. Fukumoto et al[16] reported that benidipine, in comparison with amlodipine, was associated with a lower incidence of cardiovascular events and cerebral infarction in patients with vasospastic angina. In animal models, benidipine inhibits both production of reactive oxygen species and oxidative stress, which contribute to renal injury in hypertension.[17] However, the renovascular, antiinflammatory, and antioxidative effects of amlodipine and benidipine have not been compared in hypertensive patients with CKD. We conducted a prospective study to compare the renoprotective, antiinflammatory, antiatherosclerotic, and antioxidative effects of benidipine and amlodipine in hypertensive patients with mild CKD.

From the Department of Medicine (TN, ES, NF, YK), Shinmatsudo Central General Hospital, Chiba, Japan; Department of Pathology (YU), Dokkyo University School of Medicine, Saitama, Japan; Department of Medicine (TS), St. Marianna University School of Medicine, Kanagawa, Japan; Department of Pathophysiology and Therapeutics of Diabetic Vascular Complications (S. YAMAGISHI), Kurume University School of Medicine, Kurume, Japan; Central Institute (S. YAMADA), Shino-Test Corporation, Kanagawa, Japan; and Department of Medicine (HK), Koto Hospital, Tokyo, Japan.

Submitted April 30, 2009; accepted in revised form October 6, 2009.

Correspondence: Hikaru Koide, MD, Department of Medicine, Koto Hospital, 6-8-5 Ojima, Koto-ku, Tokyo 136-0072, Japan (E-mail: hkoide@koto-hospital.or.jp).

Copyright © by the Southern Society for Clinical Investigation. Unauthorized reproduction of this article is prohibited.

PATIENTS AND METHODS

Study Design and Subjects

Forty nondiabetic patients with stage 1 to 2 CKD (22 men and 18 women; mean age, 32 ± 6 years) and hypertension (blood pressure ≥130/80 mm Hg) were enrolled in the study. CKD was diagnosed by renal biopsy. For light microscopic examination, renal biopsy specimens were stained with hematoxylin and eosin, periodic acid-Schiff, and Masson trichrome. Global glomerulosclerosis was assessed in specimens stained with periodic acid-Schiff and is presented as a percentage of the total number of glomeruli. For each patient, 10 to 30 glomeruli per specimen (median, 18 glomeruli) were inspected. The degree of interstitial fibrosis was scored semiquantitatively on Masson-stained specimens as follows: 0, no fibrosis; 1, mild fibrosis (25% fibrotic tissue); 2, moderate fibrosis (25%–50%); and 3, severe fibrosis (>50%). The exclusion criteria were as follows: age <20 years, serum creatinine level >1.5 mg/dL, urinary protein excretion >3.0 g/d (severe proteinuria), histologically confirmed global glomerulosclerosis (≥50%), κ myocardial infarction or a cerebrovascular incident within the preceding 6 months, congestive heart failure, liver disease, chronic pulmonary disease, cancer, pregnancy, and/or collagen disease. Initially, 52 nondiabetic patients with CKD were enrolled, but 12 patients were excluded because of moderately decreased renal function, severe proteinuria, and/or severe histopathologic changes. All patients with CKD had already been treated with ARBs or ACEIs per the recommendations of the JSH 2004. The 40 remaining patients with CKD with hypertension, despite treatment with ARBs or ACEIs, were assigned randomly to 1 of the 2 treatment groups in a blinded fashion. Patients in group A received 8 mg benidipine once daily (n = 20), and patients in group B received 5 mg amlodipine once daily (n = 20). These are the maximum doses recognized in Japan. Other antihypertensive agents, including α-blockers, β-blockers, and diuretics, could be added if required to achieve blood pressure <130/80 mm Hg. Other CCBs were not permitted. During treatment, all patients were advised to restrict their dietary salt intake (4–6 g/d). Clinical and laboratory findings are summarized in Table 1. Informed consent was obtained from each patient, and the ethics committee of Shinmatsudo Central General Hospital approved the study protocol. The doses of other antihypertensive agents, drugs for glomerulonephritis, and statins were held constant during the study period. Treatment was continued for 12 months, during which time clinical and laboratory values were checked once a month, and there were no changes in medications during the experimental period.

Measurement of Renal Function

Two hours after administration of the test drug, outpatient blood pressure measurements were obtained twice in the sitting position after 2 minutes of rest, and the mean of the 2 values was determined. The serum creatinine level, estimated glomerular filtration rate (eGFR), urinary protein levels, and urinary L-FABP level were determined as markers of renal function. The eGFR was calculated according to the modified Modification of Diet in Renal Disease (MDRD) Study equation [GFR = 0.741 × 175 (serum creatinine in mg/dL) − 1.154 (age) − 0.203 (0.742 if female)].[18] The mean difference between measured inulin clearance and eGFR was significantly smaller with the modified MDRD equation than with the original MDRD equation. However, eGFR values calculated by the modified equation were still underestimated in the range of inulin clearance >60 mL/min/1.73 m^2.[18]

TABLE 1. Characteristics of patients with CKD

	Benidipine (group A, n = 20)	Amlodipine (group B, n = 20)
Sex		
Male	11	11
Female	9	9
Age (yr)	33.5 ± 7.0	31.6 ± 5.3
CKD stage (n)		
1	9	10
2	11	10
Dose (mg/d)	8	5
SBP (mm Hg)	154 ± 7	153 ± 6
DBP (mm Hg)	92 ± 4	91 ± 3
Heart rate (beats/min)	70 ± 4	71 ± 3
Serum creatinine (mg/dL)	0.72 ± 0.06	0.71 ± 0.07
eGFR (mL/min)	91.2 ± 15.3	93.5 ± 10.3
Proteinuria (g/d)	1.6 ± 0.5	1.5 ± 0.3
Urinary L-FABP (μg/g crea)	39.3 ± 13.5	37.9 ± 12.6
Urinary 8-OHdG (ng/mg crea)	12.3 ± 2.7	12.9 ± 3.7
Blood IL-6 (pg/mL)	25.1 ± 6.6	25.9 ± 7.6
Blood HMGB1 (ng/mL)	2.5 ± 0.9	2.6 ± 0.8
PWV (cm/sec)	1314 ± 90	1303 ± 79
IMT (mm)	0.601 ± 0.031	0.606 ± 0.024
Blood ADMA (nmol/mL)	0.53 ± 0.10	0.52 ± 0.11
LDLC (mg/dL)	122 ± 10	120 ± 8
HDLC (mg/dL)	52 ± 8	54 ± 6
Triglyceride (mg/dL)	128 ± 12	126 ± 10
Histology		
Global GS (%)	5.0 ± 2.0	5.3 ± 3.0
TI score	0.92 ± 0.24	0.96 ± 0.32
Antihypertensive drugs (n)		
ARB	15	14
ACEI	5	6
α-blocker	4	4
β-blocker	3	4
Others	2	2
Antiglomerulonephritis drugs (n)		
Antiplatelet	14	13
Steroid	3	3
Immunosuppressant	1	1
Statins (n)	3	3
Primary disease (n)		
IgA nephropathy	14	13
Non-IgA PGN	4	5
MN	1	1
MPGN	1	1

CKD, chronic kidney disease; SBP, systolic blood pressure; DBP, diastolic blood pressure; eGFR, estimated glomerular filtration rate; L-FABP, liver-type fatty acid binding protein; 8-OHdG, 8-hydroxydeoxyguanosine; IL-6, interleukin-6; HMGB1, high mobility box protein-1; PWV, pulse wave velocity; IMT, intima-media thickness; ADMA, asymmetric dimethylarginine; LDLC, low-density lipoprotein cholesterol; HDLC, high-density lipoprotein cholesterol; GS, glomerulosclerosis; TI, tubulointerstitial; ARB, angiotensin-II receptor blocker; ACEI, angiotensin-converting enzyme inhibitor; PGN, primary glomerulonephritis; MN, membranous nephropathy; MPGN, membranoproliferative glomerulonephritis.

Copyright © by the Southern Society for Clinical Investigation. Unauthorized reproduction of this article is prohibited.

TABLE 2. Changes in blood pressure in groups A and B

	Benidipine (group A)			Amlodipine (group B)		
	Before	6 mo	12 mo	Before	6 mo	12 mo
SBP (mm Hg)	154 ± 7	135 ± 5[a]	128 ± 4[a]	153 ± 6	137 ± 5[a]	128 ± 5[a]
DBP (mm Hg)	92 ± 4	83 ± 3[a]	78 ± 3[a]	91 ± 3	84 ± 3[a]	76 ± 3[a]

Blood pressure at 6 months vs. 12 months in both groups is statistically not significant.
[a] $P < 0.001$ vs. before.
SBP, systolic blood pressure; DBP, diastolic blood pressure.

Measurement of Urinary L-FABP and 8-Hydroxy-2′-Deoxyguanosine

Urinary L-FABP was measured with a sandwich enzyme-linked immunosorbent assay (ELISA) kit per the manufacturer's protocol (CIMC, Tokyo, Japan) as reported previously.[5] When intraassay reproducibility was examined by repeated analyses (8×) of the same sample, the coefficient of variation was <10%. The sensitivity of this kit was between 4 and 400 ng/mL. Urinary 8-hydroxy-2′-deoxyguanosine (8-OHdG) as a marker of oxidative stress was measured by ELISA as reported previously.[19] We used morning samples to measure urinary 8-OHdG and urinary L-FABP. Urinary 8-OHdG levels in morning urine correlated significantly with those in 24-hour pooled urine.[20] In addition, we confirmed that urinary L-FABP levels in 24-hour pooled urine correlated significantly with those in spot urine ($r = 0.92$, $P < 0.0005$) (Sugaya, unpublished data).

Assessment of IMT and PWV

Carotid artery IMT and brachial-ankle PWV were determined as indicators of atherosclerosis. High-resolution B-mode ultrasound examination was performed with a 7.5-MHz mechanical sector transducer installed on an Aloka SSD-2000 ultrasound system (Aloka, Tokyo, Japan). An experienced technologist who was blinded to the patients' clinical data made all ultrasound measurements (scans and image analyses) as described previously.[21] Carotid IMT was measured at points 20, 25, and 30 mm proximal to the flow divider on the far walls of the right and left common carotid arteries at the end of the diastolic phase.[22] A mean value for the left carotid artery (mean of 3 values) and a mean value for the right carotid artery (mean of 3 values) were obtained, and then the larger of the 2 mean values was used as the representative common carotid artery IMT. Brachial-ankle PWV was measured with a pulse pressure analyzer (model BP-203RPE, Nihon Colin, Tokyo, Japan).

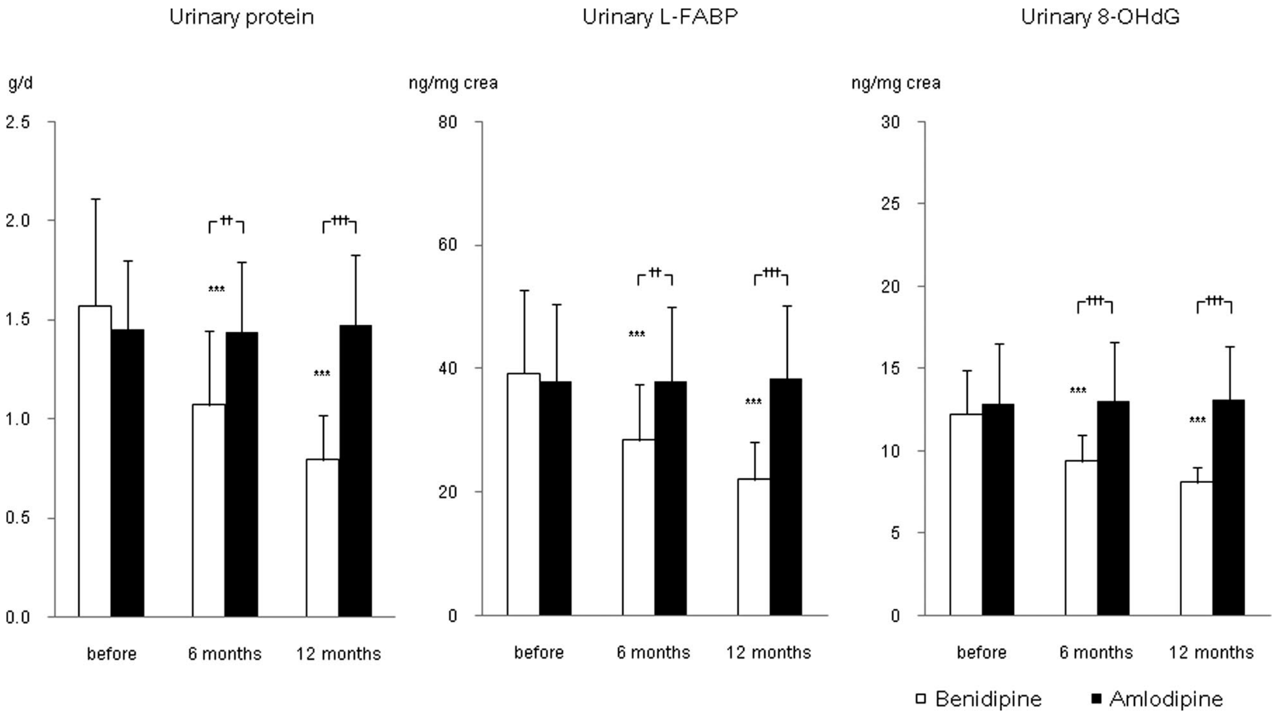

FIGURE 1. Changes in urinary protein, urinary L-FABP, and urinary 8-OHdG levels in patients with CKD treated with benidipine or amlodipine. Urinary protein, urinary L-FABP, and urinary 8-OHdG decreased significantly in patients in group A, whereas these markers changed little during the experimental period in patients in group B. L-FABP, liver-type fatty acid-binding protein; 8-OHdG, 8-hydroxydeoxyguanosine. Data are shown as mean ± SD. ***$P < 0.001$ versus before treatment, and ††$P < 0.01$ and †††$P < 0.001$ versus amlodipine.

© 2010 Lippincott Williams & Wilkins

Copyright © by the Southern Society for Clinical Investigation. Unauthorized reproduction of this article is prohibited.

Patients were examined in the supine position. Electrocardiographic electrodes were placed on both wrists: a microphone for detecting heart sounds was placed on the left edge of the sternum, and sphygmomanometer cuffs were placed around both upper arms and both ankles. Pulse waves were recorded by means of sensors placed on both posterior tibial arteries. The times required for the pulse waves to travel from the heart to both posterior tibial arteries were determined in relation to the patient's height. The 10 best consecutive pulses were analyzed, and the average PWV from the heart to the posterior tibial artery was calculated by dividing the distance by the time. PWV is expressed in cm/sec. The PWV coefficient of variation was <5%. IMT and PWV studies were performed by a single trained observer who was unaware of the clinical and biochemical data.

Measurement of Blood IL-6 and HMGB1

The blood IL-6 level was determined as a marker of inflammation. Blood IL-6 levels were measured by ELISA (Quantikine Human IL-6 Immunoassay, R&D Systems, Minneapolis, MN). This assay can detect IL-6 at a concentration as low as 7 pg/mL. The upper limit of the normal blood IL-6 level is 10 pg/mL. Blood HMGB1 levels were measured by ELISA (Shino-Test Corporation, Kanagawa, Japan) as described previously.[23] Validated interassay and intraassay coefficients of variation were <10%, and the limit of detection of this ELISA system was 0.3 ng/mL.[24] Blood concentrations of ADMA were measured by high-performance liquid chromatography as described previously.[25]

Statistical Analysis

Results are presented as mean ± standard deviation (SD). To analyze differences in values between the 2 groups and differences before and after treatment, we used the Mann-Whitney *U* test for unpaired data and the Wilcoxon signed-rank test for paired data, respectively. A P value of <0.05 was considered significant.

RESULTS

All 40 patients completed the 12-month study without experiencing adverse effects. As shown in Table 1, there were

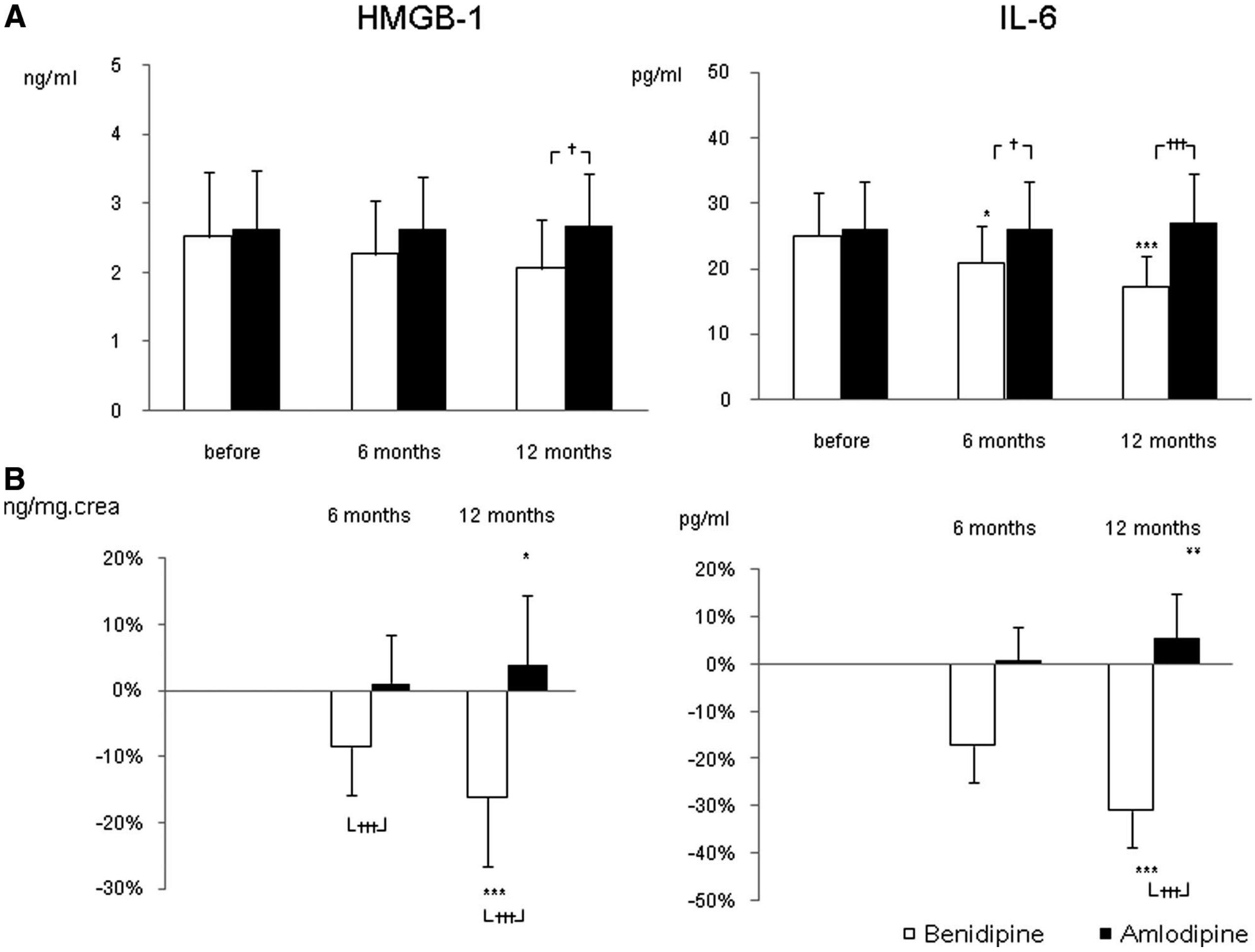

FIGURE 2. (A) Changes in blood IL-6 and HMGB1 levels in patients with CKD treated with benidipine (group A) and amlodipine (group B). (B) Percent changes in these markers. Blood IL-6 and HMGB1 levels in group B changed little during the experimental period. The percent reductions in IL-6 and HMGB1 levels were significantly greater in group A than in group B. IL-6, interleukin-6; HMGB1, high mobility group box-1 protein. Data are expressed as mean ± SD. *$P < 0.05$, ***$P < 0.001$ versus before treatment, †$P < 0.05$ and †††$P < 0.001$ versus amlodipine (A); *$P < 0.05$, **$P < 0.01$, and ***$P < 0.001$ at 6 months versus 12 months, †††$P < 0.001$ versus amlodipine (B).

Copyright © by the Southern Society for Clinical Investigation. Unauthorized reproduction of this article is prohibited.

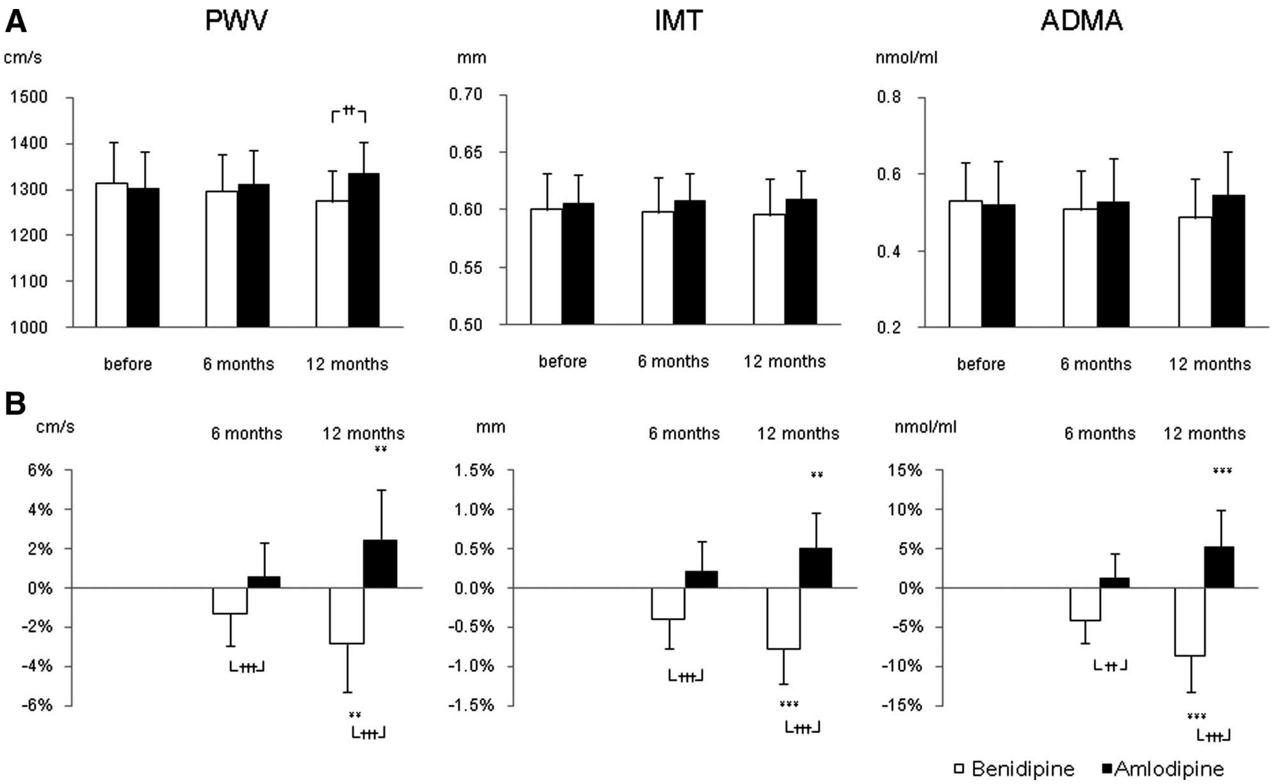

FIGURE 3. A) Changes in PWV, IMT, and blood ADMA levels in patients with CKD treated with benidipine (group A) and amlodipine (group B). (B) Percent changes in these markers. PWV, IMT, and blood ADMA levels changed little during the experimental period in group B. The percent reductions in levels of these markers were significantly greater in group A than in group B. PWV, pulse wave velocity; IMT, intima-media thickness; ADMA, asymmetric dimethylarginine. Data are shown as mean ± SD. ††$P < 0.01$ versus before treatment (A); **$P < 0.01$, ***$P < 0.001$, at 6 months versus 12 months, ††$P < 0.01$, †††$P < 0.001$ versus amlodipine (B).

no differences in baseline characteristics, including age, sex, systolic blood pressure, diastolic blood pressure, heart rate, serum creatinine, eGFR, low-density lipoprotein cholesterol, high-density lipoprotein cholesterol, triglyceride, urinary protein, urinary L-FABP, urinary 8-OHdG, IL-6, HMGB1, PWV, IMT, ADMA, and primary renal disease between the 2 treatment groups. Histopathologic variables also did not differ between the 2 treatment groups. The percentages of patients taking steroids and statins did not differ between the 2 groups. Blood pressures showed a similar gradual and significant decrease with time in both groups (at 6 and 12 months of treatment, $P < 0.001$, versus before treatment), and there was no difference in the degree of change between the 2 groups (Table 2). Serum creatinine, eGFR, low-density lipoprotein cholesterol, high-density lipoprotein cholesterol, triglyceride, and heart rate changed little during the experimental period in both groups. Changes in urinary protein, urinary L-FABP, and urinary 8-OHdG levels are shown in Figure 1. These markers changed little during the experimental period in group B, but they decreased significantly after 6 and 12 months of treatment in group A ($P < 0.001$ versus before treatment). The decrease in urinary protein, urinary L-FABP, and urinary 8-OHdG was significantly greater in group A than in group B (at 6 months: urinary protein, $P < 0.01$; L-FABP, $P < 0.01$; and 8-OHdG, $P < 0.001$. At 12 months: urinary protein, L-FABP, and 8-OHdG, $P < 0.001$). Changes in blood IL-6 and HMGB1 levels are shown in Figures 2(A) and 2(B). In group B, blood IL-6 and HMGB1 levels changed little during the experimental period, whereas in group A, blood IL-6 levels decreased significantly after 6 and 12 months of treatment (at 6 months, $P < 0.05$ and at 12 months, $P < 0.001$ versus before treatment). The percent decline in blood IL-6 was significantly greater in group A than in group B after 12 months of treatment ($P < 0.001$). Blood HMGB1 levels were also significantly higher in group B than in group A after 12 months of treatment ($P < 0.05$ versus before treatment). The percent reduction in blood HMGB1 was greater in group A than in group B at 6 and 12 months of treatment ($P < 0.001$). Changes in PWV, IMT, and blood ADMA levels are shown in Figures 3(A) and 3(B). In group B, PWV, IMT, and blood ADMA changed little during the experimental period. In group A, IMT and blood ADMA levels decreased slightly but not significantly after 6 and 12 months of treatment, whereas PWV decreased significantly ($P < 0.01$ versus before treatment). The percent reductions in PWV, IMT, and blood ADMA were significantly greater in group A than in group B (at 6 months: PWV, $P < 0.001$; IMT, $P < 0.001$; and ADMA, $P < 0.01$. At 12 months: PWV, IMT, and ADMA, $P < 0.001$).

DISCUSSION

Recently published guidelines, such as the JSH 2004,[11] recommend that blood pressure be maintained at <130/80 mm Hg in patients with CKD. Use of CCBs is not recommended as part of the initial treatment strategy; however, we cannot always control blood pressure with ARBs or ACEIs. In this study, we showed that CCBs effectively reduce blood pressure and that benidipine is more effective than amlodipine in pro-

© 2010 Lippincott Williams & Wilkins

Copyright © by the Southern Society for Clinical Investigation. Unauthorized reproduction of this article is prohibited.

tecting against glomerular and tubulointerstitial injury and in reducing oxidative stress, inflammation, and atherosclerosis in hypertensive patients with CKD.

Amlodipine is commonly used throughout the world, and it is not only cost effective, but it is predicted to save costs when compared with usual care, warranting its consideration as an agent of choice for patients with coronary artery disease.[26] Recently, Kaneshiro et al[27] reported that add-on treatment with amlodipine can have beneficial effects in nondiabetic patients with stage 1 to 2 CKD already being treated with valsartan. Das et al[28] reported that suppression of plasminogen receptors may contribute to the antiinflammatory effects of amlodipine. Kumagai et al[29] reported that the effect of amlodipine on renal function is likely the same as that of ACEIs and that amlodipine is better tolerated than ACEIs in hypertensive patients with renal dysfunction. However, Iino et al[30] reported that losartan and amlodipine were of similar efficacy for blood pressure control and that losartan significantly reduced urinary protein levels, whereas amlodipine did not change the protein levels. L-type CCBs dilate afferent arterioles, whereas ACEIs/ARBs, similar to T-type CCBs, dilate both afferent and efferent arterioles.

Benidipine dilates both efferent and afferent arterioles and reduces glomerular pressure.[14] Benidipine prevents intimal thickening in mice and has a protective effect against vascular remodeling independent of its effect on blood pressure.[31] Some investigators have reported that benidipine reduces proteinuria in patients with diabetic nephropathy,[32] whereas others have reported that proteinuria in patients with CKD does not change in response to treatment with benidipine.[14] Seino et al[32] suggested that T-type CCB could be involved in the renoprotective effect of benidipine. Recently, Ohishi et al[33] reported that benidipine may be more effective than amlodipine in reducing proteinuria. Our current data are consistent with their findings. Ishimitsu et al[34] reported that efonidipine, an L- and T-type CCB, results in a greater reduction of proteinuria than does amlodipine by a mechanism independent of blood pressure reduction in patients with chronic glomerulonephritis.

Oxidative stress is associated with exacerbation of renal injuries in hypertension,[17] and benidipine is reported to reduce oxidative stress.[17,35] In this study, we found that benidipine is more effective than amlodipine in reducing urinary 8-OHdG, a marker of oxidative stress.

Little is known about the effect of benidipine or amlodipine on regulation of urinary L-FABP, blood HMGB1, blood IL-6, and blood ADMA levels in patients with CKD. The urinary L-FABP level is a more sensitive marker than the urinary protein level for predicting progression of CKD, indicating that urinary L-FABP is a useful clinical biomarker for monitoring CKD.[5] In this study, we report that benidipine, but not amlodipine, may prevent tubulointerstitial injury in patients with CKD.

HMGB1 has unique biochemical functions as a biologically intrinsic requisite factor and as a toxin.[36] HMGB1 is increased in the blood of patients with severe sepsis and septic shock.[37] Recently, investigators have reported that blood HMGB1 is significantly increased in patients with CKD.[38,39] In this study, we found that HMGB1 levels are significantly lower in patients treated with benidipine than in those treated with amlodipine, suggesting that benidipine may have greater efficacy than amlodipine in reducing inflammation in patients with CKD.

The blood ADMA level is associated with cardiovascular risk factors such as hypertension, diabetes mellitus, and CKD and is a strong predictor of CKD.[40] ADMA may participate actively in atherogenesis in patients with CKD. One group of investigators reported a positive correlation between ADMA and proteinuria in patients with CKD.[41] Blood ADMA levels are decreased by renin-angiotensin system inhibitors,[42] whereas amlodipine does not affect ADMA levels.[43] In this study, we found that benidipine, not amlodipine, suppresses blood ADMA levels in patients with CKD. Increased IMT and PWV are associated with atherosclerosis. One group of investigators reported that amlodipine reduces IMT in hypertensive patients,[44] whereas others reported that IMT is not affected by amlodipine.[45] In addition, some reported that amlodipine reduces PWV in hypertensive patients,[45] whereas others reported that PWV is not affected by amlodipine.[46] In this study, we found that benidipine, in comparison with amlodipine, causes more significant reductions in IMT and PWV in patients with CKD.

One limitation of our study is that the dose of each drug was limited to one amount, therefore we cannot rule out the possibility that the differences we observed were primarily dose dependent. Amlodipine at 5 mg/d and benidipine at 8 mg/d are the maximal doses recognized in Japan. Further study is needed to determine whether the renovascular protection of these drugs is dose dependent.

In conclusion, amlodipine blocks L- and N-type calcium channels and dilates the afferent arterioles more than the efferent arterioles, and benidipine, a T-, L,- and N-type CCB, dilates both efferent and afferent arterioles and reduces glomerular pressure. Our study results indicate that benidipine is more effective than amlodipine in protecting renal and vascular function and protecting against inflammation in patients with early-stage CKD with hypertension.

REFERENCES

1. **Peterson JC, Adler S, Burkart JM, et al.** The Modification of Diet in Renal Disease Study: blood pressure control, proteinuria, and the progression of renal disease. Ann Intern Med 1995;123:754–62.
2. **Segura J, Campo C, Gil P, et al.** Development of chronic kidney disease and cardiovascular prognosis in essential hypertensive patients. J Am Soc Nephrol 2004;15:1616–22.
3. **Dogra G, Irish A, Chan D, et al.** Insulin resistance, inflammation, and blood pressure determine vascular dysfunction in CKD. Am J Kidney Dis 2006;48:926–34.
4. **De Leeuw PW, Thijs L, Birkenhager WH, et al.** Prognostic significance of renal function in elderly patients with isolated systolic hypertension: results from the Syst-Eur trial. J Am Soc Nephrol 2002;13: 2213–22.
5. **Kamijo A, Sugaya T, Hikawa A, et al.** Clinical evaluation of urinary excretion of liver-type fatty acid-binding protein as a marker for the monitoring of chronic kidney disease: a multicenter trial. J Lab Clin Med 2005;145:125–33.
6. **Wang MC, Tsai WC, Chen JY, et al.** Stepwise increase in arterial stiffness corresponding with the stages of chronic kidney disease. Am J Kidney Dis 2005;45:494–501.
7. **Preston E, Ellis MR, Kulinskaya E, et al.** Association between carotid artery intima-media thickness and cardiovascular risk factors in CKD. Am J Kidney Dis 2005;46:856–62.
8. **Ueda S, Yamagishi S, Kaida Y, et al.** Asymmetric dimethylarginine may be a missing link between cardiovascular disease and chronic kidney disease. Nephrology 2007;12:582–92.
9. **Inoue K, Kawahara K, Biswas KK, et al.** HMGB1 expression by activated smooth muscle cells in advanced human atherosclerosis plaques. Cardiovasc Pathol 2007;16:136–43.
10. **Porto A, Palumbo R, Pieroni M, et al.** Smooth muscle cells in human atherosclerotic plaques secrete and proliferate in response to high mobility group box-1 protein. FASEB J 2006;20:2565–6.

Copyright © by the Southern Society for Clinical Investigation. Unauthorized reproduction of this article is prohibited.

11. **Japanese Society of Hypertension Guidelines Committee for Renal Disease and Hypertension.** Guidelines for the management of hypertension (JSH 2004), Tokyo, Japan: Life Science; 2004. p. 41–4.
12. **Furukawa T, Yamakawa T, Midera T, et al.** Selectivities of dihydropyridine derivatives in blocking Ca^{2+} channel subtypes expressed in *Xenopus* oocytes. J Pharmacol Exp Ther 1999;291:464–73.
13. **Hayashi K, Nagahama T, Oka K, et al.** Disparate effects of calcium antagonists on renal microcirculation. Hypertens Res 1996;19:31–6.
14. **Morikawa T, Okumura M, Konishi Y, et al.** Effects of benidipine on glomerular hemodynamics and proteinuria in patients with nondiabetic nephropathy. Hypertens Res 2002;25:571–6.
15. **Abe M, Okada K, Maruyama T, et al.** Comparison of the antiproteinuric effects of the calcium channel blockers benidipine and amlodipine administered in combination with angiotensin receptor blockers to hypertensive patients with stage 3–5 chronic kidney disease. Hypertens Res 2009;31:270–5.
16. **Fukumoto Y, Yasuda S, Ito A, et al.** Prognostic effects of benidipine in patients with vasospastic angina: comparison with diltiazem and amlodipine. J Cardiovasc Pharmacol 2008;51:253–7.
17. **Matsubara M, Akizuki O, Ikeda J, et al.** Benidipine, an antihypertensive drug, inhibits reactive oxygen species production in polymorphonuclear leukocytes and oxidative stress in salt-loaded strokeprone spontaneously hypertensive rats. Eur J Pharmacol 2008;580:201–13.
18. **Imai E, Horio M, Nitta K, et al.** Estimation of glomerular filtration rate by the MDRD study equation modified for Japanese patients with chronic kidney disease. Clin Exp Nephrol 2007;11:41–50.
19. **Saito S, Yamauchi H, Hasui Y, et al.** Quantitative determination of urinary 8-hydroxydeoxyguanosine (8-OH-dG) by using ELISA. Res Commun Mol Pathol Pharmacol 2000;107:39–44.
20. **Miwa M, Matsumaru H, Akimoto Y, et al.** Quantitative determination of urinary 8-hydroxy-2′-deoxyguanosine level in healthy Japanese volunteers. Biofactors 2004;22:249–53.
21. **Nakamura T, Matsuda T, Kawagoe Y, et al.** Effect of pioglitazone on carotid intima-media thickness and arterial stiffness in type 2 diabetic nephropathy patients. Metabolism 2004;53:1382–6.
22. **Maeda N, Sawayama Y, Tatsukawa M, et al.** Chlamydia pneumoniae, seropositivity and early carotid atherosclerosis in a suburban Japanese population. Atherosclerosis 2002;164:313–9.
23. **Yamada S, Inoue K, Yakabe K, et al.** High mobility group protein 1 (HMGB1) quantified by ELISA with a monoclonal antibody that does not cross-react with HMGB2. Clin Chem 2003;49:1535–7.
24. **Yamada S, Yakabe K, Ishii J, et al.** New high mobility group box 1 assay system. Clin Chim Acta 2006;372:173–8.
25. **Matsuguma K, Ueda S, Yamagishi S, et al.** Molecular mechanism for elevation of asymmetric dimethylarginine and its role for hypertension in chronic kidney disease. J Am Soc Nephrol 2006;17:2176–83.
26. **de Portu S, Mantovani LG.** Amlodipine: a pharmacoeconomic review. J Med Econ 2009;12:60–8.
27. **Kaneshiro Y, Ichihara A, Sakoda M, et al.** Add-on benefits of amlodipine and thiazide in nondiabetic chronic kidney disease stage 1/2 patients treated with valsartan. Kidney Blood Press Res 2009;32:51–8.
28. **Das R, Burke T, Van Wagoner DR, et al.** L-type calcium channel blockers exert an anti-inflammatory effect by suppressing expression of plasminogen receptors on macrophages. Cir Res 2009;105:167–75.
29. **Kumagai H, Hayashi K, Kumamaru H, et al.** Amlodipine is comparable to angiotensin-converting enzyme inhibitor for long-term renoprotection in hypertensive patients with renal dysfunction: a one-year, prospective, randomized study. Am J Hypertens 2000;13:980–5.
30. **Iino Y, Hayashi M, Kawamura T, et al.** Renoprotective effect of losartan in comparison to amlodipine in patients with chronic kidney disease and hypertension —a report of the Japanese Valsartan Therapy Intended for the Global Renal Protection in Hypertensive Patients (JLIGHT) study. Hypertens Res 2004;27:21–30.
31. **Yamashita T, Kawashima S, Ozaki M, et al.** A calcium channel blocker, benidipine, inhibits intimal thickening in the carotid artery of mice by increasing nitric oxide production. J Hypertens 2001;19:451–8.
32. **Seino H, Miyaguchi S, Yamazaki T, et al.** Effect of benidipine hydrochloride, a long-acting T-type calcium channel blocker, on blood pressure and renal function in hypertensive patients with diabetes mellitus. Analysis after switching from cilnidipine to benidipine. Arzneimittelforschung 2007;57:526–31.
33. **Ohishi M, Takagi T, Ito N, et al.** Renal-protective effect of T- and L-type calcium channel blockers in hypertensive patients: an Amlodipine-to-Benidipine Changeover (ABC) study. Hypertens Res 2007;30: 797–806.
34. **Ishimitsu T, Kameda T, Akashiba A, et al.** Efonidipine reduces proteinuria and plasma aldosterone in patients with chronic glomerulonephritis. Hypertens Res 2007;30:621–6.
35. **Suzuki O, Yoshida T, Tani S, et al.** Antioxidative effects of benidipine hydrochloride in patients with hypertension independent of antihypertensive effects. Relationship between blood pressure and oxidative stress. Arzneimittelforschung 2004;54:505–12.
36. **Yamada S, Maruyama I.** HMGB1, a novel inflammatory cytokine. Clin Chim Acta 2007;375:36–42.
37. **van Zoelen MA, Laterre PF, van Veen SQ, et al.** Systemic and local high mobility group box 1 concentrations during severe infection. Crit Care Med 2007;35:2799–804.
38. **Bruchfeld A, Qureshi AR, Lindholm B, et al.** High mobility group box protein 1 correlates with renal function in chronic kidney disease (CKD). Mol Med 2008;14:109–15.
39. **Sato F, Maruyama S, Hayashi H, et al.** High mobility group box chromosomal protein 1 in patients with renal diseases. Nephron Clin Pract 2008;108:c194–201.
40. **Ueda S, Yamagishi S, Matsumoto Y, et al.** Asymmetric dimethylarginine (ADMA) is a novel emerging risk factor for cardiovascular disease and the development of renal injury in chronic kidney disease. Clin Exp Nephrol 2007;11:115–21.
41. **Caglar K, Yilmaz MI, Sonmez A, et al.** ADMA, proteinuria, and insulin resistance in non-diabetic stage 1 chronic kidney disease. Kidney Int 2006;70:781–7.
42. **Aslam S, Santha T, Leone A, et al.** Effects of amlodipine and valsartan on oxidative stress and plasma methylarginines in end-stage renal disease patients on hemodialysis. Kidney Int 2006;70:2109–15.
43. **Jepson RE, Syme HM, Vallance C, et al.** Plasma asymmetric dimethylarginine, symmetric dimethylarginine, l-arginine, and nitrite/nitrate concentrations in cats with chronic kidney disease and hypertension. J Vet Intern Med 2008;22:317–24.
44. **Terpstra WF, May JF, Smit AJ, et al.** Effects of amlodipine and lisinopril on intima-media thickness in previously untreated, elderly hypertensive patients (the ELVERA trial). J Hypertens 2004;22:1309–16.
45. **Ichihara A, Kaneshiro Y, Takemitsu T, et al.** Effects of amlodipine and valsartan on vascular damage and ambulatory blood pressure in untreated hypertensive patients. J Hum Hypertens 2006;20:787–94.
46. **Rajzer M, Klocek M, Kawecka-Jaszcz K.** Effect of amlodipine, quinapril, and losartan on pulse wave velocity and plasma collagen markers in patients with mild-to-moderate arterial hypertension. Am J Hypertens 2003;16:439–44.

© 2010 Lippincott Williams & Wilkins

Copyright © by the Southern Society for Clinical Investigation. Unauthorized reproduction of this article is prohibited.
