# Supplementary material for: Effects of T-Type Calcium Channel Blockers on Renal Function and Aldosterone in Patients with Hypertension: A Systematic Review and Meta-Analysis
Source: PLoS One. 2014 Oct 17;9(10):e109834. doi: 10.1371/journal.pone.0109834 (PMC4201480; doi:10.1371/journal.pone.0109834)
Supplement: File S3 — PDF files of twenty-four studies included in the meta-analysis. (ZIP) [file pone.0109834.s007.zip › Supporting information-PDF files/19. Hypertens Res 2009[32(8)]670-674.pdf]

Hypertension Research (2009) 32, 670–674
© 2009 The Japanese Society of Hypertension All rights reserved 0916-9636/09 $32.00
www.nature.com/hr

ORIGINAL ARTICLE

Long-term effect of efonidipine therapy on plasma aldosterone and left ventricular mass index in patients with essential hypertension

Takayoshi Tsutamoto, Toshinari Tanaka, Keizo Nishiyama, Masayuki Yamaji, Chiho Kawahara, Masanori Fujii, Takashi Yamamoto and Minoru Horie

A certain percentage of aldosterone (ALD) breakthrough generally occurs in patients with hypertension and chronic heart failure and is an important issue during long-term treatment with angiotensin-converting enzyme inhibitors (ACE-I) and angiotensin receptor blockers (ARB). It has been reported that efonidipine decreases the plasma levels of ALD. However, the long-term effects of efonidipine on the plasma levels of ALD and the left ventricular mass index (LVMI) remain unknown in patients with hypertension. Sixty stable outpatients with essential hypertension who had received amlodipine and ACE-I or ARB for more than 1 year were randomized into two groups (amlodipine group (n=30): continuous amlodipine treatment at a stable dose; efonidipine group (n=30): amlodipine (5 mg day^{-1}) was changed to efonidipine at a dose of 40 mg day^{-1}). There was no difference in their baseline characteristics including the LVMI and plasma levels of ALD. In the amlodipine group, there were no significant changes in blood pressure, LVMI or plasma levels of ALD for 18 months. In the efonidipine group, blood pressure did not change after replacement of amlodipine with efonidipine, although there was a significant decrease in the plasma levels of ALD after 6 months. The decrease in ALD was sustained for 18 months and LVMI was significantly decreased after 18 months (121 ± 25 *vs.* 114 ± 21 g m^{-2}, P<0.05). There was a significant correlation between the changes in LVMI and % changes of ALD in the efonidipine group. These findings indicate that the effect of efonidipine on the suppression of plasma ALD was sustained for at least 18 months and that long-term efonidipine therapy decreases LVMI in patients with essential hypertension.
Hypertension Research (2009) **32,** 670–674; doi:10.1038/hr.2009.78; published online 5 June 2009

Keywords: aldosterone; aldosterone breakthrough; efonidipine; left ventricular mass index

INTRODUCTION

Recent clinical trials have shown that inhibiting the biological actions of aldosterone (ALD) using an ALD blocker alone or in combination with angiotensin-converting enzyme inhibitors (ACE-I) or angiotensin receptor blockers (ARB) is reported to be a useful add-on therapy in hypertensive patients, reducing the incidence of cardiovascular events among patients with chronic heart failure (CHF).[1,2] Therefore, to prevent cardiovascular disease, targeting ALD synthesis and release may be clinically important. One of the major incentives for implementing the RALES and EPHESUS trial was the investigation of the ALD breakthrough phenomenon[3,4] in which ACE-I does not reliably induce significant decreases in plasma ALD levels. Therefore, in a certain percentage of patients with hypertension or CHF, ALD breakthrough generally occurs. This phenomenon is an important issue during a long-term treatment with ACE-I and ARB.[1,5–11] Although the mechanisms of ALD breakthrough remain obscure, there was no apparent reduction of the left ventricular mass index (LVMI) in patients with ALD breakthrough(+) during the administration of ACE-I.[8]

Aldosterone production in the adrenal gland is mediated mainly by the T-type calcium channel *in vitro*.[12] Recently, it has been reported that efonidipine, a T-type and L-type calcium channel blocker, significantly decreases the plasma levels of ALD.[13,14] However, the long-term effects of efonidipine on the plasma levels of ALD and LVMI remain unknown in patients with hypertension.

This study evaluated the long-term effects of efonidipine after the replacement of amlodipine on the plasma levels of ALD and LVMI in patients with essential hypertension.

METHODS

Sixty stable outpatients with essential hypertension who had received anti-hypertension drugs including amlodipine and ACE-I or ARB for more than 1 year were randomized into two groups (control group (n=30): continuous amlodipine treatment at a stable dose; efonidipine group (n=30): amlodipine (5 mg day^{-1}) was changed to efonidipine at a dose of 40 mg/day, which is a comparable antihypertensive dose). Patients with renal insufficiency (serum creatinine >1.5 mg per 100 ml, angina pectoris or moderate-to-severe CHF

Department of Cardiovascular and Respiratory Medicine, Shiga University of Medical Science, Tsukinowa, Seta, Otsu, Japan
Correspondence: Dr T Tsutamoto, Department of Cardiovascular and Respiratory Medicine, Shiga University of Medical Science Tsukinowa, Seta, Otsu 520-2192, Japan.
E-mail: tutamoto@belle.shiga-med.ac.jp
Received 25 February 2009; revised 25 March 2009; accepted 14 April 2009; published online 5 June 2009

(New York Heart Association functional class III or IV) were excluded from this study. Although the use of other drugs was allowed, the doses of these agents were not changed during the study period. The general condition of each patient had been stable for more than 6 months before the study.

In the outpatient clinic, resting heart rate was determined from ECG and blood pressure measurements and data were independently confirmed by attending physicians. Blood samples were collected from the antecubital vein after resting in a seated position for at least 20 min at baseline, after 6 months, after 12 months and after 18 months. Echocardiography was performed at baseline and after 18 months. Left ventricular ejection fraction was measured by echocardiography at the same time. LVMI was calculated from M-mode ECGs according to the formula by Devereux *et al.*[15]

Blood samples were assessed for serum potassium and serum creatinine, for plasma renin concentration, plasma levels of ALD and for brain natriuretic peptide (BNP). ALD breakthrough was defined as an increased value compared with the baseline value. The attending physicians were blinded to the neurohumoral and echocardiographic data. Informed consent was obtained from all patients for participation in the study, following protocol approval by the Committee on Human Investigation at our institution.

Measurement of neurohumoral factors

Blood samples were collected from the antecubital vein after resting in a seated position for at least 20 min. Blood was centrifuged at 3000 r.p.m for 15 min at 4 °C, and the plasma thus obtained was stored at −30 °C until assay. The plasma levels of ALD and plasma renin concentration were measured using the commercial radioimmunoassay kits as previously reported.[14] The samples for the assay of plasma BNP concentrations were transferred to chilled disposable tubes containing aprotinin (500 kallikrein inactivator U ml^{-1}). The blood samples were immediately placed on ice and centrifuged at 4 °C, and then the plasma was frozen in aliquots and stored at −30°C until assay. Plasma BNP concentrations were measured with a specific immunoradiometric assay for human BNP using a commercial kit (Shionogi, Osaka, Japan) as previously reported.[16]

Statistical analysis

All results are expressed as the mean ± s.d. Univariate analysis was performed using Student's *t*-test. Categorical data were compared against χ^2 distribution. Comparisons between groups were performed by analysis of variance with Scheffe's test for continuous variables. Analysis of variance was used to determine the significance of differences between groups. A *P*-value <0.05 was regarded as significant.

RESULTS

Table 1 lists the characteristics of the participants. The participants were 60 patients with essential hypertension. There was no difference in baseline characteristics including left ventricular ejection fraction and LVMI and plasma levels of ALD and BNP between the two groups (Table 1). Nineteen patients had CHF (NYHA class I or II) and there was no difference in the incidence of CHF complication between the two groups. Concomitant therapy other than with amlodipine and ACE-I or ARB was maintained for at least 12 months and there was no difference in baseline medication between the two groups.

In both the control and efonidipine groups, there were no significant changes in blood pressure over the18-month observation period (Table 2). In the efonidipine group, the heart rate decreased after 6 months and the decrease in heart rate was sustained during the 18 months (Table 2).

In the control group, plasma levels of ALD and BNP did not change during the 18 months but serum creatinine was significantly increased after 6 months. In the efonidipine group, plasma levels of ALD decreased after 6 months and the decrease in ALD was sustained for 18 months (106 ± 49 pg ml^{-1} at baseline, 76 ± 30 pg ml^{-1} at 6 months, 79 ± 43 pg ml^{-1} at 12 months, 77 ± 34 pg ml^{-1} at 18 months) (Figure 1); the plasma ALD level eventually increased again in two patients (7% ALD breakthrough). In the efonidipine group, the plasma levels of BNP did not change during the 18 months and serum creatinine was significantly increased after 6 months, although LVMI was significantly decreased after 18 months (121 ± 25 *vs.* 114 ± 21 g m^{-2}, $P<0.05$) (Figure 2). There was a significant positive correlation between the changes of LVMI (LVMI after 18 months –LVMI at baseline) and % changes of ALD ((ALD after 18 months –ALD at baseline)/ALD at baseline×100%) in the efonidipine group (r=0.427, P=0.018) (Figure 3).

DISCUSSION

In this study, (1) we extended the previous pilot study to confirm the long-term effects of replacing amlodipine with efonidipine on the plasma levels of ALD in patients with essential hypertension who had already received ACE-I or ARB; and (2) we showed for the first time

Table 1 Clinical characteristics of patients in the amlodipine and efonidipine groups

	Amlodipine (n=*30)*	*Efonidipine* (n=*30)*	P-*value*
Age (years)	63.8 ± 8.1	64.1 ± 10.5	0.913
Sex (male/female)	16/14	17/13	0.802
Chronic heart failure, *n* (%)	8 (27)	11 (37)	0.423
Heart rate (beats min^{-1})	75 ± 8.9	77.7 ± 12	0.337
Systolic blood pressure (mm Hg)	136.5 ± 13.8	139.3 ± 14	0.438
Diastolic blood pressure (mm Hg)	78.5 ± 10.4	77.6 ± 9.6	0.720
LVEF (%)	63 ± 7.3	63 ± 7.3	0.97
LVMI (g m^{-2})	114 ± 23	121 ± 27	0.295
Serum creatinine (mg per 100 ml)	0.81 ± 0.23	0.84 ± 0.24	0.567
Renin concentration (pg ml^{-1})	45 ± 84	29 ± 46	0.646
Aldosterone (pg ml^{-1})	95 ± 33	106 ± 49	0.312
Brain natriuretic peptide (pg ml^{-1})	49 ± 54	75 ± 63	0.084
Potassium (mequiv l^{-1})	4.2 ± 0.4	4.3 ± 0.4	0.207
Baseline therapy			
ACE-I or ARB, *n* (%)	30 (100)	30 (100)	—
β-Blockers, *n* (%)	9 (30)	15 (50)	*P*=0.187
Spironolactone, *n* (%)	11 (37)	8 (27)	*P*=0.579
Loop diuretics, *n* (%)	3 (10)	7 (23)	*P*=0.189

Abbreviations: ACE-I, angiotensin-converting enzyme inhibitor; ARB, angiotensin receptor blocker; LVEF, left ventricular ejection fraction; LVMI, left ventricular mass index.

Table 2 Clinical and neurohumoral data during 18 months

	Treatment	*Baseline*	*6 months*	*12 months*	*18 months*
HR (beats min^{-1})	Amlodipine	75±8.8	74±7.9	75±7.2	76±7.7
	Efonidipine	77±12	71±9.5*	72±11*	71±10*
SBP (mm Hg)	Amlodipine	136±14	135±11	133±11	135±12
	Efonidipine	139±14	135±15	135±14	133±12
DBP (mm Hg)	Amlodipine	79±10	79±9.3	77±9	77±8.3
	Efonidipine	78±9.6	76±10	77±11	77±9.2
LVEF (%)	Amlodipine	63.8±7.0	—	—	64.4±5.9
	Efonidipine	63.3±7.4	—	—	65.1±7.3
LVMI (g m^{-2})	Amlodipine	114±22	—	—	113±27
	Efonidipine	121±25	—	—	114±21$
Creatinine (pg ml^{-1})	Amlodipine	0.81±0.23	0.84±0.23#	0.85±0.27#	0.86±0.26*
	Efonidipine	0.84±0.24	0.85±0.24	0.89±0.23*	0.89±0.26*
Serum K (mequiv l^{-1})	Amlodipine	4.2±0.4	4.2±0.4	4.1±0.4	4.2±0.4
	Efonidipine	4.3±0.3	4.3±0.4	4.3±0.4	4.3±0.4
BNP (pg ml^{-1})	Amlodipine	49±54	44±49	46±53	54±60
	Efonidipine	75±63	67±62	74±70	70±65
PRC (pg ml^{-1})	Amlodipine	45±84	44±84	38±64	39±60
	Efonidipine	29±46	33±72	38±81	27±72
ALD (pg ml^{-1})	Amlodipine	95±33	101±37	98±43	100±43
	Efonidipine	106±49	76±30*	79±43*	77±34*

Abbreviations: ALD, aldosterone; BNP, brain natriuretic peptide; DBP, diastolic blood pressure; HR, heart rate; LVEF, left ventricular ejection fraction; LVMI, left ventricular mass index; PRC, plasma renin concentration; SBP, systolic blood pressure.
*$P<0.01$ *vs.* the baseline value by analysis of variance with Scheffe's test.
#$P<0.05$ *vs.* the baseline value by analysis of variance with Scheffe's test.
$\$P<0.05$ *vs.* the baseline value.

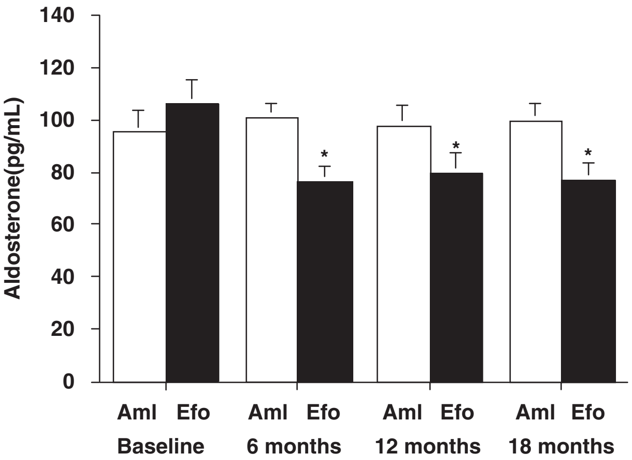

Figure 1 Changes in plasma aldosterone (ALD) levels for 18 months. Closed columns represent patients who received efonidipine (Efo) replaced by amlodipine (Aml), open columns represent patients who continued amlodipine. *$P<0.01$ *vs.* the baseline value by analysis of variance with Scheffe's test.

that the suppression of plasma ALD by efonidipine was sustained for at least 18 months concomitant with a significant decrease in LVMI without changes in blood pressure.

A long-term treatment with ACE-I or ARB does not necessarily induce significant decreases in plasma ALD levels (ALD breakthrough).[1,5–11] ALD breakthrough generally occurs in about half of the cases within 12 months.[8,17] The incidence of ALD breakthrough depends on the length of the follow-up period.[18] In previous studies that defined breakthrough as any increase from an individual's baseline ALD level, the incidence ranged from 10% over 6 months to 53% over 12 months.[8,14–16,19,20] In this study, the follow-up period was 18 months and the incidence of ALD breakthrough was 7% with efonidipine treatment, which was relatively low compared with that in previous studies.[8,14–17,19,20] Moreover, in our study, all patients had already been receiving ACE-I or ARB for more than 1 year, suggesting that some patients had ALD breakthrough. In this population, only two patients (7%) had ALD breakthrough after the replacement of amlodipine with efonidipine, suggesting the usefulness of the combination of ACE-I or ARB and efonidipine.

Calcium ion is conveyed through the T-type calcium channel to the mitochondria, where it activates ALD synthesis. Thereafter, ALD stimulates a T-type calcium channel expression,[21,22] creating a positive feedback loop for ALD biosynthesis in the adrenal cells. Recently, efonidipine, a T-type and L-type calcium channel blocker, has been shown to inhibit ALD synthesis and secretion *in vitro*[12] and *in vivo*.[13,14] Therefore, the different site of action from ACE-I and ARB on ALD secretion and production by efonidipine is key to the assessment of ALD breakthrough during treatment with ACE-I and ARB. In this study, plasma renin concentration did not change significantly in spite of the sustained decrease of ALD for 18 months, suggesting that efonidipine, as a T-type calcium channel blocker, may inhibit renin secretion as shown *in vitro*.

Recently, it has been clarified that ALD can directly damage various organs, such as the heart and blood vessels, through mineralocorticoid receptors, independent of changes in blood pressure.[23–25] In humans, it is suggested that hypertensive incidents and organ damage may occur, even at plasma ALD levels within the normal range.[24,26,27] We previously reported that treatment with atrial natriuretic peptide infusion could prevent left ventricular remodeling in patients with myocardial infarction owing to the suppression of plasma ALD levels.[28,29] Along with the decreased heart rate, efonidipine may have more cardiovascular protective effects than amlodipine because of the decreased plasma ALD level in CHF patients with hypertension.

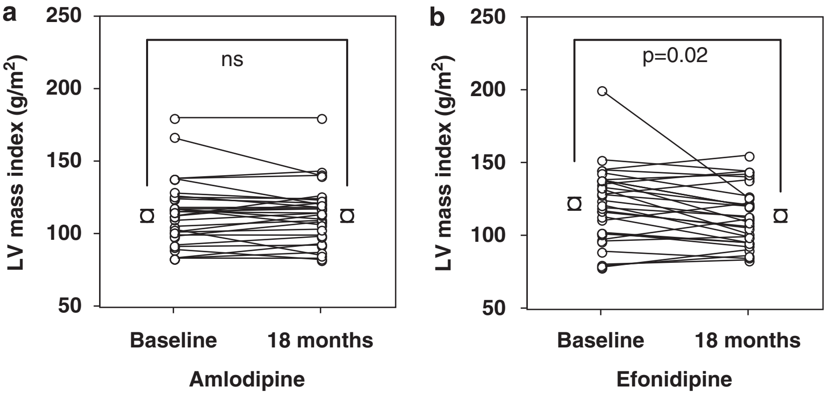

Figure 2 Changes in the left ventricular mass index (LVMI) before and after 18 months. (**a**) Amlodipine group, (**b**) efonidipine group.

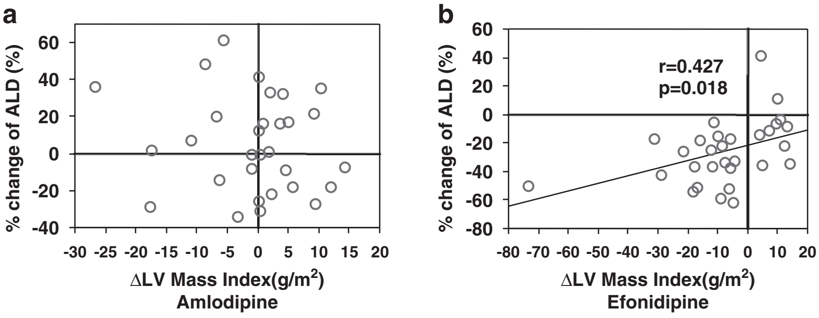

Figure 3 Correlation between the changes in LVMI (LVMI after 18 months −LVMI at baseline) and % changes in ALD ((ALD after 18 months −ALD at baseline)/ALD at baseline×100%). (**a**) Amlodipine group, (**b**) efonidipine group. LVMI, left ventricular mass index; ALD, aldosterone.

This study showed that during the 18-month observation, a clinical dose of efonidipine could decrease the heart rate, an independent risk factor for cardiovascular death in patients with hypertension.[30] This finding is consistent with previous reports.[14,31] Moreover, the plasma ALD level was decreased after the administration of efonidipine and the decrease in ALD was sustained for 18 months, suggesting that efonidipine may be useful for blood pressure control, especially in CHF patients with hypertension. T-type calcium channels are distributed in the sinoatrial node and the conduction system, but not in the cardiac muscle in a normal adult heart. Moreover, recent reports suggest that the reexpression of the T-type calcium channel in the failing heart may contribute to cardiac hypertrophy,[32] to remodeling after myocardial infarction[33,34] and to the development of cardiomyopathy.[35] In this study, LVMI, an important surrogate marker,[36] was significantly decreased after 18 months in the efonidipine group with the sustained reduction of ALD. Taken together with our findings, T-type calcium channel blockers such as efonidipine may be useful for the management of hypertension in patients with CHF. Indeed, our findings may help to explain why the mortality rate was markedly lower in the JATOS study[37] than in other similar clinical trials.

This study has several limitations. Although we took blood samples after patients rested for at least 20 min in a seated position in the present study, and the attending physicians and sonographers were blinded to the neurohumoral data, a single measurement of ALD may have limited value because of variations in the levels, and further studies are required to evaluate the daily urinary ALD secretion. In addition, the LVMI and plasma levels of ALD were slightly higher in the efonidipine group, which may reflect the results. Further studies are needed to clarify this problem in a large number of patients.

CONCLUSIONS

At effective antihypertensive doses of efonidipine and amlodipine, efonidipine significantly decreased the plasma ALD level at 6 months and the suppression of plasma ALD by efonidipine was sustained for at least 18 months. Furthermore, long-term efonidipine therapy decreased LVMI in patients with essential hypertension.

ACKNOWLEDGEMENTS

We thank Miss Ryoko Watanabe for her excellent technical assistance. We also express our thanks to Mr Daniel Mrozek for his assistance in preparing the manuscript. This study was supported by a Grant-in-Aid for Scientific Research in Japan.

1 McKelvie RS, Yusuf S, Pericak D, Avezum A, Burns RJ, Probstfield J, Tsuyuki RT, White M, Rouleau J, Latini R, Maggioni A, Young J, Pogue J. Comparison of candesartan, enalapril, and their combination in congestive heart failure: randomized evaluation of strategies for left ventricular dysfunction (RESOLVD) pilot study. The RESOLVD Pilot Study Investigators. *Circulation* 1999; **100**: 1056–1064.
2 Krum H, Nolly H, Workman D, He W, Roniker B, Krause S, Fakouhi K. Efficacy of eplerenone added to renin-angiotensin blockade in hypertensive patients. *Hypertension* 2002; **40**: 117–123.
3 Pitt B. 'Escape' of aldosterone production in patients with left ventricular dysfunction treated with an angiotensin converting enzyme inhibitor: implications for therapy. *Cardiovasc Drugs Ther* 1995; **9**: 145–149.
4 The RALES Investigators. Effectiveness of spironolactone added on an angiotensin-converting enzyme inhibitor and a loop diuretics for severe chronic congestive heart failure. *Am J Cardiolol* 1996; **78**: 902–907.

5 Staessen J, Lijnen P, Fagard R, Verschueren LJ, Amery A. Rise in plasma concentration of aldosterone during long-term angiotensin II suppression. *J Endocrinol* 1981; **91**: 457–465.
6 Borghi C, Boschi S, Ambrosioni E, Melandri G, Branzi A, Magnani B. Evidence of a partial escape of renin-angiotensin-aldosterone blockade in patients with acute myocardial infarction treated with ACE inhibitors. *J Clin Pharmacol* 1993; **33**: 40–45.
7 Struthers AD. Aldosterone escape during angiotensin-converting enzyme inhibitor therapy in chronic heart failure. *J Card Fail* 1996; **2**: 47–54.
8 Sato A, Saruta T. Aldosterone escape during angiotensin-converting enzyme inhibitor therapy in essential hypertensive patients with left ventricular hypertrophy. *J Int Med Res* 2001; **29**: 13–21.
9 Naruse M, Tanabe A, Sato A, Takagi S, Tsuchiya K, Imaki T, Takano K. Aldosterone breakthrough during angiotensin II receptor antagonist therapy in stroke-prone spontaneously hypertensive rats. *Hypertension* 2002; **40**: 28–33.
10 Sato A, Hayashi K, Naruse M, Saruta T. Effectiveness of aldosterone blockade in patients with diabetic nephropathy. *Hypertension* 2003; **41**: 64–68.
11 Sato A, Saruta T, Funder JW. Combination therapy with aldosterone blockade and renin-angiotensin inhibitors confers organ protection. *Hypertens Res* 2006; **29**: 211–216.
12 Imagawa K, Okayama S, Takaoka M, Takaoka M, Kawata H, Naya N, Nakajima T, Horii M, Uemura S, Saito Y. Inhibitory effect of efonidipine on aldosterone synthesis and secretion in human adrenocarcinoma (H295R) cells. *J Cardiovasc Pharmacol* 2006; **47**: 133–138.
13 Okayama S, Imagawa K, Naya N, Iwama H, Somekawa S, Kawata H, Horii M, Nakajima T, Uemura S, Saito Y. Blocking T-type Ca2+ channels with efonidipine decreased plasma aldosterone concentration in healthy volunteers. *Hypertens Res* 2006; **29**: 493–497.
14 Tanaka T, Tsutamoto T, Sakai H, Fujii M, Yamamoto T, Horie M. Comparison of the effects of efonidipine and amlodipine on aldosterone in patients with hypertension. *Hypertens Res* 2007; **30**: 691–697.
15 Devereux RB, Alonso DR, Lutas EM, Gottlieb GJ, Campo E, Sachs I, Reichek N. Echocardiographic assessment of left ventricular hypertrophy: comparison to necropsy findings. *Am J Cardiol* 1986; **56**: 450–458.
16 Tsutamoto T, Wada A, Maeda K, Hisanaga T, Maeda Y, Fukai D, Ohnishi M, Sugimoto Y, Kinoshita M. Attenuation of compensation of endogenous cardiac natriuretic peptide system in chronic heart failure: prognostic role of plasma brain natriuretic peptide concentration in patients with chronic symptomatic left ventricular dysfunction. *Circulation* 1997; **96**: 509–516.
17 Horita Y, Taura K, Taguchi T, Furusu A, Kohno S. Aldosterone breakthrough during therapy with angiotensin-converting enzyme inhibitors and angiotensin II receptor blockers in proteinuric patients with immunoglobulin A nephropathy. *Nephrology (Carlton)* 2006; **11**: 462–466.
18 Bomback AS, Klemmer PJ. The incidence and implications of aldosterone breakthrough. *Nat Clin Pract Nephrol* 2007; **9**: 486–492.
19 Cicoira M, Zanolla L, Franceschini L, Rossi A, Golia G, Zeni P, Caruso B, Zardini P. Relation of aldosterone 'escape' despite angiotensin-converting enzyme inhibitor administration to impaired exercise capacity in chronic congestive heart failure secondary to ischemic or idiopathic dilated cardiomyopathy. *Am J Cardiol* 2002; **89**: 403–407.
20 Yoneda T, Takeda Y, Usukura M, Usukura M, Oda N, Takata H, Yamamoto Y, Karashima S, Yamagishi M. Aldosterone breakthrough during angiotensin II receptor blockade in hypertensive patients with diabetes mellitus. *Am J Hypertens* 2007; **20**: 1329–1333.
21 Rossier MF, Lesouhaitier O, Perrier E, Bockhorn L, Chiappe A, Lalevee N. Aldosterone regulation of T-type calcium channels. *J Steroid Biochem Mol Biol* 2003; **85**: 383–388.
22 Lalevee N, Rebsamen MC, Barrere-Lemaire S, Perrier E, Nargeot J, Benitah JP, Rossier MF. Aldosterone increases T-type calcium channel expression and *in vitro* beating frequency in neonatal rat cardiomyocytes. *Cardiovasc Res* 2005; **67**: 216–224.
23 Pitt B, Zannad F, Remme WJ, Cody R, Castaigne A, Perez A, Palensky J, Wittes J. The effect of spironolactone on morbidity and mortality in patients with severe heart failure. Randomized Aldactone Evaluation Study Investigators. *N Engl J Med* 1999; **341**: 709–717.
24 Tsutamoto T, Wada A, Maeda K, Mabuchi N, Hayashi M, Tsutsui T, Ohnishi M, Sawaki M, Fujii M, Matsumoto T, Matsui T, Kinoshita M. Effect of spironolactone on plasma brain natriuretic peptide and left ventricular remodeling in patients with congestive heart failure. *J Am Coll Cardiol* 2001; **37**: 1228–1233.
25 Pitt B, Remme W, Zannad F, Neaton J, Martinez F, Roniker B, Bittman R, Hurley S, Kleiman J, Gatlin M. Eplerenone, a selective aldosterone blocker, in patients with left ventricular dysfunction after myocardial infarction. *N Engl J Med* 2003; **348**: 1309–1321.
26 Schunkert H, Hense HW, Muscholl M, Luchner A, Kurzinger S, Danser AH, Riegger GA. Associations between circulating components of the renin-angiotensin-aldosterone system and left ventricular mass. *Heart* 1997; **77**: 24–31.
27 Vasan RS, Evans JC, Larson MG, Wilson PW, Meigs JB, Rifai N, Benjamin EJ, Levy D. Serum aldosterone and the incidence of hypertension in nonhypertensive persons. *N Engl J Med* 2004; **351**: 33–41.
28 Hayashi M, Tsutamoto T, Wada A, Maeda K, Mabuchi N, Tsutsui T, Matsui T, Fujii M, Matsumoto T, Yamamoto T, Horie H, Ohnishi M, Kinoshita M. Relationship between transcardiac extraction of aldosterone and left ventricular remodeling in patients with first acute myocardial infarction: extracting aldosterone through the heart promotes ventricular remodeling after acute myocardial infarction. *J Am Coll Cardiol* 2001; **38**: 1375–1382.
29 Hayashi M, Tsutamoto T, Wada A, Tsutsui T, Ishii C, Ohno K, Fujii M, Taniguchi A, Hamatani T, Nozato Y, Kataoka K, Morigami N, Ohnishi M, Kinoshita M, Horie M. Immediate administration of mineralocorticoid receptor antagonist spironolactone prevents post-infarct left ventricular remodeling associated with suppression of a marker of myocardial collagen synthesis in patients with first anterior acute myocardial infarction. *Circulation* 2003; **107**: 2559–2565.
30 Gillman MW, Kannel WB, Belanger A, D'Agostino RB. Influence of heart rate on mortality among persons with hypertension: the Framingham Study. *Am Heart J* 1993; **125**: 1148–1154.
31 Harada K, Nomura M, Nishikado A, Uehara K, Nakaya Y, Ito S. Clinical efficacy of efonidipine hydrochloride, a T-type calcium channel inhibitor, on sympathetic activities. *Circ J* 2003; **67**: 139–145.
32 Martinez ML, Heredia MP, Delgado C. Expression of T-type Ca(2+) channels in ventricular cells from hypertrophied rat hearts. *J Mol Cell Cardiol* 1999; **31**: 1617–1625.
33 Mulder P, Richard V, Compagnon P, Henry JP, Lallemand F, Clozel JP, Koen R, Mace B, Thuillez C. Increased survival after long-term treatment with mibefradil, a selective T-channel calcium antagonist, in heart failure. *J Am Coll Cardiol* 1997; **29**: 416–421.
34 Huang B, Qin D, Deng L, Boutjdir M, N E-S. Reexpression of T-type Ca2+ channel gene and current in post-infarction remodeled rat left ventricle. *Cardiovasc Res* 2000; **46**: 442–449.
35 Sen L, Smith TW. T-type Ca2+ channels are abnormal in genetically determined cardiomyopathic hamster hearts. *Circ Res* 1994; **75**: 149–155.
36 Ruilope LM, Schmieder RE. Left ventricular hypertrophy and clinical outcomes in hypertensive patients. *Am J Hypertens* 2008; **21**: 500–508.
37 JATOS Study Group. Principal results of the Japanese trial to assess optimal systolic blood pressure in elderly hypertensive patients (JATOS). *Hypertens Res* 2008; **31**: 2115–2127.
