# Supplementary material for: Effects of T-Type Calcium Channel Blockers on Renal Function and Aldosterone in Patients with Hypertension: A Systematic Review and Meta-Analysis
Source: PLoS One. 2014 Oct 17;9(10):e109834. doi: 10.1371/journal.pone.0109834 (PMC4201480; doi:10.1371/journal.pone.0109834)
Supplement: File S3 — PDF files of twenty-four studies included in the meta-analysis. (ZIP) [file pone.0109834.s007.zip › Supporting information-PDF files/20. J Atheroscler Thromb 2009[16(5)]568-575.pdf]

Original Article

Protective Effects of Efonidipine, a T- and L-Type Calcium Channel Blocker, on Renal Function and Arterial Stiffness in Type 2 Diabetic Patients with Hypertension and Nephropathy

Hidehisa Sasaki[1], Atsuhito Saiki[2], Kei Endo[2], Noriko Ban[2], Takashi Yamaguchi[2], Hidetoshi Kawana[2], Daizi Nagayama[2], Masahiro Ohhira[2], Tomokazu Oyama[2], Yoh Miyashita[2], and Kohji Shirai[2]

[1] Departments of Pharmacy, Sakura Medical Center, School of Medicine, Toho University, Chiba, Japan
[2] Departments of Internal Medicine, Sakura Medical Center, School of Medicine, Toho University, Chiba, Japan

Aim: The three types of calcium channel blocker (CCB), L-, T- and N-type, possess heterogeneous actions on endothelial function and renal microvascular function. In the present study, we evaluated the effects of two CCBs, efonidipine and amlodipine, on renal function and arterial stiffness.

Methods: Forty type 2 diabetic patients with hypertension and nephropathy receiving angiotensin receptor II blockers were enrolled and randomly divided into two groups: the efonidipine group was administered efonidipine hydrochloride ethanolate 40 mg/day and the amlodipine group was administered amlodipine besilate 5 mg/day for 12 months. Arterial stiffness was evaluated by the cardio-ankle vascular index (CAVI).

Results: Changes in blood pressure during the study were almost the same in the two groups. Significant increases in serum creatinine and urinary albumin and a significant decrease in the estimated glomerular filtration rate were observed in the amlodipine group, but not in the efonidipine group. On the other hand, significant decreases in plasma aldosterone, urinary 8-hydroxy-2'-deoxyguanosine and CAVI were observed after 12 months in the efonidipine group, but not in the amlodipine group.

Conclusions: These results suggest that efonidipine, which is both a T-type and L-type calcium channel blocker, has more favorable effects on renal function, oxidative stress and arterial stiffness than amlodipine, an L-type calcium channel blocker.

J Atheroscler Thromb, 2009; 16:568-575.

Key words; T-type calcium channel blocker, Efonidipine, Oxidative stress, Diabetic nephropathy, Cardio-ankle vascular index

Introduction

In diabetic patients, hypertension is a frequent complication and accelerates cardiovascular diseases. The main purpose of blood pressure-lowering therapy in diabetic patients is to protect against the progression of nephropathy and atherosclerosis. In diabetes mellitus, angiotensin II receptor blockers (ARB) are mainly used as the first-choice antihypertensive drug. If the hypotensive effect of an ARB is insufficient, calcium channel blockers (CCB) are added in many cases. There exist three types of CCB, the L-, T- and N-type, which have heterogeneous actions on endothelial function and renal microvascular function[1, 2].

Chronic kidney disease (CKD), which includes diabetic nephropathy, is an importance risk factor for the development of cardiovascular diseases[3, 4]. Several studies have found that CKD is a predictor of cardiovascular mortality[5, 6]. The importance of kidney function as a risk factor of CVD is increasingly recognized. Efonidipin, which is both a T-type and L-type CCB,

Address for correspondence: Yoh Miyashita, Department of Internal Medicine, Sakura Medical Center, School of Medicine, Toho University, 564-1 Shimoshizu, Sakura-City, Chiba, 285-0841, Japan
E-mail: mumon@sf6.so-net.ne.jp
Received: January 26, 2009
Accepted for publication: March 19, 2009

has been reported to improve vascular endothelial dysfunction and to have a renoprotective effect[1, 2].

A novel arterial stiffness parameter termed the cardio-ankle vascular index (CAVI) has been developed recently, and essentially reflects the stiffness of the aorta, femoral artery and tibial artery[7].

CAVI is independent of blood pressure, and has adequate reproducibility for clinical use[7]. Furthermore, no special technique is required for the measurement of CAVI. Several reports have demonstrated the usefulness of CAVI for the detection of atherosclerotic diseases[7-10].

In the present study, we evaluated the effect of two CCBs, efonidipine (a T-type and L-type CCB) and amlodipine (an L-type CCB), on renal function and CAVI in type 2 diabetic patients with nephropathy and hypertension.

Subjects and Methods

Subjects

A randomized, open study was performed. Randomization was performed with sequentially numbered, sealed envelopes using a previously determined code for the administration of either efonidipine or amlodipine. Forty type 2 diabetic patients with nephropathy and hypertension, who attended Sakura Medical Center of Toho University as outpatients and had been receiving ARB therapy with candesartan, were enrolled. They had high systolic blood pressure (≧130 mmHg) and/or high diastolic blood pressure (≧80 mmHg) when measurement was performed on two different days within 4 weeks before the start of the study. Patients were excluded if they had previous cardiovascular and cerebrovascular diseases and showed high glycosylated hemoglobin (HbA1c >7.5%). The enrolled subjects were randomly divided into two groups. One group was administered efonidipine 40 mg/day for 12 months (efonidipine group, $n = 20$), and the other group was administered amlodipine 5 mg/day for 12 months (amlodipine group, $n = 20$). During this study, all patients maintained the same dietary and exercise therapies, and did not change medications. All subjects received nutrition education from a dietitian once every 3 months. The clinical profile of the subjects is shown in **Table 1**. This study was approved by the Ethics Committee of Toho University. The purpose, nature and potential risks of this study were explained to all patients, and their voluntary written consent for participation in the study and also for release of the study data was obtained before they were enrolled.

Table 1. Baseline data of the study population

Background factor	Efonidipine	Amlodipine
n (male/femal)	20 (14/6)	20 (12/8)
Age (y.o)	63.3 ± 2.5	65.5 ± 3.0
BMI (kg/m^2)	23.9 ± 0.8	24.2 ± 0.9
Systolic BP (mmHg)	157 ± 4.0	160 ± 3.2
Diastolic BP (mmHg)	85 ± 1.9	87 ± 2.7
Pulse rate (/min)	74 ± 2.6	74 ± 3.0
HbA1c (%)	6.6 ± 0.4	6.7 ± 0.4
TC (mg/dL)	200 ± 12	202 ± 14
TG (mg/dL)	179 ± 30	161 ± 27
HDL-C (mg/dL)	51 ± 3.6	53 ± 4.2
LDL-C (mg/dL)	119 ± 7.4	110 ± 9.6
S-Cr (mg/dL)	1.46 ± 0.18	1.46 ± 0.17
eGFR (mL/min/1.73 m^2)	46 ± 4.9	46 ± 4.6
U-Alb/Cr (mg/g Cr)	1,338 ± 273	1,369 ± 414
8-OHdG (ng/mg Cr)	10.4 ± 0.8	9.8 ± 1.0
ALD (pg/mL)	75 ± 12	78 ± 11
Blood sugar-lowering drugs		
Sulfonyloreas (n)	16	17
Thiazolidinedione (n)	1	0
Alpha-glucosisase inhibitor (n)	12	10
Biguanide (n)	2	1
Lipid-lowering drugs (all atatins)		
atorvastatin (n)	5	7
pitavastatin (n)	5	4
Smoking (n)	4	4

Data are presented as the mean ± SD. BMI, body mass index; BP, blood pressure; HbA1c, glycosylated hemoglobin; TC, total cholesterol; TG, triglycerides; HDL-C, HDL-cholesterol; LDL-C, LDL-cholesterol; S-Cr, serum creatinine; U-Alb/Cr, urinary albumin-to-creatinine ratio; 8-OHdG, 8-hydroxy-2'-deoxyguanosine; ALD, aldosterone; statin, HMG-CoA reductase inhibitor

Measurement of Blood Pressure

Blood pressure (BP) was measured at least twice in a sitting position in the morning after 12 hours of fasting with only candesartan and efonidipine or amlodipine at 7:30 AM.

Assay of HbA1c and Serum Lipids

Blood samples were collected in a sitting position in the morning after 12 hours of fasting. Serum was separated within 1 hour, and samples were used to measure the following chemical parameters. Stable and unstable glycosylated hemoglobin (HbA1c) fractions were measured by high pressure liquid chromatography using the Hi-Auto A1c kit (Kyoto Daiichi Kagaku, Kyoto, Japan). Data of the stable type were used in the present analysis. Total cholesterol (TC), triglyceride (TG) and low-density lipoprotein cholesterol (LDL-C)

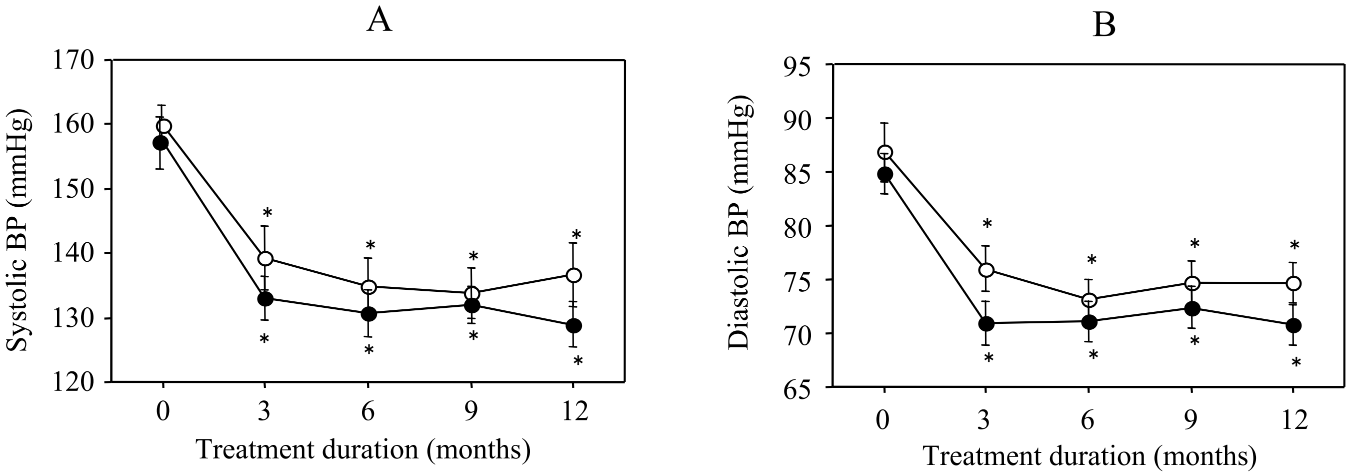

Fig. 1. Changes in systolic (A) and diastolic (B) blood pressure during treatment with efonidipine or amlodipine.

Open circle denotes amlodipine group, and closed circle denotes efonidipine group. Data are presented as the mean ± S.D. $^*p < 0.05$ vs baseline, paired t-test.

were measured with an automatic analyzer (Hitachi 7150 available from Hitachi Tokyo, Japan). High-density lipoprotein cholesterol (HDL-C) was measured by the selective inhibition method (Daiichi Pure Chemicals, Tokyo)[11].

Measurement of Kidney Function

In this study, kidney function was assessed using serum creatinine (Cr), urinary albumin (u-albumin) and the estimated glomerular filtration rate (eGFR). Serum creatinine was assayed enzymatically (Iatro LQ CRE; Mitsubishi Kagaku Iatron). GFR was estimated using a modified MDRD equation, as recently proposed by the Working Group of Japan Chronic Kidney Disease Initiative[12]:

$$\text{eGFR (mL/min per 1.73 m}^2) = 0.741 \times 175 \times \text{age}^{-0.203} \times \text{serum creatinine}^{-1.154} \text{ (if female} \times 0.742)$$

Urinary albumin was measured using the latex agglutination method and adjusted for urinary creatinine (mg per g Cr). Microalbuminuria was defined as ≥30 mg/g creatinine[13].

Plasma Aldosterone and Urinary 8-OHdG Analysis

Plasma aldosterone levels (ALD) were measured using a commercial radioimmunoassay kit (SPAC-S aldosterone kit; TFB Company, Tokyo, Japan).

Urine samples were centrifuged at 800 g for 10 min and the supernatants were used to determine 8-hydroxy-2'-deoxyguanosine (8-OHdG) by a competitive enzyme-linked immunosorbent assay (8-Hydroxy-deoxyguanosine Check; Japan Institute for the Control of Aging, Shizuoka, Japan). The monoclonal antibody has been characterized and found to be specific for 8-OHdG[14]. The results were adjusted for creatinine content (ng per mg Cr) measured in the same urine sample.

Measurement of CAVI

CAVI was measured in the morning, after 12 hours of fasting and taking only efonidipine or amlodipine, using a VaSera CAVI instrument (Fukuda Denshi Co. Ltd., Tokyo) by the methods described previously[7]. Briefly, cuffs were applied to bilateral upper arms and ankles, with the subject supine and the head held in the midline position. After resting for 10 minutes, the examinations were performed. To detect brachial and ankle pulse waves with cuffs, a low cuff pressure of 30 to 50 mmHg was used to ensure minimal effect of cuff pressure on hemodynamics. Blood pressure was measured thereafter. CAVI was calculated by the following formula:

$$\text{CAVI} = a\{(2\rho/\Delta P) \times \ln(Ps/Pd)PWV^2\} + b$$

where Ps is systolic blood pressure, Pd is diastolic blood pressure, PWV is pulse wave velocity, ΔP is Ps − Pd, ρ is blood density, and a and b are constants.

Scale conversion was performed to compare CAVI with PWV (Hasegawa's method). The VaSera was equipped with both measurement and calculation systems, and automatically calculated the CAVI. The average coefficient of variation of CAVI is less than 5%, which is sufficiently small for clinical usage and indicates that CAVI has good reproducibility[7].

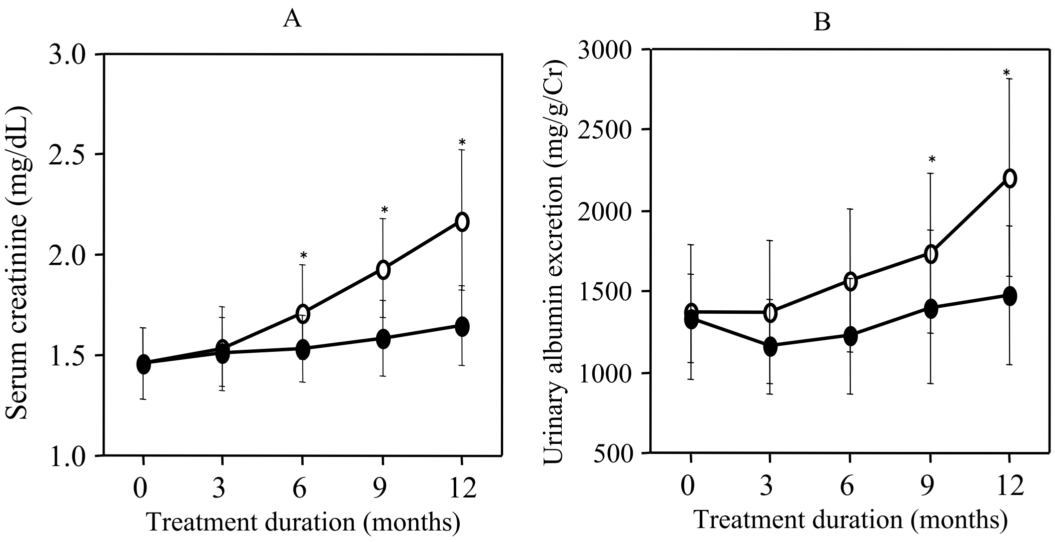

Fig. 2. Changes in serum creatinine (A) and urinary albumin (B) during treatment with efonidipine or amlodipine.

Open circle denotes amlodipine group, and closed circle denotes efonidipine group. Data are presented as the mean ± S.D. $^*p<0.05$ vs baseline, paired t-test.

Statistical Analysis

Groups were compared using Student's t-test or the paired t-test. In all comparisons, values of p less than 0.05 were considered significant.

Results

Changes in BP, BMI, HbA1c and Serum Lipid Levels in Efonidipine and Amlodipine Groups

The changes of BP during this study are shown in **Fig. 1**. In both groups, BP decreased significantly from 3 months of CCB administration, and significant decreases compared to baseline were observed from 3 months through 12 months of treatment; however, no significant differences in changes of BP were observed between groups. During this study, no significant changes in pulse rate were observed in both groups (data not shown). Furthermore, no significant changes in BMI, HbA1c, TC, TG, HDL-C and LDL-C were observed during this study (data not shown).

Effects of Efonidipine or Amlodipine Administration on Renal Functions

Changes in serum Cr and u-albumin are shown in **Fig. 2**. Significant increases in serum Cr (from 6 to 12 months) and u-albumin (at 9 and 12 months) were observed in the amlodipine group, but no significant changes were observed in the efonidipine group during the entire study period. A significant decrease in eGFR was found in the amlodipine group at 12 months, but no significant change was observed in the efonidipine group (**Fig. 3**).

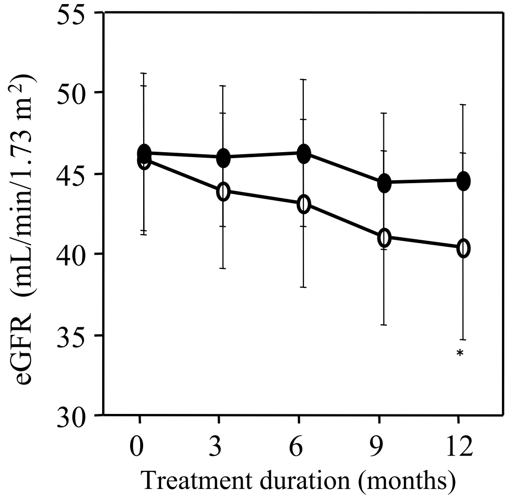

Fig. 3. Changes in estimated glomerular filtration rate (eGFR) during treatment with efonidipine or amlodipine.

Open circle denotes amlodipine group, and closed circle denotes efonidipine group. Data are presented as the mean ± S.D. $^*p<0.05$ vs baseline, paired t-test.

ALD and 8-OHdG Levels Before and After Administration of Efonidipine or Amlodipine

The levels of plasma ALD and urinary 8-OHdG

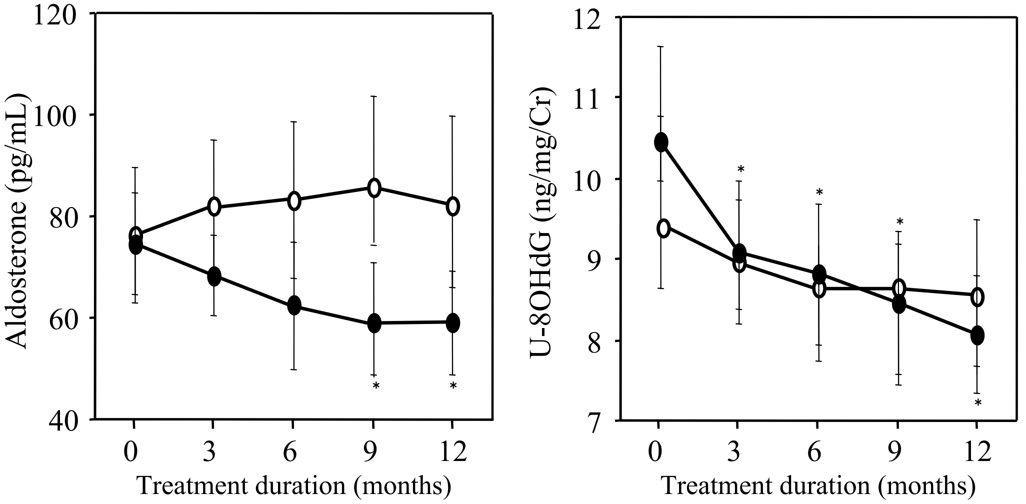

Fig. 4. Changes in plasma aldosterone (A) and urinary 8-hydroxy-2'-deoxyguanosine (8-OHdG) (B) during treatment with efonidipine or amlodipine.

Open circle denotes amlodipine group, and closed circle denotes efonidipine group. Data are presented as the mean ± S.D. $^*p<0.05$ vs baseline, paired t-test.

before and during CCB administration are shown in **Fig. 4**. In the efonidipine group, significant decreases in plasma ALD were observed from 9 to 12 months of treatment while significant decreases of urinary 8-OHdG were observed from 3 through 12 months of treatment; however, no significant changes in ALD and 8-OHdG were observed in the amlodipine group during the entire study period.

Changes of CAVI in Efonidipine or Amlodipine Group

The changes of CAVI are shown in **Fig. 5**. In the efonidipine group, a significant decrease in CAVI was observed after 12 months of treatment; however, no significant change in CAVI was observed in the amlodipine group.

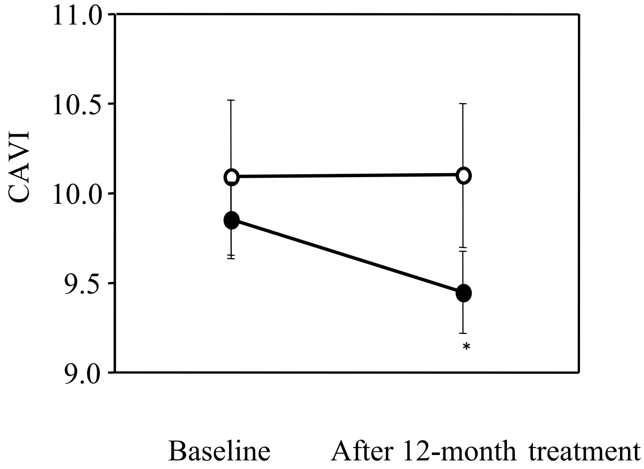

Fig. 5. Change in CAVI before and after treatment with efonidipine or amlodipine.

Open circle denotes amlodipine group, and closed circle denotes efonidipine group. Data are presented as the mean ± S.D. $^*p<0.05$ vs baseline, paired t-test.

Discussion

In the present study, serum Cr and u-albumin increase significantly and eGFR decreased significantly during treatment in the amlodipine group, while these parameters remained unchanged in the efonidipine group. Furthermore, significant decreases of plasma ALD, urinary 8-OHdG and CAVI were observed in the efonidipine group, but not in the amlodipine group. These results suggest that efonidipine has more favorable effects on preserving renal function, reducing oxidative stress and decreasing arterial stiffness compared to amlodipine. In this study, age and the male/female ratio were not significantly different between groups; therefore, the results were considered not to be influenced by these factors.

Several studies indicate that T-type CCBs is superior to L-type CCBs in terms of the protective effect of renal function[2, 15, 16]. The mechanism of this effect is generally considered to be due to the aldosterone-decreasing effect by T-type CCBs[2, 17]. ALD is synthesized in zona glomerulosa of the adrenal gland, and secreted mainly by calcium influx through the T-type

calcium channel[18, 19]. In an *in vitro* study, efonidipine has been shown to inhibit aldosterone synthesis in cultured adrenocortical cells via T-type CCBs[20]. It has also been observed that the administration of efonidipine lowers plasma aldosterone in healthy subjects and hypertensive subjects[21, 22]. The ALD-decreasing potential of efonidipine is reported to result in a renoprotection effect[2]. In the present study, ALD also decreased significantly in the efonidipine group; therefore, we consider that the ALD-decreasing potential of efonidipine may contribute to the reno-protection effect observed in this study. However, we also consider that the decrease of oxidative stress by efonidipine could contribute to the protection of renal function, because oxidative stress has been associated with nephropathy and other diabetic complications[23-25]. In this study, significant decreases of urinary 8-OHdG were observed in the efonidipine group, but not in the amlodipine group. This effect of efonidipine has been already reported by Oshima T. *et al.*[1]. Compound 8-OHdG is one of the most common markers used to evaluate oxidative DNA damage, and it is a product formed by specific attack of a hydroxyl radical on DNA[26]. Furthermore, several studies have demonstrated the free radical scavenging activity of CCBs *in vitro*[27-29]. So, we consider that efonidipine might have an improving effect on oxidative stress. This effect of efonidipine might be accounted for by a reno-protection effect. Further studies are required to clarify whether the ALD-decreasing effect and oxidative stress-reducing effect are independent or related.

Arterial stiffness is known to be associated with renal insufficiency[30-32]. Mourad *et al.*[31] reported a negative association between creatinine clearance and carotid-femoral PWV in patients with mild renal insufficiency. Recently, Wang *et al.*[32] showed a stepwise increase in aortic PWV corresponding to an increase in the severity of CKD from stage 1 to 5 classified according to the KDOQI guidelines[33]. These previous studies evaluated arterial stiffness using PWV, but PWV essentially depends on blood pressure[34]. In the present study, CAVI was used to evaluate arterial stiffness and CAVI is independent of blood pressure[34]; therefore, we consider that arterial stiffness was evaluated more accurately in this study than in previous reports.

There are several reports about the mechanism of the association between arterial stiffness and renal insufficiency[30, 35]. Among them, oxidative stress and endothelial dysfunction are important mechanisms that link renal insufficiency and arterial stiffness[36, 37]. Increased 8-OHdG has been suggested to contribute to atherogenetic processes by inducing endothelial dysfunction[38, 39]. Endothelial dysfunction is associated with the accumulation of cardiovascular risk factors[40] and involves both large and small arteries, promoting progression of renal disease and contributing to acceleration of atherogenesis[41]. Thus, progression of renal dysfunction may in turn induce vascular injury in large and small arteries and contribute to increased arterial stiffness.

Past study has already suggested that efonidipine could be superior to L-type CCB in improving oxidative stress and endothelial function, and that the improvement of vascular endothelial dysfunction may be due to a reduction of oxidative stress[1]. In the present clinical study, we demonstrated that efonidipine was superior to amlodipine in reducing 8-OHdG and CAVI and in maintaining renal function in diabetic patients with hypertension and nephropathy. We speculate that the oxidative stress-reducing potential of efonidipine probably accounts for its favorable effects on renal function and arterial stiffness. The inhibition of T-type calcium channel by efonidipine and not by amlodipine may explain the differential effect of the two CCBs on oxidative stress. T-type calcium channels have been identified in the vasculature[42-44]; however, the physiological roles and the action mechanisms of these channels in the vasculature have not yet been clarified. Therefore, it remains difficult to speculate about the precise mechanism by which efonidipine improves the state of oxidative stress.

HMG-CoA reductase inhibitors (statins) are also known to improve endothelial dysfunction and arterial stiffness[45, 46]. Furthermore, several reports indicate that statins improve oxidative stress[47, 48]. So, statins might expect to improve CAVI although there are still no reports about the effects of statins on CAVI. We report that pitavastatin may be effective to improve CAVI in patients with enhanced oxidative stress (J Atheroscler Thromb, in press). In the present study, the statin administration ratio was not significantly different between two groups; therefore, the results were considered not to be influenced by statins.

There are several limitations of this study, the most important of which was the study protocol. This study was designed for only 12 months and with a small subject pool. To clarify the different effects between efonidipine and amlodipine, a large trial over a long period is needed in the future. In addition, we evaluated oxidative stress using only 8-OHdG. This marker is not always consistent with oxidative stress in the whole body and sometimes varies widely. To evaluate the oxidative stress-reducing potential of efonidipine, other markers, such as NADPH oxidase, free radicals and superoxide dismutase, should be used.

In summary, a T- and L-type calcium channel blocker, but not an L-type calcium channel blocker, protected renal function and improved arterial stiffness in diabetic patients with hypertension and nephropathy, probably through reducing oxidative stress. This finding is expected to be useful in the selection of CCBs when the hypotensive effect of an ARB is insufficient in diabetic patients with hypertension and nephropathy.

References

1) Oshima T, Ozono R, Yano Y, Higashi Y, Teragawa H, Miho N, Ishida T, Ishida M, Yoshizumi M, Kambe M: Beneficial effect of T-type calcium channel blockers on endothelial function in patients with essential hypertension. Hypertens Res, 2005; 28: 889-894
2) Ishimitsu T, Kameda T, Akashiba A, Takahashi T, Ohta S, Yoshii M, Minami J, Ono H, Numabe A, Matsuoka H: Efonidipine reduces proteinuria and plasma aldosterone in patients with chronic glomerulonephritis. Hypertens Res, 2007; 30: 621-626
3) Sarnak MJ, Levey AS, Schoolwerth AC, Coresh J, Culleton B, Hamm LL, McCullough PA, Kasiske BL, Kelepouris E, Klag MJ, Parfrey P, Pfeffer M, Raij L, Spinosa DJ, Wilson PW: American Heart Association Councils on Kidney in Cardiovascular Disease, High Blood Pressure Research, Clinical Cardiology, and Epidemiology and Prevention: Kidney disease as a risk factor for development of cardiovascular disease: A statement from the American Heart Association Councils on kidney in cardiovascular disease, high blood pressure research, clinical cardiology, and epidemiology and prevention. Circulation, 2003; 108: 2154-2169
4) Eknoyan G, Lameire N, Barsoum R, Eckardt KU, Levin A, Levin N, Locatelli F, MacLeod A, Vanholder R, Walker R, Wang H: The burden of kidney disease: improving global outcomes. Kidney Int, 2004; 66: 1310-1314
5) K/DOQI clinical practice guidelines for chronic kidney disease: Evaluation, classification, and stratification. Am J Kidney Dis, 2002; 39: S1-S266
6) Weiner DE, Tighiouart H, Amin MG, Stark PC, MacLeod B, Griffith JL, Salem DN, Levey AS, Sarnak MJ: Chronic kidney disease as a risk factor for cardiovascular disease and all-cause mortality: A pooled analysis of community-based studies. J Am Soc Nephrol, 2004; 15: 1307-1315
7) Shirai K, Utino J, Otsuka K, Takata M: A novel blood pressure-independent arterial wall stiffness parameter; cardio-ankle vascular index (CAVI). J Atheroscler Thromb, 2006; 13: 101-107
8) Yambe T, Meng X, Hou X, Wang Q, Sekine K, Shiraishi Y, Watanabe M, Tamaguchi T, Shibata M, Kuwayama T, Maruyama M, Konno S, Nitta S: Cardio-ankle vascular index (CAVI) for the monitoring of the atherosclerosis after heart transplantation. Biomed Pharmacother, 2005; 59 (Suppl 1): S177-S179
9) Kubozono T, Miyata M, Ueyama K, Nagaki A, Otsuji Y, Kusano K, Kubozono O, Tei C: Clinical significance and reproducibility of new arterial distensibility index. Circ J, 2007; 71: 89-94
10) Nakamura K, Tomaru T, Yamamura S, Miyashita Y, Shirai K, Noike H: Cardio-ankle vascular index is a candidate predictor of coronary atherosclerosis. Circ J, 2008; 72: 598-604
11) Shirai K, Nema T, Hiroh Y, Itoh Y, Miyashita Y, Watanabe H: Clinical efficacy of the direct assay method using polymers for serum high density lipoprotein cholesterols. J Clin Lab Anal, 1992; 11: 82-86
12) Imai E, Horio M, Nitta K, Yamagata K, Iseki K, Hara S, Ura N, Kiyohara Y, Hirakata H, Watanabe T, Moriyama T, Ando Y, Inaguma D, Narita I, Iso H, Wakai K, Yasuda Y, Tsukamoto Y, Ito S, Makino H, Hishida A, Matsuo S: Estimation of glomerular filtration rate by the MDRD study equation modified for Japanese patients with chronic kidney disease. Clin Exp Nephrol, 2007; 11: 41-50
13) American Diabetes Association: Nephropathy in diabetes, Diabetes Care, 27 (Suppl. 1) (2004), pp. S79-S83
14) Toyokuni S, Tanaka T, Hattori Y, Nishiyama Y, Yoshida A, Uchida K, Hiai H, Ochi H, Osawa T: Quantitative immunohistochemical determination of 8-hydroxy-2' deoxyguanosine by a monoclonal antibody N45.1: its application to ferric nitrilotriacetate-induced renal carcinogenesis model. Lab Invest, 1997; 76: 365-374
15) Honda M, Hayashi K, Matsuda H, Kubota E, Tokuyama H, Okubo K, Takamatsu I, Ozawa Y, Saruta T: Divergent renal vasodilator action of L- and T-type calcium antagonists in vivo. J Hypertens, 2001; 19: 2031-2037
16) Ohishi M, Takagi T, Ito N, Terai M, Tatara Y, Hayashi N, Shiota A, Katsuya T, Rakugi H, Ogihara T: Renal-protective effect of T-and L-type calcium channel blockers in hypertensive patients: an Amlodipine-to-Benidipine Changeover (ABC) study. Hypertens Res, 2007; 30: 797-806
17) Sato A, Hayashi K, Saruta T: Antiproteinuric effects of mineralocorticoid receptor blockade in patients with chronic renal disease. Am J Hypertens, 2005; 1: 44-49
18) Barrett PQ, Isales CM, Bollag WB, McCarthy RT: Ca^{2+} channels and aldosterone secretion: modulation by K^+ and atrial natriuretic peptide. Am J Physiol, 1991; 261: F706-719
19) Rossier MF, Ertel EA, Vallotton MB, Capponi AM: Inhibitory action of mibefradil on calcium signaling and aldosterone synthesis in bovine adrenal glomerulosa cells. J Pharmacol Exp Ther, 1998; 287: 824-831
20) Imagawa K, Okayama S, Takaoka M, Kawata H, Naya N, Nakajima T, Horii M, Uemura S, Saito Y: Inhibitory effect of efonidipine on aldosterone synthesis and secretion in human adrenocarcinoma (H295R) cells. J Cardiovasc Pharmacol, 2006; 47: 133-138
21) Okayama S, Imagawa K, Naya N, Iwama H, Somekawa S, Kawata H, Horii M, Nakajima T, Uemura S, Saito Y: Blocking T-type Ca^{2+} channels with efonidipine decreased plasma aldosterone concentration in healthy volunteers. Hypertens Res, 2006; 29: 493-497
22) Tanaka T, Tsutamoto T, Sakai H, Fujii M, Yamamoto T, Horie M: Comparison of the effects of efonidipine and amlodipine on aldosterone in patients with hypertension. Hypertens Res, 2007; 30: 691-697

23) Baynes JW, Thorpe SR: The role of oxidative stress in diabetic complications. Curr Opin Endocrinol, 1997; 3: 277-284
24) Van Dam PS, Van Asbeck BS, Erkelens DW, Marx JJM, Gispen WH, Bravenboer B: The role of oxidative stress in neuropathy and other diabetic complications. Diabetes Metab Rev, 1995; 11: 181-192
25) Baynes JW, Thorpe SR: Role of oxidative stress in diabetic complications. Diabetes, 1999; 48: 1-9
26) Shigenaga MK, Gimeno CJ, Ames BN: Urinary 8-hydroxy-2'-deoxyguanosine as a biological marker of in vivo oxidative DNA damage. Proc Natl Acad Sci USA, 1989; 86: 9697-9701
27) Mak TI, Boehme P, Weglicki WB: Antioxidant effects of calcium channel blockers against free radical injury in endothelial cells. Circ Res, 1992; 70: 1099-1103
28) Umemoto S, Tanaka M, Kawahara S, Kubo M, Umeji K, Hashimoto R, Matsuzaki M: Calcium antagonist reduces oxidative stress by upregulating Cu/Zn superoxide dismutase in stroke-prone spontaneously hypertensive rats. Hypertens Res, 2004; 27: 877-885
29) Yasunari K, Maeda K, Nakamura M, Watanabe T, Yoshikawa J: Benidipine, a long-acting calcium channel blocker, inhibits oxidative stress in polymorphonuclear cells in patients with essential hypertension. Hypertens Res, 2005; 28: 107-112
30) Safar ME, London GM, Plante GE: Arterial stiffness and kidney function. Hypertension, 2004; 43: 163-168
31) Mourad JJ, Pannier B, Blacher J, Rudnichi A, Benetos A, London GM, Safar ME: Creatinine clearance, pulse wave velocity, carotid compliance and essential hypertension. Kidney Int, 2001; 59: 1834-1841
32) Wang MC, Tsai WC, Chen JY, Huang JJ: Stepwise increase in arterial stiffness corresponding with the stages of chronic Kidney disease. Am J Kidney Dis, 2005; 45: 494-501
33) Levey AS, Eckardt KU, Tsukamoto Y, Levin A, Coresh J, Rossert J, De Zeeuw D, Hostetter TH, Lameire N, Eknoyan G: Definition and classification of chronic kidney disease: A position statement from Kidney Disease: Improving Global Outcomes (KDIGO). Kidney Int, 2005; 67: 2089-2100
34) Takaki A, Ogawa H, Wakeyama T, Iwami T, Kimura M, Hadano Y, Matsuda S, Miyazaki Y, Hiratsuka A, Matsuzaki M: Cardio-ankle vascular index is superior to brachial-ankle pulse wave velocity as an index of arterial stiffness. Hypertens Res, 2008; 31: 1347-1355
35) Schiffrin EL, Lipmann ML, Mann JF: Chronic kidney disease: effects on the cardiovascular system. Circulation, 2007; 116: 85-97
36) Stam F, van Guldener C, Becker A, Dekker JM, Heine RJ, Bouter LM, Stehouwer CD: Endothelial dysfunction contributes to renal function-associated cardiovascular mortality in a population with mild renal insufficiency: The Hoorn study. J Am Soc Nephrol, 2006; 17: 537-545
37) Amabile N, Guérin AP, Leroyer A, Mallat Z, Nguyen C, Boddaert J, London GM, Tedgui A, Boulanger CM: Circulating endothelial microparticles are associated with vascular dysfunction in patients with end-stage renal failure. J Am Soc Nephrol, 2005; 16: 3381-3388
38) Fan J, Watanabe T: Inflammatory reactions in the pathogenesis of atherosclerosis. J Atheroscler Thromb, 2003; 10: 63-71
39) Lei YC, Hwang JS, Chan CC, Lee CT, Cheng TJ: Enhanced oxidative stress and endothelial dysfunction in streptozotocin-diabetic rats exposed to fine particles. Environ Res, 2005; 99: 335-343
40) Tsuchiya K, Nakayama C, Iwashima F, Sakai H, Izumiyama H, Doi M, Hirata Y: Advanced endothelial dysfunction in diabetic patients with multiple risk factors; importance of insulin resistance. J Atheroscler Thromb, 2007; 14: 303-309
41) Endemann DH, Schiffrin EL: Endothelial dysfunction. J Am Soc Nephrol, 2004; 15: 1983-1992
42) Chen CC, Lamping KG, Nuno DW, Barresi R, Prouty SJ, Lavoie JL, Cribbs LL, England SK, Sigmund CD, Weiss RM, Williamson RA, Hill JA, Campbell KP: Abnormal coronary function in mice deficient in α1H T-type Ca^{2+} channels. Science, 2003; 302: 1416-1418
43) Vanbavel E, Sorop O, Andreasen D, Pfaffendorf M, Jensen BL: Role of T-type calcium channels in myogenic tone of skeletal muscle resistance arteries. Am J Physiol Circ Physicl, 2002; 283: H2239-H2243
44) Nilius B, Prenen J, Kamouchi M, Viana F, Voets T, Droogmans G: Inhibition by mibefradil, a novel calcium antagonist, of Ca^{2+}- and volume-activated Cl- channels in macrovascular endothelial cells. Br J Pharmacol, 1997; 121: 547-555
45) Shinohara K, Shoji T, Kimoto E, Yokoyama H, Fujiwara S, Hatsuda S, Maeno T, Shoji T, Fukumoto S, Emoto M, Koyama H, Nishizawa Y: Effect of atorvastatin on regional arterial stiffness in patients with type 2 diabetes mellitus. J Atheroscler Thromb, 2005; 12: 205-210
46) Hongo M, Tsutsui H, Mawatari E, Hidaka H, Kumazaki S, Yazaki Y, Takahashi M, Kinoshita O, Ikeda U: Fluvastatin improves arterial stiffness in patients with coronary artery disease and hyperlipidemia: a 5-year follow-up study. Circ J, 2008; 72: 722-728
47) Matsuo T, Iwade K, Hirata N, Yamashita M, Ikegami H, Tanaka N, Aosaki M, Kasanuki H: Improvement of arterial stiffness by the antioxidant and anti-inflammatory effects of short-term statin therapy in patients with hypercholesterolemia. Heart Vessels, 2005; 20: 8-12
48) Endo K, Miyashita Y, Sasaki H, Ebisuno M, Ohira M, Saiki A, Koide N, Oyama T, Takeyoshi M, Shirai K: Probucol and atorvastatin decrease urinary 8-hydroxy-2'-deoxyguanosine in patients with diabetes and hypercholesterolemia. J Atheroscler Thromb, 2006; 13: 68-75
