# Supplementary material for: Effects of T-Type Calcium Channel Blockers on Renal Function and Aldosterone in Patients with Hypertension: A Systematic Review and Meta-Analysis
Source: PLoS One. 2014 Oct 17;9(10):e109834. doi: 10.1371/journal.pone.0109834 (PMC4201480; doi:10.1371/journal.pone.0109834)
Supplement: File S3 — PDF files of twenty-four studies included in the meta-analysis. (ZIP) [file pone.0109834.s007.zip › Supporting information-PDF files/21. Hypertens Res 2009[32(4)]270-275.pdf]

Hypertension Research (2009) 32, 270–275
© 2009 The Japanese Society of Hypertension All rights reserved 0916-9636/09 $32.00
www.nature.com/hr

ORIGINAL ARTICLE

Comparison of the antiproteinuric effects of the calcium channel blockers benidipine and amlodipine administered in combination with angiotensin receptor blockers to hypertensive patients with stage 3–5 chronic kidney disease

Masanori Abe, Kazuyoshi Okada, Takashi Maruyama, Noriaki Maruyama and Koichi Matsumoto

Benidipine, an L- and T-type calcium channel blocker, dilates both efferent and afferent arterioles and reduces glomerular pressure. Thus, it may exert renoprotective effects. We conducted an open-labeled, randomized trial to compare the blood pressure (BP)-lowering effect and antiproteinuric effect of benidipine with those of amlodipine in hypertensive patients with moderate-to-advanced-stage chronic kidney disease (CKD) (stages 3–5). These patients were already being administered the current maximum recommended doses of angiotensin receptor blockers (ARBs). Patients with BP $\geqslant$140/90 mm Hg, despite treatment with the maximum recommended dose of ARBs, were randomly assigned to two groups. The patients received either of the following treatment regimens: 4 mg day^{-1} of benidipine, which was increased up to a dose of 16 mg day^{-1} (B group; n=24), and 2.5 mg day^{-1} of amlodipine, which was increased up to a dose of 10 mg day^{-1} amlodipine (A group; n=23). After 6 months of treatment, a significant and comparable reduction in the systolic and diastolic BP was seen in both groups. The decrease in the urinary protein to creatinine ratio in the B group was significantly lower than that in the A group. Benidipine exerted antiproteinuric effect to a greater extent than did amlodipine, even in patients with diabetic nephropathy. We conclude that the addition of benidipine, rather than amlodipine, ameliorates urinary protein excretion in hypertensive patients with CKD who are already being administered ARBs. Therefore, we propose a combination therapy with benidipine and ARBs, even for patients with moderate-to-advanced-stage CKD.
Hypertension Research (2009) **32,** 270–275; doi:10.1038/hr.2009.11; published online 27 February 2009

Keywords: angiotensin receptor blocker; benidipine; chronic kidney disease; proteinuria

INTRODUCTION

On the basis of the results of several mega trials, including the MDRD (Modification of Diet in Renal Disease) study, a strict control of blood pressure (BP) is recommended in hypertensive patients with chronic kidney disease (CKD).[1] Proteinuria is one of the clinical parameters for diagnosing renal damage, particularly in glomerular hypertension, and it has been reported to be a risk factor and predictor of cardiovascular events.[2,3] Therefore, suppression of proteinuria is a major goal in the treatment of hypertensive patients with CKD. The renoprotective effects of angiotensin receptor blockers (ARBs) and angiotensin-converting enzyme inhibitors (ACEIs) have been shown earlier.[4–6] Blockade of the renin–angiotensin system (RAS) with ARBs or ACEIs is currently the most effective pharmacological tool for renoprotection. These agents reduce proteinuria more effectively than other antihypertensive agents.[7,8] On the basis of these results, ARBs and ACEIs are recommended as first-choice drugs for the treatment of hypertensive patients with CKD, according to the Japanese Society of Hypertension Guidelines for the Management of Hypertension (JSH 2004).[9] However, it is difficult-to-control BP with monotherapy, particularly in patients with CKD; this highlights the need for a combination drug therapy.[10,11]

Calcium channel blockers (CCBs) reduce BP and are useful anti-hypertensive drugs. There are three types of calcium channels: L-type calcium channels, which are distributed widely in smooth muscle cells of peripheral resistance arteries; N-type channels, which are located in the cells of the brain; and T-type channels, which are localized to the sinus node and the brain. In renal tissues, the L-type calcium channels are present only in the afferent arterioles, whereas the N-type and T-type calcium channels are localized in both efferent and afferent arterioles.[12,13] Amlodipine is a representative CCB that is used widely

Division of Nephrology, Hypertension and Endocrinology, Department of Medicine, Nihon University School of Medicine, Tokyo, Japan
Correspondence: Dr M Abe, Division of Nephrology, Hypertension and Endocrinology, Department of Medicine, Nihon University School of Medicine, 30-1, Oyaguchi-kamimachi, Itabashi-ku, Tokyo 173-8610, Japan.
E-mail: mabe@med.nihon-u.ac.jp
Received 24 October 2008; revised 25 December 2008; accepted 12 January 2009; published online 27 February 2009

all over the world; it blocks the L-type calcium channel and dilates the afferent arterioles to a higher degree than the efferent arterioles.[14,15] However, it has recently been reported that both a dual L-/N-type CCB cilnidipine and an L-/T-type CCB efonidipine exhibit a greater antiproteinuric effect than does amlodipine.[16,17] Benidipine, another dual L-/T-type CCB, dilates both efferent and afferent arterioles and reduces glomerular pressure.[18] Therefore, benidipine also may exert renoprotective effects.

However, few studies have reported the renoprotective effect of benidipine in hypertensive patients with mild albuminuria.[13,19] The mechanism by which benidipine exerts renoprotective and antihypertensive effects in hypertensive patients with CKD has not been elucidated. The objective of this study is to determine the effects of benidipine on BP and renoprotection in Japanese hypertensive patients with moderate-to-advanced stages of CKD (stages 3–5) and who are already being treated with a maximum recommended dose of ARBs.

METHODS

This study was a 6-month-long, single-center, prospective, randomized, open-labeled clinical trial. It was designed to compare the BP-lowering and antiproteinuric effects of benidipine and amlodipine in hypertensive patients with stage 3–5 CKD. These subjects were already being administered the maximum recommended dose of ARBs. We obtained written informed consents for participation in the trial from all patients, and the protocol of the trial was approved by the ethics committee of our institution. The study was conducted in accordance with the Declaration of Helsinki.

The enrollment criteria for the subjects included: (1) hypertension that is, systolic and diastolic BP $\geqslant$140/90 mm Hg, as measured in the sitting position on at least two separate visits to the clinics; (2) stage 3–5 CKD, as indicated by an estimated glomerular filtration rate (eGFR) of $<60\,ml\,min^{-1}$ per $1.73\,m^2$; (3) proteinuria, that is, urinary protein–creatinine (Cr) ratio $\geqslant 300\,mg\,g^{-1}$ (average of two consecutive measurements taken during a 4-week period before the treatment); (4) treatment with the maximum recommended dose of an ARB ($40\,mg\,day^{-1}$ of olmesartan or $80\,mg\,day^{-1}$ of telmisartan) for at least 8 weeks before the study.

The exclusion criteria were as follows: (1) age <20 years; (2) hypertensive emergency; (3) history of severe heart failure, angina, myocardial infarction or stroke within 6 months before the start of the trial; (4) earlier treatment with steroids or immunosuppressants; (5) renovascular hypertension or endocrine hypertension; (6) severe diabetes mellitus that led to hospitalization because of extremely high plasma glucose or that was associated with complications, such as diabetic ketoacidosis.

The overview of the study design is shown in Figure 1. The subjects were randomly assigned to two groups at the time of registration. An independent monitoring investigator, who did not treat the study subjects and who did not know the profile of the subjects before the start of the trial, monitored randomization in the order of entry of the subjects; thereafter, the particulars of the assignments were delivered immediately to the individual investigators. Dynamic balancing randomization was carried out on the basis of the serum Cr (sCr), urinary protein–Cr ratio measured at the time of registration and the presence or absence of diabetic nephropathy. This was to ensure that there were no significant differences between the baseline characteristics of the two groups. The patients received either of the following treatment regimens: $4\,mg\,day^{-1}$ benidipine, which was increased to daily doses of 16 mg (B group), and 2.5 mg amlodipine, which was increased to daily doses of 10 mg (A group).

Blood pressure was measured in the outpatient clinic at fixed times after the medications were ingested. BP measurement was carried out according to the Japanese Society of Hypertension Guidelines for the Management of Hypertension.[9] On each monthly visit to the outpatient clinic, the BP was measured using a sphygmomanometer (Nippon Colin Co. Ltd., Tokyo, Japan); the measurements were carried out twice with the patient in the sitting position after a 5-min rest. The patients were provided guidance on how to maintain their diet, particularly those following dietary restrictions.

The doses of the ARB and ACEI were not altered during the study period. The target BP level was $<$130/80 mm Hg. During the first 3 months of study, the patients were administered a combination drug therapy with other conventional antihypertensive agents at baseline. However, if benidipine or amlodipine in combination with an RAS inhibitor failed to reduce the BP to the target level within 3 months, additional antihypertensive medications (other than RAS inhibitors or CCBs) were administered to achieve the target BP. Withdrawal of drug treatment was considered in patients who developed an allergy/intolerance to benidipine or amlodipine during the study period, developed a hypertensive emergency or showed any other condition or received another therapy that, in the opinion of the investigators, might pose a risk for the patient or confound the results of the study.

All the parameters that were used for monitoring the effects of the drugs were evaluated once every month during the 24-week period of treatment. Laboratory values, such as sCr and potassium, were measured using commercial kits employing routinely used clinical chemistry procedures. To assess urinary protein excretion, we measured the urinary concentrations of protein and Cr (protein–Cr ratio). Urinary protein was measured using the pyrogallol red method. The treatment compliance and safety variables were checked at each visit to our hospital.

The glomerular filtration rate (GFR) was estimated using the final recommended modified equation for Japanese patients by the JSN-CKDI (Japanese Society of Nephrology-Chronic Kidney Disease Initiatives), because the eGFR obtained by this method is more accurate for application in Japanese patients

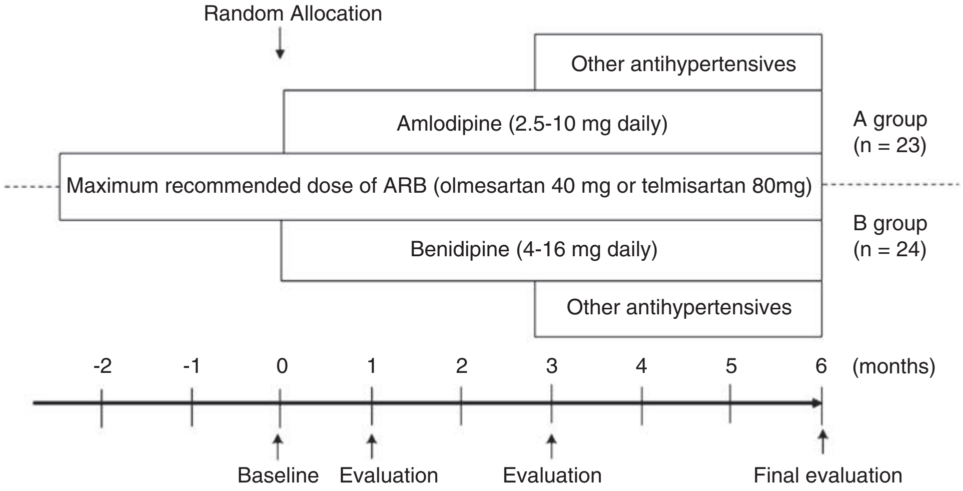

Figure 1 Study design. ARB, angiotensin receptor blocker.

with CKD.[20] The eGFR was calculated by the following formula: eGFR (ml min^{-1} per 1.73 m^2)=194×sCr$^{-1.094}$×Age$^{-0.287}$(×0.739 in the case of women).

Statistical analysis

Data were analyzed according to the randomly assigned groups of the participants, regardless of their subsequent medication status (intention-to-treat analysis), and expressed as the mean ± s.e.m. The baseline characteristics of the enrolled patients were tested to compare between the A and B groups using the unpaired *t*-test or Fisher's exact test. The mean values in the two groups were compared using the unpaired *t*-test. Analysis of variance with repeated measurements and a subsequent multiple comparison test were applied to test the effect of treatment on BP, heart rate and urinary protein–Cr ratio. Statistical significance was set at $P<0.05$.

RESULTS

Baseline characteristics

All the enrolled patients (n=47) continued to participate in the study until the end of the trial. The baseline characteristics and medications administered to the subjects in the two groups are shown in Table 1. No significant differences were observed between the two groups with regard to the baseline characteristics or the number of patients with diabetic nephropathy. Although the subjects were not obese (mean body mass index of all subjects, 23.6 ± 0.85 kg m^{-2}), and their blood glucose and lipid levels at the baseline were well under control, adequate BP control was not achieved in any of the enrolled patients.

Table 1 Baseline characteristics and medication details of the study subjects

	A group	*B group*	P-*value*
Number of patients (male/female)	23 (15/8)	24 (15/9)	NS
Age (years)	65.5 ± 2.1	65.9 ± 2.2	NS
Body mass index (kg m^{-2})	23.8 ± 0.4	23.5 ± 0.5	NS
Type II diabetes (*n*)	11	11	NS
Hyperlipidemia (*n*)	9	8	NS
Smoker (*n*)	6	6	NS
Systolic blood pressure (mm Hg)	153.2 ± 3.1	153.9 ± 2.9	NS
Diastolic blood pressure (mm Hg)	86.8 ± 9.0	87.1 ± 8.8	NS
Serum creatinine (mgper 100 ml)	3.06 ± 0.24	2.98 ± 0.25	NS
eGFR (ml min^{-1} per 1.73 m^2)	22.1 ± 2.10	21.8 ± 1.80	NS
Urinary protein–Cr ratio (mg g^{-1})	3447 ± 405	3282 ± 403	NS
Na (mEq l^{-1})	139.9 ± 0.5	139.7 ± 0.5	NS
K (mEq l^{-1})	4.6 ± 0.07	4.6 ± 0.09	NS
Plasma glucose (mg per 100 ml)	128 ± 10.5	132 ± 8.7	NS
HbA1c (%) (for diabetes; *n*=11)	6.3 ± 0.12	6.3 ± 0.11	NS
Diagnosis of CKD (n)			
Diabetic nephropathy	11	11	NS
Chronic glomerulonephritis	6	7	NS
Hypertensive nephrosclerosis	5	5	NS
Tubulointerstitinal nephritis	1	1	NS
CKD stage (n)			
Stage 3	6	6	NS
Stage 4	8	9	NS
Stage 5	9	9	NS
Antihypertensive agents (n)			
Details of ARB			
Olmesartan 40 mg day^{-1}	12	13	NS
Telmisartan 80 mg day^{-1}	11	11	NS
ACE-inhibitor	8	9	NS
α-blocker	3	3	NS
β-blocker	1	2	NS
Diuretics	2	2	NS
Central sympatholytic agent	1	0	NS

Abbreviations: ACE, angiotensin converting enzyme; ARB, angiotensin receptor blocker; CKD, chronic kidney disease; Cr, creatinine; eGFR, estimated glomerular filtration rate; HbA1c, hemoglobin A1c; K, potassium; Na, sodium.

BP-lowering effect

Other antihypertensive agents listed in Table 1, such as ACE inhibitors and α-blockers, were administered throughout the trial period. Furthermore, antihypertensive drugs other than ARBs or CCBs were administered additionally to four patients from the A group and to five patients from the B group during the study period (A group: α-blocker, n=4; B group: furosemide, n=1; α-blocker, n=4).

The final doses were 7.6 ± 0.56 mg day^{-1} of amlodipine and 11.7 ± 0.87 mg day^{-1} of benidipine in the A and B groups, respectively. Figure 2 shows the changes in the systolic and diastolic BP. After treatment for 1 month, the systolic BP was significantly lower in the A group than in the B group, but no such difference was observed at other time points. Diastolic BP did not differ between the two groups during the course of treatment. In the last month of treatment, no differences were observed between systolic BP (A group: 133.4 ± 1.3 mm Hg, B group; 136.1 ± 1.8 mm Hg; not significant, NS) and diastolic BP levels (A group: 75.2 ± 1.6 mm Hg, B group: 78.6 ± 1.7 mm Hg; NS) in the patients in the two groups. Patients with BP <140/90 mm Hg accounted for 65.2 and 58.3% of the patients in the A and B groups (NS), respectively. The recommended BP target (130/80 mm Hg) was achieved in 43.4 and 37.5% of the total subjects of the A and B groups (NS), respectively. In both groups, the systolic and diastolic BP values were significantly lower than the baseline values at 1 month after initiation of the CCB therapy. The heart rate did not differ between the two groups (A group: 74.6 ± 2.3 beats min^{-1}, B group: 76.1 ± 2.2 beats min^{-1}, NS).

Renoprotective effects

As shown in Figure 3, the sCr levels were increased significantly in both groups after 24 weeks of treatment; however, the difference between the increase noted in both the groups was not significant. Furthermore, the eGFR levels were reduced significantly in both groups at the end of the study period. As shown in Figure 4, the urinary protein–Cr ratio in the last month was significantly lower in the B group than in the A group (2565 ± 299.9 *vs.* 3187 ± 372.2 mg per gram Cr, $P<0.05$). The percentage reduction in urinary protein excretion from the baseline showed a significant difference between

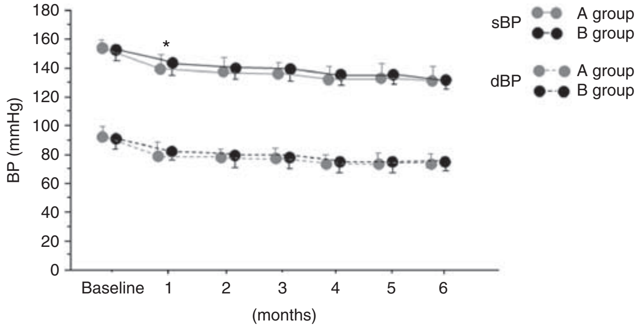

Figure 2 Changes in systolic and diastolic blood pressure during the study period. *$P<0.05$, A group *vs.* B group. BP, blood pressure; sBP, systolic blood pressure; dBP, diastolic blood pressure

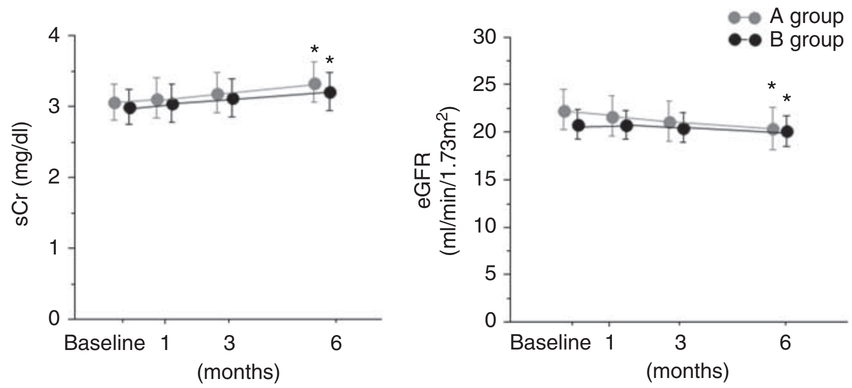

Figure 3 Changes in sCr and eGFR during the study period. *$P<0.05$ *vs.* baseline. sCr, serum creatinine; eGFR, estimated glomerular filtration rate.

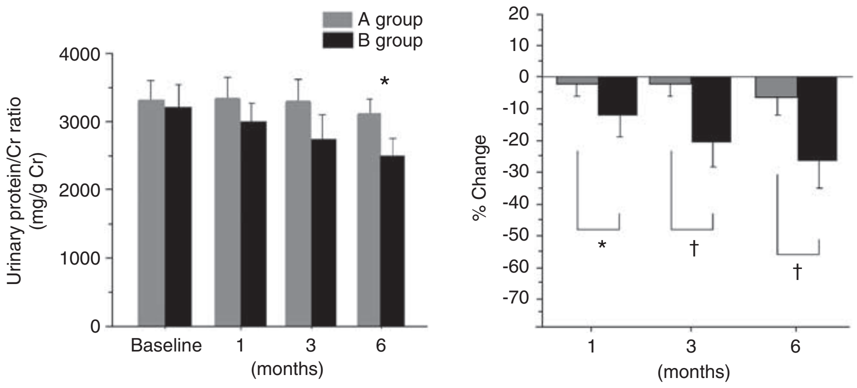

Figure 4 Changes in the urinary protein–Cr ratio and the respective percent changes from baseline. *$P<0.05$, †$P<0.01$, A group *vs.* B group. Cr, creatinine.

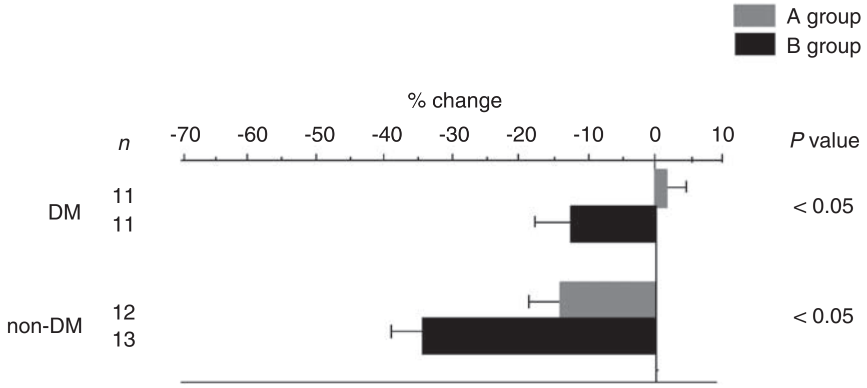

Figure 5 Percentage changes in the urinary protein–Cr ratio after 6 months of treatment in diabetic and non-diabetic patients with different baseline characteristics. Cr, creatinine; DM, diabetes mellitus; non-DM, non-diabetes mellitus.

the two treatment groups after 1 month of treatment and thereafter. Additionally, after 6 months of treatment, the percentage change from the baseline value decreased in the B group but not in the A group; the values were significantly different between the two groups (-29.4 ± 5.9 *vs.* $-7.8\pm6.9\%$, $P<0.05$). Even in a subgroup of patients with diabetic nephropathy, there was a significant difference in the percentage reduction in the urinary protein excretion from baseline values between the two treatment groups. In addition, even in the subgroup with non-diabetic subjects, benidipine caused a significant reduction in the urinary protein–Cr ratio as compared with that caused by amlodipine (Figure 5).

Adverse events

During the observation period, none of the patients in either group exhibited a significant increase in the occurrence of adverse effects, such as liver impairment, skin rash or frequent urination. Further-

more, none of the patients required renal replacement therapy for hyperkalemia or progressive loss of renal function during the observation period.

DISCUSSION

Treatment with an ACEI or ARB is recommended as the first-line therapy for achieving the target BP level in patients with CKD.[9,21,22] However, monotherapy with either of these drugs is effective in only a few patients; in most patients, two or three drugs with different mechanisms of action are required. The European Society of Hypertension/European Society of Cardiology recommends a combination therapy of either ARB/diuretics or ARB/CCB for the management of difficult-to-control hypertension. Recently, it was reported that the administration of a combination therapy with ARB and CCB exerted protective effects on organs in a rodent model.[23,24] Although CCBs and ARBs are considered first-line antihypertensive drugs in Japan, the rate of attaining the target BP level by the administration of monotherapy with either drug is ~12–40%.[25,26] According to a Japanese cross-sectional survey, the combination therapy with a CCB and an ARB is the most prescribed treatment for hypertensive patients in Japan.[26] Therefore, physicians frequently prescribe ARBs or ACEIs as first-choice drugs and CCBs as second-choice drugs to hypertensive patients who have CKD with proteinuria.

Recently, in the CARTER (Cilnidipine versus Amlodipine Randomized Trial for Evaluation in Renal Disease) study, it was reported that, when coupled with an RAS inhibitor, treatment with cilnidipine—an L-/N-type CCB—was superior to treatment with amlodipine for the prevention of the progression of proteinuria in hypertensive patients.[16] However, the types and dosages of ARBs or ACEIs were not reported in the study. In this study, we showed that benidipine is more beneficial than amlodipine as an additional medication for hypertensive patients with moderate-to-advanced-stage CKD associated with significant proteinuria (urinary protein–Cr ratio >300 mg per gram Cr), and who are under treatment with the current maximum recommended dose of ARBs.

In addition to RAS inhibition, a strict control of BP is considered to be important for preventing the progression of CKD.[21,22,27] In this study, the most consistent and statistically significant difference between the groups was in BP control: amlodipine was more efficacious in reducing BP than benidipine, particularly during the first 1 month of treatment. Furthermore, although the reduction in BP level brought about by benidipine and amlodipine was not statistically significant at the end of the study, BP levels tended to lower slightly in the amlodipine group than in the benidipine group (systolic–diastolic BPs were 133.4/75.2 *vs.* 136.1/78.6 mm Hg, A group *vs.* B group, respectively). Therefore, it might be possible that amlodipine, which is an L-type CCB, is beneficial in reducing systemic BP. However, the antiproteinuric effect of benidipine was more than that of amlodipine, suggesting that benidipine, in addition to lowering the BP, has beneficial effects. The CARTER study reported that the urinary protein–Cr ratio was lowered significantly after 12 months of treatment; however, in our study, it was reduced after 6 months of treatment. In another study, it was reported that a higher urinary protein excretion ($P<0.0001$), lower GFR ($P=0.0011$) and the presence of diabetes ($P=0.0284$) correlated with the reduction of urinary proteins when the treatment with amlodipine was replaced with treatment with benidipine.[13] The difference between our study and the CARTER study could be attributed to the large number of diabetic nephropathy cases included in our study. Moreover, the baseline proteinuria and sCr levels reflected a greater deterioration in patients in our study than in those of the CARTER study. These differences may explain why the antiproteinuric effect was detected earlier in our study than in the CARTER study. Furthermore, the patients in our study were treated with the current recommended maximum dose of ARBs for a considerably long time. Hence, the improvement in the glomerular hypertension on treatment with ARBs and benidipine resulted in a more pronounced antiproteinuric effect.

Some CCBs not only block the L-type calcium channels but also exert additional pleiotropic effects. CCBs that block either T-type or N-type calcium channels may exert renoprotective effects by dilating the efferent artery and protecting the glomerulus from hyperfiltration injury.[17,28] Benidipine, which blocks the L- and T-type calcium channels, decreases the resistance of the efferent and afferent arteries, and lowers the glomerular capillary hydrostatic pressure in rats.[29] Therefore, benidipine, as well as cilnidipine may exert renoprotective effects in hypertensive patients with CKD. The intraglomerular pressure is higher in patients with diabetic nephropathy than in patients with chronic glomerulonephritis or hypertensive nephroscler-eosis, and the number of patients with diabetic nephropathy in this study was high. Hence, it was considered that benidipine was more effective than amlodipine in the case of diabetic patients.

Our study is limited by the relatively small sample size and the short period of treatment. Moreover, although the prospective, randomized, open-label and parallel-groups comparison design was used in this study, more longitudinal, double-blind, comparative multicenter clinical trials should be conducted in a larger number of patients in order to further clarify the difference in the usefulness between the two agents; in addition to the BP profile, the percentage of renal replacement therapy requiring patients and other renal or cardiovascular events should also be considered as endpoints. In our study, we noted that benidipine significantly reduced BP and the severity of proteinuria even when administered for only a short duration. Therefore, we believe that this agent may be beneficial for hypertensive patients with stage 3–5 CKD.

In conclusion, the administration of benidipine, rather than amlodipine, ameliorates urinary protein excretion in hypertensive patients with moderate-to-advanced-stage CKD who are already being treated with ARBs. Therefore, we propose a combination therapy with benidipine and ARBs even for patients with advanced-stage CKD. Moreover, the administration of this combination therapy might lead to the delay of the initiation of renal replacement therapy.

CONFLICT OF INTEREST

The authors declare no conflict of interest.

1 Peterson JC, Adler S, Burkart JM, Greene T, Hebert LA, Hunsicker LG, King AJ, Klahr S, Massry SG, Seifter JL. The modification of diet in renal disease study group: blood pressure control, proteinuria, and the progression of renal disease. *Ann Intern Med* 1995; **123**: 754–762.
2 De Leeuw PW, Thijs L, Birkenhager WH, Voyaki SM, Efstratopoulos AD, Fagard RH, Leonetti G, Nachev C, Petrie JC, Rodicio JL, Rosenfeld JJ, Sarti C, Staessen JA, Systolic Hypertension in Europe (Syst-Eur) Trial Investigators. Prognostic significance of renal function in elderly patients with isolated systolic hypertension: results from the Syst-Eur trial. *J Am Soc Nephrol* 2002; **13**: 2213–2222.
3 Marin R, Gorostidi M, Fernandez-Vega F, Alvarez-Navascues R. Systemic and glomerular hypertension and progression of chronic renal disease: the dilemma of nephrosclerosis. *Kidney Int* 2005; **99**: S52–S56.
4 Viberti G, Wheeldon MN, MicroAlbuminuria Reduction with VALsartan (MARVAL) Study Investigators. Microalbuminuria reduction with valsartan in patients with type 2 diabetes mellitus: a blood pressure-independent effect. *Circulation* 2002; **106**: 672–678.
5 Lewis EJ, Hunsicker LG, Clarke WR, Berl T, Pohl MA, Lewis JB, Ritz E, Atkins RC, Rohde R, Raz I, Collaborative Study Group. Renoprotective effect of angiotensin-receptor antagonist irbesartan in patients with nephropathy due to type 2 diabetes. *N Engl J Med* 2001; **345**: 851–860.

6 Jafar TH, Stark PC, Schmid CH, Landa M, Maschio G, de Jong PE, de Zeeuw D, Shahinfar S, Toto R, Levey AS, AIPRD Study Group. Progression of chronic kidney disease: the role of blood pressure control, proteinuria, and angiotensin-converting enzyme inhibition, a patient-level meta-analysis. *Ann Intern Med* 2003; **139**: 244–252.
7 Weidmann P, Boehlen LM, Courten M. Effects of different antihypertensive drugs on human proteinuria. *Nephrol Dial Transplant* 1993; **8**: 582–584.
8 Lavermann GD, Henning RH, De Jong PE, Navis G, Zeeuw D. Optimal antiproteinuric dose of losartan in nondiabetic patients with nephrotic range proteinuria. *Am J Kidney Dis* 2001; **38**: 1381–1384.
9 Japanese Society of Hypertension. Japanese Society of Hypertension Guidelines for Management of Hypertension (JSH 2004). *Hypertens Res* 2006; **29**(Suppl): S1–S105.
10 Sheinfield GR, Bakris GL. Benefits of combination angiotensin-converting enzyme inhibitor and calcium antagonist therapy for diabetic patients. *Am J Hypertens* 1999; **12**: 80–85.
11 Waeber B. Managing hypertension in high-risk patients: lessons and promises from the STRATHE and ADVANCE trials. *J Hypertens* 2006; **24**: 19–27.
12 Hayashi K, Wakino S, Homma K, Sugano N, Saruta T. Pathophysiological significance of T-type Ca^{2+} channels: role of T-type Ca^{2+} channels in renal microcirculation. *J Pharmacol Sci* 2005; **99**: 221–227.
13 Ohishi M, Takagi T, Ito N, Terai M, Tatara Y, Hayashi N, Shiota A, Katsuya T, Rakugi H, Ogihara T. Renal-protective effect of T- and L-type calcium channel blockers in hypertensive patients: an amlodipine-to-benidipine changeover (ABC) study. *Hypertens Res* 2007; **30**: 797–806.
14 Furukawa T, Yamakawa T, Midera T, Sagawa T, Mori Y, Nukada T. Selectives of duhydropyridine derivatives in blocking Ca^{2+} channel subtypes expressed in Xenopus oocytes. *J Pharmacol Exp Ther* 1999; **291**: 464–473.
15 Hayashi K, Nagahama T, Oka K, Epstein M, Saruta T. Disparate effects of calcium antagonists on renal microcirculation. *Hypertens Res* 1996; **19**: 31–36.
16 Fujita T, Ando K, Nishimura H, Ideura T, Yasuda G, Isshiki M, Takahashi K, Cilnidipine versus Amlodipine Randomised Trial for Evaluation in Renal Desease(CARTER) Study Investigators. Antiproteinuric effect of the calcium channel blocker cilnidipine added to renin-angiotensin inhibition in hypertensive patients with chronic renal disease. *Kidney Int* 2007; **72**: 1543–1549.
17 Ishimitsu T, Kameda T, Akashiba A, Takahashi T, Ohta S, Yoshii M, Minami J, Ono H, Numabe A, Matsuoka H. Efonidipine reduces proteinuria and plasma aldosterone in patients with chronic glomerulonephritis. *Hypertens Res* 2007; **30**: 621–626.
18 Morikawa T, Okumura M, Konishi Y, Okada N, Imanishi M. Effects of benidipine on glomerular hemodyanamics and proteinuria in patients with nondiabetic nephropathy. *Hypertens Res* 2002; **25**: 571–576.
19 Saito F, Fujita H, Takahashi A, Ichiyama I, Harasawa S, Oiwa K, Takahashi N, Otsuka Y, Uchiyama T, Kanmatsuse K, Kushiro T. Renoprotective effect and cost-effectiveness of using benidipine, a calcium channel blocker, to lower the dose of angiotensin receptor blocker in hypertensive patients with albuminuria. *Hypertens Res* 2007; **30**: 39–47.
20 The Japanese Society of Nephrology. New eGFR equation for Japanese. Available at: http://www.jsn.or.jp/jsn_new/eng/index_e.html; accessed June 30, 2008.
21 Chobanian AV, Bakris GL, Black HR, Cushman WC, Green LA, Izzo Jr JL, Jones DW, Materson BJ, Oparil S, Wright Jr JT, Roccella EJ, National Heart, Lung, and Blood Institute Joint National Committee on Prevention, Detection, Evaluation, and Treatment of High Blood Pressure; National High Blood Pressure Education Program Coordinating Committee. The Seventh Report of the Joint National Committee on Prevention, Detection, Evaluation, and Treatment of High Blood Pressure: the JNC 7 report. *JAMA* 2003; **289**: 2560–2572.
22 European Society of Hypertension-European Society of Cardiology Guidelines Committee. 2003 European Society of Hypertension-European Society of Cardiology guidelines for the management of arterial hypertension. *J Hypertens* 2003; **21**: 1011–1053.
23 Kim-Mitsuyama S, Izumi Y, Izumiyz Y, Yoshida K, Yoshiyama M, Iwao H. Additive beneficial effects of the combination of calcium channel blocker and an angiotensin blocker on a hypertensive rat-heart failure model. *Hypertens Res* 2004; **27**: 771–779.
24 Suzuki J, Iwai M, Li Z, Li JM, Min LJ, Ide A, Yoshii T, Oshita A, Mogi M, Horiuchi M. Effect of combination of calcium antagonist, azelnidipine, and AT1 receptor blocker, olmesartan, on atherosclerosis in apolipoprotein E-deficient mice. *J Hypertens* 2005; **23**: 1383–1389.
25 Mancia G, Seravalle G, Grassi G. Tolerability and treatment compliance with angiotensin II receptor antagonists. *Am J Hypertens* 2003; **16**: 1066–1073.
26 Mori H, Ukai H, Yamamoto H, Saitou S, Hirao K, Yamauchi M, Umemura S. Current status of antihypertensive prescription and associated blood pressure control in Japan. *Hypertens Res* 2006; **29**: 143–151.
27 Mancia G, De Backer G, Dominiczak A, Cifkova R, Fagard R, Germano G, Grassi G, Heagerty AM, Kjeldsen SE, Laurent S, Narkiewicz K, Ruilope L, Rynkiewicz A, Schmieder RE, Boudier HA, Zanchetti A, Vahanian A, Camm J, De Caterina R, Dean V, Dickstein K, Filippatos G, Funck-Brentano C, Hellemans I, Kristensen SD, McGregor K, Sechtem U, Silber S, Tendera M, Widimsky P, Zamorano JL, Erdine S, Kiowski W, Agabiti-Rosei E, Ambrosioni E, Lindholm LH, Viigimaa M, Adamopoulos S, Agabiti-Rosei E, Ambrosioni E, Bertomeu V, Clement D, Erdine S, Farsang C, Gaita D, Lip G, Mallion JM, Manolis AJ, Nilsson PM, O'Brien E, Ponikowski P, Redon J, Ruschitzka F, Tamargo J, van Zwieten P, Waeber B, Williams B, Management of Arterial Hypertension of the European Society of Hypertension; European Society of Cardiology. 2007 Guidelines for the Management of Arterial Hypertension: The Task Force for the Management of Arterial Hypertension of the European Society of Hypertension (ESH) and of the European society of Cardiology (ESC). *J Hypertens* 2007; **25**: 1105–1187.
28 Yue W, Kimura S, Fujisawa Y, Tian R, Li F, Rahman M, Nishiyama A, Fukui T, Abe Y. Benidipine dilates both pre- and post-glomerular arteriole in the canine kidney. *Hypertens Res* 2001; **24**: 429–436.
29 Kawata T, Hashimoto S, Koike T. Diversity in the renal hemodynamic effects of dihydropyridine calcium blockers in spontaneously hypertensive rats. *J Cardiovasc Pharmacol* 1997; **30**: 431–436.
