# Supplementary material for: Effects of T-Type Calcium Channel Blockers on Renal Function and Aldosterone in Patients with Hypertension: A Systematic Review and Meta-Analysis
Source: PLoS One. 2014 Oct 17;9(10):e109834. doi: 10.1371/journal.pone.0109834 (PMC4201480; doi:10.1371/journal.pone.0109834)
Supplement: File S3 — PDF files of twenty-four studies included in the meta-analysis. (ZIP) [file pone.0109834.s007.zip › Supporting information-PDF files/22. Expert Rev Cardiovasc Ther 2008[6(10)]1347-1355.pdf]

For reprint orders, please contact reprints@expert-reviews.com

Expert Reviews

Add-on manidipine versus amlodipine in diabetic patients with hypertension and microalbuminuria: the AMANDHA study

Expert Rev. Cardiovasc. Ther. 6(10), 1347–1355 (2008)

Francisco Javier Martinez-Martin† and Margarita Saiz-Satjes

†Author for correspondence
Endocrinology and Nutrition Department, Hospital General de Gran Canaria Dr. Negrin, 35020 Las Palmas de Gran Canaria, Canary Islands, Spain
Tel.: +34 928 450 494
Fax: +34 928 202 919
dr.j.martinez@gmail.com

The aim of this study was to compare the efficacy and safety of adding manidipine 20 mg versus amlodipine 10 mg to the treatment of diabetic patients with uncontrolled hypertension and microalbuminuria despite full-dose treatment with a renin–angiotensin system blocker for at least 6 months. Patients were randomized to receive manidipine (n = 61) or amlodipine (n = 30) in a 2:1 ratio for 6 months and monitored for microalbuminuria for an additional extension phase of 18 months. Manidipine and amlodipine decreased blood pressure values to a similar extent. Urinary albumin excretion was reduced by 65.5% with manidipine versus 20% with amlodipine ($p < 0.01$) at 6 months and 62.7 versus 16.6% ($p < 0.01$) at the end of the extension phase. Manidipine was better tolerated than amlodipine. Thus, the addition of manidipine, but not amlodipine, resulted in a large reduction in the urinary albumin excretion rate despite similar blood pressure reductions.

Keywords: amlodipine • diabetes • hypertension • manidipine • microalbuminuria

Diabetes is associated with an increased risk of death from cardiovascular and cerebrovascular diseases [1]. In Spain, the prevalence of diabetes has been estimated at approximately 8% in women and 12% in men [2,3]. Owing to the increasing prevalence of obesity and sedentary lifestyles, the incidence of diabetes is expected to increase in coming years such that it is estimated there will be approximately 350 million people worldwide with diabetes by the year 2030 [4].

Diabetes is itself a major cardiovascular risk factor and its presence increases the risk for other risk factors, particularly hypertension. Hypertension affects approximately 50–80% of patients with Type 2 diabetes and the concomitance of these conditions increases the risk of patients presenting with cardiovascular outcomes [5]. The United Kingdom Prospective Diabetes Study demonstrated that diabetes and hypertension were independent and additive risk factors for cardiovascular disease [6].

Guidelines recommend a blood pressure (BP) goal of less than 130/80 mmHg for diabetics [7–9]. Notwithstanding this recommendation, available data suggest that more intensive treatment resulting in lower BP values confers greater protection against vascular complications [10]. It has been estimated that over 60% of patients with hypertension and diabetes require at least two antihypertensive agents to achieve BP goals. However, only approximately 12% of hypertensive patients with diabetes achieve their BP objectives [11–13].

Approximately 50% of patients with Type 2 diabetes will develop microalbuminuria within 10 years from first diagnosis of the disease. Without specific treatment, 20–40% of these patients will progress to nephropathy and eventually to end-stage renal disease [14]. The presence of microalbuminuria is the most important predictive sign of renal damage [15]. Inhibition of the renin–angiotensin system is thus a logical first-line treatment in the management of patients with diabetes and hypertension, particularly those with microalbuminuria [7–9].

Calcium channel blockers represent a good option to combine with renin–angiotensin

 10.1586/14779072.6.10.1347 © 2008 Expert Reviews Ltd ISSN 1477-9072

system inhibitors [9]. However, not all calcium channel blockers have the same effects. Although dihydropyridines can achieve a significant reduction in BP, most have not been able to consistently demonstrate a reduction in urinary albumin excretion. This finding may be related to the selective relaxation of afferent arterioles, with a relative increase in intraglomerular pressure. Newer dihydropyridines, such as manidipine, that have a selective affinity for efferent arterioles can be expected to overcome this drawback [16–18].

The aim of the Efficacy and Safety Assessment of Manidipine in Type 2 Diabetic Patients with Hypertension and Microalbuminuria Uncontrolled with Renin–Angiotensin System Blockers (AMANDHA) study was to compare the efficacy and safety of adding manidipine 20 mg or amlodipine 10 mg once daily to the treatment of diabetic patients with uncontrolled hypertension and microalbuminuria despite full-dose treatment with a renin–angiotensin system blocker (e.g., angiotensin-converting enzyme inhibitors [ACE-Is] or angiotensin receptor blockers [ARBs]) for at least 6 months. In addition, treatment compliance and effects on urinary albumin excretion and sympathetic tone (estimated by metanephrine and normetanephrine excretion) were also analyzed.

Methods

A total of 91 patients (age > 30 years), with Type 2 diabetes, hypertension and persistent microalbuminuria, who had been treated for at least 6 months with a renin–angiotensin system inhibitor at the highest recommended doses were studied. To be included, patients needed to demonstrate good compliance with treatment (>80%), good tolerability to therapy, insufficient BP control (BP > 130/80 mmHg) despite treatment with ACE-Is or ARBs, and increased urinary albumin excretion (>20 µg/min), but not overt-proteinuria (>200 µg/min). Other antihypertensive agents, such as diuretics and sympathetic system blockers, were only permitted when the patient had been taking them previously; no dosage changes were allowed during the study. Other calcium channel blockers or ACE-I plus ARB combinations were not allowed. Exclusion criteria were:

- Known hypersensitivity or history of severe adverse effects to any renin–angiotensin system inhibitor or calcium channel blocker;
- Evidence of unstable angina or decompensated congestive heart failure;
- Myocardial infarction within the previous 30 days; left ventricular outflow obstruction;
- Liver dysfunction (>twofold increase in serum aminotransferases or >1.5-fold increase in serum bilirubin above the upper limit of normal);
- Renal insufficiency (serum creatinine concentration >1.5 mg/dl [>133 µmol/l] in men and >1.4 mg/dl [>124 µmol/l] in women]; hyperkalemia >5 meq/l;
- Any contraindication for prescribing manidipine or amlodipine, as stated in the drug technical form;
- Pregnant women, nursing mothers or women of childbearing potential not using adequate methods of contraception were also excluded.

The study was conducted according to Good Clinical Practice guidelines and was approved by the local ethics committee. All participants provided written informed consent before taking part in the study.

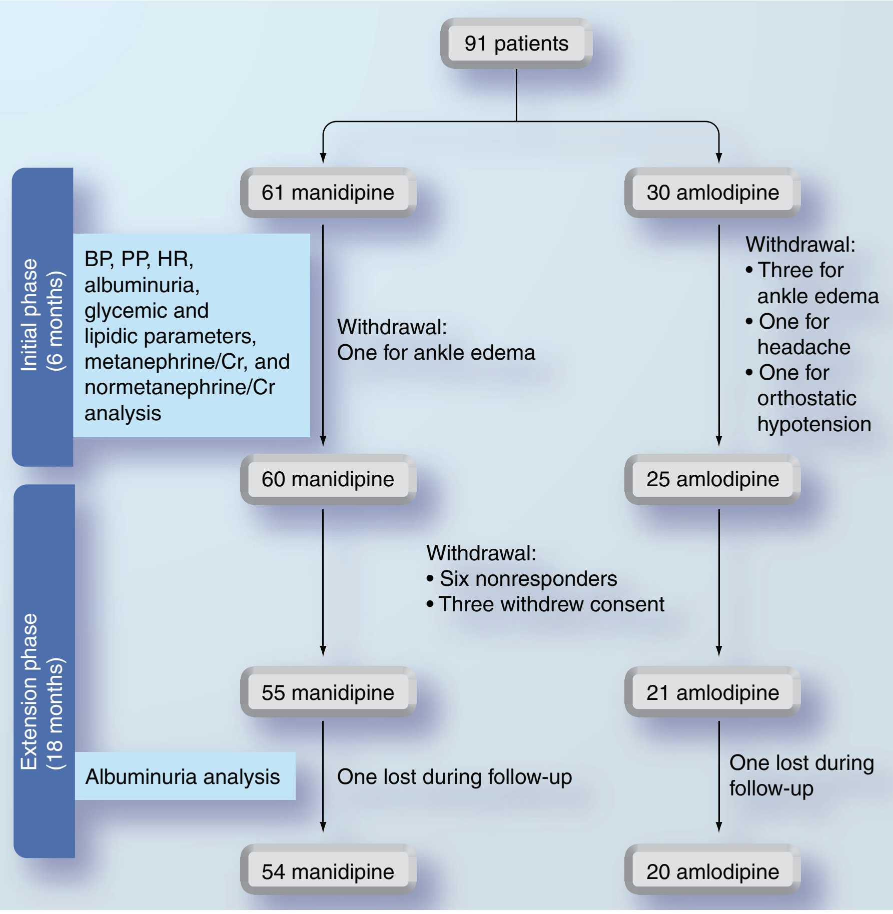

Figure 1. Study flow chart. A total of 91 patients were randomized to receive manidipine 20 mg/day (n = 61) or amlodipine 10 mg/day (n = 30) in a 2:1 ratio. During the initial phase (6 months), BP, PP, HR, albuminuria, glycemic and lipidic parameters, metanephrine/Cr, and normetanephrine/Cr analysis were recorded. Patients who completed the first phase of the study were asked to participate in a further 18-month extension phase. Urinary albumin excretion was measured in these patients after 24 ± 2 weeks and at the end of the extension phase.
BP: Blood pressure; Cr: Creatinine; HR: Heart rate; PP: Pulse pressure.

The study was conducted using a prospective randomized open blinded end point (PROBE) design [19]. Patients were randomly allocated to receive manidipine 20 mg/day (n = 61) or amlodipine 10 mg/day (n = 30) in a 2:1 ratio. Patients' previous antihypertensive therapy was maintained without changes in dosage or type of drugs. At baseline and at each visit (weeks 0, 12 and 24), biodemographic data (i.e., weight, height, BP and heart rate), concomitant treatments, plasma fasting glucose, glycosylated hemoglobin, ions, lipid profile (standard) and urinary albumin excretion (geometric mean of three overnight collections) were recorded. At 12 ± 2 weeks and 24 ± 2 weeks follow-up, compliance and tolerability were also measured, using pill counting and a questionnaire, respectively. At visit 0 and after 24 ± 2 weeks of follow-up, metanephrine/creatinine and normetanephrine/creatinine levels in overnight urine were determined. Patients who completed the first phase of the study after 24 ± 2 weeks of follow-up were asked to continue in the study for a further 18-month extension phase. Urinary albumin excretion was measured in these patients after 24 ± 2 weeks and at the end of the extension phase.

Table 1. Baseline characteristics*.

	Manidipine	Amlodipine	p-value
Women (%)	60.0	56.7	NS
Age (years)	56.9 ± 13.3	55.8 ± 12.7	NS
Height (cm)	167.3 ± 12.0	165.8 ± 11.9	NS
Weight (kg)	80.1 ± 12.9	79.8 ± 11.5	NS
Systolic blood pressure (mmHg)	159 ± 14	158 ± 17	NS
Diastolic blood pressure (mmHg)	96 ± 12	95 ± 13	NS
Albumin excretion rate (µg/min)	109 ± 54	112 ± 57	NS
Heart rate (bpm)	77 ± 9	75 ± 11	NS
Glycemia (mmol/l)	10.3 ± 4.5	9.9 ± 3.8	NS
HbA_{1c} (%)	8.1 ± 1.1	8.2 ± 1.0	NS
PlCr (µmol/l)	96.4 ± 23.9	91.1 ± 29.2	NS
Na (meq/l)	138 ± 3	138 ± 4	NS
K (meq/l)	4.22 ± 0.44	4.25 ± 0.37	NS
Total cholesterol (mmol/l)	6.06 ± 0.98	6.19 ± 1.06	NS
HDL-cholesterol (mmol/l)	1.15 ± 0.20	1.14 ± 0.20	NS
Triglycerides (mmol/l)	2.14 ± 0.67	2.20 ± 0.70	NS

*At baseline, no significant differences were found between both groups in the clinical characteristics.
HbA1C: Glycosylated hemoglobin; K: Potassium; Na: Sodium; NS: Not significant; PlCr: Plasma creatinine.

Office BP was measured according to current guidelines using validated automatic devices. BP was recorded with the patient in a sitting position and with the back supported, after resting for 5 min. Patients were advised to avoid smoking or drinking coffee for 30 min prior to BP assessment. The BP value obtained at each visit was the average of two measurements separated by at least 2 min. A third measure was obtained when there was a difference of 5 mmHg or more between the two readings. Adequate BP control was defined as less than 130/80 mmHg [7].

Statistical analysis

An intention-to-treat analysis was performed for patients who had received at least one dose of study medication and had a subsequent efficacy observation. Results were presented as the mean ± standard deviation. BP and heart rate were analyzed by analysis of variance for repeated measures. Changes from baseline and between-group differences in laboratory parameters were evaluated using a Student's t-test for paired and unpaired data. A chi-squared test or a Fisher's exact test was used to compare categorical variables as appropriate. Pearson's test was used for linear correlation (R). All tests were two-tailed and p-values that were less than 0.05 were considered to indicate statistical significance.

Results

The flow chart of the study is shown in Figure 1. At baseline, 91 patients (61 in manidipine group and 30 in amlodipine group) were included in the study. During the initial phase of 24 weeks, six patients (one vs five patients, respectively) withdrew from the study because of adverse effects. A total of 85 patients began the extension phase and 74 of these patients (54 vs 20, respectively) completed the study. The reasons for discontinuation are indicated in Figure 1.

Baseline characteristics are expressed in Table 1 and concomitant medications in Table 2. No significant differences were found between groups at baseline in terms of biodemographic data and concomitant medications. A total of 47% of patients were taking an ACE-I and the remaining 53% an ARB. Only 36% of the study population were receiving monotherapy. In total, 51% were taking diuretics and 13% adrenergic blockers. With regard to other medications, 66% were taking statins, 7% fibrates, 55% aspirin and 15% clopidogrel. Use of oral antidiabetics and insulin was modified during the study according to local practice.

Throughout the study, manidipine and amlodipine decreased mean BP values by a similar extent (-11.5 mmHg for both treatments, $p < 0.001$ vs baseline, p = not significant [NS] between groups). However, significant differences were noted when decreases in systolic and diastolic BP were compared. Reductions in systolic BP were greater with manidipine (19.1 vs 12.7 mmHg; $p = 0.006$) and in diastolic BP with amlodipine (7.7 vs 10.9 mmHg; $p = 0.011$). A total of 23.3% of patients treated with manidipine achieved their BP targets versus 20.0% of patients treated with amlodipine (p = NS).

Table 2. Medications at baseline*.

Drug	Patients receiving mandipine (n)	Patients receiving amlodipine (n)
Renin–angiotensin system blockers		
Irbesartan	9	4
Valsartan 160 mg	5	2
Candesartan 16 mg	5	2
Losartan 100 mg	5	2
Eprosartan 600 mg	3	2
Telmisartan 80 mg	2	1
Fosinopril 20 mg	10	5
Enalapril 20 mg	10	4
Ramipril 10 mg	6	2
Trandolapril 2 mg	5	1
Antihypertensive therapy		
Diuretics	29	14
Adrenergic blockers	8	3
None	23	8
Hypolipemiant therapy		
Statins	40	16
Fibrates	3	3
None	17	6
Antiaggregation therapy		
Acetylsalicylic acid	34	13
Clopidogrel	11	5
None	15	7

p = NS (x^2).
*At baseline, no significant differences were found between both groups in the treatments received.
A: Amlodipine; M: Manidipine.

Pulse pressure was only reduced in the manidipine group (11.3 vs 1.8 mmHg, p < 0.001 manidipine vs baseline and between groups; p = NS amlodipine vs baseline). At baseline, 63.3% of patients allocated to manidipine had a high pulse pressure (>60 mmHg). From this population, 68.4% attained a pulse pressure of under 60 mmHg at study end (p = 0.001 between groups). 60.0% of patients treated with amlodipine had a high pulse pressure at baseline. At the end of follow-up, only 13.3% achieved a pulse pressure of less than 60 mmHg.

Heart rate increased by 5.6 bpm with amlodipine (p=0.011 vs baseline), whereas no significant differences were found with manidipine (-1.2 bpm; p = NS vs baseline, p = 0.011 vs amlodipine). This is in accordance with the changes noted in urinary metanephrine and normetanephrine excretion rates (Figure 2). Urinary metanephrine and normetanephrine levels increased in the amlodipine group, whereas no changes were found in the manidipine group. Significant correlations were found between both metanephrine excretion and pulse pressure (Pearson's R = 0.300; p = 0.005); normetanephrine excretion and pulse pressure (R = 0.315; p = 0.003); metanephrine excretion and heart rate (R = 0.222; p = 0.041), and normetanephrine excretion and heart rate (R = 0.228; p = 0.036).

The 95% of patients treated with manidipine were considered to be good compliers (i.e., took >80% of study medication). During the initial phase, one patient withdrew because of mild ankle edema. Other adverse effects attributable to treatment were hypotension-related (e.g., headache, dizziness); these occurred in four patients but were mild and transient and did not lead to treatment withdrawal. A total of 60 patients (98%) completed 12 weeks of follow-up with a compliance rate of over 50%, of which 58 (95%) had a compliance rate of over 80% (both p < 0.05 vs amlodipine).

A total of 92% of patients treated with amlodipine were considered to be good compliers (i.e., took >80% of study medication). During the first part of the study, three patients withdrew because of mild-to-moderate ankle edema, one patient withdrew because of severe headache and flushing and one patient withdrew because of orthostatic hypotension. An additional three patients had mild ankle edema but did not withdraw from the study. Other adverse effects reported were hypotension-related effects (e.g., mild headache and dizziness), seen in three patients. These were transient and did not lead to treatment withdrawal. In total, 25 patients (83%) completed 12 weeks' follow-up with a compliance rate of over 50%, 23 (77%) of which had a compliance rate of over 80%.

No serious adverse events were recorded in either group. However, discontinuation of study medication due to adverse effects was more frequent with amlodipine than with manidipine (16.7 vs 1.6%; p = 0.014). The frequency of adverse effects not leading to discontinuation of study drug was also higher in the amlodipine group (20 vs 6.6%; p = 0.03). The presence of ankle edema was less frequent with manidipine (1.6 vs 20%; p = 0.001). No other significant differences were found with respect to adverse effects.

The results of urinary albumin excretion are shown in Figure 3. During the initial phase, the amlodipine-treated group showed a trend towards a reduction in urinary albumin excretion rates (-14.9% at week 12 and -20% at week 24; p = NS vs baseline). By contrast, in the group treated with manidipine, there were significant decreases in urinary albumin excretion rates throughout this phase (-54.0% at week 12 and -65.5% at week 24; p < 0.001 vs baseline and p < 0.01 vs amlodipine at each time). Similarly, during the extension phase, no significant decreases were found in the amlodipine-treated group (-16.9% at week 52 and -16.6% at week 104; p = NS vs baseline). But, in the group treated with manidipine, there were significant reductions in urinary albumin excretion rates during the extension phase (-63.4% at week 52 and 62.7% at week 104; p < 0.001 vs baseline and p < 0.01 vs amlodipine at each time).

The 38.3% of patients treated with manidipine achieved albuminuria targets (< 20 µg/min) versus only 8% with amlodipine (p = 0.004). No patients treated with manidipine versus 8% treated with amlodipine developed a urinary albumin excretion

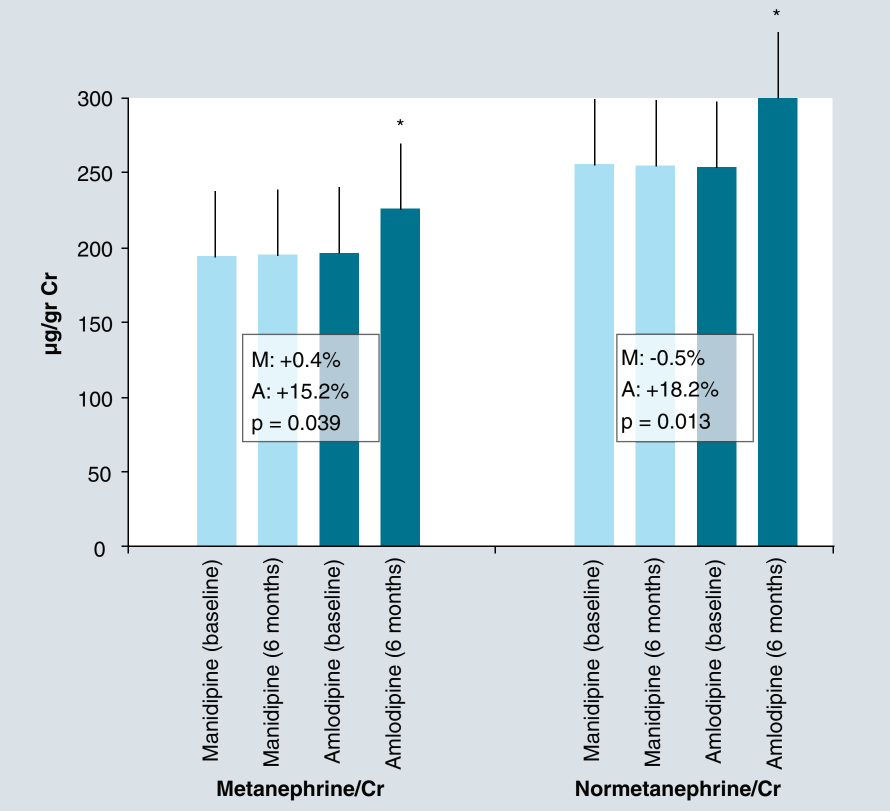

Figure 2. Urinary metanephrine and normetanephrine excretion rates. Metanephrine/Cr (left cluster) and normetanephrine/Cr (right cluster) at baseline and after 6-month treatment.
*p < 0.001 versus baseline and p < 0.05 between treatments.
A: Amlodipine; Cr: Creatinine; M: Manidipine.

rate of over 200 µg/min. These results were independent of initial treatment with ACE-I or ARB. The linear regression model is presented in Figure 4 (percentage decrease of albumin excretion plotted against mean blood pressure reduction). The figure shows that in amlodipine-treated patients, a large reduction in BP is needed in order to provide some benefit in terms of urinary albumin excretion, while in their manidipine-treated counterparts, a very relevant albuminuria reduction is achieved at whatever BP reduction level obtained. Considering that the mean BP reduction is similar for both groups, the effect of amlodipine on microalbuminuria is exclusively dependent on BP reduction, while manidipine elicits an additional, non-BP-dependent antialbuminuric effect. The result is a superior reduction of albumin excretion with manidipine as compared with amlodipine, throughout all the range of BP reductions attained. Additional linear regression analyses were performed for the percentage decrease of albumin excretion against the pulse pressure and systolic BP reductions. For pulse pressure, no statistical significance was found, ruling out a relevant independent contribution of the observed pulse pressure reduction to the anti-albuminuric effect of the treatments. For systolic BP, the correlation was weaker than for mean BP (Pearson's R coefficients were 0.39 and 0.75, respectively, with $p = 0.007$ and $p < 0.001$).

Discussion

Most diabetics with hypertension will need at least two antihypertensive agents to achieve BP targets [11–13]. It is well-established that renin–angiotensin system inhibitors are a useful first-line treatment in this patient population [7–9]. Combining ACE-Is or ARBs with a calcium channel blocker appears to be one of the best approaches in these patients. More specifically, the combination of a renin–angiotensin system inhibitor and dihydropyridine is particularly effective owing to complementary mechanisms that enhance antihypertensive efficacy while being associated with a low incidence of adverse effects [20].

Dihydropyridines are potent vasodilators that induce reflex activation of the renin–angiotensin and sympathetic systems. Concomitant use of ACE-Is or ARBs may buffer this excessive activation. Moreover, because calcium channel blockers promote an increase in angiotensin II levels and a negative sodium balance, this may strengthen the antihypertensive effect of renin–angiotensin system inhibitors [21]. Using both treatments concomitantly may decrease the presence of adverse effects, mainly peripheral edema [22]. Mechanistically, the development of lower extremity edema during calcium entry blockade is due to an increase in intracapillary pressure as a result of selective increase in the postcapillary tone, which is in turn due to sympathetic activation. Notably, renin–angiotensin

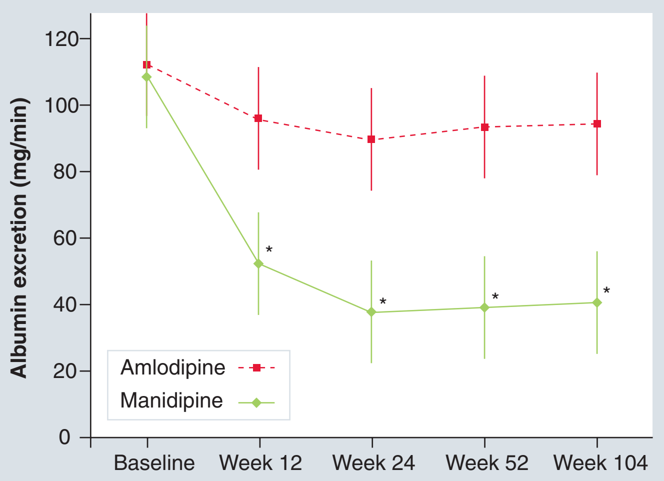

Figure 3. Urinary albumin excretion. Changes in urinary albumin excretion during 2 years of treatment with manidipine (solid line line, diamonds) versus amlodipine (dashed line, squares) on top of the maximum reccommended dose of a renin–angiotensin system blocker in Type 2 diabetes patients with uncontrolled hypertensive and persistent microalbuminuria.

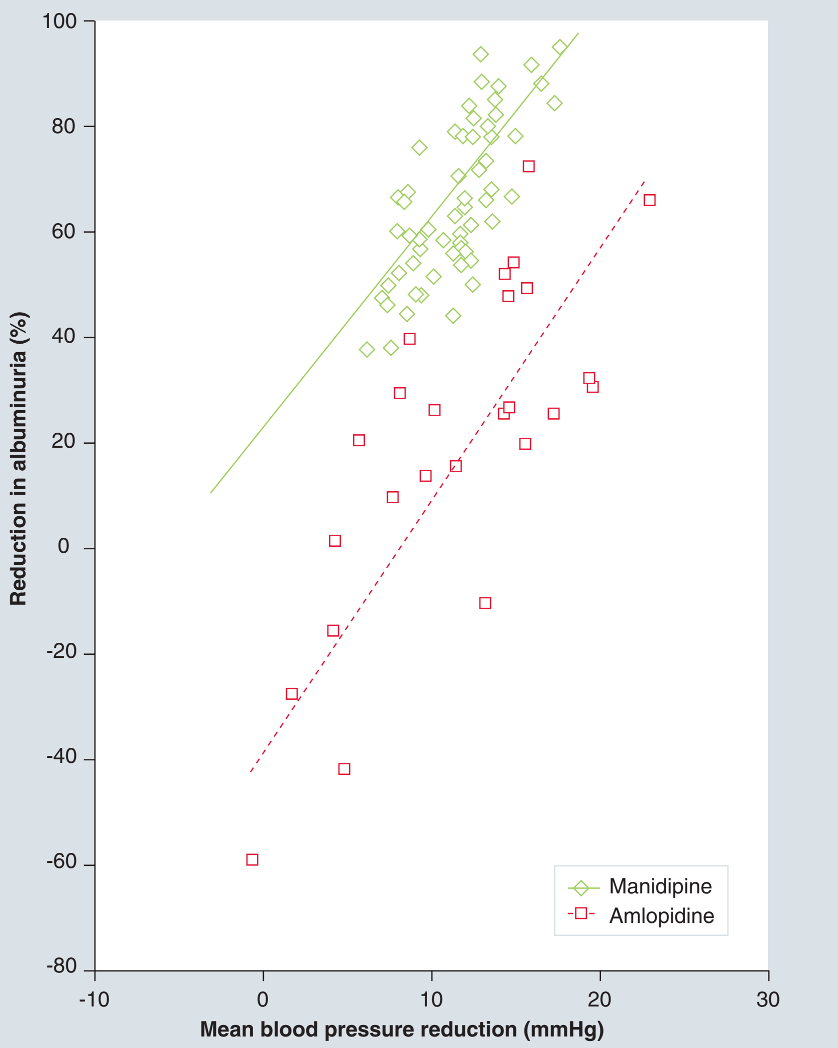

Figure 4. Scatter plot and linear regression analysis. Percentage reduction in albuminuria against absolute reduction in mean blood pressure (mmHg) after 24 weeks of treatment with manidipine (solid line, diamonds) versus amlodipine (dashed line, squares). The reduction in albumin excretion is significantly superior with manidipine throughout all the attained mean blood pressure reduction range.

system inhibitors reduce the lower extremity edema caused by dihydropiridines, most likely because of their ability to dilate both the arterial vascular bed and venous capacitance vessels [21].

Microalbuminuria is an independent predictor of mortality in hypertensive patients with diabetes, but it is not clear whether calcium channel blockers are able to reduce urinary albumin excretion beyond their capacity to decrease BP values [23–25]. Amlodipine, a long-acting hydrophilic dihydropiridine, is a potent antihypertensive agent and one of the most frequently prescribed calcium channel blockers in clinical practice [26]. Manidipine is a third-generation dihydropyridine calcium antagonist that causes systemic vasodilation by inhibiting the voltage-dependent calcium inward currents in smooth muscle cells.

This study compared the efficacy and safety of adding either manidipine or amlodipine to the treatment of diabetic patients with uncontrolled hypertension and microalbuminuria despite full-dose treatment with a renin–angiotensin system blocker. The study demonstrated that, despite a similar effect on BP values, manidipine attained a reduction of 62.7 versus 16.6% with amlodipine on urinary albumin excretion. Considering that microalbuminuria is an independent predictor of mortality in these patients, it seems clear that a greater reduction in urinary albumin excretion may translate to better prognosis in this high-risk patient population. The primary reason for this difference is the ability of manidipine to block T-type calcium channels.

Most dihydropyridines block only L-type calcium channels; this produces peripheral vasodilatation and leads to a decrease in vascular resistance. However, some important vascular beds, such as the efferent arterioles, lack L-type receptors. As a result, these dihydropyridines may cause glomerular hypertension and, secondarily, cause an increase in urinary albumin excretion. T-type calcium channel receptors are present in both afferent and efferent arterioles. Manidipine blocks both L- and T-type receptors and, therefore, has a more specific effect on intraglomerular pressure compared with other dihydropyridines. Through this mechanism, manidipine may reduce urinary albumin excretion rates beyond what would be expected with BP reduction alone [18,27,28].

Robust available evidence shows that calcium channel blockers are potent antihypertensive agents [7–9]. Many trials have reported that manidipine is an effective antihypertensive drug when used as monotherapy or in combination treatment [29–35]. Although global BP reductions were similar in both groups, reductions in pulse pressure were greater in patients treated with manidipine. Several studies have reported that pulse pressure is a predictor of cardiovascular outcomes in hypertensive patients and, importantly, also in high-risk populations such as those with diabetes or metabolic syndrome [36,37]. This supports the added value of manidipine in the treatment of patients with hypertension and diabetes.

Tolerability is a key factor in the treatment of chronic diseases. A good tolerability profile implies lower withdrawal rates, indicating satisfactory patient compliance and, in turn, better control of BP [38]. The results of our study clearly show that manidipine is better tolerated than amlodipine. In fact, the discontinuation of study medication due to adverse effects (16.7 vs 1.6%; $p = 0.014$), the frequency of adverse effects that did not result in discontinuation of study drug (20 vs 6.6%; $p = 0.03$) and the presence of ankle edema (20 vs 1.6%; $p = 0.001$) were less frequent with manidipine.

Another important issue regarding safety is the potential effect of dihydropyridines on heart rate [18]. Our study showed that amlodipine, but not manidipine, increases heart rate through activation of the sympathetic system. Urinary metanephrine and normetanephrine levels were increased in the amlodipine group, whereas no changes were found in the manidipine group.

It is possible that the PROBE design may have introduced investigator bias; however, this design has been used in numerous trials to date and is thought to more closely reflect clinical practice than a randomized controlled design [39].

In conclusion, the addition of manidipine to the treatment of patients with diabetes and hypertension who were already being treated with an ACE-I or ARB was effective and well-tolerated. The addition of manidipine, but not amlodipine, resulted in a large reduction in the urinary albumin excretion rate. Previous studies in patients with chronic renal failure have also shown more favorable results on proteinuria with manidipine monotherapy compared with amlodipine therapy, in spite of a similar reduction in BP [40]. This supports the added value of manidipine beyond reduction of BP in the treatment of patients with diabetes and hypertension.

Expert commentary & five-year view

Diabetes and hypertension are independent and additive risk factors for cardiovascular disease. Current guidelines recommend a BP goal of less than 130/80 mmHg for diabetics. For this purpose, the majority of diabetics will need at least two antihypertensive agents to achieve BP targets. Moreover, approximately 50% of patients with Type 2 diabetes will develop microalbuminuria within 10 years of first diagnosis of the disease, further worsening the prognosis of these patients. Although all antihypertensive drugs reduce BP values, only some of them have additional benefits beyond BP reductions. Calcium channel blockers represent a good option to combine with renin–angiotensin system inhibitors, the first-line treatment in this patient population. This study clearly demonstrates that the addition of manidipine, but not amlodipine, to the treatment of patients already treated with a full-dose of renin–angiotensin system inhibitors, results in a large reduction in the urinary albumin excretion rate, regardless of mean BP reduction. The primary reason for this difference is the ability of manidipine to block T-type calcium channels. As a result of this particular property of manidipine, it is likely that, in the next few years, manidipine will be one of the dihydropiridines most frequently used in combination with renin–angiotensin system inhibitors for the treatment of diabetics with hypertension.

Financial & competing interests disclosure

FJ Martinez Martin has received lecture fees from Chiesi, Pfizer, Menarini, Daiichi-Sankyo, Bayer, Lilly, Novo-Nordisk Sanofi-Aventis, Bristol-Myers-Squibb, Abbot, Novartis and others, and also grant funding from Pfizer, but none directly related to this study. The authors have no other relevant affiliations or financial involvement with any organization or entity with a financial interest in or financial conflict with the subject matter or materials discussed in the manuscript apart from those disclosed.

Writing assistance was utilized in the production of this manuscript. Editorial assistance was provided by Content Ed Net Communications, Madrid, Spain, with funding from Chiesi Pharma.

Key issues

- Diabetes is associated with an increased risk of death from cardiovascular and cerebrovascular diseases. Owing to the increasing prevalence of obesity and sedentary lifestyles, the incidence of diabetes is expected to increase in coming years.
- The presence of diabetes increases the risk for other risk factors, particularly hypertension. Diabetes and hypertension are independent and additive risk factors for cardiovascular disease.
- Current guidelines recommend a blood pressure (BP) goal of less than 130/80 mmHg for diabetics.
- For this purpose, a majority of diabetic patients will need at least two antihypertensive agents to achieve BP targets. However, only approximately 12% of hypertensive patients with diabetes achieve their BP objectives.
- Moreover, approximately 50% of patients with diabetes will develop microalbuminuria within 10 years of first diagnosis of the disease, further worsening the prognosis of these patients.
- Although all antihypertensive drugs reduce BP values, only some of them have additional benefits beyond BP reductions. In particular, renin–angiotensin system blockers have specific, non-BP-reduction dependent benefits in diabetic nephropathy that have been proved in large studies such as Collaborative Study Group [CSG], Reduction of Endpoints in Niddm with the Angiotensin II Antagonist (RENAAL), Irbesartan Diabetic Nephropathy Trial (IDNT), African–American Study of Kidney disease and Hypertension (AASK) and many others. All present guidelines recommend these drugs as first-line treatment in this patient population.
- However, monotherapy is usually inadequate and the obvious question about the best add-on treatment remains. Calcium channel blockers represent a good option to combine with renin–angiotensin system inhibitors, having attained excellent results in large trials such as Systolic Hypertension in Europe trial (SYST-EUR) and Anglo-Scandinavian Cardiac Outcomes Trial (ASCOT).
- The Efficacy and Safety Assessment of Manidipine in Type 2 Diabetic patients with Hypertension and Microalbuminuria uncontrolled with renin–angiotensin system blockers study demonstrates that the addition of manidipine, but not amlodipine, to the treatment of this patient population results in a large reduction in the urinary albumin excretion rate, independently of BP reduction.
- The primary reason for this difference is the ability of manidipine to block T-type calcium channels.
- Manidipine was better tolerated than amlodipine. In fact, the discontinuation of study medication owing to adverse effects, the frequency of adverse effects not resulting in discontinuation of study medication and the presence of ankle edema were less frequent with manidipine.
- Manidipine is an effective and well-tolerated drug in the treatment of patients with diabetes and hypertension.

References

1 Dobesh PP. Managing hypertension in patients with Type 2 diabetes mellitus. *Am. J. Health. Syst. Pharm.* 63(12), 1140–1149 (2006).

2 Medrano MJ, Cerrato E, Boix R, Delgado-Rodríguez M. Cardiovascular risk factors in Spanish population: metaanalysis of cross-sectional studies. *Med. Clin. (Barc).* 124(16), 606–612 (2005).

3 de Pablos-Velasco PL, Martínez-Martín FJ, Molero R *et al.* Patterns of prescription of hypoglycaemic drugs in Gran Canaria (Canary Islands, Spain) and estimation of the prevalence of diabetes mellitus. *Diabetes Metab.* 31(5), 457–462 (2005).

4 Wild S, Roglic G, Green A *et al.* Global prevalence of diabetes; estimates for the year 2000 and projections for 2030. *Diabetes Care* 21(5), 1047–1053 (2004).

5 Landsberg L, Molitch M. Diabetes and hypertension: pathogenesis, prevention and treatment. *Clin. Exp. Hypertens.* 26(7–8), 621–628 (2004).

6 Stratton IM, Cull CA, Adler AI *et al.* Additive effects of glycaemia and blood pressure exposure on risk of complications in Type 2 diabetes: a prospective observational study (UKPDS 75). *Diabetologia* 49(8), 1761–1769 (2006).

7 European Society of Hypertension–European Society of Cardiology Guidelines Committee. 2003 European Society of Hypertension–European Society of Cardiology guidelines for the management of arterial hypertension. [Errata in *J. Hypertens.* 21, 2203–2204 (2003) and *J. Hypertens.* 22, 435 (2004)] *J. Hypertens.* 21(6), 1011–1053 (2003).

8 Chobanian AV, Bakris GL, Black HR *et al.* The seventh report of the joint national committee on prevention, detection, evaluation, and treatment of high blood pressure: the JNC 7 report. *JAMA* 289(19), 2560–2572 (2003).

9 Mancia G, De Backer G, Dominiczak A *et al.* 2007 guidelines for the management of arterial hypertension: the task force for the management of arterial hypertension of the European Society of Hypertension (ESH) and of the European Society of Cardiology (ESC). [Erratum in *J. Hypertens.* 25, 1749 (2007)] *J. Hypertens.* 25(9), 1105–1187 (2007).

10 Turnbull F; Blood Pressure Lowering Treatment Trialists' Collaboration. Effects of different blood-pressure-lowering regimens on major cardiovascular events: results of prospectively-designed overviews of randomised trials. *Lancet* 362 (9395), 1527–1535 (2003).

11 Franklin SS, Neutel JM. Initial combination therapy for rapid and effective control of moderate and severe hypertension. *J. Hum. Hypertens.* DOI:10.1038/jhh.2008.72 (Epub ahead of print) (2008).

12 McLean DL, Simpson SH, McAlister FA, Tsuyuki RT. Treatment and blood pressure control in 47,964 people with diabetes and hypertension: a systematic review of observational studies. *Can. J. Cardiol.* 22(10), 855–860 (2006).

13 Banegas JR, Segura J, Ruilope LM *et al.* Blood pressure control and physician management of hypertension in hospital hypertension units in Spain. *Hypertension* 43(6), 1338–1344 (2004).

14 Weir MR. Microalbuminuria in Type 2 diabetics: an important, overlooked cardiovascular risk factor. *J. Clin. Hypertens.* 6(3), 134–141 (2004).

15 Tobe SW, McFarlane PA, Naimark DM. Microalbuminuria in diabetes mellitus. *Can. Med. Assoc. J.* 167(5), 499–503 (2002).

16 Richard S. Vascular effects of calcium channel antagonists: new evidence. *Drugs* 65(Suppl. 2), 1–10 (2005).

17 Hirakata H, Iino K, Ishida I *et al.* Effects of a new calcium antagonist, manidipine, on the renal hemodynamics and the vasoactive humoral factors in patients with diabetes mellitus. *Blood Press. Suppl.* 3, 124–129 (1992).

18 Martínez Martín FJ. Calcium channel-blockers for managing metabolic syndrome-associated hypertension. Trials with manidipine. *Nefrologia* 27(Suppl. 6), 26–35 (2007).

19 Hansson L, Hedner T, Dahlöf B. Prospective randomized open blinded end-point (PROBE) study. A novel design for intervention trials. Prospective Randomized Open Blinded End-Point. *Blood Press.* 1(2), 113–119 (1992).

20 McInnes GT. Antihypertensive drugs in combination: additive or greater than additive? *J. Hum. Hypertens.* 21(12), 914–916 (2007).

21 Gojanovic B, Feihl F, Liaudet L, Waeber B. Concomitant calcium entry blockade and inhibition of the renin–angiotensin system: a rational and effective means for treating hypertension. *J. Renin Angiotensin Aldosterone Syst.* 9(1), 1–9 (2008).

22 Messerli FH. Vasodilatory edema: a common side effect of antihypertensive therapy. *Curr. Cardiol. Rep.* 4(6), 479–482 (2002).

23 Tuomilehto J, Rastenyte D, Birkenhäger WH *et al.* Effects of calcium-channel blockade in older patients with diabetes and systolic hypertension. Systolic Hypertension in Europe Trial Investigators. *N. Engl. J. Med.* 340(9), 677–684 (1999).

24 Robles NR, Ocón J, Gómez CF *et al.* Lercanidipine in patients with chronic renal failure: the ZAFRA study. *Ren. Fail.* 27(1), 73–80 (2005).

25 Agodoa LY, Appel L, Bakris GL *et al*; African American Study of Kidney Disease and Hypertension (AASK) Study Group. Effect of ramipril versus amlodipine on renal outcomes in hypertensive nephrosclerosis: a randomized controlled trial. *JAMA* 285(21), 2719–2728 (2001).

26 Sakamaki Y, Sasamura H, Ikeda S *et al.* Comparison of health costs associated with treatment of hypertension with a calcium channel blocker and angiotensin-converting enzyme inhibitor in the United States and Japan. *Hypertens. Res.* 29(5), 333–338 (2006).

27 Fogari R, Mugellini A, Zoppi A *et al.* Effect of successful hypertension control by manidipine or lisinopril on albuminuria and left ventricular mass in diabetic hypertensive patients with microalbuminuria. *Eur. J. Clin. Pharmacol.* 61(7), 483–490 (2005).

28 Hayashi K, Wakino S, Sugano N *et al.* $Ca2^{+}$ channel subtypes and pharmacology in the kidney. *Circ. Res.* 100(3), 342–353 (2007).

29 Roca-Cusachs A, Schmieder RE, Triposkiadis F *et al.* MORE Study Group. Efficacy of manidipine/delapril versus losartan/hydrochlorothiazide fixed combinations in patients with hypertension and diabetes. *J. Hypertens.* 26(4), 813–818 (2008).

30 Coca A. Manidipine plus delapril in patients with Type 2 diabetes and hypertension: reducing cardiovascular risk and end-organ damage. *Expert Rev. Cardiovasc. Ther.* 5(2), 147–159 (2007).

31 Mugellini A, Preti P, Zoppi A *et al.* Effect of delapril-manidipine combination vs irbesartan-hydrochlorothiazide combination on fibrinolytic function in hypertensive patients with type II diabetes mellitus. *J. Hum. Hypertens.* 18(10), 687–691 (2004).

32 Fogari R, Derosa G, Zoppi A *et al.* Effects of manidipine/delapril versus olmesartan/hydrochlorothiazide combination therapy in elderly hypertensive patients with Type 2 diabetes mellitus. *Hypertens. Res.* 31(1), 43–50 (2008).

33 Otero ML, Claros NM; Study Investigators Group. Manidipine versus enalapril monotherapy in patients with hypertension and Type 2 diabetes mellitus: a multicenter, randomized, double-blind, 24-week study. *Clin. Ther.* 27(2), 166–173 (2005).

34 Zanchetti A, Omboni S, La Commare P, De Cesaris R, Palatini P. Efficacy, tolerability, and impact on quality of life of long-term treatment with manidipine or amlodipine in patients with essential hypertension. *J. Cardiovasc. Pharmacol.* 38(4), 642–650 (2001).

35 Payeras AC, Sladek K, Lembo G, Alberici M. Antihypertensive efficacy and safety of manidipine versus amlodipine in elderly subjects with isolated systolic hype rtension: MAISH study. *Clin. Drug Investig.* 27(9), 623–632 (2007).

36 Mulè G, Nardi E, Cottone S *et al.* Relationship of metabolic syndrome with pulse pressure in patients with essential hypertension. *Am. J. Hypertens.* 20(2), 197–203 (2007).

37 Mazza A, Zamboni S, Tikhonoff V *et al.* Pulse hypertension: a new component of the metabolic syndrome in elderly women? *J. Hum. Hypertens.* 21(12), 934–941 (2007).

38 Smith DH. Fixed-dose combination antihypertensives and reduction in target organ damage: are they all the same? *Am. J. Cardiovasc. Drugs* 7(6), 413–422 (2007).

39 Peng RD, Dominici F, Zeger SL. Reproducible epidemiologic research. *Am. J. Epidemiol.* 163(9), 783–789 (2006).

40 Bellinghieri G, Mazzaglia G, Savica V, Santoro D. Effects of manidipine and nifedipine on blood pressure and renal function in patients with chronic renal failure: a multicenter randomized controlled trial. *Ren. Fail.* 25(5), 6818–6819 (2003).

Affiliations

- Francisco Javier Martinez-Martin
  Endocrinology and Nutrition Department, Hospital General de Gran Canaria Dr. Negrin, 35020 Las Palmas de Gran Canaria, Canary Islands, Spain
  Tel.: +34 928 450 494
  Fax: +34 928 202 919
  dr.j.martinez@gmail.com
- Margarita Saiz-Satjes
  CAP Rambla Terrassa, Barcelona, Spain
  Tel.: +34 609 049 946
  margui@accesovirtual.com
