# Supplementary material for: Effects of T-Type Calcium Channel Blockers on Renal Function and Aldosterone in Patients with Hypertension: A Systematic Review and Meta-Analysis
Source: PLoS One. 2014 Oct 17;9(10):e109834. doi: 10.1371/journal.pone.0109834 (PMC4201480; doi:10.1371/journal.pone.0109834)
Supplement: File S3 — PDF files of twenty-four studies included in the meta-analysis. (ZIP) [file pone.0109834.s007.zip › Supporting information-PDF files/23. Hypertens Res 2007[30(8)]691-697.pdf]

Original Article

Comparison of the Effects of Efonidipine and Amlodipine on Aldosterone in Patients with Hypertension

Toshinari TANAKA[1)], Takayoshi TSUTAMOTO[1)], Hiroshi SAKAI[1)], Masanori FUJII[1)], Takashi YAMAMOTO[1)], and Minoru HORIE[1)]

To prevent cardiovascular disease, targeting aldosterone synthesis and release may be clinically important. Aldosterone production in the adrenal gland is mediated mainly by the T-type calcium channel *in vitro*. Efonidipine inhibits both L- and T-type Ca channels. To compare the effects of efonidipine on neurohumoral factors with those of amlodipine, an L-type Ca channel blocker, we studied 40 essential hypertensive outpatients. Forty patients who had been administered amlodipine for more than 1 year were treated with efonidipine for 6 months in place of amlodipine. Substituting efonidipine for amlodipine had no significant effect on clinic systolic blood pressure or the plasma levels of brain natriuretic peptide, norepinephrine or active renin. However, the heart rate was significantly decreased (72.0±1.3 *vs*. 69.8±1.3 beats/min, $p<0.01$) and the plasma aldosterone level was also significantly decreased after efonidipine treatment (97.7±7.9 *vs*. 79.7±5.6 pg/mL, $p<0.0001$). Changes in the aldosterone level correlated with the baseline value before the replacement of amlodipine by efonidipine ($r=-0.769$, $p<0.0001$). These findings indicate that at the effective antihypertensive doses of efonidipine and amlodipine, efonidipine significantly decreases heart rate and plasma aldosterone level compared with those under amlodipine treatment in hypertensive patients. (*Hypertens Res* 2007; 30: 691–697)

Key Words: **efonidipine, amlodipine, heart failure, aldosterone, hypertension**

Introduction

Blood pressure control is the most important component in the management of patients with hypertension. In particular, dihydropyridine calcium channel blockers are widely used for the treatment of hypertension and cardiovascular disease. However, calcium channel blockers may stimulate neurohumoral factors such as sympathetic nervous activity and the renin-angiotensin-aldosterone system (*1*, *2*). Recent clinical trials have shown that inhibition of the biological actions of aldosterone (ALD) using an ALD blocker alone or in combination with angiotensin-converting enzyme inhibitors (ACE-I) or angiotensin receptor blockers (ARB) is a useful add-on therapy in hypertensive patients and reduces the incidence of cardiovascular events among patients with chronic heart failure (CHF) (*3*, *4*).

Aldosterone has been implicated as a contributor to structural remodeling of the left ventricle (*5*–*7*), a marker of cardiovascular events, and high plasma ALD levels have been associated with an increase in cardiovascular death (*8*–*10*). ALD is synthesized in the zona glomerulosa of the adrenal gland, from which it is secreted mainly by calcium influx through the T-type calcium channel in a calcium-dependent manner in response to stimuli such as angiotensin II, adrenocorticotropic hormone, and increased extra cellular potassium

From the [1)]Department of Cardiovascular and Respiratory Medicine, Shiga University of Medical Science, Otsu, Japan.
The present study was presented at the 2006 American Heart Association Scientific Session in Chicago, Illinois, USA.
Address for Reprints: Takayoshi Tsutamoto, M.D., Ph.D., Department of Cardiovascular and Respiratory Medicine, Shiga University of Medical Science, Tsukinowa, Seta, Otsu 520–2192, Japan. E-mail: tutamoto@belle.shiga-med.ac.jp
Received October 24, 2006; Accepted in revised form March 15, 2007.

(*11*–*15*). Three types of calcium channel blocker, *i.e.*, L-, T-, and N-type calcium channel blockers, have been used as clinical drugs and medicines. Recently, the dual calcium channel blocker efonidipine, which is both a T-type and an L-type calcium channel blocker, has been shown to inhibit ALD synthesis and secretion *in vitro* (*16*). In healthy humans, efonidipine has been shown to decrease plasma ALD concentration (*17*). However, there has been no previous study comparing the effects of efonidipine, a T-type and L-type calcium channel blocker, and amlodipine, an L-type calcium channel blocker, on the plasma ALD levels in patients with hypertension.

This study compared the effects of efonidipine and amlodipine on the ALD levels and other neurohumoral factors in patients with hypertension.

Methods

Forty stable outpatients (average age, 67.4 years; range, 51–81 years) with essential hypertension who had received antihypertensive treatment with amlodipine (average dose, 6.2±0.4 mg once a day) for more than a year were enrolled in this study. Patients with renal insufficiency (serum creatinine >1.5 mg/dL), angina pectoris, or moderate to severe CHF (New York Heart Association [NYHA] functional class III or IV) were excluded from this study. To maintain the clinic systolic blood pressure, a comparable antihypertensive dose of efonidipine (36±2.3 mg once daily) was used when patients were changed from amlodipine to efonidipine. Although the use of other drugs was allowed, the doses of these agents were not changed during the study period. The general condition of each patient had been stable for more than 6 months prior to the study.

In the outpatient clinic, resting heart rate was determined from an electrocardiogram, and blood pressure measurements were independently confirmed by attending physicians. Blood sampling was performed before regimen modification (at baseline) and 6 months after amlodipine was replaced by efonidipine. Blood samples were assessed for serum potassium and serum creatinine, plasma levels of neurohumoral factors such as norepinephrine (NE), ALD and brain natriuretic peptide (BNP), and the plasma active renin concentration (PARC). The attending physicians were blinded to the neurohumoral data. Informed consent was obtained from all patients before participation in the study.

Measurement of Neurohumoral Factors

Blood samples were collected from the antecubital vein after the subject had rested in a seated position for at least 20 min. The time of blood sampling was 10 AM–12 AM. Blood was centrifuged at 3,000 rpm for 15 min at 4°C, and the plasma thus obtained was stored at −30°C until assay. The plasma NE concentration was measured by high performance liquid chromatography as soon as possible, while ALD levels were measured using commercial radioimmunoassay kits (SPAC-S aldosterone kit; TFB Company, Tokyo, Japan) within 1 month. The intra-assay and inter-assay coefficients of variation were 8.3% (n=10) and 3.2% (n=6) at 103 pg/mL, respectively. The minimal detectable quantity was 10 pg/ mL. This assay system did not cross-react with PARC, NE or BNP. Cross-reactivity with spironolactone was <0.0004% on a molar basis. Plasma concentrations of BNP were measured with a specific immunoradiometric assay using a commercial kit (Shionogi, Osaka, Japan) as previously reported (*2*, *18*).

Table 1. Baseline Characteristics of Subjects

Age (years)	67.4±1.0
Gender (male/female)	27:13
Hyperlipidemia (*n* (%))	19 (47.5)
Diabetes mellitus (*n* (%))	6 (15)
Coronary artery disease (*n* (%))	10 (25)
Chronic heart failure (*n* (%))	13 (32.5)
Etiology of chronic heart failure	
Hypertensive heart disease (*n* (%))	8 (20.0)
Previous myocardial infarction (*n* (%))	5 (12.5)
Heart rate (beats/min)	72.0±1.3
Systolic blood pressure (mmHg)	138±2.2
Diastolic blood pressure (mmHg)	78.4±2.7
Serum creatinine (mg/dL)	0.77±0.03
Brain natriuretic peptide (pg/mL)	56.7±10.5
Active renin concentration (pg/mL)	29.8±7.4
Norepinephrine (pg/mL)	648±52.5
Aldosterone (pg/mL)	97.7±7.9
Potassium (mEq/L)	4.3±0.1
Baseline therapy	
Dose of amlodipine (mg)	6.2±0.4
ACE-I (*n* (%))	8 (20.0)
ARB (*n* (%))	14 (35.0)
ACE-I or ARB (*n* (%))	21 (52.5)
β-Blockers (*n* (%))	12 (30.0)
Spironolactone (*n* (%))	8 (20.0)
Loop diuretics (*n* (%))	9 (22.5)
Digitalis (*n* (%))	5 (12.5)
Statin (*n* (%))	16 (40.0)

ACE-I, angiotensin-converting enzyme inhibitors; ARB, angiotensin receptor blockers.

Statistical Analysis

All results are expressed as the mean±SEM. Univariate analysis was performed using Student's *t*-test. Categorical data were compared against a χ^2 distribution. Differences in mean PARC were tested for statistical significance using the Kruskal-Wallis test, because the PARC was not normally distributed. Linear regression analysis was used to determine the relationship between continuous variables. A p value <0.05 was regarded as significant.

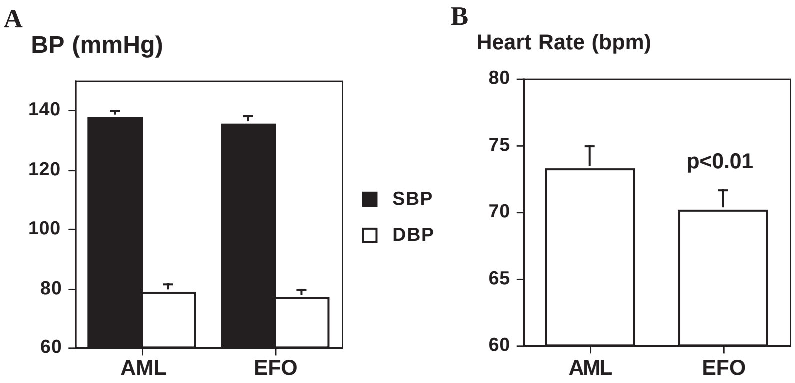

Fig. 1. *Changes in blood pressure (A) and heart rate (B) after amlodipine was replaced by efonidipine. A: Closed columns represent systolic blood pressure (SBP), and open columns diastolic blood pressure (DBP). AML, amlodipine; EFO, efonidipine.*

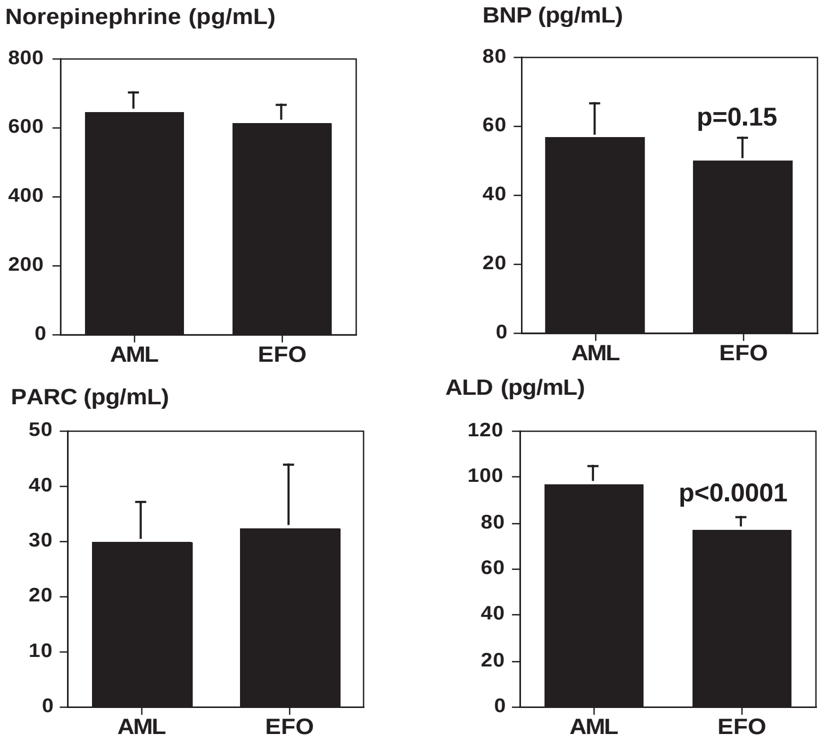

Fig. 2. *Changes in neurohumoral factors after amlodipine was replaced by efonidipine. BNP, brain natriuretic peptide; PRAC, plasma active renin concentration; ALD, aldosterone; AML, amlodipine; EFO, efonidipine.*

Results

Table 1 lists the characteristics of the subjects. The subjects consisted of 40 patients with essential hypertension, including 13 patients with CHF (NYHA class I or II) due to ischemic heart disease ($n=5$) or CHF due to hypertensive heart disease ($n=8$). Concomitant therapy other than amlodipine was maintained for at least 6 months.

There were no significant changes in blood pressure after the replacement of amlodipine by efonidipine, indicating that the two drugs had almost equivalent hypotensive effects (Fig. 1). However, the heart rate was significantly decreased (72.0±1.3 *vs*. 69.8±1.3 beats/min, $p<0.01$) after the replacement of amlodipine by efonidipine (Fig. 1).

With respect to the effects of efonidipine on neurohumoral factors, the plasma concentrations of NE and BNP and the PARC did not change after replacement (Fig. 2). After the replacement of amlodipine by efonidipine, the serum creatinine (0.77±0.03 *vs*. 0.77±0.03 mg/dL) and potassium level

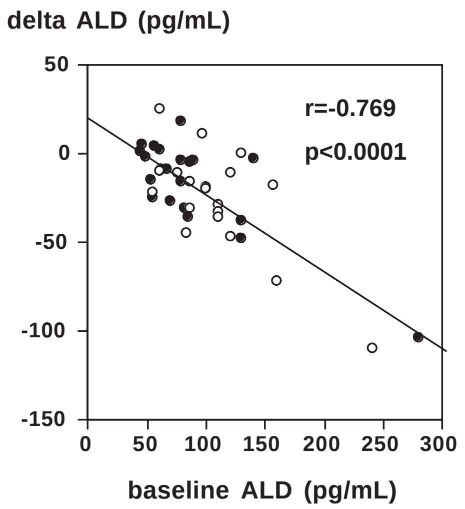

Fig. 3. *Correlation between the baseline aldosterone (ALD) level and the changes of the plasma ALD after the replacement of amlodipine by efonidipine. Open circles represent the patients who did not receive angiotensin-converting enzyme inhibitor (ACE-I) or angiotensin receptor blocker (ARB) and closed circles represent the patients who received ACE-I or ARB. ALD, aldosterone; delta ALD, [ALD level after efonidipine] – [ALD level before efonidipine].*

(4.3±0.1 *vs.* 4.4±0.1 mEq/L) did not change, but the plasma ALD level decreased significantly from 97.7±7.9 pg/mL to 79.7±5.6 pg/mL ($p<0.0001$) (Fig. 2). The changes in the ALD level correlated with the baseline value of ALD before replacement of amlodipine by efonidipine ($r=-0.769$, $p<0.0001$, Fig. 3).

Twenty-one patients received concomitant treatments with ACE-I or ARB at the baseline (Table 2). The plasma ALD level was slightly but not significantly lower in patients who also received ACE-I or ARB before the replacement of amlodipine by efonidipine. Following the replacement of amlodipine by efonidipine, plasma ALD levels decreased significantly in patients with or without ACE-I or ARB treatment (Fig. 4) and in patients with or without CHF (Fig. 5).

Discussion

Targeting ALD synthesis and release may be clinically important in preventing cardiovascular disease (*5*, *8*, *19*–*25*). Calcium ions are conveyed through the T-type calcium channel to the mitochondria, where they activate ALD synthesis, which in turn stimulates T-type calcium channel expression (*26*, *27*), creating a positive feedback loop of ALD biosynthesis in the adrenal cells. Recently, efonidipine, which is both a T-type and an L-type calcium channel blocker, has been shown to inhibit ALD synthesis and secretion *in vitro* (*16*). In healthy humans, efonidipine has been shown to suppress plasma ALD concentration (*17*). In the present study, substituting efonidipine for amlodipine in hypertensive subjects had no significant effect on clinic blood pressure, but both the heart rate and plasma ALD level were significantly decreased. These findings indicate that at the effective antihypertensive doses of efonidipine and amlodipine, efonidipine acts as not only an L-type calcium channel blocker but also a T-type calcium channel blocker in hypertensive patients.

T-type calcium channel blockers inhibit renin secretion and renin gene expression *in vivo*, while L-type calcium channel blockers act as stimulators of the renin system (*28*, *29*). A recent report suggested that amlodipine increased the plasma renin concentration in patients with essential hypertension (*30*). However, a T-type calcium channel blocker had a dual effect on plasma renin activity and renin messenger RNA expression *in vivo*, with the low concentration stimulating and the high concentration inhibiting the renin system (*31*). Since the inhibitory effect of T-type calcium channel blockers is apparent in conscious rats *in vivo* but not *in vitro*, the effect of T-type calcium channel blockers on PARC in comparison with amlodipine remains controversial (*28*, *31*). In the present study, PARC did not change significantly; therefore, the change in ALD may not have been due to the effect of renin but rather to a direct inhibition of ALD secretion.

Recently, it has been clarified that ALD can directly damage various organs, such as the heart and blood vessels, *via* mineralocorticoid receptors, independent of changes in blood pressure (*5*, *21*, *24*). In humans, it is suggested that hypertensive incidents and organ damage may occur even at plasma ALD levels within the normal range (*5*, *7*, *32*, *33*). We previously reported that treatment with atrial natriuretic peptide infusion could prevent left ventricular remodeling in patients with myocardial infarction due to the suppression of plasma ALD level (*23*, *25*). Along with its heart rate reduction, efonidipine may have additional cardiovascular protective advantages over amlodipine, based on its decrease of the plasma ALD level in CHF patients with hypertension in the present study. A previous study suggested that T-type calcium channel–mediated calcium influx was a primary component of the mechanism mediating stimulation of ALD secretion by angiotensin II (*14*). In the present study, plasma ALD levels were significantly decreased in patients with or without ACE-I or ARB treatment. Therefore, we considered that the contribution of T-type calcium channels to stimulation of ALD secretion by angiotensin II was probably limited. However, it is difficult to clarify the details of this contribution in a pilot study; further investigations will be needed.

Blood pressure control is the most important component in the management of patients with hypertension. In the present study, we did not perform 24-h ambulatory blood pressure monitoring, which is a more reliable index of blood pressure control than clinic blood pressure (*34*). Therefore, further studies are needed to compare ambulatory blood pressure data between the two treatments. In the present study, changes in the ALD level correlated with the baseline value of ALD (Fig. 3). Although it remains to be clarified whether the

Table 2. Comparisons of Baseline Characteristics between Patients Who Received ACE-I or ARB and Patients Who Did Not Received ACE-I or ARB

	ACE-I or ARB (–) (*n*=19)	ACE-I or ARB (+) (*n*=21)	*p* value
Age (years)	68.8±1.1	66.6 ±1.9	n.s.
Gender (male/female)	7:12	11:10	n.s.
Diabetes mellitus (*n* (%))	1 (2.5)	4 (10)	n.s.
Hyperlipidemia (*n* (%))	5 (12.5)	13 (32.5)	0.03
Coronary heart disease (*n* (%))	4 (10)	6 (15)	n.s.
Chronic heart failure (*n* (%))	4 (10)	9 (22.5)	n.s.
Heart rate (beats/min)	74.3±1.6	72.7±3.0	n.s.
Systolic blood pressure (mmHg)	135±3.1	139±3.1	n.s.
Diastolic blood pressure (mmHg)	79.6±3.2	77.2±4.2	n.s.
Serum creatinine (mg/dL)	0.73±0.04	0.80±0.05	n.s.
Potassium (mEq/L)	4.3±0.1	4.3±0.1	n.s.
Brain natriuretic peptide (pg/mL)	32.3±5.1	78.7±18.4	0.026
Active renin concentration (pg/mL)	21.5±9.0	37.2±11.5	n.s.
Norepinephrine (pg/mL)	736±88.2	569±57.0	n.s.
Aldosterone (pg/mL)	108.6±10.2	86.4±11.1	n.s.
Dose of amlodipine (mg/day)	6.3±0.6	6.2±0.6	n.s.
Dose of efonidipine (mg/day)	36.8±4.4	33.3±2.1	n.s.
Concomitant therapy			
Loop diuretics, (%)	3 (7.5)	6 (15)	n.s.
β-Blockers, (%)	6 (15)	6 (15)	n.s.

ACE-I, angiotensin-converting enzyme inhibitors; ARB, angiotensin receptor blockers.

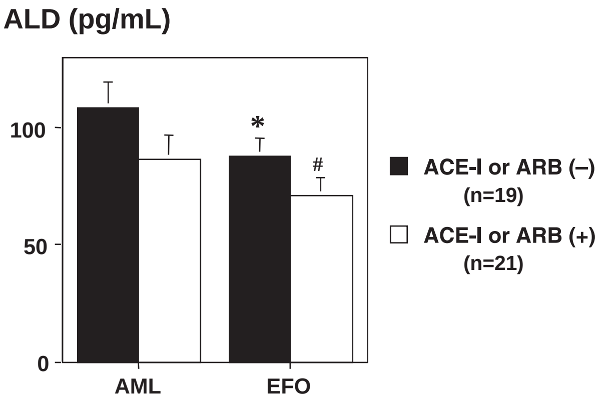

Fig. 4. *Changes in plasma aldosterone (ALD) levels after amlodipine was replaced by efonidipine. Closed columns represent patients who did not receive an angiotensin-converting enzyme inhibitor (ACE-I) or angiotensin receptor blocker (ARB) (n = 19) and open columns represent patients who received an ACE-I or ARB (n = 21). ALD, aldosterone; AML, amlodipine; EFO, efonidipine.* $^*p<0.01$, $^{\#}p<0.05$ *vs. the value during amlodipine administration.*

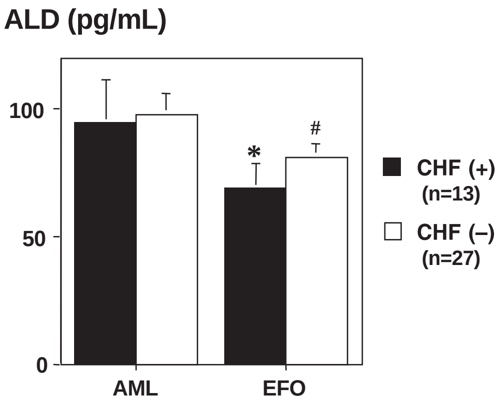

Fig. 5. *Changes in plasma aldosterone (ALD) levels after amlodipine was replaced by efonidipine. Closed columns represent the hypertensive patients with chronic heart failure (CHF) (n = 13) and open columns represent the hypertensive patients without CHF (n = 27). ALD, aldosterone; AML, amlodipine; EFO, efonidipine.* $^*p<0.01$, $^{\#}p<0.05$ *vs. the value during amlodipine administration.*

decrease in plasma ALD is associated with an improvement of surrogate markers such as endothelial function or left ventricular mass, efonidipine may have appears to have a beneficial effect in hypertensive patients, particularly those with high plasma ALD levels.

This pilot study showed that the clinical dose of efonidipine could decrease heart rate, an independent risk factor for car-

diovascular death in patients with hypertension (*35*), which is consistent with previous reports (*36*). Moreover, the plasma ALD level was decreased after the administration of efonidipine, suggesting that efonidipine may be useful for blood pressure control, especially in CHF patients with hypertension. T-type calcium channels are distributed in the sinoatrial node and conduction system, but not in cardiac muscle in the normal adult heart, and recent reports suggest that reexpression of the T-type calcium channel in the failing heart may contribute to cardiac hypertrophy (*37*), remodeling after myocardial infarction (*38*, *39*), and the development of cardiomyopathy (*40*). Taken together with our findings, these facts suggest that T-type calcium channel blockers such as efonidipine may be useful for the management of hypertension in patients with CHF. However, further studies are needed to evaluate the cardiovascular effect over the long term.

This pilot study has several limitations. First, we longitudinally evaluated differences in neurohumoral factors in the same patients before and after replacement of amlodipine by efonidipine to maintain the clinic systolic blood pressure, which may influence neurohumoral factors. However, a parallel group design would have been more appropriate; therefore, further studies are needed to confirm our findings. Finally, although we took blood samples after patients had rested for at least 20 min in a seated position and the attending physicians were blinded to the neurohumoral data, a single measurement of plasma ALD may have limited value due to the wide variation of this factor over time, and thus further studies using daily evaluation of urinary ALD will be needed.

Conclusions

At the effective antihypertensive doses of efonidipine and amlodipine, efonidipine induced significant decreases in heart rate and plasma ALD level compared with amlodipine treatment in hypertensive patients.

Acknowledgements

We wish to thank Ms. Aoi Murata for her excellent technical assistance. We also express our thanks to Mr. Daniel Mrozek for his assistance in preparing the manuscript.

References

1. Minami J, Ishimitsu T, Kawano Y, Matsuoka H: Effects of amlodipine and nifedipine retard on autonomic nerve activity in hypertensive patients. *Clin Exp Pharmacol Physiol* 1998; **25**: 572–576.
2. Tsutamoto T, Tsutsui T, Maeda K, *et al*: Effects of long-acting calcium channel antagonists on neurohumoral factors: comparison of nifedipine coat-core with amlodipine. *J Cardiovasc Pharmacol* 2003; **41** (Suppl 1): S77–S81.
3. McKelvie RS, Yusuf S, Pericak D, *et al*, The RESOLVD Pilot Study Investigators: Comparison of candesartan, enalapril, and their combination in congestive heart failure: randomized evaluation of strategies for left ventricular dysfunction (RESOLVD) pilot study. *Circulation* 1999; **100**: 1056–1064.
4. Krum H, Nolly H, Workman D, *et al*: Efficacy of eplerenone added to renin-angiotensin blockade in hypertensive patients. *Hypertension* 2002; **40**: 117–123.
5. Tsutamoto T, Wada A, Maeda K, *et al*: Effect of spironolactone on plasma brain natriuretic peptide and left ventricular remodeling in patients with congestive heart failure. *J Am Coll Cardiol* 2001; **37**: 1228–1233.
6. Weber KT: Cardioreparation in hypertensive heart disease. *Hypertension* 2001; **38**: 588–591.
7. Vasan RS, Evans JC, Benjamin EJ, *et al*: Relations of serum aldosterone to cardiac structure: gender-related differences in the Framingham Heart Study. *Hypertension* 2004; **43**: 957–962.
8. Brunner HR, Laragh JH, Baer L, *et al*: Essential hypertension: renin and aldosterone, heart attack and stroke. *N Engl J Med* 1972; **286**: 441–449.
9. Swedberg K, Eneroth P, Kjekshus J, Snapinn S, CONSENSUS Trial Study Group: Effects of enalapril and neuroendocrine activation on prognosis in severe congestive heart failure (follow-up of the CONSENSUS trial). *Am J Cardiol* 1990; **66**: 40D–45D.
10. Cohn JN, Anand IS, Latini R, Masson S, Chiang YT, Glazer R: Sustained reduction of aldosterone in response to the angiotensin receptor blocker valsartan in patients with chronic heart failure: results from the Valsartan Heart Failure Trial. *Circulation* 2003; **108**: 1306–1309.
11. Barrett PQ, Isales CM, Bollag WB, McCarthy RT: Ca^{2+} channels and aldosterone secretion: modulation by K^+ and atrial natriuretic peptide. *Am J Physiol* 1991; **261**: F706–F719.
12. Rossier MF, Burnay MM, Vallotton MB, Capponi AM: Distinct functions of T- and L-type calcium channels during activation of bovine adrenal glomerulosa cells. *Endocrinology* 1996; **137**: 4817–4826.
13. Rossier MF, Ertel EA, Vallotton MB, Capponi AM: Inhibitory action of mibefradil on calcium signaling and aldosterone synthesis in bovine adrenal glomerulosa cells. *J Pharmacol Exp Ther* 1998; **287**: 824–831.
14. Chen XL, Bayliss DA, Fern RJ, Barrett PQ: A role for T-type Ca^{2+} channels in the synergistic control of aldosterone production by ANG II and K^+. *Am J Physiol* 1999; **276**: F674–F683.
15. Lesouhaitier O, Chiappe A, Rossier MF: Aldosterone increases T-type calcium currents in human adrenocarcinoma (H295R) cells by inducing channel expression. *Endocrinology* 2001; **142**: 4320–4330.
16. Imagawa K, Okayama S, Takaoka M, *et al*: Inhibitory effect of efonidipine on aldosterone synthesis and secretion in human adrenocarcinoma (H295R) cells. *J Cardiovasc Pharmacol* 2006; **47**: 133–138.
17. Okayama S, Imagawa K, Naya N, *et al*: Blocking T-type Ca^{2+} channels with efonidipine decreased plasma aldosterone concentration in healthy volunteers. *Hypertens Res* 2006; **29**: 493–497.
18. Tsutamoto T, Wada A, Maeda K, *et al*: Attenuation of compensation of endogenous cardiac natriuretic peptide system in chronic heart failure: prognostic role of plasma brain

natriuretic peptide concentration in patients with chronic symptomatic left ventricular dysfunction. *Circulation* 1997; **96**: 509–516.
19. The CONSENSUS Trial Study Group: Effects of enalapril on mortality in severe congestive heart failure. Results of the Cooperative North Scandinavian Enalapril Survival Study (CONSENSUS). *N Engl J Med* 1987; **316**: 1429–1435.
20. Cohn JN, Johnson G, Ziesche S, *et al*: A comparison of enalapril with hydralazine-isosorbide dinitrate in the treatment of chronic congestive heart failure. *N Engl J Med* 1991; **325**: 303–310.
21. Pitt B, Zannad F, Remme WJ, *et al*, Randomized Aldactone Evaluation Study Investigators: The effect of spironolactone on morbidity and mortality in patients with severe heart failure. *N Engl J Med* 1999; **341**: 709–717.
22. Hayashi M, Tsutamoto T, Wada A, *et al*: Intravenous atrial natriuretic peptide prevents left ventricular remodeling in patients with first anterior acute myocardial infarction. *J Am Coll Cardiol* 2001; **37**: 1820–1826.
23. Hayashi M, Tsutamoto T, Wada A, *et al*: Relationship between transcardiac extraction of aldosterone and left ventricular remodeling in patients with first acute myocardial infarction: extracting aldosterone through the heart promotes ventricular remodeling after acute myocardial infarction. *J Am Coll Cardiol* 2001; **38**: 1375–1382.
24. Pitt B, Remme W, Zannad F, *et al*: Eplerenone, a selective aldosterone blocker, in patients with left ventricular dysfunction after myocardial infarction. *N Engl J Med* 2003; **348**: 1309–1321.
25. Hayashi M, Tsutamoto T, Wada A, *et al*: Immediate administration of mineralocorticoid receptor antagonist spironolactone prevents post-infarct left ventricular remodeling associated with suppression of a marker of myocardial collagen synthesis in patients with first anterior acute myocardial infarction. *Circulation* 2003; **107**: 2559–2565.
26. Rossier MF, Lesouhaitier O, Perrier E, Bockhorn L, Chiappe A, Lalevee N: Aldosterone regulation of T-type calcium channels. *J Steroid Biochem Mol Biol* 2003; **85**: 383–388.
27. Lalevee N, Rebsamen MC, Barrere-Lemaire S, *et al*: Aldosterone increases T-type calcium channel expression and *in vitro* beating frequency in neonatal rat cardiomyocytes. *Cardiovasc Res* 2005; **67**: 216–224.
28. Wagner C, Kramer BK, Hinder M, Kieninger M, Kurtz A: T-type and L-type calcium channel blockers exert opposite effects on renin secretion and renin gene expression in conscious rats. *Br J Pharmacol* 1998; **124**: 579–585.
29. Karam H, Clozel JP, Bruneval P, Gonzalez MF, Menard J: Contrasting effects of selective T- and L-type calcium channel blockade on glomerular damage in DOCA hypertensive rats. *Hypertension* 1999; **34**: 673–678.
30. Lamarre-Cliche M, de Champlain J, Lacourciere Y, Poirier L, Karas M, Larochelle P: Effects of circadian rhythms, posture, and medication on renin-aldosterone interrelations in essential hypertensives. *Am J Hypertens* 2005; **18**: 56–64.
31. Schweda F, Kurtz A: Cellular mechanism of renin release. *Acta Physiol Scand* 2004; **181**: 383–390.
32. Schunkert H, Hense HW, Muscholl M, *et al*: Associations between circulating components of the renin-angiotensin-aldosterone system and left ventricular mass. *Heart* 1997; **77**: 24–31.
33. Vasan RS, Evans JC, Larson MG, *et al*: Serum aldosterone and the incidence of hypertension in nonhypertensive persons. *N Engl J Med* 2004; **351**: 33–41.
34. Kario K, Yasui N, Yokoi H: Ambulatory blood pressure monitoring for cardiovascular medicine. *IEEE Eng Med Biol Mag* 2003; **22**: 81–88.
35. Gillman MW, Kannel WB, Belanger A, D'Agostino RB: Influence of heart rate on mortality among persons with hypertension: the Framingham Study. *Am Heart J* 1993; **125**: 1148–1154.
36. Harada K, Nomura M, Nishikado A, Uehara K, Nakaya Y, Ito S: Clinical efficacy of efonidipine hydrochloride, a T-type calcium channel inhibitor, on sympathetic activities. *Circ J* 2003; **67**: 139–145.
37. Martinez ML, Heredia MP, Delgado C: Expression of T-type Ca^{2+} channels in ventricular cells from hypertrophied rat hearts. *J Mol Cell Cardiol* 1999; **31**: 1617–1625.
38. Mulder P, Richard V, Compagnon P, *et al*: Increased survival after long-term treatment with mibefradil, a selective T-channel calcium antagonist, in heart failure. *J Am Coll Cardiol* 1997; **29**: 416–421.
39. Huang B, Qin D, Deng L, Boutjdir M, El-Sherif N: Reexpression of T-type Ca^{2+} channel gene and current in post-infarction remodeled rat left ventricle. *Cardiovasc Res* 2000; **46**: 442–449.
40. Sen L, Smith TW: T-type Ca^{2+} channels are abnormal in genetically determined cardiomyopathic hamster hearts. *Circ Res* 1994; **75**: 149–155.
