# Supplementary material for: Effects of T-Type Calcium Channel Blockers on Renal Function and Aldosterone in Patients with Hypertension: A Systematic Review and Meta-Analysis
Source: PLoS One. 2014 Oct 17;9(10):e109834. doi: 10.1371/journal.pone.0109834 (PMC4201480; doi:10.1371/journal.pone.0109834)
Supplement: File S3 — PDF files of twenty-four studies included in the meta-analysis. (ZIP) [file pone.0109834.s007.zip › Supporting information-PDF files/24. Am J Med Sci 2007[333(6)]321-326.pdf]

Azelnidipine Reduces Urinary Protein Excretion and Urinary Liver-Type Fatty Acid Binding Protein in Patients with Hypertensive Chronic Kidney Disease

TSUKASA NAKAMURA, MD; TAKESHI SUGAYA, MD, PhD;* YASUHIRO KAWAGOE, MD; TSUKASA SUZUKI, MD; YOSHIHIKO UEDA, MD;** HIKARU KOIDE, MD;*** TERUO INOUE, MD;**** KOICHI NODE, MD****

ABSTRACT: *Background:* Hypoxia plays a significant role in the pathogenesis and progression of chronic renal disease. Urinary liver-type fatty acid binding protein (L-FABP) levels reflect the clinical prognosis of chronic renal disease. The calcium channel blocker azelnidipine has anti-oxidative properties and these may contribute to the beneficial effects of this drug. The aim of the present study was to determine whether azelnidipine and/or amlodipine affected urinary protein excretion or the urinary levels of 8-OHdG and L-FABP in hypertensive patients with mild chronic kidney disease (CKD). *Methods:* Thirty moderately hypertensive chronic kidney disease patients were randomly assigned to 2 treatment groups: azelnidipine 16 mg once daily or amlodipine 5 mg once daily. Treatment was continued for 6 months. Urinary protein excretion and urinary levels of 8-OHdG and urinary L-FABP were measured before 3 and 6 months after the treatment period. *Results:* Both drugs exhibited comparable and significant effects on the systolic and diastolic blood pressure. Azelnidipine decreased heart rate significantly after 3 and 6 months whereas amlodipine increased it significantly after 3 and 6 months. Urinary protein excretion, urinary 8-OHdG and urinary L-FABP levels decreased significantly after 3 months ($p < 0.05$) and 6 months ($p < 0.05$) in the azelnidipine group. In contrast, amlodipine showed little effect on urinary protein excretion or the urinary levels of 8-OHdG and L-FABP throughout the experimental period. *Conclusions:* Azelnidipine is renoprotective in hypertensive patients with mild CKD and this action is, at least in part, due to the anti-oxidative effect. **Key Words:** Ca channel blocker, chronic kidney disease, hypertension, oxidant stress. **[Am J Med Sci 2007;333(6):321–326.]**

Derangement of the peritubular capillary circulation with consequent tubulointerstitial hypoxia plays a pivotal role in the pathogenesis of renal injury.[1] Chronic kidney disease (CKD) including IgA nephropathy runs a highly variable clinical course and commonly exhibits tubulointerstitial damage.[2] The presence and severity of tubulointerstitial injury is often associated with rapid progression to end-stage renal failure. Various pathophysiological stresses on the proximal tubules such as significant proteinuria induces upregulation of human liver-type fatty acid binding protein (L-FABP) gene expression resulting in increased proximal tubular excretion of L-FABP and increased urinary L-FABP excretion.[3] Various disease-associated stresses such as severe proteinuria or ischemia result in an overload of free fatty acids in the proximal tubules and exacerbate tubulointerstitial damage.[3] We have previously reported that urinary L-FABP levels were increased in patients with IgA nephropathy, diabetic nephropathy, and polycystic kidney disease.[4–6] Urinary L-FABP, therefore, represents a potential clinical biomarker that may be of use in monitoring and predicting the progression of chronic renal disease.[3]

Urinary 8-OHdG has been reported to serve as a sensitive biomarker of oxidative DNA damage and also of oxidative stress.[7] Increased urinary 8-OHdG and the risk of vascular complication may be present at early stages of diabetes.[8]

*Department of Internal Medicine, Shinmatsudo Central General Hospital, Chiba, *Research Unit for Organ Regeneration, Riken Kobe Institute, Hyogo, **Department of Pathology, Koshigaya Hospital, Dokkyo University School of Medicine, Saitama, ***Department of Internal Medicine, Koto Hospital, Tokyo, ****Department of Cardiovascular & Renal Medicine, Saga University Faculty of Medicine, Saga, Japan.*

Submitted August 1, 2006; accepted in revised form November 17, 2006.

Correspondence: Koichi Node, MD, Department of Cardiovascular & Renal Medicine, Saga University Faculty of Medicine, 5-1-1, Nabeshima, Saga 849-8501, Japan (E-mail: node@med.saga-u.ac.jp).

Copyright © by the Southern Society for Clinical Investigation. Unauthorized reproduction of this article is prohibited.

Table 1. Clinical and Laboratory Findings of the Subject

	Azelnidipine	Amlodipine
N	15	15
Sex (male/female)	8/7	7/8
Age (years)	48 ± 16	46 ± 14
Dose (mg/day)	16	5
SBP (mm Hg)	158 ± 15	154 ± 13
DBP (mm Hg)	98 ± 7	94 ± 5
Heart rate (beats/min)	74 ± 6	76 ± 4
Serum creatinine (mg/dL)	1.0 ± 0.2	1.1 ± 0.2
24h Ccr (ml/min)	98 ± 8	102 ± 10
Other drugs		
Antiplatelet	n = 10	n = 9
ARB	n = 4	n = 5
ACEI	n = 4	n = 4
Statin	n = 4	n = 4
Primary disease		
IgA nephropathy	n = 8	n = 9
Non-IgA proliferative GN	n = 4	n = 3
Membranous nephropathy	n = 1	n = 2
Membranoproliferative GN	n = 2	n = 1

Data expressed as mean ± SD.
SBP = systolic blood pressure, DBP = diastolic blood pressure, GFR = glomerular filtration rate, ARB = angiotensin II receptor blockade, ACEI = angiotensin converting enzyme inhibitor, GN = glomerulonephritis, Ccr = creatinine clearance.

Recently some calcium channel blockers have been demonstrated to exhibit an organ protection effect that is independent of their antihypertensive action. Azelnidipine is a novel dihydropyridine, L-type calcium channel blocker with a long-acting antihypertensive action and a low reported incidence of tachycardia.[9] Azelnidipine attenuates angiotensin II-induced peritubular ischemia. In addition, recent experimental evidence suggests that azelnidipine has anti-oxidative properties and it may be the case that these actions may be involved in its beneficial effects on renal injury.[1] However, there is little data available regarding the effect of azelnidipine and/or amlodipine on renal function in patients with hypertensive chronic kidney disease.

We therefore conducted a study to evaluate and compare treatment with azelnidipine or amlodipine with a focus on the effect upon urinary L-FABP levels (tubular function) and/or urinary protein excretion (glomerular function) in hypertensive patients with CKD.

Methods

We enrolled 30 nondiabetic hypertensive patients with CKD (16 males and 14 females, age; 47 ± 15 years, systolic blood pressure; 156 ± 14 mm Hg, diastolic blood pressure; 96 ± 6 mm Hg) on the basis of a serum creatinine concentration less than 1.5 mg/dL in order to assess the effects of azelnidipine and amlodipine on mild CKD. Office blood pressures were manually measured, using a mercury sphygmomanometer with an appropriately sized cuff. In each patient the diagnosis of CKD had been made on the basis of renal biopsy. Table 1 summarizes the clinical and laboratory findings of the subjects. Informed consent was obtained from each patient and the local ethical committee approved the study. The doses of other antihypertensive agents and statins were held constant during the study period. Patients were randomly assigned into 2 groups and were treated with either azelnidipine (16 mg once daily) or amlodipine (5 mg once daily). Treatment was continued for 6 months and clinical parameters were monitored every month for 6 months, with no change in medication during the experimental period. Urinary L-FABP and urinary 8-OHdG were measured at baseline before treatment and at 3 and 6 months. First, we compared the baseline values in CKD patients with the values in Urinary L-FABP and 8-OHdG and healthy control subjects (15 males and 15 females, age; 47 ± 15 years), We then evaluated the serial changes in the values after initiation of the various treatments. Urinary protein excretion, serum creatinine, urinary creatinine, and 24h creatinine clearance were also measured. Urinary L-FABP levels[3–6] and urinary 8-OHdG levels[10] were measured by a specific ELISA as previously described. We used morning urine samples for measuring urinary 8-OHdG and urinary L-FABP. Miwa et al.[11] have shown that urinary 8-OHdG levels in morning spot urine significantly correlated with those in 24h pooled urine and that a morning spot urine sample can be used for the measurement of 8-OHdG instead of inconvenient 24h sample. In addition, we recognized that urinary L-FABP of 24h pooled urine correlated ($r = 0.92$, $p < 0.0001$) with that of spot urine (Sugaya T, et al. unpublished data). We have previously reported the reproducibility of urinary L-FABP levels.[3] Data are shown as mean ± SD. To compare the parameters from the 2 groups, we used the Mann-Whitney U test for unpaired data and the Wilcoxon rank-sum test for paired data. A P value of less than 0.05 was considered statistically significant.

Results

Both drugs showed similar effects upon systolic and diastolic blood pressure (systolic blood pressure: azelnidipine before; 158 ± 15 mm Hg, 3 months; 138 ± 12 mm Hg, $p < 0.05$, 6 months; 132 ± 12 mm Hg, $p < 0.05$, amlodipine before; 154 ± 13 mm Hg, 3 months: 136 ± 10 mm Hg, $p < 0.05$, 6 months, 130 ± 8 mm Hg, $p < 0.05$) (Figure 1) Both at months 3 and 6, blood pressure showed no differences between azelnidipine group and amlodipine group (3 months: $p = 0.334$, 6 months: $p = 0.413$, statistically not significant). The heart rate decreased from 74 ± 6 to 71 ± 5 beats/min after 3 months and to 71 ± 4 beats/min after 6 months in the azelnidipine groups ($p < 0.05$), whereas it significantly increased from 76 ± 4 to 79 ± 6 beats/min after 3 months and to 79 ± 5 beats/min after 6 months in the amlodipine group ($p < 0.05$) (Figure 2). Urinary protein levels decreased from 1.5 ± 0.4 to 0.9 ± 0.3 g/day after 3 months ($p < 0.05$) and to 0.8 ± 0.3 g/day ($p < 0.05$) after 6 months in the azelnidipine group.

Baseline urinary 8-OHdG levels in all CKD patients (26.5 ± 8.0 ng/mg creatinine) were significantly higher than those in healthy controls (7.5 ± 1.5 ng/mg creatinine) ($p < 0.001$). Urinary 8-OHdG levels decreased from 26.5 ± 8.0 to 20.5 ± 6.0 ng/mg creatinine ($p < 0.05$) after 3 months and to 14.0 ± 4.0 ng/mg creatinine ($p < 0.05$) after 6 months.

Baseline urinary L-FABP levels in all CKD patients (110.5 ± 40.5 μg/g creatinine) were significantly higher than those in healthy controls (5.5 ± 4.0 μg/g creatinine) ($p < 0.001$). Urinary

Copyright © by the Southern Society for Clinical Investigation. Unauthorized reproduction of this article is prohibited.

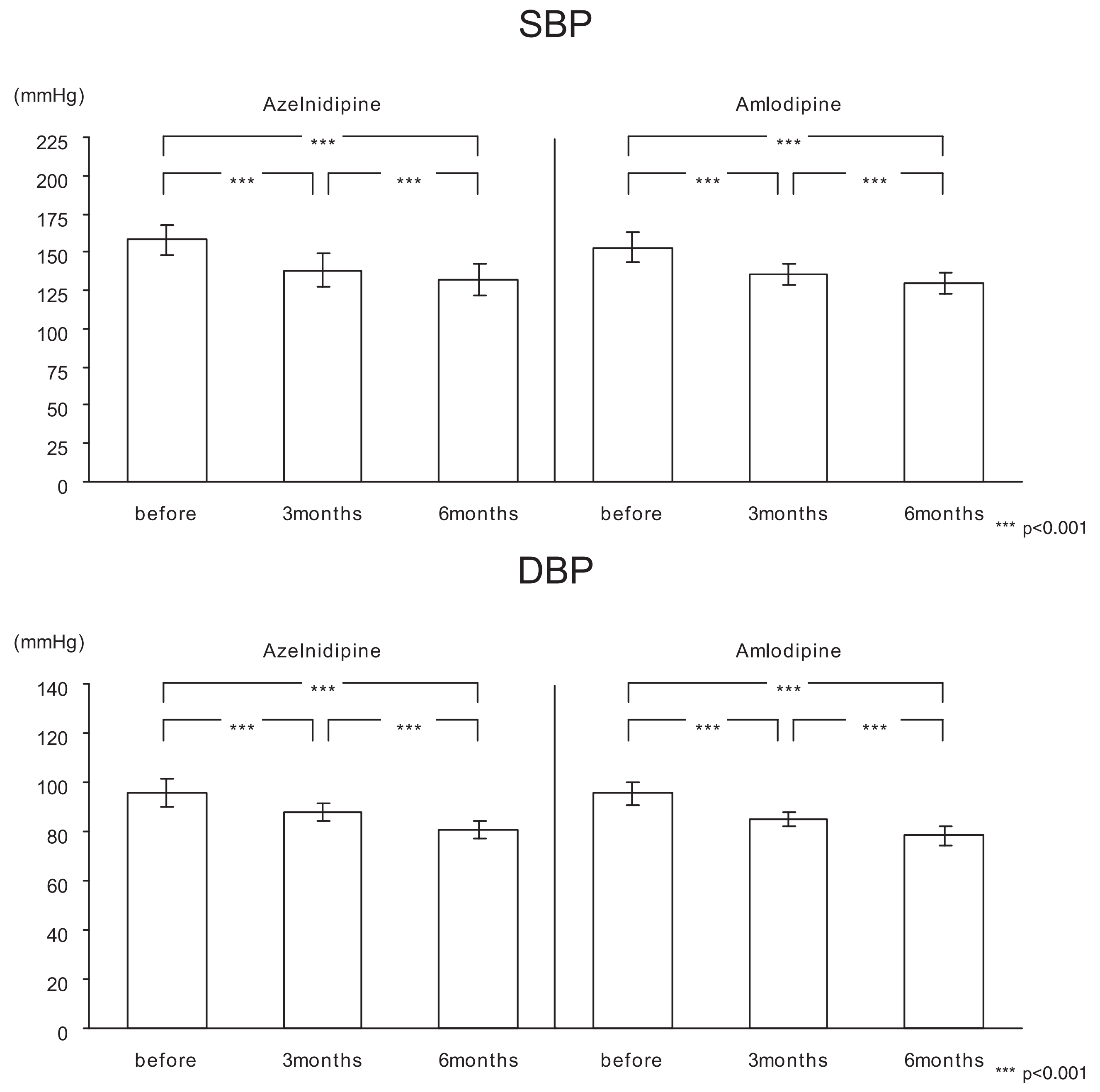

Figure 1. Effects of azelnidipine and amlodipine on blood pressure. Both drugs exhibited similar effects on systolic and diastolic blood pressure.

L-FABP levels decreased from 110.5 ± 40.5 to 90.0 ± 30.5 μg/g creatinine ($p < 0.05$) after 3 months and to 75.0 ± 25.5 μg/g creatinine ($p < 0.05$) after 6 months in the azelnidipine group. In addition, baseline urinary 8-OHdG levels were significantly correlated with baseline urinary L-FABP levels ($r = 0.49$, $p < 0.01$). The magnitude of baseline proteinuria was significantly correlated with baseline urinary L-FABP levels ($r = 0.68$, $p < 0.001$) and urinary 8-OHdG levels ($r = 0.65$, $p < 0.0001$). The changes (reduction rates) in proteinuria were significantly correlated with the changes in urinary L-FABP levels ($r = 0.61$, $p < 0.001$) and those in urinary 8-OHdG levels ($r = 0.78$, $p < 0.0001$). In contrast, amlodipine showed little effects on urinary protein excretion or the urinary levels of 8-OHdG and L-FABP throughout the experimental period (Table 2). Blood urea nitrogen, serum creatinine and 24h creatinine clearance were comparable between the azelnidipine and amlodipine groups throughout the experimental period.

Copyright © by the Southern Society for Clinical Investigation. Unauthorized reproduction of this article is prohibited.

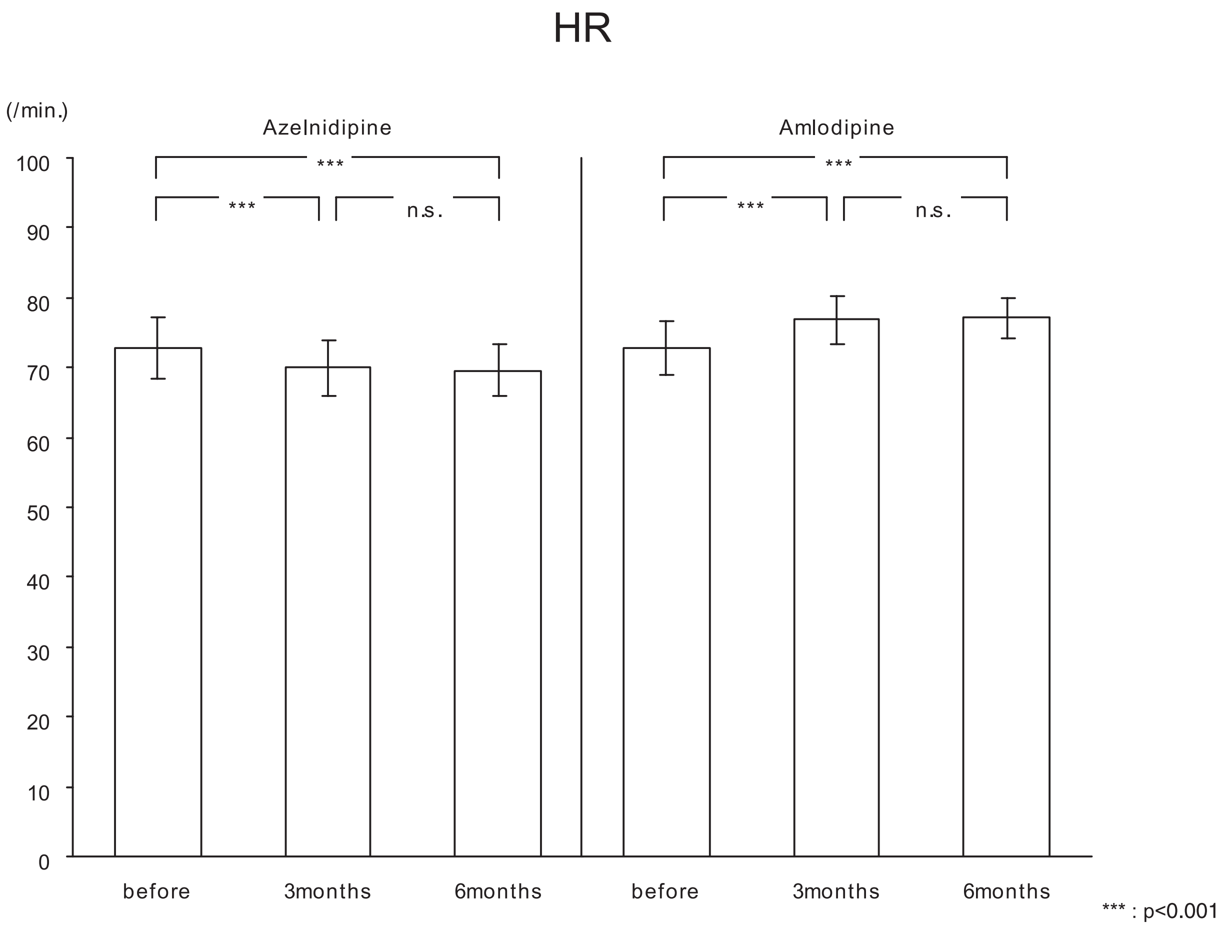

Figure 2. Effects of azelnidipine and amlodipine on heart rate. Azelnidipine decreased heart rate significantly after 3 and 6 months whereas amlodipine increased it.

Discussion

In the present study, we demonstrated that the baseline proteiuria was correlated with the baseline urinary L-FABP levels, suggesting a linkage between glomerular and tubulointerstitial injury in CKD. In addition, we reported that azelnidipine, an L-type calcium channel blocker, ameliorated markers of glomerular and tubulointerstitial injury in hypertensive patients with CKD. Tanaka et al[12] previously reported that azelnidipine protects tubular cells from hypoxic injury *in vitro* and ameliorated ischemic/reperfusion injury in an *in vivo* experimental model. Tubulointerstitial ischemia (hypoxia) is caused by loss of peritubular capillary flow. Matsumoto et al[13] have reported that blood flow in peritubular capillaries was decreased by approximately 40% in the model of tubulointerstitial injury (irreversible glomerulonephritis model induced by uninephrectomy and repeated anti-Thy-1 antibody injections) compared with the controls. Therefore, maintenance of the blood flow in the peritubular capillary network may protect from loss of renal function. Fujimoto[14] reported that azelnidipine may increase peritubular capillary blood flow and reduce renal hypoxia and tubulointerstitial injury in a rat model. However, little is known about the effect of azelnidipine on tubulointerstitial injury in human

Table 2. Changes of Urinary Protein Excretion (g/day), Urinary 8-OHdG (ng/mg crea tinine), Urinary L-FABP (Ī-g/g crea tinine), Serum Creatinine (mg/dl) and 24h Ccr (ml/min) Before and 3 and 6 Months After Treatment

	Before	3 Months	6 Months
Urinary protein			
Azelnidipine	1.5 ± 0.4	0.9 ± 0.3*	0.8 ± 0.3*
Amlodipine	1.6 ± 0.3	1.7 ± 0.4	1.7 ± 0.5
Urinary 8-OHdG			
Azelnidipine	26.5 ± 8.0	20.5 ± 6.0*	14.0 ± 4.0*
Amlodipine	25.0 ± 7.5	26.0 ± 8.0	26.2 ± 8.3
Urinary L-FABP			
Azelnidipine	110.5 ± 40.5	90.0 ± 30.5*	75.0 ± 25.5*
Amlodipine	115.0 ± 38.0	120.0 ± 30.0	122.5 ± 35.0
Serum creatinine			
Azelnidipine	1.0 ± 0.2	1.0 ± 0.1	1.0 ± 0.2
Amlodipine	1.1 ± 0.2	1.0 ± 0.1	1.1 ± 0.1
24h Ccr			
Azelnidipine	98 ± 8	100 ± 8	98 ± 10
Amlodipine	102 ± 10	101 ± 12	98 ± 10

*Before versus after: *p < 0.05.*

Copyright © by the Southern Society for Clinical Investigation. Unauthorized reproduction of this article is prohibited.

patients with CKD. Recently, Kamijo et al[3] reported that various stresses such as massive proteinuria and ischemia induce free fatty acid overload in the proximal tubules and exacerbate tubulointerstitial damage. They also reported[15] that urinary L-FABP levels were more sensitive than proteinuria in predicting the progression of CKD thereby indicating that urinary L-FABP is a useful clinical biomarker in the monitoring of CKD. Recently, Kamijo et al[16] reported that the estimated contribution of serum L-FABP to urinary L-FABP in CKD was only $3 \pm 3\%$, suggesting that serum L-FABP levels do not influence urinary L-FABP levels.

Urinary 8-OHdG has been reported as a sensitive biomarker of oxidative DNA damage, and the excretion of urinary 8-OHdG is significantly correlated with the severity of tubulointerstitial lesions.[17] In the present study, we showed that urinary 8-OHdG levels in patients with CKD were significantly higher than those in healthy subjects and that these levels significantly correlated with urinary L-FABP levels. These data suggest that increased urinary L-FABP levels may occur, in part, as a response to oxidative stress induced by hypoxia. Azelnidipine but not amlodipine has a potent anti-oxidant effect that may provide additive and significant clinical benefit when combined with its long-lasting antihypertensive action and low incidence of tachycardia.[9] Recently, Nakama et al[18] reported that the beneficial effects of azelnidipine are not solely due to its calcium channel blocking property, but result from its anti-oxidative ability. Therefore, our data suggest that azelnidipine may reduce urinary L-FABP levels, at least in part, due to ameliorating oxidative stress within the tubulointerstitium.

Azelnidipine decreased heart rate significantly and this heart rate reduction may be associated with renoprotection. In a preliminary study, we noted that the administration of a β-blocker reduced heart rate in hypertensive patients with CKD but did not affect urinary protein excretion or the urinary levels of 8-OHdG or L-FABP (data not shown). It is apparent that azelnidipine mediated renoprotection is not due solely to heart rate reduction. In the present study, angiotensin II receptor antagonists (ARBs) and statins had been administered before azelnidipine or amlodipine treatment. ARBs have been shown to prevent vascular changes, ameliorate tubular hypoxia and reduce oxidative stress.[19,20] We and other investigators have reported that statins ameliorate tubular injury and podocyte injury and that they preserve GFR and reduce proteinuria in renal disease.[21–23] Therefore, the use of ARB and/or statins may have influenced the urinary protein excretion and/or urinary L-FABP concentrations in the patients with CKD enrolled in this study. However, the patient profile was comparable at the initiation of the study thereby excluding the previous use of ARB and/or statins as a confounding factor. Among the patients treated with ACEI and/or ARB, there showed no differences in the effects of additional azelnidipine administration on proteinuria and the urinary biomarkers compared to those without ACEI/ARB treatment. Thus, the reduction in the levels of urinary protein, urinary 8-OHdG and urinary L-FABP might be mainly due to the administration of azelnidipine. We therefore believe that azelnidipine therapy may be beneficial beyond the conventional therapy of ACEI/ARB and statin, which are commonly used in the CKD populations.

A limitation of our study is that we measured only 24h creatinine clearance but not GFR. Because amlodipine is known to increase GFR acutely, there is a possibility that excretion of urinary biomarkers resulted, in part, from changes in GFR by hemodynamic alterations. Thus, simultaneous GFR measurement would be helpful to assess more precisely whether the amelioration of urinary biomarker excretion by azelnidipie was independent of changes in GFR.

In summary, we reported that azelnidipine but not amlodipine may ameliorate tubulointerstitial injury in hypertensive patients with CKD.

References

1. **Kondo N, Kiyomoto H, Yamamoto T, et al.** Effects of calcium channel blockade on angiotensin II-induced peritubular ischemia in rats. J Pharmacol Exp Ther 2006;316:1047–52.
2. **Lai KN, Chan LY, Leung JC.** Mechanisms of tubulointerstitial injury in IgA nephropathy. Kidney Int Suppl 2005;94:S110–5.
3. **Kamijo A, Kimura K, Sugaya T, et al.** Urinary fatty acid-binding protein as a new clinical marker of the progression of chronic renal disease. J Lab Clin Med 2004;143:23–30.
4. **Nakamura T, Sugaya T, Kawagoe Y, et al.** Candesartan reduces urinary fatty acid-binding protein excretion in patients with autosomal dominant polycystic kidney disease. Am J Med Sci 2005;330:161–5.
5. **Nakamura T, Sugaya T, Ebihara I, et al.** Urinary liver-type fatty acid binding protein: discrimination between IgA nephropathy and thin basement membrane nephropathy. Am J Nephrol 2005;25:447–50.
6. **Nakamura T, Sugaya T, Kawagoe Y, et al.** Effect of pitavastatin on urinary liver-type fatty acid-binding protein levels in patients with early diabetic nephropathy. Diabetes Care 2005;28:2728–32.
7. **Xu GW, Yao OH, Weng OF, et al.** Study of urinary 8-hydroxydeoxyguanosine as a biomarker of oxidative DNA damage in diabetic nephropathy. J Pharm Biomed Anal 2004;36:101–4.
8. **Hata I, Kaji M, Hirano S, et al.** Urinary oxidative stress markers in young patients with type 1 diabetes. Pediatr Int 2006;48:58–61.
9. **Shinomiya K, Mizushige K, Fukunaga M, et al.** Antioxidant effect of a new calcium antagonist, azelnidipine, in cultured human arterial endothelial cells. J Int Med Res 2004;32:170–5.
10. **Saito S, Yamauchi H, Hasui Y, et al.** Quantitive determination of urinary 8-hydroxy deoxyguanosine (8-OHdG) by using ELISA. Res Commun Mol Pathol Pharmacol 2000;107:39–44.

Copyright © by the Southern Society for Clinical Investigation. Unauthorized reproduction of this article is prohibited.

11. **Miwa M, Matsumaru H, Akimoto Y, et al.** Quantitative determination of urinary 8-hydroxy-2′-deoxyguanosine level in healthy Japanese volunteers. Biofactors 2004;22:249–53.
12. **Tanaka T, Nangaku M, Miyata T, et al.** Blockade of calcium influx through L-type calcium channels attenuates mitochondrial injury and apoptosis in hypoxic renal tubular cells. J Am Soc Nephrol 2004;15:2320–33.
13. **Matsumoto M, Tanaka T, Yamamoto T, et al.** Hypoperfusion of peritubular capillaries induces chronic hypoxia before progression of tubulointerstitial injury in a progressive model of rat glomerulonephritis. J Am Soc Nephrol 2004;15: 1574–81.
14. **Fujimoto S.** Renoprotective effects of azelnidipine by improvement of renal microcirculation. Kawasaki Igakkaishi 2004;30:111–22.
15. **Kamijo A, Sugaya T, Hikawa A, et al.** Clinical evaluation of urinary excretion of liver-type fatty acid-binding protein as a marker for the monitoring of chronic kidney disease: a multicenter trial. J Lab Clin Med 2005;145: 125–33.
16. **Kamijo A, Sugaya T, Hikawa A, et al.** Urinary liver-type fatty acid binding protein as a useful biomarker in chronic kidney disease. Mol Cell Biochem, in press.
17. **Kanauchi M, Nishioka H, Hashimoto T.** Oxidative DNA damage and tubulointerstitial injury in diabetic nephropathy. Nephron 2002;91:327–9.
18. **Nakamura K, Yamagishi S, Inoue H.** Unique atheroprotective property of azelnidipine, a dihydropyridine-based calcium antagonist. Med Hypothesis 2005;65:155–7.
19. **Manotham K, Tanaka T, Matsumoto M, et al.** Evidence of tubular hypoxia in the early phase in the remnant kidney model. J Am Soc Nephrol 2004;15:1277–88.
20. **Fliser D, Wagner KK, Loos A, et al.** Chronic angiotensin II receptor blockade reduces intrarenal vascular resistance in patients with type 2 diabetes. J Am Soc Nephrol 2005;16: 1135–40.
21. **Shibata S, Nagase M, Fujita T.** Fluvastatin ameliorates podocyte injury in proteinuria rats *via* modulation of excessive Rho signaling. J Am Soc Nephrol 2006;17:754–64.
22. **Agarwal R.** Effects of statins on renal function. Am J Cardiol 2006;97:748–55.
23. **Nakamura T, Sugaya T, Kawagoe Y, et al.** Effect of pitavastatin on urinary liver-type fatty acid binding protein in patients with nondiabetic mild chronic kidney disease. Am J Nephrol 2006;26:82–6.

Copyright © by the Southern Society for Clinical Investigation. Unauthorized reproduction of this article is prohibited.
