# Supplementary material for: Effects of T-Type Calcium Channel Blockers on Renal Function and Aldosterone in Patients with Hypertension: A Systematic Review and Meta-Analysis
Source: PLoS One. 2014 Oct 17;9(10):e109834. doi: 10.1371/journal.pone.0109834 (PMC4201480; doi:10.1371/journal.pone.0109834)
Supplement: File S3 — PDF files of twenty-four studies included in the meta-analysis. (ZIP) [file pone.0109834.s007.zip › Supporting information-PDF files/25. Hypertens Res 2007[30(7)]621-626.pdf]

Original Article

Efonidipine Reduces Proteinuria and Plasma Aldosterone in Patients with Chronic Glomerulonephritis

Toshihiko ISHIMITSU[1], Tomoko KAMEDA[1], Akira AKASHIBA[1], Toshiaki TAKAHASHI[1], Satoshi OHTA[1], Masayoshi YOSHII[1], Junichi MINAMI[1], Hidehiko ONO[1], Atsushi NUMABE[1], and Hiroaki MATSUOKA[1]

Efonidipine, a dihydropirydine calcium channel blocker, has been shown to dilate the efferent glomerular arterioles as effectively as the afferent arterioles. The present study compared the chronic effects of efonidipine and amlodipine on proteinuria in patients with chronic glomerulonephritis. The study subjects were 21 chronic glomerulonephritis patients presenting with spot proteinuria greater than 30 mg/dL and serum creatinine concentrations of ≤1.3 mg/dL in men or ≤1.1 mg/dL in women. All patients were receiving antihypertensive medication or had a blood pressure ≥130/85 mmHg. Efonidipine 20–60 mg twice daily and amlodipine 2.5–7.5 mg once daily were given for 4 months each in a random crossover manner. In both periods, calcium channel blockers were titrated when the BP exceeded 130/85 mmHg. Blood sampling and urinalysis were performed at the end of each treatment period. The average blood pressure was comparable between the efonidipine and the amlodipine periods (133±10/86±5 *vs*. 132±8/86±5 mmHg). Urinary protein excretion was significantly less in the efonidipine period than in the amlodipine period (1.7±1.5 *vs*. 2.0±1.6 g/g creatinine, *p*=0.04). Serum albumin was significantly higher in the efonidipine period than the amlodipine period (4.0±0.5 *vs*. 3.8±0.5 mEq/L, *p*=0.03). Glomerular filtration rate was not significantly different between the two periods. Plasma aldosterone was lower in the efonidipine period than in the amlodipine period (52±46 *vs*. 72±48 pg/mL, *p*=0.009). It may be concluded that efonidipine results in a greater reduction of plasma aldosterone and proteinuria than amlodipine, and that these effects occur by a mechanism independent of blood pressure reduction. A further large-scale clinical trial will be needed in order to apply the findings of this study to the treatment of patients with renal disease. (*Hypertens Res* 2007; 30: 621–626)

***Key Words*: calcium channel blocker, efonidipine, chronic glomerulonephritis, proteinuria, aldosterone**

Introduction

According to the hyperfiltration theory, an increase in glomerular capillary pressure, *i.e.*, glomerular hypertension, plays an important role in the progression of diabetic and nondiabetic renal diseases (*1*, *2*). Intraglomerular capillary pressure is subject to the tone of the glomerular arterioles as well as the level of systemic arterial pressure. Because angiotensin II is a potent constrictor of the efferent arterioles, angiotensin converting enzyme (ACE) inhibitors and angiotensin II receptor blockers (ARB), which inhibit the generation or actions of angiotensin II, are effective in alleviating the glomerular hypertension (*3*). On the other hand, calcium channel blockers (CCBs) generally dilate the afferent arterioles and may not be as effective in reducing intraglomerular pressure as the inhibitors of the renin-angiotensin system (*4*).

Both L-type and T-type Ca channels are present in the

From the [1]Department of Hypertension and Cardiorenal Medicine, Dokkyo Medical University, Tochigi, Japan.
Address for Reprints: Toshihiko Ishimitsu, M.D., Department of Cardiovascular Medicine, Dokkyo Medical University, Mibu, Tochigi 321–0293, Japan. E-mail: isimitu@dokkyomed.ac.jp
Received August 10, 2006; Accepted in revised form March 15, 2007.

Table 1. Basic Characteristics of the Study Subjects

Pamameter	Value
Age (year)	54±13
Gender (men/women)	16/5
Body mas index (kg/m^2)	25.1±2.9
Histological diagnosis	
Membranous nephropathy	6
IgA nephropathy	6
Mesangial proliferative glomerulonephritis	4
Membranoproliferative glomerulonephritis	3
Minimal change	2
Systolic blood pressure (mmHg)	134±7
Diastolic blood pressure (mmHg)	88±7
Pulse rate (bpm)	71±7
Antihypertensive medication	
Diuretics	4
β-Blockers	1
Calcium channel blockers	6
ACE inhibitors	9
Angiotensin II receptor antagonists	8
Drugs for glomerulonephritis	
Steroids	3
Immunosuppressants	1
Antiplatelet drugs	15
Serum creatinine (mg/dL)	0.87±0.20

Data are mean±SD. ACE, angiotesin converting enzyme.

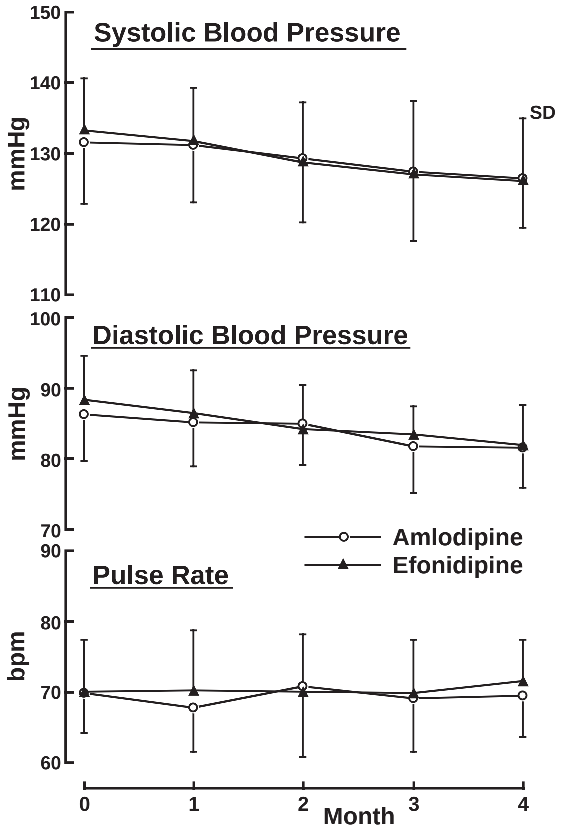

Fig. 1. *Changes in systolic blood pressure (SBP; upper), diastolic blood pressure (DBP; middle) and pulse rate (lower) during the period of treatment with amlodipine or efonidipine.*

afferent arterioles, while only T-type Ca channels are present in the efferent arterioles. Because most dihydropyridine CCBs principally block the L-type Ca channels, they preferentially reduce the afferent arteriolar resistance. Efonidipine, a dihydropyridine CCB, has been shown to block both L-type and T-type Ca channels, and experimental studies have shown that efonidipine dilates both the efferent and afferent arterioles (*5*–*7*). This property of efonidipine may confer protective effects against the progression of renal injuries by alleviating glomerular hypertension; however, its clinical implications in patients with renal disease have not been examined. In addition, aldosterone has been shown to influence the urinary protein excretion in patients with chronic renal disease (*8*), and an *in vitro* experiment has demonstrated that T-type CCBs inhibit the aldosterone production of cultured adrenal cells (*9*). We therefore considered that it would be intriguing to compare the effects of a T-type CCB and an L-type CCB on proteinuria and on the renin-angiotensin-aldosterone system (RAAS) in patients with renal disease.

In this random crossover study, we compared the clinical effects of efonidipine, an L- and T-type CCB, with amlodipine, an L-type CCB, in patients with chronic glomerulonephritis.

Methods

We enrolled a total of 21 biopsy-proven chronic glomerulonephritis patients presenting spot proteinuria greater than 30 mg/dL and serum creatinine concentrations of ≤1.3 mg/dL in men or ≤1.1 mg/dL in women. All patients were receiving antihypertensive medication or had a blood pressure exceeding 130 mmHg in systole or 85 mmHg in diastole. The study protocol was in accordance with the recommendations of the World Medical Association for biomedical research involving human subjects (Edinburgh version, 2000) and was approved by the institutional review board. Informed consent was obtained from all subjects. After discontinuing all antihypertensive medications, each patient underwent a 4-month treatment period with efonidipine and a 4-month treatment period with amlodipine. The order of the study drugs was randomized blindly. During the study periods, 20–60 mg efonidipine was given twice daily after breakfast and supper, and 2.5–7.5 mg amlodipine was given once daily after breakfast. In order to achieve comparable blood pressure control between the two study periods, the doses of efonidipine and

Table 2. Body Weight and Laboratory Data at the End of Period Given Amlodipine or Efonidipine

Variable	Amlodipine period	Efonidipine period
Body weight (kg)	67.4±8.2	67.2±8.4
Blood cell count		
White blood cell (×10³/mm³)	6.76±1.97	6.53±1.77
Red blood cell (×10⁶/mm³)	4.71±0.61	4.72±0.59
Hemoglobin (g/dL)	14.7±1.6	14.8±1.8
Hematocrit (%)	44.3±5.0	44.5±5.2
Platelet (×10³/mm³)	23.6±7.0	23.3±6.2
Blood chemistry		
AST (U/L)	28±8	31±12
ALT (U/L)	29±16	32±19
Total protein (g/dL)	6.9±0.8	7.1±0.8
Albumin (g/dL)	3.8±0.5	4.0±0.5 *
Na (mEq/L)	142±2	142±2
K (mEq/L)	4.2±0.3	4.3±0.3
Urea nitrogen (mg/dL)	16±3	16±4
Creatinine (mg/dL)	0.89±0.22	0.91±0.25
Uric acid (mg/dL)	5.8±0.9	6.1±1.0
Glucose (mg/dL)	102±11	100±12
Total cholesterol (mg/dL)	203±37	212±45
HDL-cholesterol (mg/dL)	45±8	49±14
Triglycerides (mg/dL)	136±84	151±104

Data are mean±SD. *$p<0.05$ *vs.* the amlodipine period. AST, aspartate aminotransferase; ALT, alanine aminotransferase; HDL, high-density lipoprotein.

amlodipine were titrated so that the blood pressure was reduced to below 130 mmHg in systole and below 85 mmHg in diastole. These values corresponded to the target blood pressure level in patients with renal parenchymal disease recommended by The Sixth Report of the Joint National Committee on Prevention, Detection, Evaluation, and Treatment of high Blood Pressure (JNC VI) (*10*). Blood pressure was measured by a sphygmomanometer at 2- to 4-week intervals with the patient in a sitting position after more than 20 min of sitting rest.

At the end of both the efonidipine and amlodipine administration periods, blood samples were collected for blood cell counts, blood chemistry and the measurements of plasma angiotensin II and aldosterone. Plasma concentrations of angiotensin II and aldosterone were directly radioimmunoassayed using an Angiotensin II RIA kit (SRL Inc., Tokyo, Japan) and Aldosterone RIA kit II (Dinabbot & Co., Ltd., Tokyo, Japan), respectively. Blood samples were taken from the antecubital vein after an overnight fast and 30 min of supine rest. Glomerular filtration rate (GFR) was calculated from the serum creatinine concentration, age and body weight using the Cockcroft-Gault formula (*11*). The first urine in the morning was used for the urinalysis. Urinary concentrations of protein and creatinine were measured by colorimetry and

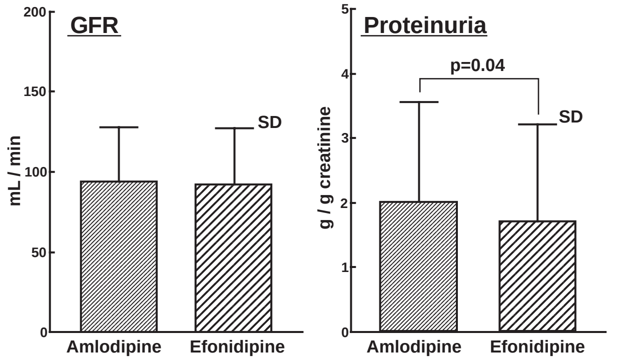

Fig. 2. *Glomerular filtration rate (GFR) (left) and urinary protein excretion (right) at the end of the period of amlodipine or efonidipine administration.*

urinary protein excretion was expressed as a ratio to the urinary creatinine concentration. The averaged value of the last 3 visits of each study period was used for the evaluation of urinary protein excretion.

Values are expressed as the means±SD. Clinical and laboratory data between the two groups were compared by Student's *t*-test for paired samples. A *p* value less than 0.05 was considered to indicate statistical significance.

Results

Table 1 shows the background characteristics of the study subjects. Membranous nephropathy and IgA nephropathy were the most frequent histological diagnoses followed by mesangial proliferative glomerulonephritis and membranoproliferative glomerulonephritis. Two patients had persistent proteinuria with minor glomerular histological abnormalities. The diagnosis of chronic glomerulonephritis was made based on biopsy as described above, and was supplemented by reference to the medical history, physical findings, laboratory data and analyses of radiological and ultrasound images. Nineteen out of 22 patients had been taking antihypertensive drugs. ACE inhibitors and ARBs were the most frequent antihypertensive drugs used followed by CCBs. The averaged blood pressure of the study subjects was below 140/90 mmHg but higher than 130/85 mmHg before entering the study.

Eleven patients started with the efonidipine period and 10 patients started with the amlodipine period. All 21 patients completed the study periods without experiencing adverse side effects. The average doses of efonidipine and amlodipine were 38±18 mg/day and 4.3±1.7 mg/day, respectively. Figure 1 shows the changes in blood pressure and pulse rate during the study periods. Blood pressure, both in systole and diastole, was gradually lowered during the period of treatment with efonidipine or amlodipine. The blood pressure levels, and their changes, were comparable between the efonidipine and the amlodipine periods. In patients who were initially administered efonidipine, the average systolic and

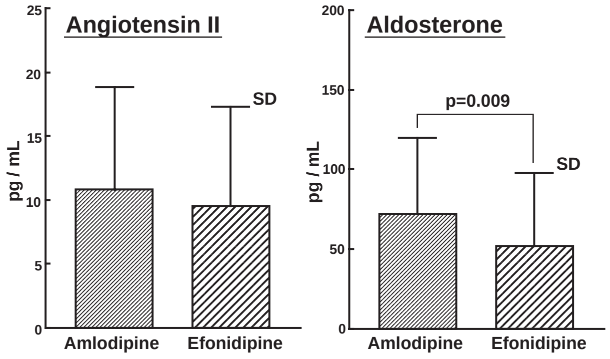

Fig. 3. *Plasma levels of angiotensin II (left) and aldosterone (right) at the end of the period of amlodipine or efonidipine administration.*

diastolic blood pressures were decreased by 0.9±5.3 mmHg and 0.5±3.2 mmHg, respectively, during the amlodipine period, but the changes were not statistically significant. In patients initially treated with amlodipine, systolic blood pressure was insignificantly increased by 1.4±3.8 mmHg and diastolic blood pressure was insignificantly decreased by 0.3±2.2 mmHg. At the ends of the efonidipine and the amlodipine periods, 15 patients (71%) and 16 patients (76%) achieved the target blood pressure level (≤130/85 mmHg). The pulse rate did not show significant changes during either the efonidipine or amlodipine treatment period. Table 2 lists the body weights and laboratory data at the end of each treatment period. The body weight did not differ significantly between the two treatment periods. There were also no significant differences in blood cell counts, serum liver enzymes, serum electrolytes, indices of renal function, serum lipids or blood glucose between the two periods. However, serum albumin was significantly higher in the efonidipine period than in the amlodipine period.

Figure 2 compares GFR and urinary protein excretion between the efonidipine and the amlodipine periods. Although GFR did not significantly differ between the two periods, urinary protein excretion was reduced by 15% in the efonidipine period as compared with the amlodipine period. The reduction in proteinuria was comparable between patients who were initially administered efonidipine and those initially given amlodipine (−16±13% and −14±15%, respectively). Figure 3 shows the plasma concentrations of angiotensin II and aldosterone at the end of each treatment period. The level of plasma angiotensin II was not significantly different between the two periods, but the plasma aldosterone level was significantly lower during the efonidipine administration period than during the amlodipine period. This intergroup difference in plasma aldosterone was not significantly affected by whether efonidipine or amlodipine was administered initially (25±22% *vs*. 34±28%, respectively). There was no significant correlation between the differences in plasma aldosterone and proteinuria.

Discussion

In order to protect the kidneys from diabetic and nondiabetic renal diseases, it is important to prevent the increase in glomerular capillary pressure. To this end, inhibitors of the renin-angiotensin system, such as ACE inhibitors and ARBs, are expected to improve glomerular hypertension by reducing the constrictive effect of angiotensin II on the efferent arterioles. With regard to the Ca channels in the glomerular arterioles, the afferent arterioles express the L- and T-type Ca channels, while the efferent arterioles express only the T-type Ca channel (*6*). This distribution of Ca channels may explain the experimental observation that efonidipine, the L- and T-type CCB, dilates both the afferent and efferent arterioles in the isolated perfused kidney, while the L-type CCB preferentially dilates the afferent arterioles (*5*). Therefore, efonidipine is thought to have an advantage in reducing glomerular capillary pressure as compared with other CCBs. The elevated glomerular hydraulic pressure promotes ultrafiltration of plasma proteins, resulting in proteinuria. In the present study, the patients were selected by semi-quantitative evaluation of spot proteinuria; however, the urinary protein to creatinine ratio of the first urine in the morning, which is less subject to the influence of urine volume and physical exercise, was lower in the period of efonidipine administration than in the period of amlodipine administration. This may have been because the two CCBs exerted different actions on efferent arteriolar tone and glomerular pressure. However, a further increase in the dose of amlodipine may also reduce the intraglomerular pressure as well as the systemic arterial pressure and may be effective in reducing proteinuria.

According to the analyses of the large-scale clinical study outcomes, the amount of urinary protein excretion was predictive for the rate of deterioration of renal function in patients with diabetic and nondiabetic renal diseases (*12*–*15*). Proteinuria itself is detrimental to the kidney because ultrafiltration of proteins across the glomerular basement membrane brings about mesangial and tubular protein overload, which provokes inflammation and ultimately results in glomerulosclerosis and tubulo-interstitial fibrosis (*16*, *17*). It is also known that proteinuria is associated with the risk of cardiovascular diseases, and that proteinuria is a modifiable cardiovascular risk factor (*18*–*20*). Therefore, the antiproteinuric effect of efonidipine would seem to make this drug more advantageous than an L-type CCB not only for slowing the progression of renal tissue injuries but also for reducing the incidence of cardiovascular events in patients with renal diseases. In terms of reducing proteinuria in diabetic and nondiabetic renal diseases, ACE inhibitors and ARBs have been shown to be more effective than other classes of antihypertensive drugs (*21*, *22*). However, considering that the antiproteinuric effect of efonidipine is not mediated by angiotensin II inhibition but is considered to be brought about by the blockade of T-type Ca channels in the glomerular arterioles, the use

of efonidipine in combination with the renin-angiotensin system inhibitors may yield a further reduction of proteinuria in renal disease patients.

Efonidipine has been shown to inhibit aldosterone synthesis in cultured adrenocortical cells *via* T-type Ca channel blockade (*9*). It has also been observed that acute administration of efonidipine lowers plasma aldosterone in healthy subjects (*23*). In the present study, plasma aldosterone was lower in the efonidipine period than in the amlodipine period. Therefore, in addition to lowering blood pressure, efonidipine would be expected to provide renoprotection by inhibiting aldosterone production. Long-term treatment with ACE inhibitors or ARBs can cause escape or rebound of plasma aldosterone. In such a situation, addition of a mineralocorticoid receptor blocker has been shown to reduce proteinuria in patients with chronic renal diseases (*8*). Accordingly, in patients taking inhibitors of the RAAS for the treatment of hypertension and renal disease, a T-type CCB might be a suitable addition for purpose of inhibiting aldosterone production and managing aldosterone escape. However, the results of this study do not suggest that the aldosterone decrease contributed to the reduction in proteinuria by efonidipine, because there was not a significant correlation between the differences in plasma aldosterone and proteinuria in this study. It appears that an additional study with a larger number of cases will be needed to analyze the relation between the changes in plasma aldosterone and proteinuria. In addition, considering the results of *in vitro* studies, a higher dose of other dihydropyridine CCBs may also inhibit the aldosterone synthesis by the adrenal cells (*9*).

A recent clinical study has indicated that efonidipine, but not nifedipine, improves the indices of endothelial function and oxidative stress in hypertensive patients (*24*). Endothelial dysfunction is recognized to be the initial step of atherosclerosis, and increased oxidative stress is thought to promote cardiovascular tissue injuries (*25–27*). On the other hand, much attention has been paid to the involvement of the RAAS in the process of development and progression of cardiovascular tissue and organ injuries (*28*, *29*). In addition, it is recognized that chronic kidney disease greatly increases the risk of cardiovascular morbidity and mortality (*30*), and proteinuria is associated with the risk of cardiovascular diseases (*16–18*). The results of the present study and the earlier studies thus far reported indicate that efonidipine reduces oxidative stress, improves endothelial function, inhibits aldosterone production and alleviates proteinuria in addition to lowering blood pressure. These properties of efonidipine may confer the benefit of additional cardiovascular and renal protection during the management of hypertension especially in patients with chronic renal diseases.

Regarding the limitations of this study, monotherapy with a CCB was performed throughout the study periods. Consequently, 20–30% of patients did not achieve the targeted blood pressure. In the practical management of renal disease patients, combination therapy is recommended in order to achieve strict blood pressure control. Thus, it should be kept in mind that the antihypertensive therapy performed in this study is not necessarily an ideal treatment for patients with renal disease.

In summary, the present study showed that efonidipine, an L-type and T-type CCB, resulted in a greater reduction of plasma aldosterone and proteinuria than amlodipine, an L-type CCB, in patients with chronic glomerulonephritis. These effects of efonidipine would appear to make the drug more advantageous than amlodipine in terms of slowing the progression of renal dysfunction and preventing cardiovascular tissue and organ injuries in patients with hypertension and chronic kidney disease. However, before the results of this random crossover study can be applied to the treatment of patients with renal disease, a further large-scale clinical trial should be conducted.

Acknowledgements

The authors thank Ms. Masako Minato, Ms. Machiko Sakata, Ms. Mika Nomura, Ms. Yasuko Mamada, and Ms. Jun Chou for their assistance in performing the study and preparing the manuscript.

References

1. Hostetter TH, Olson JL, Rennke HG, Venkatachalam MA, Brenner BM: Hyperfiltration in remnant nephrons: a potentially adverse response to renal ablation. *Am J Physiol* 1981; **241**: F85–F93.
2. Brenner BM, Lawler EV, Mackenzie HS: The hyperfiltration theory: a paradigm shift in nephrology. *Kidney Int* 1996; **49**: 1774–1777.
3. Remuzzi G, Perico N, Macia M, Ruggenenti P: The role of renin-angiotensin-aldosterone system in the progression of chronic kidney disease. *Kidney Int Suppl* 2005; **99**: S57–S65.
4. Carmines PK, Mitchell KD, Navar LG: Effects of calcium antagonists on renal hemodynamics and glomerular function. *Kidney Int Suppl* 1992; **36**: S43–S48.
5. Hayashi K, Ozawa Y, Fujiwara K, Wakino S, Kumagai H, Saruta T: Role of actions of calcium antagonists on efferent arterioles—with special references to glomerular hypertension. *Am J Nephrol* 2003; **23**: 229–244.
6. Tanaka H, Shigenobu K: Pathophysiological significance of T-type Ca^{2+} channels: T-type Ca^{2+} channels and drug development. *J Pharmacol Sci* 2005; **99**: 214–220.
7. Richard S: Vascular effects of calcium channel antagonists: new evidence. *Drugs* 2005; **65** (Suppl 2): 1–10.
8. Sato A, Hayashi K, Saruta T: Antiproteinuric effects of mineralocorticoid receptor blockade in patients with chronic renal disease. *Am J Hypertens* 2005; **18**: 44–49.
9. Imagawa K, Okayama S, Takaoka M, *et al*: Inhibitory effect of efonidipine on aldosterone synthesis and secretion in human adrenocarcinoma (H295R) cells. *J Cardiovasc Pharmacol* 2006; **47**: 133–138.
10. Joint National Committee on Prevention, Detection, Evaluation, and Treatment of High Blood Pressure: The sixth

report of the Joint National Committee on prevention, detection, evaluation, and treatment of high blood pressure. *Arch Intern Med* 1997; **157**: 2413–2446.
11. Cockcroft DW, Gault MH: Prediction of creatinine clearance from serum creatinine. *Nephron* 1976; **16**: 31–41.
12. Peterson JC, Adler S, Burkart JM, *et al*: Blood pressure control, proteinuria, and the progression of renal disease. The Modification of Diet in Renal Disease Study. *Ann Intern Med* 1995; **123**: 754–762.
13. Remuzzi G, Chiurchiu C, Ruggenenti P: Proteinuria predicting outcome in renal disease: nondiabetic nephropathies (REIN). *Kidney Int Suppl* 2004; **92**: S90–S96.
14. de Zeeuw D, Remuzzi G, Parving HH, *et al*: Proteinuria, a target for renoprotection in patients with type 2 diabetic nephropathy: lessons from RENAAL. *Kidney Int* 2004; **65**: 2309–2320.
15. Hunsicker LG, Atkins RC, Lewis JB, *et al*: Impact of irbesartan, blood pressure control, and proteinuria on renal outcomes in the Irbesartan Diabetic Nephropathy Trial. *Kidney Int Suppl* 2004; **92**: S99–S101.
16. Abbate M, Benigni A, Bertani T, Remuzzi G: Nephrotoxicity of increased glomerular protein traffic. *Nephrol Dial Transplant* 1999; **14**: 304–312.
17. Abbate M, Zoja C, Morigi M, *et al*: Transforming growth factor-beta1 is up-regulated by podocytes in response to excess intraglomerular passage of proteins: a central pathway in progressive glomerulosclerosis. *Am J Pathol* 2002; **161**: 2179–2193.
18. Kannel WB, Stampfer MJ, Castelli WP, Verter J: The prognostic significance of proteinuria: the Framingham study. *Am Heart J* 1984; **108**: 1347–1352.
19. Culleton BF, Larson MG, Parfrey PS, Kannel WB, Levy D: Proteinuria as a risk factor for cardiovascular disease and mortality in older people: a prospective study. *Am J Med* 2000; **109**: 1–8.
20. Dykeman-Sharpe J: Proteinuria, a modifiable risk factor: angiotensin converting enzyme inhibitors (ACEIs) and angiotensin II receptor blockers (ARBs). *CANNT J* 2003; **13**: 34–38.
21. Raij L: Recommendations for the management of special populations: renal disease in diabetes. *Am J Hypertens* 2003; **16**: 46S–49S.
22. Ruggenenti P: Angiotensin-converting enzyme inhibition and angiotensin II antagonism in nondiabetic chronic nephropathies. *Semin Nephrol* 2004; **24**: 158–167.
23. Okayama S, Imagawa K, Naya N, *et al*: Blocking T-type Ca^{2+} channels with efonidipine decreased plasma aldosterone concentration in healthy volunteers. *Hypertens Res* 2006; **29**: 493–497.
24. Oshima T, Ozono R, Yano Y, *et al*: Beneficial effect of T-type calcium channel blockers on endothelial function in patients with essential hypertension. *Hypertens Res* 2005; **28**: 889–894.
25. Ross R: Atherosclerosis—an inflammatory disease. *N Engl J Med* 1999; **340**: 115–126.
26. Landmesser U, Hornig B, Drexler H: Endothelial function: a critical determinant in atherosclerosis? *Circulation* 2004; **109** (Suppl 1): II27–II33.
27. Madamanchi NR, Vendrov A, Runge MS: Oxidative stress and vascular disease. *Arterioscler Thromb Vasc Biol* 2005; **25**: 29–38.
28. Brewster UC, Setaro JF, Perazella MA: The renin-angiotensin-aldosterone system: cardiorenal effects and implications for renal and cardiovascular disease states. *Am J Med Sci* 2003; **326**: 15–24.
29. Struthers AD, MacDonald TM: Review of aldosterone- and angiotensin II–induced target organ damage and prevention. *Cardiovasc Res* 2004; **61**: 663–670.
30. Tonelli M, Wiebe N, Culleton B, *et al*: Chronic kidney disease and mortality risk: a systematic review. *J Am Soc Nephrol* 2006; **17**: 2034–2047.
