# Supplementary material for: Effects of T-Type Calcium Channel Blockers on Renal Function and Aldosterone in Patients with Hypertension: A Systematic Review and Meta-Analysis
Source: PLoS One. 2014 Oct 17;9(10):e109834. doi: 10.1371/journal.pone.0109834 (PMC4201480; doi:10.1371/journal.pone.0109834)
Supplement: File S3 — PDF files of twenty-four studies included in the meta-analysis. (ZIP) [file pone.0109834.s007.zip › Supporting information-PDF files/26. Hypertens Res 2005[28(11)]889-894.pdf]

Original Article

Beneficial Effect of T-Type Calcium Channel Blockers on Endothelial Function in Patients with Essential Hypertension

Tetsuya OSHIMA, Ryoji OZONO, Yoko YANO, Yukihito HIGASHI*,
Hiroki TERAGAWA**, Narimasa MIHO**, Takafumi ISHIDA**,
Mari ISHIDA**, Masao YOSHIZUMI*, and Masayuki KAMBE

Endothelial function is impaired in essential hypertension. T-type but not L-type voltage-gated Ca^{2+} channels were detected in the vascular endothelium. The purpose of the present study was to clarify the role of T-type Ca^{2+} channels in endothelial function. We studied flow-mediated vasodilation (FMD) and sublingual nitroglycerin (NTG)-induced vasodilation in the brachial artery. Forty patients with essential hypertension were randomly assigned to treatment with efonidipine, a T- and L-type Ca^{2+} channel blocker, or with nifedipine, an L-type Ca^{2+} channel blocker. Twenty healthy normotensive individuals were included as a control group. In patients with essential hypertension, FMD was attenuated and NTG was similar that of compared to healthy controls. After 12 weeks, the decrease in mean blood pressure in the efonidipine and nifedipine groups were similar. The endothelial function index, a ratio of FMD/NTG, was significantly increased by efonidipine (73±24 to 94±20%) but unchanged by nifedipine. Urinary excretion 8-hydroxy-2′-deoxyguanosine (8-OHdG) and serum malondialdehyde-modified low-density lipoprotein (LDL) were decreased by efonidipine but unchanged by nifedipine. These results suggest that a T-type Ca^{2+} channel blocker, but not an L-type Ca^{2+} channel blocker, may improve vascular endothelial dysfunction in patients with essential hypertension *via* a reduction in oxidative stress. (*Hypertens Res* 2005; 28: 889–894)

***Key Words*: calcium, endothelium, nitroglycerin, vasodilation, ion channels**

Introduction

Atherosclerosis, which results in coronary heart diseases and stroke, is one of the main factors contributing to complications of hypertension. Endothelial dysfunction is recognized to be the first step of atherosclerosis (*1*) and may be involved in the development of cardiovascular and cerebrovascular diseases. Endothelium-dependent vascular relaxation has been found to be impaired in brachial (*2*, *3*), renal (*4*), and coronary arteries (*5*) of patients with essential hypertension, since oxidative stress causes a reduction in NO availability. Moreover, endothelial dysfunction and oxidative stress are predictors of cardiovascular events (*6*). It is important for the establishment of an appropriate therapeutic strategy to determine whether or not antihypertensive treatment improves endothelial dysfunction in addition to lowering blood pressure in patients with essential hypertension.

From the Department of Clinical Laboratory Medicine, *Department of Cardiovascular Physiology and Medicine, and **Department of Medicine and Molecular Science, Hiroshima University Graduate School of Biomedical Sciences, Hiroshima, Japan.
This study was supported in part by a Grant-in-Aid for Scientific Research (No. 15390177) from the Ministry of Education, Culture, Sports, Science and Technology of Japan.
Address for Reprints: Tetsuya Oshima, M.D,. Ph.D., Department of Clinical Laboratory Medicine, Hiroshima University Graduate School of Biomedical Sciences, 1–2–3 Kasumi, Minami-ku, Hiroshima 734–8551, Japan. E-mail: teto@hiroshima-u.ac.jp
Received July 7, 2005; Accepted in revised form September 5, 2005.

Table 1. Clinical Characteristics of Hypertensive Patients and Normotensive Controls

Parameter	Normotensive (n=20)	Hypertensive (n=40)
Body mass index (kg/m^2)	22.2±3.0	23.6±3.3
Systolic blood pressure (mmHg)	115±9	153±14*
Diastolic blood pressure (mmHg)	69±7	93±7*
Heart rate (bpm)	66±6	63±6
Serum total cholesterol (mmol/l)	4.6±1.0	4.5±1.1
Serum malondialdehyde-modified LDL (U/l)	69±19	119±29*
Serum triglyceride (mmol/l)	1.3±0.5	1.4±0.6
Serum glucose (mmol/l)	4.8±0.5	4.9±0.4
Serum creatinine (μmol/l)	86±22	88±27
Urinary 8-OHdG (ng/mg of creatinine)	10±4	15±8*

LDL, low-density lipoprotein; 8-OHdG, 8-hydroxy-2′-deoxyguanosine. *p<0.05 *vs*. normotensive controls.

Table 2. Vascular Studies of Hypertensive Patients and Normotensive Controls

Parameter	Normotensive (n=20)	Hypertensive (n=40)
Baseline diameter (mm)	3.90±0.60	3.91±0.58
Baseline blood flow (ml/min)	40±14	43±17
Hyperemia flow (ml/min)	260±44	257±41
FMD (%)	11.0±2.0	9.7±3.7*
NTG (%)	13.3±2.1	13.3±2.2
EFI (%)	82.7±24.0	72.7±22.1*

FMD, flow-mediated vasodilation; NTG, nitroglycerin-induced vasodilation; EFI, endothelial function index. *p<0.05 *vs*. normotensive controls.

Ca^{2+} channel blockers are most frequently used as antihypertensive drugs (*7*), and much interest has been shown in their effects on endothelial function. However, the effects of Ca^{2+} channel blockers on endothelial function are controversial (*8–12*). Negative findings regarding Ca^{2+} channel blockers may be expected due to the finding that vascular endothelial cells lack L-type Ca^{2+} channels. Recently, T-type Ca^{2+} channels have been detected in vascular endothelial cells (*13–15*), although their roles have not been clarified. In the present study, in order to determine the role of T-type Ca^{2+} channels in vascular endothelial cells, the effects on the endothelium-dependent vasodilation of brachial arteries exerted by efonidipine, a T-type and L-type Ca^{2+} channel blocker (*16*), and nifedipine, an L-type Ca^{2+} channel blocker, were comparatively studied in patients with essential hypertension.

Methods

Subjects

We recruited 40 Japanese patients (26 men and 14 women; mean age, 55±11 years old) with mild to moderate essential hypertension. Hypertension was defined as systolic blood pressure of >140 mmHg and/or diastolic blood pressure of >90 mmHg, measured on at least 3 different occasions while subjects were in a sitting position. Measurements were performed in the outpatient clinic of Hiroshima University Hospital. Patients with secondary forms of hypertension were excluded on the basis of appropriate clinical, biochemical, and radiological examinations. None of the patients had diabetes mellitus, hypercholesterolemia, liver disease, or renal failure. Patients with a medical history of cardiovascular or cerebrovascular disease were excluded from the study. As a control group, twenty healthy normotensive individuals (13 men and 7 women; 54±10 years old) were recruited from among members of the medical staff and people undergoing annual examinations. Normal blood pressure was defined as a systolic blood pressure of less than 130 mmHg and a diastolic blood pressure of less than 80 mmHg. The normotensive control individuals had normal findings on physical and laboratory examinations. The study protocol was approved by the Ethics Committee of Hiroshima University Faculty of Medicine. Informed consent for participation in the study was obtained from all subjects.

Treatment Protocol

No patient had a history of antihypertensive treatment before the study. Hypertensive patients were randomly assigned in a double-blind fashion to treatment with efonidipine or nifedipine-controlled release (CR). A 4-week run-in period with a placebo was followed by a 12-week treatment period. During the first 4 weeks of the active treatment period, patients were treated with single daily doses of efonidipine (20 mg) or nifedipne-CR (20 mg) in the morning. When diastolic blood pressure was found to be >85 mmHg or a decrease of <10 mmHg was seen at the end of the first 4 weeks, doses were increased to 40 mg during the following 4-week period.

Ultrasound Studies of the Brachial Artery

High-resolution ultrasound (Philips, SONOS 550, Amsterdam, The Netherlands) and a broad-band (multiple frequency: 8–15 MHz) linear array transducer (Philips, 15-6L) were used to measure arterial diameter in response to increased flow (flow-mediated vasodilation: FMD) and in response to nitroglycerin (NTG) spray (400 μg) as previously described (*17*). The study began at 9:00 AM after the subjects had completed an overnight fast. All smokers abstained from smoking for at least 3 h before the forearm vessel study.

Increased flow was then achieved by inflation of a pneumatic tourniquet placed around the forearm to a pressure of 250 mmHg followed by rapid cuff deflation after 5 min. Flow

Table 3. Clinical Characteristics of Hypertensive Patients before and after Treatment

Parameter	Efonidipine ($n=20$)		Nifedipine ($n=20$)	
	Before	After	Before	After
Body mass index (kg/m^2)	22.3±3.3	22.4±3.3	23.8±3.2	23.9±3.3
Systolic blood pressure (mmHg)	152±14	132±13*	153±14	130±12*
Diastolic blood pressure (mmHg)	92±7	81±6*	93±7	80±6*
Heart rate (bpm)	63±6	64±6	63±6	65±7
Serum total cholesterol (mmol/l)	4.5±1.1	4.4±1.0	4.4±1.0	4.4±1.1
Serum malondialdehyde-modified LDL (U/l)	119±30	71±20*	119±28	125±24
Serum triglyceride (mmol/l)	1.4±0.6	1.3±0.6	1.3±0.5	1.4±0.7
Serum glucose (mmol/l)	4.8±0.4	4.7±0.4	4.9±0.4	4.8±0.4
Serum creatinine (μmol/l)	87±27	85±26	88±27	87±26
Urinary 8-OHdG (ng/mg of creatinine)	16±9	11±4*	14±7	15±6

LDL, low-density lipoprotein; 8-OHdG, 8-hydroxy-2′-deoxyguanosine. *$p<0.05$ *vs*. before.

was calculated based on velocity and vessel diameter. Reactive hyperemia was calculated as the maximum flow after cuff deflation.

Laboratory Analyses

Routine chemical methods were used to determine serum concentrations of cholesterol, triglyceride, creatinine and glucose. The urinary concentrations of 8-hydroxy-2′-deoxyguanosine (8-OHdG) were assayed by an enzyme-linked immunosorbent assay (ELISA) with the use of 8-OHdG kits (Nihon Yushi, Tokyo, Japan). The serum concentrations of malondialdehyde-modified low-density lipoprotein (LDL) were also assayed by ELISA (antimalondialdehyde-modified LDL antibody, SRL, Tokyo, Japan).

Statistical Analysis

Results are presented as means±SD. Values of $p<0.05$ were considered significant. The Mann-Whitney *U* test was used to evaluate differences between normotensive controls and essential hypertensive patients, and between the efonidipine and the nifedipine groups in parameters at baseline before treatment. Comparisons between treatment groups with respect to changes in parameters were performed with adjusted means by ANCOVA using the baseline data as covariates.

Results

Clinical Characteristics and Vascular Studies to Compare Hypertensive Patients and Normotensive Controls

The clinical characteristics of normotensive individuals ($n=20$, 13 men and 7 women; mean age: 54±10 years old; age range: between 40 and 67 years old) and hypertensive patients ($n=40$, 26 men and 14 women; mean age: 55±11 years old; age range: between 41 and 70 years old) are summarized in Table 1. Blood pressure, serum malondialdehyde-modified LDL and urinary 8-OHdG excretion were significantly greater in patients with essential hypertension than in normotensive controls. The results for the other parameters were similar in the two groups.

Vascular studies of normotensive controls and hypertensive patients are shown in Table 2. Baseline diameter and blood flow of the brachial artery were similar in normotensives and hypertensives. FMD was significantly lower in patients with essential hypertension than in normotensive controls, whereas the response to NTG was similar in the two groups. The ratio of response to intrinsic NO/response to exogenous NO (FMD/NTG) was considered as the endothelial function index, which was significantly lower in hypertensives than in normotensives.

Effects of Efonidipine and Nifedipine on Baseline Clinical Characteristics

Using a double-blind, randomized, and parallel method, 40 patients were divided into an efonidipine group ($n=20$, 13 men and 7 women; mean age: 56±11 years old) and a nifedipine group ($n=20$, 13 men and 7 women; mean age: 55±10 years old). No patients in either group withdrew. To achieve blood pressure control, 20 patients received an efonidipine dose of 25.0±8.9 mg/day and 20 patients received a nifedipine dose of 24.0±8.2 mg/day. There were no significant differences in age or gender between the two groups. The average of blood pressure level and severity of hypertension were similar in the two groups. The baseline values for the other parameters in the efonidipine and nifedipine groups at the beginning of the treatment period were similar, as shown in Table 3. As based on the results of echocardiography (left ventricular mass index >110 g/m^2 for women and >134 g/m^2 for men), left ventricular hypertrophy was present in 6 patients in the efonidipine group and 7 patients in the nifedipine group. Five patients with apparent proteinuria (>30 mg/dl) were in the efonidipine group and 5 in the nifedipine

Table 4. Vascular Studies of Hypertensive Patients before and after Treatment

Parameter	Efonidipine (n=20)		Nifedipine (n=20)	
	Before	After	Before	After
Baseline diameter (mm)	3.91±0.65	4.09±0.63*	3.90±0.58	4.10±0.66*
Baseline blood flow (ml/min)	42±15	50±16*	43±18	52±18*
Hyperemia flow (ml/min)	257±48	262±40	256±33	260±42
FMD (%)	9.6±3.3	9.5±2.9	9.7±3.0	8.0±2.0*
NTG (%)	13.2±2.1	10.9±2.2*	13.4±2.2	10.8±2.0*
EFI (%)	73.2±24.1	88.3±26.3*	72.2±20.1	74.0±22.0

FMG, flow-mediated vasodilation; NTG, nitroglycerin-induced vasodilation; EFI, endothelial function index. *$p<0.05$ *vs*. before.

group. Two patients in each group were current smokers.

The effects of efonidipine and nifedipine on the baseline values of the parameters are shown in Table 3. After 12 weeks, the magnitude of decrease in blood pressure in the efonidipine and nifedipine groups was similar. Blood pressure was significantly reduced with efonidipine treatment, *i.e.*, from 152±14/92±7 to 132±13/81±6 mmHg (all $p<0.01$) and with nifedipine treatment, *i.e.*, from 153±14/93±7 to 130±12/80±6 mmHg (all $p<0.01$). Other parameters such as lipid and glucose metabolism remained unchanged by either of the antihypertensive treatments. The serum concentration of malondialdehyde-modified LDL and urinary excretion of 8-OHdG were significantly reduced by treatment with efonidipine ($p<0.05$) but remained unchanged by nifedipine treatment.

Vascular Studies at the Beginning and End of the 12-Week Treatment Period

Basal diameter, basal blood flow, hyperemic blood flow, FMD, and the response to NTG at the beginning of the treatment period were similar in both the efonidipine and nifedipine groups, as shown in Table 4.

Basal diameter and basal blood flow of the brachial artery were increased by both types of antihypertensive treatment. The increases in these parameters with treatment were similar in the efonidipine and nifedipine groups. Hyperemic blood flow did not change with either treatment. FMD was decreased by nifedipine, but was not changed by efonidipine treatment. The response to NTG was significantly reduced by both efonidipine and nifedipine. The magnitude of decrease in the response to NTG in the two treatment groups was similar. The endothelial function index was significantly enhanced by efonidipine treatment, but this value did not change with nifedipine treatment. The change in the endothelial function index was not associated with either pre-treatment blood pressure or the presence of target organ damage (left ventricular hypertrophy or proteinuria).

Discussion

The ultimate goal of antihypertensive treatment is the prevention of cardiovascular and cerebrovascular complications of hypertension. According to the response-to-injury hypothesis (*1*), endothelial dysfunction is the first step of the process leading to atherosclerosis and may be involved in the pathogenesis of target organ damage resulting from hypertension. Therefore, the use of antihypertensive agents a therapeutic strategy appears to be important both for the improvement of endothelial dysfunction, as well as for the reduction of blood pressure in patients with hypertension. Although there have been several reports on the effects of Ca^{2+} channel blockers on *in vivo* endothelial function, these results have remained controversial (*8*–*12*).

In the present study, endothelial dysfunction was improved by efonidipine, a T-type and L-type Ca^{2+} channel blocker, but remained unaffected by nifedipine, an L-type Ca^{2+} channel blocker. These findings suggest that the inhibition of T-type Ca^{2+} channels may enhance the release of vasodilatory factors from endothelial cells. It has been reported that mibefradil, a T-type Ca^{2+} channel antagonist withdrawn from the market, potentiated endothelium-dependent relaxation in the rings of arteries from animals (*18*, *19*). This finding is similar to the present findings. Therefore, T-type Ca^{2+} channels may play an important role in the physiology of the vascular endothelium. In fact, T-type Ca^{2+} channels have been detected in endothelial cells (*13*–*15*). The ability of efonidipine to reduce levels of platelet and monocyte activation markers in hypertensive patients with diabetes (*20*) can be explained by its beneficial effects on endothelial function.

L-type voltage-dependent Ca^{2+} channels are widely distributed in heart muscle, smooth muscle, and skeletal muscle; these channels are known to be involved in the excitement-coupling contraction of muscles. In contrast, little is known about T-type Ca^{2+} channels, except that they are present in sinoatrial node cells and Purkinje cells, and they are involved in the pacemaker function of the myocardium (*21*, *22*). Recently, T-type Ca^{2+} channels were found in the vasculature (*13*–*15*, *23*, *24*). However, the physiological roles and the mechanisms of action of this channel in the vasculature have not been clarified. Therefore, in the present study, it remained difficult to speculate about the precise mechanism by which efonidipine becomes involved in the vasodilatory response of the brachial artery.

The second finding of note in the present study was that efonidipine, but not nifedipine, reduced the levels of urinary excretion of 8-OHdG and the serum concentration of malondialdehyde-modified LDL, both of which act as indexes of oxidative stress. The compound 8-OHdG is one of the most common markers used for evaluating oxidative DNA damage, and it is a product formed by the specific attack of a hydroxyl radical on DNA (*25*). Several studies have suggested that oxidative DNA damage is enhanced in some forms of hypertension (*26*). The concentration of malondialdehyde-modified LDL has been proposed as the biologic signature of clinical LDL oxidation and has been reported to be higher in patients with essential hypertension than in normal controls (*27*). Therefore, one possible mechanism to account for the ability of efonidipine to improve vascular endothelial dysfunction in patients with essential hypertension would be a reduction of oxidative stress, which can otherwise directly injure the endothelium. Several studies have demonstrated the free radical scavenging activity *in vitro* of certain Ca^{2+} channel blockers (*28–30*). The effects of efonidipine might be accounted for by a direct activity, other than Ca^{2+} channel blockade, on the endothelium.

In the present study, both efonidipine and nifedipine were shown to increase the baseline diameter of the brachial artery, possibly *via* an antagonism of the L-type Ca^{2+} channels of vascular smooth muscle cells, and both drugs attenuated the response of the brachial artery to exogenous NO from NTG. A potential factor that can confound the interpretation of the FMD is the baseline diameter (*31*). Namely, if the baseline diameter changes, the resulting percent change in diameter may be affected. An increase in baseline diameter by dihydropyridines may contribute to a decrease in response to another vasodilating factor. In the experimental setting, the decrease in FMD induced by nifedipine may have reflected a change in resting tone, and not a reduction of endothelial function. Therefore, in the present study, endothelial function was assessed according to the endothelial function index, as calculated by the ratio of the FMD to the response to NTG, because the effect of a vascular response to NO could be ruled out. It was thus possible to safely evaluate the effects of the two different Ca^{2+} channel blockers on endothelium function.

In conclusion, the present study is the first to demonstrate the specific effects of a T-type Ca^{2+} channel blockade on *in vivo* endothelial function. Efonidipine, a T-type and L-type Ca^{2+} channel blocker, was found capable of reversing endothelial dysfunction in patients with essential hypertension, whereas nifedipine, an L-type Ca^{2+} channel blocker, had no such ameliorative effect on endothelial function. This finding is expected to be useful for the selection of antihypertensive agents among dihydropyridines when hypertensive patients have a disturbance in endothelium-dependent vasodilation.

Acknowledgements

We thank Ms. Chizuko Suyama for her excellent technical assistance and Ms. Michiko Aoyama for her secretarial assistance.

References

1. Ross A: Atherosclerosis—an inflammatory disease. *N Engl J Med* 1999; **340**: 115–126.
2. Panza JA, Quyyumi AA, Brush JE Jr, Epstein SE: Abnormal endothelium-dependent vascular relaxation in patients with essential hypertension. *N Engl J Med* 1990; **323**: 22–27.
3. Moriguchi J, Itoh H, Harada S, *et al*: Low frequency regular exercise improves flow-mediated dilation of subjects with mild hypertension. *Hypertens Res* 2005; **28**: 315–321.
4. Higashi Y, Oshima T, Ozono R, Matsuura H, Kajiyama G: Aging and severity of hypertension attenuate endothelium-dependent renal vascular relaxation in humans. *Hypertension* 1997; **30**: 252–258.
5. Egashira K, Suzuki S, Hirooka Y, *et al*: Impaired endothelium-dependent vasodilation in large epicardial and resistance coronary arteries in patients with essential hypertension. *Hypertension* 1995; **25**: 201–206.
6. Tarutani Y, Matsumoto T, Takashima H, Yamane T, Horie M: Brachial artery flow-mediated vasodilation is correlated with coronary vasomotor and fibrinolytic response induced by bradykinin. *Hypertens Res* 2005; **28**: 59–66.
7. Staessen JA, Li Y, Thijs L, Wang JG: Blood pressure reduction and cardiovascular prevention: an update including the 2003–2004 secondary prevention trials. *Hypertens Res* 2005; **28**: 385–407.
8. Ghiadoni L, Magagna A, Versari D, *et al*: Different effect of antihypertensive drugs on conduit artery endothelial function. *Hypertension* 2003; **41**: 1281–1286.
9. Iwatsubo H, Nagano M, Sakai T, *et al*: Converting enzyme inhibitor improves forearm reactive hyperemia in essential hypertension. *Hypertension* 1997; **29**: 286–290.
10. Hirooka Y, Imaizumi T, Masaki H, *et al*: Captopril improves impaired endothelium-dependent vasodilation in hypertensive patients. *Hypertension* 1992; **20**: 175–180.
11. Millgard J, Hagg A, Sarabi M, Lind L: Captopril, but not nifedipine, improves endothelium-dependent vasodilation in hypertensive patients. *J Hum Hypertens* 1998; **12**: 511–516.
12. Taddei S, Virdis A, Ghiadoni L, *et al*: Restoration of nitric oxide availability after calcium antagonist treatment in essential hypertension. *Hypertension* 2001; **37**: 943–948.
13. Wu S, Haynes J, Taylor JT, *et al*: Cav 3.1 (α_1G) T-type Ca^{2+} channels mediate vaso-occlusion of sickled erythrocytes in lung microcirculation. *Circ Res* 2003; **93**: 346–353.
14. Yazawa K, Ono K, Iijima T: Modulation by mibefradil of the histamine-induced Ca^{2+} entry in human aoritic endothelial cells. *Jpn J Pharmacol* 2002; **90**: 125–130.
15. Nilius B, Prenen J, Kamouchi M, Viana F, Voets T, Droogmans G: Inhibition by mibefradil, a novel calcium antagonist, of Ca^{2+}- and volume-activated Cl^- channels in macrovascular endothelial cells. *Br J Pharmacol* 1997; **121**: 547–555.

16. Masumiya H, Shijuku T, Tanaka H, Shigenobu K: Infibition of myocardial L- and T-type Ca^{2+} currents by efonidipine: possible mechanism for its chronotropic effect. *Eur J Pharmacol* 1998; **349**: 351–357.
17. Oshima T, Ozono R, Yano Y, *et al*: Association of *Helicobacter pylori* infection with systemic inflammation and endothelial dysfunction in healthy male subjects. *J Am Coll Cardiol* 2005; **45**: 1219–1222.
18. Boulanger CM, Nakashima M, Olmos L, Joly G, Vanhoutte PM: Effects of the Ca^{2+} antagonist RO 40-5967 on endothelium-dependent responses of isolated arteries. *J Cardiovasc Pharmacol* 1994; **23**: 869–876.
19. Boulanger CM, Desta B, Clozel JP, Vanhoutte PM: Chronic treatment with the Ca^{2+} channel inhibitor RO 40-5967 potentiates endothelium-dependent relaxations in the aorta of the hypertensive salt sensitive Dahl rat. *Blood Press* 1994; **3**: 193–196.
20. Nomura S, Kanazawa S, Fukuhara S: Effects of efonidipine on platelet and monocyte activation markers in hypertensive patients with and without type 2 diabetes mellitus. *J Hum Hypertens* 2002; **16**: 539–547.
21. Hagiwara N, Irisawa H, Kameyama M: Contribution of two types of calcium currents to the pacemaker potentials of rabbit sinoatrial node cells. *J Physiol* 1988; **395**: 233–253.
22. Tseng GN, Boyden PA: Multiple types of Ca^{2+} currents in single canine Purkinje cells. *Circ Res* 1989; **65**: 1735–1750.
23. Chen C, Lamping KG, Nuno DW, *et al*: Abnormal coronary function in mice deficient in α_{1H} T-type Ca^{2+} channels. *Science* 2003; **302**: 1416–1418.
24. Vanbavel E, Sorop O, Andreasen D, Pfaffendorf M, Jensen BL: Role of T-type calcium channels in myogenic tone of skeletal muscle resistance arteries. *Am J Physiol* 2002; **283**: H2239–H2243.
25. Hornig B, Landmesser U, Kohler C: Comparative effect of ACE inhibition and angiotensin II type 1 receptor antagonism on the biovailability of nitric oxide in patients with coronary artery disease: role of superoxide dismutase. *Circulation* 2001; **103**: 799–805.
26. Higashi Y, Sasaki S, Nakagawa K, Matsuura H, Oshima T, Chayama K: Endothelial function and oxidative stress in renovascular hypertension. *N Engl J Med* 2002; **346**: 1954–1962.
27. Maggi E, Marchesi E, Ravette V, Martignoni A, Finardi G, Bellomo G: Presence of autoantibodies against oxidatively modified low-density lipoprotein in essential hypertension: a biochemical signature of an enhanced *in vivo* low-density lipoprotein oxidation. *J Hypertens* 1995; **13**: 129–138.
28. Mak TI, Boehme P, Weglicki WB: Antioxidant effects of calcium channel blockers against free radical injury in endothelial cells. *Circ Res* 1992; **70**: 1099–1103.
29. Umemoto S, Tanaka M, Kawahara S, *et al*: Calcium antagonist reduces oxidative stress by upregulating Cu/Zn superoxide dismutase in stroke-prone spontaneously hypertensive rats. *Hypertens Res* 2004; **27**: 877–885.
30. Yasunari K, Maeda K, Nakamura M, Watanabe T, Yoshikawa J: Benidipine, a long-acting calcium channel blocker, inhibits oxidative stress in polymorphonuclear cells in patients with essential hypertension. *Hypertens Res* 2005; **28**: 107–112.
31. Correti MC, Anderson TJ, Benjamin EJ, *et al*: Guidelines for the ultrasound assessment of endothelial-dependent flow-mediated vasodilation of the brachial artery. *J Am Coll Cardiol* 2002; **39**: 257–265.
