# Supplementary material for: Effects of T-Type Calcium Channel Blockers on Renal Function and Aldosterone in Patients with Hypertension: A Systematic Review and Meta-Analysis
Source: PLoS One. 2014 Oct 17;9(10):e109834. doi: 10.1371/journal.pone.0109834 (PMC4201480; doi:10.1371/journal.pone.0109834)
Supplement: File S3 — PDF files of twenty-four studies included in the meta-analysis. (ZIP) [file pone.0109834.s007.zip › Supporting information-PDF files/27. Intern Med 2004[43(7)]561-565.pdf]

□ ORIGINAL ARTICLE □

Effects of the Long-acting Calcium Channel Blockers, Amlodipine, Manidipine and Cilnidipine on Steroid Hormones and Insulin Resistance in Hypertensive Obese Patients

Hajime UESHIBA and Yukitaka MIYACHI

Abstract

Objective **To demonstrate that calcium channel blockers can improve insulin resistance clinically, we investigated the effects of the calcium channel blockers, amlodipine, manidipine and cilnidipine on serum levels of steroid hormones and insulin.**

Subjects and Methods **Thirty hypertensive obese patients [15 men and 15 women; mean age 55.9 years, mean body mass index (BMI) 27.6] were divided into three groups and treated with either 5 mg of amlodipine, 20 mg of manidipine or 10 mg of cilnidipine. Blood pressure (BP), fasting plasma glucose (FPG), HbA_{1c}, fasting serum immunoreactive insulin (F-IRI), insulin resistance index [as assessed by the homeostasis model assessment (HOMA-R)], serum DHEA, serum DHEA-S, plasma ACTH, serum cortisol, plasma renin activity (PRA), and serum aldosterone, were measured before and after 1, 2, 3 and 6 months of treatment.**

Results **In all three groups, BP decreased significantly after 1 month and F-IRI and HOMA-R decreased significantly after 2–3 months. A concurrent rise in serum DHEA and DHEA-S levels was also observed, however, the differences were not significant. No changes in FPG, HbA_{1c}, ACTH, cortisol, PRA or aldosterone levels were observed during treatment.**

Conclusions **We conclude that amlodipine, manidipine and cilnidipine all improve insulin resistance and consequently increase serum levels of DHEA and DHEA-S.**

(Internal Medicine 43: 561–565, 2004)

Key words: calcium channel blocker, steroid hormone, insulin resistance, hypertention, obesity

Introduction

Insulin resistance with hyperinsulinemia may place obese or hypertensive patients at an increased risk of developing atherosclerosis. Previous studies have reported an increased prevalence of insulin resistance amongst patients with essential hypertension (1–3). Therefore, whenever treating such patients, we should select antihypertensive agents that may have the added effect of improving insulin resistance. Dehydroepiandrosterone (DHEA), an adrenal androgen, has been shown to have anti-atherogenic, anti-obesity, anti-diabetic, anti-osteoporotic and anti-tumorogenic actions (4–8). The metabolism of DHEA and DHEA sulfate (DHEA-S) may be regulated by insulin (9). There is a negative correlation between serum insulin and DHEA-S levels (10, 11) and in experimentally induced acute hyperinsulinemia, serum DHEA and DHEA-S levels are reduced (10, 12). These findings have led to the hypothesis that hyperinsulinemia may promote atherosclerosis by decreasing serum DHEA and DHEA-S levels (13). Moreover, there is a close correlation between low levels of serum DHEA-S and coronary heart disease (14, 15).

Some patients with obesity or hypertension display impaired glucose tolerance with hyperinsulinemia following an oral glucose challenge. Such patients may require therapy for insulin resistance. Angiotensin-converting enzyme (ACE) inhibitors have beneficial effects on glucose, insulin and lipid metabolism, and treatment with captopril has shown an improvement in insulin sensitivity (16, 17). Several studies have reported that long-acting calcium channel blockers (CCBs), such as amlodipine and nitrendipine, can improve glucose tolerance and lower insulin levels (18, 19). Although one coronary heart disease prevention trial suggested that CCBs may increase mortality in patients with coronary heart disease (20), the results of the antihypertensive and lipid-

From the First Department of Internal Medicine, Toho University School of Medicine, Tokyo
Received for publication September 18, 2003; Accepted for publication February 25, 2004
Reprint requests should be addressed to Dr. Hajime Ueshiba, the First Department of Internal Medicine, Toho University School of Medicine, 6-11-1 Ohmori-Nishi, Ohta-ku, Tokyo 143-0015

Table 1. Summary of Patient Characteristics

	Amlodipine	Manidipine	Cilnidipine
Number	10	10	10
Sex (M/F)	5/5	5/5	5/5
Age (years)	57.4±6.6	55.3±9.2	56.9±9.5
BMI (kg/m^2)	27.7±2.0	27.6±1.9	27.8±1.5
SBP (mmHg)	166±5	166±13	169±5
DBP (mmHg)	96±7	102±6	98±8
FPG (mg/dl)	101±7	105±8	101±8
HbA_{1C} (%)	5.8±0.4	5.8±0.5	5.7±0.3
F-IRI (μU/ml)	11.0±1.1	13.3±3.3	15.2±3.8

Data are means±SD. There were no significant differences between the patients who were assigned to the amlodipine, manidipine and cilnidipine treatment groups.

lowering treatment to prevent heart attack trial (ALLHAT) did not support this finding (21). Long-acting CCBs will therefore likely continue to be used actively as antihypertensive agents in the future.

In this study, we investigated the effects of the CCBs, amlodipine, manidipine and cilnidipine on serum levels of steroid hormones and insulin in hypertensive patients who are obese.

Materials and Methods

Study design

A total of 30 hypertensive patients who were also obese (15 men and 15 women; mean age 55.9±4.8 years) were recruited from the endocrine clinics at the Toho University Hospital in Tokyo. Patients were eligible for the study if they met the following criteria: BMI >25.0; HbA_{1c} <6.5%; blood pressure >160/95 mmHg on at least two occasions during a 2-week observation period, when taking no antihypertensive medication; and no evidence of kidney, liver, endocrine, or other major organ system disease, as determined by medical history, physical examination, and routine laboratory tests. Patients were excluded if found to be taking any medication which was known to affect steroid hormone levels, during or prior to this study. Informed consent was obtained from all subjects.

Patients were randomly assigned to treatment with either 5 mg of amlodipine (n=10), 20 mg of manidipine (n=10) or 10 mg of cilnidipine (n=10). Each dose was constant during this study. Baseline characteristics of age, sex and BMI were similar between the three groups (Table 1). The following variables were measured prior to treatment and then after 1, 2, 3 and 6 months of drug therapy: BMI, blood pressure (BP), fasting plasma glucose (FPG), HbA_{1c}, fasting serum immunoreactive insulin (F-IRI), insulin resistance index [as assessed by the homeostasis model assessment (HOMA-R)], serum DHEA, serum DHEA-S, plasma ACTH, serum cortisol, plasma renin activity (PRA) and serum aldosterone.

Analytical methods

Plasma glucose levels were determined by the glucose oxidase method. HbA_{1c} was measured by high-performance liquid chromatography. Serum levels of insulin, DHEA, DHEA-S, ACTH, cortisol, PRA and aldosterone were determined by radioimmunoassay. The inter-assay coefficient of variation (CV) and the intra-assay CV were less than 7.5% for all assays.

Statistical analysis

Data are reported as the mean±SD. Pre-treatment and post-treatment variables were compared by repeated measure one-way ANOVA and Fisher's protected least significant difference. *P* values less than 0.05 were considered to be statistically significant.

Results

Amlodipine, manidipine and cilnidipine significantly reduced the mean systolic BP and mean diastolic BP after 1 month of treatment (Fig.1). There was no significant difference between the three groups in terms of antihypertensive effect. BMI did not change significantly during any of the treatments. The changes of mean F-IRI and HOMA-R are shown in Fig.2. After 2 months of treatment these were significantly reduced, and they continued to decrease in each group. However, the mean FPG and HbA_{1c} did not change significantly in any of the groups (Table 2). The changes in mean serum DHEA and DHEA-S levels are shown in Fig.3. Concurrent with the fall in F-IRI and HOMA-R, amlodipine, manidipine and cilnidipine increased serum DHEA and DHEA-S levels after 2 or 3 months. There was a negative corelation between the changes of F-IRI or HOMA-R and those of DHEA or DHEA-S (F-IRI and DHEA: r=–0.997, p=0.0004, F-IRI and DHEA-S: r=–0.943, p=0.0371, HOMA-R and DHEA: r=–0.953, p=0.0278, HOMA-R and DHEA-S: r=–0.994, p=0.0012). There were no significant differences in ACTH, cortisol, PRA or aldosterone concentrations for any of the treatments (Table 2).

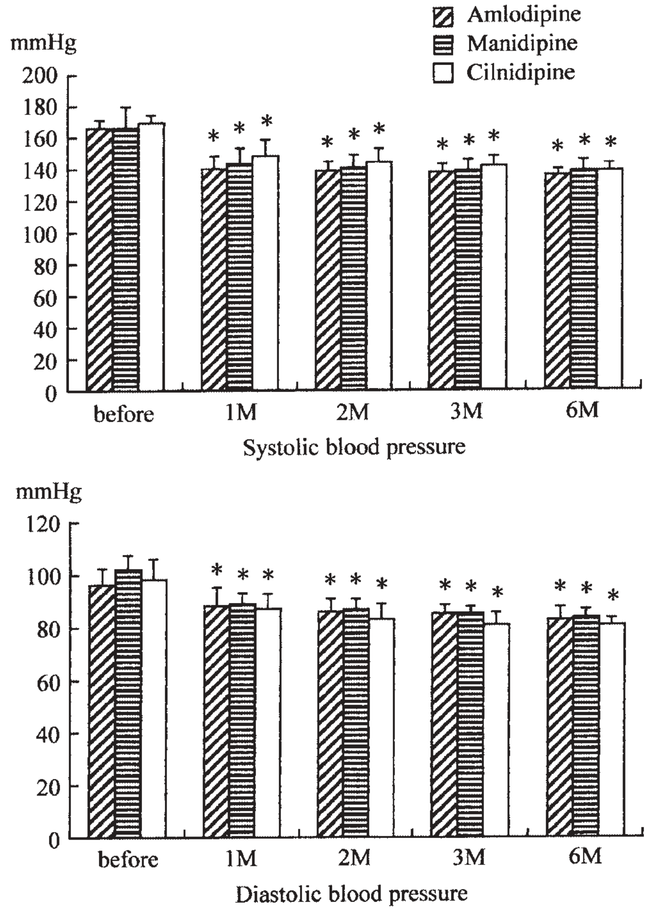

Figure 1. Effects of antihypertensive therapy. *p<0.05 vs before therapy.

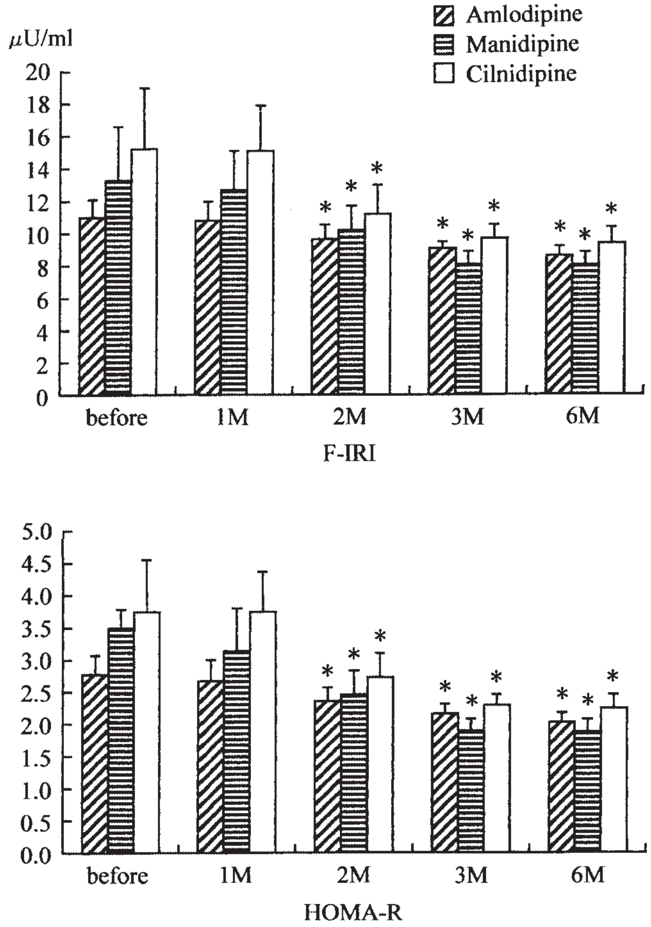

Figure 2. Changes in F-IRI and HOMA-R. *p<0.05 vs before therapy.

Table 2. Changes in FPG, HbA_{1c}, Plasma ACTH, Serum Cortisol, PRA and Serum Aldosterone

		before	6 months
FPG (mg/dl)	Amlodipine	101±7	99±6
	Manidipine	105±8	103±7
	Cilnidipine	101±8	98±5
HbA_{1c} (%)	Amlodipine	5.8±0.4	5.7±0.2
	Manidipine	5.8±0.5	5.7±0.2
	Cilnidipine	5.7±0.3	5.8±0.2
ACTH (pg/ml)	Amlodipine	26±6	29±6
	Manidipine	27±11	27±5
	Cilnidipine	30±7	30±4
cortisol (μg/dl)	Amlodipine	9.7±1.1	9.9±0.5
	Manidipine	10.5±1.9	9.8±0.9
	Cilnidipine	10.2±1.3	9.4±0.6
PRA (ng/ml/h)	Amlodipine	0.93±0.29	0.98±0.15
	Manidipine	1.20±0.41	0.92±0.18
	Cilnidipine	0.88±0.18	0.95±0.19
aldosterone (pg/ml)	Amlodipine	85.0±15.5	95.5±10.7
	Manidipine	94.2±12.0	88.5±9.3
	Cilnidipine	84.8±9.4	94.4±8.9

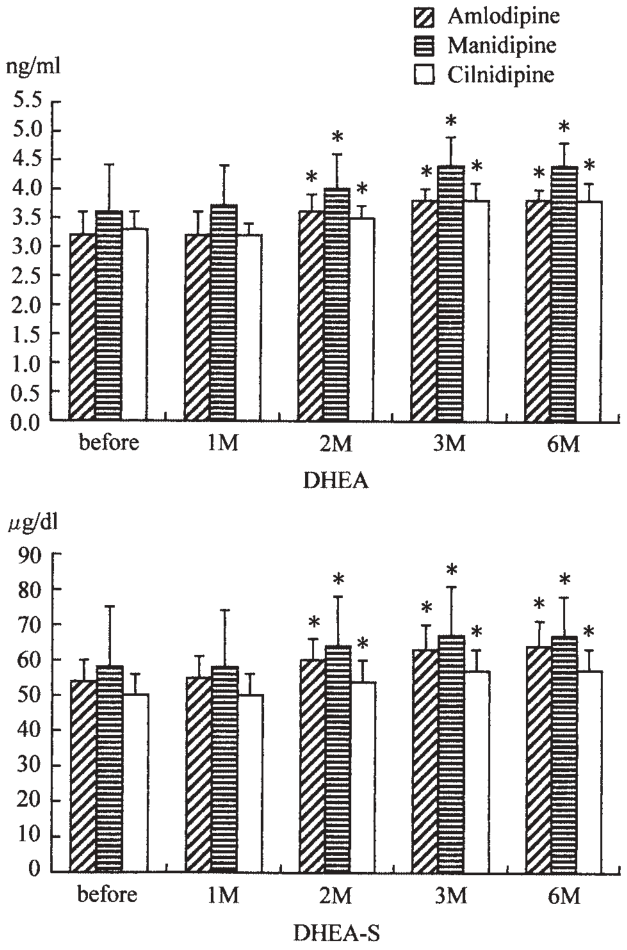

Figure 3. Changes in serum DHEA and DHEA-S. *p<0.05 vs before therapy.

Discussion

From our observation period of 6 months, we demonstrated that the long-acting CCBs, amlodipine, manidipine and cilnidipine, can lower blood pressure and improve insulin resistance in hypertensive patients who are obese. The increase in serum DHEA and DHEA-S levels seems to reflect the concurrent reduction in serum insulin. Our results are consistent with previous reports (10, 11), which serum insulin levels were negatively correlated with DHEA-S levels. However, it seems that amlodipine, manidipine and cilnidipine do not clinically affect the ACTH-cortisol system and the renin-aldosterone system and these drugs appear neutral with respect to their action on plasma glucose levels, with the mean FPG and HbA_{1c} not changing significantly in any of the groups.

Several studies report the effects of antihypertensive agents on insulin resistance. Both α-adrenergic blockers and ACE inhibitors have been shown to improve insulin sensitivity (22, 23). The CCB nifedipine can worsen insulin sensitivity (24), inhibit insulin release (25) and impair glucose tolerance (26). It has been speculated that an acute antihypertensive effect may activate the sympathetic nervous system and aggravate insulin resistance. In contrast, nitrendipine, a long-acting CCB that gradually lowers blood pressure, can improve hyperinsulinemia, glucose tolerance and insulin resistance (19). This drug can also increase serum levels of DHEA-S. As our study had a long observation period of 6 months with follow-up in an outpatient clinic setting, the assessment of insulin resistance was performed by measuring fasting serum immunoreactive insulin and HOMA-R. Two mechanisms have been proposed for the reduction of insulin resistance that is seen with CCBs (27, 28). First, these drugs produce vasodilation and increase blood flow to skeletal muscle, which may in turn lead to increased delivery of insulin and glucose and enhance the non-oxidative pathways of glucose utilization (27). Secondly, CCBs also improve insulin sensitivity at the cellular level by decreasing the cytosolic free calcium concentration (28).

The physiological effects of decreased serum levels of DHEA and DHEA-S are not well understood. It has been suggested that in the context of severe chronic illness, there is a shift in adrenal steroid synthesis, which may be necessary for survival (29, 30). Cortisol levels are elevated and those of DHEA and DHEA-S are reduced. However, during the prelude to the severe phase of an illness, DHEA and DHEA-S levels are decreased without a corresponding increase in cortisol levels. As the severe state progresses and stress increases, cortisol levels become elevated. The potential harmful effects of reduced levels of DHEA and DHEA-S can be addressed by reducing the degree of insulin resistance, which will lead to a subsequent increase in DHEA and DHEA-S levels. In conclusion, our results suggest that in hypertensive patients who are obese, in addition to lowering blood pressure, amlodipine, manidipine and cilnidipine decrease serum insulin levels and increase serum DHEA and DHEA-S levels.

This work was presented in part at the 26th International Congress of Internal Medicine, Kyoto, 2002.

References

1) Modan M, Halkin H, Almog S, et al. Hyperinsulinemia: a link between hypertension, obesity and glucose intolerance. J Clin Invest **75**: 809–817, 1985.
2) Ferrannini E, Buzzigoli G, Bonadonna R, et al. Insulin resistance in essential hypertension. N Engl J Med **317**: 350–357, 1987.
3) Shen D-C, Shei S-M, Fuh M, Wu DA, Chen YD, Reaven GM. Resistance to insulin-stimulated glucose uptake in patients with hypertension. J Clin Endocrinol Metab **66**: 580–583, 1988.
4) Gordon GB, Bush DE, Weisman HF. Reduction of atherosclerosis by administration of dehydroepiandrosterone. A study in the hypercholesterolemic New Zealand white rabbit with aortic intimal injury. J Clin Invest **82**: 712–720, 1998.
5) Nestler JE, Barlascini CO, Clore JN, Blackard WG. Dehydroepiandrosterone reduces serum low density lipoprotein levels and body fat but does not alter insulin sensitivity in normal men. J Clin Endocrinol

Metab **66**: 57–61, 1988.
6) Coleman DL, Leifer EH, Schwizer RW. Therapeutic effects of dehydroepiandrosterone (DHEA) in diabetic mice. Diabetes **31**: 830–833, 1982.
7) Turner RT, Lifrak ET, Beckner M, Wakley GK, Hannon KS, Parker LN. Dehydroepiandrosterone reduces cancellous bone osteopenia in ovariectomized rats. Am J Physiol **258**: E673–677, 1990.
8) Ciolino HP, Yeh GC. The steroid hormone dehydroepiandrosterone inhibits CYP1A1 expression in vitro by a post-transcriptional mechanism. J Biol Chem **274**: 35186–35190, 1999.
9) Nestler JE, Strauss JF III. Insulin as an effector of human ovarian and adrenal steroid metabolism. Endocrinol Metab Clin North Am **20**: 807–823, 1991.
10) Smith S, Ravnikar VA, Barbieri RL. Androgen and insulin response to an oral glucose challenge in hyperandrogenic women. Fertil Steril **48**: 72–77, 1987.
11) Schriock ED, Buffington CK, Hubert GD. Divergent correlation of circulatory dehydroepiandrosterone sulfate and testosterone with insulin levels and insulin receptor binding. J Clin Endocrinol Metab **66**: 1329–1331, 1988.
12) Falcone T, Finegood DT, Fantus IG, Morris D. Androgen response to endogenous insulin secretion during the frequently sampled intravenous glucose tolerance test in normal and hyperandrogenic women. J Clin Endocrinol Metab **71**: 1653–1657, 1990.
13) Nestler JE, Clore JN, Blackard WG. Dehydroepiandrosterone: the “missing link” between hyperinsulinemia and atherosclerosis? FASEB J **6**: 3073–3075, 1992.
14) Herrington DM, Gordon GB, Achuff SC, et al. Plasma dehydroepiandrosterone and dehydroepiandrosterone sulfate in patients undergoing diagnostic coronary angiography. J Am Coll Cardiol **16**: 862–870, 1990.
15) Mitchell LE, Sprecher DL, Borecki IB, Rice T, Laskarzewski PM, Rao DC. Evidence for an association between dehydroepiandrosterone sulfate and nonfatal, premature myocardial infarction in males. Circulation **89**: 89–93, 1994.
16) Pollare T, Lithell H, Berne C. A comparison of the effects of hydrochlorothiazide and captopril on glucose and lipid metabolism in patients with hypertension. N Engl J Med **321**: 868–873, 1989.
17) Borntorp K, Lindgarde F, Mattiasson I. Long-term effects on insulin sensitivity and sodium transport in glucose-intolerant hypertensive subjects when beta-blockade is replaced by Captopril treatment. J Hum Hypertens **6**: 291–298, 1992.
18) Beer NA, Jakubowicz DJ, Beer RM, Nestler JE. The calcium channel blocker amlodipine raises serum dehydroepiandrosterone sulfate and androstenedione, but lowers serum cortisol, in insulin-resistant obese and hypertensive men. J Clin Endocrinol Metab **76**: 1464–1469, 1993.
19) Beer NA, Jakubowicz DJ, Beer RM, Arocha IR, Nestler JE. Effects of nitrendipine on glucose tolerance and serum insulin and dehydroepiandrosterone sulfate levels in insulin-resistant obese and hypertensive men. J Clin Endocrinol Metab **76**: 178–183, 1993.
20) Furberg CD, Psaty BM, Meyer JV. Nifedipine: dose-related increase in mortality in patients with coronary heart disease. Circulation **92**: 1326–1331, 1995.
21) The ALLHAT Officers and Coordinators for the ALLHAT Collaborative Research Group. Major outcomes in high-risk hypertensive patients randomized to angiotensin-converting enzyme inhibitor or calcium channel blocker vs diuretic. The antihypertensive and lipid-lowering treatment to prevent heart attack trial (ALLHAT). JAMA **288**: 2981–2997, 2002.
22) Pollare T, Lithell HL, Selinus I, Berne C. Application of prazosin is associated with an increase of insulin sensitivity in obese patients with hypertension. Diabetologia **31**: 415–420, 1988.
23) Torlone E, Britta M, Rambotti AM, et al. Improved insulin action and glycemic control after long-term angiotensin-converting enzyme inhibition in subjects with arterial hypertension and type II diabetes. Diabetes Care **16**: 1347–1355, 1993.
24) Lind L, Berne C, Pollare T, Lithell H. Metabolic effects of antihypertensive treatment with nifedipine or furosemide: a double-blind, cross-over study. J Hum Hypertens **9**: 137–141, 1995.
25) Charles S, Ketelslegers JM, Buysschaert M, Lambert AE. Hyperglycemic effects of nifedipine. Br Med J **283**: 19–20, 1981.
26) Vessby B, Abelin J, Finnson M. Effects of nifedipine treatment on carbohydrate and lipoprotein metabolism. Curr Ther Res **33**: 1075–1081, 1983.
27) Baron AD, Brechtel GB, Wallace P, Edelman SV. Rates and tissue sites of non-insulin and insulin-mediated glucose uptake in humans. Am J Physiol **255**: E769–E774, 1988.
28) Draznin B, Sussman KE, Eckel RH, Kao M, Yost T, Sherman MA. Possible role of cytosolic free calcium concentration in mediating insulin resistance of obesity and hyperinsulinemia. J Clin Invest **82**: 1848–1852, 1988.
29) Parker LN, Levin ER, Lifrak ET. Evidence for adrenocortical adaptation to severe illness. J Clin Endocrinol Metab **60**: 947–952, 1985.
30) Wade CE, Lindberg JS, Cockrell JL, et al. Upon-admission adrenal steroidogenesis is adapted to the degree of illness in intensive care unit patients. J Clin Endocrinol Metab **67**: 223–227, 1988.
