# Supplementary material for: Effects of T-Type Calcium Channel Blockers on Renal Function and Aldosterone in Patients with Hypertension: A Systematic Review and Meta-Analysis
Source: PLoS One. 2014 Oct 17;9(10):e109834. doi: 10.1371/journal.pone.0109834 (PMC4201480; doi:10.1371/journal.pone.0109834)
Supplement: File S3 — PDF files of twenty-four studies included in the meta-analysis. (ZIP) [file pone.0109834.s007.zip › Supporting information-PDF files/28. Ren Fail 2003[25(5)]681-689.pdf]

MARCEL DEKKER, INC. • 270 MADISON AVENUE • NEW YORK, NY 10016

©2003 Marcel Dekker, Inc. All rights reserved. This material may not be used or reproduced in any form without the express written permission of Marcel Dekker, Inc.

RENAL FAILURE
Vol. 25, No. 5, pp. 681–689, 2003

CLINICAL STUDY

Effects of Manidipine and Nifedipine on Blood Pressure and Renal Function in Patients with Chronic Renal Failure: A Multicenter Randomized Controlled Trial

Guido Bellinghieri,[1] Giampiero Mazzaglia,[2] Vincenzo Savica,[1] and Domenico Santoro[1],*

[1]Chairs and Division of Nephrology, School of Medicine, University of Messina Viale Gazzi, Messina, Italy
[2]The Health Search Database, Florence, Italy

ABSTRACT

Several studies suggest the distinctive advantages of ACE-inhibitors and calcium-channel blockers in protecting the residual renal function in hypertensive patients. Pre-clinical and clinical studies have shown rare adverse events in the treatment with manidipine, which is commonly used as antihypertensive drug. We therefore decided to compare the effects of manidipine and nifedipine, on blood pressure, and renal function. One hundred and one hypertensive patients with chronic renal failure were randomly assigned to receive either manidipine 20 mg daily or nifedipine 60 mg daily, respectively. Patients were assessed every two weeks during the active treatment period with the final follow-up after three months. The primary endpoint was the achievement of DBP $\leq$90 mmHg or a 10 mmHg DBP reduction from the baseline values, whilst the secondary endpoints was the improvement of the renal function assessed through the

*Correspondence: Domenico Santoro, M.D., Chairs and Division of Nephrology, School of Medicine, University of Messina Viale Gazzi, C. da Conte Coop. Sperone is. 3 pal. E, 98168 Messina, Italy; Fax: 090/5731904; E-mail: santisi@hotmail.com.

DOI: 10.1081/JDI-120024284
0886-022X (Print); 1525-6049 (Online)
www.dekker.com
Copyright © 2003 by Marcel Dekker, Inc.

©2003 Marcel Dekker, Inc. All rights reserved. This material may not be used or reproduced in any form without the express written permission of Marcel Dekker, Inc.

creatinine clearance, creatinine blood levels, protein and sodium urine excretion. Significant reduction in SBP ($p < 0.001$) and DBP ($p < 0.001$), compared to the baseline values, was reached in both treatments. Creatinine blood levels ($p < 0.05$) and creatinine clearance ($p < 0.01$) significantly increased in the manidipine group. Protenuria did not significantly change in the manidipine group but increased in the nifedipine group ($p < 0.05$). The number of patients with severe adverse reactions differed significantly ($p < 0.01$) between the groups with the highest frequency for nifedipine (14.5%) compared to manidipine (8.5%). The withdrawal rate was not significantly different between the groups. Manidipine is equally safe and effective as nifedipine and it may have more activity on renal function and less severe side effects compared to nifedipine.

Key Words: Hypertension; Kidney failure; Manidipine; Nifedipine; Randomized controlled trial; Side effects.

INTRODUCTION

Hypertension is highly prevalent in all developed countries and tends to increase with age.[1] Reports of the Working Group on Primary Prevention of Hypertension indicates a linear increase of hypertension among US general population from 4% in young adults to 65% in those patients aged over 80 years.[2]

The goal of treating patients with hypertension is to prevent the morbidity and mortality associated with high blood pressure by the least intrusive means possible. This should be accomplished by achieving and maintaining systolic blood pressure (SBP) below 140 mmHg and diastolic blood pressure (DBP) below 90 mmHg, while concurrently controlling other modifiable risk factors.[3] Hypertension is the most common underlying disease associated with different renal diseases and several studies reported a prevalence of 60% in glomerular disease, 33% in chronic pyelonephritis, and 75% when end stage renal failure is reached.[4]

The advent of antihypertensive therapy has improved both cardiovascular and renal prognosis of hypertensive patients and clinical trials have also demonstrates that lowering blood pressure preserves renal function.[5] The US National Institutes of Health consensus panel recommended four different therapeutic groups as effective, first-line, single agent therapy of hypertension: diuretics, β-blockers, ACE-inhibitors, and calcium-channel blockers (CCB).[6] Nevertheless, accumulating evidence suggests that ACE-inhibitors and CCB might be more renoprotective compared with other classes of antihypertensive agents.[7] Moreover, several studies indicate their distinctive advantages in protecting the residual renal function.[8]

Manidipine is a new CCB selected from 4-aryl-1, 4-dihydropyridine derivatives with a piperazine-alkyl side chain. Pre-clinical and clinical studies have shown an important antihypertensive action and the presence of severe adverse effects reported with other dihydropyridine CCB is rare with manidipine.[9]

At present, very few controlled trials have been carried out in order to evaluate the efficacy of manidipine in the treatment of hypertension in patients with renal failure. In the present study we have therefore compared the effects of manidipine and nifedipine, on blood pressure, renal function, and adverse effects.

MARCEL DEKKER, INC. • 270 MADISON AVENUE • NEW YORK, NY 10016

©2003 Marcel Dekker, Inc. All rights reserved. This material may not be used or reproduced in any form without the express written permission of Marcel Dekker, Inc.

PATIENTS AND METHODS

Patients' Selection

Hypertensive patients (diastolic blood pressure (DBP) $\geq$95 to $\leq$115 mmHg; systolic blood pressure (SBP) $\geq$160 to $\leq$210) aged 20–60 years with chronic renal failure were selected from 12 Italian Hospitals. Ethical committee approval was obtained at each institutional review board involved into the study and all the patients gave the written informed consent. The cause of renal disease in each patient was assessed by the history, physical examination, urine analysis, biochemical tests, radiological examinations, and by renal biopsies.

Creatinine levels $\geq$1.5 to $\leq$5.0 mg/dL kept stable for at least 6 month were also required as inclusion criteria. Subjects with nephrotic syndrome (proteinuria >5.0 gr/die), lupus nephritis, systemic vasculitis, and cryoglobulinemic vasculitis and those patients under treatment either with CCB or with other drugs that changed the creatinine metabolism, were excluded from the study.

Study Outline

After a run-in period of 2 weeks on placebo, with a wash-out from any previous antihypertensive drugs, eligible patients were randomly assigned to receive manidipine 10 mg daily and, nifedipine 30 mg daily, during the first two weeks. All tablets were dispensed every two weeks by the physician who checked patient compliance by the tablet counting. After two weeks, if a reduction of DBP of < 90 mmHg was not obtained, doses were increased to 20 mg daily in manidipine group and 60 mg daily in nifedipine group. The visits were carried out every two weeks during the active treatment period (visit 1–6) with the final follow-up after three months (visit 7). During each visit, blood pressure, hearth rate, weight, and drug compliance were checked. All patients were instructed to reduce their daily sodium intake to 100–120 mEq/die, their protein intake to 0.6 gr/kg/die with a total intake of 35 Kcal/kg/die. Evaluating 24 h urinary urea, phosphorus, and sodium excretion assessed dietary compliance during the study.

The primary outcome measure (endpoint) of the study was the achievement of DBP $\leq$90 mmHg or a 10 mmHg DBP reduction from the baseline values, whilst the secondary endpoints was the improvement of the renal function assessed through the creatinine clearance, creatinine blood levels, protein and sodium urine excretion. All changes in DBP and renal function were the differences between average values at follow-up (visit 7) and baseline (visit 2).

Clinical Evaluation

Blood pressure was assessed with a standard mercury sphygmomanometer by a blind observer with patients in the sitting position after a rest period of at least 10 min. SBP was measured as the point appearance (phase I) of Korotkoff sounds whilst DBP was measured as the point of disappearance (phase V).

MARCEL DEKKER, INC. • 270 MADISON AVENUE • NEW YORK, NY 10016

©2003 Marcel Dekker, Inc. All rights reserved. This material may not be used or reproduced in any form without the express written permission of Marcel Dekker, Inc.

Blood samples were taken before each visit in the morning after an overnight fast for measurement of complete blood count, blood chemistry including creatinine, blood urea nitrogen, uric acid, sodium, calcium, phosphorus, potassium, bilirubin, glucose, total cholesterol, HDL cholesterol, triglyceride, glutamic oxalacetic transaminase (GOT), glutamic-pyruvic transaminase (GPT), lactic dehydrogenase (LDH), and total proteins. A routine urinalysis was done and 24 h urines were collected for the measurement of sodium, potassium, phosphorus, proteinuria, nitrogen, glucose, and creatinine. Glomerular filtration rate was measured both with 24 h estimated creatinine clearance and Cockcroft and Gault method.[10]

The adverse effects were assessed by a physician during each visit. The severity of symptoms was recorded and the relationship to the drug was standardized as certain, probable, possible, unknown and uncertain.

STATISTICAL ANALYSIS

Results are expressed as percentage or means $\pm$ SD. Percentages have been compared by using the chi-square test. Means have been compared by Student's *t*-test when analysis of variance (ANOVA) had shown a significant difference between the groups. The intention to treat analysis included all patients who entered the study. The difference for a probability of 0.05 and a power of 0.80 was also calculated for each test and was considered the smallest difference between treatments likely to be detected as significant. A difference was considered as significant if *p*-value was less than 0.05.

RESULTS

Baseline Assessment

One hundred and one patients were recruited and included in the initial washout/run-in period. At the end of the two weeks of placebo run-in, 98 patients met the inclusion criteria and were randomized to active receive treatment.

Sixteen patients in the manidipine group and fifteen in the nifedipine group were withdrawn for several reasons such as adverse events, insufficient response, and lost to follow-up. Consequently, 32 patients in the manidipine group and 35 in the nifedipine group completed the study.

The main demographic and clinical characteristics of the study population according to treatment received are reported in Table 1. At baseline there were no significant differences between the two groups in age, sex, body weight, height, smoking, or drinking status. The distribution of renal function impairment also showed no significant differences.

Clinical Evaluation at Follow-Up

The main values related to the four BP parameters measured are shown in Table 2. In the population both manidipine and nifedipine induced a clinical and statistical

MARCEL DEKKER, INC. • 270 MADISON AVENUE • NEW YORK, NY 10016
©2003 Marcel Dekker, Inc. All rights reserved. This material may not be used or reproduced in any form without the express written permission of Marcel Dekker, Inc.

Table 1. Initial characteristics of subjects.

	Manidipine	Nifedipine
No. of patients	50	48
Gender (males/females)	37/22	38/18
Age (years)	50.7 ± 11.9	51.3 ± 10.9
Body weight (kg)[a]	70.2 ± 10.9	67.0 ± 11.1
Laboratory measurements (Mean ± SD)		
SBP (mm/Hg)	162.3 ± 15.4	163.5 ± 15.7
BP (mm/Hg)	97.2 ± 7.5	98.1 ± 9.5
Heart rate (beats/min)	76.5 ± 11.1	75.4 ± 9.1
Creatinine blood levels (mg/dL)	2.3 ± 1.0	2.5 ± 1.1
Creatinine clearance (mL/min)	38.4 ± 19.5	37.6 ± 16.4
Proteinuria (mg/24 h)[a]	1195.1 ± 1364.5	912.4 ± 981.4
Causes of renal impairment[b]		
Chronic glomerulonephritis	31.3 (19.0–46.4)	18.0 (9.0–31.9)
Hypertensive nephropathy	25.0 (14.1–39.8)	28.0 (16.6–47.2)
Diabetic nephropathy	10.4 (3.8–23.4)	10.0 (3.7–22.5)
Reflux nephropathy	8.3 (2.7–20.8)	2.0 (0.1–12.0)
Tubulo-interstitial nephropathy	6.3 (1.6–18.2)	6.0 (1.5–17.5)
Polycystic kidney	2.0 (0.1–12.4)	12.0 (4.9–25.0)
Other	16.7 (7.9–30.7)	24.0 (13.5–38.4)

SBP: systolic blood pressure; DBP: diastolic blood pressure.
[a]$P < 0.05$; a significant difference between the groups.
[b]Percentages and 95% confidence intervals.

Table 2. Effects of treatments with manidipine and nifedipine in reducing blood pressure.

Blood pressure	Manidipine			Nifedipine		
	Baseline	End of follow-up	Mean change (95% CI)	Baseline	End of follow-up	Mean change (95% CI)
Sitting						
DBP (mm/Hg)	99.8 ± 4.7	85.2 ± 9.1	14.5 (11.7−7.4)	102.2 ± 6.2	84.8 ± 6.4	17.4 (15.0−19.8)
SBP (mm/Hg)	166.7 ± 47.5	147.8 ± 15.2	19.0 (13.6−24.4)	169.3 ± 16.2	143.8 ± 9.9	25.5 (20.8−30.2)
Standing						
DBP (mm/Hg)	100.2 ± 5.2	86.4 ± 9.1	13.8 (10.9−16.6)	102.3 ± 6.9	85.5 ± 6.4	16.8 (14.2−19.3)
SBP (mm/Hg)	166.3 ± 13.3	146.5 ± 15.8	19.8 (14.5−25.2)	166.2 ± 17.6	142.2 ± 11.4	24.0 (18.9−29.2)

highly significant mean reduction at follow-up in SBP ($p < 0.001$) and DBP ($p < 0.001$), compared to the baseline values. On the 48 patients in the manidipine group, 42 patients were normalized (87.5%), none were responders, and 6 (12.5%) did not respond. The corresponding numbers in the nifedipine group were 44 (88%), 2 (4.0%), and 4 (8.0%).

The effects of both treatment groups on renal function and on laboratory values are reported in Tables 3 and 4. Significant changes from the baseline values were reported in the manidipine group for the creatinine blood levels ($P < 0.001$) and the

©2003 Marcel Dekker, Inc. All rights reserved. This material may not be used or reproduced in any form without the express written permission of Marcel Dekker, Inc.

Table 3. Effects of treatments on renal functions.[a]

	Manidipine		Nifedipine	
	Visit 2	Visit 7	Visit 2	Visit 7
Creatinine blood levels (mg/dL)	2.3 ± 1.0	2.1 ± 0.9[b]	2.5 ± 1.1	2.5 ± 1.4
Creatinine clearance (mL/min)	38.4 ± 19.5	44.8 ± 21.3[c]	37.6 ± 16.4	38.2 ± 19.3
Proteinuria (mg/24 h)	1195.1 ± 1364.5	890.3 ± 1156.2	912.4 ± 981.4	1234.3 ± 2101.9[b]
Sodium (mEq/24 h)	122.7 ± 50.9	125.3 ± 47.8	139.0 ± 122.6	133.7 ± 71.0
Potassium (mEq/24 h)	48.1 ± 18.7	50.3 ± 17.7	50.2 ± 45.9	42.8 ± 18.8

[a]Analysis within treatment on the differences compared to basal values (Visit 7/Visit 2); significant changes from baseline values.
[b]$p < 0.05$.
[c]$p < 0.001$.

Table 4. Mean laboratory measurements for patients at baseline and after treatments.

	Manidipine		Nifedipine	
	Visit 2	Visit 7	Visit 2	Visit 7
Glucose (mg/dL)	96.6	94.2	92.8	93.6
Sodium (mEq/L)	141.5	141.7	140.8	141.5
Potassium (mEq/L)	4.7	4.7	4.7	4.7
Calcium (mg/dL)	9.3	9.4	9.3	9.6
Phosphorus (mg/dL)	3.8	4.1	3.9	4.0
Total bilirubin (mg/dL)	0.6	0.6	0.6	0.6
Total cholesterol (mg/dL)	216.1	220.7	221.4	229.4
HDL-cholesterol (mg/dL)	54.3	54.2	53.2	52.4
Triglyceride (mg/dL)	149.6	125.3	164.8	169.4
SGOT (U/L)	19.9	18.9	18.6	20.1
SGPT (U/L)	20.7	19.1	19.2	20.1
LDH (U/L)	279.3	248.6	294.7	276.1

creatinine clearance ($P < 0.05$). In the nifedipine group treatments significantly increased at the end of follow-up the proteinuria ($P < 0.001$).

Overall, 24 patients during treatment reported 32 adverse events. Thirteen adverse events were reported by 11 patients (22.9%) treated with manidipine and 19 events were reported by 13 patients (26%) treated with nifedipine. Six patients (12.5%) discontinued manidipine and seven (14%) discontinued nifedipine because of adverse reactions.

DISCUSSION

The results of this study demonstrate that both manidipine at the daily dose of 10–20 mg orally and nifedipine at the daily dose of 30–60 mg significantly reduced in

©2003 Marcel Dekker, Inc. All rights reserved. This material may not be used or reproduced in any form without the express written permission of Marcel Dekker, Inc.

blood pressure in patients with mild to moderate hypertension and moderate chronic renal function impairment (CRF) due to either primary or secondary nephropathy.

The difference in the mean reduction in sitting DBP in both groups was 1.2 mm/Hg (95% CI: −2.0–4.4), which is low enough to conclude that the therapeutic efficacy of manidipine may be considered equivalent to nifedipine in this patient population.

Such results are consistent with other studies demonstrating that patients treated with CCB discontinue the initial therapy less than the other antihypertensive drugs.[11,12] Although we are not able to evaluate the long-term antihypertensive effects, after visit 2 the hypertension decreased and blood pressure was kept stable by both drugs throughout the duration of the study. Moreover, data indicates a comparable and significant efficacy of manidipine and nifedipine in lowering mild to moderate hypertension within the treatment groups.

It has been shown that sympathetic activation occurred during chronic treatment with amlodipine and felodipine, whereas manidipine and lacidipine did not showed the same effect on plasma noradrenaline. According to the authors, the difference may be related to different pharmacological characteristics of the last two drugs.[13]

However, the use of older dihydropyridines is associated with such well-known problems as negative inotropic activity, a rapid onset and short duration of action, reflex tachycardia, headache and ankle edema. The newer dihydropyridines, such as manidipine, lead to less negative inotropic effects due to the ‘‘vascular selectivity’’ that provides to exert a stronger action on the entire vascular system than on myocardial and nodal structure. Moreover, their onset of action is slow and for this reason little or no severe reflex tachycardia is triggered.[14]

These hypotheses have been confirmed by this study where the incidence of adverse events was similar in both groups, whereas there were favorable differences in the manidipine group regarding severity and specificity. Besides a high rate of ankle edema, patients treated with nifedipine have shown more severe adverse events compared to manidipine group in which we have observed a higher frequency of mild to moderate palpitations or tachycardia.

In presence of norepinephrine, CCB markedly increase glomerular filtration rate (GFR), through a preferential vasodilatation of preglomerular vessels, inducing only a modest improvement in the renal plasma flow.[15] Moreover, recent studies conducted in spontaneously hypertensive rats (SHR), demonstrated that manidipine acts both on afferent and efferent glomerular arterioles.[16] This mechanism constitutes a means of lowering systemic blood pressure while preserving renal perfusion. An additional renal protective mechanism could be related to the prospect that CCB may attenuate mesangial entrapment of macromolecules, thus reducing the stimulation of local inflammation and expansion of mesangial cells with subsequent progression to glomerular sclerosis.[17]

We observed a significant increase in creatinine clearance and reduction in creatinine blood levels in manidipine group, which suggest its better efficacy in improving the renal function compared to nifedipine. Proteinuria showed a mild increase in nifedipine group ($p < 0.05$) whilst it decreased in manidipine group. The different effect of manidipine on proteinuria may be related to his action on mesangial cells. Indeed treatment with manidipine in SHR showed reduction of mesangial cell proliferation and improvement in glomerular morphology.[16]

©2003 Marcel Dekker, Inc. All rights reserved. This material may not be used or reproduced in any form without the express written permission of Marcel Dekker, Inc.

Although the data generated has the limitations imposed by the study design, such as the short-term follow-up (six months), our study represents one of the first clinical experiences, over a large amount of patients, in which efficacy and safety of manidipine have been evaluated. Although long-term trials are necessary, in order to evaluate time-related effects, this study has demonstrated a comparable efficacy of manidipine and nifedipine for treating mild to moderate hypertension in patients with renal disease.

INVESTIGATORS AND INVOLVED CENTERS

Prof. V. E. Andreucci, Chair of Nephrology, Federico II University of Naples; Prof. G. Bellinghieri, Chair and Division of Nephrology, University of Messina; Dr. A. M. Bernardi, Division of Nephrology, Civil Hospital, Rovigo; Prof. R. Fogari, Chair of Internal Medicine, University of Pavia; Prof. G. Fuiano, Division of Nephrology, Mater Domini Hospital, Catanzaro; Dr. W. De Simone, Division of Nephrology and Dialysis, G. Crisuoli Hospital, S Angelo de Lombardi (AV); Dr. M. Giattanasio, Division of Nephrology and Dialysis, S. Maria degli Angeli Hospital , Putignano; Prof. V. Marinelli, Division of Nephrology, Civil Hospital, Scafati (SA); Dr. F. Perticone, Department of Cardiology, Mater Domini Hospital, Catanzaro; Dr. F. Scanferla , Division of Nephrology and Dialysis, Umberto I Civil Hospital, Mestre (VE); Prof. F. P. Schena, Division of Nephrology, University of Bari; Dr. Renzo Tarchini, Division of Nephrology and Dialysis, C Poma Civil Hospital, Mantova.

REFERENCES

1. Whelton, P.K. Epidemiology of hypertension. Lancet **1994**, *344*, 101–106.
2. Working Group on Primary Prevention of Hypertension. Report of the National High Blood Pressure Education Program Working Group on Primary Prevention of Hypertension. Archives of Internal Medicine **1993**, *151*, 186–208.
3. The Fifth Report of the Joint National Committee on Detection, Evaluation, and Treatment of High Blood Pressure (JNC V). Archives of Internal Medicine **1993**, *153*, 154–183.
4. Danielson, H.; Kornerup, H.; Olsen, S.; Posberg, V. Arterial hypertension in chronic glomerulonephritis. An analysis of 310 cases. Clinical Nephrology **1983**, *19*, 3353–3358.
5. Brazy, P.C.; Fitzwilliam, J.F. Progressive renal disease: role of race and antihypertensive medications. Kidney International **1990**, *37*, 1113–1119.
6. The National High Blood Pressure Education Program Working Group: National High Blood Pressure Education Program Working Group Report on Hypertension in Diabetes. Hypertension **1994**, *8*, 2–6.
7. Epstein, M. Calcium antagonists and renal protection: current status and future perspectives. Archives of Internal Medicine **1992**, *152*, 1573–1584.
8. Bretzel, R.G. Protecting the residual renal function: which drugs of choice? American Journal of Hypertension **1997**, *10*, 159s–166s.

MARCEL DEKKER, INC. • 270 MADISON AVENUE • NEW YORK, NY 10016

©2003 Marcel Dekker, Inc. All rights reserved. This material may not be used or reproduced in any form without the express written permission of Marcel Dekker, Inc.

9. Takabatake, T.; Ohta, H.; Sasaki, T.; Satoh, S.; Ohta, K.; Ise, T.; Kobayashi, K. Renal effects of manidipine hydrochloride. European Journal of Clinical Pharmacology **1993**, *45*, 321–325.
10. Gault, M.H.; Longerich, L.L.; Harnett, J.D.; Wesolowsky, C. Predicting glomerular function from adjusted serum creatinine. Nephron **1992**, *62*, 249–256.
11. Neaton, J.D.; Grimm, R.H., Jr.; Prineas, R.J. Treatment of mild hypertension study. JAMA **1993**, *270*, 713–724.
12. Elliot, W.J. Drug therapy and drops out in a tertiary hypertension clinic. American Journal of Hypertension **1994**, *25*, 1356.
13. Fogari, R.; Zoppi, A.; Corradi, L.; Preti, P.; Malalamani, G.D.; Mugellini, A. Effect of different dyhydropyridine calcium antagonist on plasma norepinephrine in essential hypertension. J. Hypertens. **2000**, *18*, 1871–1875.
14. Van Zwieten, P.A.; Pfaffendorf, M.; Mancia, G. Attempts to improve the selectivity of calcium antagonistic drugs. Blood Pressure **1996**, *5* (suppl 5), 7–9.
15. Epstain, M. Calcium antagonist and the kidney. American Journal of Hypertension **1993**, *6*, 251s–259s.
16. Sabbatini, M.; Leonardi, A.; Testa, R.; Vitaioli, L.; Amenta, F. Effects of calcium antagonists on glomerular arterioles in spontaneously hypertensive rats. Hypertension **2000**, *35*, 775–779.
17. Keane, W.J.; Raij, L. Relationship among altered glomerular barrier permselectivity, angiotensin II, and mesangial uptake of macromolecules. Laboratory Investigation **1985**, *52*, 599–604.

MARCEL DEKKER, INC. • 270 MADISON AVENUE • NEW YORK, NY 10016
©2003 Marcel Dekker, Inc. All rights reserved. This material may not be used or reproduced in any form without the express written permission of Marcel Dekker, Inc.

Copyright © 2003 EBSCO Publishing

Copyright of Renal Failure is the property of Taylor & Francis Ltd and its content may not be copied or emailed to multiple sites or posted to a listserv without the copyright holder's express written permission. However, users may print, download, or email articles for individual use.
