# Supplementary material for: Effects of T-Type Calcium Channel Blockers on Renal Function and Aldosterone in Patients with Hypertension: A Systematic Review and Meta-Analysis
Source: PLoS One. 2014 Oct 17;9(10):e109834. doi: 10.1371/journal.pone.0109834 (PMC4201480; doi:10.1371/journal.pone.0109834)
Supplement: File S3 — PDF files of twenty-four studies included in the meta-analysis. (ZIP) [file pone.0109834.s007.zip › Supporting information-PDF files/29. Clinical drug of the world 2013[34(7)]401-404.pdf]

【简报】

贝尼地平联合贝那普利治疗高血压肾病的疗效观察

韩荣旗，罗朝利，姜 梅，李红月，单福军
（中国人民解放军北京军区北戴河疗养院药剂科，秦皇岛 066000）

摘要：**目的** 观察贝尼地平联合贝那普利治疗高血压肾病的临床疗效。**方法** 120 例高血压肾病患者随机分为 3 组 ：A 组口服贝尼地平一日 4 mg、B 组口服贝那普利一日 10 mg、C 组联用贝尼地平和贝那普利治疗，用法用量同 A、B 组。疗程均为 12 周，观察治疗前后 3 组患者血压和肾功能变化以及不良反应。**结果** 与治疗前相比，治疗后 3 组患者血压和 24 h 尿蛋白定量均明显降低（$P < 0.01$），且与 A 组和 B 组比较，C 组降低更为显著（$P < 0.01$）。3 组均未见严重不良反应发生。**结论** 贝尼地平联合贝那普利治疗高血压肾病可有效控制血压，降低 24 h 尿蛋白，疗效及安全性好。

关键词：贝尼地平；贝那普利；高血压肾病
中图分类号：R972^{+}.4；R544.1^{+}4　**文献标志码**：A　**文章编号**：1672-9188(2012)07-0401-04

The efficacy of benidipine combined with benazepril in the treatment of hypertension nephropathy

HAN Rong-qi, LUO Chao-li, JIANG Mei, LI Hong-yue, SHAN Fu-jun
(*The Beidaihe Sanatorium in Beijing Military, Qinhuangdao 066000,China*)

Abstract: ***Objective*** To observe the efficacy of benidipine and benazepril on hypertension nephropathy. ***Methods*** Totally 120 patients with hypertension nephropathy were randomly divided into 3 groups: group A (oral administration of 4 mg/d of benidipine), group B (oral administration of 10 mg/d of benazepril)and group C (combination of benidipine and benazepril, the usage was same to group A, B). After 12 weeks of treatment, the changes of blood pressure and renal function before and after treatment and adverse reactions were observed. ***Results*** The blood pressure and 24 h urinary protein of patients were significantly decreased after treatment with benidipine and benazepril ($P<0.01$). The group C was superior to group A and B($P<0.01$). There was no serious adverse reactions found among 3 groups. ***Conclusion*** The treatment of hypertension nephropathy with combination of benidipine and benazepril can effectively control the level of blood pressure and reduce 24 h urinary protein.

Key words: benidipine; benazepril; hypertension nephropathy

高血压肾病系原发性高血压引起的良性小动脉肾硬化和恶性小动脉肾硬化，并伴有相应临床表现的疾病，是高血压常见并发症及致患者肾衰竭及心血管意外的主要原因。蛋白尿的出现能较早期显示肾脏损害，不仅是肾损害的标志之一，也是促进肾脏病变进展的独立危险因素 [1]。因此，选择良好的降压及保护肾脏的药物，是治疗高血压肾病和降低高血压肾病患者死亡率的重要保障。有效控制血压和及早干预蛋白尿对于控制和逆转高血压患者肾脏损害意义重大。本研究选择钙拮抗剂（CCB）贝尼地平和血管紧张素转化酶抑制剂（ACEI）贝那普利进行高血压肾病治疗，观察并评价这两类药物单用和联合应用在降压和保护肾脏中的临床疗效。

1 资料与方法

1.1 一般资料

选择 2010 年 10 月—2012 年 10 月在我院住院

收稿日期：2013-04-01；修回日期：2013-04-29
作者简介：韩荣旗，副主任药师，研究方向：临床药学。

治疗的高血压肾病患者 120 例，均为 2 级高血压且伴蛋白尿。其中，男性 78 例，女性 42 例，年龄 42 ～ 65 岁，平均 (48.6±6.8) 岁。入选患者均排除继发性高血压、糖尿病、冠心病、严重心脑血管疾病 (如急性心肌梗死、急性脑血管意外和充血性心力衰竭)、原发性肾脏病变和肝脏损害等。本研究已经我院伦理委员会批准，患者知情同意。

1.2　药品

贝尼地平，商品名 ：可力洛，规格 ：8 mg/ 片 ×7 片 / 盒，批号 ：181AIE，生产厂家 ：日本协和发酵工业株式会社。贝那普利，商品名 ：洛汀新，规格 ：10 mg/ 片 ×14 片 / 盒，批号 ：X1375，生产厂家 ：北京诺华制药。

1.3　治疗方法

120 例入选患者治疗前 1 周停服降压药，随机分为 A 组 (贝尼地平)、B 组 (贝那普利) 和 C 组 (贝尼地平＋贝那普利)，各 40 例。A 组予贝尼地平一日 4 mg，早晨口服 1 次 ；B 组予贝那普利一日 10 mg，早晨口服 1 次 ；C 组予贝尼地平一日 4 mg 联合贝那普利一日 10 mg 治疗，早晨口服 1 次。3 组均连续用药 12 周。每天测血压 1 次，如 4 周后血压未降至 130 mmHg /80 mmHg 以下，贝尼地平增至一日 8 mg, 贝那普利增至一日 20 mg, 早晨 1 次，顿服。

1.4　观察指标

1.4.1　血压

分别观察患者治疗前及治疗后的收缩压、舒张压，应用袖带水银柱式血压计，取患者坐位测右肱动脉血压。所有患者治疗前 1 周停服降压药后及治疗期间最后 1 周测 7 次血压，于每日上午 8 ～ 9 时测 1 次血压，连续测 7 d，取其平均值作为治疗前基础血压和治疗后血压。

1.4.2　肾功能检查

治疗前后分别采集静脉血和留取 24 h 尿液标本，测定各组患者治疗前后的尿素氮 (BUN)、肌酐清除率 (CCr)、血肌酐 (SCr)、血尿 β_2 微球蛋白 (β_2-M)、24 h 尿白蛋白 (Alb)、肾小球滤过率 (GFR) 及 24 h 尿蛋白等生化指标，并记录不良反应情况。BUN、CCr 和 SCr 测定应用全自动生化分析仪 (奥林巴斯 AU640)，血尿 β_2-M 及 24 h 尿 Alb 应用西门子试剂盒测定，GFR 测定应用 99 mTc-dtpa 肾动态显像定，24 h 尿蛋白定量测定采用比色法。

1.5　统计学方法

数据由 Excel 建立数据库，导入 SPSS 12.0 软件进行统计学分析，对于服从正态分布及方差齐性的数据，计数资料以百分率表示，采用 χ^2 检验，计量资料以均数 ± 标准差 ($\bar{x}\pm s$) 表示，采用 t 检验。以 P < 0.05 为差异有统计学意义。

2　结果

2.1　一般资料

患者性别、年龄、病程、收缩压、舒张压、BUN、CCr、SCr、血 / 尿 β_2-M、24 h 尿 Alb、GFR 及 24 h 尿蛋白等组间比较，差异无统计学意义，具有可比性，见表 1。

2.2　血压变化情况

与治疗前相比，治疗后三组收缩压和舒张压均显著下降 (P < 0.01)，且与 A、B 组比较，C 组改善更为显著，组间差异有统计学意义 (P < 0.01,

表 1　高血压肾病患者的一般资料 ($\bar{x}\pm s$，n=40)

组别	年龄/岁	例数(男/女)	收缩压/舒张压/mmHg	24 h尿Alb/mg	BUN/mmol·L^{-1}	CCr/mL·min^{-1}	SCr/μmol·L^{-1}	血/尿β_2-M/mg·L^{-1}	GFR/mL·min^{-1}	24 h尿蛋白/g·L^{-1}
A组	48.4	29/11	153.2±8.5/96.8±5.7	104.6±42.8	5.21±0.98	114.6±17.6	87.5±21.5	2.16±0.30/0.121±0.016	112.2±15.2	0.241±0.063
B组	48.6	25/15	154.8±8.7/97.2±5.6	102.6±35.9	5.40±0.96	113.6±18.5	97.2±22.5	2.08±0.36/0.116±0.034	111.9±15.3	0.241±0.062
C组	48.8	24/16	155.6±8.3/97.3±5.8	101.9±36.5	4.95±1.08	115.7±19.2	91.3±26.7	2.16±0.27/0.116±0.019	110.9±15.4	0.231±0.067

表 2　高血压肾病患者治疗前后收缩压和舒张压变化

($\bar{x}\pm s$, n=40)

组别	收缩压/mmHg		舒张压/mmHg	
	治疗前	治疗后	治疗前	治疗后
A组	153.2±8.5	135.7±7.5*	96.8±5.7	85.5±6.6*
B组	154.8±8.7	137.2±7.4*	97.2±5.6	86.8±5.4*
C组	155.6±8.3	130.4±6.4*,Δ	97.3±5.8	79.9±5.3*,Δ

与本组治疗前相比，*P＜0.01；与 A、B 组治疗后相比，ΔP＜0.01

见表 2)。

2.3　24 h 尿 Alb、24 h 尿蛋白、血 / 尿 β_2-M、尿蛋白 / 尿肌酐和肾功能指标评价

3 组患者治疗 12 周后，24 h 尿 Alb、24 h 尿蛋白、血 / 尿 β_2-M 和尿蛋白 / 尿肌酐、SCr 较治疗前均下降 (P＜0.01)，GFR 和 CCr 较治疗前均升高 (P＜0.01)，与 A、B 组比较，C 组变化更为显著 (P＜0.01，表 3)。

2.4　不良反应

A 组 5 例出现头痛、面部潮红和四肢冰冷，不良反应发生率为 12.5%。B 组 5 例出现咳嗽和头晕，不良反应发生率为 12.5%。C 组 3 例出现咳嗽和头晕，不良反应发生率为 7.5%。症状均较轻微，未影响继续治疗，并在治疗过程中逐渐自行缓解；未见其他严重不良反应发生，3 组比较，差异无统计学意义 (表 4)。

3　讨论

高血压持续存在可引起肾脏小动脉硬化，管壁增厚，管腔变窄，继发肾实质缺血性损害，造成良性小动脉性肾硬化症，患者可出现尿蛋白阳性，待血肌酐和尿素氮增高时，肾脏病变已进入失代偿期。采用不同降压机制药物组合，可在血压控制，特别是血压达标、靶器官保护和代谢方面发挥协同效应。联合治疗有益于患者血压检测 [3]。

ACEI 是高血压肾病治疗的基石，多项随机临床研究均提示 ACEI 可降压和减少尿蛋白，保护慢性肾脏疾病患者的肾功能，明显延缓慢性肾脏疾病进展 [4-5]。《中国高血压防治指南 2010》[6] 指出：ACEI 或血管紧张素受体拮抗剂既有降压，又有降低蛋白尿的作用，因此，对于高血压伴肾脏病患者，尤其有蛋白尿患者，应作为首选。贝那普利是第三代 ACEI，可有效降低血管紧张素 II 浓度发挥降压作用。同时其能扩张肾小球小动脉，改善入球小动脉的阻力，进而降低肾小球血管压力，延缓或防治高血压肾病的发生。

CCB 主要通过阻断血管平滑肌细胞上的钙离子通道发挥扩张血管降低血压的作用，同时可降低肾小球内压和尿蛋白，保护肾脏功能。此外 CCB 通过调节大分子物质通过肾小球基底膜，降低残余肾组织的代谢活性、减少自由基形成等机制达到保护

表 3　高血压肾病患者治疗前后 24 h 尿 Alb、尿蛋白与血 / 尿 β_2-M、尿蛋白 / 尿肌酐以及肾功能评价指标变化

($\bar{x}\pm s$, n=40)

组别	时间	BUN/mmol·L^{-1}	CCr/mL·min^{-1}	SCr/μmol·L^{-1}	血/尿β_2-M/mg·L^{-1}	24 h尿Alb/mg	GFR/mL·min^{-1}	24 h尿蛋白/g·L^{-1}	尿蛋白/尿肌酐(g/gcr)
A组	治疗前	5.21±0.98	114.6±17.6	87.5±21.5	2.16±0.30/0.121±0.016	104.6±42.8	112.2±15.2	0.241±0.063	0.321±0.084
	治疗后	5.10±0.93*	118.7±14.9*	82.4±20.9*	1.76±0.31*/ 0.088±0.023*	66.2±26.4*	124.8±15.6*	0.166±0.031*	0.221±0.041*
B组	治疗前	5.40±0.96	113.6±18.5	97.2±22.5	2.08±0.36/0.116±0.034	102.6±35.9	111.9±15.3	0.241±0.062	0.321±0.082
	治疗后	4.85±0.93*	116.4±22.6*	92.6±22.1*	1.83±0.26*/0.089±0.023*	68.5±29.4*	120.8±17.4*	0.168±0.032*	0.223±0.043*
C组	治疗前	4.95±1.08	115.7±19.2	91.3±26.7	2.16±0.27/0.116±0.019	101.9±36.5	110.9±15.4	0.231±0.067	0.307±0.089
	治疗后	4.70±0.98*,Δ	120.5±18.5*,Δ	80.9±22.2*,Δ	1.62±0.22*,Δ/ 0.072±0.024*,Δ	44.6±16.7*,Δ	130.9±17.5*,Δ	0.109±0.040*,Δ	0.145±0.053*,Δ

与本组治疗前相比，*P＜0.01；与 A、B 组治疗后相比，ΔP＜0.01

表4 3组不良反应比较（n=40）

组别	头痛	面部潮红	咳嗽	头晕	四肢冰冷	总发生率
A组	2	2	0	0	1	12.5%
B组	0	0	3	2	0	12.5%
C组	0	0	2	1	0	7.5%

肾脏功能的目的[7-8]。贝尼地平是新型长效CCB，对3种钙通道（L、N和T型）都具有较高的结合率，对钙通道中的T型钙通道具有独特的阻滞作用[9]。T型钙通道在肾小球出球小动脉占主要地位，贝尼地平可以同时扩张肾脏入、出球小动脉，增加肌酐清除率，除具有强大的降压作用外，还具有多方面的肾脏保护作用[10]。由于贝尼地平对肾功能的独特保护作用，可作为高血压患者控制血压和防止肾脏损害的首选药物。

本研究显示，贝尼地平联合贝那普利对高血压肾病患者治疗效果明显优于2种药物单独使用。3组治疗后收缩压和舒张压都较治疗前明显下降，且联用组下降更为明显。3组均未见严重不良反应发生。

4 结论

综上所述，采用贝尼地平联合贝那普利治疗高血压肾病在改善高血压和改善肾功能等方面有协同作用，且具有良好的安全性和耐受性。

参考文献：

[1] 赵静茜，晋万强．坎地沙坦与拉西地平联合应用对高血压患者蛋白尿的影响[J]．中华高血压杂志，2010，18(11)：1085-1087.

[2] 王芹．氯沙坦联合硝苯地平对老年2型糖尿病肾病合并高血压患者肾功能的影响[J]．中国药业，2012，21(8)：38-39.

[3] 戴伦．高危高血压患者可否直接起始三联药物[J]．中华高血压杂志，2012，20(4)：307-311.

[4] 郝柯，姜红，曾朝荣．慢性肾脏疾病的血压目标及降压药物选择[J]．中华高血压杂志，2008，16(12)：1147-1149.

[5] 侯凡凡，谢进．慢性肾脏病应用肾素-血管紧张素系统阻断剂的治疗目标[J]．中华高血压杂志，2007，7(15)：32-33.

[6] 中国高血压防治指南修订委员会．中国高血压防治指南2010[J]．中华高血压杂志，2011，19(8)：701-743.

[7] 王玉慧，罗朝利，史文慧，等．慢性肾功能不全合并高血压的临床用药选择[J]．中国药业，2011，20(22)：70-71.

[8] 宫明莲，高宇新，张静．贝尼地平和缬沙坦联合治疗对高血压病患者肾功能的影响[J]．中国医药导报，2012，9(14)：95-96.

[9] 王纯，杨光敏，罗梅宏，等．盐酸贝尼地平治疗稳定性心绞痛的疗效观察[J]．临床医药实践，2008,1 7(B07)：552-555.

[10] 刘国树．钙离子通道阻滞剂治疗高血压病新进展[J]．中国药物应用与检测，2006，4(6)：1-4.

（责任编辑：李 琨）

广告索引

封二 浙江普洛康裕制药有限公司
封三 江苏恒瑞医药股份有限公司
封底 上海新亚药业有限公司
前插1 山西振东制药股份有限公司
前插2 山东罗欣药业股份有限公司
前插3 上海医工院医药有限公司
前插4 上海现代制药股份有限公司
前插5 江苏豪森药业股份有限公司
中插1 辰欣药业股份有限公司
中插2 合肥立方制药股份有限公司
