# Supplementary material for: Effects of T-Type Calcium Channel Blockers on Renal Function and Aldosterone in Patients with Hypertension: A Systematic Review and Meta-Analysis
Source: PLoS One. 2014 Oct 17;9(10):e109834. doi: 10.1371/journal.pone.0109834 (PMC4201480; doi:10.1371/journal.pone.0109834)
Supplement: File S3 — PDF files of twenty-four studies included in the meta-analysis. (ZIP) [file pone.0109834.s007.zip › Supporting information-PDF files/30. Chinese Medicine 2012[9(14)] 95-96+99.pdf]

贝尼地平和缬沙坦联合治疗对高血压病患者肾功能的影响

宫明莲　高宇新　张　静
辽宁省大连市第五人民医院心内科，辽宁大连　116021

[摘要] 目的 探讨贝尼地平、缬沙坦单独治疗和联合治疗对高血压病患者肾脏的影响。方法 将 140 例高血压病患者分为三组：A 组（口服贝尼地平 4 mg）、B 组（口服缬沙坦 80 mg）、C 组（口服贝尼地平 4 mg 和缬沙坦 80 mg）。疗程 24 周，观察治疗前后血压和肾功能的变化。结果 C 组降低血压和降低尿蛋白排泄的幅度优于 A、B 组（$P < 0.01$），治疗后肾小球滤过率在 C 组及 A、B 组之间，差异有高度统计学意义（$P < 0.01$）。结论 贝尼地平、缬沙坦长期单独治疗均可减少蛋白尿，保护肾脏，两药联合治疗对减少蛋白尿、保护肾脏有一定协同作用。

[关键词] 高血压病；贝尼地平；缬沙坦；肾功能
[中图分类号] R544.1　[文献标识码] A　[文章编号] 1673-7210(2012)05(b)-0095-03

Influence of Benidipine and Valsartan combination therapy on renal function of patients with hypertension

GONG Minglian　GAO Yuxin　ZHANG Jing
Department of Cardiology, Dalian Fifth People´s Hospital, Liaoning Province, Dalian　116021, China

[Abstract] Objective To investigate the influence of Benidipine and Valsartan monotherapy and combination therapy on the renal function of patients with hypertension. **Methods** 140 patients with hypertension were randomly divided into three groups: group A (oral administration of 4 mg of Benidipine), group B (oral administration of 80 mg of Valsartan) and group C (oral administration of 4 mg of Benidipine and 80 mg of Valsartan). After 24 weeks of treatment, the changes of blood pressure and renal function before and after treatment were observed. **Results** The group C was superior to group A and B in the reduction of blood pressure and urinary protein excretion ($P < 0.01$). After treatment, the glomerular filtration rate was different among group C with group A and B, with statistical significance ($P < 0.01$). **Conclusion** Long-term Benidipine and Valsartan monotherapies can both reduce proteinuria and protect the kidneys. The combination therapy has certain synergistic effect on the reduction of proteinuria and protection of kidneys.

[Key words] Hypertension; Benidipine; Valsartan; Renal function

高血压是心内科的常见病、多发病，致死、致残率高。肾损害是其严重的并发症之一，肾脏分泌多种物质调节人体血压，是高血压损害的主要靶器官之一。高血压病患者发生尿白蛋白的程度显著高于血压正常者[1]。因此，选用减轻高血压肾损害、减少尿蛋白排泄、保护肾功能的药物对于有效地降低血压尤为重要。本研究旨在探讨贝尼地平、缬沙坦联合治疗对高血压病患者肾脏的影响。现报道如下：

1 资料与方法

1.1 一般资料

选择 2009 年 1 月~2011 年 1 月在我院住院治疗的高血压合并早期肾损害 140 例，均为 2 级且伴蛋白尿，其中，男84 例，女 56 例；年龄 40~68 岁，平均（53.0±6.5）岁。排除继发性高血压、糖尿病、冠心病、原发性肾脏病变、心衰、肾衰、肺部疾病和肝脏损害等。服用 2 周安慰剂后 24 h 尿白蛋白在 50~200 mg，将 140 例入选患者分为 A 组（45 例，贝尼地平）、B 组（45 例，缬沙坦）、C 组（50 例，贝尼地平+缬沙坦）三组，三组患者年龄、性别、血压等基础资料比较，差异无统计学意义（$P > 0.05$），具有可比性。

1.2 治疗方法

A 组予贝尼地平 4 mg，早晨 1 次，口服；B 组予缬沙坦80 mg，早晨 1 次，口服；C 组予贝尼地平 4 mg、缬沙坦 80 mg，早晨 1次，口服。每天测血压 1 次，如 4 周后血压未降至 140/90 mm Hg（1 mm Hg=0.133 kPa）以下，贝尼地平增至 8 mg，增至缬沙坦 160 mg，早晨 1 次，顿服。疗程 24 周，所有患者治疗前及治疗后 1 周测 7 次血压取其平均值为治疗后血压，并测定肌酐清除率（CCr）、24 h 尿白蛋白（Alb）、24 h 尿蛋白、血尿 β_2 微球蛋白（β_2-M）、尿素氮（BUN）、血肌酐（SCr）及肾小球滤过率（GFR）。BUN、SCr、CCr 测定应用全自动生化分析仪（日本岛津），血尿 β_2-M 及 24 h 尿 Alb 应用放射免疫法测定（试剂盒由中国原子能科学研究院提供），24 h 尿蛋白定量测定应用磺基水杨酸法，GFR 测定应用 99mTc-dtpa 肾动态显像定。

1.3 统计学方法

数据由 Excel 建立数据库，导入 SPSS 12.0 软件进行统计学分析，对于服从正态分布及方差齐性的数据，计数资料以百分率表示，采用 χ^2 检验，计量资料以均数±标准差（$\bar{x}\pm s$）表示，采用 t 检验。以 $P < 0.05$ 为差异有统计学意义。

2 结果

2.1 三组治疗前后血压变化比较

三组患者治疗 24 周后，收缩压、舒张压较治疗前均下降（$P < 0.01$），C 组较 A、B 组下降更明显（$P < 0.01$），且达到目标值。见表 1。

表 1 三组治疗前后血压变化比较（$\bar{x}\pm s$）

组别	例数	时间	收缩压	舒张压
A 组	45	治疗前	157.2±8.3	97.1±5.4
		治疗后	135.9±7.6*	82.4±5.3*
B 组	45	治疗前	158.1±9.6	96.8±6.1
		治疗后	133.8±7.4*	83.4±4.8*
C 组	50	治疗前	157.9±8.9	97.2±5.1
		治疗后	120.6±6.2*#	75.7±4.8*#

注：与本组治疗前比较，*$P < 0.01$；与 A、B 组比较，#$P < 0.01$

2.2 三组治疗前后 24 h 尿 Alb、24 h 尿蛋白、血尿 β_2-M 变化比较

三组患者治疗 24 周后，24 h 尿 Alb、24 h 尿蛋白、血 β_2-M、尿 β_2-M 较治疗前均下降（$P < 0.01$），联合治疗组较单药治疗组下降更明显（$P < 0.01$）。见表 2。

表 2 三组治疗前后 24 h 尿 Alb、24 h 尿蛋白、血尿 β_2-M 变化比较（$\bar{x}\pm s$）

组别	例数	时间	24 h 尿 Alb（mg/24 h）	24 h 尿蛋白（g/L）	血 β_2-M（mg/L）	尿 β_2-M（mg/L）
A 组	45	治疗前	102.9±36.2	0.241±0.063	2.08±0.35	0.117±0.034
		治疗后	68.7±29.3*	0.169±0.032*	1.83±0.26*	0.089±0.023*
B 组	45	治疗前	104.8±42.8	0.241±0.062	2.16±0.30	0.121±0.017
		治疗后	66.4±26.5*	0.169±0.043*	1.76±0.31*	0.092±0.020*
C 组	50	治疗前	101.8±36.4	0.231±0.067	2.15±0.27	0.116±0.019
		治疗后	44.6±16.8*#	0.109±0.040*#	1.62±0.22*#	0.072±0.024*#

注：与本组治疗前比较，*$P < 0.01$；与 A、B 组比较，#$P < 0.01$

2.3 三组治疗前后肾功能指标变化比较

三组患者治疗 24 周后，GFR、CCr 较治疗前均升高（$P<0.01$），C 组较 A、B 组升高更明显 （$P < 0.01$）；SCr 较治疗前均下降（$P < 0.01$），C 组较 A、B 组下降更明显（$P < 0.01$）。见表 3。

表 3 三组治疗前后肾功能指标变化比较（$\bar{x}\pm s$）

组别	例数	时间	GFR（mL/L）	CCr（L/24 h）	BUN（mmol/L）	SCr（μmol/L）
A 组	45	治疗前	112.4±15.3	113.6±18.5	5.40±0.92	97.4±22.5
		治疗后	120.8±17.4*	116.4±22.6*	4.80±0.93	92.6±22.1*
B 组	45	治疗前	111.8±13.2	114.5±17.8	5.20±0.98	87.5±21.5
		治疗后	124.9±15.6*	118.7±14.9*	5.10±0.93	82.4±20.9*
C 组	50	治疗前	110.9±15.3	115.8±19.2	4.90±1.08	91.3±26.7
		治疗后	130.9±17.5*#	120.5±18.5*#	4.70±0.98	80.9±22.1*#

注：与本组治疗前比较，*$P < 0.01$；与 A、B 组比较，#$P < 0.01$

3 讨论

高血压是导致肾脏疾病的重要危险因素，约 18%的高血压患者最终出现肾功能不全。美国肾脏病学会的统计资料表明，每 13 个高血压患者中就有 1 例发展成终末期肾病（ESRD）。本组笔者将 140 例高血压病患者分为 A 组、B 组和 C 组，疗程 24 周，疗程结束后，C 组降低血压和降低尿蛋白排泄的幅度优于 A、B 组（$P < 0.01$），治疗后肾小球滤过率在 C 组及 A、B 组之间，差异有高度统计学意义（$P < 0.01$），说明贝尼地平、缬沙坦长期单独治疗均可减少蛋白尿，保护肾脏，两药联合治疗对减少蛋白尿，保护肾脏有一定协同作用，与相关报道一致[3]。

钙通道阻滞剂可能是通过以下机制保护肾脏功能：钙通道阻滞剂扩张肾小球入球小动脉作用大于扩张出球小动脉作用，使肾脏血流量增加和肾小球滤过率升高，改善肾脏缺血状态，同时也造成肾小球内压力升高，损害肾小球基底膜，加重肾脏的损伤。但钙通道阻滞剂作用于周围血管系统，使全身血压降低，抵消了扩张入球小动脉造成的肾小球内压升高，总效果是降低肾小球内压、降低尿蛋白和保护肾脏功能。此外钙通道阻滞剂通过调节大分子物质通过肾小球基底膜，降低残余肾组织的代谢活性、减少自由基形成等机制达到保护肾脏功能的目的。

贝尼地平属于二氢吡啶类钙通道阻滞剂，对 3 种钙通道（L、T 和 N 型）都具有较高的结合率。T 型钙通道在肾小球出球小动脉占主要地位，因此贝尼地平可以扩张出球小动脉，减轻内压，保护肾脏功能[4-7]。而其他类型的钙通道阻滞剂主要与 L 型通道结合，对出球小动脉的作用较轻微。贝尼地平的另一个优点是，其经胃肠道吸收并由血管平滑肌细胞膜摄取后，在细胞膜内移动并与钙通道结合，缓慢释放，因此降压作用平缓，即使药物分子在血流中消失，其作用仍然持续存在[8-9]。

缬沙坦是一种新型的抗高血压药物，通过阻断 Ang Ⅱ介导的生理效应，阻断相关的血管紧张素Ⅰ受体（AT1），松弛血管平滑肌，扩张血管，提高肾血流灌注量，增加水钠排泄，减少血容量，而使血压下降，起到降压和保护靶器官作用[11-14]。李德环等[12]选取 110 例高血压并出现早期肾损害患者，口服给予缬沙坦治疗 8 周，观察、评价缬沙坦降压疗效的同时，收集患者尿样，采用放免法测定治疗前后的尿微量白蛋白（MA）水平。结果显示，给予缬沙坦治疗后，早期高血压肾损害患者的收缩压、舒张压及尿 MA 水平均显著降低（$P < 0.01$）。以上证明，对高血压早期肾损害患者，缬沙坦可有效降压及保护肾脏功能。

综上所述，联合应用贝尼地平、缬沙坦片治疗有较好地降低血压和肾脏保护作用，值得临床推广应用。

[参考文献]

[1] Tamsma JT，Jazet IM，Beishuizen ED，et al. The metabolic sydrome：a vascular perspective [J]. Eur J Intem Med，2005，16（5）：314-320.

[2] 李为民，刘巍.2005 年中国高血压防治指南评价[J].中国实用内科杂志，2007，27（12）：899-903.

[3] Qin XP，Ye F，Liao DF，et al. Involvement of calciton in generelated peptide in depressor effects of losartan and perindopril in rats [J]. Eur J Pharmacol，2003，464（1）：63-67.

[4] Uzu T，Nishimura M，Fujii T，et al. Benidipine attenuates glomerruler hypertension and reduces albuminuria in patients with metabolic syndrome [J]. Hypertens Res，2007，30：161-165.

[5] Akizuki O，lnayoshi A，Kitayama T，et al. Blockade of T-type voltage-

（下转第 99 页）

of schizophrenia [J]. Plos Med，2005，2(5)：413-433.
[2] Edward K，Shaloo G，Susan B，et al. Adherence and outcomes associated with copayment burden in schizophrenia：a cross-sectional survey [J]. J Med Econ，2010，13(2)：185-192.
[3] Leucht S，Burkard T，Henderson J，et al. Physical illness and schizophrenia：a review of the literature [J]. Aeta Psychiatr Scand，2007，116(5)：317-333.
[4] Bellivier F. Schizophrenia antipsychotics and diabetes：genetic aspects [J]. Eur Psychiatry，2005，20(S4)：335-339.
[5] Mehta S，Johnson ML，Chen H，et al. Risk of cerebrovascular adverse events in older adults using antipsychotic agents：a propensity-matched retrospective cohort study [J]. J Clin Psychiat，2010，71(6)：689.
[6] Ren XS，Herz L，Qian，et al. Measurement of treatment adherence with antipsychotic agents in patients with schizophrenia [J]. Neuropsychiatr Dis Treat，2009，5：491-498.
[7] Ray WA. Both typical and atypical antipsychotic agents were associated with increased risk for sudden cardiac death [J]. Ann Intern Med，2009，151(4)：2-12.
[8] McIntyre RS，Jerrell JM. Antipsychotic agents and cardiometabolic morbidity in youth reply [J]. Arch Pediat Adol Med，2009，163(4)：395.
[9] 刘俊彪，吴靖华.氯丙嗪、氯氮平、利培酮对首发精神分裂症患者糖代谢、血脂和体重的影响[J].中国神经精神疾病杂志，2004，30(4)：293-295.
[10] 郑一瑾，王高华，程自立，等.氯氮平和利培酮对首发精神分裂症患者糖代谢影响的研究[J].中华精神科杂志，2003，36(4)：207-210.
[11] Citrome LL. The increase in risk of diabetes mellitus from exposure to second-generation antipsychotic agents [J]. Drugs Today (Barc)，2004，40：445-464.
[12] 陶建青，曾强，刘佳斌，等.氯氮平与利培酮对精神分裂症患者血糖、血脂、体重影响的 meta 分析[J].中国神经精神疾病杂志，2007，33(12)：747-749.
[13] Watanabe J，Suzuki Y，Fukui N，et al. Hypoglycemia asso ciated with second generation antipsychotic agents [J]. Int J Neuropsychop，2010，13(S1)：111.
[15] 吴仁容，赵靖平.四种抗精神病药对糖代谢及脂代谢的不良影响[J].中华精神科杂志，2005，38(3)：130-133.
[16] 刘燕，李华芳，王慧芳，等.氯氮平和利培酮对精神分裂症病人体重、瘦素及脂代谢的影响[J].中国新药与临床杂志，2004，23(9)：579-582.
[17] Kraus T，Haack M，Schuld A，et al. Body weight and leptin plasma levels during treatment with antipsychotic drugs [J]. Am J Psychiatry，1999，156：312-314.
[18] 江学锋，李则挚，吴志国，等.利培酮与舒必利对首发精神分裂症患者血脂代谢的影响[J].临床精神医学杂志，2011，21(5)：329-330.
[19] Ota M，Mori K，Nakashima A，et al. mRNA expression levels of leptin-related substances can be modified in risperidone-injected rats [J]. Biogenic Amines，2005，19(4-6)：299-308.
[20] Hung GCL，Kuo CJ，Huang MC，et al. Sex differences in the association of weight gain and risperidone efficacy among schizophrenic patients [J]. Progress in neuro-psychopharmacology & biological psychiatry，2010，34(8)：1523-1524.

（收稿日期：2012-03-28 本文编辑：张瑜杰）

（上接第 94 页）

[3] De Wald TA，Hernandez AF. Efficacy and safety of nesirtide in patients with acute decompensated heart failure [J]. Expert Rev Cardiovasc Ther，2010，8(2)：159-169.
[4] Rademaker MT，Charles CJ，Melton IC，et al. Monitoring of heart failure：comparison of left atrial pressure with intrathoracic impedance and natriuretic peptide measurements in an experimental model of ovine heart failure [J]. Clin Sci(Lond)，2011，120(5)：207-217.
[5] 易岂建.利钠肽与心力衰竭[J].儿科药学杂志，2007，13(2)：4-6.
[6] 符允衡.脑钠尿肽在心力衰竭中的临床研究现状和进展[J].内科，2009，4(6)：921-923.
[7] 吴建新，王一心，钮荣祥，等.心脉隆对大鼠异丙肾上腺素性缺血心肌的保护作用[J].中国病理生理杂志，2002，18(1)：97-98.
[8] 彭芳，刘晓波，方春生，等.心脉隆注射液对大鼠缺氧及缺氧-复氧心肌细胞内游离钙离子及脂质过氧化物的影响[J].药物研究，2003，12(1)：33-35.
[9] 吴建新，杨玲玲，袁国林，等."心脉隆"抗野百合碱性肺动脉高压的作用及机制探讨[J].医学研究杂志，2009，38(1)：29-32.
[10] 李树楠，张华明，杜一民.心脉隆对大鼠肠系膜微循环的效应观察[J].大理医学院学报，1992，1(1)：10-13.
[11] 何为.老年心力衰竭 313 例诱因分析[J].现代医院，2008，8(8)：29-30.

（收稿日期：2012-03-22 本文编辑：张瑜杰）

（上接第 96 页）

dependent Ca^{2+} channels by benidipine A dihydropyridine calcium channel blocker，inhibits aldosterone production in human adrenocortical cell line NCI-H295R [J]. Eur J Pharmacol，2008，584：424-434.
[6] Saito F，Fujita H，Takahashi A，et al. Renoprotective effect and cost-effectiveness of using benidipine，a calcium channel blocker，to lower the dose of angiotensin receptor blocker in hypertensive patients with albuminuria [J]. Hylxntens Res，2007，30：39-47.
[7] Hayashi K，Wakino S，Homma K，et al. Pathophysiological significance of T-type Ca^{2+} channel：role of T-type Ca^{2+} channel in renal microcirculation [J]. J Pharmacol Sci，2005，99：221-227.
[8] Gashti CN，Bakris GL. The role of calcium antagonists in chronic kidney disease [J]. Curr Opin Nephrol Hypertens，2004，13：155-161.
[9] Matsuzaki G，Ishizaka N，Furuta K，et al. Comparison of vasculoprotective effects of benidipine and losartan in a rat model of metabolic syndrome [J]. Eur J Pharmacol，2008，587：237-242.
[10] Croom KF，Curran MP，Goa KL，et al. Irbesartan：a review of its use in hypertension and in the management of diabetic nephropathy [J]. Drugs，2004，64：999-1028.
[11] 魏新彤.缬沙坦治疗原发性高血压病疗效观察[J].临床合理用药杂志，2010，3(6)：32-33.
[12] 李德环，赵琛.缬沙坦对高血压早期肾损害患者的疗效观察[J].青岛医药卫生，2011，43(2)：973.
[13] 欧茂发.缬沙坦与氢氯噻嗪联合治疗高血压病疗效观察[J].现代医院，2008，8(7)：67.
[14] 成迎晖.苯磺酸氨氯地平与缬沙坦联合治疗原发性高血压 48 例临床疗效[J].内科，2011，6(6)：558-559.

（收稿日期：2012-02-28 本文编辑：张瑜杰）
