# Supplementary material for: Effects of T-Type Calcium Channel Blockers on Renal Function and Aldosterone in Patients with Hypertension: A Systematic Review and Meta-Analysis
Source: PLoS One. 2014 Oct 17;9(10):e109834. doi: 10.1371/journal.pone.0109834 (PMC4201480; doi:10.1371/journal.pone.0109834)
Supplement: File S3 — PDF files of twenty-four studies included in the meta-analysis. (ZIP) [file pone.0109834.s007.zip › Supporting information-PDF files/31. China Pharmaceutical Guide 2012[10(27)]201-202.pdf]

说明低分子肝素在治疗急性冠状动脉综合征上拥有显著的疗效。另两组患者治疗7d后，患者出血情况以及血小板减少情况无明显差异（P[illegible]0.05），研究结果与文献报道一致。

综上所述，对急性冠状动脉综合征患者采用低分子肝素钙进行治疗具有明显的临床疗效，此药物使用安全可靠，不良反应小，故值得推广应用。

参考文献

[1] 胡大一.黄元铸.急性冠状动脉综合征[M].北京:人民卫生出版社.2001:1-2.

[2] 中华医学会心血管病学会.中华心血管病杂志编辑委员会.不稳定性心绞痛和非ST段抬高心肌梗死诊断与治疗指南[J].中华心血管病杂志.2007.35(4):295-297.

[3] 郭锋.林爱民.龚晓辉.低分子肝素钙治疗急性冠状动脉综合征79例临床疗效观察[J].医学综述.2008.14(8):1275-1276.

[4] 阮毅敏.低分子肝素钙联合冠心宁治疗不稳定性心绞痛66例临床观察[J].中医药导报.2008.14(3):19-20.

[5] 裁红果.赵学军.低分子肝素联合氯吡格雷治疗不稳定型心绞痛的临床观察[J].山西医药杂志.2008.37(1):41-42.

缬沙坦及贝尼地平对原发性高血压伴蛋白尿患者肾脏保护作用的研究

甘贱生　　甘　露

（江西省丰城市中医院心脑血管科，江西 丰城 331100）

【摘要】**目的** 探讨缬沙坦与贝尼地平对原发性高血压伴蛋白尿患者的疗效及保护肾脏差别。**方法** 随机选取2009年1月至2011年1月就诊我院的原发性高血压伴蛋白尿患者286例，分为A组（143例）和C组（143例），分别采用缬沙坦与贝尼地平治疗24周，对比两组患者治疗后的高血压总有效率、血清肌酐、肌酐清除率、尿微量蛋白的变化情况。**结果** 经治疗后，两组患者血压控制总有效率相似，肌酐清除率均升高，血清肌酐及尿微量蛋白均降低，两组之间差异无统计学意义，P[illegible]0.05。**结论** 缬沙坦及贝尼地平均能通过不同的途径有效地控制血压，保护肾脏，且疗效相当。

【关键词】缬沙坦；贝尼地平；原发性高血压；蛋白尿；肾脏

中图分类号：R544.1　　文献标识码：B　　文章编号：1671-8194（2012）27-0201-02

我国原发性高血压患病率逐年上升，而高血压长时间持续会引起全身小动脉硬化，重要脏器功血不足，尤其以肾脏最显著。随着肾脏功能的损伤，会出现如蛋白尿等一系列肾功能受损的表现。我院分别采用缬沙坦及与贝尼地平治疗原发性高血压伴蛋白尿患者，效果显著，现将实验报道如下。

1 资料与方法

1.1 一般资料

随机筛选2009年1月至2011年1月我院确诊为原发性高血压患者并且均伴有蛋白尿286例，其中其中男性175例，女性111例；年龄45~87岁，平均年龄（64.5±5.6）岁；1、2、3级高血压患者分别为98、75、113例。分为A组和C组，各143例。

1.2 治疗方法

治疗前均停止其他降压药1周，1周后A组采用缬沙坦胶囊（生产厂商：海南澳美华制药有限公司；国药准字H20030153）80mg/次口服治疗，1次/日。C组采用贝尼地平（生产厂商：华夏药业集团有限公司；国药准字H20030079）8mg/次口服治疗，1次/日。每天清晨6点，下午16点~18点及睡前23：00三个时间段均有固定医师测量两组患者的坐位血压以检测其变化。治疗24周。

1.3 评价项目及指标

分别记录治疗前后两组患者的血压、血清肌酐、肌酐清除率、尿微量蛋白并比较。高血压治疗效果依据如下：显效：治疗24周后，临床症状得到明显控制，收缩压下降≥30mmHg，或降至120~140mmHg。舒张压下降＞20mmHg，或降至70~90mmHg。有效：治疗24周后，临床表现好转，收缩压下降10~30mmHg，舒张压下降10~20mmHg。无效：治疗24周后，一般症状未得到控制，降压效果未达到上述标准，患者对治疗效果不满意。总有效率为显效、有效病例数与全部病例数之百分比。

1.4 统计学处理

本实验所有统计学计算采用SPSS17.0统计学软件完成，以P[illegible]0.05为有统计学意义。

2 结　果

2.1 两组患者高血压治疗效果

治疗24周后，两组患者症状得到有效控制，A组和C组总有效率达分别为95.8%和96.5%，两组无统计学差异，P[illegible]0.05。

2.2 两组治疗前后肾功能指标

治疗后两组患者的患肌酐清除率均升高，血清肌酐及尿微量蛋白均降低但无明显差异。见表1。

表1　治疗前后肾功能监测指标变化情况

组别	n	血清肌酐（μmol/L）		肌酐清除率(mL/min)		尿微量蛋白(mg/L)	
		治疗前	治疗后	治疗前	治疗后	治疗前	治疗后
A组	143	102.1±1.3	94.2±2.5	81±5	83±7	236±75	201±87
C组	143	101.3±2.4	95.4±2.4	80±7	82±6	234±76	199±88

注：治疗后两组肾功能比较差异无统计学意义，P[illegible]0.05

3 讨　论

近年，我国原发性高血压患病率逐年上升。高血压长时间持续会导致全身小动脉硬化，重要脏器供血不足，尤其以肾脏最显著，可以导致肾脏缺血缺氧，从而引起肾小球屏障功能障碍而出现蛋白尿。如何在有效控制血压的同时阻止肾脏病变进程引起医学界的关注。

贝尼地平是一种钙离子拮抗剂，可以阻滞不同亚型的钙离子通道，通过抑制血管细胞膜上Ca^{2+}内流，从而扩张周围血管，达到降压的效果，此外，据L型通道位于肾小球部位具有特征性，对于肾小球入球小动脉的扩张作用大于出球小动脉，在不升高肾小球滤过率的前提下，使肾脏灌注增多，改善缺氧缺血状态，从而保护肾功能[1]。缬沙坦作为一种血管紧张素Ⅱ（AngⅡ）受体拮抗剂，可以选择性地作用于AT_1受体，进而阻断AngⅡ与AT_1受体结合，减少AngⅡ介导的生理效应[2]，抑制血管收缩和醛固酮释放，不仅可以达到降压的目的，

© 1994-2013 China Academic Journal Electronic Publishing House. All rights reserved.　http://www.cnki.net

还可以通过扩张包括肾脏出球小动脉在内的全身小动脉而降低肾脏内压，降低肾小球基底膜通透性，减少蛋白尿排泄，进而延缓肾小球硬化达到保护肾脏的目的[3]。我院分别采用缬沙坦及与贝尼地平治疗原发性高血压伴蛋白尿患者，从结果可以得出：缬沙坦及贝尼地平治疗高血压疗效相当，并可以改善肾功能。

参考文献

[1] 彭涛,胡昭,郭玲,等.贝尼地平和缬沙坦对原发性高血压伴蛋白尿患者肾脏保护作用的对比[J].中华心血管病杂志,2010,38(1):20-22.
[2] 卢宏.缬沙坦治疗原发性高血压临床观察[J].中国医药指南,2008,6(9):5-6.
[3] 陈刚.缬沙坦对降血压和保护肾脏的作用研究[J].中国基层医疗,2008,15(5):776-777.

高渗复合盐治疗急性大面积脑梗死的临床研究

陈 专
（河南省南阳油田总医院神经内科，河南 南阳 473132）

【摘要】目的 探讨高渗复合盐治疗急性大面积脑梗死临床应用。**方法** 2008 年 3 月至 2011 年 3 月收治的急性大面积脑梗死患者 65 例依据治疗方法不同分为甘露醇组 35 例、高渗复合盐组 30 例、进行颅内压监测。**结果** 两组病例降低颅内压起效时间、作用时间、以及治疗前后颅内压降低幅度、比较 $P > 0.05$、无显著差异性；甘露醇组血浆 BUN 显著升高有显著差异性。**结论** 高渗复合盐治疗急性大面积脑梗死降低颅内压显著、作用和缓、改善平均动脉压、改善脑灌注压、降低神经细胞凋亡、可以作为降低颅内压的一种方法。

【关键词】 高渗复合盐；急性大面积脑梗死；临床研究
中图分类号：R743.33 **文献标识码**：B **文章编号**：1671-8194（2012）27-0202-02

急性大面积脑梗死是临床常见的急危重症，由于梗死面积较大，引起脑水肿，使得颅内压显著升高，导致严重的高颅压[1]，严重者出现脑疝，是急性大面积脑梗死死亡的主要原因。针对急性大面积脑梗死形成的高颅压，常用的高渗脱水疗法是应用甘露醇，由于大量的甘露醇应用可以导致低脑灌注压、急性肾功能衰竭等严重的不良反应，近年来应用高渗复合盐进行降颅压，具有显著的降低颅内压、和保护脑细胞、改善脑血流作用，已被广泛认识，我们就高渗复合盐在急性大面积脑梗死治疗中应用进行总结，现汇报如下。

1 资料与方法

1.1 临床资料

选取2008年3月至2011年3月收治的急性大面积脑梗死患者65例，其中男性45例，女性20例，年龄49~78岁，平均年龄68.4岁。所有病例均经头颅CT、MRI影像学检查明确诊断为大面积脑梗死。

1.2 方法

1.2.1 分组

将65例急性大面积脑梗死患者依据治疗方法不同分为甘露醇组35例，高渗复合盐组30例，两组病例在年龄、性别、疾病程度上无差异，临床具有可比性。

1.2.2 治疗方法

高渗复合盐组：在积极治疗原发急性大面积脑梗死同时应用高渗复合盐进行降颅内压，采取4mL/kg 7.5%高渗盐水联合10%右旋糖酐500mL每日2次静脉滴注。

甘露醇组：采用5mL/kg，20%甘露醇每日2次静脉滴注。

1.2.3 观察内容

应用410型无创颅内压监测仪（重庆海威康医疗仪器有限公司生产）观察两组病例降低颅内压起效时间、作用时间，以及治疗前后颅内压降低幅度，并进行比较。观察两组病例与治疗前、治疗48h后进行血钠、血氯、血钾、血浆渗透压、BUN检测并进行比较。

1.2.4 统计学方法

使用统计软件SPSS10.0进行分析，($\bar{x} \pm s$)表示计量资料，组间比较采用t检验进行数据处理。

2 结 果

2.1 颅内压监测仪

观察两组病例降低颅内压起效时间、作用时间，以及治疗前后颅内压降低幅度，并进行比较，具体见表 1 。

表1 两组颅内压监测($\bar{x} \pm s$)

组别	例	起效时间(min)	作用时间(min)	颅内压降低幅度(mmHg)
甘露醇组	35	21.5±4.8	230±31	8.9±2.1
高渗复合盐组	30	19.4±5.2	270±46	9.1±1.8

注：经统计学分析、两组颅内压监测内容比较$P>0.05$、无显著差异性

2.2 观察内容

观察两组病例与治疗前、治疗48 h后进行血钠、血氯、血钾、血浆渗透压、BUN检测并进行比较，具体见表2。

表2 两组观察内容统计并比较($\bar{x} \pm s$)

高渗复合盐组	甘露醇组		观察内容	
	治疗前	治疗后	治疗前	治疗后
血钠浓度（mmol/L）	140.3±3.8	144.6±4.2	141.1±4.1	138.3±3.9
血氯浓度（mmol/L）	101.2±4.3	106.7±5.1	101.6±3.9	100.5±2.2
血钾浓度（mmol/L）	3.8±0.1	3.7±0.2	3.9±0.3	3.6±0.3
血浆渗透压（mOsm/L）	298.7±6.8	300.4±6.1	330.1±6.5	298.7±5.5
BUN（mmol/L）	7.0±3.3	6.4±2.7#	6.9±3.1	9.7±4.0#

注：经统计学分析、两组病例治疗前观察内容比较$P>0.05$、无显著差异性。#两组治疗后比较$P<0.05$、有显著差异性

3 讨 论

急性大面积脑梗死在临床上是指梗死波及两个脑叶以上，梗死范围直径在4.0cm以上，称为大面积脑梗死[2]；由于脑梗死后梗死区出现严重的缺氧性损伤，使得大量的脑细胞出现变性细胞内肿胀及坏死，从而出现细胞毒性水肿，随后梗死病程导致血管通透性改变出现血管源性水肿，导致双相水肿，引起颅内压升高，甚至引起脑疝，对脑组织的损伤加重，控制颅内压升高和脑水肿是治疗大面积脑梗死非常重要的措施。

降低颅内压目前常用的药物主要有甘露醇、甘油果糖、速尿、白蛋白等，最长用的是甘露醇，由于大量的甘露醇应用可以导致低脑灌

© 1994-2013 China Academic Journal Electronic Publishing House. All rights reserved. http://www.cnki.net
