# Supplementary material for: Effects of T-Type Calcium Channel Blockers on Renal Function and Aldosterone in Patients with Hypertension: A Systematic Review and Meta-Analysis
Source: PLoS One. 2014 Oct 17;9(10):e109834. doi: 10.1371/journal.pone.0109834 (PMC4201480; doi:10.1371/journal.pone.0109834)
Supplement: File S3 — PDF files of twenty-four studies included in the meta-analysis. (ZIP) [file pone.0109834.s007.zip › Supporting information-PDF files/32. Journal of China Modern Medicine 2011[13(11)]54-56.pdf]

贝尼地平和培哚普利对老年高血压病伴蛋白尿患者肾功能的影响

董波　屈晓冰

【摘要】 目的　比较新型钙通道阻滞剂贝尼地平与血管紧张素转换酶抑制剂培哚普利对老年高血压病伴蛋白尿患者肾脏保护作用的差别。方法　将 60 例 24h 尿蛋白总量<1g 的老年高血压病患者，随机分为贝尼地平组(A 组)与培哚普利组(B 组)，分别给予贝尼地平与培哚普利治疗 12 月，比较 A、B 两组治疗前后收缩压(SBP)，舒张压(DBP)，24h 尿蛋白(Pro)、血清肌酐(Scr)、肾小球滤过率(GFR)的差别。结果　①治疗前 A、B 两组 SBP、DBP、Pro、Scr、GFR 无显著性差异(P>0.05)；②A、B 两组治疗后 SBP、DBP、Scr 较治疗前有显著下降，GFR 均明显升高(P<0.05)；③治疗后 AB 两组间 SBP、DBP、Scr、GFR 无明显差异(P>0.05)。结论　贝尼地平与培哚普利比较，对老年高血压伴蛋白尿的患者，具有相似的肾脏保护作用。

【关键词】 贝尼地平　培哚普利　老年高血压　蛋白尿　肾小球滤过率

Renal function effects of benidipine and perindopril in elderly hypertensive patients with proteinuria *Dong Bo, Qu Xiaobing. Ma Wangdui Hospital of Hunan Province, Changsha* 410016

【Abstract】 Objective To compare the difference of the new type of calcium channel blockers benidipine and angiotensin-converting enzyme inhibitor perindopril in elderly hypertensive patients with proteinuria in patients with renal protective effect. **Methods** The 24h urine total protein <1g of 60 elderly patients with hypertension were randomly assigned benidipine group (A guoup) and perindopril group (B group) were treated with benidipine or perindopril for 12 months, systolic blood pressure (SBP), diastolic blood pressure (DBP), 24h urine protein (Pro), serum creatinine (Scr), glomerular filtration rate (GFR) of both A and B groups were measured at baseline,12 months. **Results** ①SBP, DBP, Pro, Scr, GFR were no significant difference in both pre-treatment and treated groups (P>0.05). ②SBP, DBP, Scr were significantly decreased in patients treated with benidipine or perindopril compared to pre-treatment patients, GFR was significantly higher (P<0.05). ③SBP, DBP, Scr, GFR were no significant difference in patients treated with benidipine compared to the patients treated with perindopril (P>0.05). **Conclusion** The protection efficiency of perindopril and benidipine is similar in elderly hypertensive patients with proteinuria.

【Key words】 Benidipine　Perindopril　Elderly hypertension　Proteinuria　Glomerular filtration rate

随着人口老龄化，我国逐渐进入老年性社会，据 2002 年卫生部组织的全国居民 27 万人营养与健康状况调查资料显示，我国 60 岁以上人群高血压的患病率为 49%，且老年高血压常与多种疾病并存，并发症多，常并发冠心病、脑血管疾病、肾功能不全等[1]。血管紧张素转换酶抑制剂(ACEI)已被国内外众多临床研究证实，对高血压伴蛋白尿的患者肾功能有保护作用，本研究分析 60 例分别使用新型钙通道阻滞剂（CCB）贝尼地平与血管紧张素转换酶抑制剂(ACEI)培哚普利老年高血压伴蛋白尿患者的治疗，观察贝尼地平对老年高血压伴蛋白尿患者的肾功能保护作用。

1　材料与方法

1.1 对象　我院心血管内科或老干科 2009 年 6 月至 2010 年 6 月收治的老年高血压伴蛋白尿患者 60 例。入选者符合以下条件：①年龄在 65 岁或以上；②符合中国高血压防治指南(2005 年版)的高血压诊断标准，且伴有蛋白尿；③24h 尿蛋白数量小于 1g；④无糖尿病、肝功能异常；⑤血脂在正常范围之内或经治疗血脂波动在正常范围之内。

1.2 研究方法　①分组：60 例老年高血压伴蛋白尿患者随机分为贝尼地平组(A 组)，贝尼地平为麒麟鲲鹏(中国)生物药业有限公司生产；培哚普利组(B 组)，培哚普利为法国施维雅制药有限公司生产。每

作者单位：410016　湖南长沙，湖南省马王堆医院(董波)；中南大学湘雅二医院(屈晓冰)

组各 30 例。②方法：A 组使用贝尼地平 4mg/d 开始治疗，血压控制不达标，2 周后增至 8mg/d；B 组使用培哚普利 4mg/d 开始治疗，血压控制不达标，2 周后增至 8mg/d，两组均治疗 12 个月，血压控制标准为≤130/80mmHg。

1.3 观察指标 ①血压测量：均按中国高血压防治指南(2005 年版)推荐的方法测量血压；②尿蛋白测量：留取第 1 天上午 7 时至第 2 天上午 7 时共 24h 的尿液测量尿蛋白(Pro)[2]；③肾小球滤过率(GFR)由中国改良 MDRD 公式计算得出，GFR (ml·min^{-1}·1.73m^{-2})；④血清肌酐(Scr)采用日立 7180 自动分析仪检测。以上观察指标均测量治疗前或治疗 12 个月后的两个时间点的值。

1.4 统计学方法 采用 SPSS16.0 软件进行数据处理，计数资料用 χ^2 检验，计量资料均以 $\bar{x}\pm s$ 表示，组间比较采用 t 检验。$P<0.05$ 为差异有统计学意义。

2 结果

2.1 A、B 两组治疗前在性别、年龄、血压、血清肌酐、尿蛋白(Pro)方面差异无统计学意见($P>0.05$)，见表 1。

表 1 一般情况比较

组别	例数	年龄（岁）	性别（男/女）	SBP (mmHg)	DBP (mmHg)	Scr	Pro
A	30	68.9±3.7	19/11	168.5±9.1	105.1±7.3	110.4±22.5	0.58±0.95
B	30	69.3±3.5	18/12	168.3±9.2	105.3±7.1	110.8±22.7	0.60±0.21

2.2 A、B 两组治疗前后血压、Scr、Pro 均有明显下降，GFR 均有明显上升，具有统计学差异($P<0.05$)；两组间治疗后比较血压、Scr、Pro、GFR 无显著性差异($P>0.05$)，见表 2。

表 2 A、B 两组治疗前后各观察指标变化

组别	SBP(mmHg)	DBP(mmHg)	Scr(μmol/L)	Pro(g/24h)	GFR(ml·min^{-1}·1.73m^{-2})
A 组治疗前	168.5±9.1	105.1±7.3	110.4±22.5	0.58±0.15	51.4±0.80
A 组治疗后	121.5±6.8^{ab}	75.1±4.2^{ab}	97.4±20.4^{ab}	0.39±0.55^{ab}	66.9±11.1^{ab}
B 组治疗前	168.3±9.2	105.3±7.1	110.8±22.7	0.60±0.21	50.8±9.5
B 组治疗后	122.1±6.4^{ab}	75.3±4.0^{ab}	97.6±20.3^{ab}	0.40±0.07^{ab}	67.1±10.2^{ab}

注：各组治疗前后比较 $^aP<0.05$，治疗后组间比较 $^bP>0.05$

3 讨论

长期高血压可导致心、脑、肾损伤[3]，国内外研究表明，高血压患者的风险不仅取决于血压水平，还取决于患者的并发症、合并症以及其他心血管危险因素[4]，高血压病治疗不单纯是降低血压，更重要的是保护靶器官，减少或延缓并发症发生。本研究表明贝尼地平与培哚普利均能有效控制血压，减少尿蛋白，提高肾小球滤过率，改善肾功能。这与以往的研究是一致的，也再次证实了严格控制血压是延缓肾脏病变的进展，预防心血管事件发生风险的关键[1]。其次，本研究表明贝尼地平在减少尿蛋白，提高肾小球滤过率方面，与培哚普利比较有类似作用，而 ACEI 或 ARB 既有降压，又有减少尿蛋白的作用，对于高血压伴肾脏病患者，尤其有蛋白尿患者，降压治疗的首选药物，已是不争的事实，而既往其它钙离子阻滞剂却没有被证实有类似作用。可能与以下几个原因有关：①贝尼地平是 T 亚型钙通道阻滞剂，而传统的 DHP 类钙通道阻滞剂通过分布在肾小球入球小动脉上的 L 亚型钙通道扩张入球小动脉，但对分布在出球小动脉上的 T 亚型钙通道没有阻滞作用，所以不能扩张出球小动脉，导致肾小球囊内压增高，部分抵消改善肾小球血流的作用，而贝尼地平具有 T 亚型钙通道的阻滞作用，能均衡地扩张入球小动脉与出球小动脉，使肾小球囊内压降低[5]，减少尿蛋白排泄，提高肾小球滤过率。②有证据表明，贝尼地平不仅对 T 亚型钙通道阻滞，还具有多方面的非血流动力学作用，例如，调节核因子-kappaB 的活性抑制炎性反应的发生，促进醛固酮的分泌释放，改善肾组织重构，从而抗氧化抗增殖效应；Suganol 等发现在肾脏模型选择 T 通道阻滞通过抑制 ROCK 活性，改善肾间质纤维化和肾小管上皮细胞转分化，从而延缓肾脏病变。③老年高血压具有单纯收缩压增高为主，脉压增大，血压波动大，晨峰血压增高，并发症多等特点，而贝尼地平具有独特“Membrane Approach”药理特性，降压平稳持久，适合老年高血压的特点，减少因血压波动对血管壁的损伤，从而也改善了肾功能。

总之，贝尼地平是一种新型钙通道阻滞剂，与其它 DHP 类的钙通道阻滞剂不同，具有培哚普利等(ACEI 类)药物同等降压效果及肾脏保护作用，尤其适合老年高血压伴蛋白尿患者的降压治疗。

参 考 文 献

1 中国高血压防治指南修订委员会. 中国高血压防治指南 2010. 中华心血管病杂志，2011，39(7)：579-615

2 Keane WF, Eknoyan G. Proteinuria, albuminuria,risk assessment,

detection, elimination (PARADE):a position paper of the national kidney foundation Am[J].Kidney Dis,1999,33:1004-1010
3 叶任高.内科学.北京:人民卫生出版社,2003:247-255
4 Zamorano J,Erdine S, Pavia A, et al. Proactive multiple cardiovascular risk factor management compared with,usual care in patients with hypertension and additional risk factors;the CRUCIAL trial. Curr Med Res Opin, 2011, 27:821-833
5 Suganol N, Wakinol S,Kandal T, et al. T-type calcium channel blockade as a therapeutic strategy against renal injury in rats with subtotal,nephrectomy. Kidney Int,2008,73:826-834

(收稿:2011-09-22)

医学论文选题的原则

1. 需求原则 这是选题的首要原则。科研工作要解决的基本问题不是空中楼阁,而是医学实践中所遇到的最基本问题,如直接威胁人类健康、死亡率高的心、脑血管疾病,肿瘤与糖尿病的治疗手段与预防措施。掌握疾病发生规律的医学基础研究如幽门螺杆菌的研究揭示了胃、十二指肠溃疡发病的过程及原因。不论是基础研究还是临床研究,都是医学实践助根本需求。为了适应我国国情,要把有限的科研资金投入到最迫切需要解决的医学课题中去。为此,基础医学研究应致力于将理论与实践相结合,从事对临床医学工作具有指导意义的科学研究工作。

2. 创新原则 创新是科学研究的固有特性,创新意味着前所未有的开创性工作,如孟德尔发现遗传定律、爱因斯坦发现相对论,都是前所未有的开创性工作,也可以说是世界公认的创新。但这不是大多数科学工作者所能达到的,医学研究也不应苛求前所未有。如果是在前人已有的基础上继续突破,无论在方法上、操作技术上和理论上及结果的解释上有所发现、有所前进,用目前已有的方法,在前人研究的基础上补充了新内容、新观点,发现了新规律;对观察方法和统计方法的改进;或在推理、逻辑思维、演绎、归纳等方法的改进都是创新,只要含有这些创新意义的选题就具有较高的学术价值与水平,就非"低能重复"。

3. 可行原则 这是评价科研选题的技术指标、实施方案、设备条件、经费要求等能否实观的可行性分析。

临床科研选题的可行性取决于下列条件:①是否有足够的样本——病人;②研究方法是否易被病人接受。③研究对象的具体条件,如有些研究对象必须住院期观察,直接影响到课题的可行性。

4. 协作原则 选题中应充分发挥多学科、多专业的相互配合,在经费、人力、物力使用上统筹安排,保证课题的顺利实施。医学课题在综合研究、综合协作方面更显重要,如有些疾病的病因不仅涉及病理、微生物等医学基础学科,而且涉及到环境、生物、地理、地质、水文等学科。

5. 最优化原则 即是指花最低的代价,取得最大效益的原则。

6. 其他原则 除上述原则之外,一般原则有:①要使题目尽可能具体而明确,不琐碎、不孤立。研究题目选得越具体、明确,说明研究者的思维越清楚;题目越集中,针对性越强;实验观察对象及使用的方法和观察指标之间的联系和因果关系越明确,结果的可信度越高。②要扬长避短,力求与自己研究的专业对口,充分发挥自己的特长,基础理论比较扎实而善于研究者,可侧重选择理论性课题;动手能力强、实践经验比较丰富者,可选择技术性课题。③要选择有发展前途的课题,如新学科、新理论的研究。这样,当一个课题题目被做过之后,还可能引出一些新课题。
