# Supplementary material for: Effects of T-Type Calcium Channel Blockers on Renal Function and Aldosterone in Patients with Hypertension: A Systematic Review and Meta-Analysis
Source: PLoS One. 2014 Oct 17;9(10):e109834. doi: 10.1371/journal.pone.0109834 (PMC4201480; doi:10.1371/journal.pone.0109834)
Supplement: File S3 — PDF files of twenty-four studies included in the meta-analysis. (ZIP) [file pone.0109834.s007.zip › Supporting information-PDF files/33. Arzneimittelforschung 2009[59(12)]647-650.pdf]

A Comparative Study of the Renoprotective Effects of Benidipine and Valsartan in Primary Hypertensive Patients with Proteinuria

For private or internal corporate use only

Tao Peng, Zhao Hu, Qing Xia, Bei Jiang, Xianhua Li, Xiangdong Yang

Department of Nephrology, Shandong University Qilu Hospital, Jinan (P. R. China)

Corresponding author: Xiangdong Yang, MD, Department of Nephrology, Shandong University Qilu Hospital, Jinan 250012 (P.R. China); e-mail: sesame_oil@126.com

Abstract

Objective: **To compare the renoprotective effects of the calcium channel blocker (CCB) benidipine (CAS 105979-17-7) and the angiotensin II receptor blocker (ARB) valsartan (CAS 137862-53-4) in primary hypertensive patients with proteinuria.**

Methods: **236 patients with primary hypertension were randomly divided into different groups and were administered either benidipine or valsartan. The alterations of the glomerular filtration rate (GFR) and proteinuria were compared between the different groups.**

Results: **Valsartan could decrease the level of proteinuria significantly as compared with that in benidipine-treated hypertensive patients with proteinuria at levels < 1 g/24 h (P < 0.01). There was no significant difference of the effects of benidipine and valsartan on proteinuria reduction in hypertensive patients with proteinuria at levels 1–3 g/24 h. There was no significant difference of the effects of benidipine and valsartan on GFR in benidipine- and valsartan-treated patients.**

Conclusion: **The results showed that valsartan was more effective in decreasing the levels of proteinuria in hypertensive patients with proteinuria at an early stage of nephropathy. The renoprotective effects of benidipine and valsartan in primary hypertensive patients with proteinuria were similar.**

Key words

- **Benidipine**
- **CAS 105979-17-7**
- **CAS 137862-53-4**
- **Primary hypertension**
- **Renoprotection**
- **Valsartan**

Arzneimittelforschung 2009;59(12):647–650

1. Introduction

At present, hypertension is increasing in prevalence worldwide. Most hypertensive patients have primary hypertension, only 10–15 % of them have seconary hypertension. Hypertension contributes to the damaging of many organs' function, including kidney. There is 7-fold higher likelihood for patients with primary hypertension of developing renal damage as compared to age-matched control subjects. With disease progress, primary hypertensive patients tend to develop proteinuria, which is an early indicator of renal damage. At present, most hypertensive patients are treated with multiple anti-hypertensive drugs. Many studies have addressed the protective effects of angiotensin II receptor blockers (ARBs) against the development of nephropathy in patients with hypertension; however, very few studies discussed the renoprotective effects of calcium channel blockers (CCBs) on patients with primary hypertension. Some studies in rats [1, 2] indicated that the renoprotective effects of CCBs were similar to those of ARBs. However, only a few studies [3] compared the difference on the renoprotective effects between CCBs and ARBs in hypertensive patients with proteinuria. In the present study, we investigated the renoprotective effects of the CCB benidipine (CAS 105979-17-7) and the ARB valsartan (CAS 137862-53-4) in primary hypertensive patients with proteinuria.

© ECV · Editio Cantor Verlag, Aulendorf (Germany)

For private or internal corporate use only

2. Materials and methods

2.1 Materials

In the present study, 236 cases (122 males, 114 females; mean age: 43.2 ± 9.5 years) were enrolled and examined at the Department of Nephrology, Shandong University Qilu Hospital, Shandong Province, China, between March 2006 and July 2007. Patients enrolled in this study had primary hypertension [4] and overt proteinuria (> 150 mg/24 h), with systolic and diastolic blood pressures ranging between 120–160 mmHg and 80–100 mmHg [4], respectively, and a glomerular filtration rate (GFR) of 30–90 ml/min/1.73 m^2. All enrolled subjects were without signs of diabetes mellitus, liver dysfunction, or dyslipoproteinemia. The study protocol was approved by the ethics committee of the hospital, and written informed consent was obtained from all patients after they had reviewed a written summary of the study plan.

2.2 Groups and drug administration

All subjects were divided into 2 groups according to the levels of proteinuria per 24 h: group A, patients with levels between 1 g/24 h and 3 g/24 h (n = 120 cases), and group B, patients with proteinuria < 1 g/24 h (n = 116). Patients in each group were assigned to either the benidipine subgroup (A1, B1) or the valsartan subgroup (A2, B2). No significant differences were noted among each group and subgroup in terms of sex, age, and blood pressure levels. All patients were administered 8 mg of benidipine (batch number 932ADG) per day or 80 mg of valsartan (batch number X0042) per day after breakfast, according to their respective subgroup. During the study period, the dosage of benidipine or valsartan was adjusted to achieve the targeted systolic (100–130 mmHg) and diastolic blood pressure (65–80 mmHg) [5] levels. The mean dose of bendipine was 10.6 mg/day, and that of valsartan was 137.2 mg/day. As a rule, the type and dosage of concomitant medication were not altered during the study period. The use of angiotensin-converting enzyme inhibitors and other ARBs or CCBs was avoided. The study period was 12 months. Both products (benidipine and valsartan) were obtained from the respective manufacturers.

2.3 Measurements

As per standard guidelines, the blood pressure was measured [6] at baseline and at 3, 6, and 12 months after the initiation of the study. Serum creatinine was measured before the study at baseline and at 3, 6, and 12 months after the study was initiated. GFR was calculated by using the Modification of Diet in Renal Disease (MDRD) [7]. Chronic kidney disease was staged on the basis of the GFR [8]. Protein excretion was measured [9] over 24 h at baseline and at 3, 6, and 12 months after the start of the study, as per established methods. In addition, the height and weight of the patients were recorded at the above-mentioned time points.

2.4 Statistical analysis

Data were expressed as mean ± SD. Statistical analysis was evaluated using SPSS for Windows 11.0 software, followed by the Student's t-test to assess the significance of the differences among the various groups. Values with P < 0.05 were considered significant.

3. Results

In all the groups, no significant difference was noted from the baseline values of GFR among subjects in the same groups/subgroups after 12 weeks. However, after 24 weeks, a significant difference (P < 0.01) from the baseline GFR was noted among subjects within the same group, while no difference was noted between the GFR values for subjects in different groups (Table 1).

There was a significant difference in the level of proteinuria from the baseline in groups A and B, but there was no difference at the intergroup level (A1 and A2 vs. B1 and B2). After 12 weeks, no significant differences were noted in the levels of proteinuria between either A1 and A2 or between B1 and B2; however, after 24 weeks, especially after 48 weeks, a significant difference (P < 0.01) was observed in these cases. In group A, after 24 and 48 weeks, the levels of proteinuria were lower for patients who were administered ARB than those who were administered CCB in the same group, but the difference was not significant. In group B, the levels of proteinuria were significantly lower (P < 0.01) for patients administered ARB than those administered CCB (Table 2).

After 12 weeks, the systolic and diastolic blood pressure levels decreased significantly in all the patients in the two groups. The targeted systolic blood pressure (100 ~ 130 mmHg) and diastolic blood pressure (65–80 mmHg) levels were achieved after 24 weeks, and significant differences (P < 0.01) from the baseline were noted. Once the targeted blood pressure levels were achieved, further administration of ARB or CCB did not have any further decreasing effect on the blood pressure levels (Tables 3, 4).

Table 1: Changes in the glomerular filtration rate during the study period (ml/min/1.73 m^2) ($\bar{x} \pm SD$).

Groups	n	Baseline	12 weeks	24 weeks	48 weeks
A1	59	51.4 ± 8.05	54.1 ± 9.10	60.6 ± 10.1**	66.9 ± 11.2**
A2	61	50.8 ± 9.32	53.9 ± 10.0	59.9 ± 9.89**	67.1 ± 10.3**
B1	59	49.7 ± 6.98	54.4 ± 8.25	61.1 ± 8.68**	67.5 ± 9.19**
B2	57	52.0 ± 7.39	54.9 ± 9.29	61.4 ± 9.78**	68.0 ± 11.0**

**p < 0.01 versus baseline.

Table 2: Changes in the level of proteinuria during the study period (g/24 h) ($\bar{x} \pm SD$).

Groups	n	Baseline	2 weeks	24 weeks	48 weeks
A1	59	2.01 ± 0.65	1.78 ± 0.09	1.24 ± 0.15##	1.19 ± 0.11##
A2	61	1.98 ± 0.71	1.72 ± 0.31	1.03 ± 0.08##	0.79 ± 0.13##
B1	59	0.59 ± 0.15	0.51 ± 0.26	0.39 ± 0.06##	0.30 ± 0.05##
B2	57	0.61 ± 0.21	0.49 ± 0.17	0.27 ± 0.07##**	0.18 ± 0.01##**

Difference between the levels in different groups at the same time points: **p < 0.01.
Difference between the levels in each group at different time points: ##p < 0.01.

Arzneimittelforschung 2009;59(12):647–650
© ECV · Editio Cantor Verlag, Aulendorf (Germany)

Table 3: Changes in the systolic blood pressure levels during the study period (mmHg) ($\bar{x} \pm SD$).

Groups	n	Baseline	12 weeks	24 weeks	48 weeks
A1	59	151.2 ± 17.2	141.1 ± 11.9*	128.2 ± 12.1**	126.32 ± 10.4**
A2	61	149.7 ± 14.9	140.1 ± 12.7*	127.9 ± 10.9**	125.7 ± 9.6**
B1	59	150.3 ± 16.0	140.9 ± 18.5*	126.9 ± 11.7**	127.4 ± 10.1**
B2	57	149.0 ± 12.9	139.8 ± 15.2*	127.6 ± 13.2**	128.0 ± 6.5**

*p < 0.05, **p < 0.01 versus baseline.

Table 4: Changes in the diastolic blood pressure levels during the study period (mmHg) ($\bar{x} \pm SD$).

Groups	n	Baseline	12 weeks	24 weeks	48 weeks
A1	59	95.2 ± 7.2	83.1 ± 5.6*	78.2 ± 7.1**	77.2 ± 6.5**
A2	61	94.8 ± 7.9	82.9 ± 7.1*	75.9 ± 4.9**	75.7 ± 9.3**
B1	59	96.1 ± 8.6	84.2 ± 8.7*	76.1 ± 5.5**	78.4 ± 6.1**
B2	57	97.0 ± 10.1	86.5 ± 5.2*	79.0 ± 6.9**	78.1 ± 5.9**

*p < 0.05, **p < 0.01 versus baseline.

4. Discussion

Primary hypertensive patients with a GFR of 30–90 ml/min/1.73 m^2 and proteinuria below 3 g/24 h were enrolled into the present study. The purpose of this study was to evaluate the renoprotective effects of antihypertensive drugs in patients with primary hypertension and proteinuria and, simultaneously, assess the difference between the effects of these two types of antihypertensive drugs.

The CCB benidipine is used for the treatment of hypertension. Benidipine has a relatively high vascular selectivity and is expected to exert protective effects on vascular endothelial cells. Renoprotective effects of benidipine also have been demonstrated in several basic and clinical studies [10–14]. Benidipine, a 1,4-dihydropyridine (DHP) derivative, is a long-acting dihydropyridine calcium channel blocker that targets single T-type Ca^{2+} channels in vascular smooth muscle cells [15]. Benidipine was developed in Japan; it exhibits a sustained hypotensive effect independent of the blood level [16], increases membrane fluidity, and improves the microviscosity of erythrocytes both *in vitro* and *in vivo* [17]. These effects are ascribed to its tendency to bind to the 1,4-dihydropyridine (DHP) receptor via a membrane approach [18] and dislodge from the receptor at a slow rate, unlike other calcium antagonists derived from DHP. Moreover, some reports have proved [19] that benidipine has the ability to cause equal distension of the glomerular afferent and efferent arterioles and that it is unable to cause an increase in the intraglomerular pressure [20, 21].

A DHP calcium channel blocker was used as an antihypertensive and renoprotective drug. After 12 weeks, no change was noted in the GFR values compared to baseline value, but a significant difference was noted after 24 weeks. A similar pattern of change was noted in the levels of proteinuria. The significant renoprotective effects in patients with hypertension and proteinuria are visible only when the drug is administered for more than 24 weeks (6 months). This finding is consistent with that of another report [22].

Valsartan is one of the angiotensin-II receptor antagonists that have been routinely used as antihypertensive drugs and for the prevention of damage to the kidneys, brain, and heart in patients with diabetic nephropathy, hypertension, cardiovascular diseases, etc. A decrease in the rate of the progression of nephropathy is reflected in a significant increase in the time to the doubling of the serum creatinine concentration, which is a measure that approximately reflects the halving in the glomerular filtration rate [23, 24].

This present study may indicate that the action of benidipine in patients with hypertension and proteinuria is similar to that of valsartan in decreasing the systolic and diastolic blood pressure levels. No significant difference was noted between the benidipine- and valsartan-treated groups in terms of the GFR. The beneficial effects of valsartan over benidipine in decreasing proteinuria in hypertensive patients with proteinuria at levels < 1 g/24 h were visible after 6 months of treatment with this drug ($P < 0.01$). However, for hypertensive patients with proteinuria at levels between 1 g and 3 g/24 h, there was no significant difference between the effects of benidipine and valsartan.

As explained above, benidipine has a mechanism of action completely different from that of valsartan in antihypertensive patients and renoprotection. In early stages (GFR 30–90 ml/min/1.73 m^2) of nephropathy, benidipine and valsartan exert similar effects on mitigating the progression of nephropathy. However, valsartan is more advantageous than benidipine in the case of patients with hypertension and proteinuria (baseline, < 1 g per day and night). The results of the present study may indicate that patients with hypertension and proteinuria should be administered ARBs in early stages of nephropathy for enhanced renoprotection.

Limitations

This study has some limitation, including the small sample size and the lack of blinding of the study investigators.

Acknowledgement

This work was supported by the Science and Technology Development Project of Shandong Province, China (No. 2000BB1CJB6).

Literature

[1] Matsuzaki G, Ishizaka N, Furuta K, Hongo M, Saito K, Sakurai R, *et al.* Comparison of vasculoprotective effects of benidipine and losartan in a rat model of metabolic syndrome. *Eur J Pharmacol.* 2008 Jun;587 (1–3):237–242.

For private or internal corporate use only

© ECV · Editio Cantor Verlag, Aulendorf (Germany)

[2] Takayama M, Arakawa E, Yao K, Ina Y, Sato H, Hawegawa K, *et al.* Effects of combination of angiotensin receptor blocker and calcium channel blocker on ox-LDL levels and cardiovascular dysfunction in Dahl rats. *Pharmacology.* 2006;77(4):179–187.

[3] Shikata C, Sekikawa T, Kimura N, Kojima A, Seki S, Oka H, *et al.* Beneficial effect of combination therapy with antihypertensive drugs in patients with hypertension. *Exp Clin Cardol.* 2007;12(1):33–36.

[4] The Joint National Committee on Prevevtion, Detection, Evaluation and Treatment of High Blood Pressure: The Sixth Report of the Joint National Committee on Prevevtion, Detection, Evaluation and Treatment of High Blood Pressure. *Arch Intern Med.* 1997;157:2413–46

[5] Mansia G, De Backer G, Dominiczak A, Cifkova R, Fagard R, Germano G, *et al.* European Society of Hypertension; European Society of Cardiology. 2007 ESH-ESC Guidelines for the management of arterial hypertension: the task force for the management of arterial hypertension of the European Society of Hypertension (ESH) and of the European Society of Cardiology (ESC). *Blood Press.* 2007;16(3):135–232.

[6] Liu Lisheng. Guidelines for the management of hypertension in china. *Clin J Hypertension.* 2005 Feb;12 (Suppl.):9–10.

[7] Levey AS, Bosch JP, Lewis JB, Greene T, Rogers N, Roth D. A more accurate method to estimate glomerular filtration rate from serum creatintine: A new prediction. Modification of Diet in Renal Disease Study Group. *Ann Intern Med.* 1999 Mar;130(2):461–70.

[8] National Kidney Foundation. K/DOQI Clinical Practice Guidelines for Chronic Kidney Diseases: Evaluation, Classification and Stratification. *Am J Kidney Dis.* 2002 Feb;39(2 suppl 1):S1–S266.

[9] Keane W, Eknoyan G. Proteinuria, albuminuria, risk, assessment, detection, elimination (PAPADE): A position paper of the National Kidney Foundation. *Am J Kidney Dis.* 1999 May;33(5):1004–10.

[10] Uzu T, Nishimura M, Fujii T, Sakaguchi M, Kanasaki M, Isshiki K, *et al.* Benidipine attenuates glomerular hypertension and reduces albuminuria in patients with metabolic syndrome. *Hypertens Res.* 2007 Feb;30(2):161–5.

[11] Akizuki O, Inayoshi A, Kitayama T, Yao K, Shirakura S, Sasaki K, *et al.* Blockade of T-type voltage-dependent Ca^{2+} channels by benidipine, a dihydropyridine calcium channel blocker, inhibits aldosterone production in human adrenocortical cell line NCI-H295R. *Eur J Pharmacol.* 2008 Apr;584 (2–3):424–34.

[12] Seino H, Miyaguchi S, Yamazaki T, Ota S, Yabe R, Suzuki S. Effect of benidipine hydrochloride, a long-acting T-type calcium channel blocker, on blood pressure and renal function in hypertensive patients with diabetes mellitus. Analysis after switching from cilnidipine to benidipine. *Arzneimittelforschung.* 2007;57(8):526–31.

[13] Saito F, Fujita H, Takahashi A, Ichiyama I, Harasawa S, Oiwa K, *et al.* Renoprotective effect and cost-effectiveness of using benidipine, a calcium channel blocker, to lower the dose of angiotensin receptor blocker in hypertensive patients with albuminuria. *Hypertens Res.* 2007 Jan;30(1):39–47.

[14] Hayashi K, Wakino S, Homma K, Sugano N, Saruta T. Pathophysiological significance of T-type Ca^{2+} channels: role of T-type Ca^{2+} channels in renal microcirculation. *J Pharmacol Sci.* 2005 Nov;99(3):221–7.

[15] Gotoh Y, Mruaki K, Imaizumi Y. Effects of benidipine, a 1,4-dihydropyridine derivative, on single L-type Ca channel currents in vascular smooth muscle. *Clin Prep.* 1992;26:3879–901.

[16] Shimada S, Nakajima Y, Yamamaoto K, Sawada Y, Iga T. Comparative pharmacodynamics of eight calcium channel blocking agents in Japanese essential hypertensive patients. Biol *Pharm Bull.* 1996 Mar;19(3):430–7.

[17] Tsuda K. Electron paramagnetic resonance investigation on modulatory effect of benidipine on membrane fluidity of erythrocytes in essential hypertension. *Heart Vessels.* 2008 Mar;23(2):134–9.

[18] Suzuki K, Imada T, Gao F, Ma H, Nagata T. Radioautographic study of benidipine hydrochloride. Localization in the mesenteric artery of spontaneously hypertensive rat. *Arzneimittelforschung.* 1994 Feb;44(2):129–33.

[19] Morikawa T, Okumura M, Konishi Y, Okada N, Imanishhi M. Effects of benidipine on glomerular hemohynamics and proteinuria in patients with nondiabetic nephropathy. *Hypertens Res.* 2002;25:571–6.

[20] Kimrua K. Effects of benidipine hydrochloride on renal arterioles. *Ther Res.* 1994;15:2237–40.

[21] Gashti CN, Bakris GL. The role of calcium antagonists in chronic kidney disease. Curr *Opin Nephrol Hypertens.* 2004 Mar;13(2):155–61.

[22] Inoue S, Tomino Y. Effcets of calcium antagonists in hypertensive patients with renal dyfunction: A protective, randomized, parallel trial comparing benidipine and nifedipine. Nephrology (Carlton). 2004 Oct;9(5):265–71.

[23] Parving HH, Lehnert H, Bröchner-Mortensen J, Gomis R, Andersen S, Arner P. Irbesartan in Patients with Type 2 Diabetes and Microalbuminuria Study Group. The effect of irbesartan on the development of diabetic nephropathy in patients with type 2 diabetes. *New Engl J Med.* 2001 Sep 20;345(12):870–8.

[24] Croom KF, Curran MP, Goa KL, Perry CM. Irbesartan: a review of its use in hypertension and in the management of diabetic nephropathy. *Drugs.* 2004;64(9):999–1028.

For private or internal corporate use only

© ECV · Editio Cantor Verlag, Aulendorf (Germany)

Copyright of Drug Research / Arzneimittel -- Forschung is the property of Editio Cantor Verlag fur Medizin und Naturwissenschaften and its content may not be copied or emailed to multiple sites or posted to a listserv without the copyright holder's express written permission. However, users may print, download, or email articles for individual use.
Any further use, especially the compilation of an archive or database for anything other than personal use is considered unauthorized use.
