# Supplementary material for: Effects of T-Type Calcium Channel Blockers on Renal Function and Aldosterone in Patients with Hypertension: A Systematic Review and Meta-Analysis
Source: PLoS One. 2014 Oct 17;9(10):e109834. doi: 10.1371/journal.pone.0109834 (PMC4201480; doi:10.1371/journal.pone.0109834)
Supplement: File S3 — PDF files of twenty-four studies included in the meta-analysis. (ZIP) [file pone.0109834.s007.zip › Supporting information-PDF files/34. J Nephrol 2004[17(2)]261-269.pdf]

J NEPHROL 2004; 17: 261-269

Efficacy and tolerability of manidipine in the treatment of hypertension in patients with non-diabetic chronic kidney disease without glomerular disease. Prospective, randomized, double-blind study of parallel groups in comparison with enalapril

Lucia Del Vecchio[1], Marco Pozzi[1], Antonio Salvetti[2], Giuseppe Maschio[3], Maurizio Fusaroli[4], Carlo Rovati[5], Francesco Antonucci[6], Carmelo Cascone[7], Flavio Scanferla[8], Vincenzo Panichi[9], Alessandra Sturani[4], Francesco Locatelli[1]

on behalf of the Manidipine Study Group

[1]Department of Nephrology and Dialysis, Alessandro Manzoni Hospital, Lecco
[2]Department of Internal Medicine, University of Pisa, Pisa
[3]Department of Nephrology, Maggiore Hospital, Verona
[4]Department of Nephrology and Dialysis, S. Maria delle Croci Hospital, Ravenna
[5]Nephrology and Dialysis Unit, S. Chiara Hospital, Trento
[6]Nephrology and Dialysis Unit, S. Maria del Prato Hospital, Feltre
[7]Nephrology and Dialysis Unit, Civile Hospital, Castelfranco Veneto
[8]Nephrology and Dialysis Unit, Umberto I Hospital, Mestre
[9]Department of Nephrology and Dialysis, S. Chiara Hospital, Pisa - Italy

ABSTRACT: *Background:* **Calcium channel blockers (CCBs) are effective blood pressure lowering agents, giving rise to a prevalent dilation of the afferent arteriole. Manidipine, a long-lasting dihydropyridine CCB, demonstrates its action not only on the afferent arteriole, but also on the efferent one. This suggests theoretically a renoprotective effect in patients with chronic kidney diseases (CKD).**
Methods: **This was a multicenter, prospective, randomized, double-blind, parallel group study, to evaluate the efficacy and tolerability of manidipine (M; 10-20 mg/day), in comparison with enalapril (E; 10-20 mg/day) in the treatment of hypertension in 136 patients with CKD secondary to primary renoparenchymal disease. Changes in blood pressure values from baseline were considered as the primary outcome of the study. Proteinuria changes and the rate of renal function decline were also evaluated.**
Results: **During a 48-week follow-up, mean SBP decreased from 155 ± 11.7 to 138.7 ± 13.9 mmHg in M and from 157.3 ± 11.8 to 134.2 ± 13.9 mmHg in E; mean DBP decreased from 100.3 ± 4.2 to 86.1 ± 6.5 mmHg in M and from 100.3 ± 4.2 to 84.7 ± 6.3 mmHg in E. Proteinuria remained unchanged in M (from 1.6 ± 1.59 to 1.62 ± 1.79 g/24h), and decreased significantly in E (from 1.37 ± 1.45 g/24h to 1 ± 1.55 g/24h). No significant difference was observed in the rate of renal function decline in the two groups.**
Conclusions: **Manidipine was safe and effective, obtaining a significant reduction in SBP and DBP from baseline. Although patients treated with enalapril showed a better antiproteinuric response, the two treatments were equally effective in reducing the rate of CRF progression in patients without glomerular disease.**

Key words: *Manidipine, Enalapril, Chronic kidney disease, Proteinuria, Hypertension, Progression*

INTRODUCTION

Hypertension is a leading cause of end-stage renal disease (ESRD) worldwide and, together with proteinuria, is probably one of the main factors contributing to progression of chronic kidney disease (CKD); the reduction of blood pressure values with antihypertensive therapy has been shown to be an effective means of slowing down the progression towards ESRD, no matter what the cause (1). Moreover, hypertension is a significant determinant of morbidity and mortality among hemodialysis patients (2) (it is well known that cardiovascular disease accounts for more than 50% of the deaths of hemodialysis patients). For this reason, effective antihypertensive therapy is the single most important treatment in these patients.

However, not all antihypertensive agents are equally effective in slowing the rate of CKD progression, and some may have additional renoprotective effects. The results of several large clinical trials have shown that the inhibition of the renin-angiotensin system (RAS) by means of ACE inhibitors (ACE-I) or angiotensin II receptor 1 antagonists (ARBs) can reduce the rate of loss of renal function in diabetic (3) and non-diabetic CKD (4-6); this effect is greatest in patients with substantial proteinuria at baseline (4-6). Calcium channel blockers (CCBs) are effective vasodilators and blood pressure lowering agents, and have been extensively used in CKD patients. CCBs also have various properties that might afford renal protection, including their ability to delay renal growth (7) and attenuate the mitogenic effect of a number of cytokines and growth factors that use changes in intracellular free Ca^{++} for signal transduction (8). Given their action of prevalent dilation of the afferent arteriole, it has also been suggested that these agents could be especially useful in patients with ischemic nephropathies. However, despite many theoretical and experimental attributes, the clinical evidence for the renoprotective efficacy of CCBs has been less consistent.

In experimental studies, manidipine, a long-lasting dihydropyridine (dh) CCB, demonstrates its action not only on the afferent arteriole, as other dhCCBs, but also on the efferent one (9). Manidipine also has a direct natriuretic effect, indicating a direct renal tubular effect (9).

In the present study, we evaluated the efficacy and tolerability of manidipine in the treatment of hypertension in patients with CKD induced by primary renoparenchymal disease. Given that ACE inhibitors could be more effective in reducing proteinuria than CCBs, we decided to test this agent in patients without primary glomerular disease and severe proteinuria. Primary aim was to obtain a reduction of systolic blood pressure (SBP) to <140 mmHg and of diastolic blood pressure (DBP) to <90 mmHg. Secondary aim was to evaluate the rate of decline in renal function and proteinuria reduction in these patients when treated with manidipine or enalapril.

PATIENTS AND METHODS

Patients

Subjects of both sexes, aged 18-70 years, with CKD induced by primary renoparenchymal disease (hypertensive kidney disease, interstitial nephropathy, nephropathy from unknown causes) were eligible for the study if they met the following entry criteria: (i) renal insufficiency duration of at least 6 months before entering the study with creatinine clearance between 20-60 mL/min and/or serum creatinine between 1.5-3.0 mg/dL and with variation of <30% during the previous 6 months; (ii) hypertension with DBP between 95-110 mmHg and SBP between 140-200 mmHg; (iii) in anti-hypertensive treatment with agents that can be stopped and substituted by protocol drugs (including ACE inhibitors). Patients with SBP/DBP > 200/110 mmHg were excluded from the trial. In addition, patients with acute renal diseases, nephropathy secondary to diabetes or other systemic diseases, polycystic kidney disease, proteinuria > 3 g/day were excluded. Other exclusion criteria were: previous stroke or myocardial infarction, heart failure, aortic or mitralic valve stenosis, severe arrhythmias, known severe atherosclerotic disease, obesity, significant liver disease, hypersensitivity or any contraindication to the use of CCBs, ACEI, furosemide and beta-blockers; contemporaneous treatment with agents, which are known to alter serum creatinine determination (metildopa, ascorbic acid, cefoxitine, trimetropim or cimetidine). Women with child-bearing potential were required to take adequate contraceptive measures and were excluded if pregnant or breast-feeding.

Figure 1 summarizes the number of participants recruited, randomized, and followed up.

Sample size calculation

Assuming a minimum difference of 3 mmHg in mean changes of DBP between the two groups as statistically significant and estimating a standard deviation of the changes of 5 mmHg, with a power equal to 90% and a type I error of 0.05 (two-tail), a sample size of 59 evaluable patients for each group was required. Considering less than 20% of the patients to be not evaluable for efficacy analysis, it was decided to enrol 150 patients.

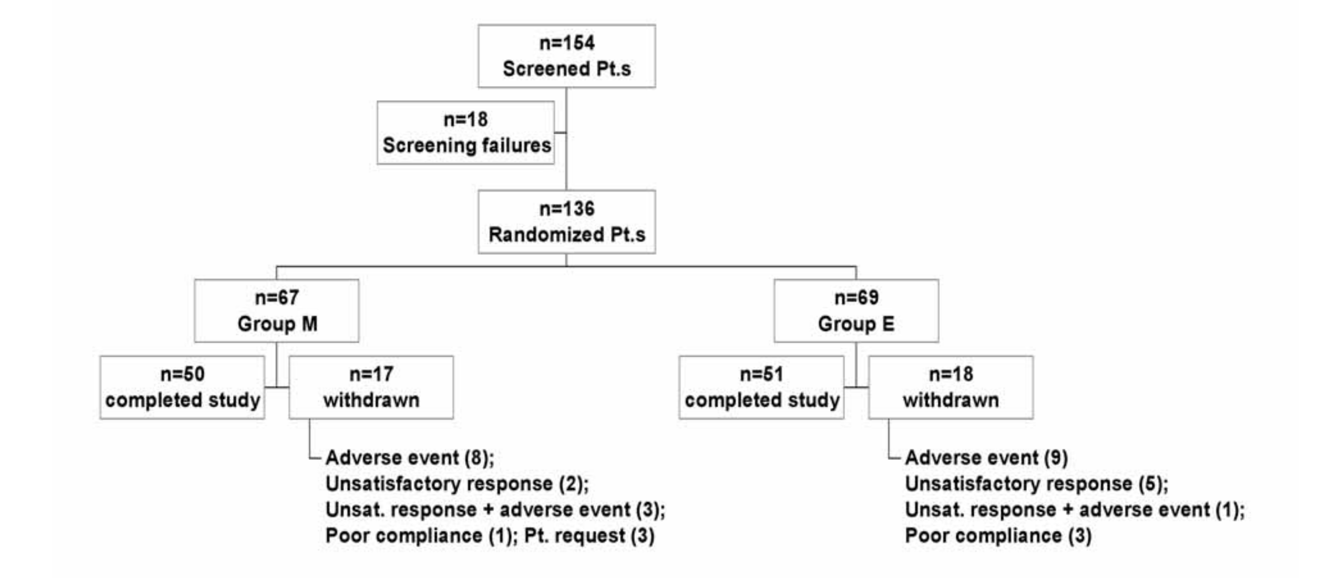

Fig. 1 - Participant recruitment and follow-up flow diagram.

Study design

This was a multicenter, prospective, randomized, double-blind, parallel group study. The protocol was approved by the local ethic committees of each hospital; all the patients gave their written informed consent to participate.
Eligible patients entered a 2 week run-in phase during which all the antihypertensive drugs were withdrawn and placebo was administered. At the first visit, a complete medical history, physical examination and ECG were carried out. Heart rate, sitting blood pressure and body weight were measured. Serum creatinine, creatinine clearance and urinary protein excretion on the basis of 24-h urine collection were planned for the following day.
After completing the run-in phase, patients who fulfilled the entry criteria were randomly assigned to receive either manidipine 10 mg or enalapril 10 mg once daily in the morning. The post-randomization phase lasted 48 weeks with clinical controls every 4 weeks. At each visit, heart rate, sitting blood pressure and body weight were measured. Concomitant treatments and adverse reactions were assessed by spontaneous reporting and on clinical and laboratory basis. Hematological, biochemical and urinary parameters were measured after 12 (visit 5), 24 (visit 7), 36 (visit 9) and 48 (visit 11) weeks.
After 4, 8, 12 weeks (visit 3, 4, 5), antihypertensive therapy was adjusted, as necessary, in order to obtain a DBP < 90 mmHg. After four weeks, the dose of manidipine or enalapril was increased to 20 mg once daily if DBP was above this target. After additional four weeks, if DBP was still above the target level, metoprolol retard (200 mg once a day) was added. If after four more weeks (week 12) DBP was still > 90 mmHg, furosemide 50-100 mg/day was added.
All blood pressure recordings were made at the end of dosing interval, using a mercury sphygmomanometer after 10 min in sitting position. DBP was determined at Korotkoff phase V; the average of three consecutive readings, taken 30 seconds apart, was registered as the blood pressure.
Daily protein intake of 0.8 g/kg/day and daily caloric intake of 35 kcal/kg/day were recommended. A restrict salt intake of less than 100 mEq/day was prescribed.

Trial outcomes

Changes in blood pressure values from baseline were considered as the primary outcome of the study. Patients who achieved a DBP value below 90 mmHg were defined as responders.
Renal function variations during the study were defined as secondary outcome of efficacy and safety in the mean time. Renal function decline was evaluated by means of serial determinations of serum creatinine and creatinine clearance values. In a subgroup of patients, a study of renal hemodynamics was performed at the screening visit and after eight weeks of treatment.

Statistical analysis

Data analysis was performed on a intention-to-treat basis in all the patients who completed at least four weeks of treatment. Continuous variables were

expressed as means ± standard deviation; qualitative variables were expressed as absolute frequencies and percentages of the different modalities. Differences between the means of blood pressure values observed at baseline and those observed after 4, 8, 12, and 48 weeks of active treatment were compared by means of the t-test for dependent samples; differences between the means of variations were evaluated by means of the t-test for independent samples.

The course of blood pressure values during follow-up in the patients who completed the study was evaluated by means of the analysis of variance for repeated tests, where blood pressure values during follow-up were the dependent variables and the randomization group, gender and age classes (<50 and ≥50; <60 and ≥60) were the explanatory variables. Changes in renal function (serum creatinine and creatinine clearance) and proteinuria from baseline were evaluated by means of models of linear analysis, considering the randomization group and the baseline value as explanatory variables. The time course of these indicators in the patients who completed the study was evaluated by means of the analysis of variance for repeated tests, considering the randomization group as the only explanatory factor.

The Chi-square test was used to compare frequencies of patients who achieved or did not DBP normalization. A Cox-regression model was applied to evaluate the rate of response to treatment by also considering therapy composition (single dose/ double dose/ association), which was defined using time-dependent variables. Observed events were the first normalization of DBP (< 90 mmHg) and a decrease in DBP of at least 10% from baseline.

Hemodynamics evaluation was performed in a subgroup of patients. The analysis was made on the changes of the single variables (mean arterial pressure, mean renal vascular resistance, renal blood flow, glomerular filtration volume), by applying general linear models, in which treatment was the explanatory factor and the value at screening the covariate.

A two-tailed *P* value < 0.05 was considered to indicate statistical significance. Data management and statistical analyses were carried out with SAS and/or SPSS software.

TABLE I - BASELINE CHARACTERISTICS OF THE PATIENTS IN THE MANIDIPINE (M) AND ENALAPRIL (E) GROUPS

Characteristics	Manidipine	Enalapril
Patients (n.)	67	64
Age (yrs)	52.9 ± 10.5	56.4 ± 10.0
Male sex (%)	73	66
Weight (kg)	74.0 ± 13.1	73.1 ± 13
Duration of hypertension (yrs)	8.6 ± 6.5	9.8 ± 6.6
Duration of nephropathy (yrs)	6.4 ± 5.7	5.5 ± 4.8
Systolic blood pressure (mmHg)	155.0 ± 11.7	157.3 ± 11.8
Diastolic blood pressure (mmHg)	100.3 ± 4.2	100.3 ± 4.2
Proteinuria (g/24 h)	1.6 ± 1.59	1.37 ± 1.45
Serum creatinine (mg/dL)	2.00 ± 0.52	1.86 ± 0.55
Creatinine clearance (ml/min)	42.9 ± 15.3	46.3 ± 46.3

Results

One hundred and thirty-six patients were eligible for the study and randomly assigned to receive manidipine (M) (67 patients) or enalapril (E) (69 patients). Five patients were excluded from "efficacy analysis" because they had stopped the study before the clinical control foreseen at week 4. The demographic, clinical and laboratory characteristics of the two groups at randomization are summarized in Table I. The male sex was prevalent in the two groups (73% in M and 66% in E); the difference between the mean ages was negligible (56.4 ± 10.0 years in E, 52.9 ± 10.5 years in M; p = 0.0502). The patients treated with E had been hypertensive before entering the study for a longer time than those treated with M (9.8 ± 6.6 years and 8.6 ± 6.5 years, respectively); the first diagnosis of nephropathy was made 6.4 ± 5.7 years and 5.5 ± 4.8 before entering the study in the M and E groups, respectively. No differences in tobacco, alcohol and coffee use were observed in the two groups.

Antihypertensive agents

Considering the subjects who received at least four weeks of treatment (intention-to-treat analysis), 17 (25.4%) patients in M and 27 (42.2%) in E received the lowest dose foreseen by the study protocol (10 mg once daily for both agents); 18 (26.9%) patients in M and 19 (29.7%) in E increased the dose to 20 mg once daily; metoprolol retard (200 mg once a day) was added in 17 (25.4%) and in 11 (17.2%) patients in M and E group, respectively; furosemide was associated in 15 (22.4%) patients in M and in 7 (10.9%) in E. Considering the subgroup completing the study, 56% of the patients (28/50) received M alone and 74% of the patients (38/51) received E alone.

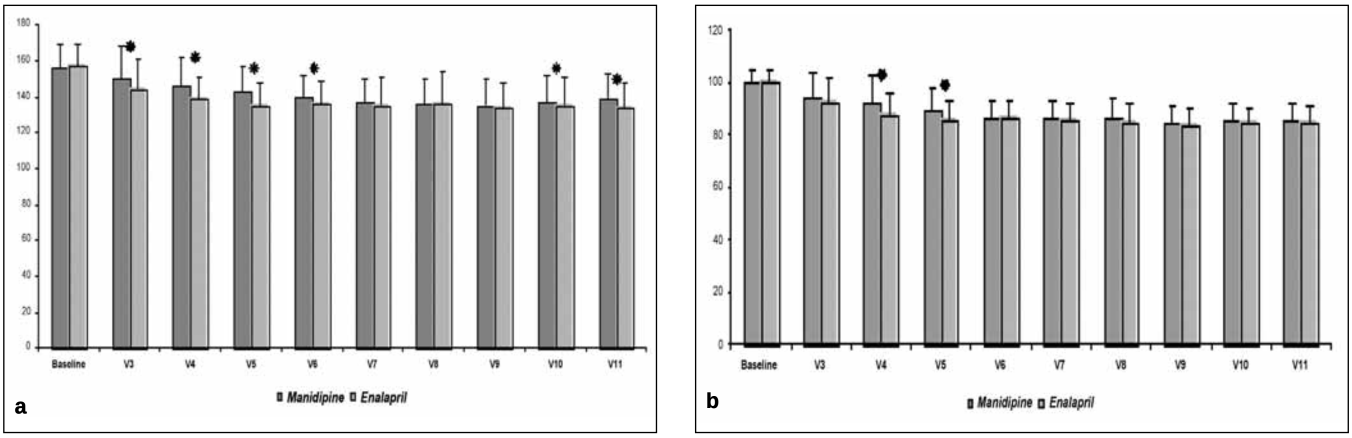

Fig. 2 - a) Systolic blood pressure in the two groups during follow-up, ITT (M=67; E=69).
*** The difference between the means of the two groups adjusted for gender, age and baseline values is statistically significant.**
b) Diastolic blood pressure in two groups during follow-up, ITT (M=67; E=69).
*** The difference between the means of the two groups adjusted for gender, age and baseline values is statistically significant.**

Blood pressure

Both treatments provided a significant reduction in DBP and SBP from baseline values. Figure 2a and 2b summarize SBP and DBP variations in the two groups during follow-up. Considering the subgroups in different time intervals, these variations in DBP and SBP, adjusted for age, sex and baseline values, were already statistically significant ($p < 0.05$) at the end of the first time interval (baseline-visit 3).

Significant differences between the two groups in DBP were observed at visit 4 (-13.5 mmHg in E vs -8.6 mmHg in M) and at visit 5 (-15.4 mmHg in E vs –10.6 mmHg in M). Gender significantly influenced treatment effect, since a larger reduction in DBP was observed in women in the first three time intervals.

Differences in SBP between the two groups were also noted at all the follow-up visits but at visit 7 (p=0.08), at visit 8 and 9 (p=0.06). Gender did not influence the degree of SBP reduction.

In relatively younger patients, a greater reduction was observed, in particular, in SBP.

Similar findings were obtained also in the subgroup of patients who completed the study.

In this subgroup, mean SBP decreased from 155 ± 11.7 mmHg at baseline to 138.7 ± 13.9 mmHg at visit 11 (week 48) in M (n = 50) and from 157.3 ± 11.8 mmHg at baseline to 134.2 ± 13.9 mmHg at visit 11 (week 48) in E (n = 51); the mean DBP decreased from 100.3 ± 4.2 mmHg at baseline to 86.1 ± 6.5 mmHg at week 48 in M and from 100.3 ± 4.2 to 84.7 ± 6.3 mmHg in E. Enalapril caused a mean decrease in SBP of 5.3 mmHg higher than manidipine ($p < 0.01$), and a mean reduction in DBP of 2.4 mmHg higher than manidipine ($p < 0.05$).

By intention-to-treat analysis, the normalization of DBP (<90 mmHg) was more rapid in E than in M, in particular in women and in younger subjects, although the contribution of randomization group was not statistically significant in the Cox regression analysis (p = 0.06).

The percentage of normalized patients was greater in the E group: it was significant at visit 4 (63.9% in E vs 42.4% in M) and at visit 5 (73% in E vs 54% in M). At the end of the study (visit 11), this difference was not statistically significant (80.4% in E vs 76% in M). It is of note that twenty-seven patients in M (40.3%) and twenty-six in E (40.6%) were normalized “once and for all”, 46.3% of the patients in M and 53.1% in E were temporarily normalized, nine subjects in M (13.4%) and four in E (6.2%) were never normalized.

A SBP < 140 mmHg was obtained in 52% of the patients in M and in the 68% of the patients in E.

Finally, the proportion of patients in whom the target blood pressure (<140/90 mmHg) was achieved was 53% in E and 38% in M.

Proteinuria

At baseline, mean urinary protein excretion was not significantly different in the two groups.

Mean proteinuria variations in the two groups during follow-up are summarized in Figure 3. Although proteinuria behavior was characterized by considerable variability in the single patient, mean changes during follow-up were substantially negative in E and positive in M; these changes from baseline were statistically significant in M at visit 5 (+0.70 g/24h, adjusted mean) and in E at visit 11 (-0.40 g/24h, adjusted mean).

When comparing the two treatments, in the first inter-

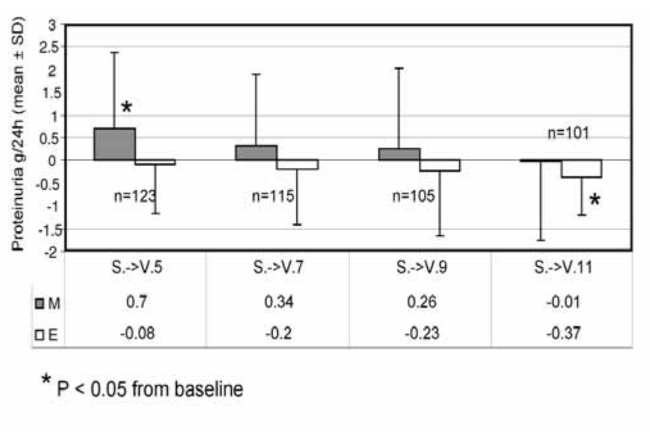

Fig. 3 - Mean proteinuria variations in the two groups during follow-up.

val (baseline-visit 1) the difference between values of absolute changes in the two groups was statistically significant (+0.70 in M vs -0.075 in E, adjusted means; $p<0.05$). The percentage changes between the two groups were statistically significant in all treatment periods.

In patients who completed the study, proteinuria remained substantially unchanged in the manidipine group (n = 49) (from 1.6 ± 1.59 g/24h at baseline to 1.62 ± 1.79 g/24h at visit 11). Similarly, in the subgroup of patients treated with manidipine alone (n = 26), proteinuria was 1.78 g/24h at baseline and 1.97 g/24h at the end of the study.

By contrast, proteinuria showed a significant decrease in the enalapril group (n = 50) (from 1.37 ± 1.45 g/24h at baseline to 1 ± 1.55 g/24h). Similar behavior was observed in the subgroup of patients treated with enalapril alone (n = 35) (from 1.29 g/24h to 0.8 g/24h).

Renal function

At baseline, mean serum creatinine showed no significant differences between the two groups. By intention-to-treat analysis, no significant difference in changes of serum creatinine from baseline were observed in the two groups, although this parameter showed considerable variability during the study period.

Considering the patients who completed the study, there were no significant changes in serum creatinine values during follow-up: from 2.00 ± 0.52 mg/dL in M (n = 46) and 1.86 ± 0.55 mg/dL in E (n = 44) at baseline to 2.09 ± 0.68 mg/dL in M and 1.99 ± 0.60 mg/dL in E at the end of the study. The slope of the reciprocal of serum creatinine against time, calculated at the end of the study as variation for month of treatment, was 0.720 in M and 1.064 in E (p = NS).

Creatinine clearance decreased from 42.9 ± 15.3 ml/min to 39.2 ± 16.8 ml/min in M and from 46.3 ± 16.9 ml/min to 44.4 ± 15.7 ml/min in E. There were no significant difference between the two groups. The slope, calculated at the end of the study as variation for month of treatment, was –0.005 in M e –0.003 in E (p = NS).

Renal hemodynamics

This study was performed in a subgroup of twenty-nine patients (13 and 16 in the manidipine and enalapril group, respectively), who were observed at screening and after four weeks of treatment (visit 3 or before visit 4). At screening, these patients had comparable values of serum creatinine, creatinine clearance and proteinuria; the patients in the enalapril group were slightly older than those in the manidipine group (mean age of 60 ± 5 years and 54 ± 12 years, respectively). Mean arterial pressure at baseline was 117.2 ± 5.6 mmHg in M and 117.1 ± 6.4 mmHg; mean variations from baseline were statistically significant in both groups (-9.3 ± 7.1 mmHg in M and –10.5 ± 8.0 mmHg in E). At baseline, the mean renal vascular resistance was higher in the enalapril than in the manidipine group (40,910±17,688 dyne/sec/cm^-5/1.73m^2 and 30,026±14,213 dyne/sec/cm^-5/1.73m^2, respectively). However, this difference was not statistically significant (p = 0.08). After treatment, the observed changes of this parameter from baseline were extremely variable in both groups (increases in 5 patients treated with manidipine and in 5 patients treated with enalapril; decreases in 8 patients in the manidipine group and in 11 patients in the enalapril group). The mean variation was negative in the enalapril group (-1077) and positive in the manidipine group (+1545), but was not statistically different.

No statistically significant differences were observed in renal blood flow at baseline (366.5 ± 131.2 ml/min/1.73m^2 in M, 279.0 ± 135.3 ml/min/1.73m^2 in E; p=0.09) and in mean variations after treatment (-23.7 and -30.3 in M and E, respectively). Glomerular filtration volume was tested in 12 patients in M and 15 patients E. Mean values at screening were higher in M than in E (69.9 ± 37.0 ml/min/1.73m^2 and 50.7 ± 23.3 ml/min/1.73m^2, respectively), but this difference was not statistically significant. Mean variation from baseline (-6.7 ± 15.9 ml/min/1.73m^2, adjusted mean) was statistically significant in E; no difference was found between groups.

Safety

Altogether, during the follow-up period, 17 (25%) patients in M and 18 (26%) in E discontinued the treatment.

Twenty-one patients (11 in M and 10 in E) experi-

enced adverse events: in particular, seven in M and 3 in E showed deterioration in renal function. Fourteen patients (6 in M and 8 in E) discontinued treatment for “various reasons”: in particular seven for uncontrolled hypertension (2 in M and 5 in E).

Discussion

Manidipine is a long-lasting CCB which demonstrated its action not only on the afferent arteriole, as other dhCCB, but also on the efferent one. This action, which should theoretically prevent any increase in intra-glomerular pressure, would be similar to that of ACE-I.

This study is the first, prospective, randomized trial comparing the effect of manidipine against an ACE-I, enalapril, in hypertensive patients with CKD. We found that manidipine was a safe and effective antihypertensive agent, capable of obtaining a significant reduction in SBP and DBP from baseline. This antihypertensive effect was already statistically significant after one month of treatment and was long-lasting. Indeed, 40.3% of the patients had their DBP normalized once and for all during the study-period. This is in line with the results of a multicenter study in 71 patients with hypertension and renal impairment receiving 5 to 20 mg/day of manidipine: blood pressure was well controlled in 65.6% of the 32 subjects who were followed for more than 48 weeks (10). In our study, the patients in the enalapril group also experienced a significant reduction in blood pressure during follow-up; the magnitude of this reduction was significantly higher than that observed in the manidipine group. Similarly to manidipine, enalapril achieved a long-lasting normalization of DBP in 40.6% of the subjects. Furthermore, patients in the enalapril group showed a better antiproteinuric response than those in the manidipine group. It is possible that the lower blood pressure values obtained with enalapril could have amplified this difference.

We did not find any difference in the rate of progression of CKD (calculated as the slope of the reciprocal of serum creatinine against time and as creatinine values variation during follow-up) between the two treatment groups. These findings possibly suggest that the two treatments were equally effective in reducing the rate of progression of CKD. However, our data are insufficient to drive definitive conclusions about this issue, considering that this was not the primary aim of the study and thus the sample size and the study follow-up were insufficient to test this aspect.

In the past few years, several controlled clinical trials have compared the effects of ACE-I with conventional antihypertensive agents or CCBs other than manidipine on blood pressure, proteinuria and residual renal function in patients with diabetic- (3) and non-diabetic renal disease (4-6, 11, 12). Zucchelli et al (11) found a significant reduction in the rate of creatinine clearance loss over a period of three years in 121 patients treated with captopril or nifedipine (-0.22 ml/min/mth in the captopril group and -0.24 ml/min/mth in the nifedipine group), in comparison with the rate of decline observed when standard antihypertensive therapy was used during the year before randomization (-0.46 ml/min/mth). However, these results were attenuated by the fact that both SBP and DBP values achieved during captopril and nifedipine therapy were much lower than during standard hypertensive therapy.

Maki et al (13) performed a multivariate analysis of controlled and uncontrolled trials involving patients with diabetic and non-diabetic renal diseases, and found that ndhCCBs, together with ACE-I, were capable of inducing a reduction in proteinuria that was partially independent of blood pressure changes, whereas dhCCBs had no apparent effect on proteinuria. Another meta-analysis confirmed that, in contrast with the absence of change or more often observed increase in albuminuria in response to dhCCB, diltiazem or verapamil administration was associated with a decline in proteinuria (14). However, caution is needed in evaluating these meta-analyses, because they mainly consider small and not always prospective studies, with a relatively short period of follow-up.

More recently, the African American Study of Kidney Disease and Hypertension (AASK) (15), a randomized, double-blind trial of 1094 African Americans with hypertensive renal disease, which was designed to evaluate the impact on progression of kidney disease of two different blood pressure goals (low and usual), revealed a renoprotective effect of the ACE-I ramipril as compared to the dhCCB amlodipine, independent of the achieved level of arterial pressure. However, these results are not of univocal interpretation because the effects of amlodipine on renal function differed according to baseline proteinuria. In patients with an urinary protein to creatinine ratio of > 0.22 (corresponding approximately to a proteinuria of > 300 mg/day), the ramipril group had a 48% reduction in the risk of the clinical endpoints (reduction in GFR of more than 50%, ESRD, or death) and a significant slower mean decline in GFR in comparison with the amlodipine group; after three years of follow-up, GFR remained above baseline values in patients without significant baseline proteinuria treated with amlodipine and it was slightly higher than in the ramipril group.

Altogether, the results of the published studies suggest that dhCCBs do not seem to afford substantial renal protection in diabetic and non-diabetic chronic renal disease, probably related to the lack of consistent decrease in proteinuria by these agents. One

possible explanation of these results could be that, since the majority of CCBs allows the linear transmission of systemic hypertension into the glomerular capillaries, the maximal renoprotective benefits of CCBs may require strict blood pressure control, and the reports of their lack of efficacy may be partially due to inadequate control. An artefact may also be introduced when short-acting CCBs are used, because these may intermittently leave renal vessels unprotected against high systemic blood pressure, particularly when preglomerular vessels are still vasodilated as a result of the drugs' preferential action on the afferent arteriole. However, ndhCCBs and some dhCCBs, such as manidipine, also vasodilate the efferent arteriole and only partially affect renal autoregulation (16, 17).
Interestingly enough, we did not find any statistical significant difference in mean renal vascular resistance, renal blood flow and glomerular filtration volume between the two treatments groups, indicating that manidipine could have similar effects to enalapril on renal hemodynamics.
A possible limitation of this study is that it was designed before the awareness of the need for strict blood pressure control in order to slow down CKD progression, and so it was not aimed at reaching the currently accepted target blood pressure (125/75 mmHg) for patients with proteinuria. However, this limitation applies to the majority of the clinical trials performed so far aimed at testing the efficacy of ACE-I in reducing the rate of progression of CKD. For this reason, it is still unclear whether they are really superior to other antihypertensive agents when low blood pressure values are achieved according to present guidelines (18).
In conclusion, we found that manidipine is safe and effective in reducing blood pressure in patients with CKD. Even if this agent was not effective in reducing proteinuria, its effect on renal function was similar to that of enalapril in patients without glomerular diseases. Certainly, the renoprotective effects of ACE-I (and angiotensin II receptor antagonists in patients with type 2 diabetes (19)) are well established nowadays, and are considered as the first-line treatment in patients with non-diabetic, proteinuric CKD. However, the majority of CKD patients need multidrug antihypertensive treatment. For this reason, it is important not only to identify the antihypertensive agent of first choice, but also to clarify whether other agents, such as CCBs and in particular manidipine, are safe and effective agents to combine with ACE-I. CCBs are well tolerated in everyday clinical practice and provide valid reduction in blood pressure values. Thus, they should be considered as second-line antihypertensive treatment in patients with CKD, at least in patients without primary glomerular disease. The possibility that combination treatments with ACEIs and CCBs may have additive or even synergistic renoprotective effects other than blood pressure control is extremely fascinating, but at present available data are insufficient to confirm this hypothesis (20).

Acknowledgements

The authors acknowledge the participation of the following Manidipine Study Group:
Study Coordinators: G. Maschio, A. Salvetti
Study Investigators: A. Albertazzi (Modena, Italy), P.P. Altieri (Cagliari, Italy), F. Antonucci and F. Modena (Feltre (BL), Italy), A.M. Bernardi (Rovigo, Italy), C. Cascone and C. Abaterusso (Castelfranco Veneto, (TV), Italy), M. Fusaroli (Ravenna, Italy), W. Huber and P. Riegler (Bolzano, Italy), F. Locatelli, L. Del Vecchio and M.Pozzi (Lecco, Italy), G. Maschio (Verona, Italy), V. Mioli (Ancona, Italy), G. Mioni (Udine, Italy), V. Panichi and D. Taccola (Pisa, Italy), C. Rovati and N. Buccella (Trento, Italy), M. Sasdelli (Arezzo, Italy), F. Scanferla (Mestre (VE), Italy), F.P. Schena (Bari, Italy), S. Stefoni (Bologna, Italy), C. Zoccali and M. Garozzo (Reggio Calabria, Italy).

This work was supported by a grant from Takeda Italia Farmaceutici, Italy.

Address for correspondence:
Prof. Francesco Locatelli, M.D.
Department of Nephrology and Dialysis
Ospedale Alessandro Manzoni
Via dell'Eremo, 9
23900 Lecco, Italy
nefrologia@ospedale.lecco.it

References

1. Peterson JC, Sharon A, Burkart JM, Greene T, Hebert LA, Hunsicker LG, for the Modification of Diet in Renal Disease (MDRD) Study Group. Blood pressure control, proteinuria and the progression of renal disease. The Modification of Diet in Renal Disease Study. Ann Intern Med 1995; 123: 754-62.
2. Foley RN, Parfrey PS, Harnett JD, Kent GM, Murray DC, Barre PE. Impact of hypertension on cardiomyopathy, morbidity and mortality in end-stage renal disease. Kidney Int 1996; 49: 1379-85.
3. Lewis EJ, Hunsicker LG, Bain RP, Rohde RD. For the Collaborative Study Group: The effect of Angiotensin-Converting-Enzyme inhibition on diabetic nephropathy. N Engl J Med 1993; 329: 1456-62.
4. Maschio G, Alberti D, Janin G, Locatelli F, Mann JF, Motolese M, Ponticelli C, Ritz E, Zucchelli P, and the Angiotensin-Converting-Enzyme Inhibition in Progressive Renal Insufficiency Study Group: Effect of the Angiotensin-Converting-Enzyme Inhibitor Benazepril on the Progression of Chronic Renal Insufficiency. N Engl J Med 1996; 334: 939-45.
5. The GISEN Group (Gruppo Italiano di Studi Epidemiologici in Nefrologia): Randomised placebo-controlled trial of effect of ramipril on decline in glomerular filtration rate and risk of terminal renal failure in proteinuric, non-diabetic nephropathy. Lancet 1997; 349: 1857-63.
6. Jafar TH, Schmid CH, Landa M, Giatras I, Toto R, Remuzzi G, Maschio G, Brenner BM, Kamper A, Zucchelli P, Becker G, Himmelmann A, Bannister K, Landais P, Shahinfar S, de Jong PE, de Zeeuw D, Lau J, Levey AS. Angiotensin-converting enzyme inhibitors and progression of nondiabetic renal disease. A meta-analysis of patient-level data. Ann Intern Med 2001; 135: 73-87.
7. Dworkin LD. Impact of calcium entry blockers on glomerular injury in experimental hypertension. Cardiovasc Drug Ther 1990; 4: 1325-30.
8. Sweeney C, Shultz P, Raji L. Interactions of the endothelium and mesangium in glomerular injury. J Am Soc Nephrol 1990; 1 (suppl): S13-20.
9. Rodicio JL. Renal effects of calcium antagonists with special reference to manidipine hydrochloride. Blood Press 1996; 5 (suppl): S10-5.
10. Saruta T, Suzuki H. Efficacy of manidipine in the treatment of hypertension with renal impairment: a multicenter trial. Am Heart J 1993; 125 (2 Pt 2): 630-4.
11. Zucchelli P, Zuccala A, Borghi M, Fusaroli M, Sasdelli M, Stallone C, Sanna G, Gaggi R. Long term comparison between captopril and nifedipine in the progression of renal insufficiency. Kidney Int 1992; 42: 452-8.
12. Cinotti GA, Zucchelli PC. Effect of Lisinopril on the progression of renal insufficiency in mild proteinuric non-diabetic nephropathies. Nephrol Dial Transplant 2001; 16: 961-6.
13. Maki DD, Ma JZ, Louis TA, Kasiske BL. Long-term effects of antihypertensive agents on proteinuria and renal function. Arch Intern Med 1995; 155: 1073-80.
14. Gansevoort RT, Sluiter WJ, Hemmelder MH, de Zeeuw D, de Jong PE. Antiproteinuric effect of blood pressure lowering agents: a meta analysis of comparative trials. Nephrol Dial Transplant 1995; 10: 1963-74.
15. Agodoa LY, Appel L, Bakris GL, Beck G, Bourgoignie J, Briggs JP, Charleston J, Cheek D, Cleveland W, Douglas JG, Douglas M, Dowie D, Faulkner M, Gabriel A, Gassman J, Greene T, Hall Y, Hebert L, Hiremath L, Jamerson K, Johnson CJ, Kopple J, Kusek J, Lash J, Lea J, Lewis JB, Lipkowitz M, Massry S, Middleton J, Miller ER 3rd, Norris K, O'Connor D, Ojo A, Phillips RA, Pogue V, Rahman M, Randall OS, Rostand S, Schulman G, Smith W, Thornley-Brown D, Tisher CC, Toto RD, Wright JT Jr, Xu S; African American Study of Kidney Disease and Hypertension (AASK) Study Group. Effect of ramipril vs amlodipine on renal outcomes in hypertensive nephrosclerosis: a randomized controlled trial. JAMA 2001; 285: 2719-28.
16. Arima S, Ito S, Omata K, Tsunoda K,Yaoite H, Abe K. Diverse effects of calcium antagonists on glomerular hemodynamics. Kidney Int 1996; 49 (suppl 55): S132-4.
17. Saruta T, Kanno Y, Hayashi K,Konishi K. Antihypertensive agents and renal protection: calcium channel blockers. Kidney Int 1996; 49 (suppl 55): S52-6.
18. Locatelli F, Del Vecchio L, D'Amico M, Andrulli S. Is it the agent or the blood pressure level that matters for renal protection in chronic nephropathies? J Am Soc Nephrol 2002; 13 (suppl): S196-201.
19. Lewis EJ, Hunsicker LG, Clarke WR, Berl T, Pohl MA, Lewis JB, Ritz E, Atkins RC, Rohde R, Raz I; Collaborative Study Group.. Renoprotective effect of the angiotensin-receptor antagonist irbesartan in patients with nephropathy due to type 2 diabetes. N Engl J Med 2001; 345: 851-60.
20. Locatelli F, Del Vecchio L, Andrulli S, Colzani S. Role of combination therapy with ACE inhibitors and calcium channel blockers in renal protection. Kidney Int 2002; 62 (suppl 82): S53-60.

Received: September 12, 2003
Revised: October 23, 2003
Accepted: January 18, 2004

© Società Italiana di Nefrologia
