# Supplementary material for: Effects of T-Type Calcium Channel Blockers on Renal Function and Aldosterone in Patients with Hypertension: A Systematic Review and Meta-Analysis
Source: PLoS One. 2014 Oct 17;9(10):e109834. doi: 10.1371/journal.pone.0109834 (PMC4201480; doi:10.1371/journal.pone.0109834)
Supplement: File S3 — PDF files of twenty-four studies included in the meta-analysis. (ZIP) [file pone.0109834.s007.zip › Supporting information-PDF files/35. Am J Hypertens 2003[16(2)]116-122.pdf]

Effect of Efonidipine and ACE Inhibitors on Proteinuria in Human Hypertension With Renal Impairment

Koichi Hayashi, Hiroo Kumagai, and Takao Saruta

Background: Although several lines of recent studies fail to demonstrate the beneficial action of calcium antagonists, a novel dihydropyridine efonidipine, which possesses dilatory action of both afferent and efferent arterioles and, therefore, shares the renal microvascular action with angiotensin converting enzyme (ACE) inhibitors, is reported to exhibit renal protection in experimental animals.

Methods: The present study evaluated the effect of efonidipine and ACE inhibitors on blood pressure (BP) and proteinuria. Sixty-eight hypertensive patients with renal impairment (serum creatinine, >1.5 mg/dL) or chronic renal parenchymal disease were randomly assigned to efonidipine or ACE inhibitor treatment. Of the 68 patients, 23 were treated with efonidipine and 20 with ACE inhibitors; these patients were analyzed for the 48-week study.

Results: Both efonidipine and ACE inhibitors produced a similar degree of reductions in BP (efonidipine, from 161 ± 2/93 ± 2 to 142 ± 5/82 ± 2 mm Hg; ACE inhibitor, from 163 ± 3/95 ± 2 to 141 ± 5/83 ± 2 mm Hg), and maintained creatinine clearance for 48 weeks. Proteinuria tended to decrease in both groups, and a significant reduction was observed in proteinuric patients (>1 g/day) (efonidipine, from 2.7 ± 0.3 to 2.1 ± 0.3 g/day; ACE inhibitor, from 3.0 ± 0.4 to 2.0 ± 0.5 g/day). Of interest, efonidipine decreased proteinuria in proteinuric patients who failed to manifest decreases in systemic BP. Finally, the incidence of adverse effects, including hyperkalemia and cough, was less in the efonidipine-treated group.

Conclusions: Both efonidipine and ACE inhibitors preserved renal function in hypertensive patients with renal impairment. The antiproteinuric effect was apparent in patients with greater proteinuria. The beneficial action of efonidipine, along with fewer side effects, may favor the use of this agent in the treatment of hypertension with renal impairment. Am J Hypertens 2003;16:116–122 © 2003 American Journal of Hypertension, Ltd.

Key Words: Efonidipine, ACE inhibitors, renal disease, proteinuria, hypertension.

The kidney plays an important role in the regulation of systemic blood pressure (BP) as well as the homeostasis of water and electrolyte balance. Because of the well-established observations that the impaired renal function is closely associated with increases in cardiovascular events,[1,2] much attention has been paid to the strategy for the prevention of progressive nature of chronic renal disease. Recently, a number of large-scale clinical trials have witnessed the beneficial action of angiotensin converting enzyme (ACE) inhibitors.[3–5] This class of antihypertensive agent possesses unique actions associated with angiotensin II (Ang II) blockade. Angiotensin II increases mesangial matrix formation directly by stimulating its production,[6] and indirectly by increasing glomerular capillary pressure.[7] The latter action is of particular interest, as the ACE inhibitor elicits efferent as well as afferent arteriolar dilation, and the subsequent amelioration of glomerular hypertension.[8] In contrast to this salutary action of the ACE inhibitor, the calcium antagonist is demonstrated to produce a preferential dilation of the afferent arteriole.[9,10] This observation suggests that although the net effect of the calcium antagonist depends on the balance between the reduction in systemic BP and the dilation of afferent arterioles, there exists the possibility that this agent may produce pressure load on the glomerulus, and might deteriorate the outcome of chronic renal disease.

Recently, a large number of calcium antagonists have been developed that differ from conventional types of these agents. Of particular interest, efonidipine, a dihydropyridine calcium antagonist, has been demonstrated to possess the ability to dilate both afferent and efferent

Received May 22, 2002. First decision June 19, 2002. Accepted September 5, 2002.

From the Department of Internal Medicine, School of Medicine, Keio University, Tokyo, Japan.

Address correspondence and reprint requests to Dr. Koichi Hayashi, Department of Internal Medicine, School of Medicine, Keio University, 35 Shinanomachi, Shinjuku-ku, Tokyo 160-8582, Japan; e-mail: khayashi@sc.itc.keio.ac.jp

0895-7061/03/$30.00
PII S0895-7061(02)03147-3

© 2003 by the American Journal of Hypertension, Ltd.
Published by Elsevier Science Inc.

arterioles in vitro[11] and in vivo.[12] The unique action of this type of the calcium antagonist sharply contrasts with traditional calcium antagonists exhibiting preferential action on the afferent arteriole, and may produce distinct effect on renal injury. Fujiwara et al[13] indicated that efonidipine improved the renal injury in subtotally nephrectomized spontaneously hypertensive rats to the same level as that of enalapril, whereas nifedipine failed to halt the progression of renal injury. This observation, therefore, lends support to the formulation that the agents with efferent as well as afferent arteriolar dilator action exert favorable action on the aggravation of renal injury. Furthermore, efonidipine may constitute an alternative tool for the treatment of hypertension with renal disease, when the ACE inhibitor cannot be prescribed. Nevertheless, no investigations have been conducted examining the effect of efonidipine on the progression of human renal disease.

In the present study, we have examined the 48-week effect of efonidipine on renal function and proteinuria in patients with renal impairment, and compared its efficacy with that of ACE inhibitors, which shared the functional characteristics of efferent arteriolar dilation with efonidipine. Furthermore, the clinical relevance of efonidipine has also been compared, including the incidence of adverse effects.

Methods

Study Population

The effect of a novel calcium antagonist, efonidipine, and an ACE inhibitor on systemic BP and urinary protein excretion were examined in hypertensive patients with renal impairment. Sixty-eight patients with hypertension more than 140 or 90 mm Hg were enrolled at 27 centers (see Acknowledgments) in this study. The inclusion criteria for entry were essential hypertension with serum creatinine >1.5 mg/dL, or hypertension with chronic renal parenchymal disease (Table 1). The patients with moderate renal dysfunction (serum creatinine >3.0 mg/dL) or severe hypertension (>180/120 mm Hg) were excluded from the study. The study protocols had been approved by each Institutional Review Committee, and written informed consent had been obtained from all patients at the entry into the study.

Table 1. Patients profiles before treatment with efonidipine or ACE inhibitors

	Efonidipine	ACE Inhibitors
Age (y/o)	58 ± 3	57 ± 3
n (male/female)	23 (18/5)	20 (12/8)
Etiology of renal disease		
Essential hypertension	8	7
Primary glomerular disease	10	6
Systemic lupus erythematodes	0	1
Polycystic kidney disease	1	2
Chronic pyelonephritis	0	2
Diabetic nephropathy	3	1
Others/unknown	1	1
Systolic blood pressure (mm Hg)	161 ± 2	163 ± 3
Diastolic blood pressure (mm Hg)	93 ± 2	95 ± 2
Heart rate (beats/min)	75 ± 2	75 ± 2
Serum creatinine (mg/dL)	1.8 ± 0.1	1.9 ± 0.1
Blood urea nitrogen (mg/dL)	24 ± 2	26 ± 2
Urinary protein (g/d)	1.7 ± 0.3	2.1 ± 0.4
Creatinine clearance (mL/min)	43 ± 4	37 ± 4

ACE = angiotensin converting enzyme.
Results are means ± SEM.
P is not significantly different between two groups.

Study Protocols

After a 4-week control/wash-out period, the patients were randomly allocated to either an ACE inhibitor-(enalapril, lisinopril, or imidapril) treated (n = 30) or efonidipine-treated group (n = 38), using concealed randomization. In this open-labeled clinical trial, either ACE inhibitor (enalapril, 2.5 mg/day; lisinopril, 5 mg/day; imidapril, 5 mg/day) or efonidipine (20 mg/day) was administered, and the doses of ACE inhibitor and efonidipine were adjusted according to the office BP measured in the sedentary position. Office BP and heart rate were measured every month, and blood samples were taken every 12 weeks for determination of serum creatinine, blood urea nitrogen, and serum potassium concentration. All patients were instructed to restrict dietary sodium intake to 6 g/day throughout the study period. Daily urinary protein and sodium excretion was measured every 6 months. Creatinine clearance was determined from the daily urinary creatinine excretion/plasma creatinine concentration and evaluated every 3 months. Urinary protein was determined by the pyrogallol red molybdate method.

Because the renal protective action of antihypertensive agents was reported to differ, with greater protection obtained in patients with proteinuria >1 g/day,[14] the patients were further stratified into two groups according to the magnitude of daily urinary protein excretion (ie, proteinuria >1 or <1 g/day). The effect of ACE inhibitor and efonidipine on systemic BP and proteinuria was then analyzed in the group treated with each agent.

Statistical Analysis

Results are expressed as means ± SEM. Statistical analysis was determined with paired t test, two-way ANOVA followed by Newman-Keuls multiple comparison post hoc

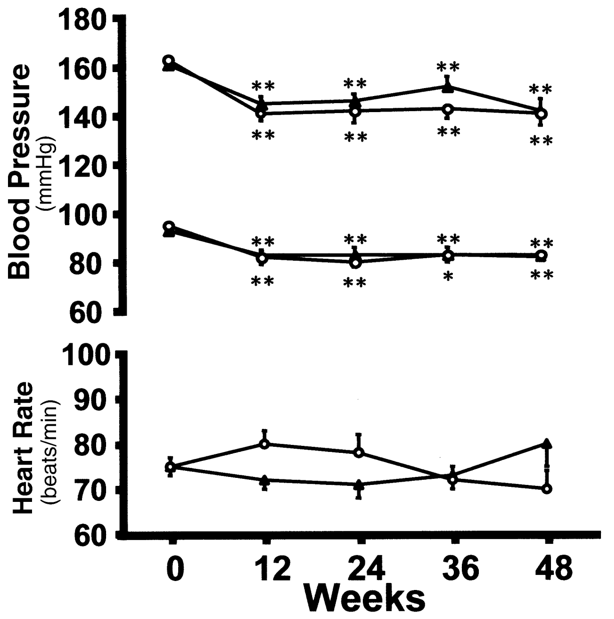

FIG. 1. Effects of angiotensin converting enzyme inhibitors (○) and efonidipine (▲) on systemic blood pressure and heart rates in patients with renal disease. Results are means ± SEM. *$P < .05$; **$P < .01$ *v* 0 week.

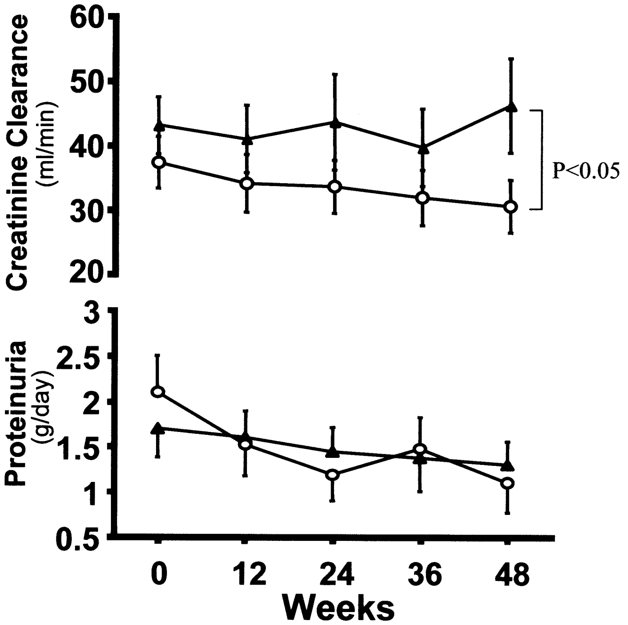

FIG. 2. Effects of angiotensin converting enzyme inhibitors (○) and efonidipine (▲) on creatinine clearance and daily urinary protein excretion in patients with renal disease. Results are means ± SEM.

test, or Wilcoxon's signed rank test, as appropriate. *P* values < .05 were considered statistically significant.

Results

Effects of ACE Inhibitor and Efonidipine on Systemic and Renal Parameters

Among 68 patients enrolled, 15 patients assigned to efonidipine and 10 to ACE inhibitor were excluded because of drop-out as a result of follow-up or physicians' violations of inclusion criteria. Therefore, 23 patients and 20 patients receiving efonidipine and ACE inhibitors (enalapril, lisinopril, and imidapril), respectively, were actually considered suitable for data analyses.

Baseline characteristics before treatment of efonidipine or ACE inhibitor are shown in Table 1. There was no difference in age or the ratio of male to female patients between the two groups. Although the etiology of renal disease is diverse, essential hypertension (efonidipine, 34.8%; ACE inhibitor, 35.0%) and primary glomerular disease (efonidipine, 43.5%; ACE inhibitor, 30.0%) constitute major underlying diseases included in this study. The baseline values for systolic/diastolic BP, heart rate, serum creatinine/blood urea nitrogen, urinary protein excretion, and creatinine clearance were nearly the same between these groups.

The treatment with ACE inhibitor and efonidipine decreases the BP, similar in magnitude with either agent (Fig. 1); both agents significantly reduced BP at 4 weeks, and these changes persisted throughout the study protocols. Heart rates did not change significantly in either group. Blood urea nitrogen or creatinine clearance did not differ between the two groups before treatment with these agents (Table 1). Furthermore, the treatment with these agents had no significant effect on creatinine clearance, although creatinine clearance observed at 48 weeks was slightly higher in the efonidipine group ($P < .05$; Fig. 2). The administration of ACE inhibitor or efonidipine produced slight decreases in urinary protein excretion; however, the changes did not attain statistical significance (Fig. 2). Urinary sodium excretion did not differ between these groups throughout the study (data not shown). The mean doses of ACE inhibitors (enalapril, lisinopril, and imidapril) at the end of the study were 8.3, 11.0, and 5.0 mg/day, respectively. The doses of efonidipine ranged from 20 to 80 mg/day (mean dose at the end of study, 38.0 mg/day); the hypotensive action tended to be dose-dependent within the doses of 20 to 60 mg/day (20 mg, −11 ± 5 mm Hg; 40 mg, −17 ± 2 mm Hg; 60 mg, −25 ± 8 mm Hg), whereas antiproteinuric action did not manifest significant dose dependency.

When the effect of ACE inhibitor and efonidipine was assessed in patients with proteinuria at less than 1 g/day, these agents did not alter urinary protein excretion (ACE inhibitor, from 0.5 ± 0.1 to 0.7 ± 0.4 g/day, $n = 6$; efonidipine, from 0.5 ± 0.1 to 0.4 ± 0.1 g/day, $n = 8$), despite significant decreases in final BP (ACE inhibitor, from 165 ± 4/93 ± 5 to 140 ± 7/81 ± 5 mm Hg, $P < .05$; efonidipine, from 165 ± 4/98 ± 3 to 139 ± 1/83 ± 5 mm Hg, $P < .05$). In contrast, in patients with greater urinary protein excretion (>1 g/day), ACE inhibitors potently reduced proteinuria (Fig. 3). Thus, systemic BP and proteinuria decreased markedly from 163 ± 5/98 ± 2 to 152 ± 9/85 ± 4 mm Hg ($P < .05$, $n = 11$) and from 3.0 ± 0.4 to 1.7 ± 0.4 g/day ($P < .01$) at 24 weeks, and this effect

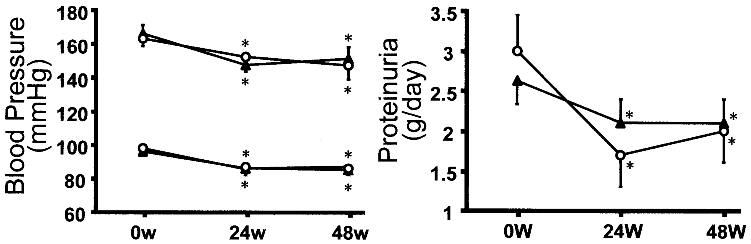

FIG. 3. Effects of angiotensin converting enzyme inhibitors (○) and efonidipine (△) on systemic blood pressure and urinary protein excretion in patients with proteinuria >1 g/day. Results are means ± SEM. *P < .05 v 0 week.

persisted until the end of the study (BP, 147 ± 8/87 ± 4 mm Hg, $P < .05$; proteinuria, 2.0 ± 0.5 g/day, $P < .05$, n = 11). Similarly, efonidipine elicited significant decreases in BP (166 ± 5/96 ± 2, 147 ± 4/86 ± 4, 151 ± 7/85 ± 4 mm Hg at 0, 24, and 48 weeks, respectively, n = 11) and urinary protein excretion (2.7 ± 0.3, 2.1 ± 0.3, and 2.1 ± 0.3 g/day at 0, 24, and 48 weeks, respectively, n = 11).

In patients with greater proteinuria (>1 g/day), the antiproteinuric effects of these agents were further evaluated on the basis of the level of final mean arterial BP (MAP, Fig. 4). In a group of patients in which final (at 48 weeks) MAP reached below 100 mm Hg (from 119 ± 4 to 93 ± 3 mm Hg, $P < .05$, n = 5), ACE inhibitors markedly reduced proteinuria ($P < .05$). The ACE inhibitors, however, failed to alter proteinuria in patients with a final MAP of >100 mm Hg (from 120 ± 3 to 116 ± 6 mm Hg, $P > .5$). In the efonidipine-treated group, proteinuria was decreased in both groups with MAP <100 mm Hg (MAP, from 119 ± 4 to 96 ± 3 mm Hg, $P < .01$; proteinuria, from 1.9 [median], 2.8 [range] to 1.4 [median], 2.2 [range] g/day, $P < .05$, n =5) and those with MAP >100 mm Hg (MAP, from 120 ± 3 to 120 ± 5 mm Hg, $P > .5$; proteinuria, from 2.9 [median], 3.6 [range] to 2.2 [median], 3.1 [range] g/day, $P < .05$. n = 6).

Adverse Effects

The incidence of adverse clinical events and the effects of these agents on other laboratory parameters were greater in the ACE inhibitor group (43.3%) than in the efonidipine group (10.5%, $P < .05$; Table 2). In the ACE inhibitor group, adverse clinical events, including nonproductive cough appeared in 5 patients (v 0 in efonidipine). Furthermore, hyperkalemia developed in 5 patients treated with ACE inhibitor, whereas none of the efonidipine-treated patients manifested this abnormality. When the effect of these agents on serum potassium levels was compared, ACE inhibitors significantly elevated the serum potassium concentration from 4.5 ± 0.1 (0 week) to 5.2 ± 0.1 mEq/L (48 weeks, $P < .01$ v baseline, n = 14). In contrast, efonidipine did not alter the serum potassium level (from 4.3 ± 0.1 to 4.3 ± 0.1 mEq/L, $P > .5$, n = 18).

Discussion

A large amount of evidence has accrued that ACE inhibitors reduce proteinuria and potently retards the progression of renal disease,[4–6] which is reflected by the guidelines announced recently for the treatment of hypertension

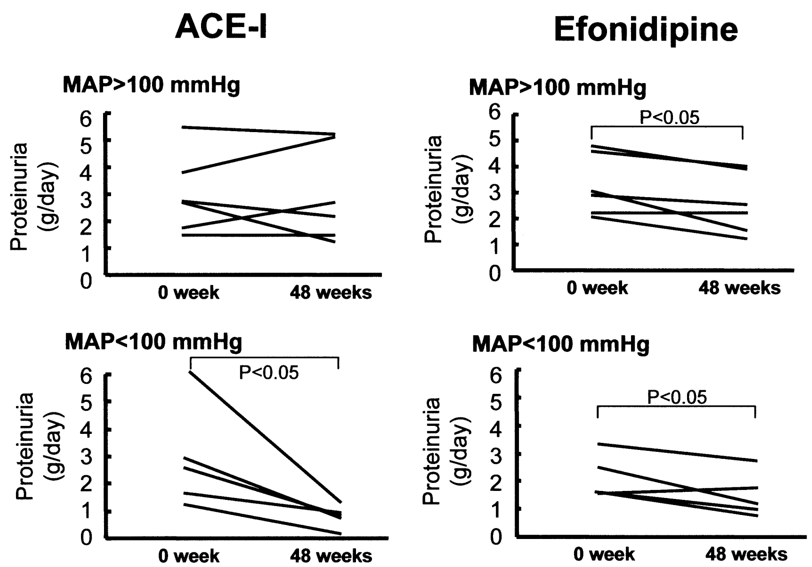

FIG. 4. Role of systemic blood pressure in the development of proteinuria in patients with proteinuria >1 g/day treated with angiotensin converting enzyme inhibitors and efonidipine. ACE-I = angiotensin converting enzyme inhibitors; MAP = mean arterial pressure.

Table 2. Incidence of adverse effects in patients treated with efonidipine and ACE inhibitors

	Efonidipine	ACE Inhibitors
Number	38	30
Incidence of adverse effects		
Cough	0	4
Irritability of throat	0	1
Dyspnea	0	1
Headache	1	1
Hypotension	1	0
Stomatitis	1	0
Proteinuria	0	1
Elevated CPK	0	1
Anemia	0	2
Elevation of serum creatinine	0	1
Elevation of uric acid	1	0
Hyperkalemia	0	5
Liver dysfunction	0	2

CPK = creatine phosphokinase; other abbreviation as in Table 1.

with renal disease.[15,16] Most of the renal action of ACE inhibitors is attributed to the blockade of angiotensin II action, and it is now established that ACE inhibitors dilate both afferent and efferent arterioles,[8,17] leading to a reduction in glomerular capillary pressure.[8] In contrast, the renal action of calcium antagonists is reported to be divergent.[11] A recent trial comparing the effect of amlodipine with that of ramipril (African American Study of Kidney Disease and Hypertension [AASK]) shows that a calcium antagonist is less beneficial than an ACE inhibitor in the progression of renal disease.[18] Because amlodipine preferentially dilates afferent arterioles,[11] amlodipine could elevate glomerular hypertension. In striking contrast to conventional types of calcium antagonists, it has recently been demonstrated that a novel calcium antagonist, efonidipine, dilates both afferent and efferent arterioles in experimental animals in vitro[11] and in vivo,[12] which may share the renal protective action with ACE inhibitors. We have demonstrated that efonidipine is equipotent with enalapril in preventing the development of renal injury in an experimental animal model of chronic renal disease.[13] It has not been examined, however, whether this premise could be extrapolated to human renal disease.

The present open-labeled randomized prospective study has demonstrated that both efonidipine and ACE inhibitors reduce systemic BP (Fig. 1) and preserve renal function in human hypertension with renal disease (Fig. 2). Furthermore, ACE inhibitors and efonidipine tended to decrease proteinuria, although these changes did not reach statistical significance. When compared in the groups with greater proteinuria (>1 g/day), both ACE inhibitors and efonidipine elicited significant reductions in urinary protein excretion (Fig. 3). Because the systemic BP was lowered to nearly the same level by efonidipine and ACE inhibitors, the contribution of the depressor action of these agents to renal protection would be the same in ACE inhibitors and efonidipine. Alternatively, based on the afferent and efferent arteriolar action, these agents may exert intrarenal action that favors reduction in glomerular hypertension, and may contribute to the amelioration of renal injury. Although such tentative speculations remain undetermined, the present study clearly demonstrates a proteinuria-sparing action of these agents in patients with chronic renal injury.

Although both ACE inhibitors and calcium antagonists are potent antihypertensive agents constituting important tools for the treatment of hypertension, there have been reported a substantial number of studies demonstrating distinct action of these agents on renal protection.[19] A large number of investigations indicate that ACE inhibitors can reduce proteinuria and retard the progression of renal disease in both diabetics[3] and nondiabetics.[4,5] In contrast, there is controversy as to the effect of calcium antagonists on renal protection.[19] Zucchelli et al[20] demonstrated that slow-release nifedipine and captopril were equally effective in inhibiting the progression of renal injury. Kumagai et al[21] have also reported that amlodipine and ACE inhibitors are equally potent in blunting the progression of nondiabetic renal disease, although the study population was relatively small. In contrast, the AASK trial demonstrates that amlodipine is less effective than ramipril in inhibiting the progression of renal injury.[18] The present study shows that efonidipine reduces proteinuria as potently as ACE inhibitors, and preserves creatinine clearance (Fig. 2). Because proteinuria is established as a prognostic marker for renal disease,[22] our findings may allow speculation that efonidipine exerts a renal protective action as least equivalent to that produced by ACE inhibitors. Of interest, it has been documented that efonidipine possesses the inhibitory action on T-type calcium channels,[23] which is reported to participate in part in renin secretion.[24] In concert with the vasodilator action on efferent as well as afferent arterioles,[11,12] the pharmacologic characteristics of efonidipine (ie, renin-angiotensin blockade) could be reminiscent of the renal action of ACE inhibitors.

A growing body of evidence has accrued that control of systemic BP constitutes a crucial determinant of renal protection in chronic renal disease.[18] Therefore, we examined whether BP control affected the antiproteinuric action of these agents. Thus, the present study demonstrates that in patients with a final MAP <100 mm Hg, both ACE inhibitors and efonidipine decrease urinary protein excretion. However, even when the final MAP remains above 100 mm Hg, efonidipine possesses the ability to retain antiproteinuric action (Fig. 3). These findings suggest a renal-specific action of efonidipine, probably independent of systemic BP. Although the present study shows that ACE inhibitors do not decrease proteinuria in this setting (ie, final MAP >100 mm Hg), it has also been demonstrated that ACE inhibitors reduce proteinuria with no

decrease in BP in nondiabetic patients,[25] and possesses the beneficial action associated with BP-dependent and -independent mechanisms.[26] The failure to demonstrate antiproteinuric effect of ACE inhibitors in this study, therefore, may reflect the different patient population enrolled in this study (eg, diabetic and nondiabetic), as well as the number of patients enrolled.

The clinical utility of these agents merits comment. It has been reported that ACE inhibitors produce various adverse effects. In the present study, we noticed 43.3% of the patients treated with ACE inhibitors manifested a variety of undesirable effects, including cough and irritability of pharyngeal mucosa. Furthermore, anemia was present in 2 patients taking ACE inhibitors. Of importance, hyperkalemia developed in 5 patients on ACE inhibitor treatment. In contrast, only 10.5% of the efonidipine-treated patients manifested untoward effects, and no serious adverse effect was noticed in this group. In concert with the equipotent efficacy of efonidipine and ACE inhibitors on renal function, efonidipine appears better tolerated than ACE inhibitors in the treatment of hypertension with renal impairment. This premise, however, should be tested by further investigations.

In conclusion, our 48-week clinical study demonstrates that both efonidipine, a novel dihydropyridine calcium antagonist, and ACE inhibitors cause similar decreases in BP as well as proteinuria in hypertensive patients with renal impairment. The beneficial effect on proteinuria is particularly apparent in patients with proteinuria >1 g/day. The incidence of adverse effects, however, suggests that efonidipine is better tolerated than ACE inhibitors, with less incidence of undesirable action. The beneficial action of efonidipine with less side effects could facilitate the clinical use of this agent in the treatment of hypertension with chronic renal disease.

Acknowledgments

We are grateful for the following investigators who have contributed significantly to this work: Drs. Yuji Nara, Yasuhiko Iwamoto, Chieko Takahashi, Shigeko Hara, Kan Nagao, Naoto Matsuno, Toshikazu Degawa, Ensei Mizuiri, Ken Sakai, Takashi Ida, Hiromi Seto, Shigeru Aida, Waichi Kitajima, Kazuhiro Kumagai, Hirohiko Tsuganezawa, Toshio Shinoda, Masayuki Takayama, Masakazu Otsuka, Terukuni Ideura, Toyokazu Eguchi, Hiroshige Katsumata, Akira Yamauchi, Hirotsugu Akashi, Yukihiko Shimada, Issei Momma, Takayuki Kanai, Shizuho Kaname, Yasufumi Masuda, Tsuneo Murasawa, Tomoko Kanamaru, Takashi Ota, Hidetaka Seguchi, Satoru Komatsumoto, Masahiro Iyori, Akiyasu Tsuchida, Yasushi Asano, Sumiko Homma, Tatsuya Kutsuwada, Akihito Shimada, Yoshiro Sudo, Satoru Iwabuchi, Tatsuya Nakatsuka, Setsuo Kumazaki, Kimimasa Hayashi, Toshiki Shimada, Matsuhiko Hayashi, and Ryuichi Kato.

References

1. Schulman NB, Ford CE, Hall WD, Blaufox MD, Simon D, Langford HG, Schneider KA: Prognostic value of serum creatinine and effect of treatment of hypertension on renal function: Results from the hypertension detection and follow-up program. Hypertension 1989;13(Suppl 1):I-80–I-93.
2. Samuelsson O, Wilheimsen L, Elmfeldt D, Pennert K, Wedel H, Wikstrand J, Berglund G: Pedictors of cardiovascular morbidity in treated hypertension: Results from the Primary Prevention Trial in Goeteborg, Sweden. J Hypertens 1985;3:167–176.
3. Lewis EJ, Hunsicker LG, Bain RP, Rohde RD, for the Collaborative Study Group: The effect of angiotensin converting enzyme inhibition on diabetic nephropathy. N Engl J Med 1993;329:1456–1462.
4. Maschio G, Alberti D, Janin G, Locatelli F, Mann JF, Motolese M, Ponticelli C, Ritz E, Zucchelli P: Effect of the angiotensin-converting-enzyme inhibitor benazepril on the progression of chronic renal insufficiency. The Angiotensin-Converting-Enzyme Inhibition in Progressive Renal Insufficiency Study Group. N Engl J Med 1996; 334:939–945.
5. Ruggenenti P, Perna A, Gherardi G, Gaspari F, Benini R, Remuzzi G: Renal function and requirement for dialysis in chronic nephropathy patients on long-term ramipril: REIN follow-up trial. Gruppo Italiano di Studi Epidemiologici in Nefrologia (GISEN). Ramipril Efficacy in Nephropathy. Lancet 1998;352:1252–1256.
6. Anderson PW, Zhang XY, Tian J, Correale JD, Xi XP, Yang D, Graf K, Law RE, Hsueh WA: Insulin and angiotensin II are additive in stimulating TGF-beta 1 and matrix mRNAs in mesangial cells. Kidney Int 1996;50:745–753.
7. Pelayo JC, Quan AH, Shanley PF: Angiotensin II control of the renal microcirculation in rats with reduced renal mass. Am J Physiol 1990;258:F414–F422.
8. Anderson S, Rennke HG, Brenner BM: Therapeutic advantage of converting enzyme inhibitors in arresting progressive renal disease associated with systemic hypertension in the rat. J Clin Invest 1986;77:1993–2000.
9. Carmines PK, Navar LG: Disparate effects of Ca channel blockade on afferent and efferent arteriolar responses to ANG II. Am J Physiol 1989;256:F677–F685.
10. Fleming JT, Parekh N, Steinhausen M: Calcium antagonists preferentially dilate preglomerular vessels of hydronephrotic kidney. Am J Physiol 1987;253:F1157–F1163.
11. Hayashi K, Nagahama T, Oka K, Epstein M, Saruta T: Disparate effects of calcium antagonists on renal microcirculation. Hypertens Res 1996;19:31–36.
12. Honda M, Hayashi K, Matsuda H, Kubota E, Tokuyama H, Okubo K, Takamatsu I, Ozawa Y, Saruta T: Divergent renal vasodilator action of L- and T-type calcium antagonists in vivo. J Hypertens 2001;19:2031–2037.
13. Fujiwara K, Hayashi K, Kanno Y, Takenaka T, Saruta T: Renal protective effects of efonidipine in partially nephrectomized spontaneously hypertensive rats. Clin & Exp Hypertens 1998;20:295–312.
14. Peterson JC, Adler S, Burkart JM, Greene T, Hebert LA, Hunsicker LG, King AJ, Klahr S, Massry SG, Seifter JL: Blood pressure control, proteinuria, and the progression of renal disease. The Modification of Diet in Renal Disease Study. Ann Intern Med 1995;123: 754–762.
15. Joint National Committee on Prevention, Detection, Evaluation, and Treatment of High Blood Pressure: The Sixth Report of the Joint National Committee on Prevention, Detection, Evaluation, and Treatment of High Blood Pressure. Arch Intern Med 1997;157: 2413–2446.
16. Guidelines Subcommittee: 1999 World Health Organization–International Society of Hypertension guidelines for the management of hypertension. J Hypertens 1999;17:151–183.

17. Matsuda H, Hayashi K, Arakawa K, Naitoh M, Kubota E, Honda M, Matsumoto A, Suzuki H, Yamamoto T, Kajiya F, Saruta T: Zonal heterogeneity in action of angiotensin-converting enzyme inhibitor on renal microcirculation: role of intrarenal bradykinin. J Am Soc Nephrol 1999;10:2272–2282.
18. Agodoa LY, Appel L, Bakris GL, Beck G, Bourgoignie J, Briggs JP, Charleston J, Cheek D, Cleveland W, Douglas JG, Douglas M, Dowie D, Faulkner M, Gabriel A, Gassman J, Greene T, Hall Y, Hebert L, Hiremath L, Jamerson K, Johnson CJ, Kopple J, Kusek J, Lash J, Lea J, Lewis JB, Lipkowitz M, Massry S, Middleton J, Miller ER 3rd, Norris K, O'Connor D, Ojo A, Phillips RA, Pogue V, Rahman M, Randall OS, Rostand S, Schulman G, Smith W, Thornley-Brown D, Tisher CC, Toto RD, Wright JT Jr, Xu S, for theAfrican American Study of Kidney Disease and Hypertension (AASK) Study Group: Effect of ramipril vs amlodipine on renal outcomes in hypertensive nephrosclerosis: a randomized controlled trial. JAMA 2001;285:2719–2728.
19. Maki DD, Ma JZ, Louis TA, Kasiske BL: Long-term effects of antihypertensive agents on proteinuria and renal function. Arch Intern Med 1995;155:1073–1080.
20. Zucchelli P, Zuccala A, Borghi M, Fusaroli M, Sasdelli M, Stallone C, Sanna G, Gaggi R: Long-term comparison between captopril and nifedipine in the progression of renal insufficiency. Kidney Int 1992;42:452–458.
21. Kumagai H, Hayashi K, Kumamaru Y, Saruta T: Amlodipine is comparable to angiotensin-converting enzyme inhibitor for long-term renoprotection in hypertensive patients with renal dysfunction: A one-year, prospective, randomized study. Am J Hypertens 2000; 13:980–985.
22. Williams PS, Fass G, Bone JM: Renal pathology and proteinuria determine progression in untreated mild/moderate chronic renal failure. Q J Med 1988;67:343–354.
23. Masumiya H, Kase J, Tanaka Y, Tanaka H, Shigenobu K: Frequency-dependent blockade of T-type Ca^{2+} current by efonidipine in cardiomyocytes. Life Sci 2000;68:345–351.
24. Wagner C, Kramer BK, Hinder M, Kieninger M, Kurtz A: T-type and L-type calcium channel blockers exert opposite effects on renin secretion and renin gene expression in conscious rats. Br J Pharmacol 1998;124:579–585.
25. Ciavarella A, Mustacchio A, Silletti A, Franchi R, Levorato M, Campieri C, Borgnino LC, Capozzi G, Morotti L, Vannini P: Low-dose angiotensin converting enzyme inhibitors: effect on renal function in normo- and hypertensive type 1 diabetic patients. Eur J Med 1992;1:268–272.
26. Jafar TH, Schmid CH, Landa M, Giatras I, Toto R, Remuzzi G, Maschio G, Brenner BM, Kamper A, Zucchelli P, Becker G, Himmelmann A, Bannister K, Landais P, Shahinfar S, de Jong PE, deZeeuw D, Lau J, Levey AS: Angiotensin-converting enzyme inhibitors and progression of non-diabetic renal disease. A meta-analysis of patient-level data. Ann Intern Med 2001;135:73–87.
